# Modular, stereocontrolled C_β_–H/C_α_–C activation of alkyl carboxylic acids

**DOI:** 10.1073/pnas.1903048116

**Published:** 2019-04-17

**Authors:** Ming Shang, Karla S. Feu, Julien C. Vantourout, Lisa M. Barton, Heather L. Osswald, Nobutaka Kato, Kerstin Gagaring, Case W. McNamara, Gang Chen, Liang Hu, Shengyang Ni, Paula Fernández-Canelas, Miao Chen, Rohan R. Merchant, Tian Qin, Stuart L. Schreiber, Bruno Melillo, Jin-Quan Yu, Phil S. Baran

**Affiliations:** ^a^Department of Chemistry, The Scripps Research Institute, La Jolla, CA 92037;; ^b^Chemical Biology and Therapeutics Science Program, Broad Institute, Cambridge, MA 02142;; ^c^Biology Department, Calibr at The Scripps Research Institute, La Jolla, CA 92037;; ^d^Department of Chemistry and Chemical Biology, Harvard University, Cambridge, MA 02138

**Keywords:** C–H activation, decarboxylative cross-coupling, modular, stereocontrolled, carboxylic acids

## Abstract

The union of two powerful transformations, directed C–H activation and decarboxylative cross-coupling, for the enantioselective synthesis of vicinally functionalized alkyl, carbocyclic, and heterocyclic compounds is described. Starting from simple carboxylic acid building blocks, this modular sequence exploits the residual directing group to access more than 50 scaffolds that would be otherwise extremely difficult to prepare. The tactical use of these two transformations accomplishes a formal vicinal difunctionalization of carbon centers in a way that is modular and thus, amenable to rapid diversity incorporation. A simplification of routes to known preclinical drug candidates is presented along with the rapid diversification of an antimalarial compound series.

## Significance

The combination of two newly emerging methods for chemical synthesis enables access to molecular space that was previously challenging or impossible to access. Thus, a C–H activation of ubiquitous carboxylic acids followed by their decarboxylative functionalization provides modular access to difunctionalized carbon frameworks with distinctly controlled stereochemistry. Application of this strategy to simplify the synthesis of medicinally important entities and to discover potent antimalarial compounds is described.

**Fig. 1.**
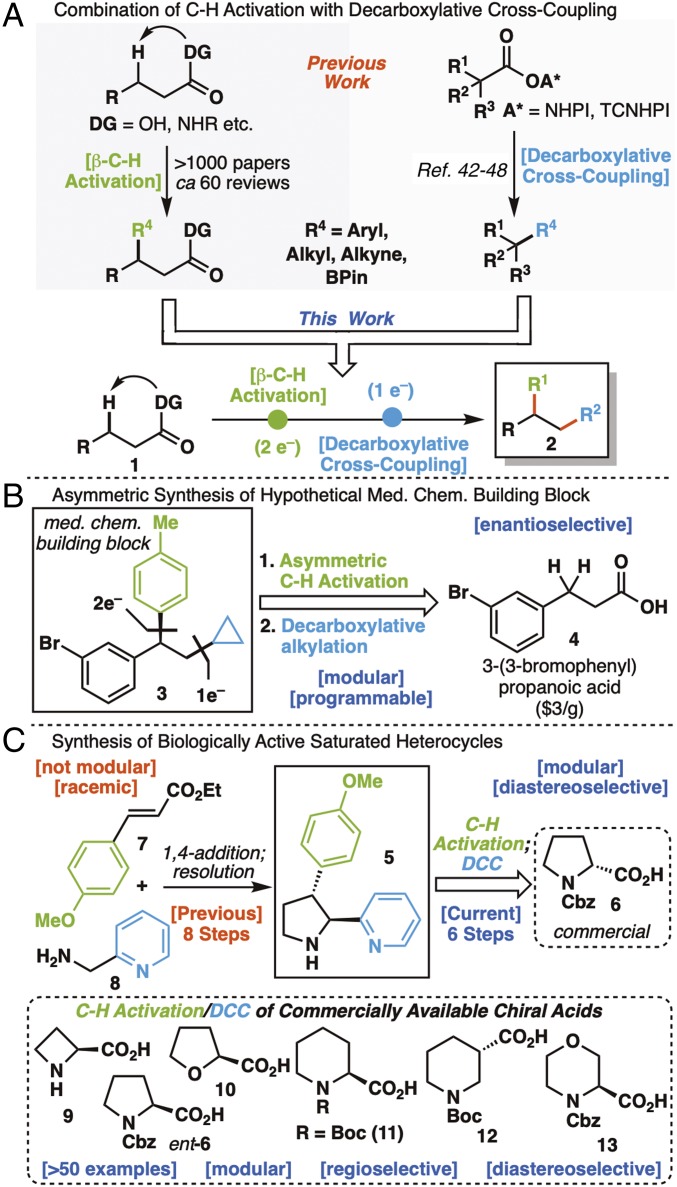
Introduction to the modular, stereocontrolled C_β_–H/C_α_–C activation of alkyl carboxylic acids. (*A*) Combination of C–H activation with decarboxylative cross-coupling. (*B*) Asymmetric synthesis of hypothetical medicinal chemistry (Med. Chem.) building block. (*C*) Synthesis of biologically active saturated heterocycles.

It is becoming increasingly clear that practitioners are no longer bound by the notion that the pervasive C–H bond is unresponsive to manipulation. In fact, the past two decades have seen a dramatic increase in the use of C–H functionalization logic (1–3) to assemble molecules (4–16). At this juncture, it can be considered part of the mainstream in terms of the way that students learn retrosynthetic analysis (17, 18). One of today’s workhorse C–H activation strategies involves the use of native functional groups to direct and guide the site of functionalization (19–24). As the most ubiquitous functional group in organic chemistry, carboxylic acids and their derivatives have naturally risen to the top in terms of directed C–H functionalization reactions available to the practitioner (Fig. 1*A*) (25–29). With over 1,000 reports now present for the use of such guided C–H activations (1–3) in synthesis, it is fair to say that this is a staple reaction manifold for modern organic synthesis. In this context, an exploration of serial reactivity in which the lingering carboxylate group is used in successive reactions has been limited in scope. Of the few notable examples, nearly all are restricted to restoration of the parent carboxylic acid followed by classic reactions, such as amidation and esterification (Fig. 1*A*) (30, 31). The recent development of robust methods to decarboxylate such systems and programmably replace them with new C–C and C–B bonds in a stereochemically predictable way, a formal type of C–C activation, opens opportunities to leverage the power of carboxylate-directed C–H activation chemistry. This combination of one- (32) and two-electron disconnections would enable pathways to potentially valuable chiral acyclic building blocks, such as **3**, that could be considered “retrosynthetically opaque,” as it is not immediately apparent how a simple building block, like 3-(3-bromophenyl) propanoic acid (**4**), could be used as its precursor (Fig. 1*B*) (33). Within the privileged realm of saturated cyclic heterocycles, such logic could be used to rapidly access libraries of enantiopure scaffolds that would be rather difficult to otherwise prepare (Fig. 1*C*) (34–37). For example, chiral pyrrolidines, such as **5**, have previously been prepared through labor-intensive routes that require chiral resolution and are not amenable to late-stage diversity incorporation (38, 39). In stark contrast, a combination of C–H activation and radical cross-coupling strategies (33) could access the same architectures in fewer steps with exquisite control of stereochemistry and allow for diverse arenes to be installed at the end of the route starting from simple commercial carboxylic acids. The difficulty in preparing such seemingly simple molecules is directly related to the challenge of “escaping the flatland” as articulated by many in the field (40, 41). Herein, we present a strategy for the net vicinal difunctionalization of cyclic and acyclic systems via sequential functionalization initiated by stereoselective C–H activation followed by decarboxylative cross-coupling (dCC) to form a variety of C–C and C–X bonds, including aryl (42, 43), alkenyl (44), alkynyl (45), alkyl (26, 46), and boryl (47, 48). The inherent modularity of this strategic advance allows access to a wealth of acyclic and cyclic systems, some of which have been prepared before in more laborious ways. Application to a promising series of heretofore inaccessible azetidine-based antimalarial agents is also disclosed.

## Results and Discussion

### Proof of Concept.

**Fig. 2.**
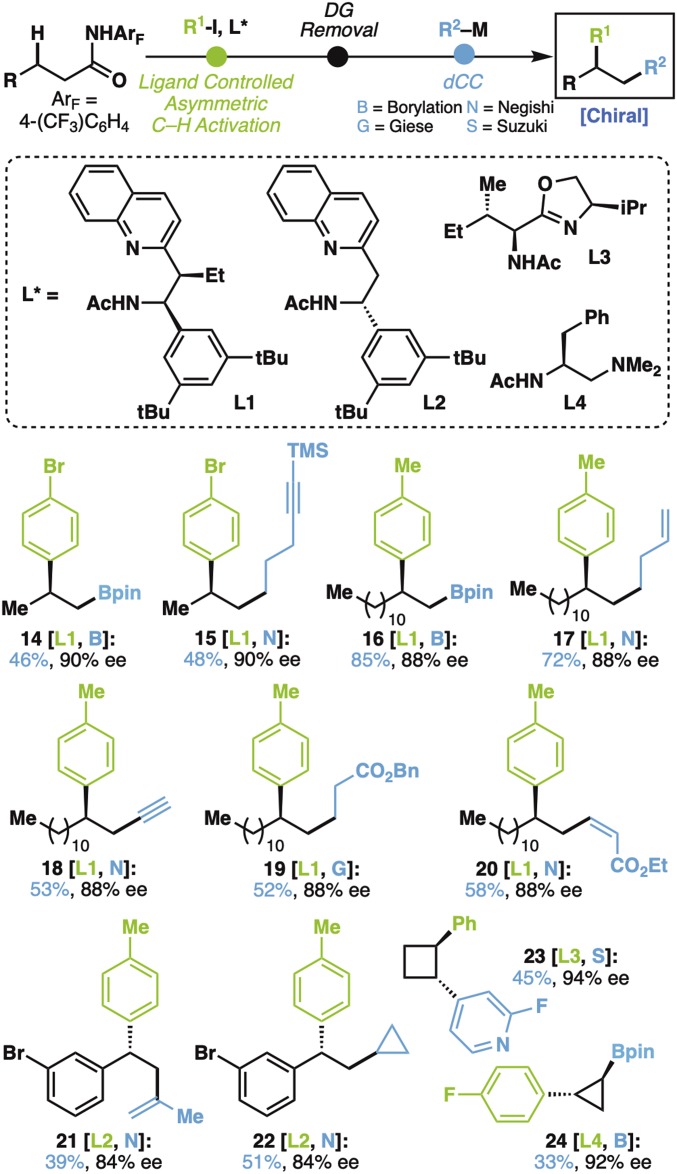
Proof of concept for the C_β_–H/C_α_–C activation strategy; enantiomeric excess (ee) were measured after the C–H activation step. General conditions for C–H activation reaction (*SI Appendix* has details): amide (1 eq), Aryl-I (2 eq), Pd(OAc)_2_ (10 mol %), ligand L* (12 mol %), Ag_2_CO_3_ (2 eq), hexafluoroisopropanol (HFIP) (0.1 M), 80 °C, 36 h. General conditions for directing group removal (*SI Appendix* has details): arylated amide (1 eq), Et_2_O•BF_3_ (35 eq), MeOH (0.025 M), 100 °C, 12 h; then, LiOH•H_2_O (2 eq), THF:H_2_O = 1:1, 0 °C, 1 h. General conditions for dCC reaction (*SI Appendix* has details): [**N**] TCNHPI ester (0.1 mmol, 1 eq), zinc reagent (0.2 mmol, 2 eq), NiCl_2_•glyme (30 mol %), ditBuBipy (60 mol %), THF:*N,N*-dimethyformamide (DMF) = 3:2, room temperature (rt), 12 h. [**S**] TCNHPI ester (0.1 mmol, 1 eq), boronic acid (0.3 mmol, 3 eq), NiCl_2_•6H_2_O (30 mol %), Bathophenantroline (30 mol %), Et_3_N (1 mmol, 10 eq), 1,4-dioxane:DMF = 10:1, 75 °C, 12 h. [**G**] NHPI ester (0.1 mmol, 1 eq), Michael acceptor (0.2 mmol, 2 eq), Ni(ClO_4_)_2_•6H_2_O (20 mol %), Zn powder (0.2 mmol, 2.0 eq), LiCl (0.3 mmol, 3 eq), MeCN, rt, 24 h. [**B**] TCNHPI ester (0.1 mmol, 1 eq), [B_2_Pin_2_Me]Li (0.33 mmol, 3.3 eq), NiCl_2_•6H_2_O (20 mol %), diOMeBipy (26 mol %), MgBr_2_•OEt_2_ (0.15 mmol, 1.5 eq), THF, rt, 2 h. DG, directing group.

To obtain a first proof of concept for the underlying strategy, a set of enantiopure carboxylic acids, prepared using Pd-catalyzed ligand-enabled asymmetric sp^3^ C–H activation, was used (Fig. 2) (49–51). Recalling the suite of dCC reactions developed over the last several years (52), the succeeding reaction was found to be tolerant of a variety of pendant functional groups, including esters (**19** and **20**) and boronic esters (**14** and **16**), as well as both free (**18**) and silyl-capped alkynes (**15**), all of which can be used in yet another reaction sequence on liberation. Importantly, pyridine and boron can be incorporated in the challenging context of strained carbocycles to access *trans*-disubstituted cyclobutane (**23**) and cyclopropane (**24**) rings with high enantiomeric purity, the latter of which could be conducted without a directing group. The ligand-controlled nature of the C–H activation step allows for easily tunable access to either desired enantiomer (i.e., **14** vs. **21**) (49–51).

### Scope of Saturated Heterocycles.

**Fig. 3.**
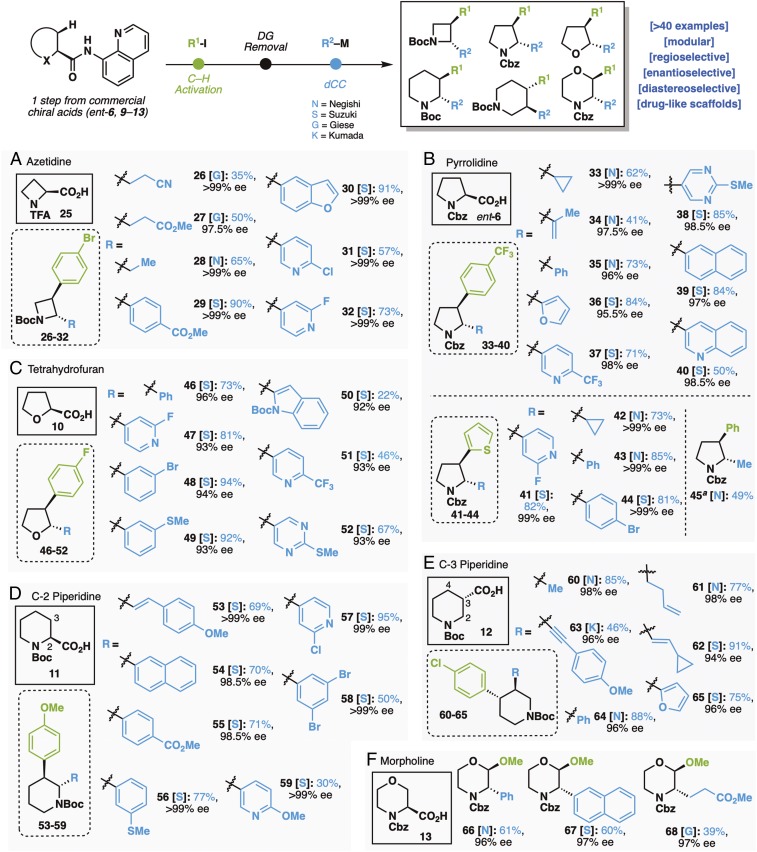
Scope of saturated heterocycles. (*A*) Azetidine core; (*B*) pyrrolidine core; (*C*) tetrahydrofuran core; (*D*) C-2 piperidine core; (*E*) C-3 piperidine core; (*F*) morpholine. General conditions for C–H activation reaction (*SI Appendix* has details): amide (1 eq), Aryl-I (3 eq), Pd(OAc)_2_ (10 mol %), and AgOAc (2 eq), 110 °C, 38 h. General conditions for directing group removal (*SI Appendix* has details): arylated amide (1 eq), Boc_2_O (20 eq), 4-dimethylaminopyridine (DMAP) (3 eq), MeCN (1 M), 70 °C, 12 h. Then, LiOH•H_2_O (2 eq), 30% H_2_O_2_ (5.0 eq), THF:H_2_O = 3:1, 0 °C to room temperature (rt), 18 h. General conditions for dCC reaction (*SI Appendix* has details): [**N**] TCNHPI ester (0.1 mmol, 1 eq), zinc reagent (0.2 mmol, 2 eq), NiCl_2_•glyme (10–50 mol %), di*t*BuBipy (20–60 mol %), THF:*N,N*-dimethylformamide (DMF) = 3:2, rt, 12 h. [**S**] TCNHPI ester (0.1 mmol, 1 eq), boronic acid (0.3 mmol, 3 eq), NiCl_2_•6H_2_O (20–50 mol %), Bathophenantroline (22–60 mol %), Et_3_N (1 mmol, 10 eq), 1,4-dioxane:DMF = 10:1, 75 °C, 12 h. [**G**] TCNHPI ester (0.1 mmol, 1 eq), Michael acceptor (0.2 mmol, 2 eq), Ni(ClO_4_)_2_•6H_2_O (20 mol %), Zn powder (0.2 mmol, 2.0 eq), LiCl (0.3 mmol, 3 eq), MeCN, rt, 24 h. [**K**] TCNHPI ester (0.1 mmol, 1 eq), Grignard reagent (0.15 mmol, 1.5 eq), FeBr_2_•H_2_O (20 mol %), NMP, −15 °C, 15 min. ^a^No ee reported for this example, as racemic compound was not prepared. DG, directing group.

In acknowledgment of the increasing demand for saturated heterocycles in drug development (53, 54), the efficacy of this reaction sequence was demonstrated using an array of commercial heterocyclic acids (both enantiomers of each are available) as shown in Fig. 3. Beyond their importance as a framework for the celebrated β-lactam therapeutic class (55–58), azetidines have garnered recent interest as scaffolds in diversity-oriented synthesis, through which a wide variety of constrained (i.e., bridged or fused) or densely substituted nitrogenous ring systems can be accessed (59). To this point, arylated intermediates derived from *N*-protected azetidine-2-carboxylic acid (Fig. 3*A*) can be rapidly diversified under the Suzuki (**29**–**32**), Negishi (**28**), and Giese (**26** and **27**) protocols. In a similar vein, elaboration of Cbz-protected proline via directed C–H arylation at C3 followed by dCC furnished a range of enantiopure *trans*-1,2-difunctionalized pyrrolidines bearing diverse substituents, such as aryl (**35** and **39**), heteroaryl (**36**–**38** and **40**), cycloalkyl (**33**), and alkenyl (**34**) functionalities (Fig. 3*B*). Substrate **45** is of particular note, as prior routes to access such scaffolds involved early-stage incorporation of methyl and phenyl substituents and a tedious separation of diastereomers (60). Fig. 3*C* features the same titular sequence as applied to a THF core to furnish a variety of enantiopure THF-based building blocks (**46**–**52**). Among the privileged *N*-heterocyclic fragments, piperidine remains prevalent, with disubstitution identified as the most common decorative pattern (53, 54). Here, commercially available *N*-Boc-l-pipecolic acid (Fig. 3*D*) first furnishes the 3-(4-methoxyphenyl) analog, after which the hydrolysis/dCC sequence provides a variety of 2,3-*trans*-substitutions, including fused aryl (**54**), heteroaryl (**57** and **59**), and alkenyl (**53**) moieties at C2. All of these structures are new chemical entities despite their simplicity—even distant relatives are rare. It is difficult to conceive of a more direct and modular approach to such scaffolds with either enantiomeric form available simply by choosing l or d forms of pipecolic acid. When 3,4-piperidine substitution is desired, *N*-Boc*-*piperidine-3-carboxylic acid (Fig. 3*E*) may be used (**60**–**65**), as the initial C–H activation takes place selectively at C–4 vs. C–2. Given the structural similarity to the blockbuster drug Paxil (paroxetine), rapid access to such systems is noteworthy. Finally, the logic outlined above can be applied to substituted morpholines (53, 54)—one of the rare saturated, bis-heteroatom–containing systems to top frequency lists in Food and Drug Administration approvals—as detailed in Fig. 3*F*. Although the C–H activation step is limited to methoxylation at this juncture (*SI Appendix* discusses attempted C–H arylation and methylation), it does represent an example of C–H activation of such heterocycle.

### Synthesis of Hit-to-Lead Candidates and Late-Stage Intermediates.

**Fig. 4.**
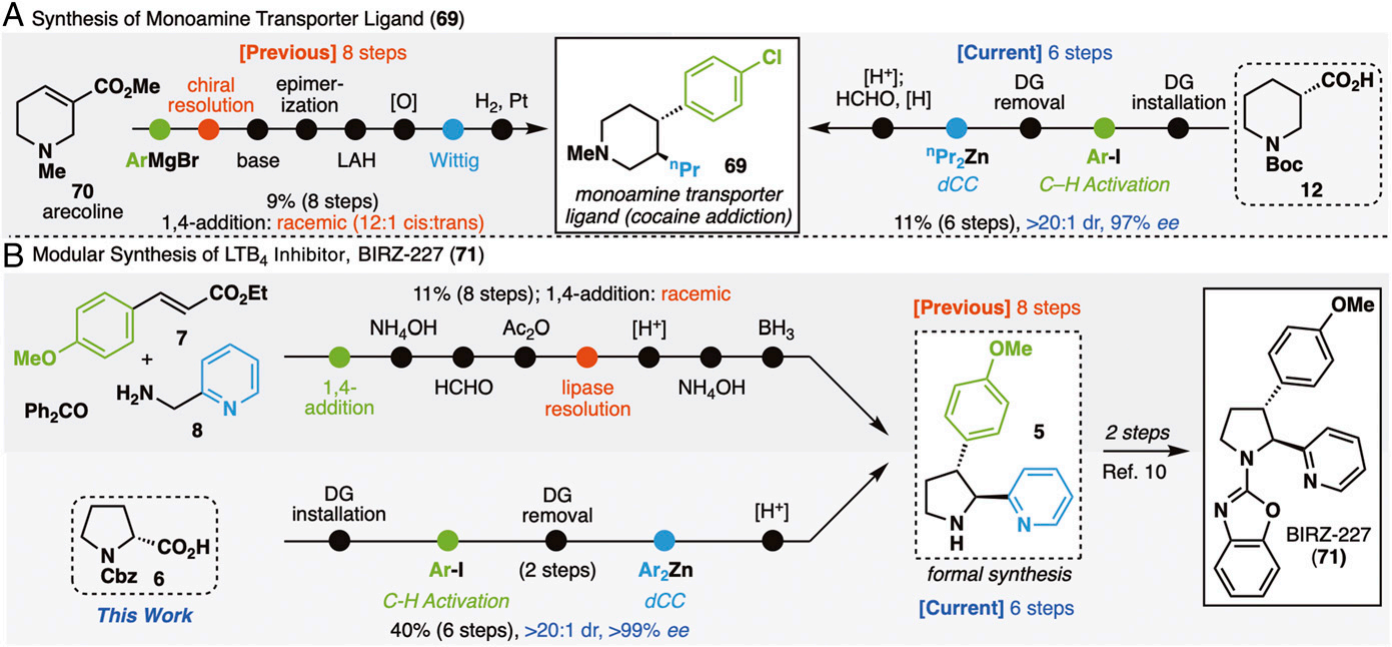
Synthesis of hit-to-lead candidates and late-stage intermediates. (*A*) Synthesis of monoamine transporter ligand (**69**); (*B*) modular synthesis of LBT4 inhibitor, BIRZ-227 (**71**). DG, directing group.

In addition to the diverse scope outlined above, the described reaction series was next evaluated for its capacity to simplify the synthesis of active hit-to-lead series and late-stage intermediates (Fig. 4). Of particular note in these case studies is how the logic presented herein can be used as both a means to effectively access a specific target or a library of similar structures simply by changing coupling partners. Monoamine transporter ligand **69** is a vivid demonstration of this (61, 62). The first synthesis of this molecule utilized conjugate addition to arecoline **70** followed by a series of functional group manipulations to arrive at **69** in 8.6% overall yield after chiral resolution (63). Use of the current vicinal difunctionalization strategy deleted many of those concession steps and could be used to access **69** (11.2% overall yield; >20:1 diastereomeric ratio; 97% enantiomeric excess) and in principle, a whole library of enantiopure analogs in only six steps. The leukotriene B_4_ inhibitor BIRZ-227 (Fig. 4*B*) (**71**), a *trans*-diarylpyrrolidine, is another prime example of how customary logic falters if a diverse, modularly assembled library is targeted. Indeed, the sole reported preparative-scale protocol to its precursor **5** opts for a pyrrolidinone construction in the initial step, placing severe limitations for rapid diversification (37, 38). To access enantiopure intermediates, an enzyme-assisted resolution is required, which itself requires multiple extraneous steps. Instead, the sequential C–H arylation/dCC approach begins from an inexpensive enantiopure amino acid building block (Pro), which is subjected to standard directing group installation, followed by C–H arylation/hydrolysis and the desired dCC to arrive at diarylated compound **5**. Should further diversification be of interest, any number of analogs may be forged in short order, with no resolution necessary.

### Structural Diversification of Azetidines with in Vitro Antimalarial Activity.

**Fig. 5.**
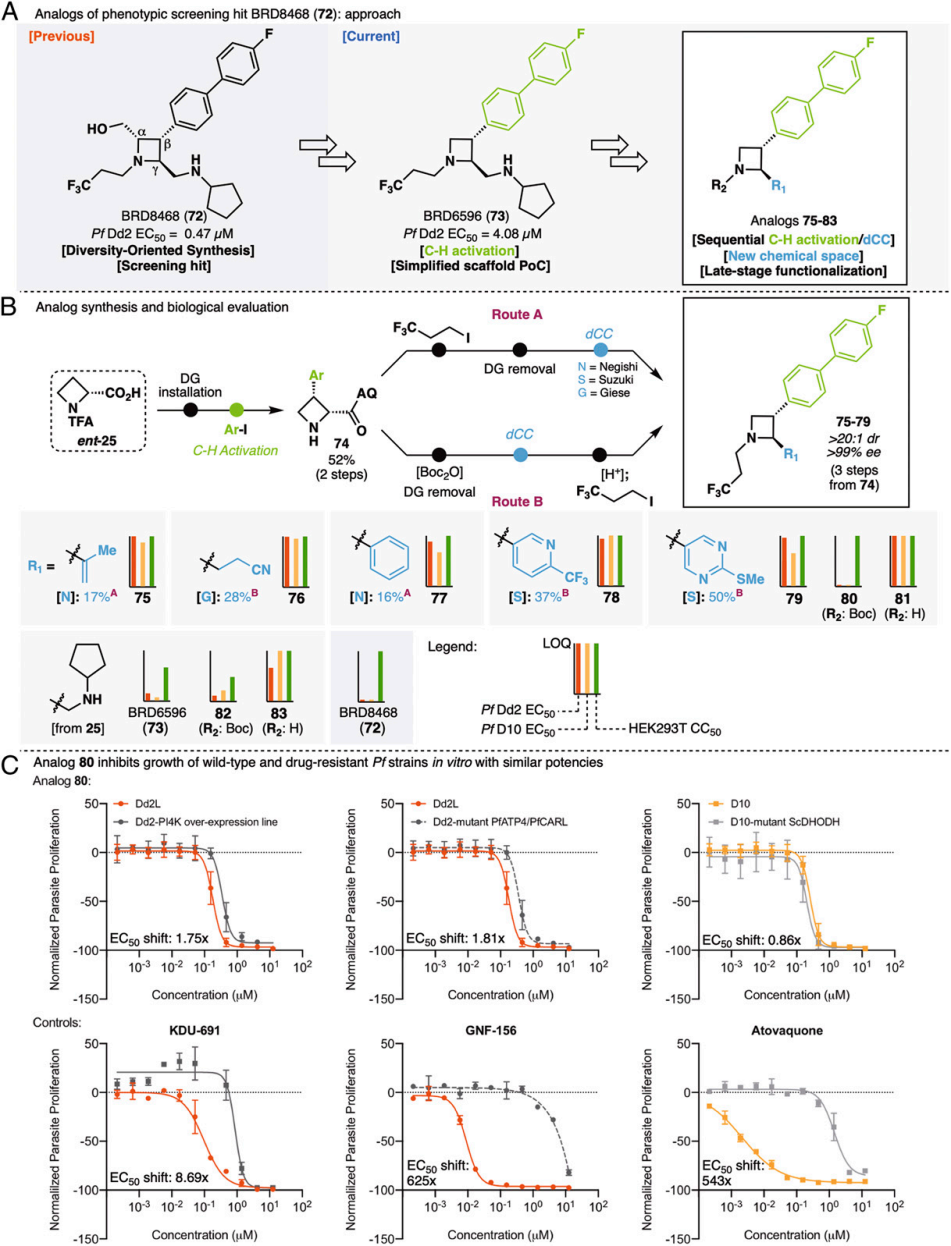
Structural diversification of azetidines with in vitro antimalarial activity. (*A*) Analogs of phenotypic screening hit BRD8488 (**72**): approach; (*B*) analog synthesis and biological evaluation; (*C*) analog 80 inhibits growth of wild-type and drug-resistant *Pf* strains in vitro with similar potencies. DG, directing group.

We finally applied the sequential functionalization tactic to the design and synthesis of azetidine-containing small molecules that potently inhibit the asexual blood stages of *Plasmodium* parasites. *Plasmodium* infections continue to cause over 200 million clinical cases of malaria each year, leading to an estimated 435,000 deaths in 2017 (64). Emerging resistance to frontline drugs, an issue currently curbed by means of combination therapies (64, 65), underscores the need for antimalarials that act via novel mechanisms of action (nMoA). Recently, Kato et al. (66) reported the discovery of potent nMoA antimalarials enabled by phenotypic high-throughput screening (see Malaria Therapeutics Response Portal, https://portals.broadinstitute.org/mtrp/). An unpursued promising hit from the same screening campaign is the trisubstituted azetidine BRD8468 (**72**; Malaria Therapeutics Response Portal) (Fig. 5*A*) (Malaria Therapeutics Response Portal). Importantly, BRD8468 may inhibit parasite growth via nMoA, because it remains equipotent against a panel of drug-resistant lines that have been used to identify compounds acting through known mechanisms of action, such as inhibitors of *Plasmodium falciparum* (*Pf*) ATP4, *Pf*PI4K, and various targets in the mitochondrial electron transport chain, like *Pf*DHODH (*SI Appendix*) (*Pf* is the deadliest species of *Plasmodium* that causes malaria in humans). Also of note, C_γ_-epimer BRD5530 showed significantly lower potency than BRD8468 (up to 25-fold) (*SI Appendix*), indicating in turn, that the *trans*-relationship between the biaryl substituent and the vicinal alkyl group is critical for activity. In this context, we envisioned a structural simplification of BRD8468 consisting of the removal of the hydroxymethyl group at C_α_. If successful (i.e., conducive to potent analogs), this strategy would enable both (*i*) significant abbreviation of synthetic routes, rendering the chemical series more attractive in terms of developability [guidelines on antimalarial development, including recommended maximal cost of goods, are in Burrows et al. (67)] and (*ii*) late-stage exploration of chemical space via the sequential C–H activation/dCC tactic described above. The Diversity-Oriented Synthesis compound collection that originally included BRD8468 **72** was constructed via functionalization of a C_γ_-nitrile (21) (*SI Appendix*)—the ability to instead perform dCC at C_γ_ would, therefore, allow the preparation of a host of previously inaccessible analogs. To establish proof of concept, we first synthesized disubstituted azetidine **73** (Fig. 5*A*). As expected, this could be achieved in four steps from C–H arylation product **74** (Fig. 5*B*). Encouragingly, in vitro evaluation of **73** showed that the structural simplification only led to a modest loss in potency (fourfold in *Pf*D10) relative to parent compound **72**, and no significant shift in potency was observed on treatment of drug-resistant strains compared with their wild-type counterparts. Given this positive preliminary result, two synthetic routes were designed to rapidly access simplified, structurally novel C_γ_ analogs of **72** (Routes A and B in Fig. 5*B*). The former would enable late-stage diversification at C_γ_; the latter would be optimal for diversification at nitrogen, although outside the scope of this study. In practice, Route A led to vinyl and aryl analogs (**75** and **77**, respectively) (Fig. 5*B*) via Negishi-type dCC, albeit in low yield. Route B, in which the dCC step is performed on a Boc-protected azetidine intermediate, allowed higher-yielding dCC. Suzuki-dCC readily led to pyridine **78** and pyrimidine **79**; Giese-dCC, in turn, gave rise to nitrile **76**. Evaluation in vitro of these *N*-trifluoroalkyl analogs revealed inhibition of *Pf* growth with only micromolar potencies (Fig. 5*B*; details are in *SI Appendix*, Table S11), with pyrimidine **79** being one of the more active congeners (*Pf*D10 EC_50_ = 10.3 µM; *Pf*Dd2 EC_50_ = 12.3 µM). Generally, no effect was observed on human cell line HEK293T at concentrations below 20 µM, suggesting that these compounds lack overt toxicity in human cells. Given that the parent scaffold (BRD8468; **72**) had previously been shown to tolerate modifications at the azetidine nitrogen (Malaria Therapeutics Response Portal), we also evaluated **80** and **81**, *tert*-butyl-carbamate and *N*-unsubstituted analogs (and synthetic intermediates), respectively, of **79**. For reference, we introduced similar modifications on BRD6596 **73**, leading, in turn, to carbamate **82** and *N*-unsubstituted azetidine **83**. Potencies varied following the same trend in both series of *N*-substitutions, with EC_50_ values decreasing in the order H > alkyl > carbamate (Fig. 5*B*). We were, however, delighted to find that C2-pyrimidine analog **80** [*Pf*D10 EC_50_ = 0.27 µM; *Pf*Dd2 EC_50_ = 0.17 µM; HEK293T cytotoxic concentration 50 (CC_50_) > 20 µM], accessible in short order via sequential C–H arylation and dCC, exhibited superior potency and selectivity relative to both BRD6596 (**73**; *Pf*D10 EC_50_ = 1.62 µM; *Pf*Dd2 EC_50_ = 4.08 µM; HEK293T CC_50_ = 16.7 µM) and the original screening hit BRD8468 (**72**; *Pf*D10 EC_50_ = 0.38 µM; *Pf*Dd2 EC_50_ = 0.47 µM; HEK293T CC_50_ > 20 µM). Finally, we demonstrated that **80** retains activity against drug-resistant *Pf* strains (Fig. 5*C* and *SI Appendix*, Table S12). Specifically, **80** remained equipotent against a *Pf*PI4K overexpression line, a *Pf*CARL/*Pf*ATP4 mutant, and transgenic *Pf*-expressing *Sc*DHODH (less than two times EC_50_ shifts relative to wild-type parasites). In good agreement with previously reported data, these parasites were resistant to KDU-691 (8.7× EC_50_ shift) (68), GNF-156 (625× EC_50_ shift) (69), and atovaquone (543× EC_50_ shift) (70), respectively. As mentioned previously, these observations suggest that **80** may inhibit parasite growth via nMoA and therefore, warrant additional study. To this end, additional structural modifications at C_γ_ and nitrogen and their impact on antimalarial activity are now being investigated. Although certainly preliminary, the encouraging results reported here demonstrate how the C–H activation/dCC tactic may easily and productively augment the scope of medicinal chemistry campaigns.

### Limitations.

The strategy presented herein is not without limitations. Foremost among these is the unfortunate requirement for directing group installation and removal, as the free carboxylates can only be utilized for cyclopropane substrates (e.g., **24**). For example, despite extensive efforts, piperazine heterocycles are completely inert to C–H activation attempts (*SI Appendix* has details). In addition, when the C–H alkynylation, alkoxylation, and fluorination of nitrogen-based saturated heterocycles were successfully achieved, the subsequent removal of the directing group has proven challenging. A *trans*-relationship between vicinal substituents is also an implicit limitation of the sequence. Finally, it is worth noting that, although an asymmetric conjugate addition strategy could be envisaged to access acyclic (Fig. 2) and C–3 piperidine systems (Fig. 3*E*), the remaining scaffolds would be difficult to obtain, as asymmetric conjugate addition to heterocyclic enamides and enol ethers is poorly developed.

### Conclusion.

The tactical combination of two powerful, recently invented transformations (carboxylate-guided C–H activation and dCC) can thus be brought to bear to simplify the preparation of useful enantiopure building blocks (71). This formal vicinal difunctionalization permits the modular installation of an almost limitless variety of *trans*-1,2-disubstituted frameworks bearing aryl, alkyl, ether, and boron functional groups. Two distinct applications of these combined polar/radical retrosynthetic disconnections illustrate the power of this approach. Finally, the logic outlined herein shows promise in accessing a generation of antimalarial leads based on a diverse library of azetidine scaffolds.

## Materials and Methods

All reagents were commercially available and used as supplied without additional purification. Solvents were obtained by passing the previously degassed solvents through an activated alumina column. The details of the materials, methods (including synthesis and characterization of compounds), and reaction optimizations are described in *SI Appendix*.

### Note.

During the preparation of this manuscript, an elegant combination of C–H activation and dCC was instituted by Reisman and coworkers (71) for the synthesis of cyclobutene-based natural products and related molecules.

## Supplementary Material

Supplementary File
