# Supplementary material for: Modular, stereocontrolled Cβ–H/Cα–C activation of alkyl carboxylic acids
Source: Proc Natl Acad Sci U S A. 2019 Apr 17;116(18):8721–7. doi: 10.1073/pnas.1903048116 (PMC6500144; doi:10.1073/pnas.1903048116)
Supplement: Supplementary File [file pnas.1903048116.sapp.pdf]

Supplementary Information for

Modular, Stereocontrolled C_β–H/C_α–C Activation of Alkyl Carboxylic Acids

Ming Shang[⊥,†], Karla S. Feu[⊥,†], Julien C. Vantourout[†], Lisa M. Barton[†], Heather L. Osswald[†], Nobutaka Kato[§], Kerstin Gagaring[‡], Case W. McNamara[‡], Gang Chen[†], Liang Hu[†], Shengyang Ni[†], Paula Fernández-Canelas[†], Miao Chen[†], Rohan R. Merchant[†], Tian Qin[†], Stuart L. Schreiber[‖,§], Bruno Melillo[*,†,§], Jin-Quan Yu[*,†], Phil S. Baran[*,†]

[†]Department of Chemistry, Scripps Research, 10550 North Torrey Pines Road, La Jolla, California 92037

[‡]California Institute for Biomedical Research (Calibr), La Jolla, California 92037

[§]Chemical Biology and Therapeutics Science Program, Broad Institute, Cambridge, Massachusetts 02142

[‖]Department of Chemistry and Chemical Biology, Harvard University, Cambridge, Massachusetts 02138

Corresponding authors:

Phil S. Baran: pbaran@scripps.edu

Jin Q. Yu: yu200@scripps.ed

Bruno Melillo: bmelillo@scripps.edu

www.pnas.org/cgi/doi/10.1073/pnas.1903048116

Table of contents

Table of contents **2**

General Experimental **10**

General procedures **11**

General Procedure A1: Asymmetric sp^3 C–H arylation sequence For Linear and Strained-Ring Systems **11**

A1.1: Installation of Directing Group 11

A1.2: C–H Activation 12

A1.3: Removal Directing Group 13

A1.4: C–H Activation - free carboxylic acid 15

A1.5 Decarboxylative Borylation using copper catalyst 16

General Procedure A2: sp^3 C–H Arylation sequence for Azetidine Scaffolds **17**

A2.1: Installation of Directing Group 17

A2.2: C–H Activation 18

A2.3: Removal Directing Group 19

General Procedure A3: sp^3 C–H Arylation Sequence for Pyrrolidine, Tetrahydrofuran and Piperidine Scaffolds **20**

A3.1: Installation of Directing Group 20

A3.2: C–H Activation 21

A3.3: Removal Directing Group 22

Graphical Supporting Information for Pyrrolidine, Piperidine and Tetrahydrofuran Scaffolds 24

General Procedure A4: sp^3 C–H Methoxylation Sequence for Morpholine Scaffold **28**

A4.1: Installation of Directing Group 28

A4.2: C–H Activation 29

A4.3: Removal Directing Group and synthesis of RAE 30

Graphical Supporting Information for Morpholine Scaffold (General Procedure A4) 31

Table S1: Optimization of C–H Methoxylation for Morpholine Scaffold 37

General Procedure B: Synthesis of Redox-Active Esters 38

Photographic Guide for Redox-active Ester Formation (General Procedure B) 40

General Procedure C1: Nickel-Catalyzed Boronic Acid Suzuki Cross-Coupling 41

Preparation of $NiCl_2$•$6H_2O$/ligand Stock Solution (0.1 M in DMF) 41

Graphical Supporting Information for Preparation of $NiCl_2$•$6H_2O$/Bathophenantroline 42

Procedure for the Suzuki Ni-Catalyzed Decarboxylative Cross-Coupling 43

Graphical Supporting Information for Suzuki type reaction 45

General Procedure C2: Nickel-catalyzed Negishi Alkylation 46

Preparation of Alkyl Zinc Reagents from Alkyl Bromides 46

Procedure for the Ni-catalyzed Decarboxylative Alkylation 47

General Procedure C3: Nickel-catalyzed Negishi Alkenylation 48

Synthesis of Alkenylzinc Reagents from Alkenyl Grignard Reagents 48

Procedure for the Ni-catalyzed Decarboxylative Alkenylation 50

General Procedure C4: Nickel-catalyzed Negishi Arylation 51

Preparation of $ZnCl_2$ Solution (1.0 M in THF) 51

Preparation of the Arylzinc Reagents 51

Titration of Arylzinc Reagents 52

Procedure for the Ni-catalyzed Decarboxylative Arylation 53

General Procedure C5: Nickel-catalyzed Negishi Ethynylation 54

Preparation of Nickel/ligand Solution 54

Preparation of Ethynylzinc Chloride Solution 54

Procedure for the Ni-catalyzed Decarboxylative Ethynylation 54

General Procedure C6: Iron-catalyzed Kumada Alkynylation 54

Preparation of Substituted Alkynylmagnesium Bromide Solution 55

Procedure for the Fe-catalyzed Decarboxylative Alkynylation 55

General Procedure C7: Nickel-catalyzed Boronic Ester Suzuki Cross-Coupling............ 56

Preparation of Nickel/ligand Solution 56

Preparation of B_2pin_2/MeLi-Complex Solution.. 56

General Procedure C8: Nickel-Catalyzed Giese Conjugate Addition............................... 57

Troubleshooting: Frequently Asked Questions 58

C–H Activation 58

Decarboxylative Cross-Couplings 59

Experimental Procedures and Characterization Data for Linear and Strained-Ring Systems (Figure 2).. 61

Compound B1 61

Compound B2 62

Compound B3 63

Compound B4 64

Compound B5 65

Compound B6 66

Compound B7 67

Compound 14 68

Compound 15 69

Compound 16 70

Compound 17 71

Compound 18 72

Compound 19 73

Compound 20 74

Compound 21 75

Compound 22 76

Compound 23 77

Compound 24 78

Experimental Procedures and Characterization Data for Azetidine Scaffolds (Figure 3–a)79

Compound B8: 79

Compound 26 81

Compound 27 83

Compound 28 85

Compound 29 87

Compound 30 89

Compound 31 91

Compound 32 93

Experimental Procedures and Characterization Data for Pyrrolidine Scaffolds (Figure 3–b) 95

Compound B10 95

Compound 33 97

Compound 34 99

Compound 35 101

Compound 36 103

Compound 37 105

Compound 38 107

Compound 39 109

Compound 40 111

Compound B11 113

Compound 41 115

Compound 42 117

Compound 43 119

Compound 44 121

Compound B15 123

Compound 45 124

Experimental Procedures and Characterization Data for Tetrahydrofuran Scaffolds (Figure 3 – c) 125

Compound SI-39: 125

Compound SI-40: 126

Compound SI-41: 127

Compound A9: 128

Compound B12: 129

Compound 46 131

Compound 47 133

Compound 48 135

Compound 49 137

Compound 50 139

Compound 51 141

Compound 52 143

Experimental Procedures and Characterization Data for Piperidine (*C-2*)- Scaffolds (Figure 3–d) 145

Compound B13 145

Compound 53 147

Compound 54 149

Compound 55 151

Compound 56 153

Compound 57 155

Compound 58 157

Compound 59 159

Experimental Procedures and Characterization Data for Piperidine (*C-3*)- Scaffolds (Figure 3–e) 161

Compound B14 161

Compound 60 163

Compound 62 167

Compound 63 169

Compound 64 171

Compound 65 173

Experimental Procedures and Characterization Data for Morpholine Scaffolds (Figure 3–e) 175

Compound SI-3 175

Compound SI-4 177

Compound B16 179

Compound 66 181

Compound 67 183

Compound 68 185

Experimental Procedures and Characterization Data for hit-to-lead candidates and late-stage intermediates (Figure 4) 187

Compound SI-42 187

Compound 69 189

Compound SI-43 190

Compound A13 191

Compound 5 193

Unsuccessful or Challenging Substrates 196

Morpholine Scaffold: C-H sp^{3}Arylation 196

Table S2: 8-*Aminoquinoline as directing group*: Screening of Bases and solvents using $Pd(OAc)_2$ as catalyst. 196

Table S3: *Boc as protecting group*: Screening of Bases and solvents using $Pd(OAc)_2$ as catalyst. 197

Table S4*: PIP as directing group*: Screening of Bases and solvents using $Pd(OAc)_2$ as catalyst. 198

Morpholine Scaffold: C-H sp^3 Methylation and Alkynylation 199

Table S5*:* Screening of conditions for C-H sp^3 Methylation 199

Table S6*:* Screening of conditions for C-H sp^3 Alkynylation 199

Piperazine Scaffold: C-H sp^{3}alkoxylation and arylation 200

Table S7*:* Screening of conditions for C-H sp^3 Alkoxylation 200

Table S8: *8-Aminoquinoline as directing group*: Screening of conditions for arylation. ... 201

Table S9: *2-thiomethylaniline as directing group:* Screening of conditions for arylation.. 201

Experimental Procedures and Characterization Data for BRD8468 (74) (Figure 5-a)...... 202

Synthetic route to compound BRD8468 (74) 202

Compound BRD8468 203

Experimental Procedures and Characterization Data for the synthesis of BRD8468 analogs (Figure 5-b) 204

Compound SI- 44 204

Compound 74 205

Compound SI-45 206

Compound A15 208

Compound A16 209

Compound B9 210

X-Ray Crystallographic Data Compound B9 211

Table S10. Crystal data and structure refinement for Baran705. 212

Table S11. Atomic coordinates (x 10^4) and equivalent isotropic displacement parameters (Å^{2}x 10^3) 213

Compound SI-46 and 73 215

N-(((2*S*,3*S*)-3-(4'-fluoro-[1,1'-biphenyl]-4-yl)-1-(3,3,3-trifluoropropyl)azetidin-2-yl)methyl)cyclopentanamine (73) 216

Compound 75 217

Compound 76 221

Compound 77 224

Compound SI-50 227

Compound 78 228

Compound 80 230

Compound 79 231

Compound 81 232

Compound 82 233

Compound 83 234

Biological assays: Comments, Procedures and Results (Figure 5-c) 235

Importance of the trans-relationship between the biaryl substituent and the vicinal group 235

Example of tolerated diversity at azetidine nitrogen 235

Materials 236

Methods 236

Table S12. *In vitro* antimalarial potency and cytotoxicity of synthesized di- and tri-substituted azetidines (s.d. = standard deviation, n = number of biological replicates). 240

Table S13. *In vitro* antimalarial potency and cytotoxicity of selected azetidines and reference compounds. Compound 80 remains equipotent against *Pf* drug-resistant strains (s.d. = standard deviation, n = number of biological replicates) 241

NMR Spectra 242

References 488

General Experimental

Tetrahydrofuran (THF), *N*,*N*-dimethylformamide (DMF), toluene, acetonitrile (CH_3CN), and dichloromethane (CH_2Cl_2) were obtained by passing the previously degassed solvents through an activated alumina column. Reagents were purchased at the highest commercial quality and used without further purification, unless otherwise stated. $NiCl_2$•glyme and $Ni(acac)_2$•xH_2O were purchased from Strem. Bipyridine ligands (2,2'-bipyridine, 4,4'-di-*tert*-butylbipyridine and 4,4'-di-OMe-bipyridine) were purchased from Sigma-Aldrich. Bathophenanthroline was purchased from Combi-Blocks. Yields refer to chromatographically and spectroscopically (^{1}H NMR) homogeneous material, unless otherwise stated. Reactions were monitored by LC/MS, and thin layer chromatography (TLC). TLC was performed using 0.25 mm E. Merck Silica plates (60F-254), using short-wave UV light as the visualizing agent, and phosphomolybdic acid and $Ce(SO_4)_2$, acidic ethanolic anisaldehyde, or $KMnO_4$ as developing agents upon heating. NMR spectra were recorded on Bruker DRX-600, DRX-500, and AMX-400 instruments and are calibrated using residual undeuterated solvent ($CHCl_3$ at 7.26 ppm ^{1}H NMR, 77.16 ppm ^{13}C NMR; acetone at 2.09 ppm ^{1}H NMR, 205.87, 30.60 ppm ^{13}C NMR; CH_3OH at 3.34 ppm ^{1}H NMR, 49.86 ppm ^{13}C NMR; C_6H_6 at 7.15 ppm ^{1}H NMR, 128.62 ppm ^{13}C NMR). The following abbreviations were used to explain multiplicities: s = singlet, d = doublet, t = triplet, q = quartet, m = multiplet, br = broad. Column chromatography was performed using E. Merck Silica (60, particle size 0.043–0.063 mm), and pTLC was performed on Merck Silica plates (60F-254). High-resolution mass spectra (HRMS) were recorded on an Agilent LC/MSD TOF mass spectrometer by electrospray ionization time of flight reflection experiments. Melting points were recorded on a Fisher-Johns 12-144 melting point apparatus and are uncorrected. The enantiomeric excesses were determined with Waters UPC^2 SFC equipped with a photodiode array detector or an Agilent Technologies 1220 Infinity II LC HPLC. Optical rotations were recorded on a Rudolph Research Analytical Autopol III Automatic Polarimeter.

General procedures

General Procedure A1: Asymmetric sp^3 C–H arylation sequence For Linear and Strained-Ring Systems

A1.1: Installation of Directing Group

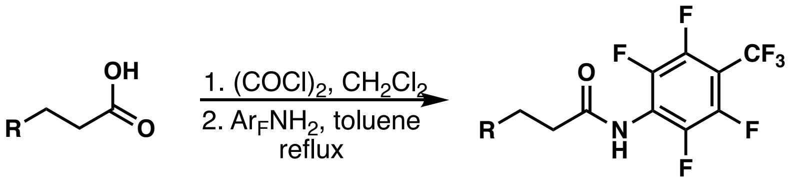

The amides were prepared according to a previously reported procedure.[1]

The carboxylic acid (1 equiv.) was suspended in anhydrous CH_2Cl_2 (0.36 M) under argon atmosphere. Oxalyl chloride (1.2 equiv.) and *N*,*N*-dimethylformamide (0.06 equiv.) were added carefully to the mixture (gas evolution observed). Upon completion, monitored by TLC, the solution was concentrated *in vacuo* to afford the acid chloride, which was used immediately in the next reaction without characterization. Then, the acid chloride was added to a vigorously stirred solution of 2,3,5,6-tetrafluoro-4-(trifluoromethyl)aniline (Ar_FNH_2) (1.1 equiv.) in toluene (1 M). The reaction mixture was stirred for 12 hours under reflux, then it was cooled to room temperature and concentrated under vacuum, followed recrystallization in EtOAc:Hexanes to give the desired product.

A1.2: C–H Activation

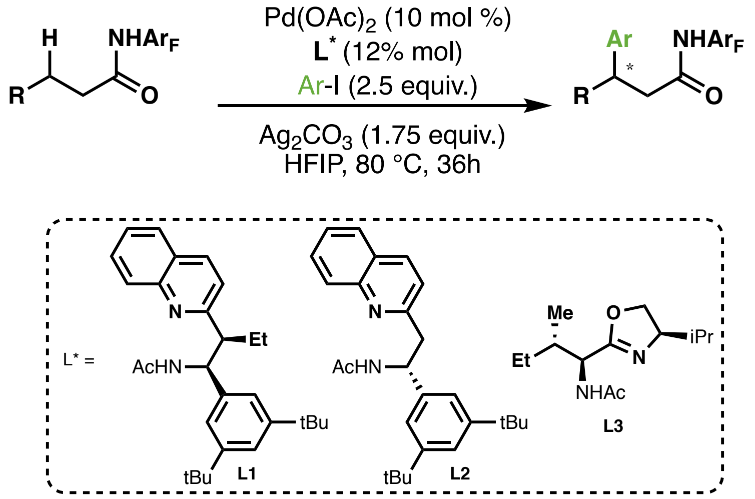

Arylations were carried out according to a previously reported procedure.[2]

The amide (1 equiv.), $Pd(OAc)_2$ (10 mol%), ligand **L***(12 mol%), and Ag_2CO_3 (2 equiv.) were weighed in air and placed in a sealed tube (100 mL) with a magnetic stir bar. To the reaction mixture, aryl iodide (2 equiv.) and HFIP (0.1M) were added. The reaction mixture was first stirred at room temperature for 10 minutes and then heated to 80 °C for 36 hours under vigorous stirring. Upon completion, the reaction mixture was cooled to room temperature and filtered through Celite® using CH_2Cl_2. The solvents were removed *in vacuo* and the resulting mixture was purified by a preparative TLC using Hexanes:EtOAc (5:1) or Toluene:EtOAc (30:1) as the eluent. In the ^{13}C NMR analysis, peaks that correspond to those of the polyfluoroarylamide auxiliary appeared as nearly invisible, complex sets of multiplets; they are omitted in the following spectroscopic analysis.

A1.3: Removal Directing Group

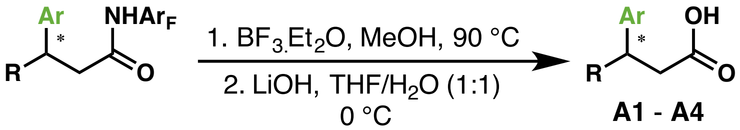

A sealable pressure flask was charged with MeOH (0.025 M) and amide. Et_2O•BF_3 (35 equiv.) was added and the reaction vessel sealed. The reaction mixture was heated to 100 °C for 12 hours under vigorous stirring. Upon completion, the reaction mixture was cooled to room temperature. The solvent was removed *in vacuo*, H_2O and EtOAc were added, organic layers were removed, and the aqueous layer was then extracted with EtOAc (3x). The combined organic layers were washed with brine, dried over $MgSO_4$, filtered and concentrated. Purification by column chromatography (Hexanes:EtOAc = 2:1 to 1:1) afforded the corresponding ester.

The ester was dissolved in THF (10 mL). The solution was cooled to 0 °C, and a cold solution of LiOH•H_2O (2 equiv.) in H_2O (10 mL) was added. The reaction was maintained at 0 °C for 1 hour. The reaction was acidified with 2 M HCl and extracted with EtOAc. The combined organic layers were dried over $MgSO_4$ and concentrated. The residue was purified by column chromatography (Hexanes:EtOAc:AcOH = 10:10:1).

The following carboxylic acids were prepared according to **General Procedures A1.1-A1.3**:

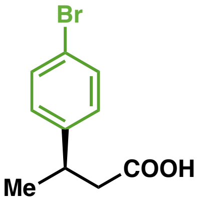

A1

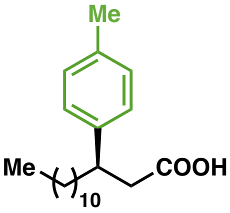

A2

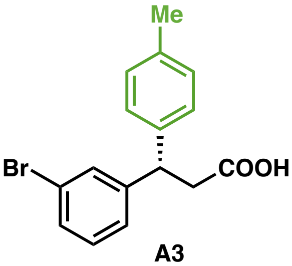

A3

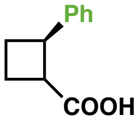

A4

A1.4: C–H Activation - free carboxylic acid

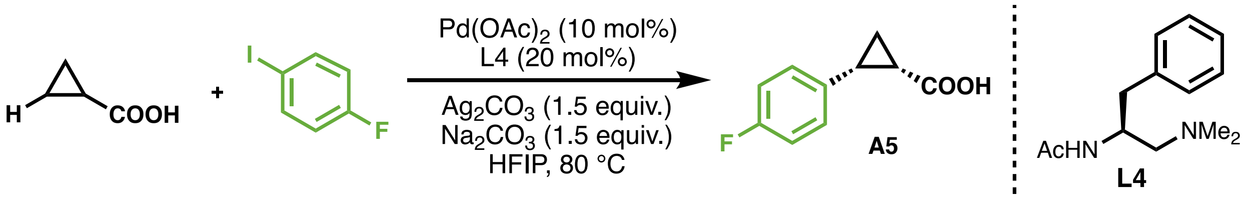

A5 was prepared according to a previously reported procedure.[3]

A 2-dram vial equipped with a magnetic stir bar was charged with $Pd(OAc)_2$ (4.4 mg, 10 mol%) and **L4** (8.8 mg, 20 mol%) in HFIP (0.25 mL). The appropriate cyclopropanecarboxylic acid substrate (0.20 mmol), Ag_2CO_3 (82.7 mg, 0.30 mmol, 1.5 equiv.), Na_2CO_3 (31.8 mg, 0.30 mmol, 1.5 equiv.) and aryl iodide (0.40 mmol, 2 equiv.) were then added. Subsequently, the vial was capped and closed tightly. The reaction mixture was then stirred at the rate of 200 rpm at 80 °C for 16 hours. After being allowed to cool to room temperature, the mixture was diluted with EtOAc, and 0.1 mL of acetic acid was then added. The mixture was passed through a pad of Celite® with EtOAc as the eluent to remove any insoluble precipitates. The resulting solution was concentrated, and the residual mixture was dissolved with a minimal amount of acetone and loaded onto a preparative TLC plate. The pure product was then isolated using preparative TLC with EtOAc:Hexanes (1:4 to 1:1) as the eluent and 1% v/v of acetic acid as the additive.

A1.5 Decarboxylative Borylation using copper catalyst

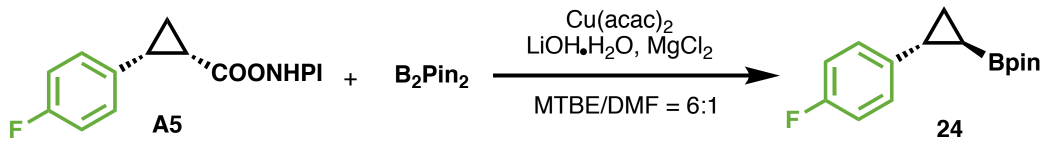

To a 15 mL culture tube equipped with a stir bar were added redox-active ester **A5** (1.0 equiv.), B_2pin_2 (3.0 equiv.), $LiOH•H_2O$ (15 equiv.), $Cu(acac)_2$ (30 mol%) and $MgCl_2$ (1.5 equiv.). The tube was evacuated and backfilled with argon for 3 times. A degassed MTBE:DMF solution (6:1, 0.14 M) was added and the resulting mixture was stirred under 1000 rpm at room temperature until dark brown color was observed (typical reaction time: < 15 min). The reaction mixture was diluted with EtOAc and saturated NH_4Cl, and the resulting mixture was shaken vigorously until getting a clear biphasic solution. The organic phase was collected, dried over anhydrous Na_2SO_4, evaporated and purified by silica gel chromatography to afford the desired product **24**.

General Procedure A2: sp^3 C–H Arylation sequence for Azetidine Scaffolds

A2.1: Installation of Directing Group

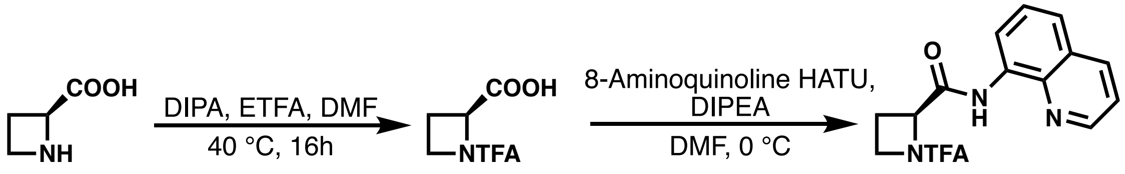

9 25 SI-1

(*S*)-*N*-(quinolin-8-yl)-1-(2,2,2-trifluoroacetyl)azetidine-2-carboxamide was prepared according to the previously reported procedure.[4]

A round-bottom flask equipped with stir bar was charged with (*S*)-azetidine-2-carboxylic acid **9** (1.0 equiv.) and DMF (0.4 M), followed by *N*,*N*-diisopropylethylamine (DIPEA, 2.0 equiv.) and ethyl trifluoroacetate (5.0 equiv.). The reaction mixture was heated to 40 °C for 16 hours, then excess reagents were removed *in vacuo*. The crude mixture was dissolved in DMF (0.25 M) and cooled to 0 °C before the addition of HATU (1.0 equiv.), 8-aminoquinoline (1.0 equiv.), DIPEA (0.95 equiv.) in sequence. The reaction mixture was maintained at 0 °C until completion. The reaction mixture was then diluted with EtOAc and washed with water (2x) and brine (1x). The resulting organic layer was dried over Na_2SO_4, filtered, and concentrated *in vacuo*. Purification by flash column chromatography (EtOAc:Hexanes) provided **SI-1**.

A2.2: C–H Activation

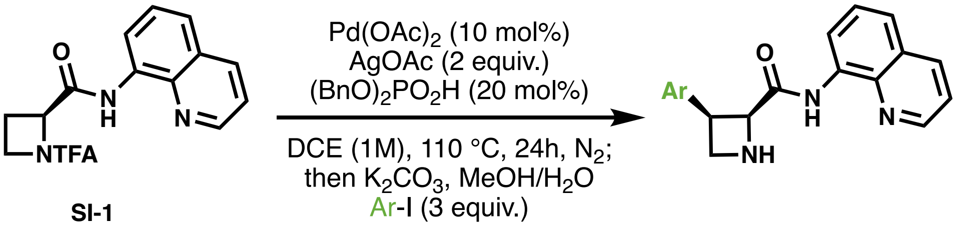

C–H arylation was preformed according to a previously reported procedure.[4]

A sealed tube was charged with **SI-1** (1.0 equiv.), dibenzyl phosphate (20 mol%), AgOAc (2.0 equiv.), aryl iodide (3.0 equiv.), and $Pd(OAc)_2$ (10 mol%). The reaction vessel was evacuated and refilled with inert atmosphere (N_2, 3x). DCE (1.0 M) was then added and placed in an oil bath pre-heated to 110 ºC for 24 hours. Then, the reaction mixture was cooled to room temperature and the crude material was transferred to a round-bottom-flask using a 9:1 MeOH:H_2O solution (8 mL of solution per 1 mmol reactant), followed by the addition of K_2CO_3 (3.0 equiv.). The mixture was allowed to stir at ambient temperature for up to 16 hours before solvent removal *in vacuo*. The resulting crude material was dissolved in CH_2Cl_2, filtered over Celite® and concentrated *in vacuo.* The resulting crude material was dissolved in 3:7 *i*-PrOH:$CHCl_3$, washed with water (1x) and with brine (1x). The resulting organic layer was dried over Na_2SO_4, filtered, concentrated *in vacuo* before purification by flash column chromatography under the specified conditions.

The following arylated amides were prepared according to the **General Procedure A2.2**:

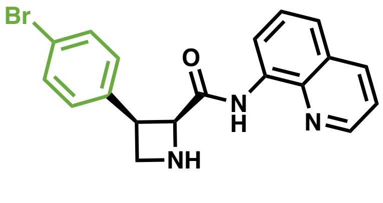

SI-2

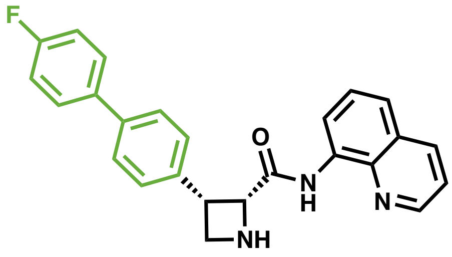

74 using *ent*-**SI-1**

A2.3: Removal Directing Group

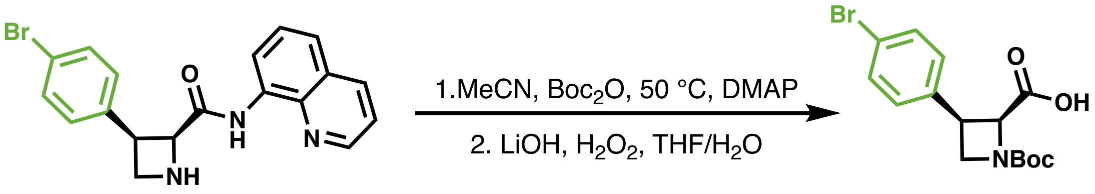

SI-2

A6

The carboxyl acid **A6** was prepared according to a previously reported procedure.[4]

The arylated product **SI-2** (1.0 equiv.) was added to a round bottom flask and dissolved in MeCN (0.2 M). Boc_2O (3 equiv.) was added, and the reaction mixture was heated to 50 ºC and stirred for 15 minutes, then DMAP (0.1 equiv.) was added, and the mixture was stirred for another 2 hours at 50 ºC. The solvent was removed *in vacuo* and the residue was dissolved in THF:H_2O (2:1, 0.1 M). After cooling the biphasic mixture to 0 ºC, 30% aq. H_2O_2 (10 equiv.) was added followed by LiOH (6 equiv.). The reaction mixture was allowed to reach room temperature, stirred for 1 hour and then heated to 50 ºC for 20 hours. The reaction mixture was separated, and the aqueous layer was washed with Et_2O (3x). The resulting aqueous solution was acidified with 1 M HCl, exhaustively extracted with 3:7 i-PrOH:$CHCl_3$ (5x) and dried. Concentration *in vacuo* gave the acid **A6** as a solid which was used directly in the following step without further purification.

General Procedure A3: sp[3] C–H Arylation Sequence for Pyrrolidine, Tetrahydrofuran and Piperidine Scaffolds

A3.1: Installation of Directing Group

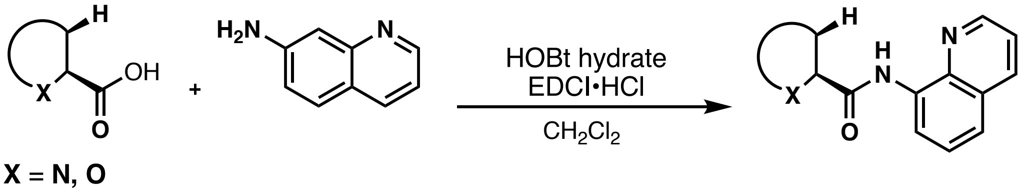

The amides were prepared according to the previously reported procedure.[5]

A round-bottom flask equipped with stir bar was charged with HOBt (1.2 equiv.), carboxylic acid (1 equiv.) and CH_2Cl_2 (0.2 M), after 5 minutes stirring at room temperature, EDCI·HCl (1.2 equiv.) was added and the solution was stirred for further 72 hours. The reaction mixture was diluted with CH_2Cl_2, aq. sat. $NaHCO_3$ was added and the aqueous layer was extracted with CH_2Cl_2 (3x), dried over Na_2SO_4 and purified by column chromatography.

A3.2: C–H Activation

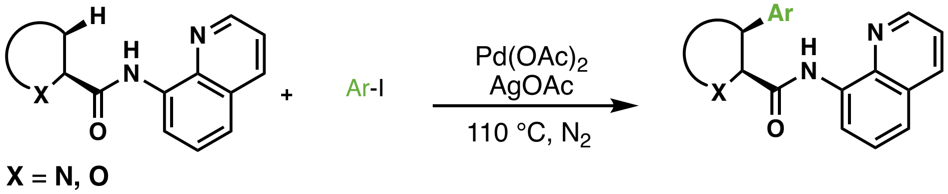

The arylated compounds were prepared according a previously reported procedure.[5]

A sealed tube was charged with amide (1.0 equiv.), AgOAc (2.0 equiv.), aryl iodide (3.0 equiv.) and $Pd(OAc)_2$ (10 mol%). The tube was flushed with argon and sealed (for tetrahydrofuran scaffolds)*, then placed in an oil bath preheated to 110 °C and stirred for 38 hours. The reaction mixture was then allowed to cool down to room temperature and EtOAc was added. The resulting solution was filtered through a pad of Celite®, eluting with further EtOAc (2x). The solvent was removed *in vacuo*, and the crude material was purified by flash column chromatography.

**The C–H arylation for pyrroline and piperidine were carried out under air.*

A3.3: Removal Directing Group

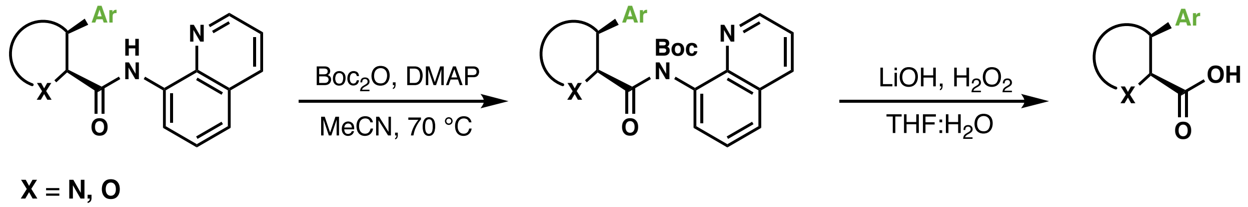

The carboxyl acids were prepared according to the previously reported procedure.[5]

A culture tube equipped with a stir bar was charged with the arylated compound (1.0 equiv.), DMAP (3.0 equiv.) and MeCN (1 M), followed by dropwise addition of $(Boc)_2O$ (20.0 equiv.) at room temperature. Then, the tube was placed in an oil bath preheated to 110 ºC for 1 hour. The mixture was then cooled to room temperature and another equivalent of $(Boc)_2O$ (10.0 equiv.) and DMAP (3.0 equiv.) was added and the reaction mixture stirred at 70 ºC overnight. The culture tube was then allowed to cool to room temperature and the solvent concentrated *in vacuo*. The residue was purified by Silica gel column chromatography to afford the Boc-protected intermediate.

**The Boc-protection of pyrroline was carried out using (20 equiv) of $(Boc)_2O$ and for piperidine and tetrahydrofuran (30 equiv) of $(Boc)_2O$.*

To a solution of Boc-amide (1.0 equiv.) in $THF:H_2O$ (0.1 M, 3:1) were added $LiOH{\bullet}H_2O$ (2.0 equiv.) and 30% H_2O_2 (5.0 equiv) at 0 ºC. The reaction mixture was stirred for 20 minutes at 0 ºC, then was allowed to warm up to room temperature and stirred for 18 hours. After completion, the mixture was extracted with Et_2O to remove the organic impurities, the aqueous layer was acidified with 1M aq. HCl to pH=3 and extracted with EtOAc (3x). The combined organic layers were washed with brine, dried over Na_2SO_4 and concentrated *in vacuo* to afford the carboxylic acid, that was used in subsequent steps without further purification.

The following carboxylic acids were prepared according to the **General Procedure A3**:

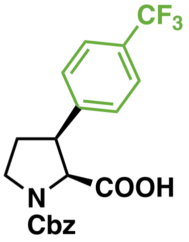

A7

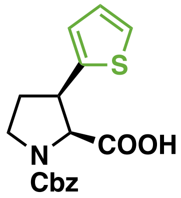

A8

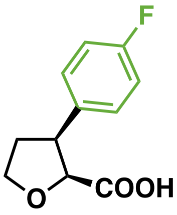

A9

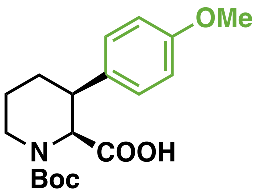

A10

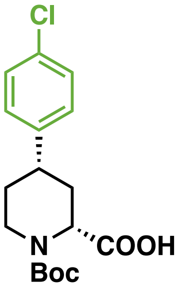

A11

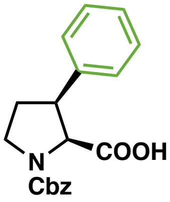

A12

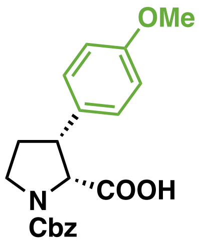

A13

Graphical Supporting Information for Pyrrolidine, Piperidine and Tetrahydrofuran Scaffolds

General Procedure A3.2

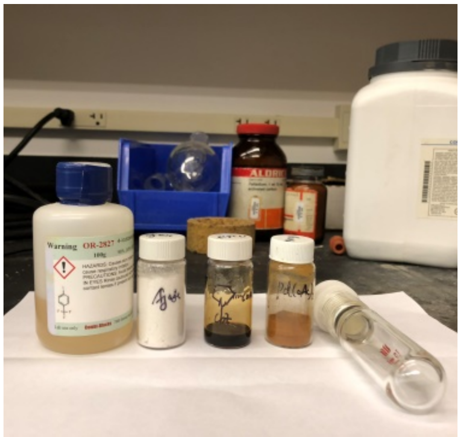
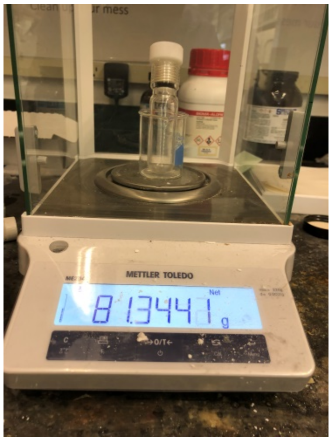

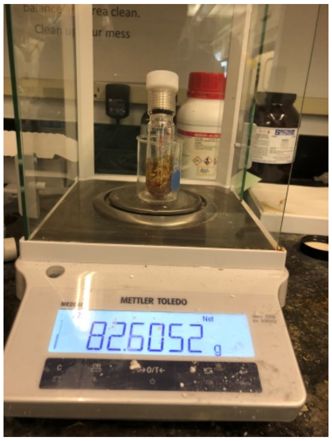

Left: Reagents. **Center:** Taring of the sealed tube. **Right**: Addition of amide.

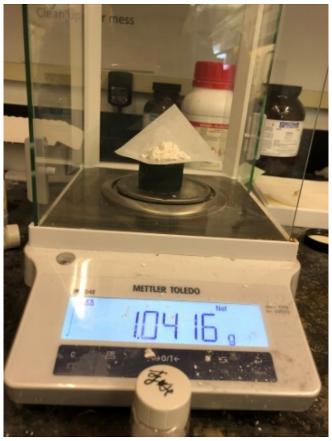

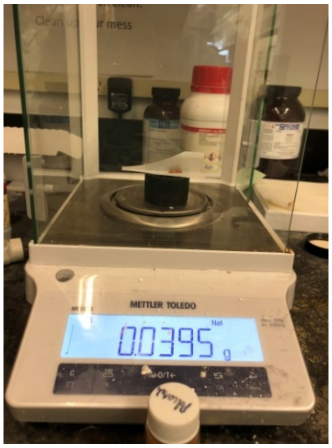

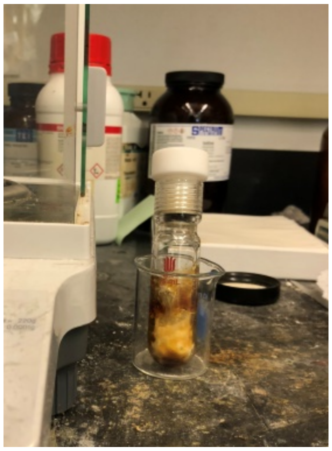

Left: AgOAc. **Center:** $Pd(OAc)_2$. **Right:** Reaction tube containing all solid reagents.

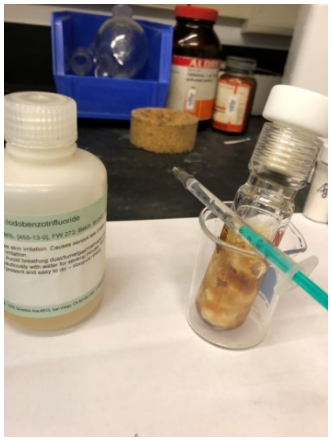
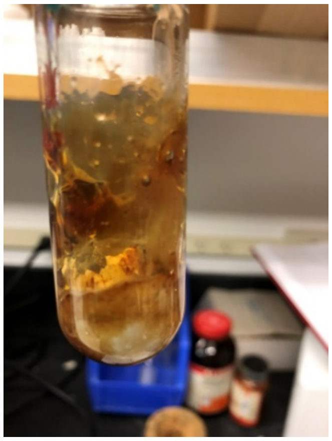
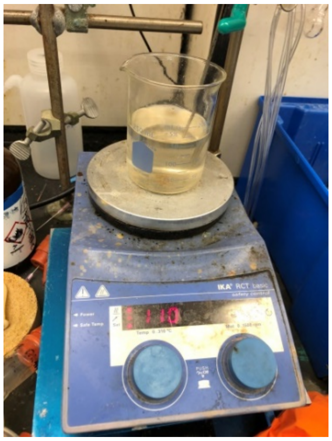

Left: ArI. **Center:** After addition of ArI. **Right:** Oil bath preheated to 110 ºC.

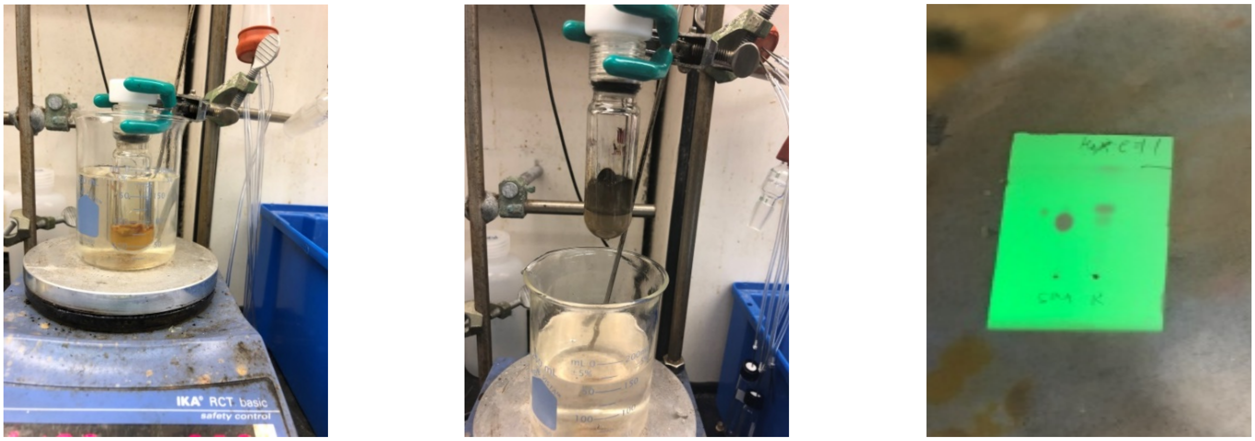

Left: Reaction after 10 mins. **Center:** Reaction after 20 hours. **Right:** TLC for starting material (left) and crude reaction mixture (right, top spot is the product).

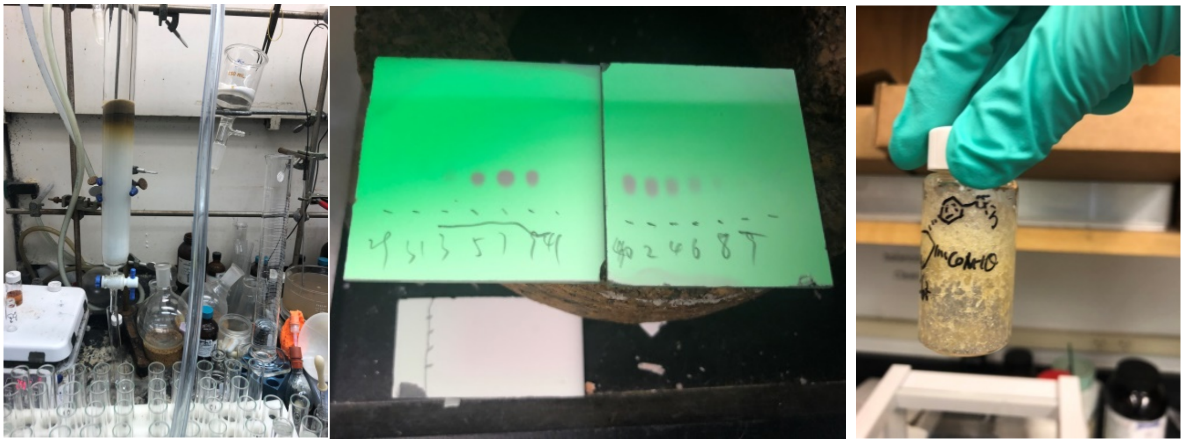

Left: Column chromatography. **Center:** TLC of column fractions. **Right:** Final product.

General Procedure A3.3: ***Boc-protection***

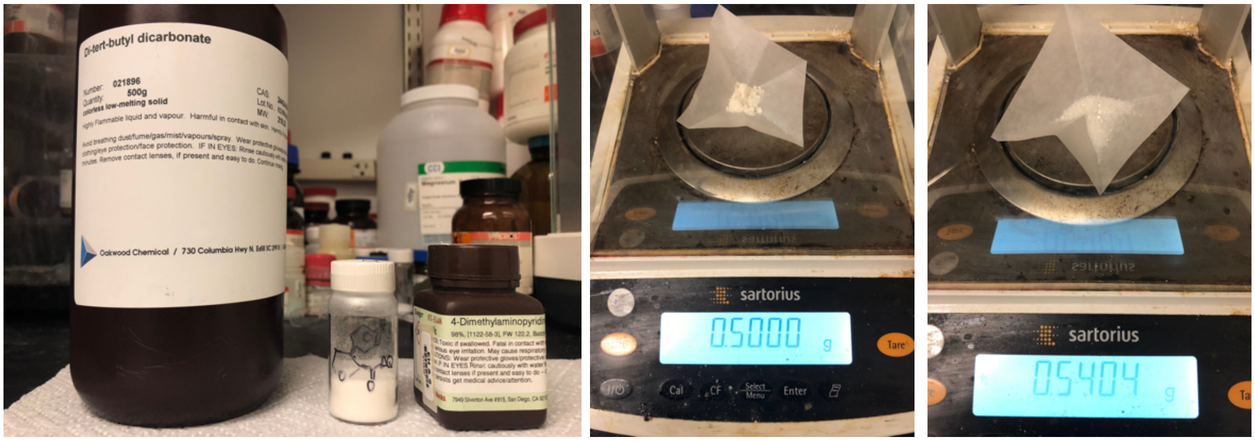

Left: Reagents. **Center:** Arylated Product (**A3.2**). **Right:** DMAP.

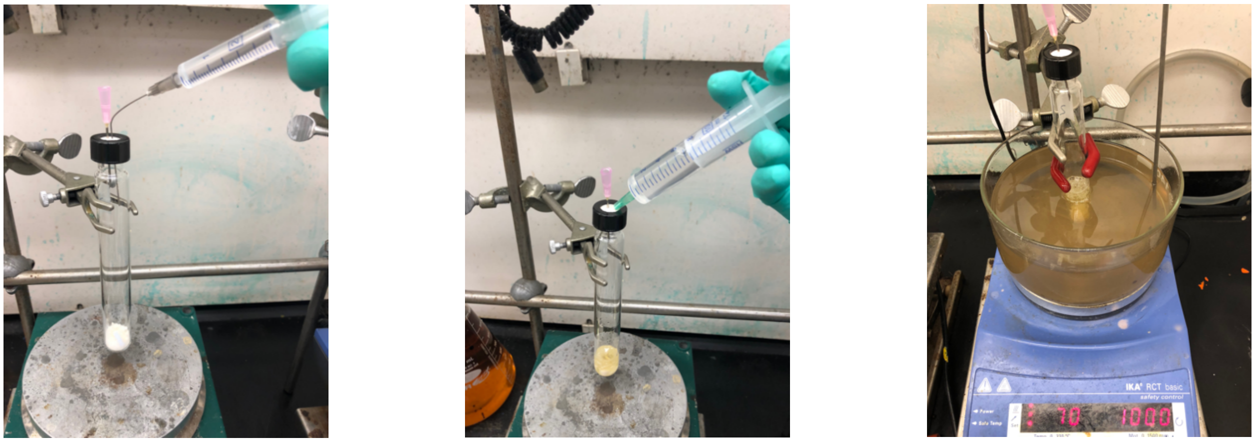

Left: Addition of MeCN to the culture tube containing solids. **Center:** Dropwise addition of $(Boc)_2O$. **Right:** Culture tube placed in oil bath at 70 ºC.

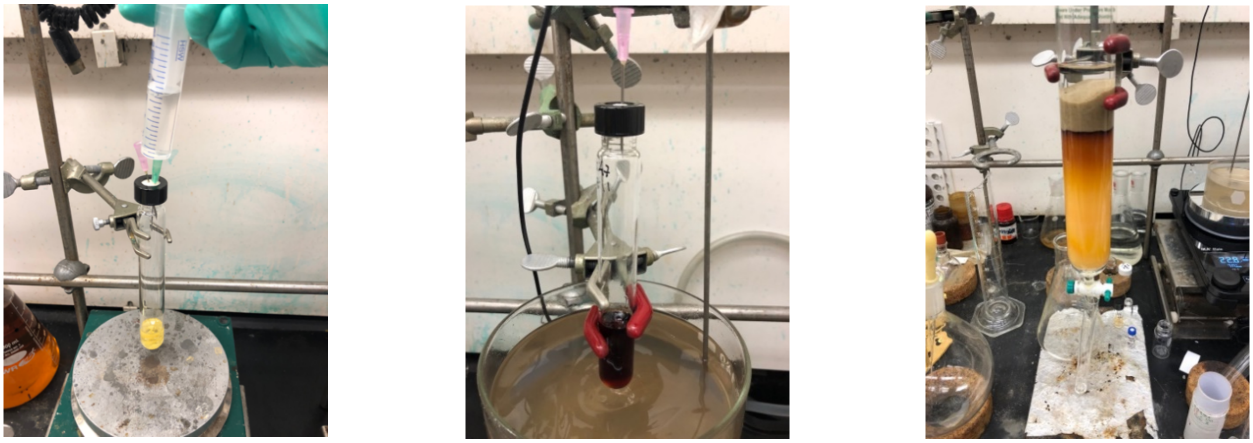

Left: Dropwise addition of $(Boc)_2O$ after 1hour**. Center:** After stirring overnight. **Right:** Purification by column chromatography.

General Procedure A3.3: ***Hydrolysis of the Amide Group***

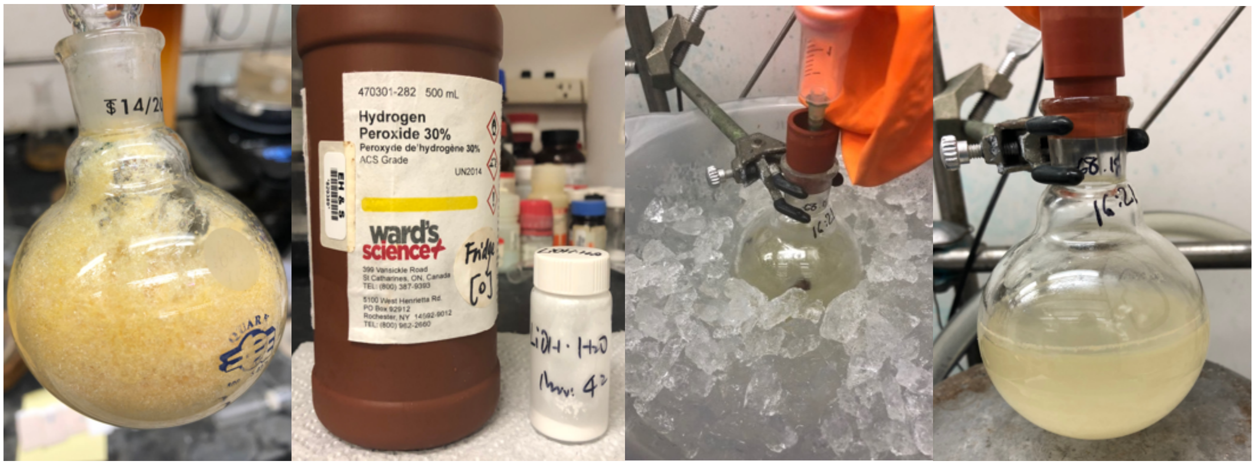

Left: Reagents. **Center:** After addition of H_2O_2 at 0 ºC. **Right:** Reaction mixture after 18 hours.

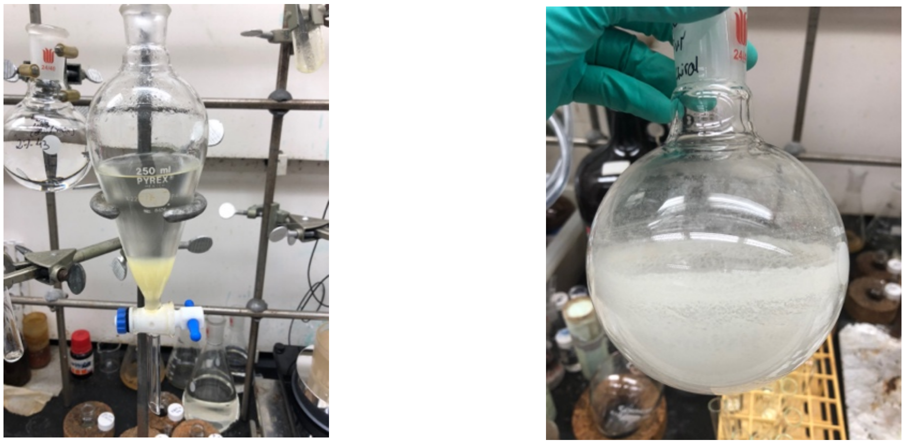

Left: Extraction of aqueous phase after acidification with HCl 1 M. **Right:** Crude product.

General Procedure A4: sp[3] C–H Methoxylation Sequence for Morpholine Scaffold

A4.1: Installation of Directing Group

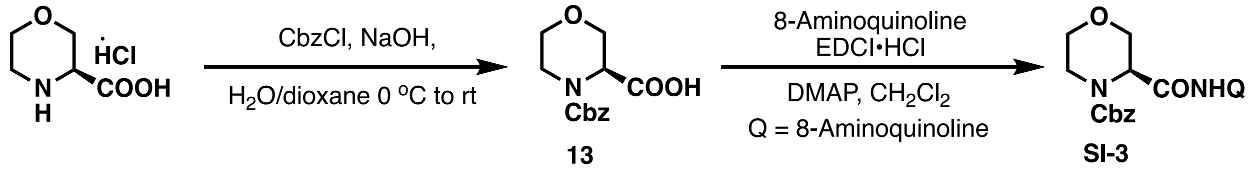

(*S*)-Morpholine-3-carboxylic acid hydrochloride (1 equiv.) was dissolved in H_2O:Dioxane (0.16 M, 1:2) then the pH of the solution was adjusted to 11 with 50 wt % aq. NaOH at 0 ºC. A solution of CbzCl (3.33 M, 2 equiv.) was then added dropwise while maintain the pH at 11 with 50 wt % aq. NaOH. Then the reaction mixture was stirred at room temperature overnight. After completion, organic impurities were removed by extracting with Et_2O. The aqueous layer was acidified to pH = 1 with 3 M HCl, then extracted with EtOAc (3x), dried over Na_2SO_4 and concentrated *in vacuo* to afford the desired product **13**, which was used without further purification.

To a solution of the acid intermediate (1 equiv.) and 8-aminoquinoline (1.1 equiv.) in anhydrous CH_2Cl_2 (0.1 M) were added EDCI•HCl (1.1 equiv.) and DMAP (0.2 equiv.). The resulting mixture was stirred at room temperature for 36 hours, then quenched with 1 M HCl and diluted with EtOAc. The organic layer was separated, and the aqueous layer was further extracted with EtOAc (3x). The combined organic layers were washed with brine, dried over Na_2SO_4 and concentrated *in vacuo*. Flash column chromatography (Silica gel, Hexanes:EtOAc) afforded **SI-3**.

A4.2: C–H Activation

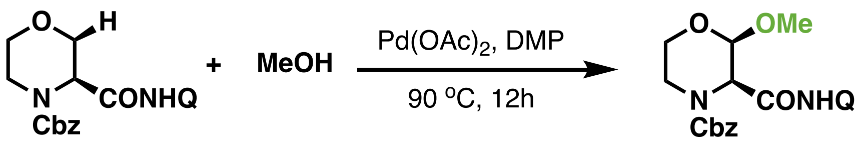

SI-3 **SI-4**

A sealed tube was charged with **SI-3** (1.0 equiv.), Dess–Martin periodinane (3 equiv.), $Pd(OAc)_2$ (20 mol%) and MeOH (0.33 M). The reaction tube was sealed, then placed in an oil bath preheated to 90 ºC for 20 hours. Then the reaction mixture was allowed to cool to room temperature and EtOAc was added. The resulting solution was filtered through a pad of Celite®, and further washed with EtOAc (2x). The solvent was removed *in vacuo*, and the crude material was purified by flash column chromatography to afford **SI-4**.

A4.3: Removal Directing Group and synthesis of RAE

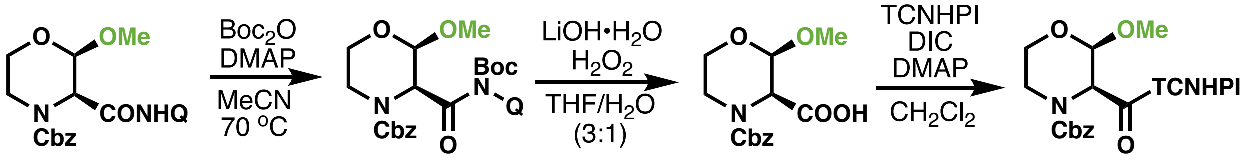

SI-4 SI-5 A14 B16

To a solution of compound **SI-4** (1.0 equiv.) and DMAP (3 equiv.) in MeCN (0.08 M) was slowly added $(Boc)_2O$ (20 equiv.) at room temperature. The reaction mixture was stirred at 70 ºC for 6 hours. After cooling to room temperature, the reaction mixture was concentrated *in vacuo*. The residue was purified by flash column chromatography (silica gel, 2:1 Hexanes:EtOAc) to yield compound **SI-5** as a brown oil.

To a solution of compound **SI-5** (1.0 equiv.) in THF:H_2O (0.08 M, 3:1) were added LiOH•H_2O (2 equiv.) and 30 % H_2O_2 (5 equiv.) at 0 ºC. The reaction mixture was stirred for 20 minutes at 0 ºC, then allowed to warm up to room temperature and stirred for 18 hours. After completion, the reaction was extracted with Et_2O to remove the organic impurities and then the aqueous layer was acidified with 1 M HCl to PH=6 and extracted with EtOAc (3x). The combined organic layers were washed with brine (3x), dried over Na_2SO_4 and concentrated *in vacuo* to afford **A14** as a white solid, which was used without further purification.

A round-bottom flask was charged with **A14** (1.0 equiv.), N-hydroxy-tetrachlorophthalimide (1.1 equiv.), and DMAP (0.2 equiv.). CH_2Cl_2 was added (0.1 M), and the mixture was stirred vigorously. DIC (1.1 equiv.) was then added dropwise via syringe, and the mixture was allowed to stir until the acid was consumed (determined by TLC). The mixture was filtered (over Celite®, silica gel, or through a fritted funnel) and rinsed with additional CH_2Cl_2. Flash column chromatography (silica gel, 30:1 CH_2Cl_2: Et_2O) afforded the compound **B16** as a white solid.

Graphical Supporting Information for Morpholine Scaffold (General Procedure A4)

C–H Activation

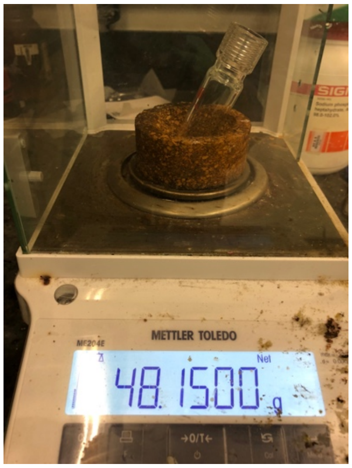

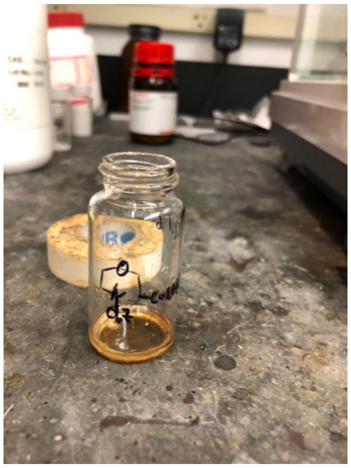
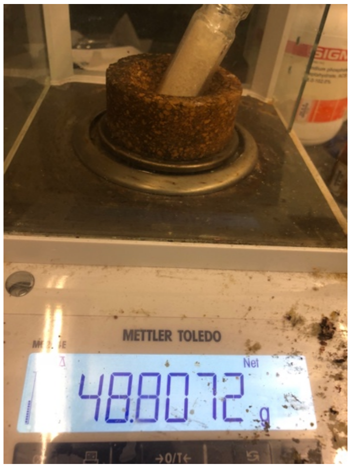

Left: Tare of the sealed tube. **Center:** Amide starting material. **Right**: Addition of amide.

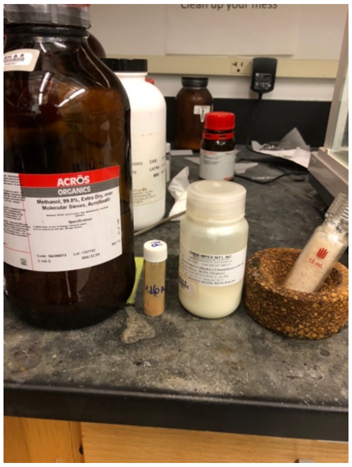

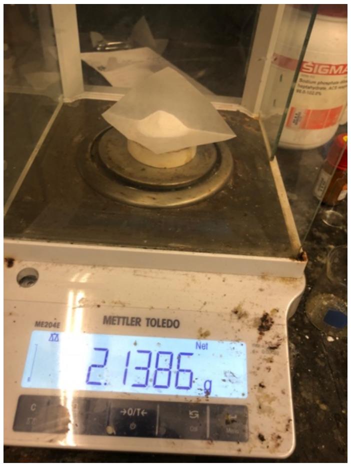

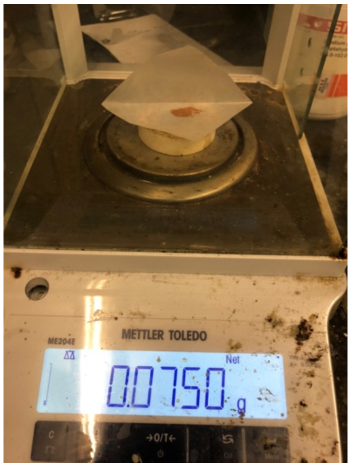

Left: Reagents. **Center:** DMP. **Right:** $Pd(OAc)_2$.

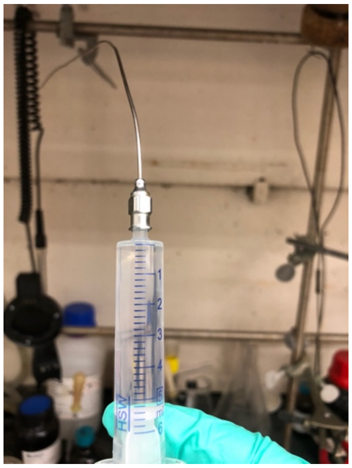
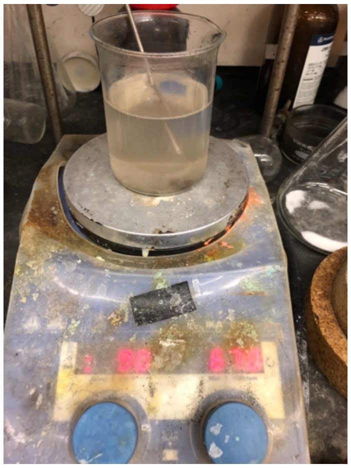
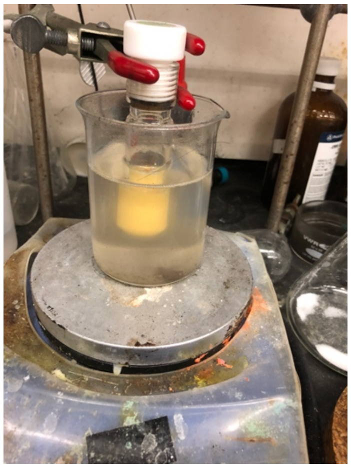

Left: MeOH. **Center:** Oil bath preheated to 110 ºC. **Right:** Reaction after 5 minutes.

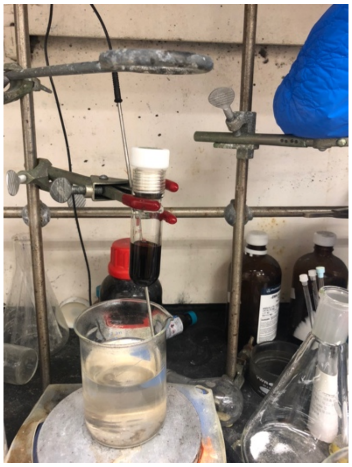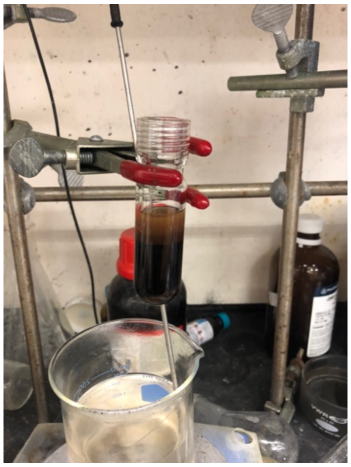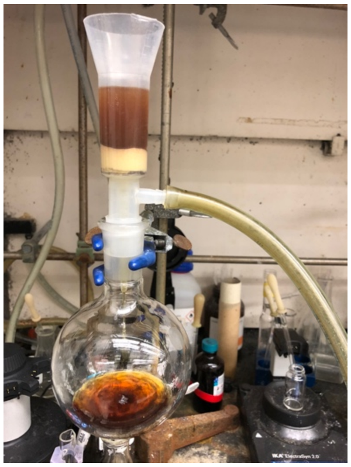

Left: Reaction after 12 hours. **Center:** Dilute the reaction with EtOAc (5 mL). **Right:** Filter through a pad of Celite®.

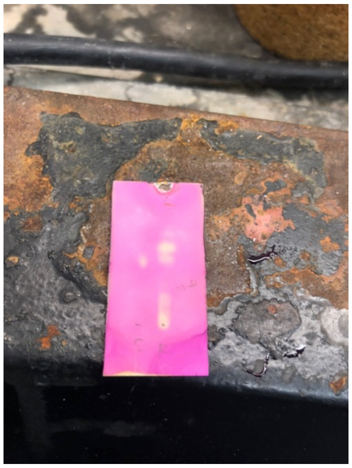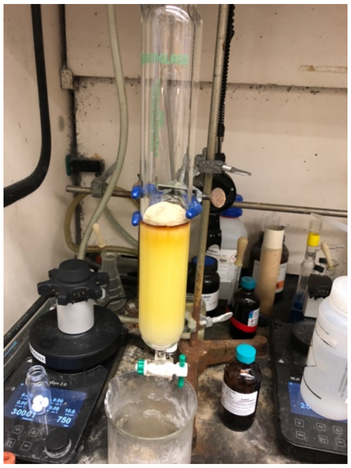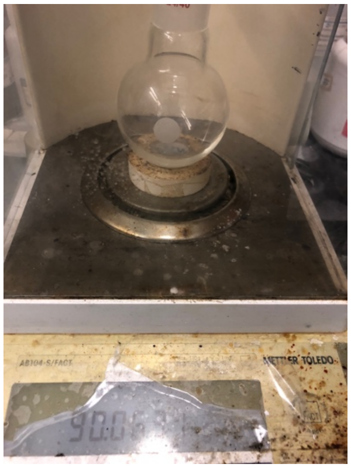

Left: TLC for starting material (left) and crude reaction mixture (right, top spot is the product). **Center:** Column chromatography. **Right:** Weight of the empty flask for product.

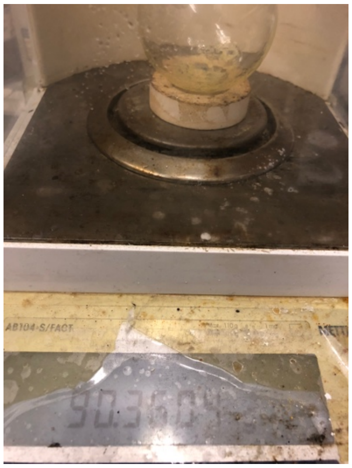

Weight of the flask containing product.

Removal of Directing Group

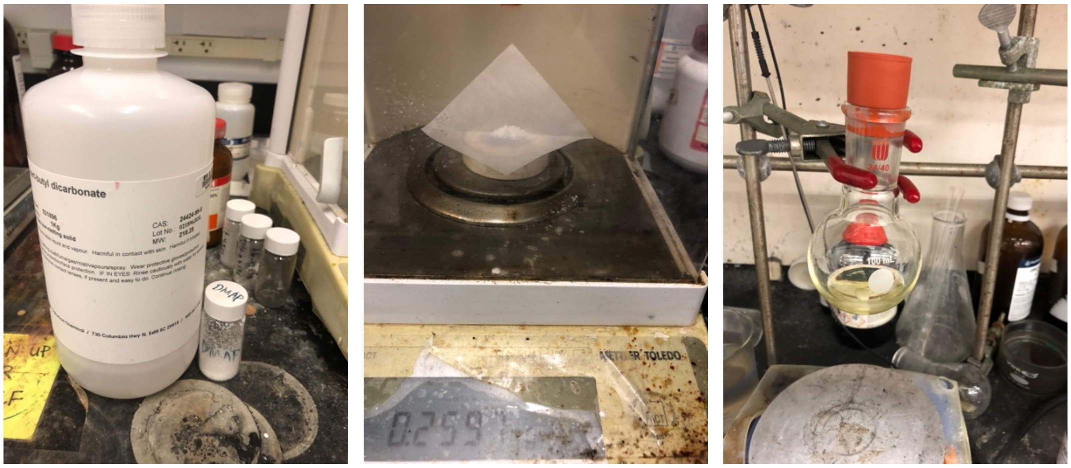

Left: Reagents. **Center:** DMAP. **Right:** After adding MeCN (5 mL).

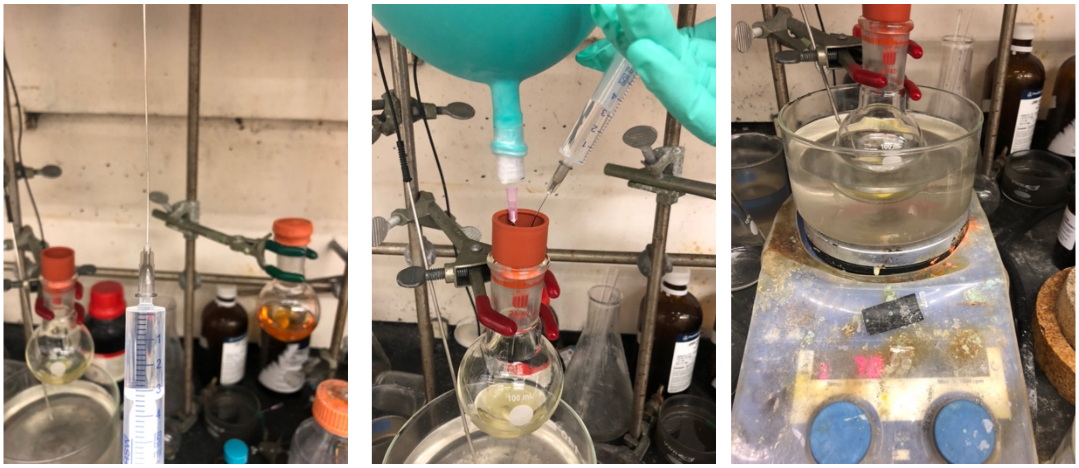

Left: Boc_2O. **Center:** Adding Boc_2O slowly. **Right:** Put the flask into a preheated oil bath (70 ºC).

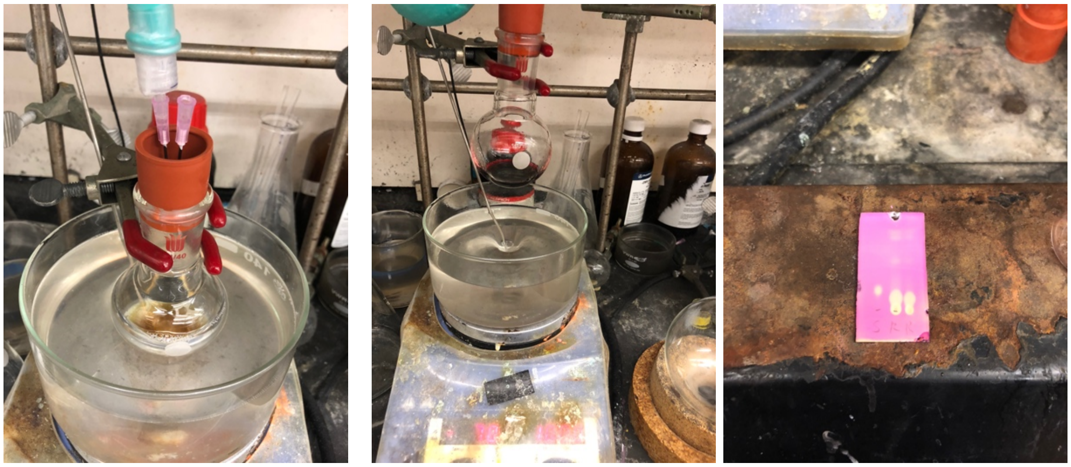

Left: Bubbling during the reaction. **Center:** Reaction completed after 6 hours. **Right:** TLC for starting material (left) and crude reaction mixture (right two, bottom spot is the product).

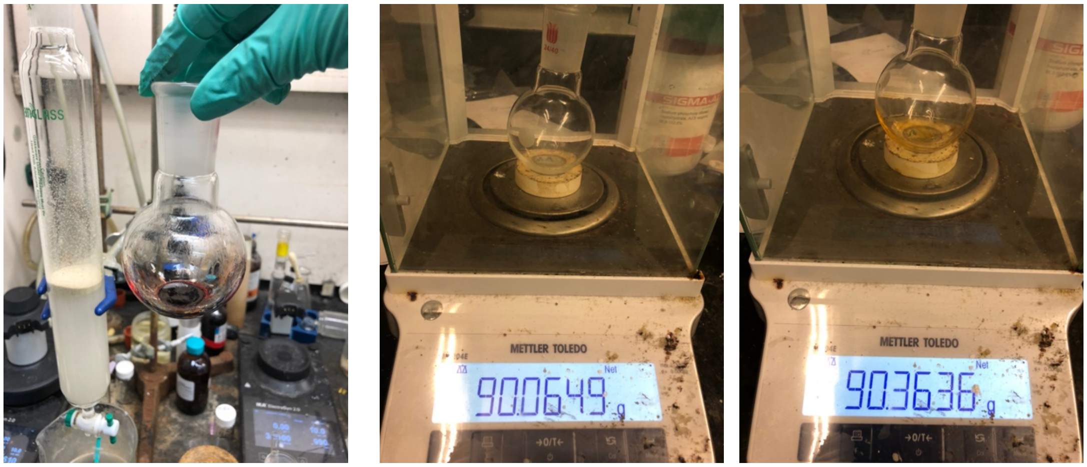

Left: Column chromatography. **Center:** Weight of the empty flask for product. **Right:** Weight of the flask containing product.

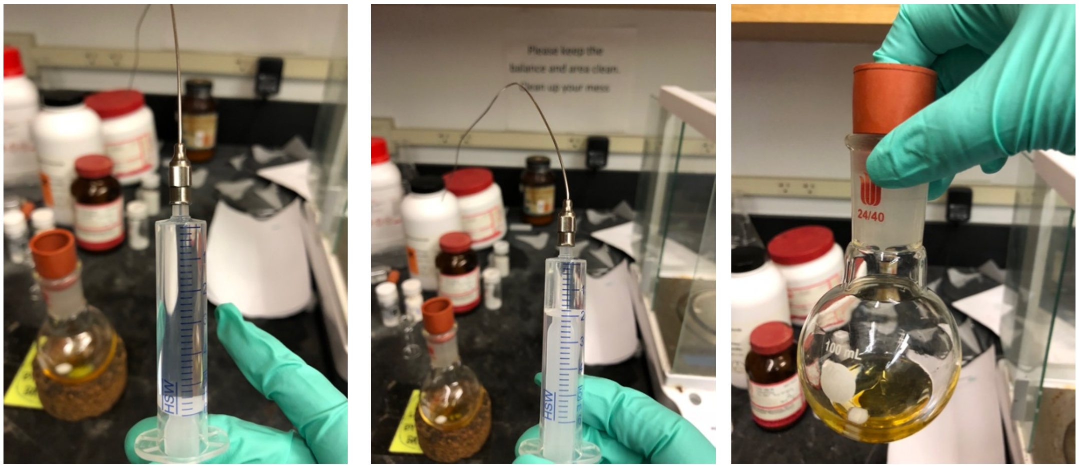

Left: THF (6 mL). **Center:** H_2O (2 mL). **Right:** After adding solvent to the reaction flask.

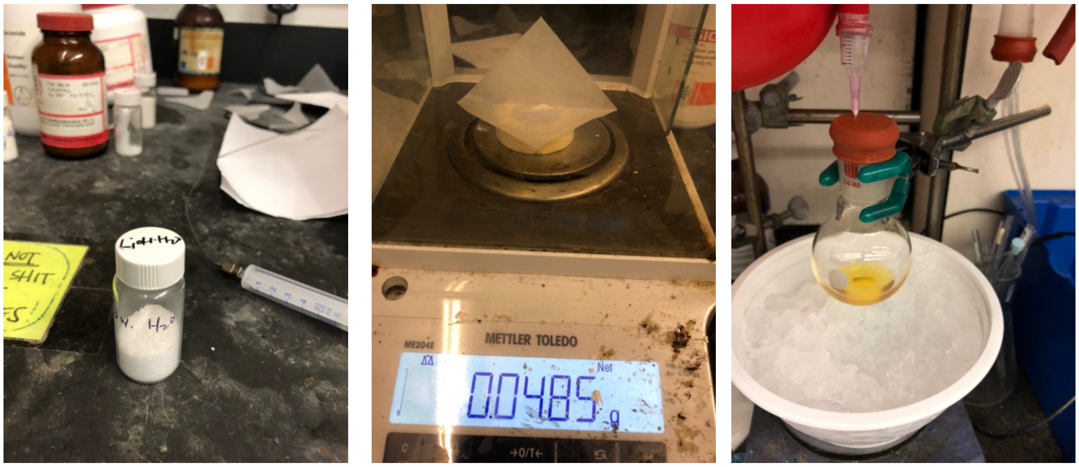

Left: LiOH•H_2O. **Center:** LiOH•H_2O (48.5 mg). **Right:** After adding LiOH•H_2O to the flask.

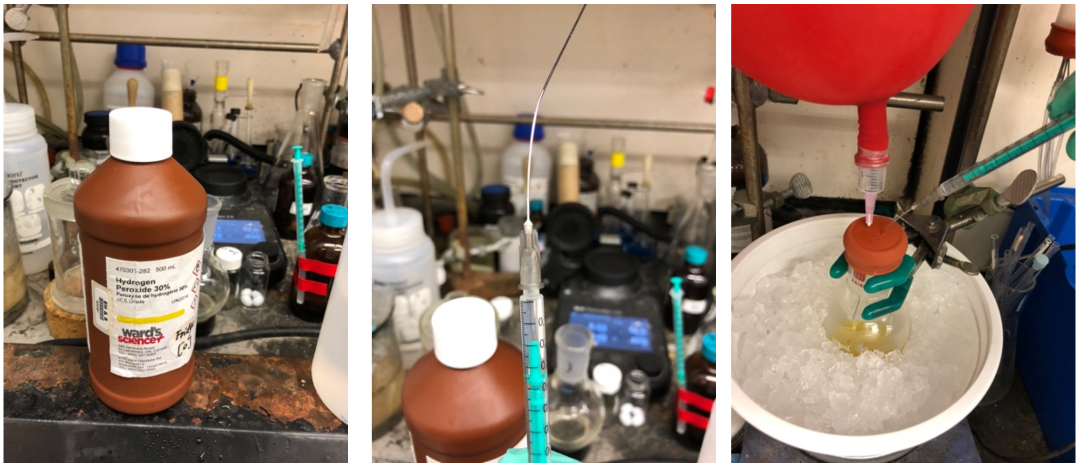

Left: H_2O_2. **Center:** H_2O_2 (0.34 mL). **Right:** adding H_2O_2 dropwise at 0 ºC.

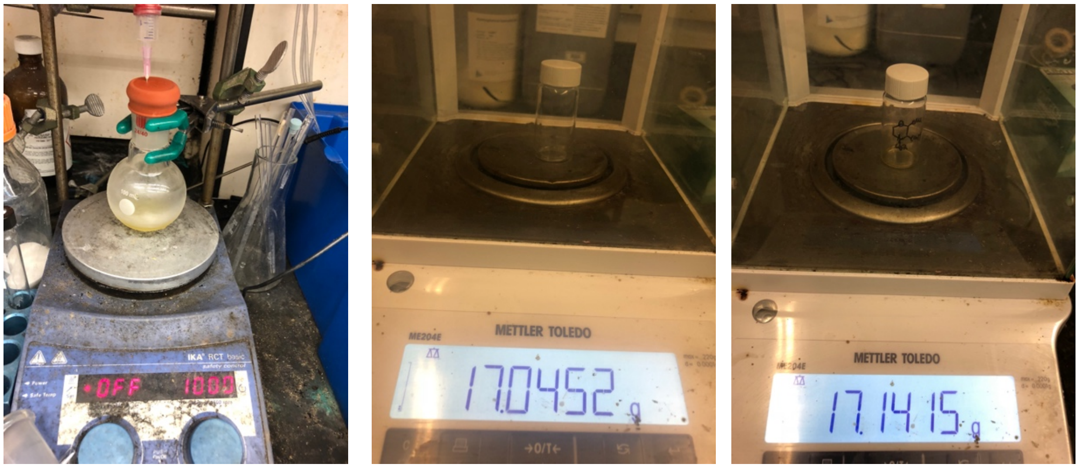

Left: Stir at room temperature overnight. **Center:** Weight of the empty flask for product. **Right:** Weight of the flask containing product.

Preparation of RAE

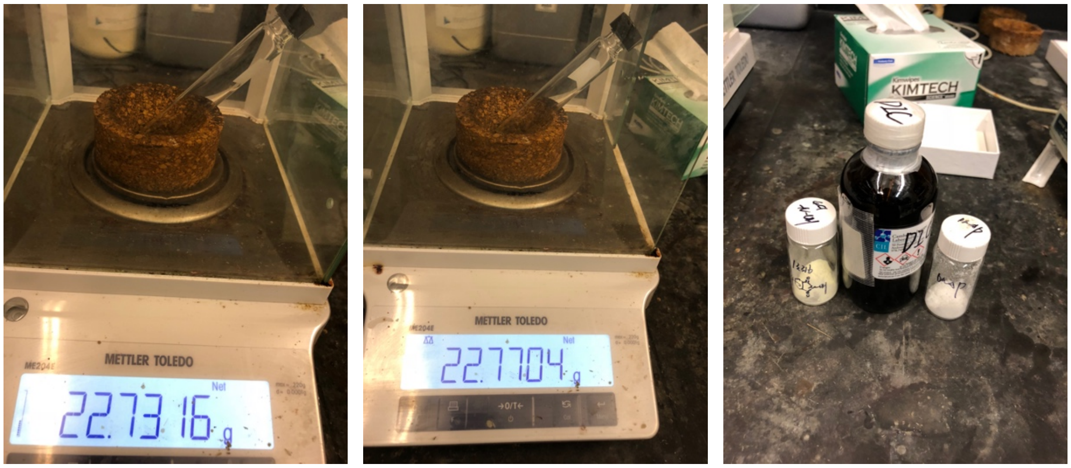

Left: Tare of the culture tube. **Center:** Addition of carboxylic acid. **Right**: Reagents.

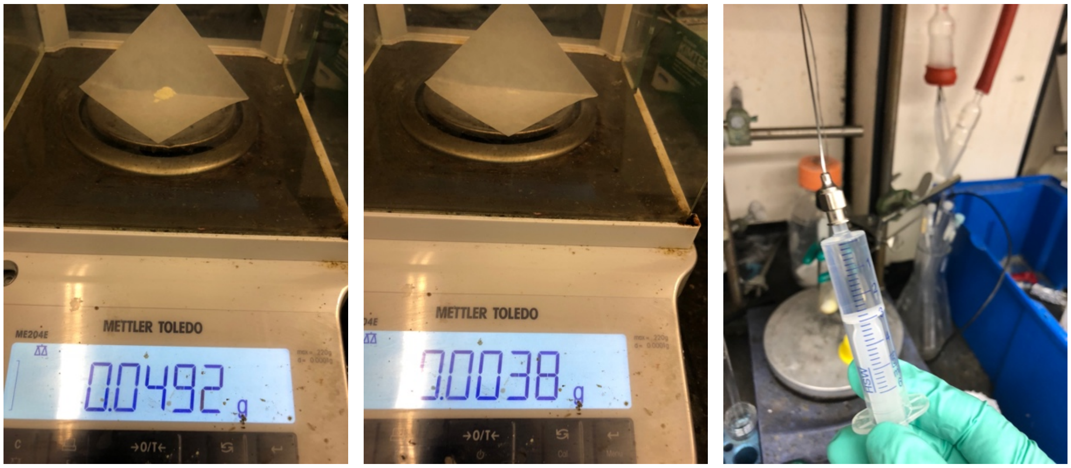

Left: TCNHPI. **Center:** DMAP. **Right**: DCM.

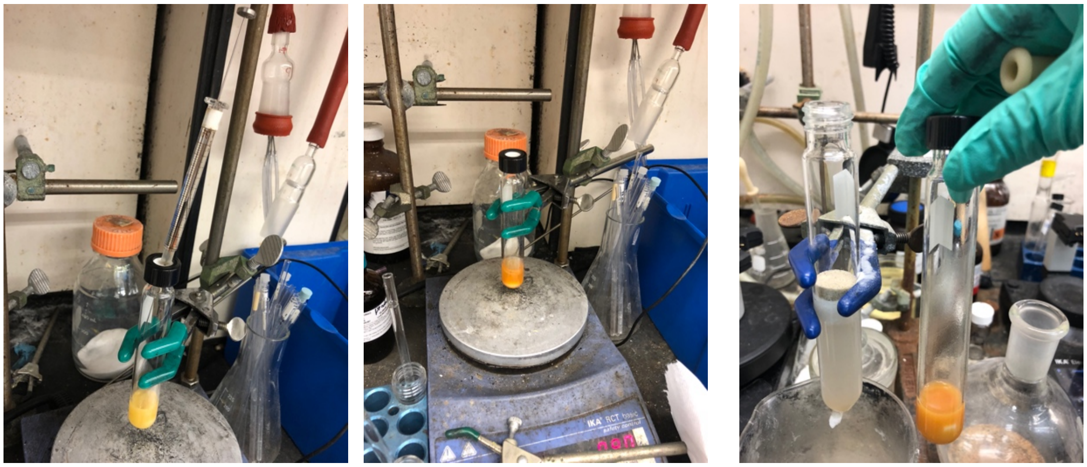

Left: Adding DIC. **Center:** stir at room temperature for 2 hours. **Right**: Column chromatography for purification.

Table S1: Optimization of C–H Methoxylation for Morpholine Scaffold

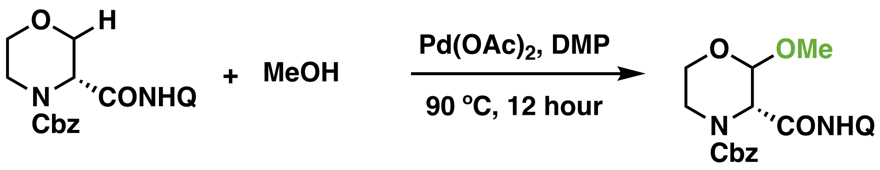

entry	$Pd(OAc)_2$ (mol%)	DMP (eq)	solvent	yield
1	10	4	MeOH	57%
2	20	2	MeOH	63%
3	50	2	MeOH	39%
4[a]	10	2	MeOH	50%
5[c]	10	2	MeOH	30%
6	10	2	MeOH/toluene	41%
7	10	2	MeOH/dioxane	25%
8	10	2	MeOH/DCE	13%

Conditons: (0.1 mmol), MeOH (1 mL), Pd(OAc) (x mol%), DMP (x equiv), 90 °C, 12h. [b]N_2. [c]O_2.

General Procedure B: Synthesis of Redox-Active Esters

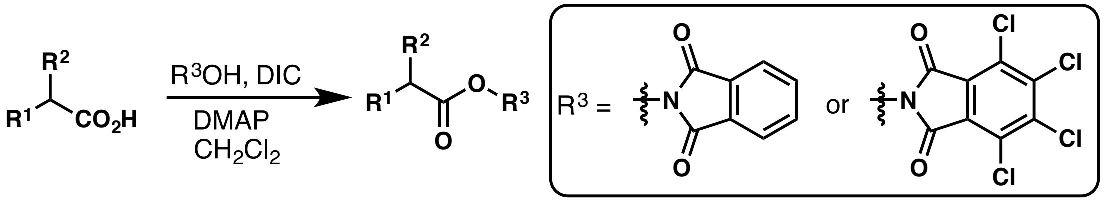

Redox-active esters were prepared according to the previously reported procedure.[6–9,11,13-15]

A round-bottom flask or culture tube was charged with (if solid) carboxylic acid (1.0 equiv.) and N-hydroxytetrachlorophthalimide or N-hydroxyphthalimide (1.0 – 1.1 equiv.). CH_2Cl_2 was added (0.1–0.2 M), and the mixture was stirred vigorously. Carboxylic acid (1.0 equiv.) was added via syringe (if liquid). DIC (1.1 equiv.) was then added dropwise via syringe, and the mixture was allowed to stir until the acid was consumed (determined by TLC). Typical reaction times were between 0.5 and 12 hours. The mixture was filtered (over Celite®, Silica gel, or through a fritted funnel) and rinsed with additional CH_2Cl_2:Et_2O. The solvent was removed *in vacuo*, and purification by column chromatography afforded the desired redox-active ester.

The following redox-active esters were prepared according to **General Procedure B**:

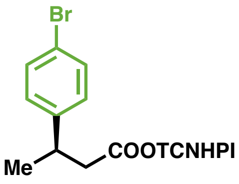

B1

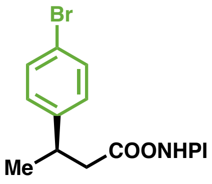

B2

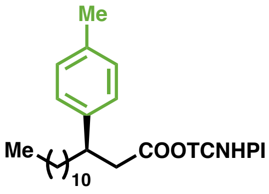

B3

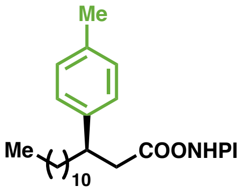

B4

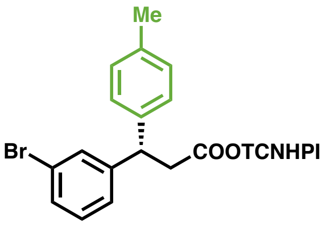

B5

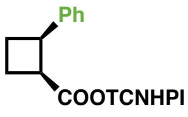

B6

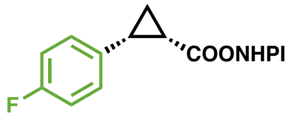

B7

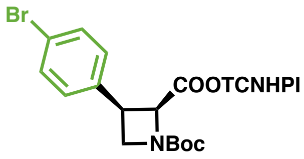

B8

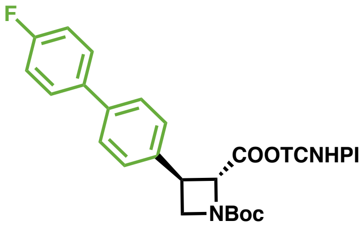

B9

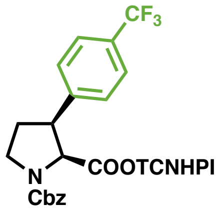

B10

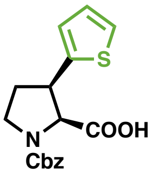

B11

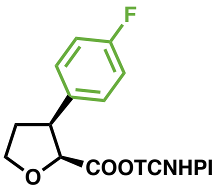

B12

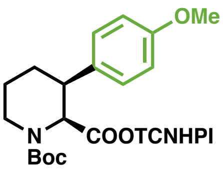

B13

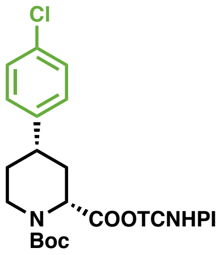

B14

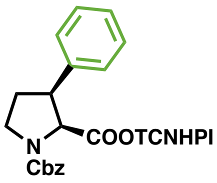

B15

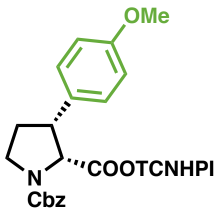

B17

Photographic Guide for Redox-active Ester Formation (General Procedure B)

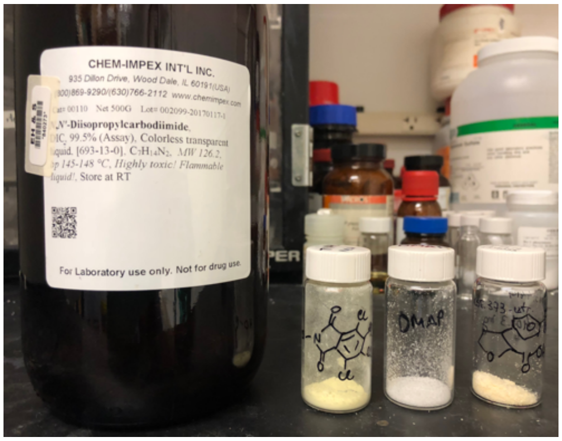

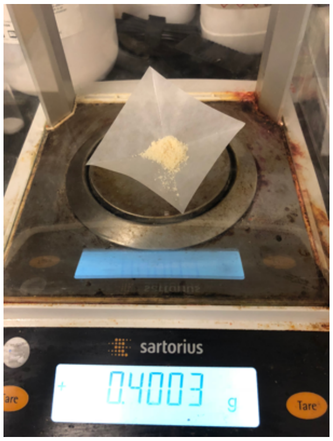

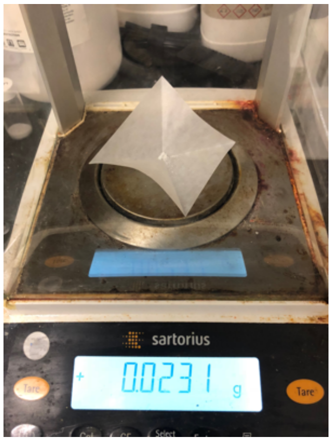

Left: Reagents. **Center:** carboxylic acid. **Right:** DMAP.

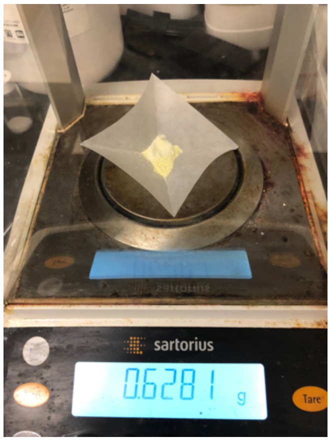

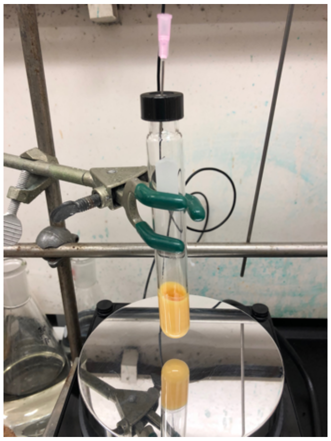

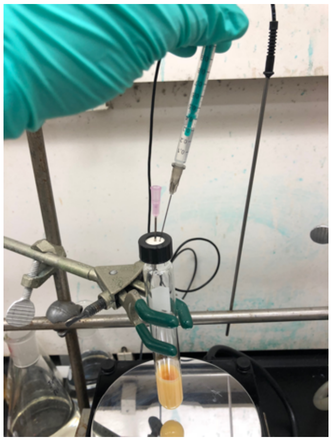

Left:. **Center:** Culture tube after addition of CH_2Cl_2. **Right:** Dropwise addition of DIC.

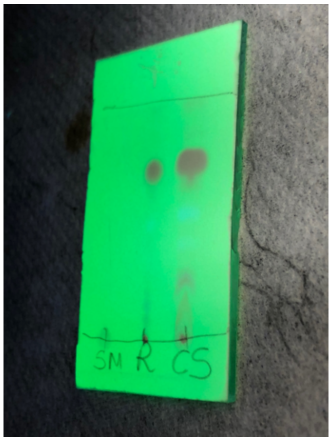

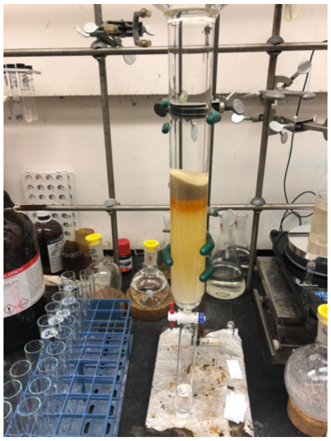

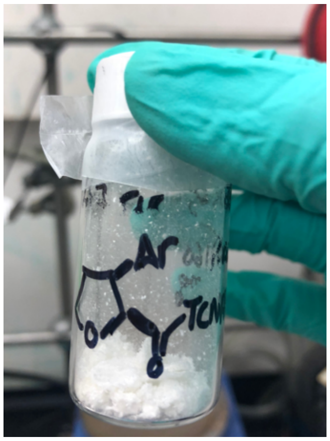

Left: TLC after 2 hours: (left) carboxylic acid; (center) reaction mixture; (right) co-spot. **Center:** Purification by flash chromatography **Right:** Final RAE.

General Procedure C1: Nickel-Catalyzed Boronic Acid Suzuki Cross-Coupling

The Nickel-catalyzed Suzuki cross-coupling of redox-active esters was performed according to a previously reported procedure.[7]

Preparation of $NiCl_2$•$6H_2O$/ligand Stock Solution (0.1 M in DMF)

A culture tube was charged with $NiCl_2$•$6H_2O$ (1 equiv.) and Bathophenanthroline (BPhen - **L6**) (1 equiv). The tube was then evacuated and backfilled with argon from a balloon 3 times. DMF (6.0 mL) was added and the resulting mixture was stirred at room temperature for at least 1 hour to give a homogeneous green solution.

Graphical Supporting Information for Preparation of $NiCl_2 \cdot 6H_2O$/Bathophenantroline

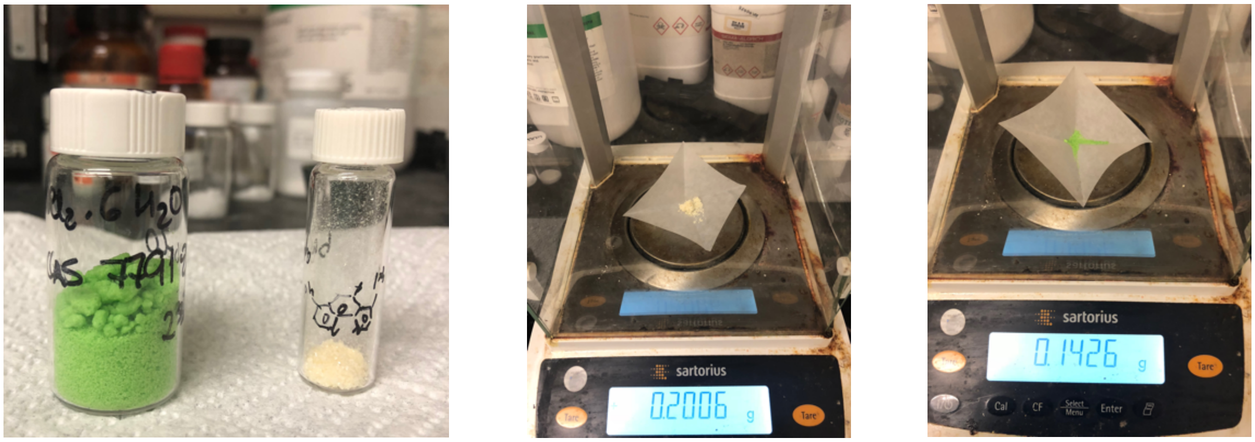

Left: Reagents. **Center:** Bathophenanthroline. **Right:** $NiCl_2 \cdot 6H_2O$.

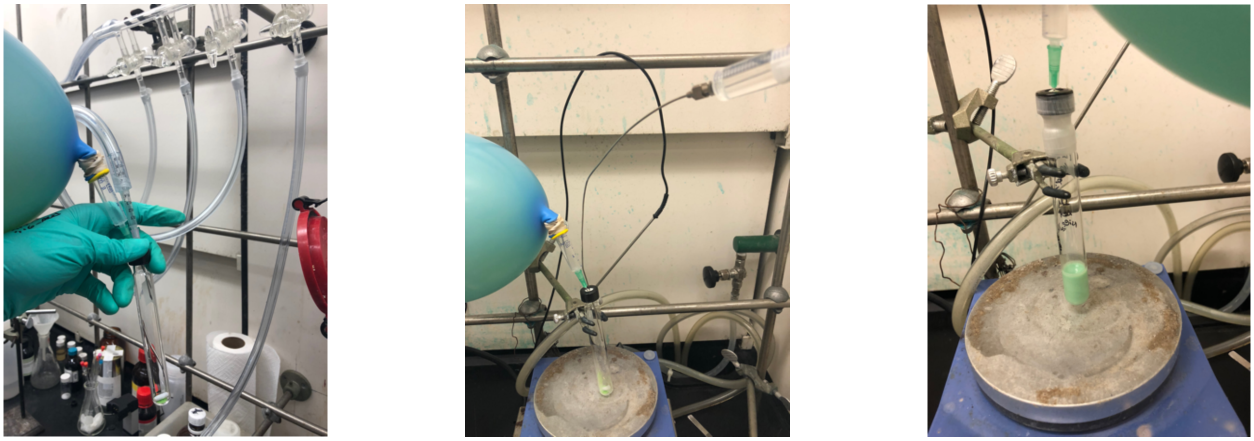

Left: Culture tube containing solids being evacuated and backfilled with Argon (3x). **Center:** Addition Dioxane. **Right:** Stock solution after 3 hours.

Procedure for the Suzuki Ni-Catalyzed Decarboxylative Cross-Coupling

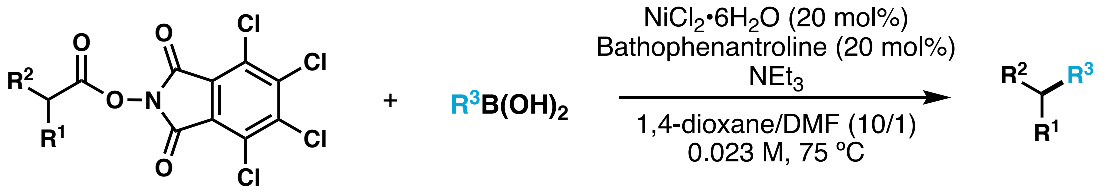

A culture tube was charged with TCNHPI RAE (1.0 equiv.), boronic acid (3.0 equiv.) and a stir bar. The tube was then evacuated and backfilled with argon from a balloon. This process was repeated for three times in total. 1,4-Dioxane (0.025 M) was added and the resulting mixture was stirred for 1 minute before NEt_3 (10.0 equiv.) was added. The mixture was stirred for 2–5 minutes until becoming homogeneous. Then, a solution of $NiCl_2$•$6H_2O$/Bathophenantroline (0.05 M in DMF, 20 mol%) was added and the tube was immediately placed in a preheated 75 ºC oil bath for 12 hours under stirring. *NOTE: It is very important that the entirety of the reaction mixture is submerged in the heated oil bath to ensure the success and reproducibility of the reaction.* After 12 hours, the reaction mixture was allowed to cool to room temperature. The mixture was then diluted with Et_2O or EtOAc, washed with 0.1 M HCl (sat. aq. NH_4Cl for acid-sensitive substrates), water and brine successively. The organic layer was dried over Na_2SO_4 and concentrated *in vacuo*. The crude product was purified by Silica gel flash column chromatography or preparative TLC (pTLC) to yield the pure compound.

The following boronic acids were used following **General Procedure C1**:

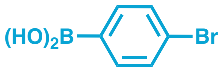

SI-6

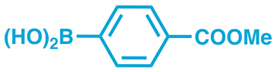

SI-7

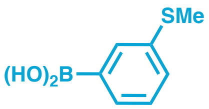

SI-8

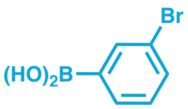

SI-9

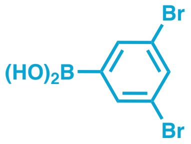

SI-10

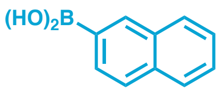

SI-11

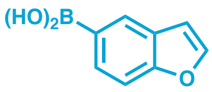

SI-12

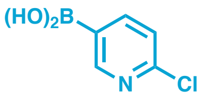

SI-13

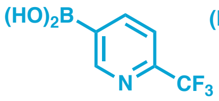

SI-14

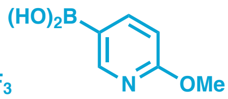

SI-15

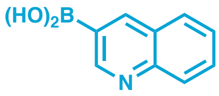

SI-16

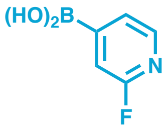

SI-17

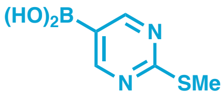

SI-18

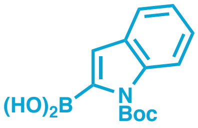

SI-19

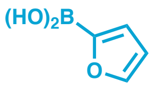

SI-20

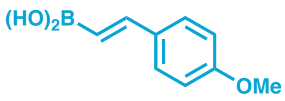

SI-21

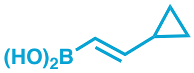

SI-22

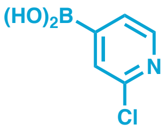

SI-23

Graphical Supporting Information for Suzuki type reaction

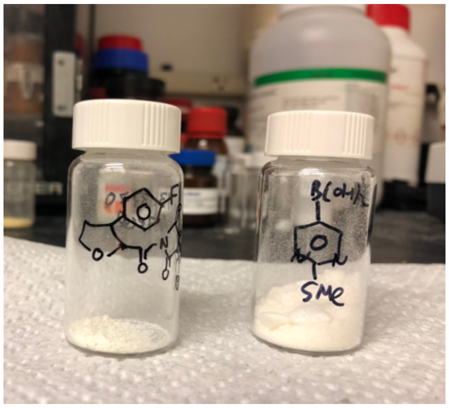

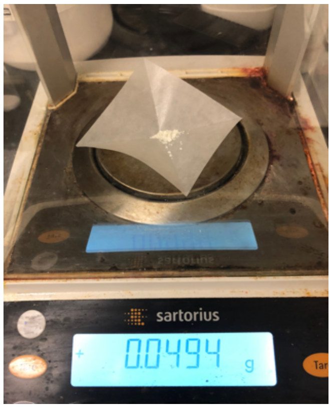

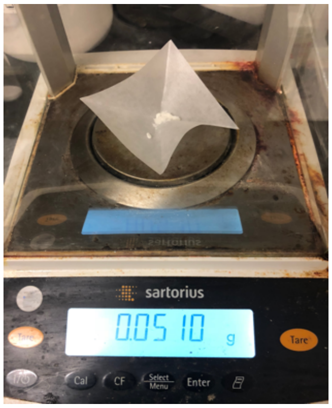

Left: Reagents. **Center:** RAE. **Right:** Boronic acid.

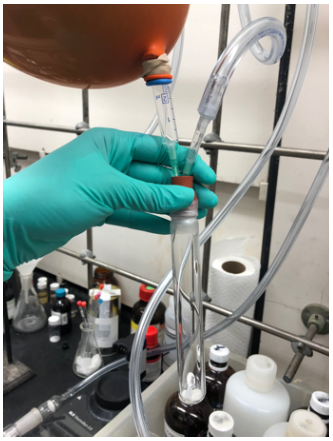

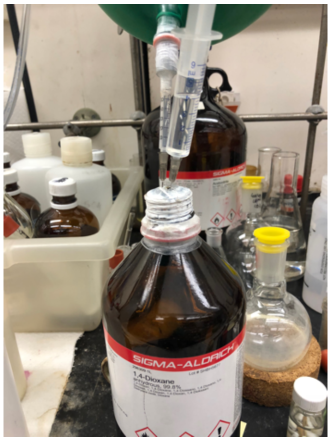

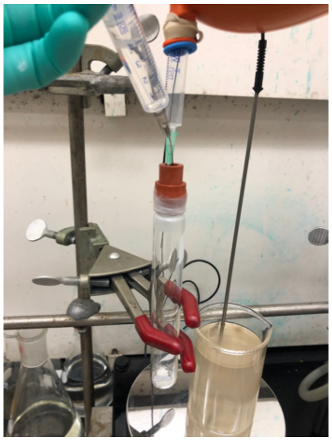

Left: Culture tube containing solids evacuated and backfilled with Argon (3x). **Center:** Dioxane (4 mL). **Right:** Addition Dioxane.

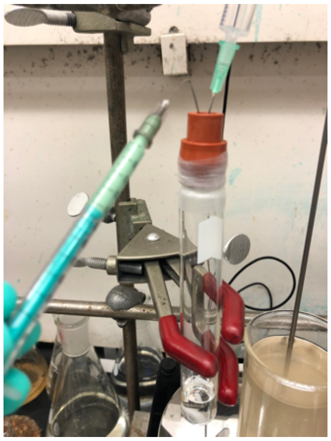

Left:

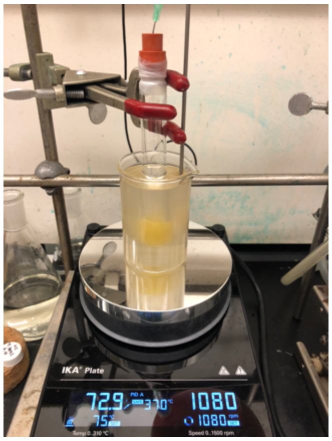

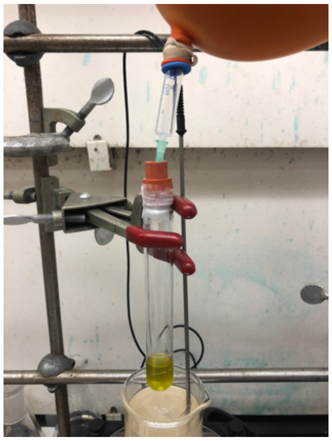

Addition of catalyst stock solution. **Center:** The culture tube placed at 75 ºC. **Right:** The reaction mixture after 12 hours.

General Procedure C2: Nickel-catalyzed Negishi Alkylation

The Nickel-catalyzed Negishi alkylation of redox-active esters was performed according to the previously reported procedure.[8] Please see this reference for graphical supporting information.

Preparation of Alkyl Zinc Reagents from Alkyl Bromides

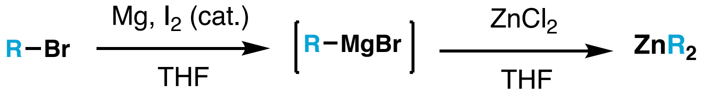

Two round bottom flasks were flame-dried and allowed to cool under vacuum. Both flasks were then backfilled with argon from a balloon. One flask was charged with Mg turnings (1.5 equiv.) and I_2 (0.01 equiv.). In the other flame-dried flask, alkyl bromide (1.0 equiv) was dissolved in anhydrous THF to make a 0.5 M solution of the alkyl bromide in THF. A small portion of the alkyl bromide solution was added to the Mg and I_2, and the mixture was stirred. The flask was heated gently with a heat gun until the dark brown color disappeared. The rest of the alkyl bromide solution was added dropwise while the flask was heated with a heat gun. After 1 hour, the resulting solution of Grignard reagent was titrated with I_2 to afford Grignard reagents with titration typically ranging 0.3–0.44 M in THF. To a separate flame-dried round-bottom flask, $ZnCl_2$ (1.0 M in THF, 1 equiv.) was added. A portion of the solution of alkyl Grignard reagent (2 equiv.) was added dropwise to the $ZnCl_2$ solution, and the mixture was stirred for at least 10 min before use. The yield was assumed to be quantitative for this step. On 0.1 mmol scale, the volume of dialkylzinc reagent solution used for the reaction was typically between 1.2–1.5 mL (corresponding to 0.2 mmol of dialkylzinc reagent).

Procedure for the Ni-catalyzed Decarboxylative Alkylation

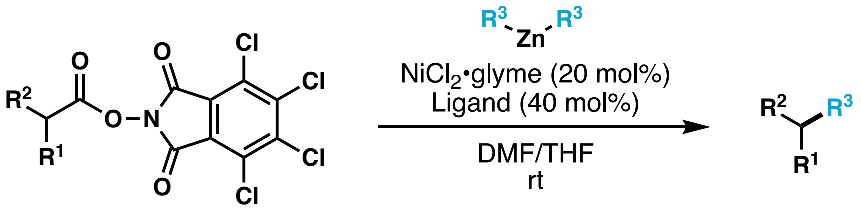

A culture tube was charged with redox-active ester (1.0 equiv.). The tube was then evacuated and backfilled with argon from a balloon. A solution of $NiCl_2$•glyme (0.2 equiv.) and ligand 2,2'-bipyridine (Bipy -**L7**) or 4,4'-di-tert- butylbipyridine (di^{t}BuBipy -**L5**) (0.4 equiv.) in DMF (0.05 M) was added. The mixture was stirred for 5 minutes. A solution of dialkylzinc (2.0 equiv.) in THF was added. The argon balloon was removed, and the culture tube was sealed with Teflon™ tape and electrical tape. The resulting mixture was allowed to stir overnight (8–16 hours) at room temperature. The reaction mixture was quenched with 1 M HCl (or half-saturated aqueous NH_4Cl solution for acid sensitive substrates) and extracted with Et_2O or EtOAc. The organic layer was washed with water and brine and dried over $MgSO_4$. The organic layer was concentrated *in vacuo*. The crude product was purified by Silica gel flash column chromatography or preparative TLC (pTLC) to yield the pure compound.

The following alkylzinc reagents were used according to **General Procedure C2**:

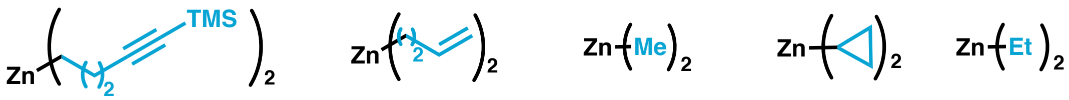

SI-24 **SI-25** **SI-26** **SI-27** **SI-28**

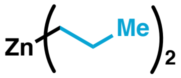

SI-51

General Procedure C3: Nickel-catalyzed Negishi Alkenylation

The Nickel-catalyzed Negishi alkenylation of redox-active esters was performed according to the previously reported procedure.[9] Please see this reference for graphical supporting information.

Synthesis of Alkenylzinc Reagents from Alkenyl Grignard Reagents

From commercial Grignard reagents (5 mmol scale)

The following Grignard reagent was purchased as solutions in THF from Sigma-Aldrich.

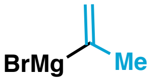

0.5 M in THF

SI-29

$ZnCl_2$ (1.0 equiv.) and LiCl (1.25 equiv.) were flame-dried under vacuum. Upon cooling, the flask was placed under argon atmosphere. Anhydrous THF (1 M) was added, and the mixture was stirred vigorously and gently heated with a heat gun until all $ZnCl_2$ and LiCl dissolved. At this time, commercial alkenyl Grignard reagent (1.0 equiv.) was added slowly via syringe to the $ZnCl_2$ and LiCl solution. Upon complete addition, the mixture was stirred for at least 15 minutes before use. The transmetallation was assumed to occur in quantitative yield.

Synthesis of Alkenylzinc Reagents from α,β-Unsaturated Alkenyl Bromide or Iodide

The following procedure was developed based on that of Knochel and coworkers.[10] A flask was charged with LiCl (2.0 equiv.) and Zn dust (2.0 equiv.). The flask was placed under vacuum and gently dried with a heat gun. Upon cooling, the flask was placed under argon atmosphere. THF (1 M) was added, and the mixture was stirred vigorously. TMSCl (0.05 equiv.) was added dropwise, and the mixture was heated to reflux with a heat gun. The mixture was then allowed to cool to room temperature, 1,2-dibromoethane (0.05 equiv.) was added dropwise, and the mixture was heated to reflux with a heat gun. Upon cooling, the flask was placed in a room temperature water bath. Next, if using an α,β-unsaturated alkenyl iodide the substrate (1.0 equiv.) was added dropwise. If using an α,β-unsaturated alkenyl bromide, the substrate was added in one portion via syringe. The mixture was then stirred at ambient temperature for 1 hour resulting in a green or red colored solution. _Note:_ If the color does not change within 5–10 minutes, it is likely that the zinc insertion reaction did not proceed. If this is the case, gently heat the reaction mixture with a heat gun. For future reactions, consider activating the Zn dust by washing with 1 M HCl, rinsing the Zn with water, *i*PrOH, MeOH, and Et_2O, and drying under vacuum. At this time, stirring was

stopped and the remaining zinc was allowed to settle to the bottom of the flask. The concentration was determined by titration with I_2.

Note: For some α,β-unsaturated alkenyl zinc reagents, the titration equivalence point is difficult to determine due to the color of the zinc reagent. In these cases, the equivalence point can either be approximated (it is possible to see the consumption of some I_2) or determined by quantitative ^1H NMR or GC/FID analysis. During the course of zinc insertion, small amounts of isomerization were noted for the zinc reagent derived from **SI-30**. Depending on the batch, typically the isomerization was from 0 to 10% to the undesired olefin isomer. We recommend working up the titrated organozinc solution and analyzing the crude product by ^1H NMR spectroscopy to determine the isomeric ratio of the organozinc reagent (as the vinyl iodide). Therefore, depending on the quality of the organozinc reagent, 0 to 10% of the undesired isomer was obtained as the coupling product. Only the isolated yields for substrates from **SI-30** have been reported; the cross-coupling of the undesired isomer was not included in the yield. For all cases in this work, small amounts of the undesired isomer could be separated by flash column chromatography.

The following α,β-unsaturated alkenyl halide was used according to a previously reported procedure.[9]

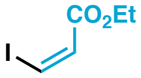

SI-30

Procedure for the Ni-catalyzed Decarboxylative Alkenylation

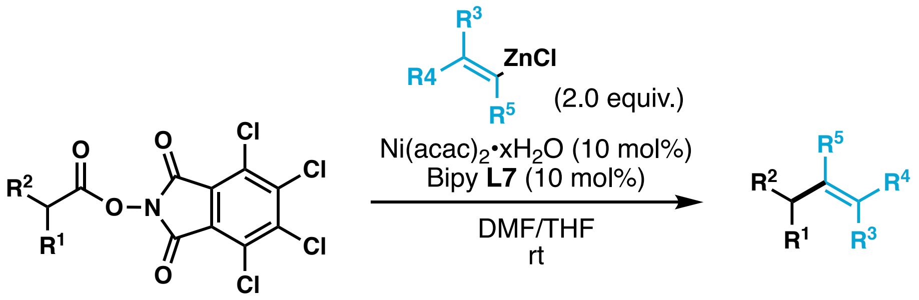

TCNHPI redox-active ester (1.0 equiv.) was added to a culture tube, which was sealed with a Teflon®-septum cap and wrapped in Parafilm®. The culture tube was evacuated and refilled with Argon three times. A solution of $Ni(acac)_2$•xH_2O (0.1 equiv.) and Bipy **L7** (0.1 equiv.) in DMF (0.1 M relative to substrate) was added to the tube, and the mixture was vigorously stirred. A solution of alkenylzinc reagent (2.0 equiv.) in THF was then added, and the mixture was allowed to stir at room temperature overnight (6–12 h) or until all starting material was consumed as determined by TLC analysis. The reaction mixture was then diluted with EtOAc or Et_2O, quenched with 1M HCl, and extracted. Organic layers were dried over $MgSO_4$, filtered and concentrated to afford the crude product, which was purified by preparative TLC (pTLC) or column chromatography.

For alkenylzinc reagent prepared from **SI-30**, $MgBr_2$•OEt_2 was added in stoichiometric amount relative to the alkenylzinc, and the addition of catalyst solution to culture tube was at 0 ºC using ice/water bath.

The following alkylzinc reagents were used following **General Procedure C3**:

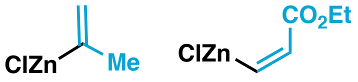

SI-31 SI-32

General Procedure C4: Nickel-catalyzed Negishi Arylation

The Nickel-catalyzed Negishi arylation of redox-active esters was conducted according to a previously reported procedure.[11] Please see this reference for graphical supporting information.

Preparation of $ZnCl_2$ Solution (1.0 M in THF)

A Schlenk flask equipped with stir bar was flame-dried and allowed to cool to room temperature under vacuum. The flask was backfilled with nitrogen, and $ZnCl_2$ (1 equiv.) was added. The flask was placed under vacuum and heated in an oil-bath preheated to 150 ºC. After stirring for 2–12 hours, the flask was removed from the oil bath and allowed to cool to room temperature. The flask was backfilled with nitrogen, and THF (1 M) was added. The mixture was vigorously stirred until all $ZnCl_2$ was dissolved (approximately 12 hours). However, if necessary, the solution can be used before all solids have completely dissolved without a decrease in yield of the subsequent reaction.

Preparation of the Arylzinc Reagents

Phenyl Zinc reagent was prepared according **General Procedure C4**, from the commercially available phenylmagnesiumgrignard reagent purchased as solutions in THF from Sigma-Aldrich.

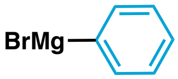

1 M in THF

SI-33

Arylzinc reagent **SI-36** was prepared from **SI-34** by a method developed by Knochel.[12] To an oven-dried culture tube backfilled with argon (3x) was added **SI-34** (1 equiv.) and THF (0.3 M). Then a solution of *i*PrMgBr•LiCl (1.3 M in THF, 1 equiv.) was added dropwise at −60 ºC. After stirring for 30 minutes, iodo-lithium exchange was completed (as determined by quenching an aliquot with water and confirming the disappearance of the aryl iodide by ^{1}H NMR). Then, a $ZnCl_2$ solution (1.0 M in THF, 1 equiv.) was added to the mixture at −60 ºC. After stirring for 10 minutes at −60 ºC, the culture tube was put into an ice bath. The solution of ArZnCl•LiCl was stirred until it became clear and was titrated with I_2 before being used for the coupling reaction.

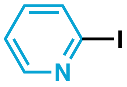

SI-34

Titration of Arylzinc Reagents

LiCl (5 equiv.) was added to a septum-containing screw-capped culture tube equipped with a stir bar. The culture tube was flame-dried under vacuum and cooled under flow of argon from a balloon. The cap was removed, I_2 (1.0 equiv.) was quickly added to the culture tube, and the cap was replaced. The exact amount of I_2 added was recorded. Anhydrous THF (0.1 M) was added, and the mixture was stirred for 5 minutes to afford a dark brown solution. A 1.00 mL syringe was filled with approximately 0.90 mL of ArZnCl•LiCl, and the solution was added dropwise via the syringe. The exact volume of ArZnCl•LiCl solution in the syringe was recorded (titration start point). Over the course of the titration the color changes from dark brown to light brown to yellow to colorless, indicating complete consumption of I_2, and upon consumption of I_2, the titration end point was recorded. The concentration of the ArZnCl•LiCl solution was then calculated. Typical concentrations of arylzinc reagents ranged from 0.18 M to 0.24 M in THF.

The following alkylzinc reagents were used according to **General Procedure C4**:

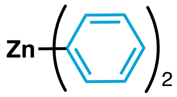

SI-35

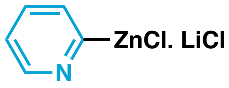

SI-36

Procedure for the Ni-catalyzed Decarboxylative Arylation

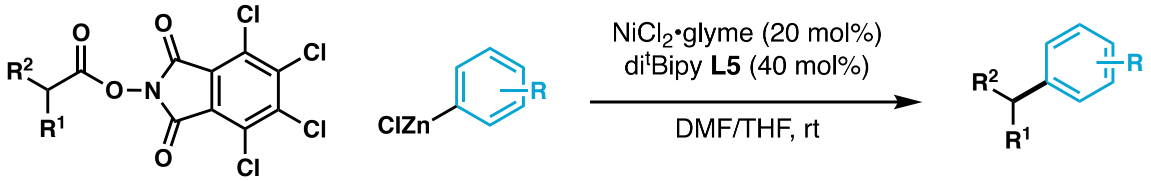

A culture tube with a Teflon™ septum screw-cap and stir bar was charged with $NiCl_2$•glyme or $NiCl_2$•$6H_2O$ (20 mol%), di*t*BuBipy **L5** (40 mol%) and (if solid) TCNHPI ester (1.0 equiv.). The tube was evacuated and backfilled with argon. Reactions were run with a 3:2 ratio of THF:DMF. The volume of DMF used was calculated based on the titration of the arylzinc reagent solution. DMF (anhydrous) was added via syringe, and the mixture stirred for 2 minutes at room temperature for solid TCNHPI-esters. TCNHPI-ester (if liquid) was dissolved in DMF (anhydrous) and added to the culture tube containing $NiCl_2$•glyme or $NiCl_2$•$6H_2O$ (20 mol%) and di*t*BuBipy **L5** (40 mol%). The mixture was stirred for 2 minutes at room temperature. Then, arylzinc reagent in THF (3.0 equiv.) was added in one portion, and the mixture was stirred for 12–16 hours at room temperature. The mixture was diluted with EtOAc or Et_2O and quenched with 1 M HCl (aq.). The reaction can also be quenched with H_2O or half-saturated aq. NH_4Cl solution for acid-sensitive substrates. The organic layer was washed with H_2O and brine, dried over anhydrous Na_2SO_4, and concentrated *in vacuo* (CAUTION: some of the products are volatile). The crude material was purified by silica gel column chromatography or preparative TLC (pTLC).

General Procedure C5: Nickel-catalyzed Negishi Ethynylation

The Nickel-catalyzed Negishi ethynylation of redox-active esters was conducted according to a previously reported procedure.[13] Please see this reference for graphical supporting information.

Preparation of Nickel/ligand Solution

A culture tube was charged with $NiCl_2$•$6H_2O$ (1 equiv.) and 4,4'-di-OMe-bipyridine (diOMeBipy - **L8)** (1 equiv.) under argon atmosphere. Anhydrous DMF (0.02 M) was added, and the homogeneous solution was stirred at room temperature for 15 minutes before being used.

Preparation of Ethynylzinc Chloride Solution

A flame-dried tube was charged with of freshly prepared 1 M $ZnCl_2$/LiCl THF solution (2 equiv.), then ethynylmagnesium bromide (1.0 equiv., 0.5 M THF solution, Sigma-Aldrich) was added dropwise at room temperature. The resulting solution was stirred at room temperature for 30 minutes until it became homogeneous. The resulting ethynylzinc solution was then titrated with I_2 before use.

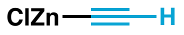

SI-37

Procedure for the Ni-catalyzed Decarboxylative Ethynylation

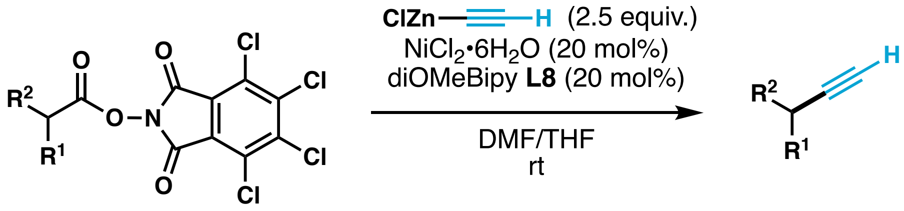

A culture tube was charged with TCNHPI redox-active ester (1.0 equiv.) and a stir bar. The tube was then evacuated and backfilled with argon from a balloon. A solution of $NiCl_2$•$6H_2O$/**L8** (0.02 M in DMF, $NiCl_2$•$6H_2O$ (20 mol%), **L8** (20 mol%)) and ethynylzinc chloride solution in THF (2.5 equiv.) were added in quick succession. The reaction mixture was then stirred at room temperature for 12 h. The mixture was then quenched with 1 M HCl and extracted with EtOAc or Et_2O three times, and the organic layer was dried over $MgSO_4$. The organic layer was concentrated *in vacuo* at 35 °C. The crude product was purified by silica gel flash column chromatography or preparative TLC (pTLC) to afford the desired compound.

General Procedure C6: Iron-catalyzed Kumada Alkynylation

The Iron-catalyzed Kumada alkynylation of redox-active esters was conducted according to a previously reported procedure.[13] Please see this reference for graphical supporting information.

Preparation of Substituted Alkynylmagnesium Bromide Solution

A flame-dried tube was charged with alkyne (1.0 equiv.) and anhydrous THF (1 M). After that, freshly titrated ethylmagnesium bromide (1.0 equiv.) THF solution was added dropwise at room temperature. Note: gas was generated during the addition. The resulting solution was stirred at 50 ºC for another 30 minutes. After cooling down to room temperature, the resulting Grignard solution was titrated with I_2 before use.

The following alkynylgrignard reagents were used according to **General Procedure C6**:

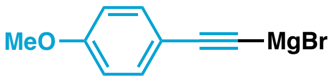

SI-38

Procedure for the Fe-catalyzed Decarboxylative Alkynylation

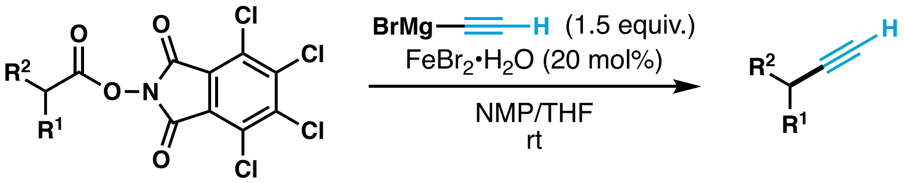

A culture tube was charged with TCNHPI redox-active ester (1.0 equiv.), $FeBr_2$•H_2O (20 mol%) and a stir bar. The tube was then evacuated and backfilled with argon from a balloon. The solid was dissolved in NMP (0.1 M) and cooled down to –15 ºC. The prepared alkynylmagnesium bromide THF solution (1.5 equiv.) was added in one portion. After stirring for another 15 minutes, the reaction was quenched with 1 M HCl and extracted with Et_2O three times, and the organic layer was dried over $MgSO_4$. The organic layer was filtered through a short silica gel plug and concentrated *in vacuo* at 35 ºC. The crude product was purified by silica gel flash column chromatography or preparative TLC (pTLC) to afford the desired product.

General Procedure C7: Nickel-catalyzed Boronic Ester Suzuki Cross-Coupling

The Nickel-catalyzed Boronic Ester Suzuki of redox-active esters was conducted according to a previously reported procedure.[14] Please see this reference for graphical supporting information.

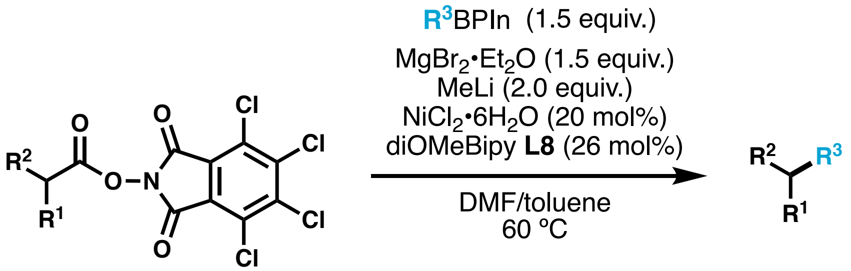

Preparation of Nickel/ligand Solution

A culture tube charged with $NiCl_2$•$6H_2O$ (1 equiv.) and diOMeBipy **L8** (1.36 equiv.) was evacuated and backfilled with argon three times. DMF (0.05 M) was added and the resulting mixture was stirred at room temperature overnight to afford a pale green suspension.

Preparation of B_2pin_2/MeLi-Complex Solution

A flame-dried culture tube was charged with B_2pin_2 (1 equiv.) under argon atmosphere and anhydrous tetrahydrofuran (1 M) was added. A solution of MeLi (1.6 M in Et_2O,1 equiv.) was added at 0 ºC and the resulting mixture was stirred at room temperature for 1 hour before use.

Procedure for the Ni-catalyzed Decarboxylative Cross-Coupling

A culture tube charged with TCNHPI redox-active ester (1.0 equiv.) and $MgBr_2$•OEt_2 (1.5 equiv.) was evacuated and backfilled with argon from a balloon 3 times. THF (0.12 M) was added, and the mixture was stirred until no granular $MgBr_2$•OEt_2 was observed. The Nickel/ligand solution ($NiCl_2$•$6H_2O$ (20 mol%), diOMeBipy **L8** (26 mol%)) was then added via a syringe and the resulting mixture was stirred vigorously until no visible solid was observed on the bottom of the reaction vessel. This mixture was cooled to 0 ºC before a suspension of [B_2pin_2Me]Li in THF (3.3 equiv.) was added in one portion. After one hour, reaction was warmed to room temperature and stirred for an additional hour before being quenched with 0.1 M HCl. The reaction was then extracted with EtOAc (3x). The organic layers were then combined, dried over Na_2SO_4, filtered and concentrated. Purification by flash column chromatography afforded the desired compound.

General Procedure C8: Nickel-Catalyzed Giese Conjugate Addition

The Nickel-catalyzed Giese Conjugate addition of redox-active esters was conducted according to a previously reported procedure.[15] Please see this reference for graphical supporting information.

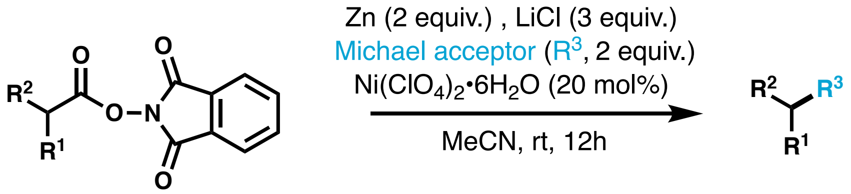

A culture tube was charged with LiCl (3.0 equiv.). *Note: Due to its hydroscope nature, LiCl can be difficult to weigh on small scale. In our experience excess LiCl is not detrimental to the success of the reaction.* Next, NHPI RAE (1.0 equiv.), Zn powder (2.0 equiv.), and $Ni(ClO_4)_2$•$6H_2O$ (20 mol%) were added. A stir bar was added, and the culture tube was evacuated. The tube was backfilled with argon from a balloon, and Michael acceptor (2.0 equiv.) was added via syringe. To the reaction mixture was added MeCN (0.4 M), and the mixture was stirred overnight at room temperature. After at least 12 hours, a H_2O and sat. aq. NH_4Cl solution (1:1) were added. The mixture was extracted with EtOAc or Et_2O (3x), and the organic layer was dried over $MgSO_4$. The crude was purified by Silica gel flash column chromatography or preparative TLC (pTLC) to afford the desired compound.

Troubleshooting: Frequently Asked Questions

C–H Activation

Question 1:

How do I monitor the reaction?

Answer:

We use TLC analysis with UV visualization and staining ($KMnO_4$) to monitor the reaction. Usually, it takes 24 hours for completion of the C–H activation step.

Question 2:

Can I run the C–H activation reaction in round bottom flask adapted with condenser instead of sealed tubes?

Answer: For large scale reaction (>2 g substrate) on azetidine heterocycle scaffolds, the reaction was carried out using a round bottom flask adapted with condenser without impacting the yield.

Question 3:

Does the enantiopurity of my compounds erode under hydrolysis conditions?

Answer: No, we transformed the carboxylic acid products into the corresponding RAEs and determined their *ee* by chiral HPLC. The RAEs have the same the *ee* compared with the corresponding C–H activation products, which means enantiopurity doesn't erode under hydrolysis conditions

Question 4:

Why are different equivalents of Boc_2O required for Boc-protection of aminoquinoline group?

Answer: This is due to different steric hindrance of arylated heterocycle. Four and five member-ring heterocycles possess higher reactivity than six member-ring towards Boc-protection, which causes different loading of Boc_2O in the Boc-protection step.

Question 5:

Is it possible to decrease the loading of Pd catalyst in C-H activation?

Answer: In order to obtain high yield for the C–H activation step, we usually used 10 mol% Pd loading. However, for some highly reactive substrates such as pyrrolidine, the catalyst loading

could be decreased to 5 mol% affording the product in comparable yield.

Question 6:

Why was only arylation achieved for C–H activation step?

Answer: We have performed some other transformation such as alkenylation, alkynylation, fluorination on heterocyclic substrates. Although the C–H activation reaction proceeded well, the following removal of directing group turns out to be problematic either because of the steric hindrance or elimination of HF to afford the corresponding unsaturated amide directing group by-product.

Decarboxylative Cross-Couplings

Question 1:

What are the advantages of using *N*-hydroxy-tetrachlorophthalimide (TCNHPI) over *N*-hydroxy-phthalimide (NHPI) redox-active esters?

Answer:

For the decarboxylative cross-couplings conducted in this manuscript, the yields were in general higher starting from TCNHPI redox-active esters compared to NHPI redox-active esters. Moreover, the high crystallinity of the TCNHPI redox-active esters facilitate their purification and offer the possibility to upgrade the enantiomeric excess by recrystallization. In general, better enantioenrichment was achieved with the solvent system (Hexanes:EtOAc) compared to (CH_2Cl_2:MeOH).

Question 2:

Does the enantiopurity erode under certain DCC conditions?

Answer: We have compared the *ee* of final products obtained after the DCC reaction with the corresponding RAEs. Both have comparable *ee* indicating that enantiopurity doesn't erode under DCC conditions.

Question 3:

I want to synthesize a large quantity of a decarboxylative cross-coupling product. Can I increase the concentration in order to lower the amount of solvent?

Answer:

When working on a larger scale we typically increase the concentration to 0.5–1 M without any detrimental effect on the yield. However, Suzuki couplings are very sensitive to concentration and should always be conducted under standard conditions.

Question 4:

Suzuki reactions seem to be the most convenient to setup, are there any limitations in terms of substrate compatibility?

Answer: Although Suzuki reaction is among one of the most robust reaction that we have developed, it still has some limitations. For example, alkyl boronic acids are completely unreactive for this reaction. For some heterocycles, such as pyrazole, imidazole, we need to use the corresponding Bpin esters instead of boronic acids.

Question 5:

Do I need a glovebox to run the decarboxylative cross-coupling reactions?

Answer: A glovebox is not necessary to run this reaction. Typically, reaction vessels are degassed and refilled with argon three times before running the reactions. The reactions are also not very sensitive to water. The reactions can even be run in an open-flask and almost no erosion in yield is observed.

Experimental Procedures and Characterization Data for Linear and Strained-Ring Systems (Figure 2)

Compound B1

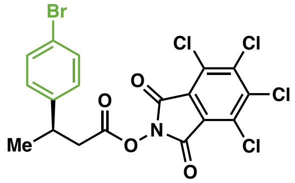

4,5,6,7-tetrachloro-1,3-dioxoisoindolin-2-yl (*R*)-3-(4-bromophenyl)butanoate (B1)

Following General Procedure **B** on 0.2 mmol scale with **A1**. Purification by recrystallization from CH_2Cl_2:MeOH afforded 81.3 mg (77%) of the title compound **B1**.

Physical State: pale yellow solid.

m.p.: 125 – 132 ºC.

^{1}H NMR (600 MHz, $CDCl_3$): δ 7.48 – 7.45 (m, 2H), 7.17 – 7.13 (m, 2H), 3.36 (h, J = 6.9 Hz, 1H), 2.95 – 2.84 (m, 2H), 1.43 (d, J = 7.0 Hz, 3H) ppm.

^{13}C NMR (151 MHz, $CDCl_3$): δ 167.7, 157.6, 143.4, 141.2, 132.0, 130.7, 128.6, 124.8, 120.9, 39.4, 36.2, 21.4 ppm.

TLC: R_f = 0.30 (9.5:0.5 Hexanes:EtOAc).

$[\alpha]_D^{20}$ = –29.8 (c = 0.104, $CHCl_3$).

*<u>Note:</u> no HRMS could be recorded for **B1***

Compound B2

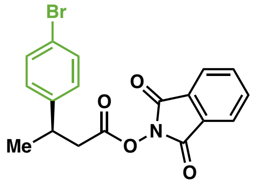

1,3-dioxoisoindolin-2-yl (*R*)-3-(4-bromophenyl)butanoate (B2)

Following General Procedure **B** on 0.145 mmol scale with **A1**. Purification by flash column chromatography (Silica, 9:1 Hexanes:EtOAc) afforded 19.6 mg (35%) of the title compound **B2**.

Physical State: amorphous white solid.

m.p.: 86 – 92 ºC.

^{1}H NMR (600 MHz, $CDCl_3$): δ 7.88 (dd, *J* = 5.5, 3.1 Hz, 2H), 7.79 (dd, *J* = 5.5, 3.1 Hz, 2H), 7.49 – 7.44 (m, 2H), 7.18 – 7.14 (m, 2H), 3.38 (dt, *J* = 8.3, 6.8 Hz, 1H), 2.95-2.85 (m, 1H), 2.87 (dd, *J* = 15.5, 8.3 Hz, 1H), 1.43 (d, *J* = 7.0 Hz, 3H) ppm.

^{13}C NMR (151 MHz, $CDCl_3$): δ 168.2, 162.0, 143.6, 134.9, 132.0, 129.0, 128.6, 124.1, 120.8, 39.5, 36.1, 21.5 ppm.

*Note: no HRMS could be recorded for **B2***

TLC: R_f = 0.26 (4:1 Hexanes:EtOAc).

$[\alpha]_D^{20}$ = -41.0 (*c* = 0.20, $CHCl_3$).

Compound B3

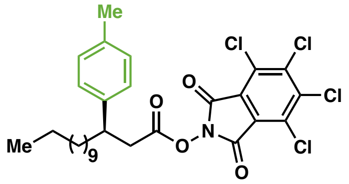

4,5,6,7-tetrachloro-1,3-dioxoisoindolin-2-yl (*R*)-3-(*p*-tolyl)tetradecanoate (B3)

Following General Procedure **B** on 0.28 mmol scale with **A2**. Purification by recrystallization from CH_2Cl_2:MeOH afforded 106 mg (63%) of the title compound **B3**.

Physical State: pale yellow solid.

m.p.: 125-132 ºC.

^{1}H NMR (600 MHz, CDCl$_3$): δ 7.14 (d, J = 8.0 Hz, 2H), 7.11 (d, J = 8.1 Hz, 2H), 3.17 – 3.10 (m, 1H), 2.91 (qd, J = 15.5, 7.4 Hz, 2H), 2.33 (s, 3H), 1.83 – 1.75 (m, 1H), 1.72 – 1.64 (m, 1H), 1.32 – 1.12 (m, 18H), 0.87 (t, J = 7.0 Hz, 3H) ppm.

^{13}C NMR (151 MHz, CDCl$_3$): δ 168.1, 157.6, 141.1, 139.9, 136.5, 130.6, 129.5, 127.3, 124.9, 41.8, 38.6, 35.9, 32.1, 29.8, 29.8, 29.7, 29.6, 29.5, 29.5, 27.3, 22.8, 21.2, 14.3 ppm.

HRMS (ESI-TOF): calc'd for $C_{29}H_{33}Cl_4NNaO_4$ $[M+Na]^+$: 622.1056, found: 622.1054.

TLC: R_f = 0.44 (9.5:0.5 Hexanes:EtOAc).

$[\alpha]_D^{20}$ = –19.0 (c = 0.10, $CHCl_3$).

Compound B4

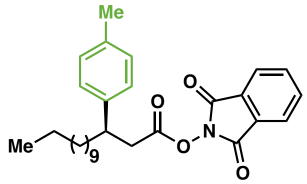

1,3-dioxoisoindolin-2-yl (*R*)-3-(*p*-tolyl)tetradecanoate (B4)

Following General Procedure **B** on 0.2 mmol scale with **A2**. Purification by flash column chromatography (Silica, 19:1 Hexanes:EtOAc) afforded 91 mg (98%) of the title compound **B4**.

Physical State: amorphous white solid.

^{1}H NMR (600 MHz, $CDCl_3$): δ 7.87 (dd, J = 5.4, 3.1 Hz, 2H), 7.77 (dd, J = 5.4, 3.1 Hz, 2H), 7.16 – 7.10 (m, 4H), 3.20 – 3.11 (m, 1H), 2.91 (qd, J = 15.5, 7.4 Hz, 2H), 2.33 (s, 3H), 1.84 – 1.76 (m, 1H), 1.73 – 1.64 (m, 1H), 1.32 – 1.12 (m, 18H), 0.87 (t, J = 7.0 Hz, 3H) ppm.

^{13}C NMR (151 MHz, $CDCl_3$): δ 168.6, 162.0, 140.1, 136.4, 134.8, 129.4, 129.1, 127.4, 124.1, 41.8, 38.7, 36.0, 32.1, 29.8, 29.8, 29.7, 29.6, 29.5, 27.4, 22.8, 21.2, 14.3 ppm.

Note: 1 carbon signal was not assigned due to overlaps.

HRMS (ESI-TOF): calc'd for $C_{29}H_{38}NO_4$ $[M+H]^+$: 464.2795, found: 464.2797.

TLC: R_f = 0.39 (4:1 Hexanes:EtOAc).

$[\alpha]_D^{20}$ = -18.8 (c = 0.25, $CHCl_3$).

Compound B5

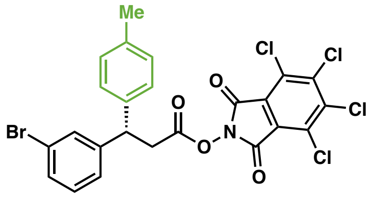

4,5,6,7-tetrachloro-1,3-dioxoisoindolin-2-yl (*S*)-3-(3-bromophenyl)-3-(*p*-tolyl)propanoate (B5)

Following General Procedure **B** on 0.65 mmol scale with **A3**. Purification by recrystallization from CH_2Cl_2:MeOH afforded 296.8 mg (76%) of the title compound **B5**.

Physical State: pale yellow solid

m.p.: 102 – 110 ºC

^{1}H NMR (600 MHz, $CDCl_3$): δ 7.41 – 7.37 (m, 1H), 7.36 (dt, *J* = 6.6, 2.1 Hz, 1H), 7.23 – 7.18 (m, 2H), 7.17 – 7.11 (m, 4H), 4.55 (t, *J* = 7.8 Hz, 1H), 3.42 – 3.32 (m, 2H), 2.32 (s, 3H) ppm.

^{13}C NMR (151 MHz, $CDCl_3$): δ 167.4, 157.4, 144.9, 141.1, 138.5, 137.1, 130.9, 130.6, 130.5, 130.3, 129.8, 127.5, 126.3, 124.8, 123.0, 46.0, 37.3, 21.2 ppm.

HRMS (ESI-TOF): calc'd for $C_{24}H_{14}BrCl_4NNaO_4$ $[M+Na]^+$: 621.8753, found: 621.8749

TLC: R_f = 0.34 (9:1 Hexanes:EtOAc)

$[\alpha]_D^{20}$ = -4.0 (*c* = 0.1, $CHCl_3$)

Compound B6

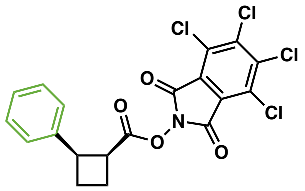

4,5,6,7-tetrachloro-1,3-dioxoisoindolin-2-yl (1S,2R)-2-phenylcyclobutane-1-carboxylate (B6)
Following General Procedure **B** on 0.5 mmol scale with **A4**. Purification by flash column chromatography (silica, 15:1 Hexanes:EtOAc) afforded 343 mg (75%) of the title compound **B6**.

Physical State: amorphous solid
^{1}H NMR (600 MHz, $CDCl_3$): δ 7.38 (dd, J = 8.3, 7.0 Hz, 2H), 7.30 – 7.26 (m, 3H), 4.16 (q, J = 8.8 Hz, 1H), 3.94 – 3.88 (m, 1H), 2.79 – 2.69 (m, 1H), 2.54 – 2.41 (m, 3H) ppm.
^{13}C NMR (151 MHz, $CDCl_3$): δ 169.5, 140.9, 139.6, 130.9, 130.4, 128.7, 127.1, 127.1, 124.8, 42.5, 41.8, 25.1, 22.0 ppm.
HRMS (ESI-TOF): calc'd for $C_{19}H_{12}Cl_4NO_4$ $[M+H]^+$: 457.9520, found: 457.9516.
TLC: R_f = 0.3 (10:1 Hexanes:EtOAc)
$[\alpha]_D^{20}$ = -78.1 (c = 1.0, $CHCl_3$)

Compound B7

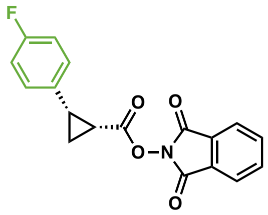

1,3-dioxoisoindolin-2-yl (1S,2S)-2-(4-fluorophenyl)cyclopropane-1-carboxylate (B7)

Following General Procedure **B** on 0.3 mmol scale with **A5**. Purification by flash column chromatography (silica, 15:1 Hexanes:EtOAc) afforded 55 mg (56%) of the title compound **B7**.

Physical State: yellow oil.

^{1}H NMR (400 MHz, CDCl$_3$): δ 7.81 (dd, *J* = 5.5, 3.1 Hz, 2H), 7.73 (dd, *J* = 5.5, 3.1 Hz, 2H), 7.32 (dd, *J* = 8.5, 5.4 Hz, 2H), 6.99 (t, *J* = 8.7 Hz, 2H), 2.88 (q, *J* = 8.4 Hz, 1H), 2.43 (td, *J* = 8.4, 5.5 Hz, 1H), 1.77 (dt, *J* = 7.8, 5.4 Hz, 1H), 1.67 (td, *J* = 8.3, 5.2 Hz, 1H) ppm.

^{19}F NMR (400 MHz, CDCl$_3$): δ -115.31 ppm.

^{13}C NMR (151 MHz, CDCl$_3$): δ 167.6, 162.2 (d, *J* = 245.8 Hz), 162.0, 134.8, 131.4 (d, *J* = 8.2 Hz), 130.5 (d, *J* = 3.3 Hz), 129.0, 124.0, 115.3 (d, *J* = 21.5 Hz), 27.4, 18.1, 14.2 ppm.

HRMS (ESI-TOF): calc'd for $C_{18}H_{13}FNO_4$ $[M+H]^+$: 326.0829, found: 326.0825.

TLC: R_f = 0.3 (10:1 Hexanes:EtOAc)

$[\alpha]_D^{20}$ = -62.7 (*c* = 0.6, $CHCl_3$)

Compound 14

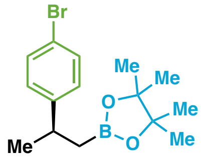

(*R*)-2-(2-(4-bromophenyl)propyl)-4,4,5,5-tetramethyl-1,3,2-dioxaborolane (14)

Following General Procedure **C7** on 0.037 mmol scale with **B2**. Purification by flash column chromatography (Silica, 25:1 Hexanes:EtOAc) afforded 5.6 mg (46%) of the title compound **14**.

Physical State: white amorphous solid.

^{1}H NMR (600 MHz, $CDCl_3$): δ 7.39 – 7.35 (m, 2H), 7.12 – 7.09 (m, 2H), 2.99 (h, J = 7.2 Hz, 1H), 1.24 (d, J = 6.9 Hz, 3H), 1.16 (d, J = 3.0 Hz, 12H), 1.13 – 1.10 (m, 2H) ppm.

^{13}C NMR (151 MHz, $CDCl_3$): δ 148.4, 131.3, 128.6, 119.4, 83.2, 35.5, 24.9, 24.9 ppm.

GC/MS (EI): m/z (%) 324/326 (12%), 309/311 (20%), 183/185 (100%), 104 (84%).

TLC: R_f = 0.38 (9.5:0.5 Hexanes:EtOAc).

$[\alpha]_D^{20}$ = -17.0 (c = 0.1, $CHCl_3$).

Compound 15

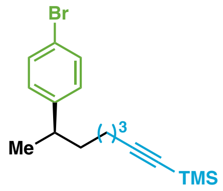

(*R*)-(7-(4-bromophenyl)oct-1-yn-1-yl)trimethylsilane (15)

Following General Procedure **C2** on 0.05 mmol scale with **B2**, $NiCl_2$•glyme (30 mol%), Bipy (60 mol%), and zinc reagent **SI-24**. Purification by pTLC (100% Hexanes) afforded 8.2 mg (48%) of the title compound **15**.

Physical State: clear oil.

^{1}H NMR (600 MHz, $CDCl_3$): δ 7.41 – 7.38 (m, 2H), 7.07 – 7.03 (m, 2H), 2.65 (h, J = 7.0 Hz, 1H), 2.17 (t, J = 7.1 Hz, 2H), 1.57 – 1.41 (m, 4H), 1.35 – 1.21 (m, 2H), 1.21 (d, J = 6.9 Hz, 3H), 0.12 (s, 9H) ppm.

^{13}C NMR (151 MHz, $CDCl_3$): δ 146.7, 131.5, 128.9, 119.5, 107.5, 84.7, 39.5, 37.8, 28.7, 26.9, 22.4, 19.9, 0.3 ppm.

GC/MS (EI): m/z (%) 321/323 (11%), 183 (96%), 104 (94%), 73 (100%).

TLC: R_f = 0.53 (9.5:0.5 Hexanes:EtOAc).

$[\alpha]_D^{20}$ = -18.8 (c = 0.25, $CHCl_3$).

Compound 16

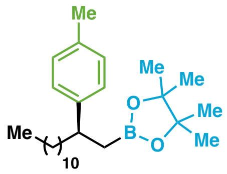

(*S*)-4,4,5,5-tetramethyl-2-(2-(*p*-tolyl)tridecyl)-1,3,2-dioxaborolane (16)

Following General Procedure **C7** on 0.052 mmol scale with **B4**. Purification by flash column chromatography (Silica, 49:1 Hexanes:EtOAc) afforded 17.8 mg (85%) of the title compound **16**.

Physical State: clear oil.

^{1}H NMR (600 MHz, $CDCl_3$): δ 7.09 – 7.04 (m, 4H), 2.77 (qd, J = 8.1, 6.3 Hz, 1H), 2.29 (s, 3H), 1.57 – 1.50 (m, 2H), 1.31 – 1.13 (m, 20H), 1.11 (d, J = 2.4 Hz, 12H), 0.88 (t, J = 7.1 Hz, 3H) ppm.

^{13}C NMR (151 MHz, $CDCl_3$): δ 144.8, 135.1, 128.8, 127.4, 83.0, 41.2, 39.6, 32.1, 29.8, 29.8, 29.8, 29.7, 29.5, 27.8, 24.8, 24.8, 22.8, 21.1, 14.3 ppm.

Note: 1 carbon signal was not assigned due to overlaps.

GC/MS (EI): m/z (%) 400 (2%), 385 (1%), 272 (4%), 245 (100%).

TLC: R_f = 0.45 (9.5:0.5 Hexanes:EtOAc).

$[\alpha]_D^{20}$ = -4.4 (c = 0.25, $CHCl_3$).

Compound 17

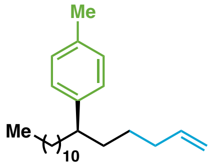

(*R*)-1-(heptadec-1-en-6-yl)-4-methylbenzene (17)

Following General Procedure **C2** on 0.05 mmol scale with **B3**, $NiCl_2$•glyme (30 mol%), Bipy (60 mol%), and zinc reagent **SI-25**. Purification by pTLC (100% Hexanes) afforded 11 mg (72%) of the title compound **17**.

Physical State: clear oil.

^{1}H NMR (500 MHz, $CDCl_3$): δ 7.09 (d, J = 7.8 Hz, 2H), 7.04 – 6.99 (m, 2H), 5.80 – 5.68 (m, 1H), 4.99 – 4.86 (m, 2H), 2.49 – 2.39 (m, 1H), 2.32 (s, 3H), 2.07 – 1.91 (m, 2H), 1.65 – 1.56 (m, 2H), 1.54 – 1.46 (m, 2H), 1.33 – 1.04 (m, 20H), 0.88 (t, J = 7.0 Hz, 3H) ppm.

^{13}C NMR (126 MHz, $CDCl_3$): δ 143.3, 139.2, 135.2, 129.0, 127.6, 114.4, 45.6, 37.1, 36.6, 34.0, 32.1, 29.9, 29.8, 29.8, 29.7, 29.5, 27.8, 27.1, 22.8, 21.2, 14.3. ppm

Note: 1 carbon signal was not assigned due to overlaps.

GC/MS (EI): m/z (%) 328 (2%), 173 (44%), 132 (19%), 105 (100%).

TLC: R_f = 0.75 (100% Hexanes).

$[\alpha]_D^{20}$ = +3.2 (c = 0.25, $CHCl_3$).

Compound 18

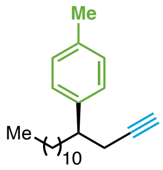

(*R*)-1-methyl-4-(pentadec-1-yn-4-yl)benzene (18)

Following General Procedure **C5** on 0.049 mmol scale with **B3**, $NiCl_2$•$6H_2O$ (20 mol%), diOMeBipy (20 mol%), and zinc reagent **SI-37**. Purification by pTLC (49:1 Hexanes:EtOAc) afforded 7.7 mg (53%) of the title compound **18**.

Physical State: colorless oil

^{1}H NMR (500 MHz, $CDCl_3$): δ 7.10 (q, J = 8.1 Hz, 4H), 2.73 (dtd, J = 9.5, 6.9, 5.3 Hz, 1H), 2.43 (dd, J = 7.0, 2.6 Hz, 2H), 2.32 (s, 3H), 1.93 (t, J = 2.6 Hz, 1H), 1.85 – 1.77 (m, 1H), 1.65 – 1.57 (m, 1H), 1.32 – 1.15 (m, 18H), 0.88 (t, J = 7.0 Hz, 3H) ppm.

^{13}C NMR (151 MHz, $CDCl_3$): δ 141.4, 136.0, 129.2, 127.5, 83.4, 69.5, 44.4, 35.2, 32.1, 29.8, 29.8, 29.8, 29.6, 29.5, 27.6, 26.5, 22.8, 21.2, 14.3 ppm.

Note: One carbon missing due to overlap.

GC/MS (EI): m/z (%) 259 (47%), 143 (3%), 128 (11%), 105 (100%)

TLC: R_f = 0.66 (9.5:0.5 Hexanes:EtOAc)

$[\alpha]_D^{20}$ = +6.0 (c = 0.25, $CHCl_3$)

Compound 19

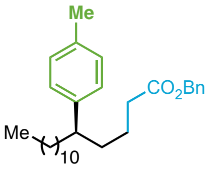

benzyl (*R*)-5-(*p*-tolyl)hexadecanoate (19)

Following General Procedure **C8** on 0.054 mmol scale with **B4** and benzyl acrylate. Purification by pTLC (20:1 Hexanes:EtOAc) afforded 12.3 mg (52%) of the title compound **19**.

Physical State: amorphous white solid

^{1}H NMR (600 MHz, CDCl$_3$): δ 7.39 – 7.29 (m, 5H), 7.08 (d, *J* = 7.8 Hz, 2H), 7.00 (d, *J* = 8.0 Hz, 2H), 5.08 (s, 2H), 2.47 – 2.40 (m, 1H), 2.32 (s, 3H), 2.31 – 2.27 (m, 2H), 1.66 – 1.42 (m, 4H), 1.32 – 1.04 (m, 20H), 0.88 (t, *J* = 7.0 Hz, 3H) ppm.

^{13}C NMR (151 MHz, CDCl$_3$): δ 173.6, 142.7, 136.2, 135.4, 129.1, 128.7, 128.3, 128.3, 127.6, 66.2, 45.5, 37.1, 36.4, 34.5, 32.1, 29.9, 29.8, 29.8, 29.7, 29.5, 27.7, 23.2, 22.8, 21.2, 14.3 ppm.

Note: 1 carbon signal was not assigned due to overlaps.

HRMS (ESI-TOF): calc'd for $C_{30}H_{44}NaO_2$ [M+Na]$^+$: 459.3234, found: 459.3238

TLC: R_f = 0.34 (9.5:0.5 hexanes:EtOAc)

$[\alpha]_D^{20}$ = +1.2 (*c* = 0.25, CHCl$_3$)

Compound 20

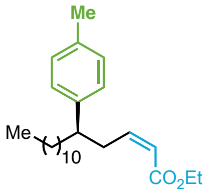

ethyl (*R*,*Z*)-5-(*p*-tolyl)hexadec-2-enoate (20)

Following General Procedure **C3** on 0.047 mmol scale with **B3** and zinc reagent **SI-32**. Purification by pTLC (9:1 Hexanes:EtOAc) afforded 10 mg (58%) of the title compound **20**.

Physical State: colorless oil

^{1}H NMR (500 MHz, $CDCl_3$): δ 7.09 (d, J = 7.8 Hz, 2H), 7.05 (d, J = 8.0 Hz, 2H), 6.05 (dt, J = 11.6, 7.3 Hz, 1H), 5.68 (dt, J = 11.6, 1.8 Hz, 1H), 4.15 (q, J = 7.1 Hz, 2H), 3.10 (dddd, J = 15.4, 7.6, 5.8, 1.8 Hz, 1H), 2.81 (dddd, J = 15.8, 9.1, 6.9, 1.8 Hz, 1H), 2.62 (tt, J = 9.2, 5.6 Hz, 1H), 2.31 (s, 3H), 1.33 – 1.09 (m, 20H), 0.89 – 0.86 (m, 6H) ppm.

^{13}C NMR (151 MHz, $CDCl_3$): δ 166.6, 149.1, 141.9, 135.7, 129.1, 127.7, 120.3, 59.9, 45.6, 36.9, 35.9, 32.1, 29.9, 29.8, 29.8, 29.8, 29.7, 29.5, 27.6, 22.8, 21.2, 14.4, 14.3 ppm.

HRMS (ESI-TOF): calc'd for $C_{25}H_{41}O_2$ $[M+H]^+$: 373.3101, found: 373.3097

TLC: R_f = 0.37 (9.5:0.5 Hexanes:EtOAc)

$[\alpha]_D^{20}$ = +4.5 (c = 0.20, $CHCl_3$)

Compound 21

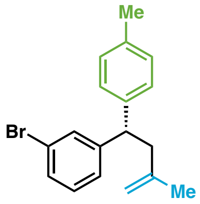

(*S*)-1-bromo-3-(3-methyl-1-(*p*-tolyl)but-3-en-1-yl)benzene (21)

Following General Procedure **C3** on 0.05 mmol scale with **B5** and zinc reagent **SI-31**. Purification by pTLC (99:1 Hexanes:EtOAc) afforded 6.2 mg (39%) of the title compound **21**.

Physical State: colorless oil

^{1}H NMR (500 MHz, $CDCl_3$): δ 7.37 (t, *J* = 1.8 Hz, 1H), 7.28 (dt, *J* = 7.7, 1.7 Hz, 1H), 7.17 – 7.11 (m, 2H), 7.12 – 7.07 (m, 4H), 4.73 – 4.68 (m, 1H), 4.62 – 4.58 (m, 1H), 4.10 (t, *J* = 7.9 Hz, 1H), 2.78 – 2.68 (m, 2H), 2.30 (s, 3H), 1.68 (d, *J* = 1.2 Hz, 3H) ppm.

^{13}C NMR (151 MHz, $CDCl_3$): δ 147.5, 143.1, 141.0, 136.1, 131.1, 130.0, 129.3, 129.3, 127.8, 126.7, 122.6, 113.0, 48.7, 43.9, 22.7, 21.1 ppm.

GC/MS (EI): m/z (%) 314/316 (1%), 259/261 (100%), 180 (43%), 165 (94%).

TLC: R_f = 0.74 (9.5:0.5 Hexanes:EtOAc)

$[\alpha]_D^{20}$ = -4.5 (*c* = 0.20, $CHCl_3$.

Compound 22

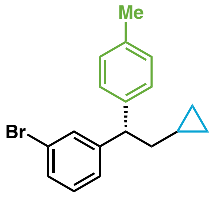

(*S*)-1-bromo-3-(2-cyclopropyl-1-(*p*-tolyl)ethyl)benzene (22)

Following General Procedure **C2** on 0.05 mmol scale with **B5,** $NiCl_2$•glyme (30 mol%), Bipy (60 mol%), and zinc reagent **SI-27**. by pTLC (49:1 Hexanes:EtOAc) afforded 8.1 mg (51%) of the title compound **22**.

Physical State: colorless oil

^{1}H NMR (500 MHz, $CDCl_3$): δ 7.39 (t, *J* = 1.9 Hz, 1H), 7.29 (ddd, *J* = 7.7, 2.0, 1.2 Hz, 1H), 7.17 (dt, *J* = 7.8, 1.5 Hz, 1H), 7.14 – 7.09 (m, 5H), 3.97 (t, *J* = 7.7 Hz, 1H), 2.31 (s, 3H), 1.96 – 1.83 (m, 2H), 0.62 – 0.53 (m, 1H), 0.43 – 0.35 (m, 2H), 0.09 – 0.01 (m, 2H) ppm.

^{13}C NMR (126 MHz, $CDCl_3$: δ 148.1, 141.5, 136.0, 131.1, 130.0, 129.3, 129.2, 127.9, 126.7, 122.6, 51.2, 41.0, 21.1, 9.8, 4.9, 4.8 ppm.

GC/MS (EI): m/z (%) 314/316 (5%), 259/261 (78%), 178/180 (39%), 165 (100%).

TLC: 0.76 (9.5:0.5 Hexanes:EtOAc)

$[\alpha]_D^{20}$ = -19.6 (*c* = 0.25, $CHCl_3$)

Compound 23

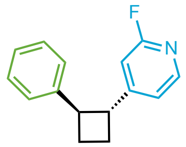

2-fluoro-4-((1*R*,2*R*)-2-phenylcyclobutyl)pyridine (23)

Following General Procedure **C1** on 0.1 mmol scale with **B6**, $NiCl_2$•$6H_2O$ (30 mol%), BPhen (30 mol%), and boronic acid **SI-17** (3.0 equiv.). Purification by pTLC (silica, 8:1 Hexanes:EtOAc) afforded 10.2 mg (45%) of the title compound **23**.

Physical State: colorless oil

^{1}H NMR (600 MHz, $CDCl_3$): δ 8.08 – 8.03 (m, 1H), 7.64 (td, *J* = 8.0, 2.6 Hz, 1H), 7.33 – 7.28 (m, 2H), 7.23 – 7.19 (m, 3H), 6.86 (dd, *J* = 8.4, 3.0 Hz, 1H), 3.59 – 3.49 (m, 2H), 2.38 – 2.34 (m, 2H), 2.24 – 2.22 (m, 1H), 2.13 – 2.10 (m, 1H) ppm.

^{19}F NMR (400 MHz, $CDCl_3$): δ -71.93 ppm.

^{13}C NMR (151 MHz, $CDCl_3$): δ 162.6 (d, J_{C-F} = 237.2 Hz), 145.9 (d, J_{C-F} = 14.3 Hz), 143.6, 139.4 (d, J_{C-F} = 7.9 Hz), 137.4 (d, J_{C-F} = 4.5 Hz), 128.7, 126.7, 126.7, 109.2 (d, J_{C-F} = 37.4 Hz), 48.4, 44.9, 26.2, 25.8 ppm.

TLC: 0.5 (10:1 Hexanes:EtOAc).

HRMS (ESI-TOF): calc'd for $C_{15}H_{15}FN$ $[M+H]^+$ 228.1189; found 228.1190.

$[\alpha]_D^{20}$ = 101.1 (*c* = 0.33, $CHCl_3$).

Compound 24

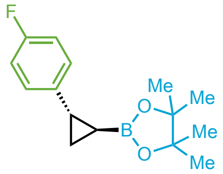

2-((1S,2S)-2-(4-fluorophenyl)cyclopropyl)-4,4,5,5-tetramethyl-1,3,2-dioxaborolane (24)

Following General Procedure **A1.5** on 0.1 mmol scale with **B7**. Purification by pTLC (silica, 25:1 Hexanes:Et_2O) afforded 8.6 mg (33%) of the title compound **24**.

Physical State: colorless oil

^{1}H NMR (600 MHz, $CDCl_3$): δ 7.03 (dd, J = 8.6, 5.5 Hz, 2H), 6.92 (t, J = 8.7 Hz, 2H), 2.08 (dt, J = 8.1, 5.4 Hz, 1H), 1.25 (d, J = 5.7 Hz, 12H), 1.17 – 1.12 (m, 1H), 0.94 (ddd, J = 9.4, 5.3, 3.7 Hz, 1H), 0.23 (ddd, J = 9.8, 6.8, 5.5 Hz, 1H) ppm.

^{19}F NMR (400 MHz, $CDCl_3$): δ -118.30 ppm.

^{13}C NMR (151 MHz, $CDCl_3$): δ 161.3 (d, $J_{C\text{-}F}$ = 243.0 Hz), 139.0 (d, $J_{C\text{-}F}$ = 3.1 Hz), 127.3 (d, $J_{C\text{-}F}$ = 7.7 Hz), 115.1 (d, $J_{C\text{-}F}$ = 21.4 Hz), 83.4, 24.9, 24.9, 21.34, 14.92 ppm.

TLC: 0.3 (20:1 Hexanes:Et_2O).

HRMS (ESI-TOF): calc'd for $C_{15}H_{21}BFO_2$ $[M+H]^+$ 263.1619; found 263.1617.

$[\alpha]_D^{20}$ = 36.3 (c = 0.2, $CHCl_3$).

Experimental Procedures and Characterization Data for Azetidine Scaffolds (Figure 3–a)

Compound B8:

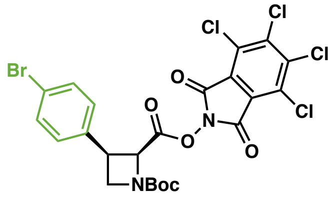

1-(tert-butyl) 2-(4,5,6,7-tetrachloro-1,3-dioxoisoindolin-2-yl) (2*S*,3*R*)-3-(4-bromophenyl)azetidine-1,2-dicarboxylate (B8)

Following General Procedure **B** on 1.12 mmol scale with **A6**. Purified by flash column chromatography (silica, 6:1 Hexanes:EtOAc) afforded 520 mg (73%) of the title compound **B8**.

Physical State: yellow oil.

^{1}H NMR (600 MHz, CDCl$_3$): δ 7.51 (d, J = 8.4 Hz, 2H), 7.25 (d, J = 8.3 Hz, 2H), 5.32 (d, J = 9.2 Hz, 1H), 4.31 (q, J = 8.6 Hz, 2H), 4.23 (td, J = 8.9, 6.4 Hz, 1H), 1.49 (s, 9H) ppm.

^{13}C NMR (151 MHz, CDCl$_3$): δ 164.9, 156.4, 155.1, 141.0, 134.1, 132.3, 130.5, 130.0, 124.6, 122.7, 81.7, 64.3, 52.9, 37.5, 28.3 ppm.

HRMS (ESI-TOF): calc'd for $C_{23}H_{18}BrCl_4N_2O_6$ [M+H]$^+$: 636.9102; found 636.9101.

TLC: R_f = 0.3 (4:1 Hexanes:EtOAc).

$[\alpha]_D^{20}$ = 38.9 (c = 1.0, $CHCl_3$).

Chiral HPLC: Chiralpak IA, 4.6 x 250 mm; 45/55 *i*PrOH/hexanes, 1 mL/min, 254 nm; t_R (minor) = 11.4 min, t_R (major) = 15.0 min, >99% *ee*.

Racemic:

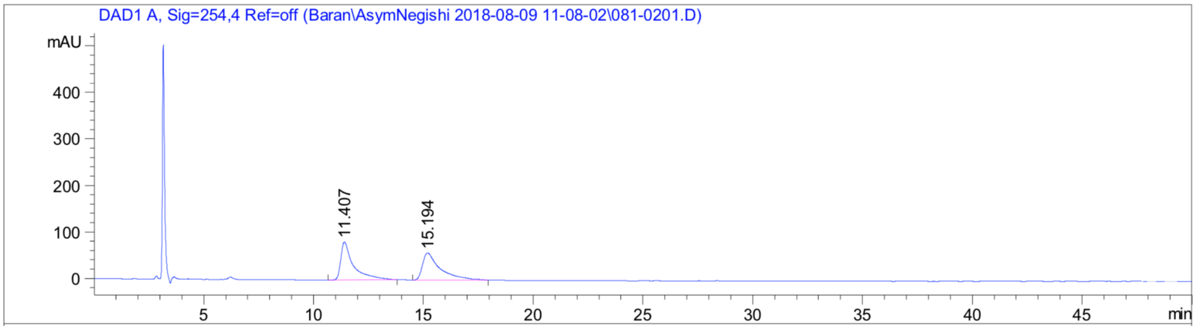

Enantioenriched **B8**:

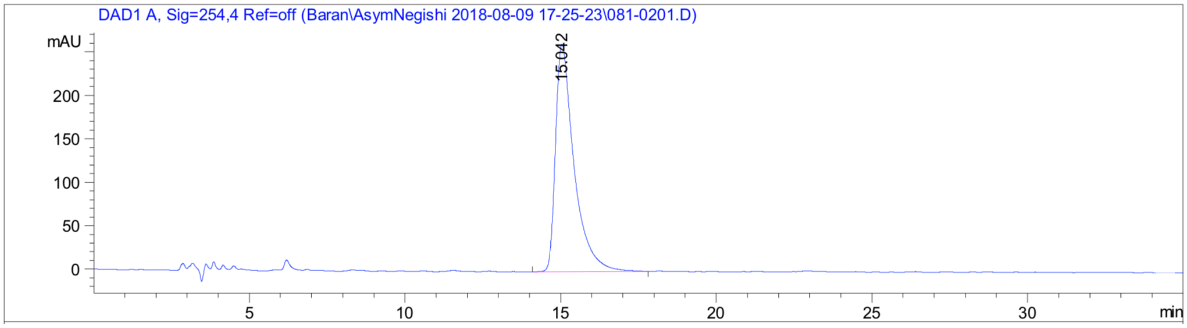

Signal 1: DAD1 A, Sig=254,4 Ref=off

Peak #	RetTime [min]	Type	Width [min]	Area [mAU*s]	Height [mAU]	Area %
1	11.407	BB	0.5591	3271.03638	81.69596	49.9908
2	15.194	BB	0.7803	3272.24634	58.16977	50.0092

Signal 1: DAD1 A, Sig=254,4 Ref=off

Peak #	RetTime [min]	Type	Width [min]	Area [mAU*s]	Height [mAU]	Area %
1	15.042	BB	0.6146	1.08628e4	261.50439	100.0000

Compound 26

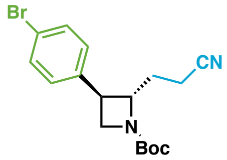

***tert*-butyl (2*S*,3*R*)-3-(4-bromophenyl)-2-(2-cyanoethyl)azetidine-1-carboxylate (26)**

Following General Procedure **C8** on 0.1 mmol scale with **B8** and acrylonitrile. Purification by pTLC (silica, 4:1 Hexanes:EtOAc) afforded 12.7 mg (35%) of the title compound **26**.

Physical State: colorless oil

^{1}H NMR (600 MHz, $CDCl_3$, both rotamers) δ 7.48 (d, J = 8.4 Hz, 2H), 7.14 (d, J = 8.4 Hz, 2H), 4.23 (q, J = 6.1 Hz, 1H), 4.18 (t, J = 8.7 Hz, 1H), 3.89 – 3.86 (m, 1H), 3.36 – 3.32 (m, 1H), 2.55 – 2.47 (m, 2H), 2.27 – 2.19 (m, 1H), 2.16 – 2.08 (m, 1H), 1.48 (s, 9H) ppm.

^{13}C NMR (151 MHz, $CDCl_3$, both rotamers) δ 157.1, 139.4, 132.2, 128.7, 121.4, 119.6, 80.7, 68.4, 54.0, 39.9, 31.7, 28.5, 13.9 ppm.

HRMS (ESI-TOF): calc'd for $C_{17}H_{22}BrN_2O_2$ $[M+H]^+$: 365.0865; found 365.0862.

TLC: R_f = 0.3 (3:1 Hexanes:EtOAc).

$[\alpha]_D^{20}$ = 2.2 (c = 0.1, $CHCl_3$).

Chiral HPLC: Chiralpak IC, 4.6 x 250 mm; 10/90 *i*PrOH/hexanes, 1 mL/min, 210 nm; t_R (minor) = 25.9 min, t_R (major) = 23.8 min, >99% *ee*.

Racemic:

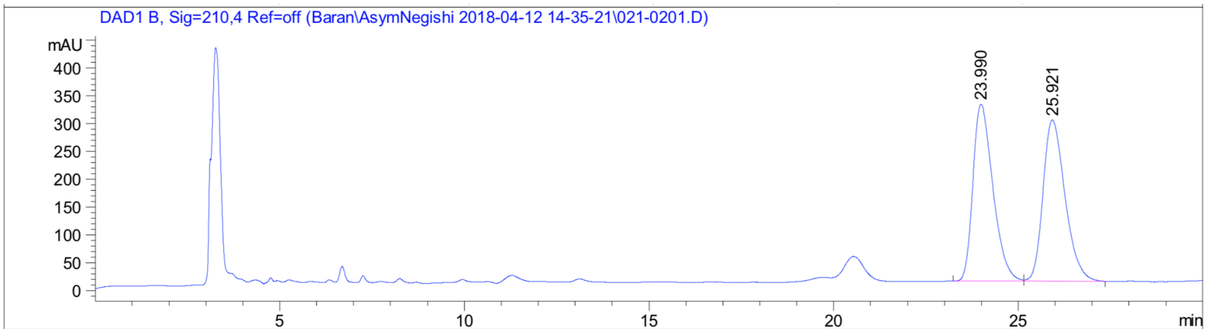

Enantioenriched **26**:

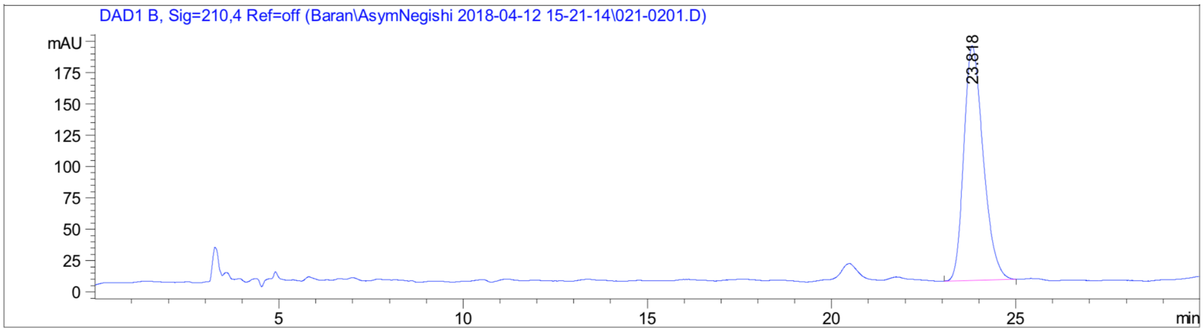

Signal 2: DAD1 B, Sig=210,4 Ref=off

Peak #	RetTime [min]	Type	Width [min]	Area [mAU*s]	Height [mAU]	Area %
1	23.990	BV	0.5731	1.19703e4	318.01227	49.8782
2	25.921	VB	0.6357	1.20287e4	290.14304	50.1218

Signal 2: DAD1 B, Sig=210,4 Ref=off

Peak #	RetTime [min]	Type	Width [min]	Area [mAU*s]	Height [mAU]	Area %
1	23.818	BB	0.5674	6852.26123	187.06097	100.0000

Compound 27

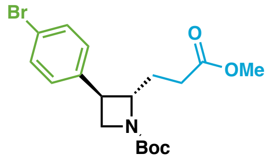

***tert*-butyl (2*S*,3*R*)-3-(4-bromophenyl)-2-(3-methoxy-3-oxopropyl)azetidine-1-carboxylate (27)**

Following General Procedure **C8** on 0.1 mmol scale with **B8** and methyl acrylate. Purification by pTLC (silica, 4:1 Hexanes:EtOAc) afforded 20.2 mg (50%) of the title compound **27**.

Physical State: colorless oil

^{1}H NMR (600 MHz, $CDCl_3$, both rotamers δ 7.46 (d, *J* = 8.4 Hz, 2H), 7.13 (d, *J* = 8.4 Hz, 2H), 4.17 (dd, *J* = 10.0, 7.4 Hz, 2H), 3.83 (t, *J* = 7.7 Hz, 1H), 3.64 (s, 3H), 3.28 (q, *J* = 6.5 Hz, 1H), 2.42 (d, *J* = 54.1 Hz, 2H), 2.27 – 2.19 (m, 1H), 2.15 – 2.08 (m, 1H), 1.47 (s, 9H) ppm.

^{13}C NMR (151 MHz, $CDCl_3$, both rotamers) δ 173.1, 156.4, 139.7, 131.4, 128.2, 120.4, 79.5, 68.6, 53.2, 51.2, 39.5, 30.1, 29.3, 28.0 ppm.

HRMS (ESI-TOF): calc'd for $C_{18}H_{24}BrNO_4Na$ $[M+Na]^+$: 420.0786; found 420.0785.

TLC: R_f = 0.3 (4:1 Hexanes:EtOAc).

$[\alpha]_D^{20}$ = 2.5 (*c* = 0.25, $CHCl_3$).

Chiral HPLC: Chiralpak IC, 4.6 x 250 mm; 20/80 *i*PrOH/hexanes, 1 mL/min, 210 nm; t_R (minor) = 9.0 min, t_R (major) = 7.9 min, 97.5% *ee*.

Racemic:

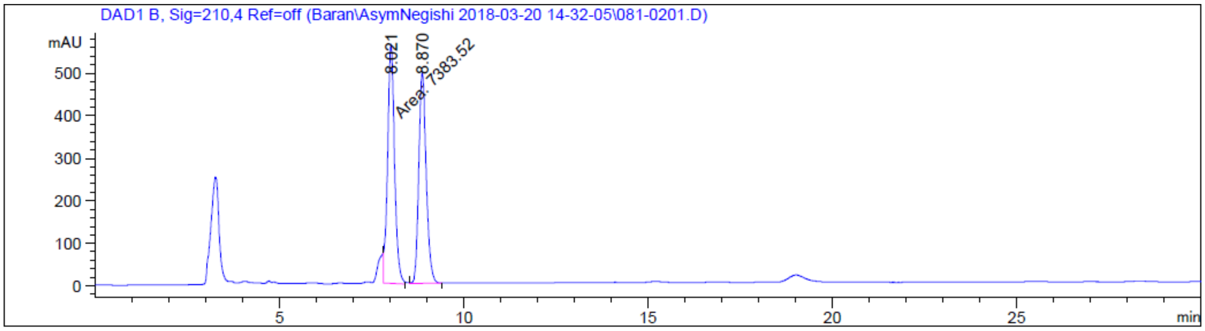

Enantioenriched **27**:

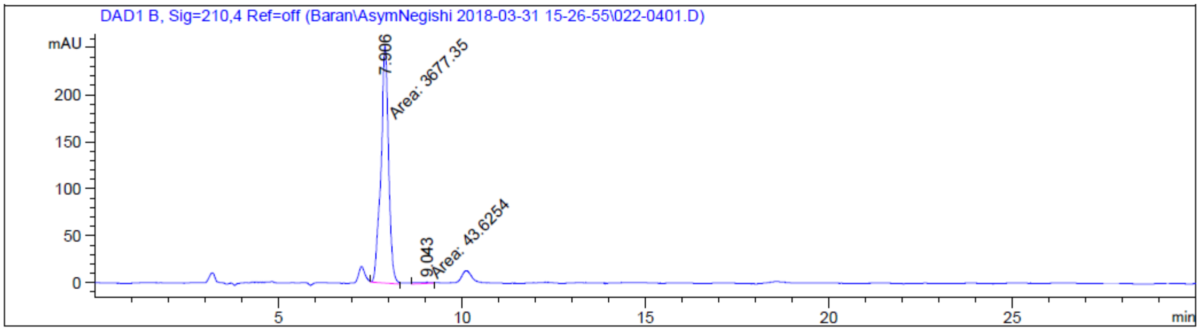

Signal 2: DAD1 B, Sig=210,4 Ref=off

Peak #	RetTime [min]	Type	Width [min]	Area [mAU*s]	Height [mAU]	Area %
1	8.021	MM	0.2194	7383.51953	560.79944	51.5350
2	8.870	VB	0.2157	6943.67236	494.93085	48.4650

Signal 2: DAD1 B, Sig=210,4 Ref=off

Peak #	RetTime [min]	Type	Width [min]	Area [mAU*s]	Height [mAU]	Area %
1	7.906	MM	0.2432	3677.34570	251.98637	98.8276
2	9.043	MM	0.6166	43.62544	1.17916	1.1724

Compound 28

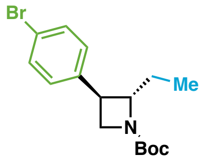

***tert*-butyl (2*S*,3*R*)-3-(4-bromophenyl)-2-ethylazetidine-1-carboxylate (28)**

Following General Procedure **C2** on 0.05 mmol scale with **B8**, $NiCl_2$•glyme (30 mol%), di*t*BuBipy (40 mol%), and Zinc reagent **SI-28** (2.0 equiv.). Purification by pTLC (silica, 6:1 Hexanes:EtOAc) afforded 11.0 mg (65%) of the title compound **28**.

Physical State: colorless oil

^{1}H NMR (600 MHz, $CDCl_3$, both rotamers) δ 7.46 (d, J = 8.5 Hz, 2H), 7.15 (d, J = 8.4 Hz, 2H), 4.17 (td, J = 8.7, 0.6 Hz, 1H), 4.09 (s, 1H), 3.82 (dd, J = 8.7, 6.5 Hz, 1H), 3.25 (dt, J = 8.7, 6.2 Hz, 1H), 2.00 – 1.96 (m, 1H), 1.79 – 1.73 (m, 1H), 1.46 (s, 9H), 0.91 (t, J = 7.5 Hz, 3H) ppm.

^{13}C NMR (151 MHz, $CDCl_3$, both rotamers) δ 156.7, 141.1, 131.9, 128.9, 120.8, 79.7, 71.2, 53.6, 39.9, 28.6, 28.0, 8.8 ppm.

HRMS (ESI-TOF): calc'd for $C_{16}H_{23}BrNO_2$ $[M+H]^+$: 340.0912; found 340.0913.

TLC: R_f = 0.3 (4:1 Hexanes:EtOAc).

$[\alpha]_D^{20}$ = 1.1 (c = 0.1, $CHCl_3$).

Chiral HPLC: Chiralpak IC, 4.6 x 250 mm; 10/80 *i*PrOH/hexanes, 0.5 mL/min, 210 nm; t_R (minor) = 16.4 min, t_R (major) = 10.6 min, >99% *ee*.

Racemic:

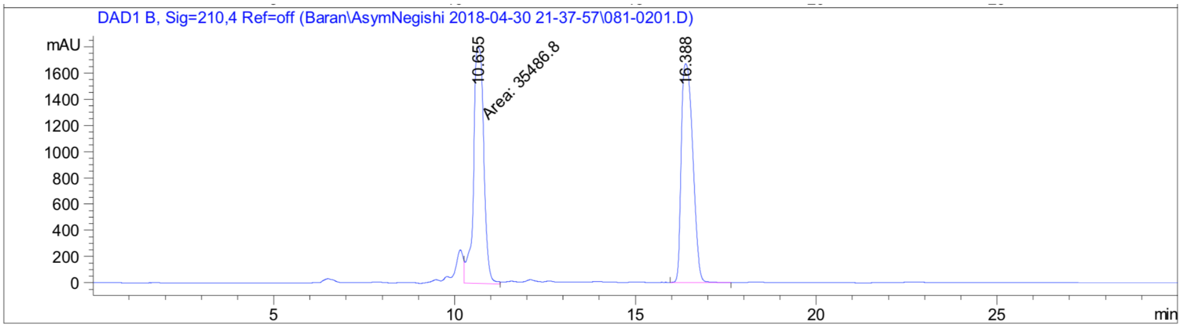

Enantioenriched **28**:

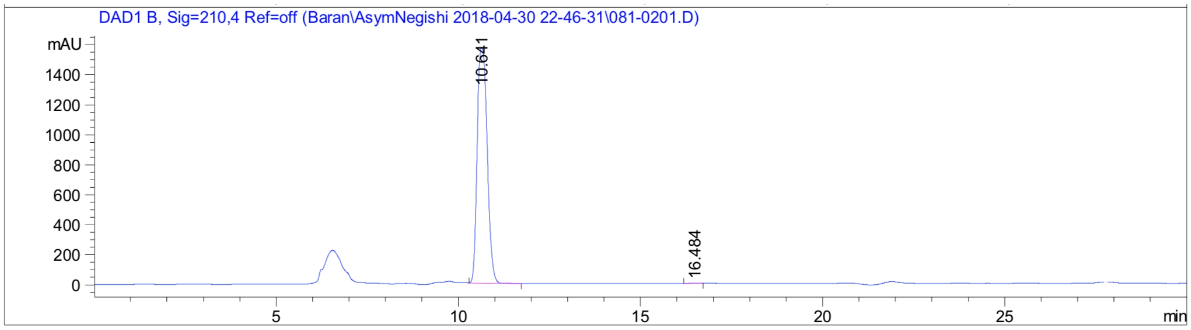

Signal 2: DAD1 B, Sig=210,4 Ref=off

Peak #	RetTime [min]	Type	Width [min]	Area [mAU*s]	Height [mAU]	Area %
1	10.655	MM	0.3288	3.54868e4	1799.06250	49.1405
2	16.388	BB	0.3569	3.67281e4	1667.99353	50.8595

Signal 2: DAD1 B, Sig=210,4 Ref=off

Peak #	RetTime [min]	Type	Width [min]	Area [mAU*s]	Height [mAU]	Area %
1	10.641	BB	0.3042	2.99220e4	1571.05786	99.8656
2	16.484	BB	0.1922	40.26966	2.75977	0.1344

Compound 29

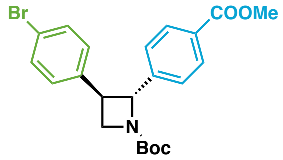

***tert*-butyl (2*R*,3*R*)-3-(4-bromophenyl)-2-(4-(methoxycarbonyl)phenyl)azetidine-1-carboxylate (29)**

Following General Procedure **C1** on 0.05 mmol scale with **B8**, $NiCl_2$•$6H_2O$ (20 mol%), BPhen (25 mol%), and boronic acid **SI-7** (3.0 equiv.). Purification by pTLC (silica, 4:1 Hexanes:EtOAc) afforded 20.1 mg (90%) of the title compound **29**.

Physical State: colorless oil

^{1}H NMR (600 MHz, $CDCl_3$, both rotamers) δ 8.04 (d, J = 8.0 Hz, 2H), 7.50 (d, J = 8.4 Hz, 2H), 7.39 (d, J = 8.0 Hz, 2H), 7.17 (d, J = 8.4 Hz, 2H), 5.12 (s, 1H), 4.35 (t, J = 8.6 Hz, 1H), 4.08 (t, J = 7.6 Hz, 1H), 3.92 (s, 3H), 3.50 (q, J = 7.0 Hz, 1H), 1.35 (s, 9H) ppm.

^{13}C NMR (151 MHz, $CDCl_3$, both rotamers) δ 167.0, 156.7, 146.4, 139.4, 132.2, 130.2, 129.7, 128.9, 125.8, 121.5, 80.5, 72.1, 53.4, 52.3, 44.0, 28.4 ppm.

HRMS (ESI-TOF): calc'd for $C_{22}H_{24}BrNO_4Na$ $[M+Na]^+$: 468.0786; found 468.0781.

TLC: R_f = 0.3 (8:1 Hexanes:EtOAc)

$[\alpha]_D^{20}$ = 25.0 (c = 0.25, $CHCl_3$).

Chiral HPLC: Chiralpak IC, 4.6 x 250 mm; 20/80 *i*PrOH/hexanes, 1 mL/min, 254 nm; t_R (minor) = 13.8 min, t_R (major) = 9.1 min, >99% *ee*.

Racemic:

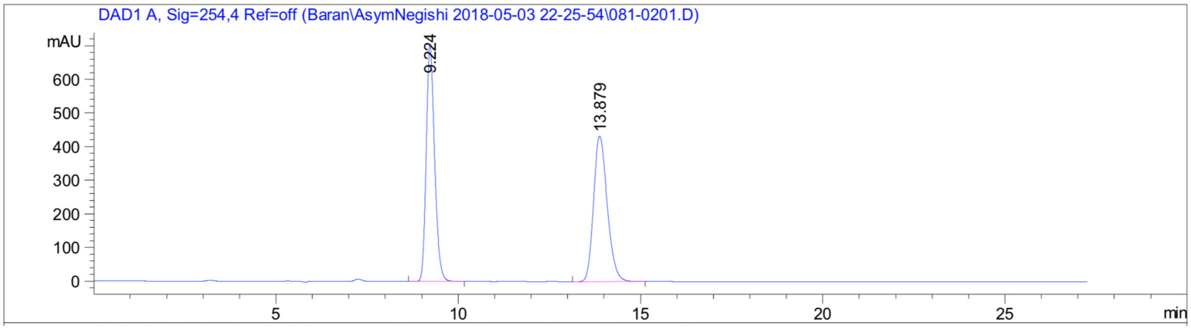

Enantioenriched **29**:

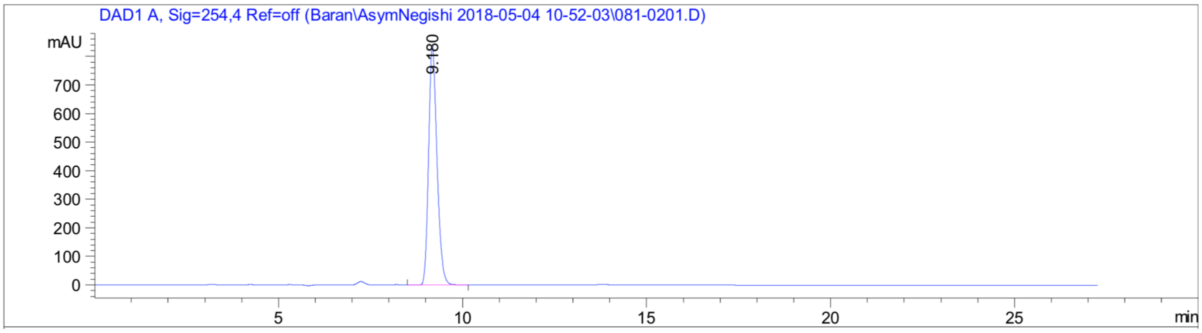

Signal 1: DAD1 A, Sig=254,4 Ref=off

Peak #	RetTime [min]	Type	Width [min]	Area [mAU*s]	Height [mAU]	Area %
1	9.224	BB	0.2467	1.12765e4	704.48834	49.9709
2	13.879	BB	0.4023	1.12897e4	432.83252	50.0291

Signal 1: DAD1 A, Sig=254,4 Ref=off

Peak #	RetTime [min]	Type	Width [min]	Area [mAU*s]	Height [mAU]	Area %
1	9.180	BB	0.2457	1.33629e4	839.36615	100.0000

Compound 30

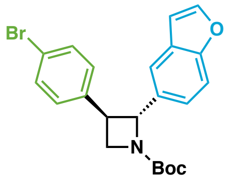

***tert*-butyl (2*R*,3*R*)-2-(benzofuran-5-yl)-3-(4-bromophenyl)azetidine-1-carboxylate (30)**

Following General Procedure **C1** on 0.1 mmol scale with **B8**, $NiCl_2$•$6H_2O$ (20 mol%), BPhen (25 mol%), and boronic acid **SI-12** (3.0 equiv.). Purification by pTLC (silica, 5:1 Hexanes:EtOAc) afforded 38.8 mg (91%) of the title compound **30**.

Physical State: colorless oil

^{1}H NMR (600 MHz, $CDCl_3$, both rotamers) δ 7.63 (s, 1H), 7.58 (s, 1H), 7.49 (dd, J = 8.4, 3.1 Hz, 3H), 7.29 (d, J = 8.2 Hz, 1H), 7.17 (d, J = 8.3 Hz, 2H), 6.75 (s, 1H), 5.16 (s, 1H), 4.39 (t, J = 8.6 Hz, 1H), 4.07 (t, J = 7.8 Hz, 1H), 3.61 – 3.55 (m, 1H), 1.34 (s, 9H) ppm.

^{13}C NMR (151 MHz, $CDCl_3$, both rotamers) δ 156.9, 154.7, 145.6, 139.9, 136.1, 132.0, 128.9, 127.7, 122.4, 121.2, 118.7, 111.6, 106.8, 80.1, 72.7, 53.3, 44.6, 28.4 ppm.

HRMS (ESI-TOF): calc'd for $C_{22}H_{22}BrNO_3Na$ $[M+Na]^+$ 450.0681; found 450.0675.

TLC: R_f = 0.3 (8:1 Hexanes:EtOAc).

$[\alpha]_D^{20}$ = 68.4 (c = 1.0, $CHCl_3$).

Chiral HPLC: Chiralpak IC, 4.6 x 250 mm; 20/80 *i*PrOH/hexanes, 1 mL/min, 254 nm; t_R (minor) = 9.5 min, t_R (major) = 5.3 min, >99% *ee*.

Racemic:

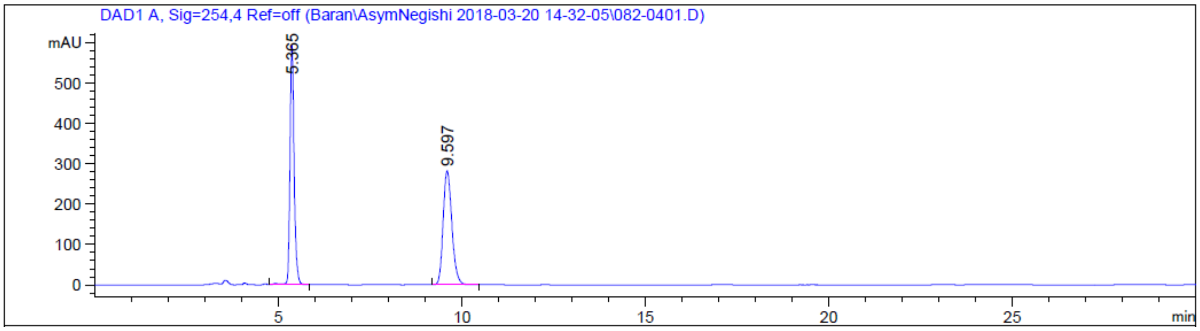

Enantioenriched **30**:

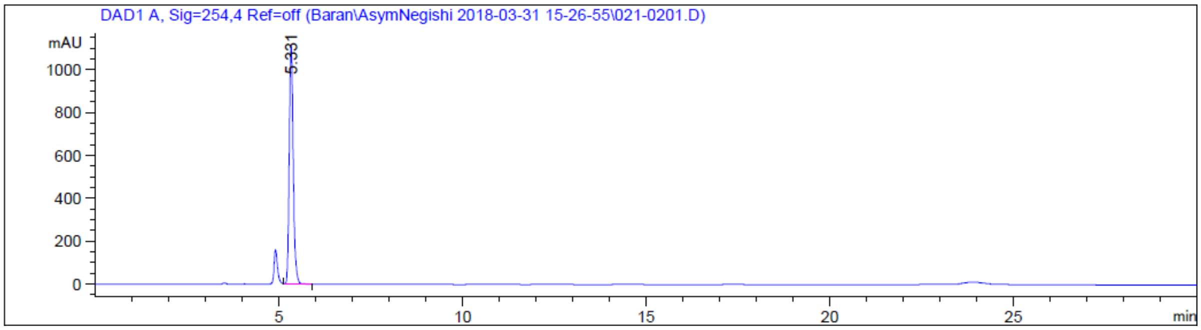

Signal 1: DAD1 A, Sig=254,4 Ref=off

Peak #	RetTime [min]	Type	Width [min]	Area [mAU*s]	Height [mAU]	Area %
1	5.365	VB R	0.1193	4678.40088	593.62848	50.0864
2	9.597	BB	0.2547	4662.25586	282.32898	49.9136

Signal 1: DAD1 A, Sig=254,4 Ref=off

Peak #	RetTime [min]	Type	Width [min]	Area [mAU*s]	Height [mAU]	Area %
1	5.331	VB	0.1162	8411.71875	1112.50671	100.0000

Compound 31

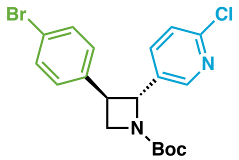

***tert*-butyl (2*R*,3*R*)-3-(4-bromophenyl)-2-(6-chloropyridin-3-yl)azetidine-1-carboxylate (31)**

Following General Procedure **C1** on 0.1 mmol scale with **B8**, $NiCl_2 \cdot 6H_2O$ (20 mol%), BPhen (25 mol%), and boronic acid **SI-13** (3.0 equiv.). Purification by pTLC (silica, 4:1 Hexanes:EtOAc) afforded 24.2 mg (57%) of the title compound **31**.

Physical State: colorless oil

^{1}H NMR (600 MHz, $CDCl_3$, both rotamers) δ 8.34 (d, J = 2.6 Hz, 1H), 7.67 (dd, J = 8.3, 2.5 Hz, 1H), 7.50 (d, J = 8.2 Hz, 2H), 7.35 (d, J = 8.2 Hz, 1H), 7.15 (d, J = 8.1 Hz, 2H), 5.08 (d, J = 6.6 Hz, 1H), 4.32 (t, J = 8.6 Hz, 1H), 4.09 (t, J = 7.9 Hz, 1H), 3.52 (q, J = 7.3 Hz, 1H), 1.38 (s, 9H) ppm.

^{13}C NMR (151 MHz, $CDCl_3$, both rotamers) δ 156.6, 151.0, 147.7, 138.6, 136.5, 135.7, 132.3, 128.8, 124.5, 121.7, 80.9, 69.4, 53.5, 43.7, 28.4 ppm.

HRMS (ESI-TOF): calc'd for $C_{19}H_{21}BrClN_2O_2$ $[M+H]^+$: 423.0475; found 423.0480.

TLC: R_f = 0.4 (4:1 Hexanes:EtOAc)

$[\alpha]_D^{20}$ = 35.1 (c = 1.0, $CHCl_3$).

Chiral HPLC: Chiralpak IC, 4.6 x 250 mm; 20/80 *i*PrOH/hexanes, 1 mL/min, 210 nm; t_R (minor) = 14.1 min, t_R (major) = 9.9 min, >99% *ee*.

Racemic:

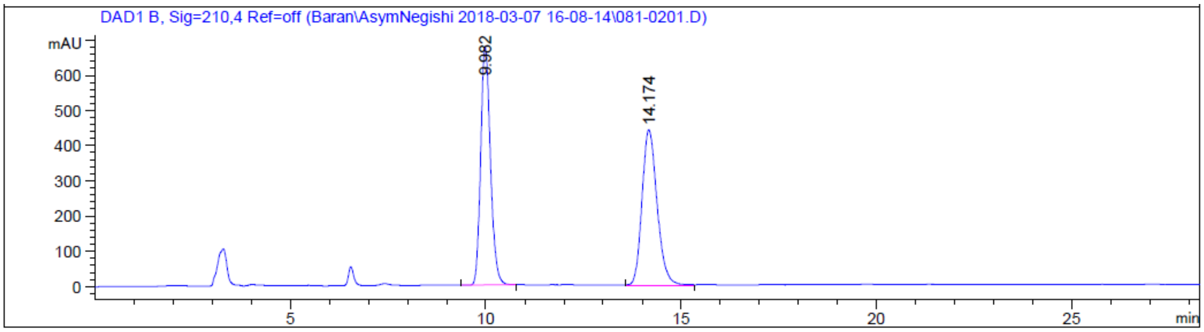

Enantioenriched **31**:

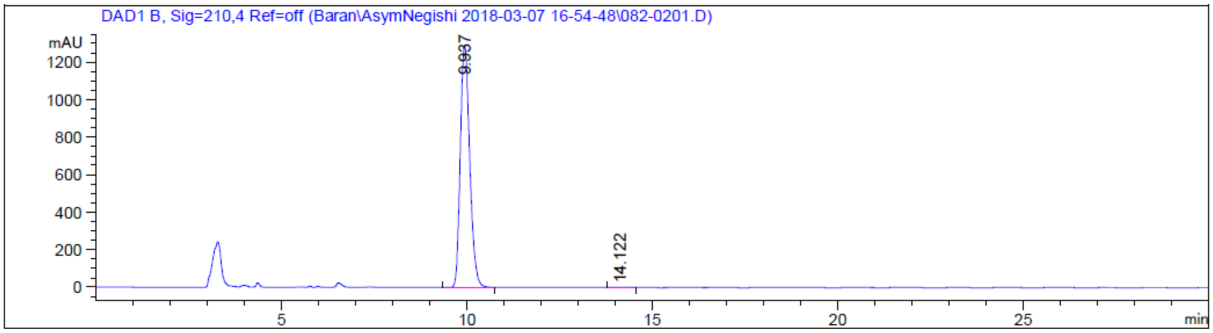

Signal 2: DAD1 B, Sig=210,4 Ref=off

Peak #	RetTime [min]	Type	Width [min]	Area [mAU*s]	Height [mAU]	Area %
1	9.982	BB	0.2690	1.17549e4	675.44183	50.0049
2	14.174	BB	0.4135	1.17525e4	440.20493	49.9951

Signal 2: DAD1 B, Sig=210,4 Ref=off

Peak #	RetTime [min]	Type	Width [min]	Area [mAU*s]	Height [mAU]	Area %
1	9.937	BB	0.2766	2.28266e4	1289.03564	99.8148
2	14.122	BB	0.2848	42.35325	1.83813	0.1852

Compound 32

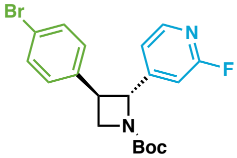

***tert*-butyl (2*R*,3*R*)-3-(4-bromophenyl)-2-(2-fluoropyridin-4-yl)azetidine-1-carboxylate (32)**

Following General Procedure **C1** on 0.1 mmol scale with **B8**, $NiCl_2$•$6H_2O$ (20 mol%), BPhen (25 mol%), and boronic acid **SI-17** (3.0 equiv.). Purification by pTLC (silica, 4:1 Hexanes:EtOAc) afforded 29.6 mg (73%) of the title compound **32**.

Physical State: colorless oil

^{1}H NMR (600 MHz, $CDCl_3$, both rotamers) δ 8.20 (d, J = 5.2 Hz, 1H), 7.52 (d, J = 8.4 Hz, 2H), 7.17 (d, J = 8.4 Hz, 2H), 7.11 – 7.06 (m, 1H), 6.89 (s, 1H), 5.10 (d, J = 6.5 Hz, 1H), 4.31 (t, J = 8.6 Hz, 1H), 4.08 (t, J = 7.8 Hz, 1H), 3.53 – 3.44 (m, 1H), 1.40 (s, 9H) ppm.

^{19}F NMR (400 MHz, $CDCl_3$): δ -67.58 ppm.

^{13}C NMR (151 MHz, $CDCl_3$, both rotamers) δ 164.4 (d, J_{C-F} = 239.2 Hz), 156.7, 156.1 (d, J_{C-F} = 7.5 Hz), 148.2 (d, J_{C-F} = 15.1 Hz), 138.5, 132.3, 128.8, 121.8, 118.2 (d, J_{C-F} = 3.9 Hz),), 106.3 (d, J_{C-F} = 38.1 Hz), 81.0, 70.5, 53.7, 43.5, 28.4 ppm.

HRMS (ESI-TOF): calc'd for $C_{19}H_{21}BrFN_2O_2$ $[M+H]^+$: 407.0770; found 407.0770.

TLC: R_f = 0.2 (8:1 Hexanes:EtOAc).

$[\alpha]_D^{20}$ = 29.7 (c = 1.0, $CHCl_3$).

Chiral HPLC: Chiralpak IC, 4.6 x 250 mm; 20/80 *i*PrOH/hexanes, 1 mL/min, 210 nm; t_R (minor) = 19.8 min, t_R (major) = 12.3 min, >99% *ee*.

Racemic:

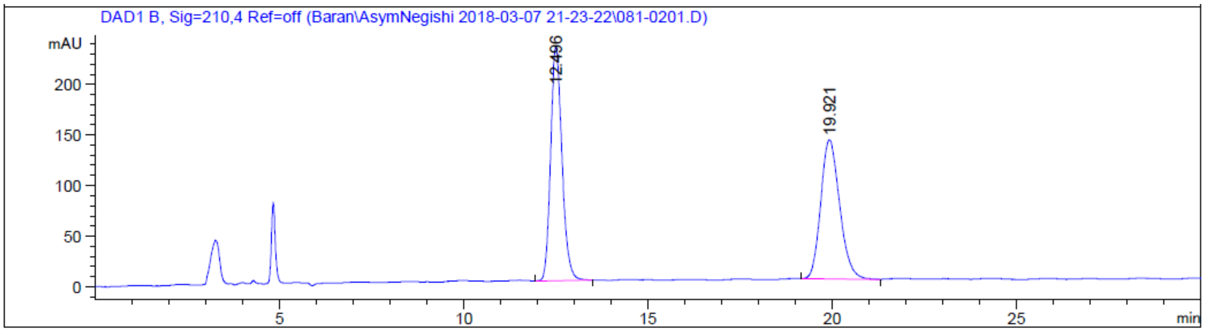

Enantioenriched **32**:

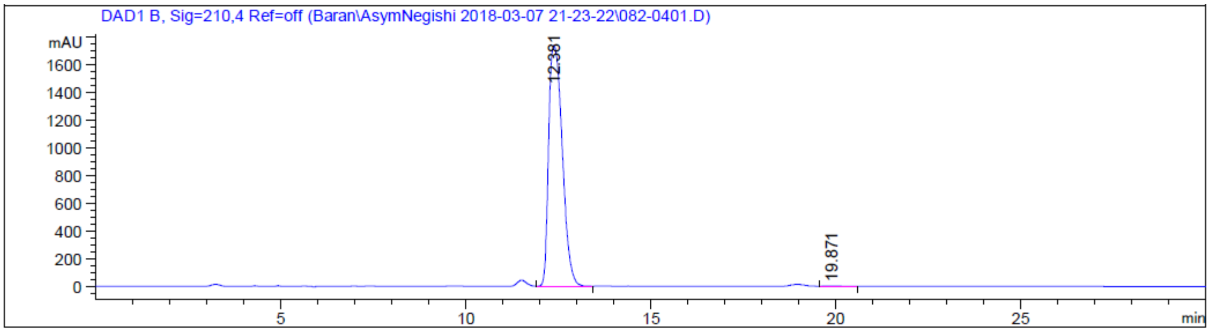

Signal 2: DAD1 B, Sig=210,4 Ref=off

Peak #	RetTime [min]	Type	Width [min]	Area [mAU*s]	Height [mAU]	Area %
1	12.496	BB	0.3277	4945.99561	231.08167	50.2319
2	19.921	BB	0.5458	4900.31934	137.50674	49.7681

Signal 2: DAD1 B, Sig=210,4 Ref=off

Peak #	RetTime [min]	Type	Width [min]	Area [mAU*s]	Height [mAU]	Area %
1	12.381	VB	0.4050	4.45020e4	1736.51575	99.8414
2	19.871	BB	0.3802	70.68443	2.25186	0.1586

Experimental Procedures and Characterization Data for Pyrrolidine Scaffolds (Figure 3–b)

Compound B10

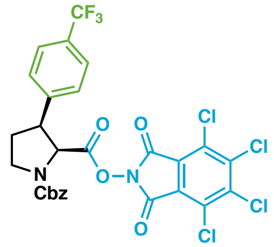

1-benzyl 2-(4,5,6,7-tetrachloro-1,3-dioxoisoindolin-2-yl) (2*S*,3*S*)-3-(4-(trifluoromethyl)phenyl)pyrrolidine-1,2-dicarboxylate (B10)

Following General Procedure **B** on 1.2 mmol scale with **A7**. Purification by flash column chromatography (silica, 4:1 Hexanes:EtOAc) afforded 842 mg (97%) of the title compound **B10**.

Physical State: yellow oil.

^{1}H NMR (600 MHz, $CDCl_3$, both rotamers): δ 7.64 (t, J = 8.5 Hz, 2H), 7.48 (d, J = 8.0 Hz, 2H), 7.47 – 7.39 (m, 2H), 7.40 – 7.28 (m, 3H), 5.45 – 5.29 (m, 1 H), 5.21 – 5.13 (m, 1H), 5.10 – 4.97 (m, 1H), 4.01 – 3.86 (m, 2H), 3.67 – 3.62 (m, 1H), 2.77 – 2.70 (m, 1H), 2.36 – 2.31 (m, 1H) ppm.

^{19}F NMR (400 MHz, $CDCl_3$): δ -62.90 ppm.

^{13}C NMR (151 MHz, $CDCl_3$, both rotamers): δ 166.5, 166.5, 156.6, 154.7, 154.0, 141.1, 141.0, 138.7, 138.5, 136.4, 130.5, 130.5(q, $J_{C\text{-}F}$ = 33.2 Hz), 130.5, 128.8, 128.7, 128.7, 128.7, 128.6, 128.3, 128.3, 125.9 (q, $J_{C\text{-}F}$ = 4.5Hz),124.7, 124.6, 124.2 (q, $J_{C\text{-}F}$ = 273.1 Hz), 124.2 (q, $J_{C\text{-}F}$ = 271.8 Hz), 68.0, 67.8, 62.5, 62.4, 48.2, 47.2, 46.3, 46.0, 28.3, 27.4 ppm.

HRMS (ESI-TOF): calc'd for $C_{28}H_{18}Cl_4F_3N_2O_6$ $[M+H]^+$: 674.987; found 674.9866.

TLC: R_f = 0.2 (4:1 Hexanes:EtOAc).

$[\alpha]_D^{20}$ = 55.3 (c = 1.0, $CHCl_3$).

Chiral HPLC: Chiralpak IA, 4.6 x 250 mm; 45/55 *i*PrOH/hexanes, 1 mL/min, 254 nm; t_R (minor) = 30.3 min, t_R (major) = 24.0 min, >99% *ee*.

Racemic:

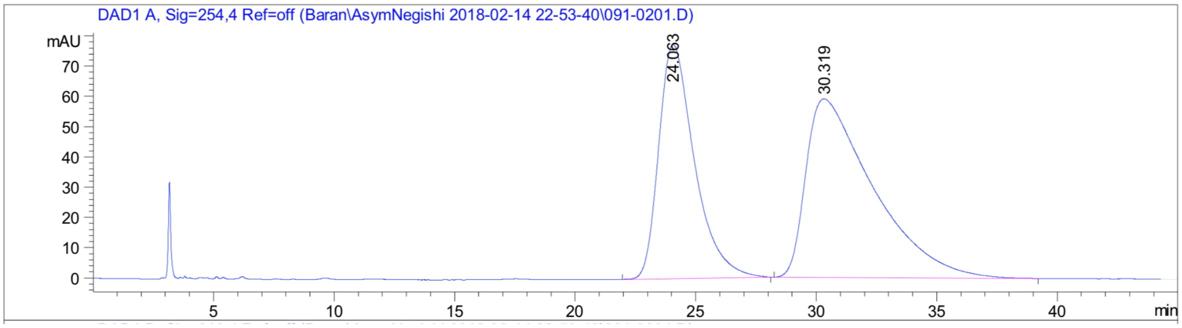

Enantioenriched **B10**:

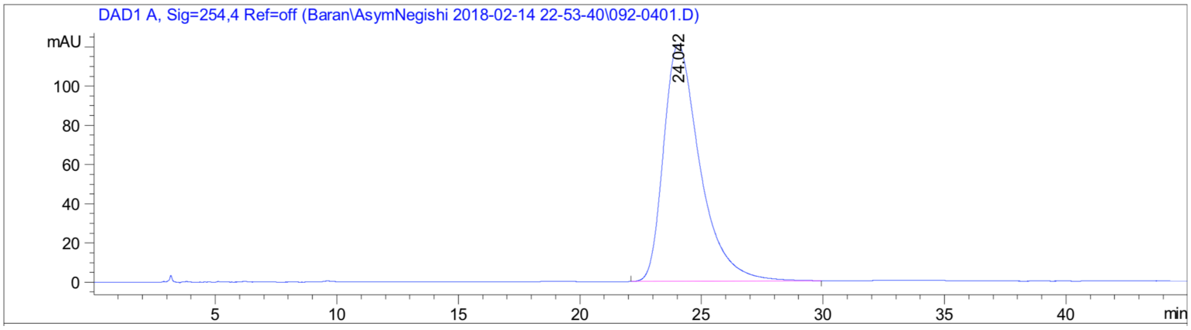

Signal 1: DAD1 A, Sig=254,4 Ref=off

Peak #	RetTime [min]	Type	Width [min]	Area [mAU*s]	Height [mAU]	Area %
1	24.063	BB	1.5191	7968.32715	77.39950	41.0140
2	30.319	BB	2.5466	1.14600e4	58.98722	58.9860

Signal 1: DAD1 A, Sig=254,4 Ref=off

Peak #	RetTime [min]	Type	Width [min]	Area [mAU*s]	Height [mAU]	Area %
1	24.042	BB	1.5656	1.25722e4	120.66509	100.0000

Compound 33

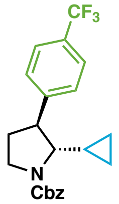

benzyl (2*S*,3*S*)-2-cyclopropyl-3-(4-(trifluoromethyl)phenyl)pyrrolidine-1-carboxylate (33)

Following General Procedure **C2** on 0.1 mmol scale with **B10**, $NiCl_2$•glyme (20 mol%), di^{t}BuBipy (25 mol%), and Zinc reagent **SI-27** (2.0 equiv.). Purification by pTLC (silica, 6:1 Hexanes:EtOAc) afforded 24.3 mg (62%) of the title compound **33**.

Physical State: colorless oil

^{1}H NMR (600 MHz, $CDCl_3$, both rotamers) δ 7.52 (d, J = 8.0 Hz, 2H), 7.36 – 7.33 (m, 5H), 7.22 (d, J = 7.6 Hz, 2H), 5.21 – 5.13 (m, 2H), 3.79 – 3.30 (m, 4H), 2.43 (dq, J = 14.8, 7.6 Hz, 1H), 1.97 – 1.89 (m, 1H), 1.05 – 0.94 (m, 1H), 0.72 – 0.42 (m, 3H), 0.08 (s, 1H) ppm.

^{19}F NMR (400 MHz, $CDCl_3$): δ -62.70 ppm.

^{13}C NMR (151 MHz, $CDCl_3$, both rotamers) δ 155.3, 148.1, 137.0, 129.0 (q, $J_{C\text{-}F}$ = 31.7 Hz), 128.6, 128.1, 127.3, 125.8 (q, $J_{C\text{-}F}$ = 3.8 Hz), 124.3 (q, $J_{C\text{-}F}$ = 271.8 Hz), 67.9, 67.1, 50.9, 50.0, 45.9, 32.5, 31.6, 16.5, 4.8, 2.0 ppm.

HRMS (ESI-TOF): calc'd for $C_{22}H_{23}F_3NO_2$ $[M+H]^+$: 390.1681; found 390.1682.

TLC: R_f = 0.3 (8:1 Hexanes:EtOAc).

$[\alpha]_D^{20}$ = -32.6 (c = 1.0, $CHCl_3$).

Chiral HPLC: Chiralpak IA, 4.6 x 250 mm; 2.5/97.5 *i*PrOH/hexanes, 0.5 mL/min, 210 nm; t_R (minor) = 21.2 min, t_R (major) = 19.8 min, >99% *ee*.

Racemic:

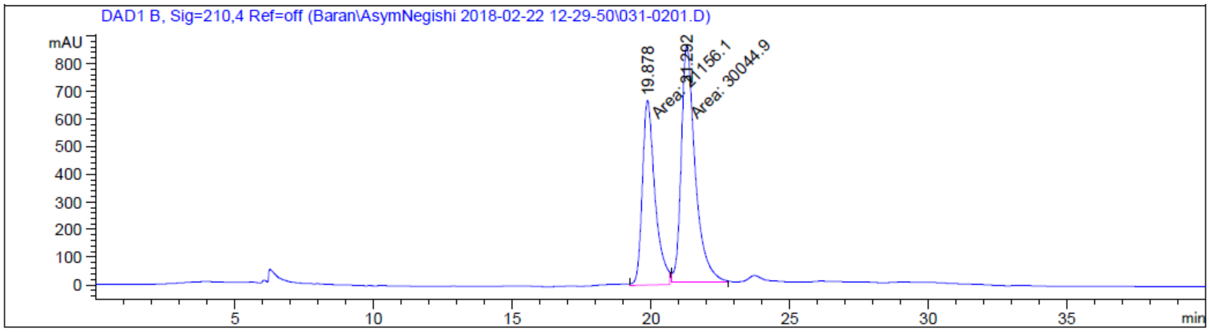

Enantioenriched **33**:

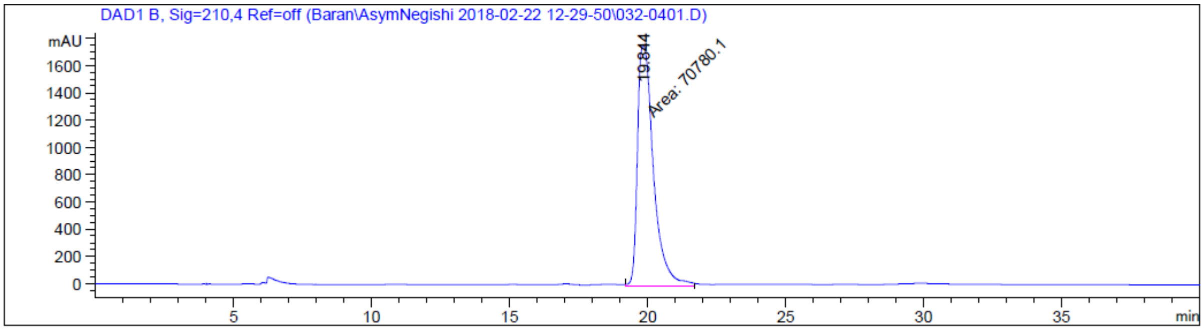

Signal 2: DAD1 B, Sig=210,4 Ref=off

Peak #	RetTime [min]	Type	Width [min]	Area [mAU*s]	Height [mAU]	Area %
1	19.878	MM	0.5287	2.11561e4	666.93622	41.3197
2	21.292	MM	0.5856	3.00449e4	855.16187	58.6803

Signal 2: DAD1 B, Sig=210,4 Ref=off

Peak #	RetTime [min]	Type	Width [min]	Area [mAU*s]	Height [mAU]	Area %
1	19.844	MM	0.6668	7.07801e4	1769.18909	100.0000

Compound 34

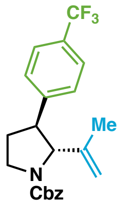

benzyl (2*R*,3*S*)-2-(prop-1-en-2-yl)-3-(4-(trifluoromethyl)phenyl)pyrrolidine-1-carboxylate (34)

Following General Procedure **C3** on 0.1 mmol scale with **B10**, Ni(acac)$_2$•xH$_2$O (30 mol%), 2,2'-bipyridine (30 mol%), and Zinc reagent **SI-29** (2.0 equiv.). Purification by pTLC (silica, 6:1 Hexanes:EtOAc) afforded 16.0 mg (41%) of the title compound **34**.

Physical State: colorless oil

^{1}H NMR (600 MHz, CDCl$_3$, both rotamers) δ 7.56 (d, *J* = 7.9 Hz, 2H), 7.38 – 7.29 (m, 7H), 5.22 – 5.05 (m, 2H), 4.86 – 4.80 (m, 1H), 4.71 – 4.64 (m, 1H), 4.39 – 4.22 (m, 1H), 3.87 – 3.79 (m, 1H), 3.56 (d, *J* = 9.3 Hz, 1H), 3.21 (d, *J* = 6.7 Hz, 1H), 2.33 – 2.26 (m, 1H), 1.98 (dd, *J* = 13.1, 7.1 Hz, 1H), 1.74 – 1.68 (m, 3H) ppm.

^{19}F NMR (400 MHz, CDCl$_3$): δ -62.73 ppm.

^{13}C NMR (151 MHz, CDCl$_3$, both rotamers) δ 155.1, 154.6, 146.5, 144.3, 143.7, 137.0, 136.9, 129.3 ((q, $J_{C\text{-}F}$ = 32.5 Hz),128.6, 128.5, 128.1, 128.0, 127.9, 127.7, 125.8 (q, $J_{C\text{-}F}$ = 3.8 Hz), 124.2 (q, $J_{C\text{-}F}$ = 271.8 Hz), 111.3, 111.2, 69.5, 67.1, 50.1, 49.0, 46.8, 46.4, 32.4, 31.6, 18.8 ppm.

HRMS (ESI-TOF): calc'd for $C_{22}H_{23}F_3NO_2$ [M+H]$^+$: 390.1681; found 390.1683.

TLC: R_f = 0.3 (8:1 Hexanes:EtOAc).

$[\alpha]_D^{20}$ = -36.1 (*c* = 1.0, CHCl$_3$).

Chiral HPLC: Chiralpak ODH, 4.6 x 250 mm; 5/95 *i*PrOH/hexanes, 0.5 mL/min, 210 nm; t_R (minor) = 17.8 min, t_R (major) = 16.3 min, 97.5% *ee*.

Racemic:

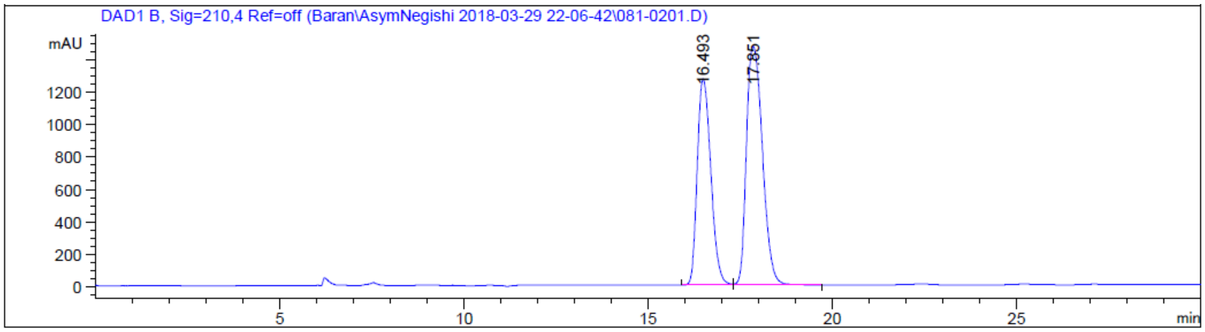

Enantioenriched **34**:

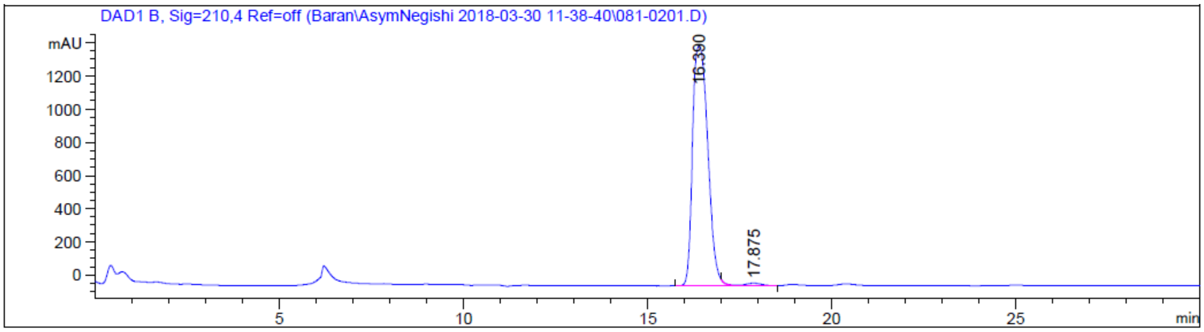

Signal 2: DAD1 B, Sig=210,4 Ref=off

Peak #	RetTime [min]	Type	Width [min]	Area [mAU*s]	Height [mAU]	Area %
1	16.493	BV	0.4010	3.25884e4	1271.60510	42.2750
2	17.851	VB	0.4770	4.44983e4	1470.84521	57.7250

Signal 2: DAD1 B, Sig=210,4 Ref=off

Peak #	RetTime [min]	Type	Width [min]	Area [mAU*s]	Height [mAU]	Area %
1	16.390	BV R	0.4628	4.16565e4	1442.90723	98.8485
2	17.875	VV E	0.4328	485.25125	15.51845	1.1515

Compound 35

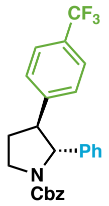

benzyl (2*R*,3*S*)-2-phenyl-3-(4-(trifluoromethyl)phenyl)pyrrolidine-1-carboxylate (35)

Following General Procedure **C4** on 0.1 mmol scale with **B10**, $NiCl_2$•glyme (20 mol%), di^{t}BuBipy (25 mol%), and Zinc reagent **SI-35** (2.0 equiv.). Purification by pTLC (silica, 6:1 Hexanes:EtOAc) afforded 31.2 mg (73%) of the title compound **35**.

Physical State: colorless oil

^{1}H NMR (600 MHz, $CDCl_3$, both rotamers δ 7.55 (d, J = 7.9 Hz, 2H), 7.42 – 7.21 (m, 7H), 7.20 – 7.04 (m, 4H), 6.79 (d, J = 7.4 Hz, 1H), 5.25 – 4.80 (m, 3H), 4.06 – 3.75 (m, 2H), 3.34 (s, 1H), 2.37 – 2.32 (m, 1H), 2.10 (s, 1H) ppm.

^{19}F NMR (400 MHz, $CDCl_3$): δ -62.73 ppm.

^{13}C NMR (151 MHz, $CDCl_3$, both rotamers δ 154.9, 154.7, 145.6, 145.4, 142.9, 142.1, 136.9, 136.5, 129.5 (q, J_{C-F} = 32.6 Hz), 128.7, 128.3, 128.1, 127.8, 127.7, 127.4, 127.3, 125.8 (q, J_{C-F} = 3.9 Hz), 125.7, 124.2 (q, J_{C-F} = 271.8 Hz), 68.3, 67.1, 67.0, 55.3, 54.1, 47.5, 47.0, 32.5, 31.8 ppm.

HRMS (ESI-TOF): calc'd for $C_{25}H_{23}F_3NO_2$ $[M+H]^+$: 426.1681; found 426.1687.

TLC: R_f = 0.3 (8:1 Hexanes:EtOAc).

$[\alpha]_D^{20}$ = -67.6 (c = 1.0, $CHCl_3$).

Chiral HPLC: Chiralpak IC, 4.6 x 250 mm; 20/80 *i*PrOH/hexanes, 1 mL/min, 210 nm; t_R (minor) = 8.0 min, t_R (major) = 6.6 min, 96% *ee*.

Racemic:

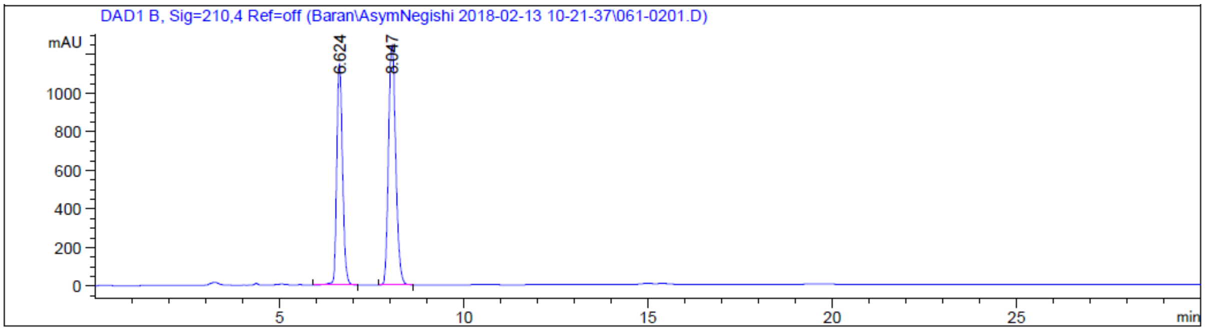

Enantioenriched **35**:

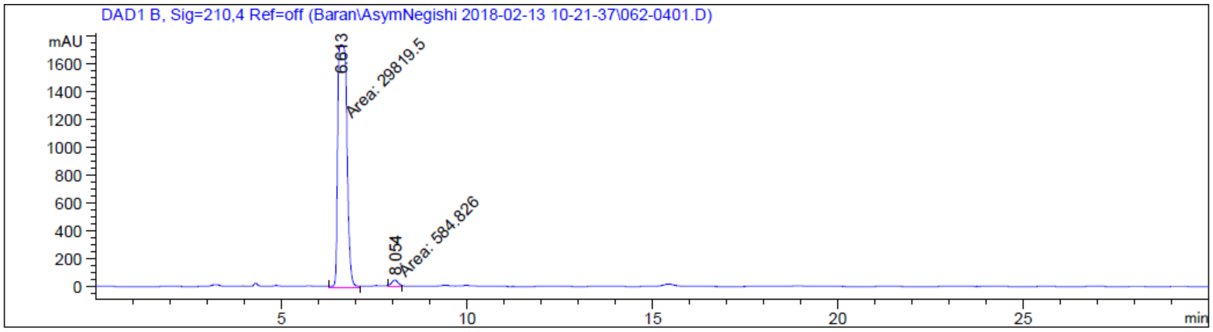

Signal 2: DAD1 B, Sig=210,4 Ref=off

Peak #	RetTime [min]	Type	Width [min]	Area [mAU*s]	Height [mAU]	Area %
1	6.624	BB	0.1678	1.23321e4	1144.03088	42.0382
2	8.047	BB	0.2159	1.70034e4	1240.74731	57.9618

Signal 2: DAD1 B, Sig=210,4 Ref=off

Peak #	RetTime [min]	Type	Width [min]	Area [mAU*s]	Height [mAU]	Area %
1	6.613	MM	0.2852	2.98195e4	1742.56702	98.0765
2	8.054	MM	0.2145	584.82611	45.44874	1.9235

Compound 36

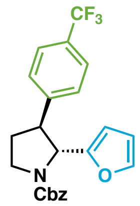

benzyl (2*R*,3*S*)-2-(furan-2-yl)-3-(4-(trifluoromethyl)phenyl)pyrrolidine-1-carboxylate (36)

Following General Procedure **C1** on 0.1 mmol scale with **B10**, $NiCl_2 \cdot 6H_2O$ (20 mol%), BPhen (22 mol%), and boronic acid **SI-20** (3.0 equiv.). Purification by pTLC (silica, 5:1 Hexanes:EtOAc) afforded 35.0 mg (84%) of the title compound **36**.

Physical State: colorless oil

^{1}H NMR (600 MHz, $CDCl_3$, both rotamers): δ 7.55 (d, J = 8.0 Hz, 2H), 7.40 – 7.21 (m, 7H), 7.14 (s, 1H), 6.30 (s, 1H), 6.22 – 6.04 (m, 1H), 5.23 – 4.89 (m, 3H), 3.87 – 3.81 (m, 1H), 3.71 – 3.66 (m, 2H), 2.48 (s, 1H), 2.10 – 2.04 (m, 1H) ppm.

^{19}F NMR (400 MHz, $CDCl_3$): δ -62.75 ppm.

^{13}C NMR (151 MHz, $CDCl_3$, both rotamers) δ 154.7, 154.1, 153.5, 145.9, 141.9, 136.8, 136.6, 129.4 (q, J_{C-F} = 32.5 Hz), 128.7, 128.6, 128.4, 128.0, 127.8, 127.4, 125.8 (q, J_{C-F} = 3.7 Hz), 124.2 (q, J_{C-F} = 271.8 Hz), 110.5, 110.4, 107.5, 106.9, 67.2, 61.4, 50.52, 5.19, 46.3, 45.9, 32.2, 31.3 ppm.

HRMS (ESI-TOF): calc'd for $C_{23}H_{21}F_3NO_3$ $[M+H]^+$: 416.1474; found 416.1469.

TLC: R_f = 0.3 (8:1 Hexanes:EtOAc).

$[\alpha]_D^{20}$ = -57.0 (c = 1.0, $CHCl_3$).

Chiral HPLC: Chiralpak IC, 4.6 x 250 mm; 10/90 *i*PrOH/hexanes, 1 mL/min, 254 nm; t_R (minor) = 9.5 min, t_R (major) = 8.5 min, 95.5% *ee*.

Racemic:

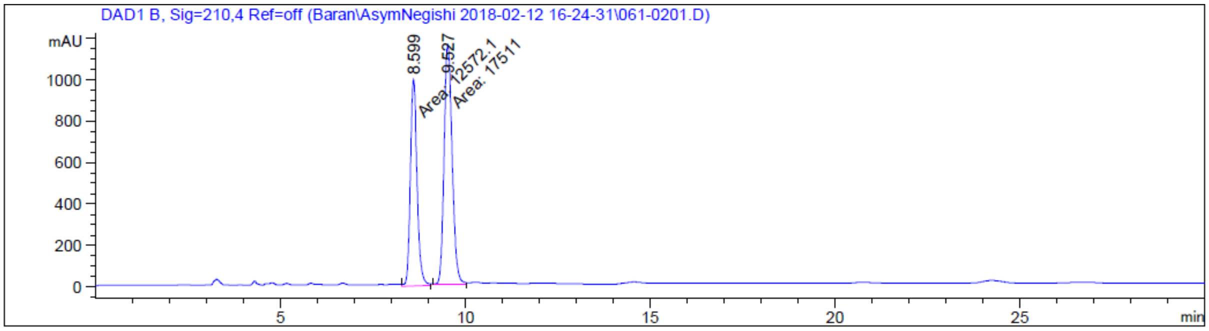

Enantioenriched **36**:

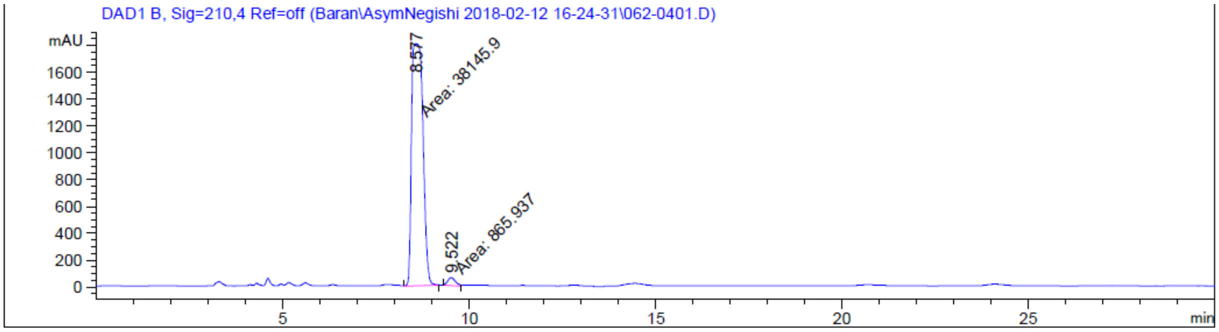

Signal 2: DAD1 B, Sig=210,4 Ref=off

Peak #	RetTime [min]	Type	Width [min]	Area [mAU*s]	Height [mAU]	Area %
1	8.599	MM	0.2103	1.25721e4	996.45453	41.7912
2	9.527	MM	0.2526	1.75110e4	1155.45422	58.2088

Signal 2: DAD1 B, Sig=210,4 Ref=off

Peak #	RetTime [min]	Type	Width [min]	Area [mAU*s]	Height [mAU]	Area %
1	8.577	MM	0.3528	3.81459e4	1802.20972	97.7803
2	9.522	MM	0.2449	865.93713	58.93631	2.2197

Compound 37

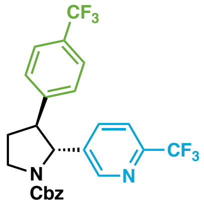

benzyl (2*R*,3*S*)-3-(4-(trifluoromethyl)phenyl)-2-(6-(trifluoromethyl)pyridin-3-yl)pyrrolidine-1-carboxylate (37)

Following General Procedure **C1** on 0.1 mmol scale with **B10**, $NiCl_2 \cdot 6H_2O$ (20 mol%), BPhen (22 mol%), and boronic acid **SI-14** (3.0 equiv.). Purification by pTLC (silica, 2:1 Hexanes:EtOAc) afforded 35.0 mg (71%) of the title compound **37**.

Physical State: colorless oil

^{1}H NMR (600 MHz, $CDCl_3$, both rotamers): δ 8.48 – 8.35 (m, 1H), 7.63 – 7.45 (m, 4H), 7.43 – 7.33 (m, 2H), 7.27 – 7.14 (m, 4H), 6.81 (d, *J* = 7.5 Hz, 1H), 5.25 – 4.75 (m, 3H), 4.12 – 4.04 (m, 1H), 3.80 – 3.76 (m, 1H), 3.30 (dt, *J* = 9.6, 7.0 Hz, 1H), 2.39 – 2.32 (m, 1H), 2.24 – 2.20 (m, 1H) ppm.

^{19}F NMR (400 MHz, $CDCl_3$): δ -62.88, 67.96 ppm.

^{13}C NMR (151 MHz, $CDCl_3$, both rotamers) δ 163.0, 155.0, 154.5, 148.0, 147.4 (q, J_{C-F} = 34.8 Hz), 143.6, 143.3, 141.6, 140.8, 136.4, 135.7, 134.8, 134.5, 130.2 (q, J_{C-F} = 31.7 Hz), 128.7, 128.5, 128.4, 128.3, 128.2, 128.0, 127.9, 126.1 (q, J_{C-F} = 3.8 Hz), 124.4, 124.3, 124.0 (q, J_{C-F} = 271.8 Hz), 121.6 (q, J_{C-F} = 273.3 Hz),120.4, 67.6, 66.2, 55.6, 54.4, 47.7, 47.3, 33.1, 32.4 ppm.

HRMS (ESI-TOF): calc'd for $C_{25}H_{21}F_6N_2O_2$ $[M+H]^+$: 495.1507; found 495.1507.

TLC: R_f = 0.2 (3:1 Hexanes:EtOAc).

$[\alpha]_D^{20}$ = -39.7 (*c* = 1.0, $CHCl_3$).

Chiral HPLC: Chiralpak IC, 4.6 x 250 mm; 20/80 *i*PrOH/hexanes, 1 mL/min, 254 nm; t_R (minor) = 6.3 min, t_R (major) = 7.8 min, 98% *ee*.

Racemic:

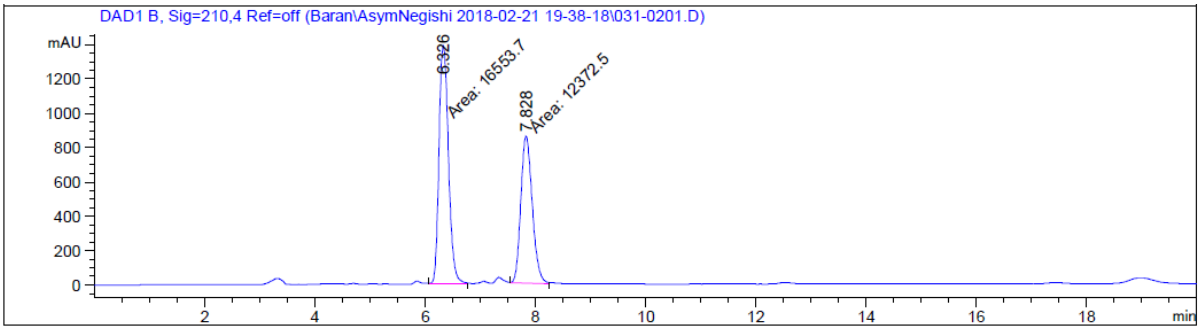

Enantioenriched **37**:

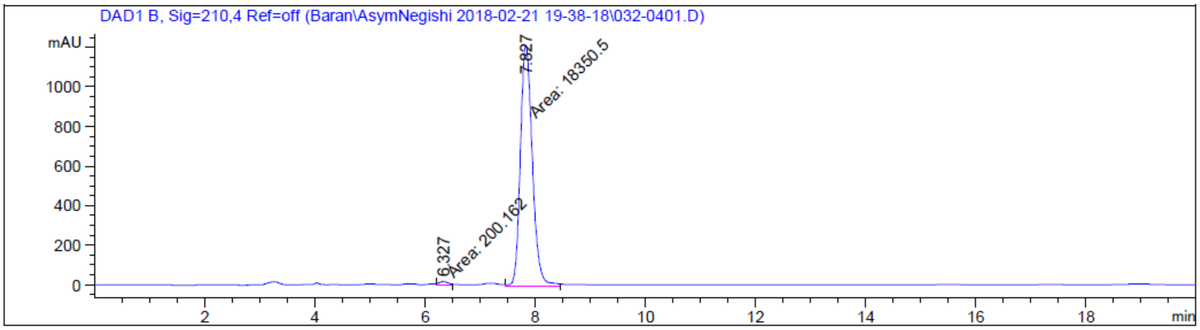

Signal 2: DAD1 B, Sig=210,4 Ref=off

Peak #	RetTime [min]	Type	Width [min]	Area [mAU*s]	Height [mAU]	Area %
1	6.326	MM	0.1989	1.65537e4	1387.16516	57.2273
2	7.828	MM	0.2408	1.23725e4	856.41144	42.7727

Signal 2: DAD1 B, Sig=210,4 Ref=off

Peak #	RetTime [min]	Type	Width [min]	Area [mAU*s]	Height [mAU]	Area %
1	6.327	MM	0.1934	200.16231	17.25369	1.0790
2	7.827	MM	0.2517	1.83505e4	1215.01892	98.9210

Compound 38

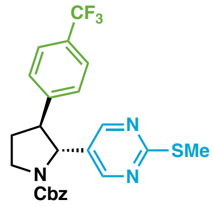

benzyl (2*R*,3*S*)-2-(2-(methylthio)pyrimidin-5-yl)-3-(4-(trifluoromethyl)phenyl)pyrrolidine-1-carboxylate (38)

Following General Procedure **C1** on 0.1 mmol scale with **B10**, $NiCl_2•6H_2O$ (20 mol%), BPhen (22 mol%), and boronic acid **SI-18** (3.0 equiv.). Purification by pTLC (silica, 2:1 Hexanes:EtOAc) afforded 40.2 mg (85%) of the title compound **38**.

Physical State: colorless oil

^{1}H NMR (600 MHz, $CDCl_3$, both rotamers): δ 8.22 (s, 1H), 8.11 (s, 1H), 7.50 (d, J = 7.8 Hz, 2H), 7.31 – 7.19 (m, 6H), 6.86 (s, 1H), 5.14 – 4.82 (m, 2H), 4.72 – 4.57 (m, 1H), 4.00 – 3.93 (m, 1H), 3.66 (d, J = 8.9 Hz, 1H), 3.23 (dt, J = 9.6, 6.8 Hz, 1H), 2.47 (s, 3H), 2.32 – 2.24 (m, 1H), 2.13 – 2.09 (m, 1H) ppm.

^{19}F NMR (400 MHz, $CDCl_3$): δ -62.87 ppm.

^{13}C NMR (151 MHz, $CDCl_3$, both rotamers δ 172.0, 155.3, 154.8, 154.7, 154.5, 143.5, 143.2, 136.4, 135.9, 130.5, 130.1 (q, J_{C-F} = 32.6 Hz), 129.7, 128.7, 128.6, 128.3, 128.2, 128.0, 127.8, 126.1 (q, J_{C-F} = 3.7 Hz), 124.0 (q, J_{C-F} = 271.8 Hz), 67.6, 64.4, 55.3, 54.1, 47.6, 47.2, 33.00, 32.2, 14.2 ppm.

HRMS (ESI-TOF): calc'd for $C_{24}H_{23}F_3N_3O_2S$ $[M+H]^+$: 474.1463; found 474.1465.

TLC: R_f = 0.2 (2:1 Hexanes:EtOAc).

$[\alpha]_D^{20}$ = -65.0 (c = 1.0, $CHCl_3$).

Chiral HPLC: Chiralpak IC, 4.6 x 250 mm; 30/70 *i*PrOH/hexanes, 1 mL/min, 254 nm; t_R (minor) = 9.0 min, t_R (major) = 24.4 min, 98.5% *ee*.

Racemic:

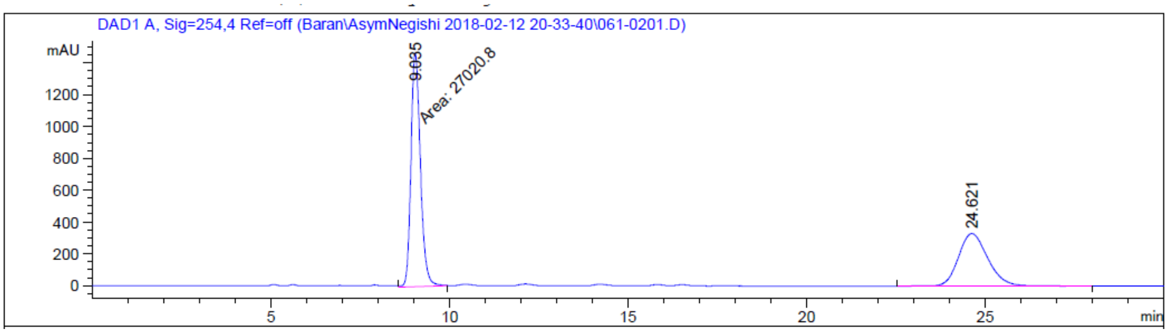

Enantioenriched **38**:

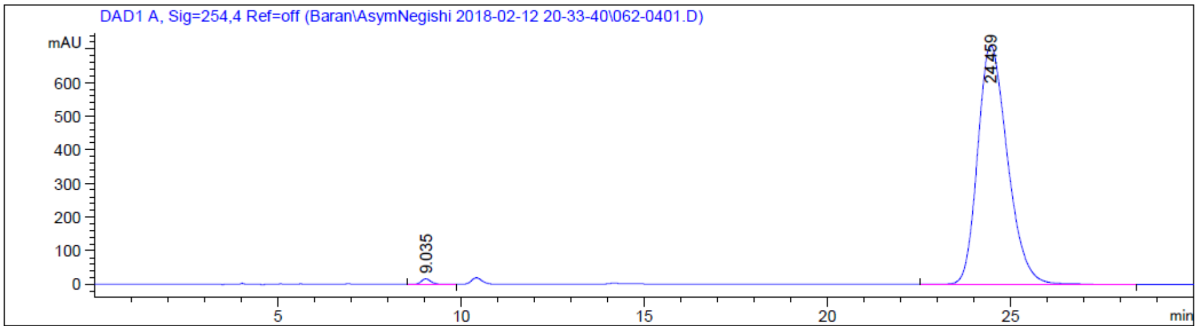

Signal 1: DAD1 A, Sig=254,4 Ref=off

Peak #	RetTime [min]	Type	Width [min]	Area [mAU*s]	Height [mAU]	Area %
1	9.035	MM	0.3073	2.70208e4	1465.43604	59.0692
2	24.621	BB	0.8803	1.87235e4	328.85065	40.9308

Signal 1: DAD1 A, Sig=254,4 Ref=off

Peak #	RetTime [min]	Type	Width [min]	Area [mAU*s]	Height [mAU]	Area %
1	9.035	BB	0.2826	298.68182	16.23542	0.7392
2	24.459	BB	0.8744	4.01099e4	708.56226	99.2608

Compound 39

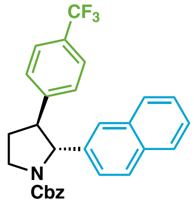

benzyl (2*R*,3*S*)-2-(naphthalen-2-yl)-3-(4-(trifluoromethyl)phenyl)pyrrolidine-1-carboxylate (39)

Following General Procedure C1 on 0.1 mmol scale with **B10**, $NiCl_2$•$6H_2O$ (20 mol%), BPhen (22 mol%), and boronic acid **SI-11** (3.0 equiv.). Purification by pTLC (silica, 3:1 Hexanes:EtOAc) afforded 40.1 mg (84%) of the title compound **39**.

Physical State: colorless oil

^{1}H NMR (600 MHz, $CDCl_3$, both rotamers): δ 7.86 – 7.78 (m, 2H), 7.74 – 7.70 (m, 1H), 7.59 – 7.32 (m, 7H), 7.26 (s, 3H), 7.09 – 6.83 (m, 2H), 6.63 (d, J = 5.7 Hz, 1H), 5.25 – 4.84 (m, 3H), 4.06 (d, J = 51.2 Hz, 1H), 3.88 (s, 1H), 3.45 (t, J = 8.1 Hz, 1H), 2.41 – 2.36 (m, 1H), 2.24 – 2.11 (m, 1H) ppm.

^{19}F NMR (400 MHz, $CDCl_3$): δ -62.75 ppm.

^{13}C NMR (151 MHz, $CDCl_3$, both rotamers) δ 162.8, 155.0, 154.8, 145.5, 145.2, 140.2, 139.5, 136.9, 136.2, 133.4, 132.9, 129.5 (q, $J_{C\text{-}F}$ = 31.7Hz), 128.7, 128.2, 128.1, 128.0, 127.9, 127.8, 127.6, 127.4, 126.3, 125.9, 125.8 (q, $J_{C\text{-}F}$ = 3.8 Hz), 124.7, 124.6, 124.2 (q, $J_{C\text{-}F}$ = 271.8 Hz), 123.9, 68.6, 67.1, 55.2, 54.0, 47.7, 47.1, 32.6, 31.9 ppm.

HRMS (ESI-TOF): calc'd for $C_{29}H_{25}F_3NO_2$ $[M+H]^+$: 476.1837; found 476.1832.

TLC: R_f = 0.3 (8:1 Hexanes:EtOAc).

$[\alpha]_D^{20}$ = -31.0 (c = 1.0, $CHCl_3$).

Chiral HPLC: Chiralpak IC, 4.6 x 250 mm; 10/90 *i*PrOH/hexanes, 1 mL/min, 210 nm; t_R (minor) = 12.7 min, t_R (major) = 10.8 min, 97% *ee*.

Racemic:

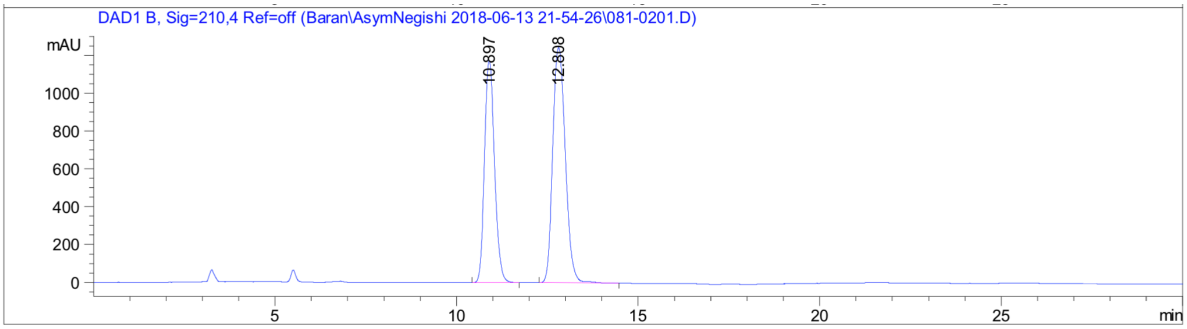

Enantioenriched **39**:

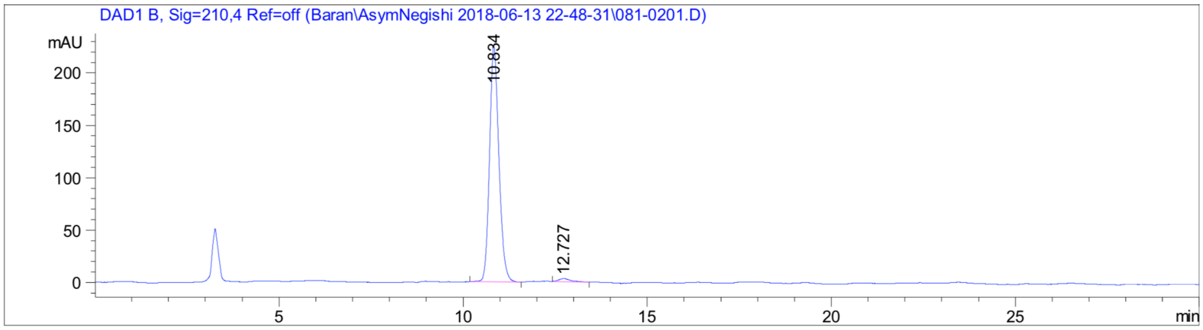

Signal 2: DAD1 B, Sig=210,4 Ref=off

Peak #	RetTime [min]	Type	Width [min]	Area [mAU*s]	Height [mAU]	Area %
1	10.897	BB	0.2994	2.25021e4	1175.09668	42.8811
2	12.808	BB	0.3814	2.99735e4	1242.72571	57.1189

Signal 2: DAD1 B, Sig=210,4 Ref=off

Peak #	RetTime [min]	Type	Width [min]	Area [mAU*s]	Height [mAU]	Area %
1	10.834	BB	0.2704	3997.51758	225.96608	98.5336
2	12.727	BB	0.2506	59.49097	2.90896	1.4664

Compound 40

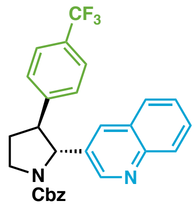

benzyl (2*R*,3*S*)-2-(quinolin-3-yl)-3-(4-(trifluoromethyl)phenyl)pyrrolidine-1-carboxylate (40)

Following General Procedure **C1** on 0.1 mmol scale with **B10**, $NiCl_2$•$6H_2O$ (20 mol%), BPhen (22 mol%), and boronic acid **SI-16** (3.0 equiv.). Purification by pTLC (silica, 2:1 Hexanes: EtOAc) afforded 24.0 mg (50%) of the title compound **40**.

Physical State: colorless oil

^{1}H NMR (600 MHz, $CDCl_3$, both rotamers): δ 8.74 – 8.64 (m, 1H), 8.10 (d, J = 8.5 Hz, 1H), 7.85 – 7.63 (m, 3H), 7.56 (d, J = 8.1 Hz, 3H), 7.44 – 7.23 (m, 4H), 7.08 – 6.68 (m, 3H), 5.21 – 4.83 (m, 3H), 4.13 – 4.06 (m, 1H), 3.87 (s, 1H), 3.47 – 3.44 (m, 1H), 2.43 – 2.38 (m, 1H), 2.22 (s, 1H) ppm.

^{19}F NMR (400 MHz, $CDCl_3$): δ -62.81 ppm.

^{13}C NMR (151 MHz, $CDCl_3$, both rotamers δ 154.8, 149.2, 147.8, 144.4, 144.1, 136.7, 135.9, 135.4, 134.6, 132.8, 132.5, 129.9 (q, $J_{C\text{-}F}$ = 33.2Hz), 129.4, 129.4, 128.7, 128.2, 127.9, 127.7, 127.0, 126.0 (q, $J_{C\text{-}F}$ = 3.7Hz), 124.1 (q, $J_{C\text{-}F}$ = 271.8 Hz), 67.4, 66.7, 55.4, 54.3, 47.7, 47.3, 32.9, 32.2 ppm.

HRMS (ESI-TOF): calc'd for $C_{28}H_{24}F_3N_2O_2$ $[M+H]^+$: 477.1790; found 477.1788.

TLC: R_f = 0.2 (2:1 Hexanes:EtOAc).

$[\alpha]_D^{20}$ = -11.0 (c = 1.0, $CHCl_3$).

Chiral HPLC: Chiralpak IC, 4.6 x 250 mm; 30/70 *i*PrOH/hexanes, 1 mL/min, 254 nm; t_R (minor) = 9.7 min, t_R (major) = 22.6 min, 98.5% *ee*.

Racemic:

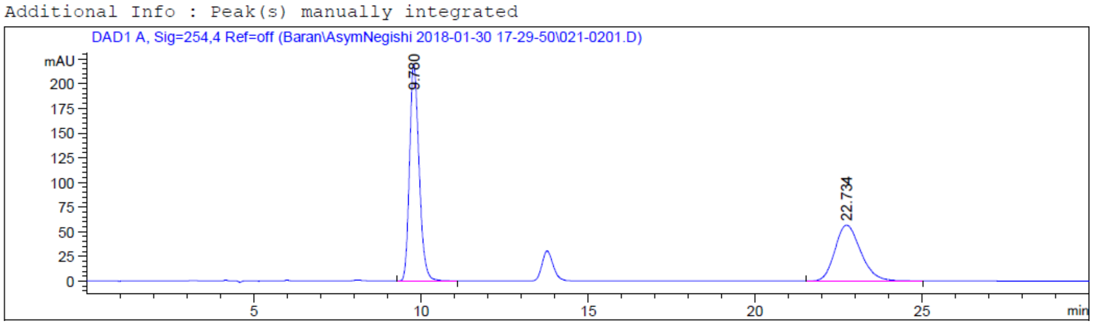

Enantioenriched **40**:

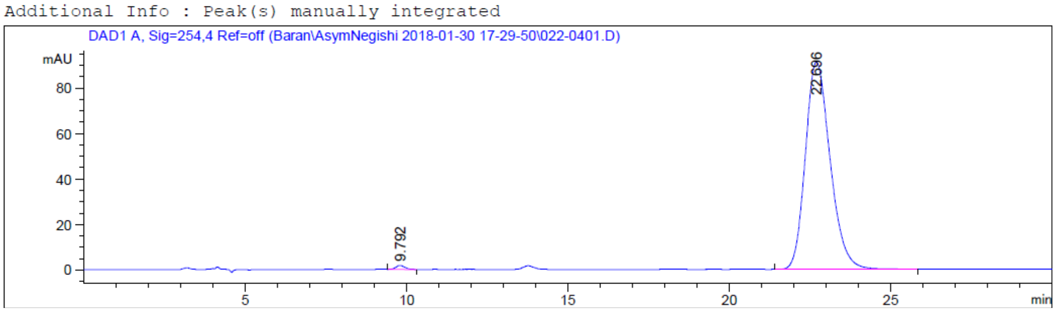

Signal 1: DAD1 A, Sig=254,4 Ref=off

Peak #	RetTime [min]	Type	Width [min]	Area [mAU*s]	Height [mAU]	Area %
1	9.780	BB	0.3060	4364.56104	219.45813	59.0228
2	22.734	BB	0.8259	3030.14307	56.66148	40.9772

Signal 1: DAD1 A, Sig=254,4 Ref=off

Peak #	RetTime [min]	Type	Width [min]	Area [mAU*s]	Height [mAU]	Area %
1	9.792	BB	0.3034	33.80537	1.73459	0.6880
2	22.696	BB	0.8191	4879.43604	91.35440	99.3120

Compound B11

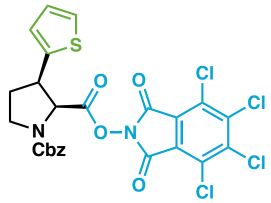

1-benzyl 2-(4,5,6,7-tetrachloro-1,3-dioxoisoindolin-2-yl) (2*S*,3*R*)-3-(thiophen-2-yl)pyrrolidine-1,2-dicarboxylate (B11)

Following General Procedure **B** on 1.03 mmol scale with **A8**. Purification by flash column chromatography (silica, 5:1 Hexanes:EtOAc) afforded 517 mg (79%) of the title compound **B11**.

Physical State: yellow oil.

^{1}H NMR (600 MHz, $CDCl_3$, both rotamers): δ 7.47 – 7.39 (m, 2H), 7.38 – 7.33 (m, 2H), 7.33 – 7.25 (m, 2H), 7.11 (t, J = 4.6 Hz, 1H), 7.07 – 7.03 (m, 1H), 5.45 – 4.94 (m, 3H), 4.09 – 3.99 (m, 1H), 3.96 – 3.87 (m, 1H), 3.66 – 3.58 (m, 1H), 2.68 (d, J = 17.6 Hz, 1H), 2.43 (d, J = 18.2 Hz, 1H) ppm.

^{13}C NMR (151 MHz, $CDCl_3$, both rotamers): δ 166.6, 166.5, 156.6, 156.6, 154.5, 153.99, 141.0, 140.9, 137.2, 137.0, 136.5, 136.4, 130.4, 130.4, 128.6, 128.6, 128.5, 128.3, 128.2, 128.2, 127.6, 126.3, 125.2, 124.7, 124.7, 67.8, 67.7, 62.8, 62.7, 46.1, 45.8, 43.5, 42.6, 30.3, 29.2 ppm.

HRMS (ESI-TOF): calc'd for $C_{25}H_{17}Cl_4N_2O_6S$ $[M+H]^+$: 612.9561; found 612.9562.

TLC: R_f = 0.3 (4:1 Hexanes:EtOAc).

$[\alpha]_D^{20}$ = 35.5 (c = 1.0, $CHCl_3$).

Chiral HPLC: Chiralpak IC, 4.6 x 250 mm; 45/55 *i*PrOH/hexanes, 1 mL/min, 254 nm; t_R (minor) = 37.1 min, t_R (major) = 32.3 min, >99% *ee*.

Racemic:

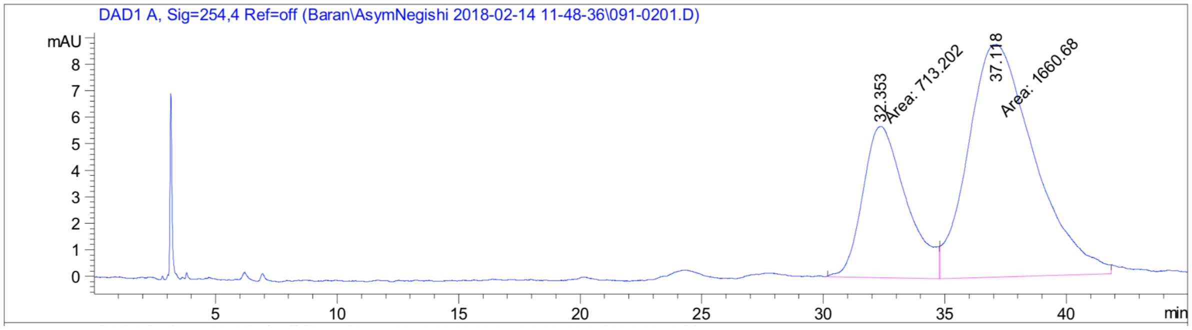

Enantioenriched **B11**:

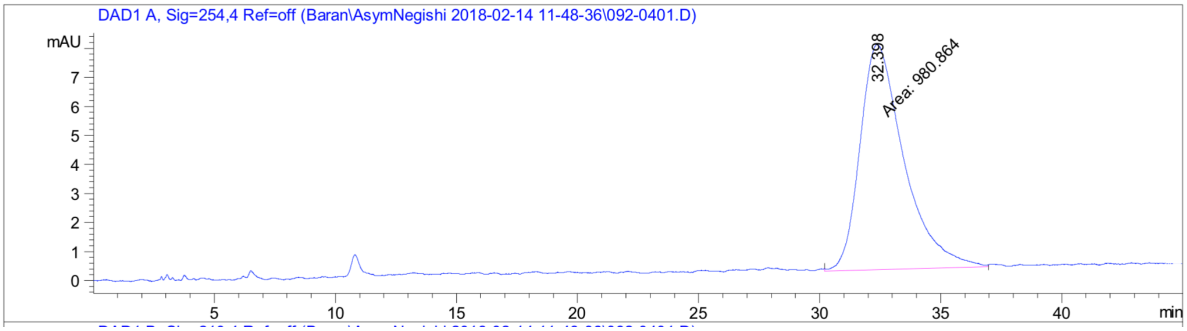

Signal 1: DAD1 A, Sig=254,4 Ref=off

Peak #	RetTime [min]	Type	Width [min]	Area [mAU*s]	Height [mAU]	Area %
1	32.353	MM	2.0849	713.20221	5.70135	30.0436
2	37.118	MM	3.1551	1660.68494	8.77246	69.9564

Signal 1: DAD1 A, Sig=254,4 Ref=off

Peak #	RetTime [min]	Type	Width [min]	Area [mAU*s]	Height [mAU]	Area %
1	32.398	MM	2.1089	980.86444	7.75190	100.0000

Compound 41

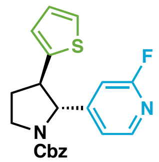

benzyl (2*R*,3*R*)-2-(2-fluoropyridin-4-yl)-3-(thiophen-2-yl)pyrrolidine-1-carboxylate (41)
Following General Procedure **C1** on 0.1 mmol scale with **B11**, $NiCl_2 \cdot 6H_2O$ (20 mol%), BPhen (22 mol%), and boronic acid **SI-17** (3.0 equiv.). Purification by pTLC (silica, 6:1 Hexanes: EtOAc) afforded 31.3 mg (82%) of the title compound **41**.

Physical State: colorless oil
^{1}H NMR (600 MHz, $CDCl_3$, both rotamers): δ 8.16 – 8.06 (m, 1H), 7.36 (d, J = 19.3 Hz, 2H), 7.21 (d, J = 4.6 Hz, 3H), 6.99 – 6.88 (m, 3H), 6.80 – 6.61 (m, 2H), 5.21 – 4.75 (m, 3H), 4.02 (d, J = 44.9 Hz, 1H), 3.72 (q, J = 7.8 Hz, 1H), 3.52 (s, 1H), 2.38 – 2.33 (m, 1H), 2.21 – 2.14 (m, 1H) ppm.
^{19}F NMR (400 MHz, $CDCl_3$): δ -70.37, -70.48 ppm.
^{13}C NMR (151 MHz, $CDCl_3$, both rotamers): δ 164.3 (d, $J_{C\text{-}F}$ = 237.1Hz), 164.2 (d, $J_{C\text{-}F}$ = 240.1Hz), 158.0, 157.9, 157.2, 157.2 – 157.1 (m), 154.9, 154.6, 147.9(d, $J_{C\text{-}F}$ = 15.1Hz), 143.3, 143.0, 136.5, 135.9, 128.7, 128.5, 128.3, 128.2, 127.8, 127.1, 124.9, 124.5, 118.8, 118.7, 106.7 (q, $J_{C\text{-}F}$ = 37.8Hz), 106.6 (d, $J_{C\text{-}F}$ = 39.3Hz), 68.2, 68.1, 67.5, 50.3, 49.3, 47.4, 46.9, 33.6, 33.1 ppm.
HRMS (ESI-TOF): calc'd for $C_{21}H_{20}FN_2O_2S$ $[M+H]^+$: 383.1230; found 383.1230.
TLC: R_f = 0.2 (4:1 Hexanes:EtOAc).
$[\alpha]_D^{20}$ = -33.0 (c = 1.0, $CHCl_3$).
Chiral HPLC: Chiralpak IC, 4.6 x 250 mm; 45/55 *i*PrOH/hexanes, 1 mL/min, 210 nm; t_R (minor) = 8.2 min, t_R (major) = 20.5 min, 99% *ee*.

Racemic:

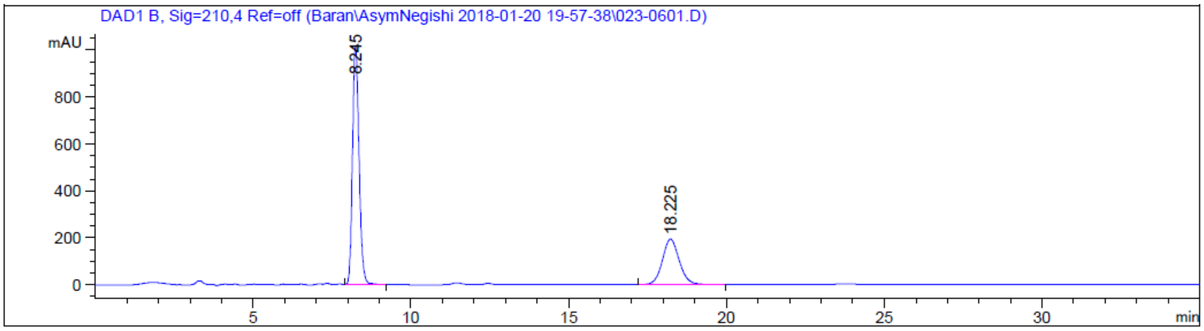

Enantioenriched **41**:

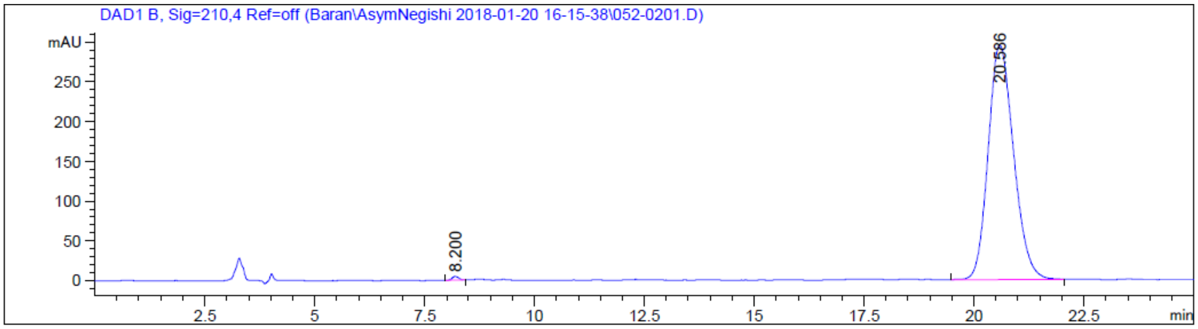

Signal 2: DAD1 B, Sig=210,4 Ref=off

Peak #	RetTime [min]	Type	Width [min]	Area [mAU*s]	Height [mAU]	Area %
1	8.245	BB	0.2246	1.46885e4	1016.64648	67.1379
2	18.225	BB	0.5675	7189.58691	193.48946	32.8621

Signal 2: DAD1 B, Sig=210,4 Ref=off

Peak #	RetTime [min]	Type	Width [min]	Area [mAU*s]	Height [mAU]	Area %
1	8.200	BB	0.1642	49.02410	4.75802	0.4102
2	20.586	BB	0.6036	1.19024e4	295.71375	99.5898

Compound 42

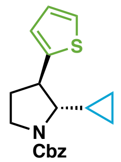

benzyl (2*S*,3*R*)-2-cyclopropyl-3-(thiophen-2-yl)pyrrolidine-1-carboxylate (42)

Following General Procedure **C2** on 0.05 mmol scale with **B11**, $NiCl_2$•glyme (20 mol%), di^{t}BuBipy (25 mol%), and Zinc reagent **SI-27** (2.0 equiv.). Purification by pTLC (silica, 4:1 Hexanes:EtOAc) afforded 12.0 mg (73%) of the title compound **42**.

Physical State: colorless oil

^{1}H NMR (600 MHz, $CDCl_3$, both rotamers): δ 7.39 – 7.29 (m, 5H), 7.12 (dd, J = 5.1, 1.2 Hz, 1H), 6.89 (dd, J = 5.1, 3.5 Hz, 1H), 6.77 (s, 1H), 5.22 – 5.08 (m, 2H), 3.71 (s, 1H), 3.62 – 3.49 (m, 3H), 2.48 – 2.42 (m, 1H), 2.03 – 1.99 (m, 1H), 0.95 (s, 1H), 0.77 – 0.36 (m, 3H), 0.20 (s, 1H) ppm.

^{13}C NMR (151 MHz, $CDCl_3$, both rotamers δ 155.5, 147.4, 137.1, 128.6, 128.2, 128.0, 126.8, 123.5, 123.4, 69.0, 68.7, 67.0, 46.1, 45.5, 32.7, 31.8, 16.3, 4.7, 2.2 ppm.

HRMS (ESI-TOF): calc'd for $C_{19}H_{22}NO_2S$ $[M+H]^+$: 328.1371; found 328.1373.

TLC: R_f = 0.3 (8:1 Hexanes:EtOAc).

$[\alpha]_D^{20}$ = -36.7 (c = 1.0, $CHCl_3$).

Chiral HPLC: Chiralpak IA, 4.6 x 250 mm; 10/90 *i*PrOH/hexanes, 1 mL/min, 254 nm; t_R (minor) = 7.5 min, t_R (major) = 7.0 min, >99% *ee*.

Racemic:

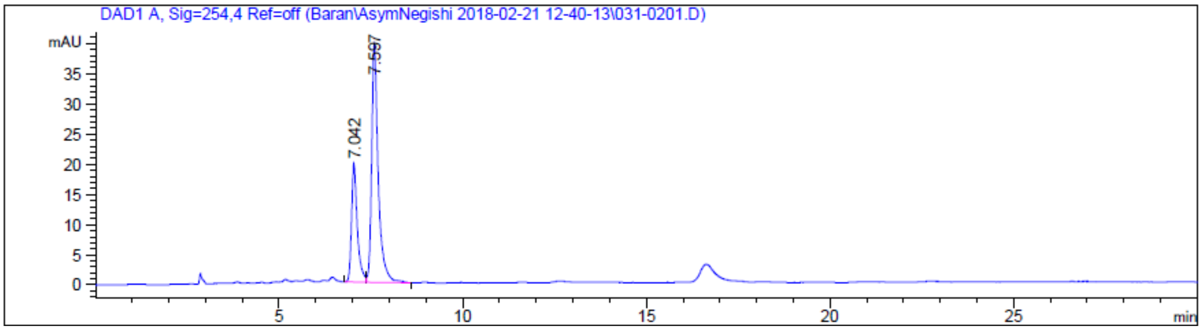

Enantioenriched **42**:

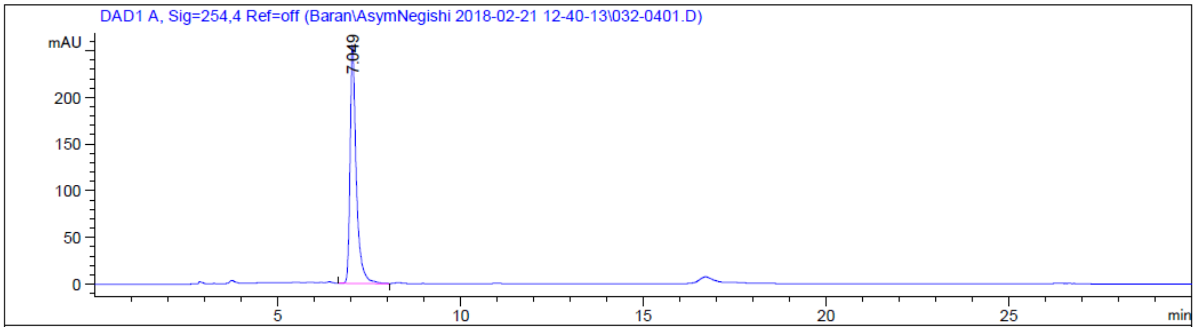

Signal 1: DAD1 A, Sig=254,4 Ref=off

Peak #	RetTime [min]	Type	Width [min]	Area [mAU*s]	Height [mAU]	Area %
1	7.042	BV	0.1649	217.04456	19.64421	30.4580
2	7.597	VB	0.1850	495.55804	39.32985	69.5420

Signal 1: DAD1 A, Sig=254,4 Ref=off

Peak #	RetTime [min]	Type	Width [min]	Area [mAU*s]	Height [mAU]	Area %
1	7.049	BB	0.1764	3023.11938	254.92511	100.0000

Compound 43

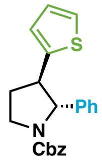

benzyl (2*R*,3*R*)-2-phenyl-3-(thiophen-2-yl)pyrrolidine-1-carboxylate (43)

Following General Procedure **C4** on 0.1 mmol scale with **B11**, $NiCl_2$•glyme (20 mol%), di^{t}BuBipy (25 mol%), and Zinc reagent **SI-35** (2.0 equiv.). Purification by pTLC (silica, 4:1 Hexanes:EtOAc) afforded 30.8 mg (85%) of the title compound **43**.

Physical State: colorless oil

^{1}H NMR (600 MHz, $CDCl_3$, both rotamers): δ 7.41 – 7.24 (m, 5H), 7.23 – 7.12 (m, 5H), 6.92 (dd, J = 5.1, 3.5 Hz, 1H), 6.86 – 6.75 (m, 2H), 5.24 – 4.85 (m, 3H), 4.08 – 3.92 (m, 1H), 3.78 (d, J = 10.3 Hz, 1H), 3.59 (t, J = 6.9 Hz, 1H), 2.45 – 2.32 (m, 1H), 2.19 – 2.08 (m, 1H) ppm.

^{13}C NMR (151 MHz, $CDCl_3$, both rotamers): δ 156.0, 155.1, 154.8, 145.2, 144.9, 142.9, 142.1, 137.0, 136.6, 129.7, 128.6, 128.3, 128.1, 127.6, 127.3, 126.9, 125.9, 124.3, 123.9, 120.6, 115.4, 69.0, 67.0, 66.9, 50.4, 49.3, 47.2, 46.7, 32.9, 32.3 ppm.

HRMS (ESI-TOF): calc'd for $C_{22}H_{22}NO_2S$ $[M+H]^+$: 364.1371; found 364.1369.

TLC: R_f = 0.2 (8:1 Hexanes:EtOAc).

$[\alpha]_D^{20}$ = -8.0 (c = 1.0, $CHCl_3$).

Chiral HPLC: Chiralpak IC, 4.6 x 250 mm; 20/80 *i*PrOH/hexanes, 1 mL/min, 254 nm; t_R (minor) = 10.3 min, t_R (major) = 8.1 min, >99% *ee*.

Racemic:

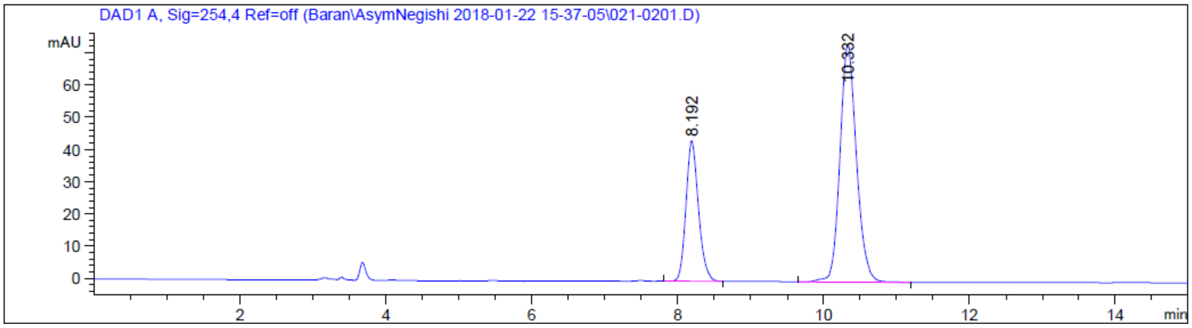

Enantioenriched **43**:

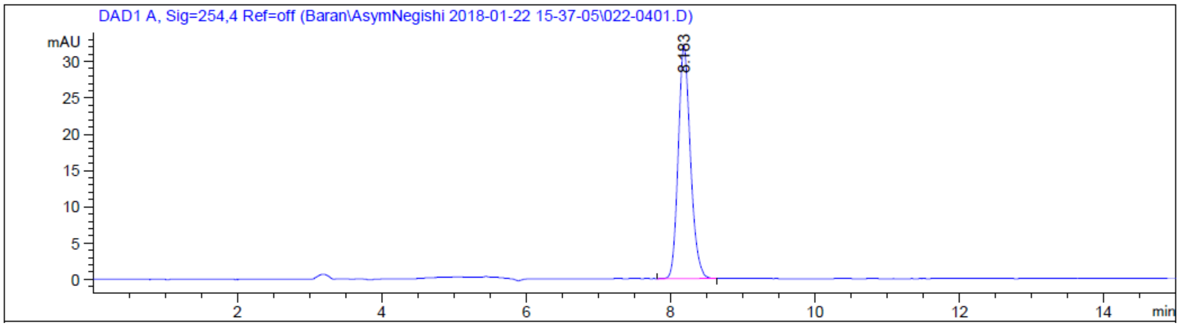

Signal 1: DAD1 A, Sig=254,4 Ref=off

Peak #	RetTime [min]	Type	Width [min]	Area [mAU*s]	Height [mAU]	Area %
1	8.192	BB	0.1841	523.31427	43.59239	31.0356
2	10.332	BB	0.2442	1162.86108	73.64996	68.9644

Signal 1: DAD1 A, Sig=254,4 Ref=off

Peak #	RetTime [min]	Type	Width [min]	Area [mAU*s]	Height [mAU]	Area %
1	8.183	BB	0.1821	380.98270	32.18630	100.0000

Compound 44

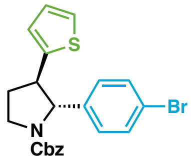

benzyl (2R,3R)-2-(4-bromophenyl)-3-(thiophen-2-yl)pyrrolidine-1-carboxylate (44)

Following General Procedure **C1** on 0.1 mmol scale with **B11**, $NiCl_2 \cdot 6H_2O$ (20 mol%), BPhen (22 mol%), and boronic acid **SI-6** (3.0 equiv.). Purification by pTLC (silica, 5:1 Hexanes:EtOAc) afforded 35.7 mg (81%) of the title compound **44**.

Physical State: colorless oil

^{1}H NMR (600 MHz, $CDCl_3$, both rotamers): δ 7.28 (d, J = 25.2 Hz, 2H), 7.14 – 6.86 (m, 8H), 6.80 – 6.64 (m, 2H), 5.13 – 4.63 (m, 3H), 3.94 – 3.88 (m, 1H), 3.67 (d, J = 9.5 Hz, 1H), 3.46 (d, J = 7.4 Hz, 1H), 2.28 (dtd, J = 13.0, 6.7, 4.8 Hz, 1H), 2.06 – 2.02 (m, 1H) ppm.

^{13}C NMR (151 MHz, $CDCl_3$, both rotamers): δ 163.0, 161.3, 155.0, 144.5, 144.3, 138.6, 137.8, 136.9, 136.4, 128.6, 128.3, 128.1, 127.8, 127.6, 127.5, 127.0, 124.4, 124.0, 115.5, 115.3, 68.5, 67.0, 50.7, 49.6, 47.3, 46.8, 33.1, 32.5, 29.8, 29.8 ppm.

HRMS (ESI-TOF): calc'd for $C_{22}H_{21}BrNO_2S$ $[M+H]^+$: 442.0476; found 442.0478.

TLC: R_f = 0.3 (5:1 Hexanes:EtOAc).

$[\alpha]_D^{20}$ = -5.6 (c = 1.0, $CHCl_3$).

Chiral HPLC: Chiralpak IC, 4.6 x 250 mm; 10/90 *i*PrOH/hexanes, 1 mL/min, 254 nm; t_R (minor) = 12.2 min, t_R (major) = 10.8 min, >99% *ee*.

Racemic:

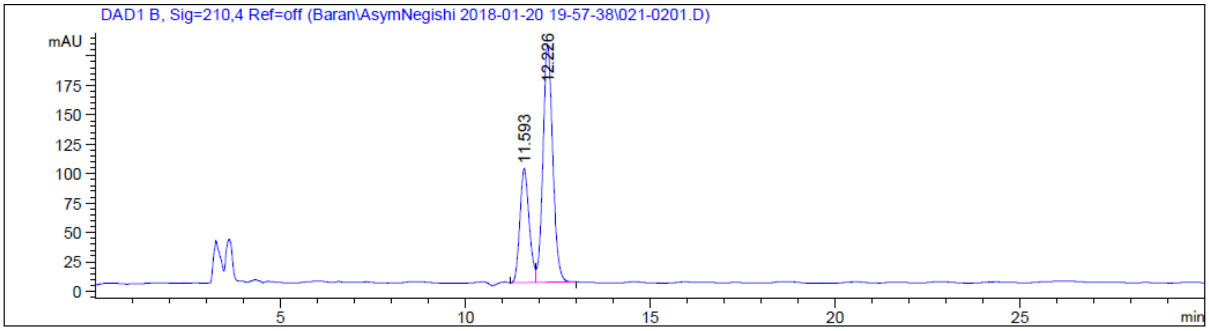

Enantioenriched **44**:

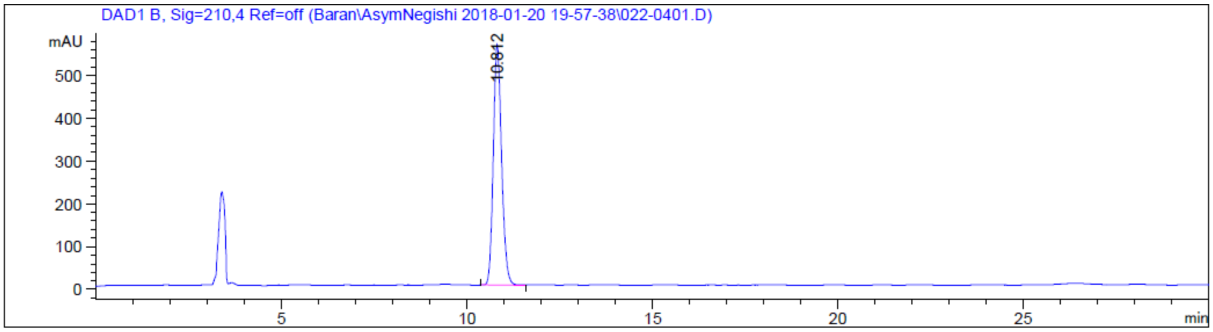

Signal 2: DAD1 B, Sig=210,4 Ref=off

Peak #	RetTime [min]	Type	Width [min]	Area [mAU*s]	Height [mAU]	Area %
1	11.593	BV	0.2695	1684.96509	96.59856	31.2101
2	12.226	VB	0.2829	3713.80835	201.64282	68.7899

Signal 2: DAD1 B, Sig=210,4 Ref=off

Peak #	RetTime [min]	Type	Width [min]	Area [mAU*s]	Height [mAU]	Area %
1	10.812	BB	0.2400	8751.74512	561.01556	100.0000

Compound B15

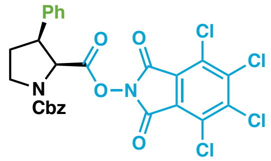

1-benzyl 2-(4,5,6,7-tetrachloro-1,3-dioxoisoindolin-2-yl) (2*S*,3*S*)-3-phenylpyrrolidine-1,2-dicarboxylate (B15)

Following General Procedure **B** on 3.4 mmol scale with **A12**. Purification by flash column chromatography (silica, 4:1 Hexanes:EtOAc) afforded 1.73 g (84%) of the title compound **B15**.

Physical State: yellow oil.

^{1}H NMR (600 MHz, $CDCl_3$, both rotamers): δ 7.49 – 7.28 (m, 10H), 5.54 – 4.88 (m, 3H), 3.99 – 3.92 (m, 1H), 3.88 – 3.82 (m, 1H), 3.68 – 3.59 (m, 1H), 2.74 (p, *J* = 12.9, 11.1 Hz, 1H), 2.34 – 2.29 (m, 1H) ppm.

^{13}C NMR (151 MHz, $CDCl_3$, both rotamers): δ 166.7, 166.6, 156.6, 154.7, 154.1, 141.0, 141.0, 136.6, 136.5, 134.6, 134.5, 130.4, 130.4, 129.0, 128.9, 128.7, 128.6, 128.6, 128.5, 128.3, 128.2, 128.2, 128.2, 124.7, 124.7, 67.8, 67.7, 62.8, 62.7, 48.6, 47.5, 46.3, 46.0, 28.3, 27.3 ppm.

HRMS (ESI-TOF): calc'd for $C_{27}H_{19}Cl_4N_2O_6$ $[M+H]^+$: 606.9997; found 606.9997.

TLC: R_f = 0.3 (2:1 Hexanes:EtOAc).

$[\alpha]_D^{20}$ = 49.1 (*c* = 1.0, $CHCl_3$).

Compound 45

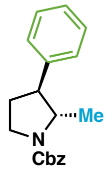

benzyl (2*S*,3*S*)-2-methyl-3-phenylpyrrolidine-1-carboxylate (45)

Following General Procedure **C2** on 0.1 mmol scale with **B15**, $NiCl_2$•glyme (50 mol%), di*t*BuBipy (70 mol%), and Zinc reagent **SI-26** (2.0 equiv.). Purification by pTLC (silica, 4:1 Hexanes:EtOAc) afforded 14.5 mg (49%) of the title compound **45**.

Physical State: colorless oil

^{1}H NMR (600 MHz, $CDCl_3$, both rotamers): δ 7.41 – 7.29 (m, 7H), 7.25 – 7.18 (m, 3H), 5.24 – 5.10 (m, 2H), 3.96 – 3.90 (m, 1H), 3.81 – 3.74 (m, 1H), 3.51 – 3.38 (m, 1H), 2.96 (s, 1H), 2.27 – 2.19 (m, 1H), 1.95 (dd, J = 12.7, 7.6 Hz, 1H), 1.40 – 1.26 (m, 3H) ppm.

^{13}C NMR (151 MHz, $CDCl_3$, both rotamers): δ 155.2, 154.8, 142.4, 142.1, 137.2, 137.1, 128.8, 128.6, 128.6, 128.0, 128.0, 127.4, 126.9, 67.0, 66.7, 60.4, 60.1, 53.2, 52.5, 46.4, 46.1, 32.8, 32.1, 20.6, 19.4 ppm.

TLC: R_f = 0.3 (4:1 Hexanes:Acetone).

HRMS (ESI-TOF): calc'd for $C_{19}H_{22}NO_2$ $[M+H]^+$: 296.1651; found 296.1649.

$[\alpha]_D^{20}$ = -9.8 (c = 0.5, $CHCl_3$).

Experimental Procedures and Characterization Data for Tetrahydrofuran Scaffolds (Figure 3 – c)

Compound SI-39:

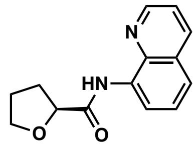

(S)-N-(quinolin-8-yl) tetrahydrofuran-2-carboxamide (SI-39)

Following General Procedure **A3.1** on 15 mmol scale. Purification by column chromatography (gradient from 9:1 to 4:1Hexanes:EtOAc) afforded 3 g (83%) of the title compound **SI-39**.

Spectroscopic data are in accordance with those reported in the literature.[5]

Compound SI-40:

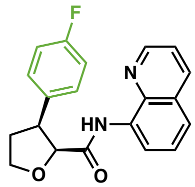

(2S,3S)-3-(4-fluorophenyl)-N-(quinolin-8-yl) tetrahydrofuran-2-carboxamide (SI-40)

Following General Procedure **A3.2** on 8.26 mmol scale with **SI-39**. Purification by flash column chromatography (gradient from 9:1 to 7:3 Hexanes:EtOAc), afforded 1.75 g (63%) of the title compound **SI-40**.

Physical State: white solid.

m.p.: 120-121 °C.

^{1}H NMR (400 MHz, $CDCl_3$): δ 10.55 (s, 1H), 8.87 – 8.84 (m, 1H), 8.43 (d, J = 7.4 Hz, 1H), 8.12 (d, J = 8.3 Hz, 1H), 7.50 – 7.38 (m, 3H), 7.29 – 7.19 (m, 2H), 6.80 (t, J = 8.7 Hz, 2H), 4.72 (d, J = 7.0 Hz, 1H), 4.58 (td, J = 8.5, 5.6 Hz, 1H), 4.18 (td, J = 8.8, 6.6 Hz, 1H), 3.88 (h, J = 4.5 Hz, 1H), 2.62 – 2.51 (m, 1H), 2.31 – 2.21 (m, 1H) ppm.

^{19}F NMR (376 MHz, $CDCl_3$): δ -116.56 ppm.

^{13}C NMR (151 MHz, $CDCl_3$): δ 168.3, 161.7 (d, J_{C-F} = 244.9 Hz), 148.6, 138.9, 136.4 (d, J_{C-F} = 3.2 Hz), 136.2, 133.6, 129.5 (d, J_{C-F} = 7.7 Hz), 128.0, 127.2, 122.0, 121.6, 116.7, 115.12 (d, J_{C-F} = 20.9 Hz), 83.9, 68.7, 47.3, 33.8 ppm.

HRMS (ESI-TOF): calc'd for $C_{20}H_{18}FN_2O_2$ $[M+H]^+$:337.1352, found: 337.1353.

TLC: R_f = 0.25 (4:1Hexanes:EtOAc).

$[\alpha]_D^{20}$ = +72.94 (c = 0.17, $CHCl_3$).

Compound SI-41:

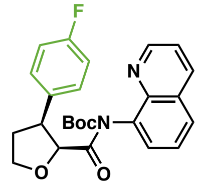

tert-butyl ((2S,3S)-3-(4-fluorophenyl)tetrahydrofuran-2-carbonyl)(quinolin-8-yl)carbamate (SI-41)

Following General Procedure **A.3.3.1** on 1.48 mmol scale with **SI-40**. Purification by flash column chromatography (silica, 1:1 Hexanes:EtOAc) afforded 0.53 g (83%) of the title compound **SI-41**.

Physical State: yellow foam.

^{1}H NMR (400 MHz, $CDCl_3$): δ 8.81 (s, 1H), 8.10 (d, J = 8.2 Hz, 1H), 7.73 (d, J = 8.2 Hz, 1H), 7.43 – 7.32 (m, 5H), 7.06 (t, J = 8.5 Hz, 2H), 6.10 (bs, 1H), 4.44 (td, J = 8.2, 4.5 Hz, 1H), 4.13 (q, J = 8.5 Hz, 1H), 4.02 (q, J = 7.8 Hz, 1H), 2.63 – 2.45 (m, 1H), 2.34 – 2.15 (m, 1H), 1.12 (s, 9H) ppm.

^{19}F NMR (376 MHz, $CDCl_3$): δ -116.36 ppm.

^{13}C NMR (151 MHz, $CDCl_3$): δ 173.5, 161.9 (d, $J_{C\text{-}F}$ = 244.9 Hz), 152.0, 150.1, 143.9, 135.8 (d, $J_{C\text{-}F}$ = 9.9 Hz), 130.5, 128.6, 128.3, 127.9, 125.8, 121.3, 115.0 (d, $J_{C\text{-}F}$ = 21.5 Hz), 82.4, 82.4, 68.5, 47.2, 34.9, 27.5 ppm.

HRMS (ESI-TOF): calc'd for $C_{25}H_{26}FN_2O_4$ $[M+H]^+$: 437.1877, found: 437.1877.

TLC: R_f = 0.26 (1:1 Hexanes:EtOAc).

$[\alpha]_D^{20}$ = 4.3 (c = 0.94, $CHCl_3$).

Compound A9:

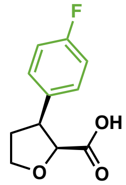

(2S,3S)-3-(4-fluorophenyl)tetrahydrofuran-2-carboxylic acid (A9)

Following General Procedure **A3.3.2** on 4.14 mmol scale with **SI-41** affording 0.714 g (82%) of the title compound **A9**. The crude reaction was used for the subsequent step without further purification,

Physical State: light-yellow solid.

m.p.: 161-163 °C.

^{1}H NMR (600 MHz, $CDCl_3$): δ 7.14 (dd, *J* = 8.7, 5.3 Hz, 2H), 6.95 (t, *J* = 8.7 Hz, 2H), 4.59 (d, *J* = 7.7 Hz, 1H), 4.35 (td, *J* = 8.5, 4.7 Hz, 1H), 4.02 (q, *J* = 8.0 Hz, 1H), 3.72 (q, *J* = 7.6 Hz, 1H), 2.40 (ddt, *J* = 12.6, 7.8, 3.9 Hz, 1H), 2.25 (dq, *J* = 12.6, 7.6 Hz, 1H) ppm.

^{19}F NMR (376 MHz, MEOD): δ -118.66 ppm.

^{13}C NMR (151 MHz, $CDCl_3$): δ 175.1, 162.1 (d, $J_{C\text{-}F}$ = 245.4 Hz), 134.5 (d, $J_{C\text{-}F}$ = 3.2 Hz), 129.5 (d, $J_{C\text{-}F}$ = 8.2 Hz), 115.4 (d, $J_{C\text{-}F}$ = 20.9 Hz), 81.4, 68.9, 47.4, 32.4 ppm.

HRMS (ESI-TOF): calc'd for $C_{11}H_{10}FO_3$ [M-H]: 209.0614, found: 209.0614.

TLC: R_f = 0.16 (9:1 CH_2Cl_2:MeOH).

$[\alpha]_D^{20}$ = +25.52 (*c* = 0.38, $CHCl_3$).

Compound B12:

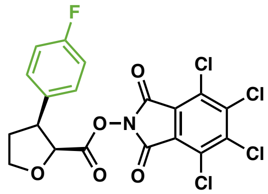

4,5,6,7-tetrachloro-1,3-dioxoisoindolin-2-yl (2S,3S)-3-(4-fluorophenyl)tetrahydrofuran-2-carboxylate (B12)

Following General Procedure **B** on 1.90 mmol scale with **A9**. Purification by flash column chromatography (silica, 100% DCM), followed by recrystallization in CH_2Cl_2 afforded 791 mg (85%) of the title compound **B12**.

Physical State: white solid.

m.p.: 164-165 °C.

^{1}H NMR (400 MHz, $CDCl_3$): δ 7.35 – 7.29 (m, 2H), 7.09 – 7.02 (m, 2H), 5.02 (d, J = 7.7 Hz, 1H), 4.45 (td, J = 8.4, 4.1 Hz, 1H), 4.15 (q, J = 8.2 Hz, 1H), 3.93 – 3.85 (m, 1H), 2.57 – 2.41 (m, 2H) ppm.

^{19}F NMR (376 MHz, $CDCl_3$): δ -115.05 ppm.

^{13}C NMR (151 MHz, $CDCl_3$): δ 166.8, 162.5 (d, J_{C-F} = 243.2 Hz), 141.1, 132.0 (d, J_{C-F} = 2.75 Hz), 130.5, 129.9 (d, J_{C-F} = 8.3 Hz), 124.7, 115.9 (d, J_{C-F} = 2.1 Hz), 80.2, 69.4, 48.2, 31.1 ppm.

HRMS (ESI-TOF): calc'd for $C_{19}H_{11}Cl_4FNO5$ $[M+H]^+$: 491.9375, found: 491.9373.

TLC: R_f = 0. 27 (3:7 Hexanes:CH_2Cl_2).

$[\alpha]_D^{20}$ = - 1.33 (c = 0.15, $CHCl_3$).

Chiral HPLC: Chiralpak IA, 4.6 x 250 mm; 45/55 *i*PrOH/hexanes, 1 mL/min, 254 nm; t_R (minor) = 33.0 min, t_R (major) = 42.2 min, 93% *ee*.

Racemic:

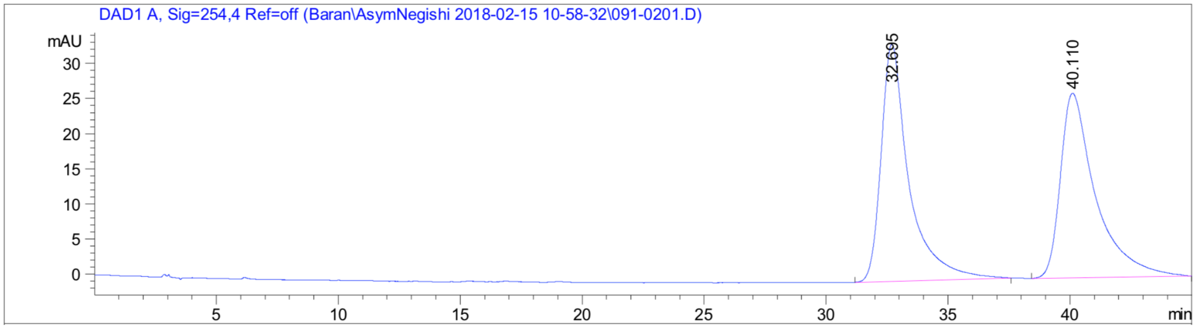

Enantioenriched **B12**:

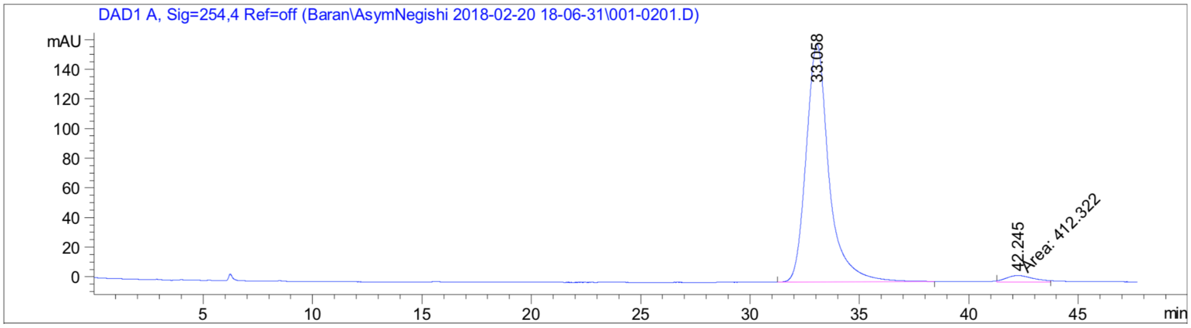

Signal 1: DAD1 A, Sig=254,4 Ref=off

Peak #	RetTime [min]	Type	Width [min]	Area [mAU*s]	Height [mAU]	Area %
1	32.695	BB	1.1441	2686.05127	33.76443	50.4350
2	40.110	BBA	1.3815	2639.72168	26.33289	49.5650

Signal 1: DAD1 A, Sig=254,4 Ref=off

Peak #	RetTime [min]	Type	Width [min]	Area [mAU*s]	Height [mAU]	Area %
1	33.058	BB	1.0974	1.16630e4	160.19925	96.5854
2	42.245	MM	1.5166	412.32156	4.53128	3.4146

Compound 46

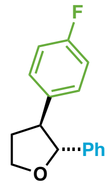

(2*R*,3*S*)-3-(4-fluorophenyl)-2-phenyltetrahydrofuran (46)

Following General Procedure **C4** on 0.05 mmol scale with **B12**, $NiCl_2$•glyme (20 mol%), di*t*BuBipy (40 mol%), and Zinc reagent **SI-35** (3.0 equiv.). Purification by pTLC (silica, 9:1 Hexanes:EtOAc) afforded 8.8 mg (73%) of the title compound **46**.

Physical State: colorless oil

R_f = 0.4 (8:1 hexanes:EtOAc).

^{1}H NMR (600 MHz, $CDCl_3$) δ 7.32 – 7.25 (m, 3H), 7.20 (d, *J* = 6.9 Hz, 2H), 7.16 (dd, *J* = 8.5, 5.5 Hz, 2H), 7.01 (t, *J* = 8.7 Hz, 2H), 4.79 (d, *J* = 8.5 Hz, 1H), 4.31 – 4.25 (m, 2H), 3.22 (q, *J* = 8.5 Hz, 1H), 2.54 – 2.48 (m, 1H), 2.31 – 2.25 (m, 1H) ppm.

^{19}F NMR (376 MHz, $CDCl_3$): δ -104.79 ppm.

^{13}C NMR (151 MHz, $CDCl_3$,) δ 161.8 (d, $J_{C\text{-}F}$ = 244.9 Hz), 141.5, 136.6 (d, $J_{C\text{-}F}$ = 3.3 Hz), 129.3 (d, $J_{C\text{-}F}$ = 8.0 Hz), 128.4, 127.6, 125.9, 115.5 (d, $J_{C\text{-}F}$ = 21.1 Hz), 87.8, 68.6, 53.7, 35.6 ppm.

TLC: R_f = 0.47 (4:1 Hexanes: EtOAc).

$[\alpha]_D^{20}$ = -5.00 (*c* = 0.1, $CHCl_3$).

Chiral HPLC: Chiralpak IC, 4.6 x 250 mm; 5/95 *i*PrOH/hexanes, 0.5 mL/min, 254 nm; t_R (minor) = 11.8 min, t_R (major) = 13.4 min, 96% *ee*.

*Note: no HRMS could be recorded for **46***

Racemic:

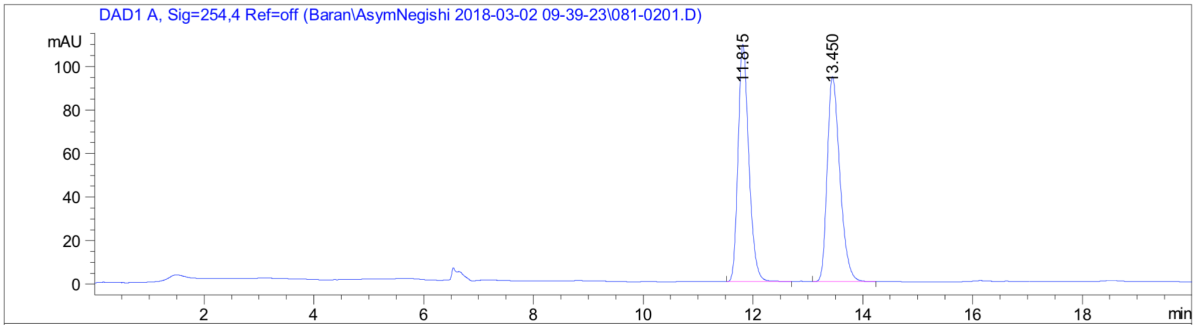

Enantioenriched **46**:

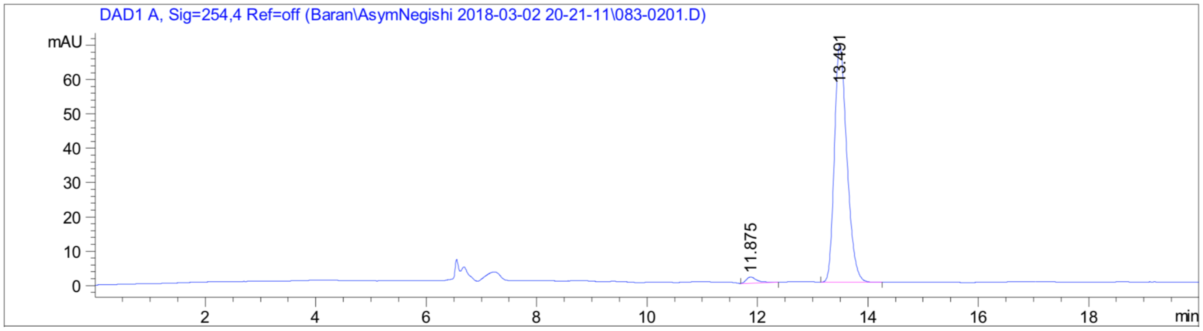

Signal 1: DAD1 A, Sig=254,4 Ref=off

Peak #	RetTime [min]	Type	Width [min]	Area [mAU*s]	Height [mAU]	Area %
1	11.815	BB	0.2094	1486.25330	108.79140	49.6154
2	13.450	BB	0.2451	1509.29260	94.13255	50.3846

Signal 1: DAD1 A, Sig=254,4 Ref=off

Peak #	RetTime [min]	Type	Width [min]	Area [mAU*s]	Height [mAU]	Area %
1	11.875	BB	0.2000	23.21353	1.75830	2.0851
2	13.491	BB	0.2418	1090.11267	69.17828	97.9149

Compound 47

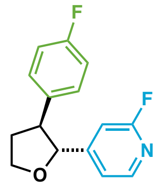

2-fluoro-4-((2*R*,3*S*)-3-(4-fluorophenyl)tetrahydrofuran-2-yl)pyridine (47)

Following General Procedure **C1** on 0.1 mmol scale with **B12**, $NiCl_2$•$6H_2O$ (40 mol%), BPhen (45 mol%), and boronic acid **SI-17** (3.0 equiv.). Purification by pTLC (silica, 4:1 Hexanes:EtOAc) afforded 24.1 mg (81%) of the title compound **47**.

Physical State: colorless oil

^{1}H NMR (600 MHz, $CDCl_3$,) δ 7.95 (d, J = 2.4 Hz, 1H), 7.60 (td, J = 8.1, 2.5 Hz, 1H), 7.11 (dd, J = 8.5, 5.4 Hz, 2H), 7.00 (t, J = 8.6 Hz, 2H), 6.85 (dd, J = 8.5, 2.9 Hz, 1H), 4.75 (d, J = 8.9 Hz, 1H), 4.25 (dd, J = 8.4, 5.7 Hz, 2H), 3.10 (q, J = 8.7 Hz, 1H), 2.51 (ddt, J = 13.4, 8.0, 5.7 Hz, 1H), 2.33 – 2.26 (m, 1H) ppm.

^{19}F NMR (400 MHz, $CDCl_3$): δ -69.91, -115.59 ppm.

^{13}C NMR (151 MHz, $CDCl_3$) δ 163.38 (d, $J_{C\text{-}F}$ = 238.6 Hz), 162.07 (d, $J_{C\text{-}F}$ = 246.1 Hz), 145.43 (d, $J_{C\text{-}F}$ = 14.8 Hz), 138.85 (d, $J_{C\text{-}F}$ = 8.1 Hz), 135.15 (d, $J_{C\text{-}F}$ = 3.3 Hz), 134.52 (d, $J_{C\text{-}F}$ = 4.4 Hz), 129.23 (d, $J_{C\text{-}F}$ = 7.9 Hz), 115.91 (d, $J_{C\text{-}F}$ = 21.3 Hz), 109.35 (d, $J_{C\text{-}F}$ = 37.4 Hz), 85.10, 68.65, 54.03, 35.57 ppm.

HRMS (ESI-TOF): calc'd for $C_{15}H_{14}F_2NO$ $[M+H]^+$: 262.1043; found 262.1047.

TLC: R_f = 0.2 (4:1 Hexanes:EtOAc).

$[\alpha]_D^{20}$ = -75.3 (c = 0.5, $CHCl_3$).

Chiral HPLC: Chiralpak IC, 4.6 x 250 mm; 20/80 *i*PrOH/hexanes, 1 mL/min, 210 nm; t_R (minor) = 8.5 min, t_R (major) = 15.0 min, 93% *ee*.

Racemic:

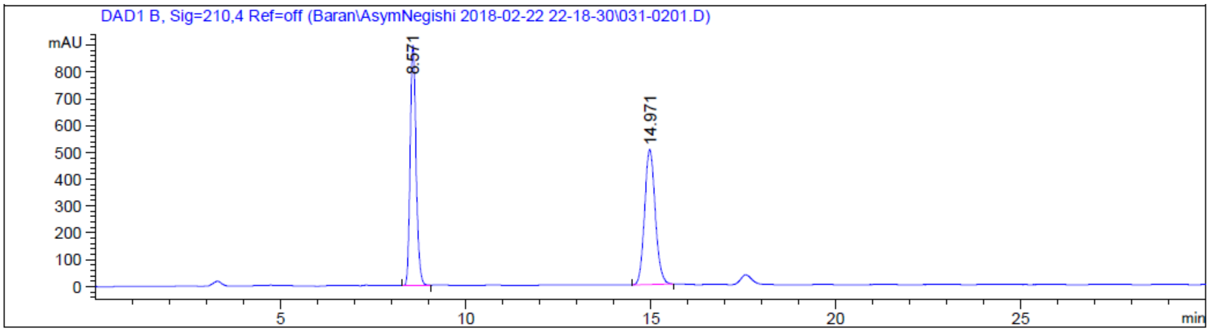

Enantioenriched **47**:

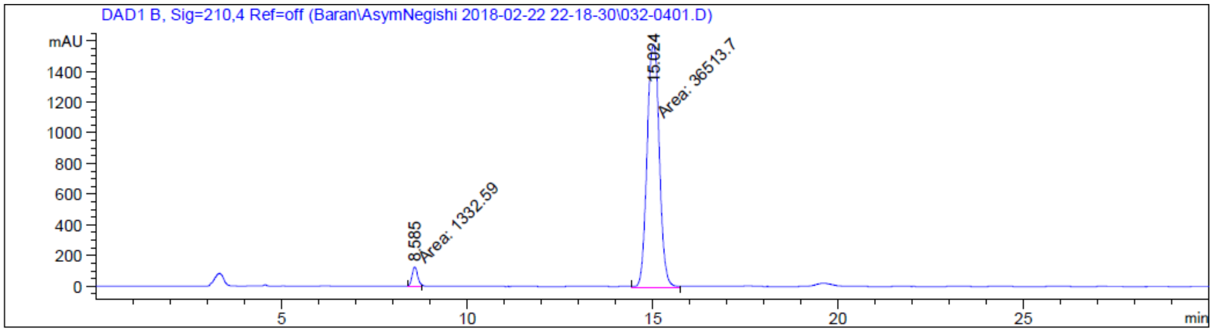

Signal 2: DAD1 B, Sig=210,4 Ref=off

Peak #	RetTime [min]	Type	Width [min]	Area [mAU*s]	Height [mAU]	Area %
1	8.571	BB	0.1713	9896.48340	892.95050	49.5795
2	14.971	BB	0.3118	1.00644e4	502.20361	50.4205

Signal 2: DAD1 B, Sig=210,4 Ref=off

Peak #	RetTime [min]	Type	Width [min]	Area [mAU*s]	Height [mAU]	Area %
1	8.585	MM	0.1740	1332.59424	127.62409	3.5211
2	15.024	MM	0.3852	3.65137e4	1579.67749	96.4789

Compound 48

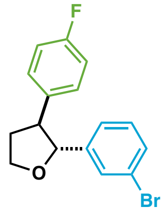

(2*R*,3*S*)-2-(3-bromophenyl)-3-(4-fluorophenyl)tetrahydrofuran (48)

Following General Procedure **C1** on 0.1 mmol scale with **B12**, $NiCl_2$•$6H_2O$ (40 mol%), BPhen (45 mol%), and boronic acid **SI-9** (3.0 equiv.). Purification by pTLC (silica, 4:1 Hexanes:EtOAc) afforded 30.0 mg (94%) of the title compound **48**.

Physical State: colorless oil

^{1}H NMR (600 MHz, $CDCl_3$, both rotamers) δ 7.40 (s, 1H), 7.37 (d, *J* = 8.0 Hz, 1H), 7.15 – 7.08 (m, 3H), 7.03 – 6.97 (m, 3H), 4.73 (d, *J* = 8.4 Hz, 1H), 4.24 (dd, *J* = 8.3, 5.7 Hz, 2H), 3.14 (q, *J* = 8.6 Hz, 1H), 2.47 (tt, *J* = 12.9, 5.7 Hz, 1H), 2.30 – 2.20 (m, 1H) ppm.

^{19}F NMR (400 MHz, $CDCl_3$): δ -116.11 ppm.

^{13}C NMR (151 MHz, $CDCl_3$, both rotamers) δ 161.94 (d, $J_{C\text{-}F}$ = 245.1 Hz), 144.04, 136.10 (d, $J_{C\text{-}F}$ = 3.1 Hz), 130.71, 129.90, 129.25 (d, $J_{C\text{-}F}$ = 7.8 Hz), 128.76, 124.64, 122.68, 115.70 (d, $J_{C\text{-}F}$ = 21.4 Hz), 87.10, 68.70, 53.91, 35.62 ppm.

HRMS (ESI-TOF): calc'd for $C_{16}H_{15}BrFO$ $[M+H]^+$: 321.0290; found 321.0296.

TLC: R_f = 0.4 (8:1 Hexanes:EtOAc).

$[\alpha]_D^{20}$ = -108.0 (*c* = 1.0, $CHCl_3$).

Chiral HPLC: Chiralpak IC, 4.6 x 250 mm; 5/95 *i*PrOH/hexanes, 0.5 mL/min, 254 nm; t_R (minor) = 9.8 min, t_R (major) = 11.4 min, 94% *ee*.

Racemic:

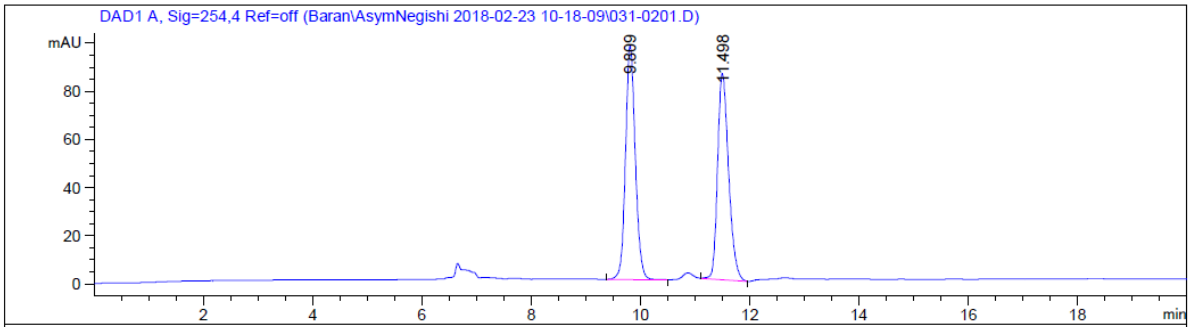

Enantioenriched **48**:

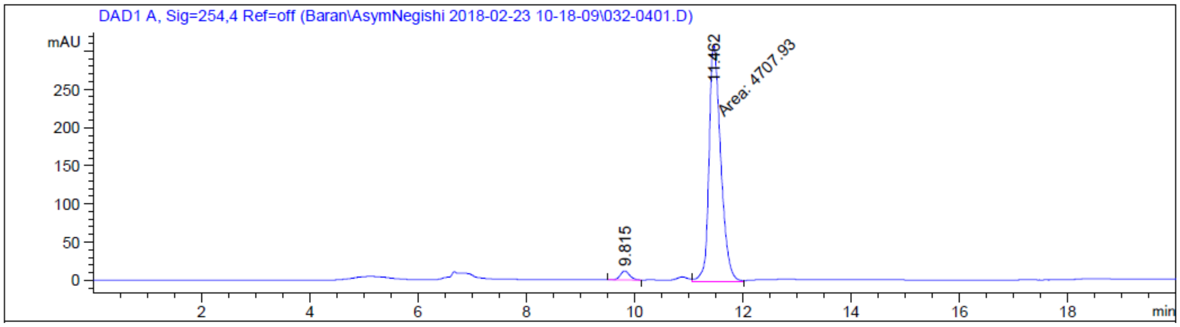

Signal 1: DAD1 A, Sig=254,4 Ref=off

Peak #	RetTime [min]	Type	Width [min]	Area [mAU*s]	Height [mAU]	Area %
1	9.809	BB	0.1886	1205.42395	97.25366	50.1686
2	11.498	BB	0.2147	1197.31995	85.84238	49.8314

Signal 1: DAD1 A, Sig=254,4 Ref=off

Peak #	RetTime [min]	Type	Width [min]	Area [mAU*s]	Height [mAU]	Area %
1	9.815	BB	0.1826	138.62724	11.67001	2.8603
2	11.462	MM	0.2527	4707.92871	310.55161	97.1397

Compound 49

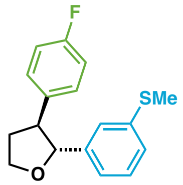

(2R,3S)-3-(4-fluorophenyl)-2-(3-(methylthio)phenyl)tetrahydrofuran (49)

Following General Procedure **C1** on 0.1 mmol scale with **B12**, $NiCl_2•6H_2O$ (40 mol%), BPhen (40 mol%), and boronic acid **SI-8** (3.0 equiv.). Purification by pTLC (silica, 9:1 Hexanes:EtOAc) afforded 26.5 mg (92%) of the title compound **49**.

Physical State: colorless oil.

^{1}H NMR (400 MHz, $CDCl_3$): δ 7.21 – 7.05 (m, 5H), 6.99 (t, J = 8.7 Hz, 2H), 6.92 – 6.86 (m, 1H), 4.74 (d, J = 8.4 Hz, 1H), 4.30 – 4.19 (m, 2H), 3.21 – 3.11 (m, 1H), 2.53 – 2.37 (m, 4H), 2.25 (ddt, J = 12.5, 9.6, 8.2 Hz, 1H) ppm.

^{19}F NMR (376 MHz, $CDCl_3$): δ -116.33 ppm.

^{13}C NMR (126 MHz, $CDCl_3$): δ 161.9 (d, J_{C-F} = 243.0 Hz), 142.3, 138.5, 136.5 (d, J_{C-F} = 2.9 Hz), 129.3 (d, J_{C-F} = 7.6 Hz), 128.8, 125.8, 123.8, 122.7, 115.6 (d, J_{C-F} = 21.5 Hz), 87.6, 68.6, 53.7, 35.6, 15.8 ppm.

HRMS (ESI-TOF): calc'd for $C_{17}H_{18}FOS$ $[M+H]^+$: 289.1062, found: 289.1056.

TLC: R_f = 0.26 (9:1 Hexanes:EtOAc).

$[\alpha]_D^{20}$ = -69.10 (c = 1, $CHCl_3$).

Chiral SFC: IG column (3 µm, 4.6x250 mm) under isocratic conditions [3% MeOH / CO_2 (4 mL/min), 1600 psi backpressure] at 30 °C. The enantiomers were detected by UV light (260 nm). t_R (minor) = 2.06 min, t_R (major) = 2.25 min, 93.5 % *ee*.

Racemic**:**

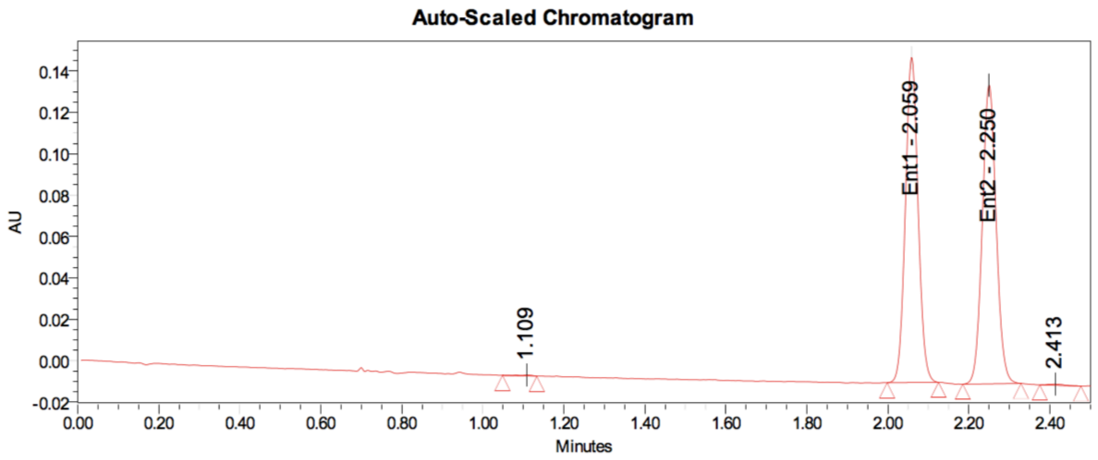

Enantioenriched **49**:

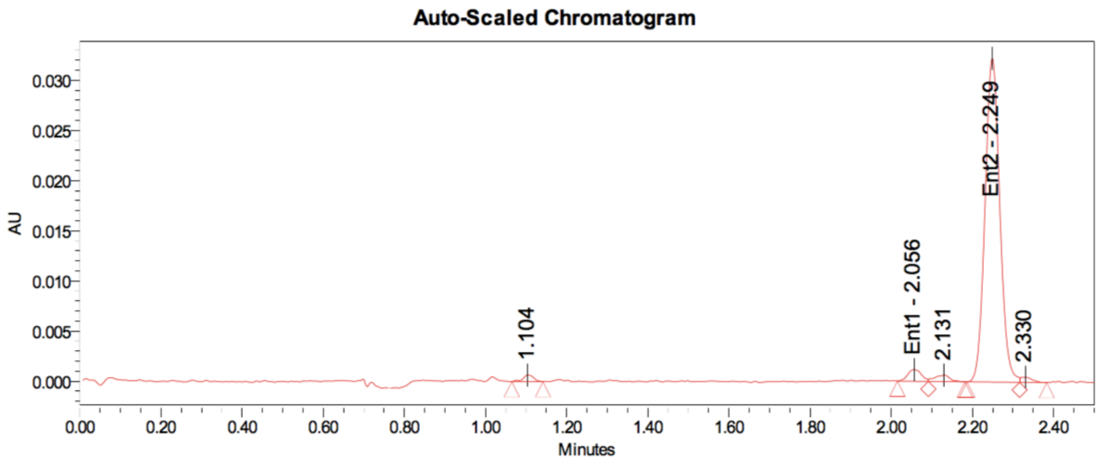

KSF-465	49.87	50.13	-0.27	342386	344211
KSF-469	3.25	96.75	-93.50	2741	81639

Compound 50

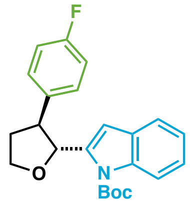

***tert*-butyl 2-((2*R*,3*S*)-3-(4-fluorophenyl)tetrahydrofuran-2-yl)-1*H*-indole-1-carboxylate (50)**

Following General Procedure **C1** on 0.1 mmol scale with **B12**, $NiCl_2$•$6H_2O$ (40 mol%), BPhen (40 mol%), and boronic acid **SI-19** (5.0 equiv.). Purification by pTLC (silica, 9:1 Hexanes:EtOAc) afforded 8.1 mg (22%) of the title compound **50**.

Physical State: sticky oil colorless.

^{1}H NMR (600 MHz, $CDCl_3$): δ 8.08 (d, J = 8.3 Hz, 1H), 7.53 – 7.50 (m, 1H), 7.31 (dd, J = 8.7, 5.3 Hz, 2H), 7.26 (dd, J = 15.5, 1.4 Hz, 1H), 7.21 (td, J = 7.5, 1.2 Hz, 1H), 7.03 (t, J = 8.7 Hz, 2H), 6.69 – 6.65 (m, 1H), 5.79 (d, J = 2.6 Hz, 1H), 4.27 (td, J = 8.5, 4.2 Hz, 1H), 4.16 (q, J = 8.2 Hz, 1H), 3.50 (dt, J = 7.4, 3.7 Hz, 1H), 2.41 (dq, J = 12.5, 8.3 Hz, 1H), 1.93 (ddt, J = 11.4, 7.6, 4.1 Hz, 1H), 1.51 (s, 9H) ppm.

^{19}F NMR (376 MHz, $CDCl_3$): δ -116.97 ppm.

^{13}C NMR (151 MHz, $CDCl_3$): δ 161.8 (d, $J_{C\text{-}F}$ = 244.3 Hz), 150.4, 142.2, 139.52 (d, $J_{C\text{-}F}$ = 3.2 Hz), 137.25, 129.0, 128.9 (d, $J_{C\text{-}F}$ = 7.8 Hz), 124.1, 122.9, 120.6, 115.73 – 115.43 (m), 107.5, 84.4, 81.6, 67.8, 50.3, 33.5, 28.2 ppm.

HRMS (ESI-TOF): calc'd for $C_{23}H_{25}FNO_3$ $[M+H]^+$: 382.1818, found: 382.1817.

TLC: R_f = 0.42 (9:1 Hexanes:EtOAc).

$[\alpha]_D^{20}$ = - 23.91 (c = 0.23, $CHCl_3$).

Chiral SFC: IG column (3 µm, 4.6x250 mm) under isocratic conditions [3% MeOH / CO_2 (4 mL/min), 1600 psi backpressure] at 30 °C. The enantiomers were detected by UV light (260 nm). t_R (minor) = 1.59 min, t_R (major) = 1.79 min, 91.65 % *ee*.

Racemic:

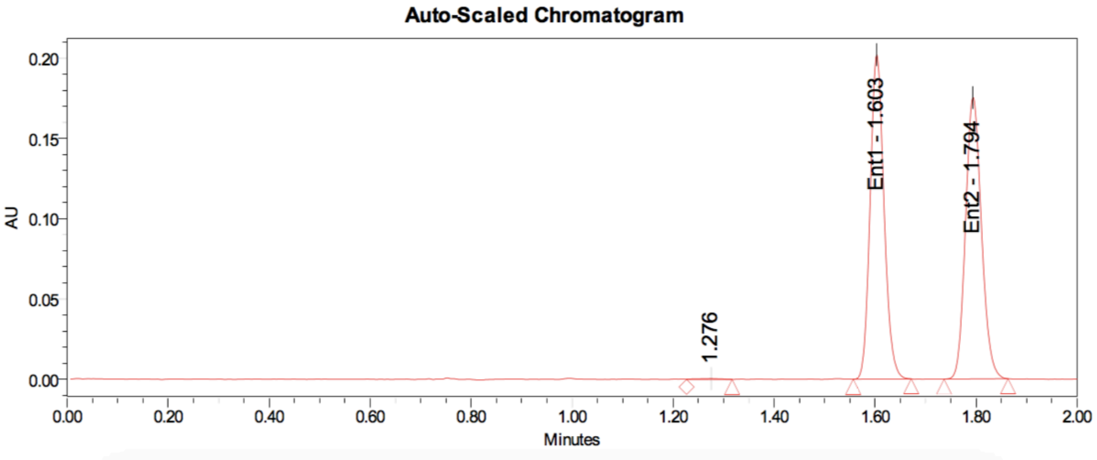

Enantioenriched **50:**

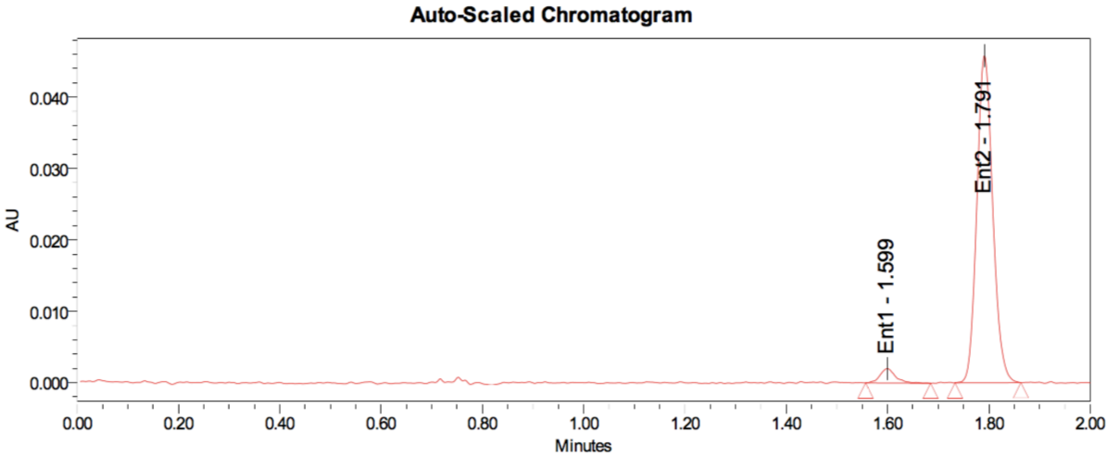

KSF-466	50.06	49.94	0.12	385889	384972
KSF-467	4.17	95.83	-91.65	4364	100179

Compound 51

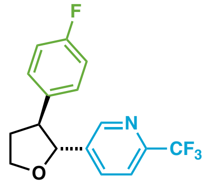

5-((2R,3S)-3-(4-fluorophenyl)tetrahydrofuran-2-yl)-2-(trifluoromethyl)pyridine (51)

Following General Procedure **C1** on 0.1 mmol scale with **B12**, $NiCl_2$•$6H_2O$ (40 mol%), BPhen (40 mol%), and boronic acid **SI-14** (3.0 equiv.). Purification by pTLC (silica, 4:1 Hexanes:EtOAc) afforded 14.4 mg (46%) of the title compound **51**.

Physical State: colorless oil.

^{1}H NMR (600 MHz, $CDCl_3$): δ 8.44 (d, J = 2.6 Hz, 1H), 7.66 (ddd, J = 8.1, 2.9, 0.7 Hz, 1H), 7.59 (dd, J = 8.1, 1.0 Hz, 1H), 7.17 – 7.11 (m, 2H), 7.05 – 6.99 (m, 2H), 4.86 (d, J = 8.9 Hz, 1H), 4.32 – 4.24 (m, 2H), 3.12 (dt, J = 9.8, 8.0 Hz, 1H), 2.52 (dddd, J = 12.5, 8.0, 6.7, 4.5 Hz, 1H), 2.31 (ddt, J = 12.6, 9.9, 8.3 Hz, 1H) ppm.

^{19}F NMR (376 MHz, $CDCl_3$): δ -68.04, -115.30 ppm.

^{13}C NMR (151 MHz, $CDCl_3$): δ 162.2 (d, $J_{C\text{-}F}$ = 246.0 Hz), 147.8, 140.4, 135.0 (d, $J_{C\text{-}F}$ = 3.2 Hz), 134.6, 129.3 (d, $J_{C\text{-}F}$ = 8.0 Hz), 120.2 (q, $J_{C\text{-}F}$ = 2.6 Hz), 116.1 (d, $J_{C\text{-}F}$ = 21.4 Hz), 85.1, 68.9, 54.2, 35.9 ppm.

HRMS (ESI-TOF): calc'd for $C_{16}H_{14}F_4NO$ $[M+H]^+$: 312.1012, found: 312.1017.

TLC: R_f = 0.41 (4:1 Hexanes:EtOAc).

$[\alpha]_D^{20}$ = -63.306 (c = 1.24, $CHCl_3$)

Chiral SFC: IG column (3 µm, 4.6x250 mm) under isocratic conditions [3% MeOH / CO_2 (4 mL/min), 1600 psi backpressure] at 30 °C. The enantiomers were detected by UV light (260 nm). t_R (minor) = 1.27 min, t_R (major) = 1.41 min, 92.7 % *ee*.

Racemic:

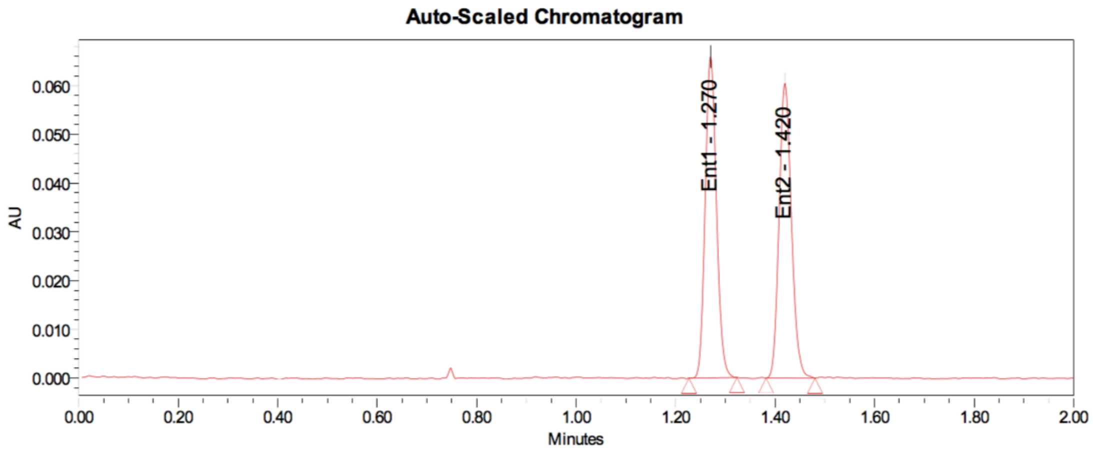

Enantioenriched **51:**

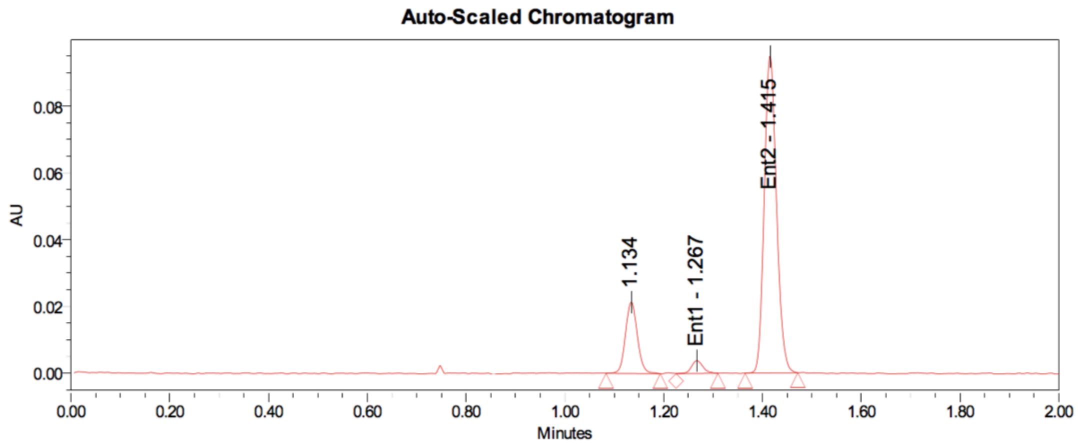

KSF-464	49.90	50.10	-0.19	103514	103919
KSF-468	3.66	96.34	-92.68	6208	163408

Compound 52

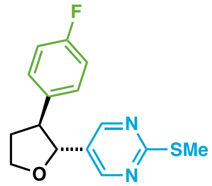

5-((2*R*,3*S*)-3-(4-fluorophenyl)tetrahydrofuran-2-yl)-2-(methylthio)pyrimidine (52)

Following General Procedure **C1** on 0.1 mmol scale with **B12**, $NiCl_2$•$6H_2O$ (40 mol%), BPhen (40 mol%), and boronic acid **SI-18** (3.0 equiv.). Purification by pTLC (silica, 6:1 Hexanes:EtOAc) afforded 19.3 mg (67%) of the title compound **52**.

Physical State: colorless oil.

^{1}H NMR (600 MHz, $CDCl_3$): δ 8.30 (s, 2H), 7.16 – 7.11 (m, 2H), 7.04 – 7.00 (m, 2H), 4.68 (d, J = 9.0 Hz, 1H), 4.28 – 4.21 (m, 2H), 3.11 (q, J = 9.1 Hz, 1H), 2.57 – 2.49 (m, 4H), 2.34 – 2.25 (m, 1H) ppm.

^{19}F NMR (376 MHz, $CDCl_3$): δ -115.60 ppm.

^{13}C NMR (151 MHz, $CDCl_3$): δ 172.2, 162.1 (d, J_{C-F} = 245.9 Hz), 155.2, 134.7 (d, J_{C-F} = 3.3 Hz), 129.3 (d, J_{C-F} = 8.2 Hz), 129.1, 116.1 (d, J_{C-F} = 21.5 Hz), 83.7 (d, J_{C-F} = 0.8 Hz), 68.7, 53.8, 35.6, 14.2 ppm.

HRMS (ESI-TOF): calc'd for $C_{15}H_{16}FN_2OS$ $[M+H]^+$: 291.0967, found: 291.0970

TLC: R_f = 0.21 (6:1 Hexanes:EtOAc).

$[\alpha]_D^{20}$ = -160.00 (c = 0.15, $CHCl_3$).

Chiral HPLC: Chiralpak IC, 4.6 x 250 mm; 30/70 *i*PrOH/hexanes, 1 mL/min, 254 nm; t_R (minor) = 11.74 min, t_R (major) = 17.22 min, 93% *ee*.

Racemic:

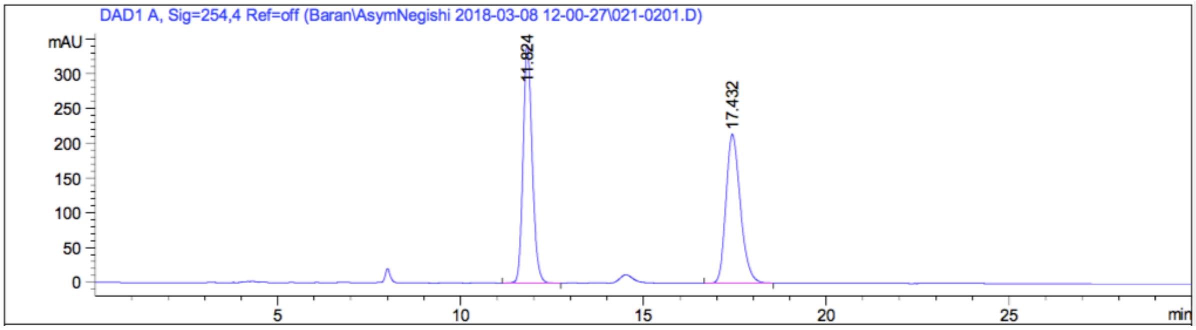

Enantioenriched **52:**

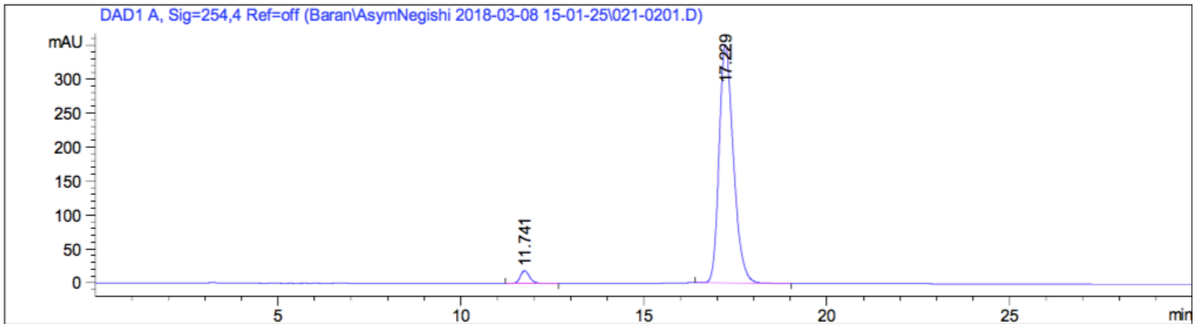

Signal 1: DAD1 A, Sig=254,4 Ref=off

Peak #	RetTime [min]	Type	Width [min]	Area [mAU*s]	Height [mAU]	Area %
1	11.824	BB	0.2665	5940.00928	342.21219	50.2913
2	17.432	BB	0.4219	5871.19775	214.10947	49.7087

Signal 1: DAD1 A, Sig=254,4 Ref=off

Peak #	RetTime [min]	Type	Width [min]	Area [mAU*s]	Height [mAU]	Area %
1	11.741	BB	0.2712	335.06812	18.86878	3.3851
2	17.229	BB	0.4183	9563.14258	350.47324	96.6149

Experimental Procedures and Characterization Data for Piperidine (*C-2*)-Scaffolds (Figure 3–d)

Compound B13

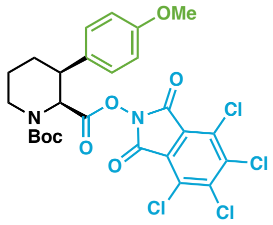

1-(*tert*-butyl) 2-(4,5,6,7-tetrachloro-1,3-dioxoisoindolin-2-yl) (2*S*,3*S*)-3-(4-methoxyphenyl)piperidine-1,2-dicarboxylate (B13)

Following General Procedure **B** on 1.37 mmol scale with **A10**. Purification by flash column chromatography (silica, 6:1 Hexanes:EtOAc) afforded 696 mg (82%) of the title compound **B13**.

Physical State: yellow oil.

^{1}H NMR (600 MHz, $CDCl_3$, both rotamers): δ 7.27 (d, J = 8.5 Hz, 2H), 6.88 (t, J = 7.2 Hz, 2H), 5.54 – 5.23 (m, 1H), 4.21 – 4.08 (m, 1H), 3.79 (s, 3H), 3.25 – 3.15 (m, 2H), 2.21 – 2.11 (m, 1H), 1.99 – 1.91 (m, 2H), 1.69 – 1.63 (m, 1H), 1.53 (s, 9H) ppm.

^{13}C NMR (151 MHz, $CDCl_3$, both rotamers): δ 166.5, 166.3, 158.9, 158.8, 157.1, 155.3, 154.6, 141.0, 131.6, 130.4, 129.2, 129.1, 124.8, 114.1, 114.0, 81.7, 81.2, 59.3, 57.7, 55.3, 43.8, 43.1, 41.4, 40.4, 28.5, 28.3, 25.4, 25.0, 24.6, 24.4 ppm.

HRMS (ESI-TOF): calc'd for $C_{26}H_{24}Cl_4N_2O_7Na$ [M+H]$^+$: 639.0235; found 639.0235.

TLC: R_f = 0.2 (4:1 Hexanes:EtOAc).

$[\alpha]_D^{20}$ = -55.8 (c = 1.0, $CHCl_3$).

Chiral HPLC: Chiralpak IA, 4.6 x 250 mm; 45/55 *i*PrOH/hexanes, 1 mL/min, 254 nm; t_R (minor) = 18.9 min, t_R (major) = 8.5 min, >99% *ee*.

Racemic:

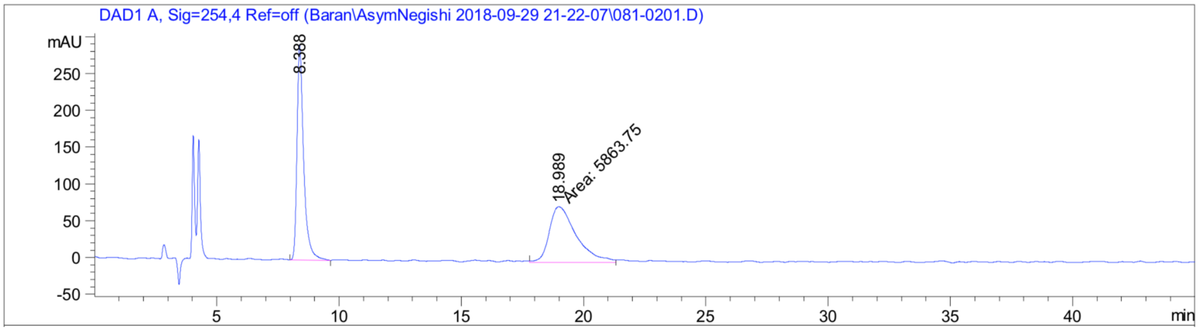

Enantioenriched **B13:**

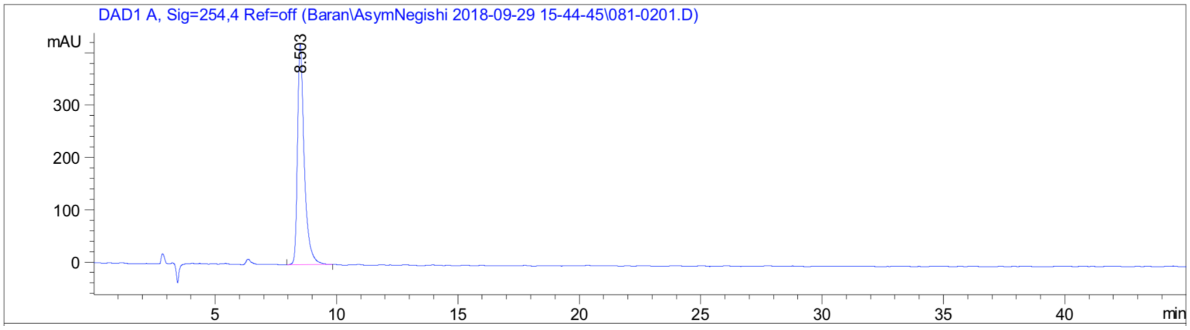

Signal 1: DAD1 A, Sig=254,4 Ref=off

Peak #	RetTime [min]	Type	Width [min]	Area [mAU*s]	Height [mAU]	Area %
1	8.388	BB	0.2817	5547.51758	291.98917	48.6144
2	18.989	MM	1.2896	5863.75000	75.78203	51.3856

Signal 1: DAD1 A, Sig=254,4 Ref=off

Peak #	RetTime [min]	Type	Width [min]	Area [mAU*s]	Height [mAU]	Area %
1	8.503	BB	0.2911	8191.09229	420.75574	100.0000

Compound 53

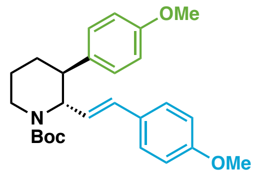

***tert*-butyl (2*S*,3*S*)-3-(4-methoxyphenyl)-2-((E)-4-methoxystyryl)piperidine-1-carboxylate (53)**

Following General Procedure **C1** on 0.1 mmol scale with **B13**, $NiCl_2$•$6H_2O$ (40 mol%), BPhen (45 mol%), and boronic acid **SI-21** (3.0 equiv.). Purification by pTLC (silica, 6:1 Hexanes:EtOAc) afforded 29.0 mg (69%) of the title compound **53**.

Physical State: colorless oil

^{1}H NMR (600 MHz, $CDCl_3$, both rotamers) δ 7.30 (d, J = 8.7 Hz, 2H), 7.28 – 7.26 (m, 2H), 6.86 (d, J = 8.7 Hz, 4H), 6.36 (dd, J = 15.9, 1.7 Hz, 1H), 6.14 (dd, J = 16.0, 5.4 Hz, 1H), 5.12 (s, 1H), 4.07 (dd, J = 14.0, 5.3 Hz, 1H), 3.81 (s, 3H), 3.80 (s, 3H), 3.08 – 2.98 (m, 2H), 2.02 – 1.97 (m, 1H), 1.80 – 1.76 (m, 1H), 1.62 – 1.58 (m, 1H), 1.49 (s, 9H), 1.48 – 1.41 (m, 1H), ppm.

^{13}C NMR (151 MHz, $CDCl_3$, both rotamers) δ 159.3, 158.0, 155.7, 136.4, 130.1, 129.9, 128.9, 127.6, 126.5, 114.1, 113.8, 79.8, 56.7, 55.5, 55.4, 42.1, 39.4, 28.7, 27.0, 20.2 ppm.

TLC: R_f = 0.2 (8:1 Hexanes:EtOAc).

HRMS (ESI-TOF): calc'd for $C_{26}H_{34}NO_4$ $[M+H]^+$: 424.2488; found 424.2480.

$[\alpha]_D^{20}$ = 9.2 (c = 1.0, $CHCl_3$).

Chiral HPLC: Chiralpak IA, 4.6 x 250 mm; 3/97 *i*PrOH/hexanes, 1 mL/min, 210 nm; t_R (minor) = 21.1 min, t_R (major) = 18.3min, >99% *ee*.

Racemic:

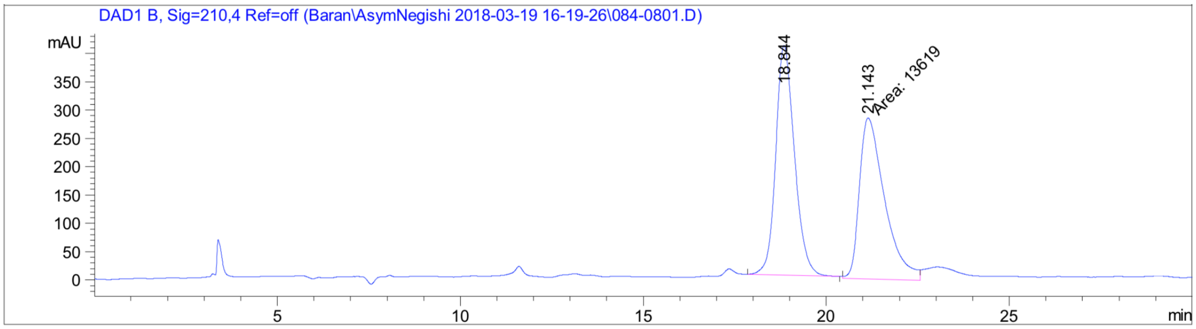

Enantioenriched **53:**

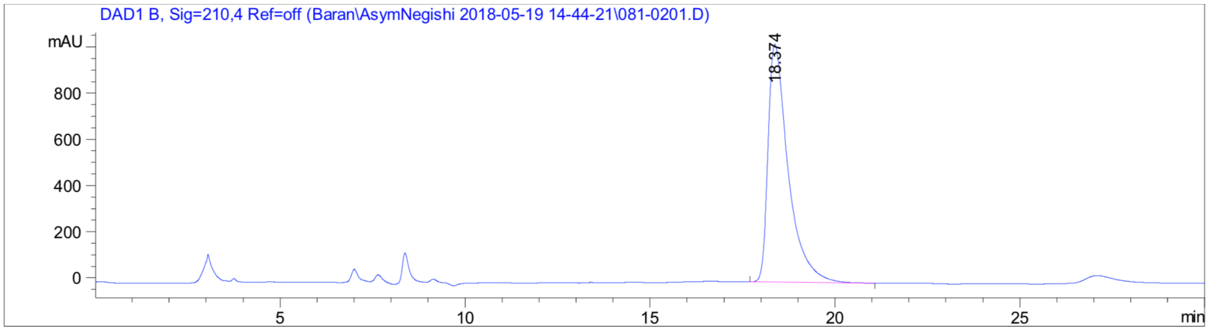

Signal 2: DAD1 B, Sig=210,4 Ref=off

Peak #	RetTime [min]	Type	Width [min]	Area [mAU*s]	Height [mAU]	Area %
1	18.844	BB	0.5411	1.44187e4	405.27939	51.4261
2	21.143	MM	0.7986	1.36190e4	284.21771	48.5739

Signal 2: DAD1 B, Sig=210,4 Ref=off

Peak #	RetTime [min]	Type	Width [min]	Area [mAU*s]	Height [mAU]	Area %
1	18.374	BB	0.5578	3.91423e4	1029.05542	100.0000

Compound 54

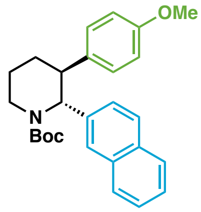

***tert*-butyl (2*R*,3*S*)-3-(4-methoxyphenyl)-2-(naphthalen-2-yl)piperidine-1-carboxylate (54)**

Following General Procedure **C1** on 0.1 mmol scale with **B13**, $NiCl_2$•$6H_2O$ (40 mol%), BPhen (45 mol%), and boronic acid **SI-11** (3.0 equiv.). Purification by pTLC (silica, 6:1 Hexanes:EtOAc) afforded 29.0 mg (70%) of the title compound **54.**

Physical State: colorless oil

^{1}H NMR (600 MHz, $CDCl_3$, both rotamers) δ 7.83 – 7.75 (m, 3H), 7.60 (s, 1H), 7.49 – 7.43 (m, 2H), 7.29 (dd, J = 8.5, 1.9 Hz, 1H), 7.26 – 7.23 (m, 2H), 6.85 (d, J = 8.7 Hz, 2H), 5.55 (d, J = 4.7 Hz, 1H), 4.27 (ddd, J = 13.8, 6.3, 2.2 Hz, 1H), 3.81 (s, 3H), 3.48 (t, J = 7.2 Hz, 1H), 3.31 – 3.22 (m, 1H), 1.94 – 1.78 (m, 3H), 1.55 (d, J = 11.1 Hz, 1H), 1.37 (s, 9H) ppm.

^{13}C NMR (151 MHz, $CDCl_3$, both rotamers) δ 158.1, 156.0, 139.8, 136.1, 133.4, 132.4, 129.0, 128.1, 128.0, 127.6, 126.1, 125.7, 125.1, 124.9, 113.8, 80.0, 59.8, 55.4, 42.8, 40.0, 28.5, 26.8, 20.5 ppm.

HRMS (ESI-TOF): calc'd for $C_{27}H_{31}NO_3Na$ $[M+Na]^+$: 440.2202; found 440.2198.

TLC: R_f = 0.3 (8:1 Hexanes:EtOAc).

$[\alpha]_D^{20}$ = 14.1 (c = 1.0, $CHCl_3$).

Chiral HPLC: Chiralpak ODH, 4.6 x 250 mm; 5/95 *i*PrOH/hexanes, 1 mL/min, 254 nm; t_R (minor) = 6.7 min, t_R (major) = 7.6 min, 98.5% *ee*.

Racemic:

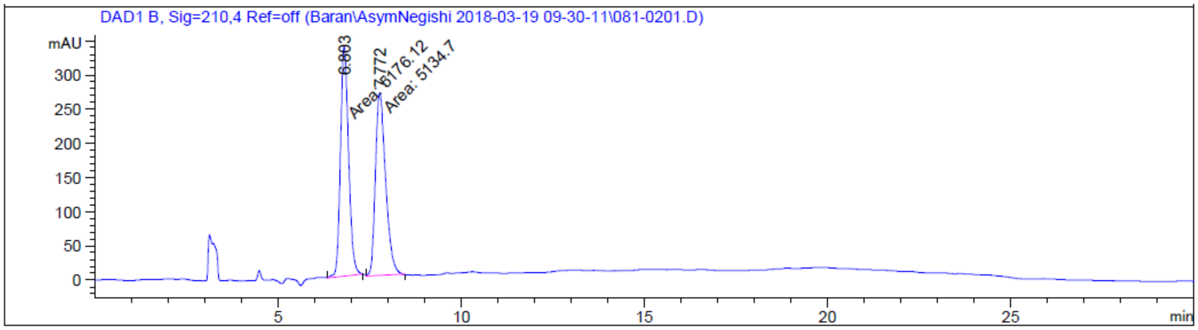

Enantioenriched **54:**

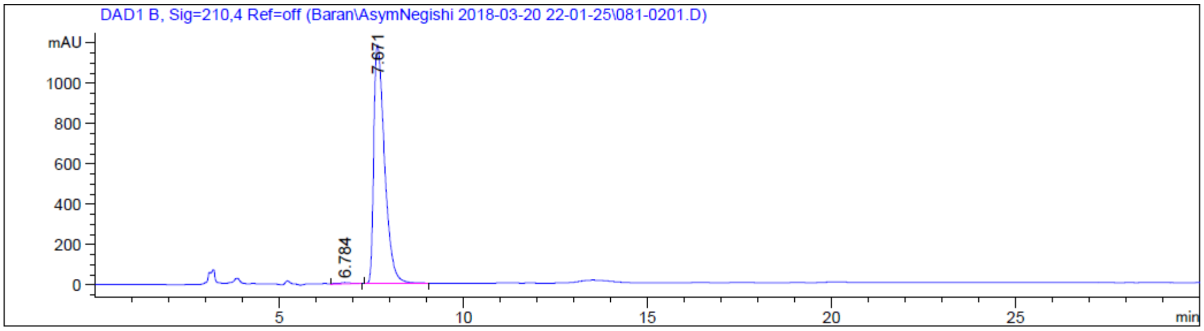

Signal 2: DAD1 B, Sig=210,4 Ref=off

Peak #	RetTime [min]	Type	Width [min]	Area [mAU*s]	Height [mAU]	Area %
1	6.803	MM	0.2570	5176.12061	335.64398	50.2009
2	7.772	MM	0.3209	5134.69580	266.67416	49.7991

Signal 2: DAD1 B, Sig=210,4 Ref=off

Peak #	RetTime [min]	Type	Width [min]	Area [mAU*s]	Height [mAU]	Area %
1	6.784	VB	0.3640	187.79649	7.06824	0.7746
2	7.671	BB	0.3114	2.40578e4	1182.08752	99.2254

Compound 55

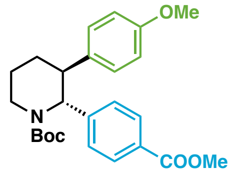

***tert*-butyl (2*R*,3*S*)-2-(4-(methoxycarbonyl)phenyl)-3-(4-methoxyphenyl)piperidine-1-carboxylate (55)**

Following General Procedure **C1** on 0.05 mmol scale with **B13**, $NiCl_2$•$6H_2O$ (40 mol%), BPhen (45 mol%), and boronic acid **SI-7** (3.0 equiv.). Purification by pTLC (silica, 6:1 Hexanes:EtOAc) afforded 15.0 mg (71%) of the title compound **55**.

Physical State: colorless oil

^{1}H NMR (600 MHz, $CDCl_3$, both rotamers) δ 7.92 (d, J = 8.2 Hz, 2H), 7.15 (d, J = 8.1 Hz, 2H), 7.12 (d, J = 8.6 Hz, 2H), 6.82 (d, J = 8.7 Hz, 2H), 5.26 (d, J = 6.0 Hz, 1H), 4.21 (dd, J = 14.1, 4.3 Hz, 1H), 3.90 (s, 3H), 3.79 (s, 3H), 3.25 – 3.20 (m, 2H), 1.86 – 1.76 (m, 3H), 1.60 (s, 1H), 1.33 (s, 9H) ppm.

^{13}C NMR (151 MHz, $CDCl_3$, both rotamers) δ 167.1, 158.3, 155.8, 148.3, 135.4, 129.7, 129.0, 128.6, 126.4, 113.9, 80.2, 60.8, 55.4, 52.2, 43.7, 39.9, 28.5, 27.0, 20.7 ppm.

HRMS (ESI-TOF): calc'd for $C_{25}H_{32}NO_5$ $[M+H]^+$: 426.2280; found 426.2279.

TLC: R_f = 0.3 (4:1 Hexanes:EtOAc).

$[\alpha]_D^{20}$ = 31.3 (c = 1.0, $CHCl_3$).

Chiral HPLC: Chiralpak IC, 4.6 x 250 mm; 10/90 *i*PrOH/hexanes, 1 mL/min, 210 nm; t_R (minor) = 15.3 min, t_R (major) = 16.8 min, 98.5% *ee*.

Racemic:

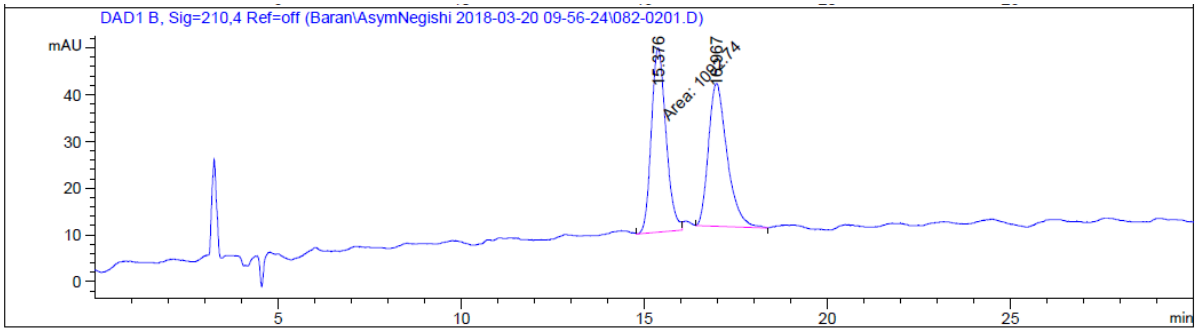

Enantioenriched **55:**

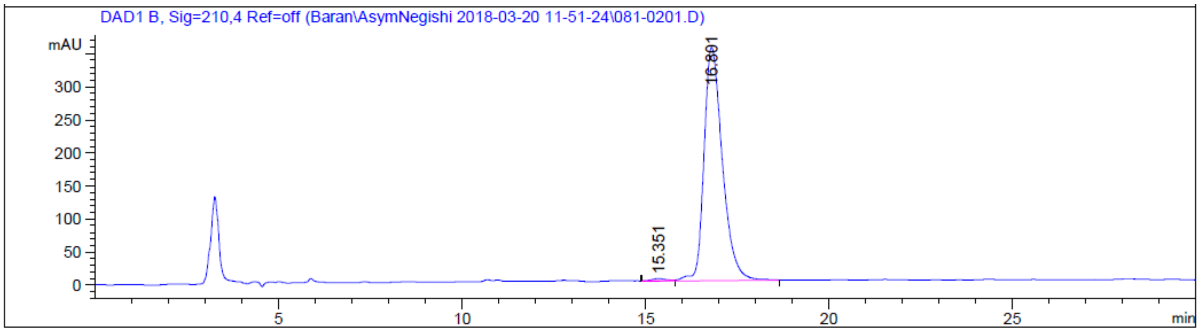

Signal 2: DAD1 B, Sig=210,4 Ref=off

Peak #	RetTime [min]	Type	Width [min]	Area [mAU*s]	Height [mAU]	Area %
1	15.376	MM	0.4625	1092.73706	39.37962	50.3752
2	16.967	BB	0.5131	1076.45874	30.54625	49.6248

Peak #	RetTime [min]	Type	Width [min]	Area [mAU*s]	Height [mAU]	Area %
1	15.351	BV E	0.3096	83.79869	3.30735	0.6788
2	16.801	VB R	0.5296	1.22609e4	352.69928	99.3212

Compound 56

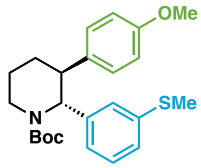

***tert*-butyl (2*R*,3*S*)-3-(4-methoxyphenyl)-2-(3-(methylthio)phenyl)piperidine-1-carboxylate (56)**

Following General Procedure **C1** on 0.1 mmol scale with **B13**, $NiCl_2$•$6H_2O$ (40 mol%), BPhen (45 mol%), and boronic acid **SI-8** (3.0 equiv.). Purification by pTLC (silica, 4:1 Hexanes:EtOAc) afforded 31.8 mg (77%) of the title compound **56**.

Physical State: colorless oil

^{1}H NMR (600 MHz, $CDCl_3$, both rotamers) δ 7.22 – 7.16 (m, 3H), 7.10 (dd, *J* = 8.3, 1.5 Hz, 1H), 7.00 (s, 1H), 6.92 (d, *J* = 7.7 Hz, 1H), 6.84 (d, *J* = 8.6 Hz, 2H), 5.31 (d, *J* = 5.0 Hz, 1H), 4.24 – 4.12 (m, 1H), 3.80 (s, 3H), 3.28 (dd, *J* = 7.8, 3.6 Hz, 1H), 3.15 (ddd, *J* = 13.3, 11.7, 4.7 Hz, 1H), 2.40 (s, 3H), 1.87 – 1.75 (m, 3H), 1.53 – 1.48 (m, 1H), 1.38 (s, 9H) ppm.

^{13}C NMR (151 MHz, $CDCl_3$, both rotamers) δ 158.1, 155.9, 143.2, 138.4, 135.9, 129.0, 128.8, 125.0, 124.7, 123.2, 113.8, 80.0, 59.8, 55.4, 43.0, 39.9, 28.5, 26.8, 20.5, 16.0 ppm.

HRMS (ESI-TOF): calc'd for $C_{24}H_{32}NO_3S$ $[M+H]^+$: 414.2103; found 414.2096.

TLC: R_f = 0.2 (8:1 Hexanes:EtOAc).

$[\alpha]_D^{20}$ = 26.0 (*c* = 1.0, $CHCl_3$).

Chiral HPLC: Chiralpak IA, 4.6 x 250 mm; 1/99 *i*PrOH/hexanes, 1 mL/min, 210 nm; t_R (minor) = 24.7 min, t_R (major) = 14.3 min, >99% *ee*.

Racemic:

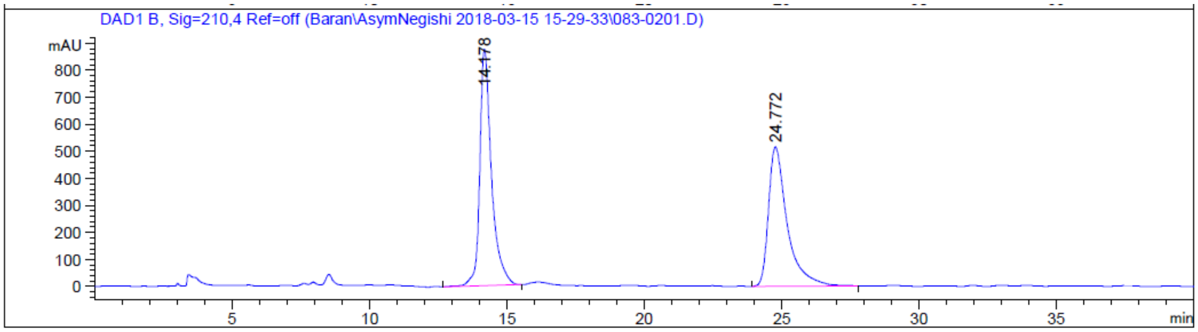

Enantioenriched **56:**

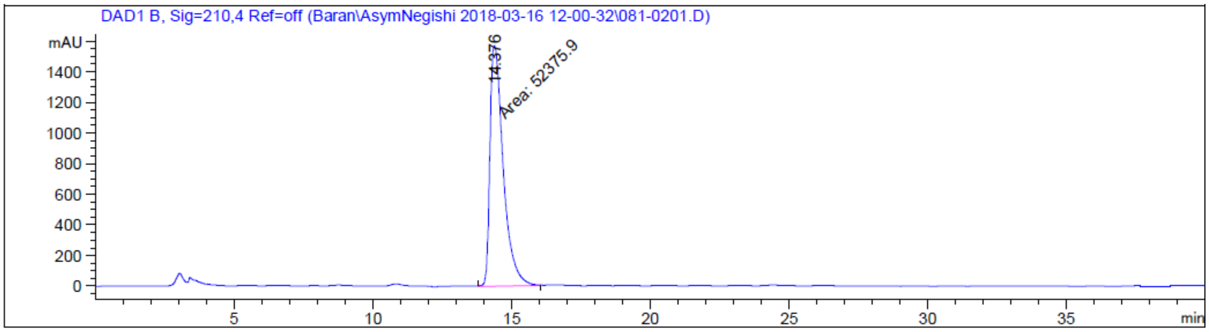

Signal 2: DAD1 B, Sig=210,4 Ref=off

Peak #	RetTime [min]	Type	Width [min]	Area [mAU*s]	Height [mAU]	Area %
1	14.178	BB	0.4387	2.58809e4	875.85364	51.5339
2	24.772	BB	0.6995	2.43402e4	514.88135	48.4661

Signal 2: DAD1 B, Sig=210,4 Ref=off

Peak #	RetTime [min]	Type	Width [min]	Area [mAU*s]	Height [mAU]	Area %
1	14.376	MM	0.5555	5.23759e4	1571.38782	100.0000

Compound 57

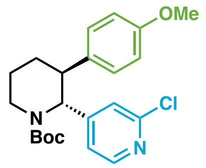

***Tert*-butyl (2*R*,3*S*)-2-(2-chloropyridin-4-yl)-3-(4-methoxyphenyl)piperidine-1-carboxylate (57)**

Following General Procedure **C1** on 0.05 mmol scale with **B13**, $NiCl_2•6H_2O$ (40 mol%), BPhen (45 mol%), and boronic acid **SI-23** (3.0 equiv.). Purification by pTLC (silica, 5:1 Hexanes:EtOAc) afforded 19.1 mg (95%) of the title compound **57**.

Physical State: colorless oil

^{1}H NMR (600 MHz, $CDCl_3$, both rotamers) δ 8.24 (d, J = 5.2 Hz, 1H), 7.15 – 7.09 (m, 2H), 7.05 (s, 1H), 6.88 (dd, J = 5.2, 1.6 Hz, 1H), 6.87 – 6.82 (m, 2H), 5.19 (d, J = 6.1 Hz, 1H), 4.18 (d, J = 13.9 Hz, 1H), 3.80 (s, 3H), 3.19 – 3.14 (m, 2H), 1.89 – 1.77 (m, 3H), 1.61 – 1.55 (m, 1H), 1.38 (s, 9H) ppm.

^{13}C NMR (151 MHz, $CDCl_3$, both rotamers) δ 158.5, 155.5, 155.5, 152.0, 149.6, 134.46, 128.9, 122.0, 120.6, 114.1, 80.8, 59.9, 55.4, 43.4, 40.0, 28.4, 27.2, 20.6 ppm.

HRMS (ESI-TOF): calc'd for $C_{22}H_{28}ClN_2O_3$ $[M+H]^+$: 403.1788; found 403.1782.

TLC: R_f = 0.3 (2:1 Hexanes:EtOAc).

$[\alpha]_D^{20}$ = 34.9 (c = 1.0, $CHCl_3$).

Chiral HPLC: Chiralpak IC, 4.6 x 250 mm; 20/80 *i*PrOH/hexanes, 1 mL/min, 210 nm; t_R (minor) = 15.3 min, t_R (major) = 10.8 min, 99% *ee*.

Racemic:

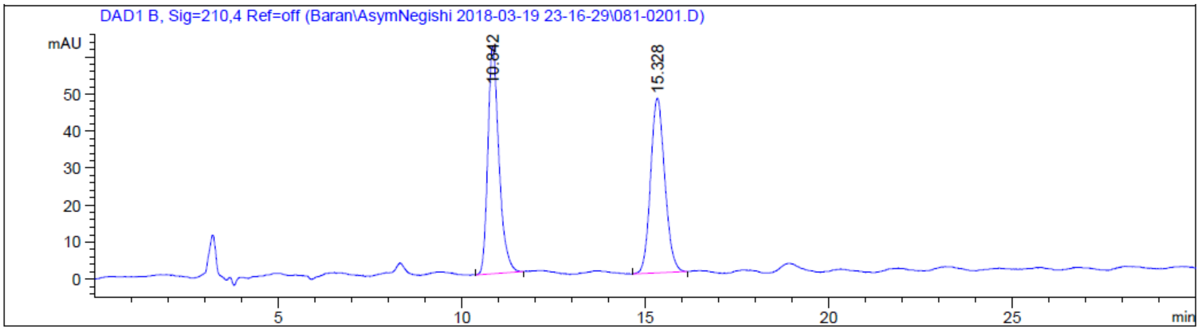

Enantioenriched **57:**

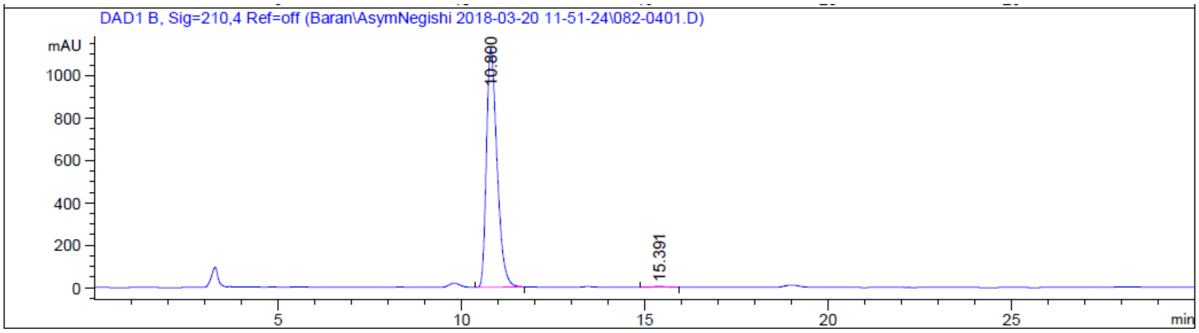

Signal 2: DAD1 B, Sig=210,4 Ref=off

Peak #	RetTime [min]	Type	Width [min]	Area [mAU*s]	Height [mAU]	Area %
1	10.842	BB	0.3175	1293.96545	61.50292	50.3203
2	15.328	BB	0.4074	1277.49341	47.25077	49.6797

Signal 2: DAD1 B, Sig=210,4 Ref=off

Peak #	RetTime [min]	Type	Width [min]	Area [mAU*s]	Height [mAU]	Area %
1	10.800	BB	0.3082	2.26019e4	1126.11255	99.4221
2	15.391	BB	0.3276	131.36803	5.21976	0.5779

Compound 58

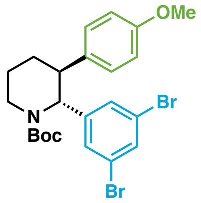

***tert*-butyl (2*R*,3*S*)-2-(3,5-dibromophenyl)-3-(4-methoxyphenyl)piperidine-1-carboxylate (58)**

Following General Procedure **C1** on 0.1 mmol scale with **B13**, $NiCl_2$•$6H_2O$ (40 mol%), BPhen (45 mol%), and boronic acid **SI-10** (3.0 equiv.). Purification by pTLC (silica, 5:1 Hexanes:EtOAc) afforded 25.9 mg (50%) of the title compound **58**.

Physical State: colorless oil

^{1}H NMR (600 MHz, $CDCl_3$, both rotamers) δ 7.50 (s, 1H), 7.15 (s, 2H), 7.12 (d, J = 8.6 Hz, 2H), 6.84 (d, J = 8.6 Hz, 2H), 5.17 (d, J = 5.8 Hz, 1H), 4.17 (dq, J = 13.9, 2.6 Hz, 1H), 3.80 (s, 3H), 3.21 – 3.11 (m, 2H), 1.82 (t, J = 4.2 Hz, 3H), 1.52 (d, J = 6.8 Hz, 1H), 1.39 (s, 9H) ppm.

^{13}C NMR (151 MHz, $CDCl_3$, both rotamers) δ 158.4, 155.6, 146.8, 135.0, 132.4, 128.9, 128.4, 123.0, 114.0, 80.5, 59.9, 55.4, 43.4, 39.9, 28.5, 26.9, 20.6 ppm.

HRMS (ESI-TOF): calc'd for $C_{23}H_{27}Br_2NO_3Na$ $[M+Na]^+$: 546.0255; found 546.0250.

TLC: R_f = 0.3 (8:1 Hexanes:EtOAc).

$[\alpha]_D^{20}$ = 21.1 (c = 1.0, $CHCl_3$).

Chiral SFC: IG column (3 µm, 4.6x250 mm) under isocratic conditions [3% MeOH / CO_2 (4 mL/min), 1600 psi backpressure] at 30 °C. The enantiomers were detected by UV light (260 nm). t_R (major) = 1.68 min, t_R (minor) = 2.41 min, >99% *ee*.

Racemic:

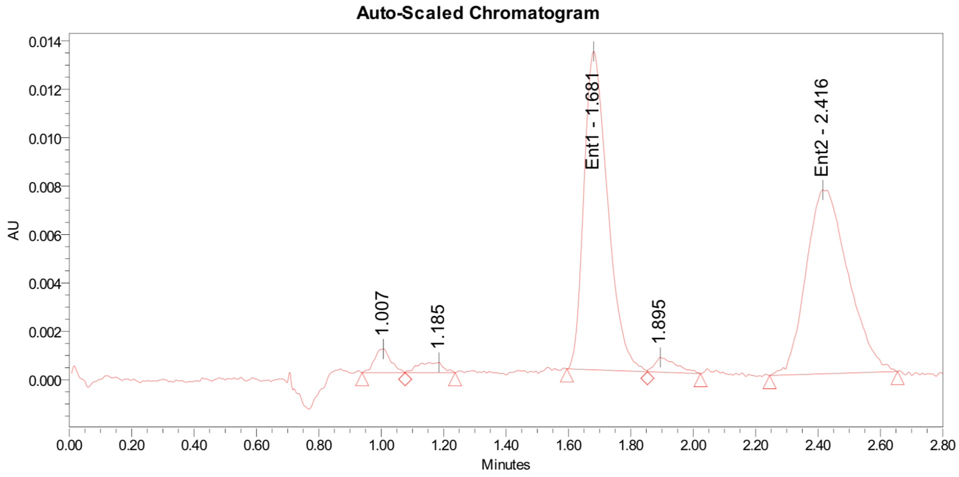

Enantioenriched **58:**

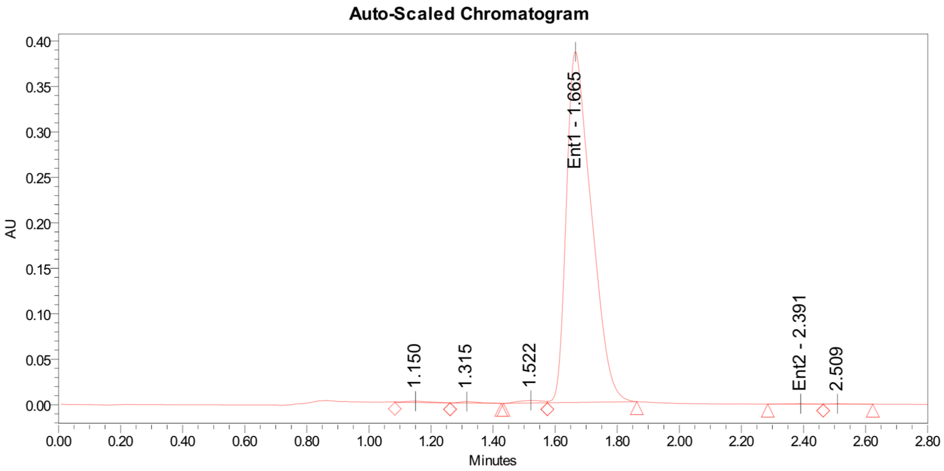

KSF-142	49.42	50.58	-1.16	69743	71385

MS-2-73-3	99.88	0.12	99.75	2156802	2690

Compound 59

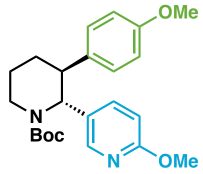

***tert*-butyl (2*R*,3*S*)-3-(4-methoxyphenyl)-2-(6-methoxypyridin-3-yl)piperidine-1-carboxylate (59)**

Following General Procedure **C1** on 0.1 mmol scale with **B13**, $NiCl_2$•$6H_2O$ (40 mol%), BPhen (45 mol%), and boronic acid **SI-15** (3.0 equiv.). Purification by pTLC (silica, 5:1 Hexanes:EtOAc) afforded 12.0 mg (30%) of the title compound **59**.

Physical State: colorless oil

^{1}H NMR (600 MHz, $CDCl_3$, both rotamers) δ 7.95 (d, J = 2.6 Hz, 1H), 7.36 (dd, J = 8.6, 2.6 Hz, 1H), 7.15 (d, J = 8.7 Hz, 2H), 6.83 (d, J = 8.7 Hz, 2H), 6.66 (d, J = 8.6 Hz, 1H), 5.25 (d, J = 5.7 Hz, 1H), 4.21 – 4.13 (m, 1H), 3.91 (s, 3H), 3.79 (s, 3H), 3.20 (td, J = 6.4, 3.8 Hz, 1H), 3.15 – 3.10 (m, 1H), 1.89 – 1.77 (m, 3H), 1.57 – 1.53 (m, 1H), 1.39 (s, 9H) ppm.

^{13}C NMR (151 MHz, $CDCl_3$, both rotamers) δ 163.1, 158.2, 155.6, 144.9, 137.4, 135.4, 130.5, 129.0, 113.9, 110.5, 80.1, 58.0, 55.4, 53.5, 43.1, 39.5, 28.5, 26.9, 20.8 ppm.

HRMS (ESI-TOF): calc'd for $C_{23}H_{31}N_2O_4$ $[M+H]^+$: 399.2284; found 399.2286.

TLC: R_f = 0.3 (2:1 Hexanes:EtOAc).

$[\alpha]_D^{20}$ = 20.4 (c = 1.0, $CHCl_3$).

Chiral HPLC: Chiralpak IA, 4.6 x 250 mm; 20/80 *i*PrOH/hexanes, 1 mL/min, 254 nm; t_R (minor) = 16.7 min, t_R (major) = 14.8 min, >99% *ee*.

Racemic:

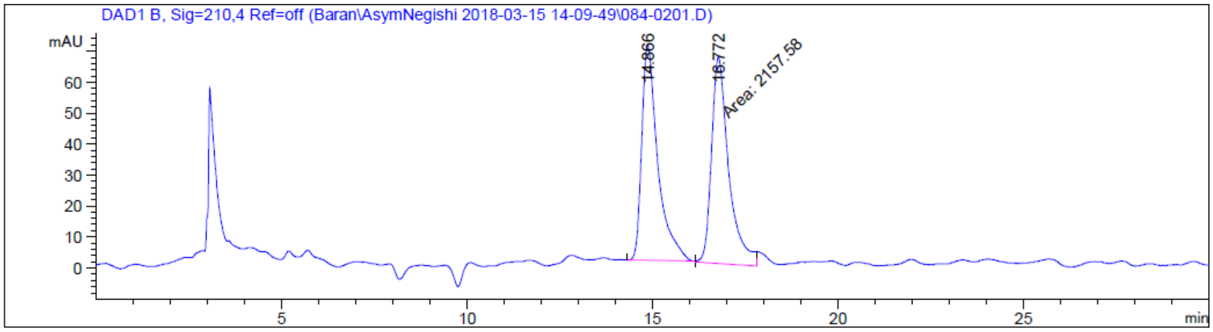

Enantioenriched **59:**

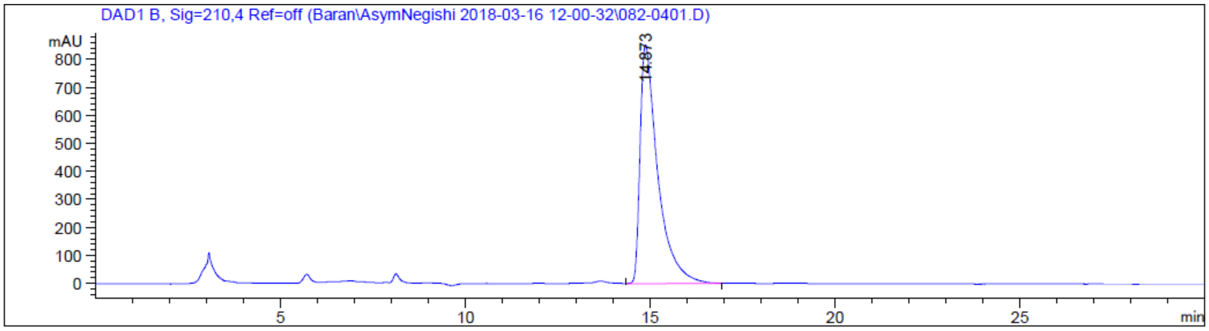

Signal 2: DAD1 B, Sig=210,4 Ref=off

Peak #	RetTime [min]	Type	Width [min]	Area [mAU*s]	Height [mAU]	Area %
1	14.866	BB	0.4433	2096.68799	69.64073	49.2844
2	16.772	MM	0.5391	2157.57544	66.70678	50.7156

Signal 2: DAD1 B, Sig=210,4 Ref=off

Peak #	RetTime [min]	Type	Width [min]	Area [mAU*s]	Height [mAU]	Area %
1	14.873	BB	0.4852	2.82749e4	852.05243	100.0000

Experimental Procedures and Characterization Data for Piperidine (*C-3*)-Scaffolds (Figure 3–e)

Compound B14

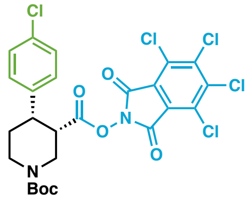

1-(*tert*-butyl) 3-(4,5,6,7-tetrachloro-1,3-dioxoisoindolin-2-yl) (3*R*,4*R*)-4-(4-chlorophenyl)piperidine-1,3-dicarboxylate (B14)

Following General Procedure **B** on 1.5 mmol scale with **A11**. Purification by flash column chromatography (silica, 4:1 Hexanes:EtOAc) afforded 772 mg (83%) of the title compound **B14**.

Physical State: yellow oil.

^{1}H NMR (600 MHz, $CDCl_3$, both rotamers): δ 7.34 (d, *J* = 7.5 Hz, 2H), 7.21 (s, 2H), 4.78 – 4.30 (m, 2H), 3.44 – 3.06 (m, 3H), 2.88 (d, *J* = 73.9 Hz, 1H), 2.56 – 2.39 (m, 1H), 1.78 (d, *J* = 13.5 Hz, 1H), 1.46 (s, 9H) ppm.

^{13}C NMR (151 MHz, $CDCl_3$, both rotamers): δ 167.4, 157.1, 154.4, 141.0, 139.4, 133.2, 130.5, 129.1, 128.9, 124.8, 80.5, 46.1, 45.3, 44.3, 43.6, 43.3, 43.1, 28.2, 25.7 ppm.

HRMS (ESI-TOF): calc'd for $C_{25}H_{22}Cl_5N_2O_6$ $[M+H]^+$: 620.9921; found 620.9901.

TLC: R_f = 0.3 (2:1 Hexanes:EtOAc).

$[\alpha]_D^{20}$ = -56.5 (*c* = 1.0, $CHCl_3$).

Chiral HPLC: Chiralpak IA, 4.6 x 250 mm; 45/55 *i*PrOH/hexanes, 1 mL/min, 254 nm; t_R (minor) = 18.2 min, t_R (major) = 9.8 min, >99% *ee*.

Racemic:

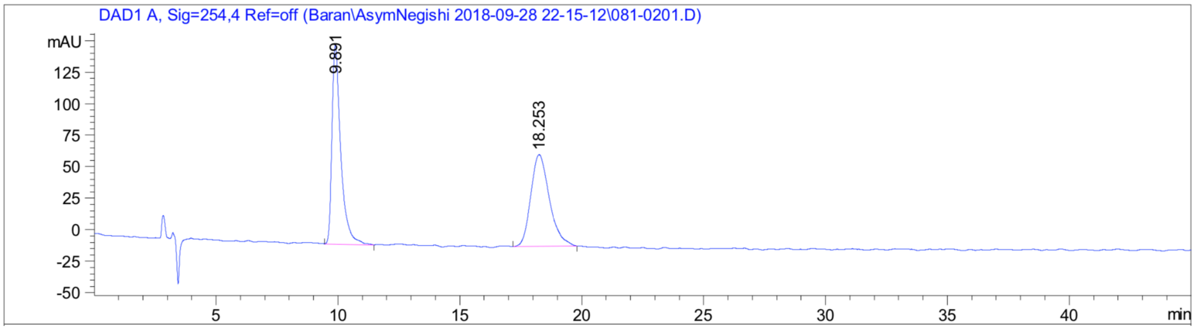

Enantioenriched **B14:**

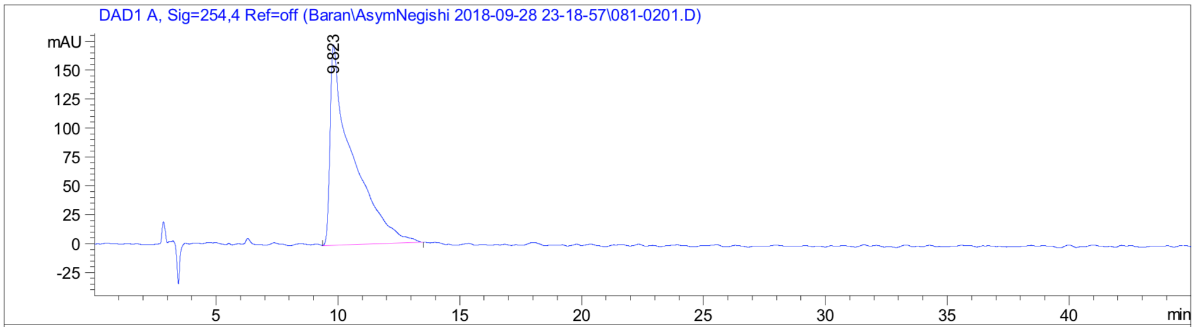

Signal 1: DAD1 A, Sig=254,4 Ref=off

Peak #	RetTime [min]	Type	Width [min]	Area [mAU*s]	Height [mAU]	Area %
1	9.891	BB	0.3728	3963.37134	157.90796	50.5948
2	18.253	BB	0.8085	3870.19067	72.76498	49.4052

Signal 1: DAD1 A, Sig=254,4 Ref=off

Peak #	RetTime [min]	Type	Width [min]	Area [mAU*s]	Height [mAU]	Area %
1	9.823	BB	0.8237	1.10843e4	172.96115	100.0000

Compound 60

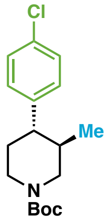

***tert*-butyl (3*R*,4*S*)-4-(4-chlorophenyl)-3-methylpiperidine-1-carboxylate (60)**

Following General Procedure **C2** on 0.1 mmol scale with **B14**, $NiCl_2$•glyme (30 mol%), di^{t}BuBipy (40 mol%), and Zinc reagent **SI-26** (2.0 equiv.). Purification by pTLC (silica, 7:1 Hexanes: EtOAc) afforded 26.3 mg (85%) of the title compound **60**.

Physical State: colorless oil

^{1}H NMR (600 MHz, $CDCl_3$, both rotamers): δ 7.26 (d, J = 8.4 Hz, 2H), 7.08 (d, J = 8.3 Hz, 2H), 4.19 (s, 2H), 2.75 (s, 1H), 2.40 (s, 1H), 2.16 (td, J = 11.6, 3.8 Hz, 1H), 1.71 (d, J = 11.4 Hz, 2H), 1.60 (d, J = 3.8 Hz, 1H), 1.48 (s, 9H), 0.66 (d, J = 6.6 Hz, 3H) ppm.

^{13}C NMR (151 MHz, $CDCl_3$, both rotamers): δ 154.8, 143.2, 132.1, 129.0, 128.7, 79.7, 51.0, 50.3, 44.8, 36.5, 34.3, 28.6, 16.9 ppm.

HRMS (ESI-TOF): calc'd for $C_{17}H_{25}ClNO_2$ $[M+H]^+$: 310.1574; found 310.1578.

TLC: R_f = 0.3 (8:1 Hexanes:EtOAc).

$[\alpha]_D^{20}$= 2.9 (c = 1.0, $CHCl_3$).

Chiral HPLC: Chiralpak IC, 4.6 x 250 mm; 5/95 *i*PrOH/hexanes, 0.5 mL/min, 210 nm; t_R (minor) = 12.3 min, t_R (major) = 14.1 min, 98% *ee*.

Racemic:

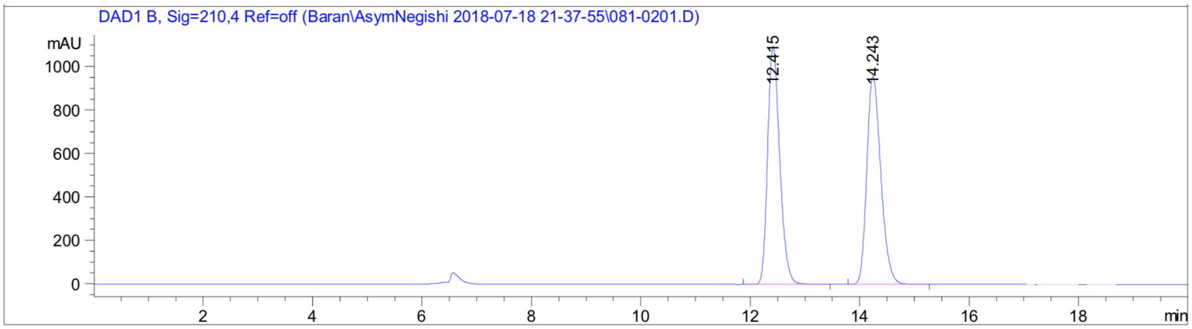

Enantioenriched **60:**

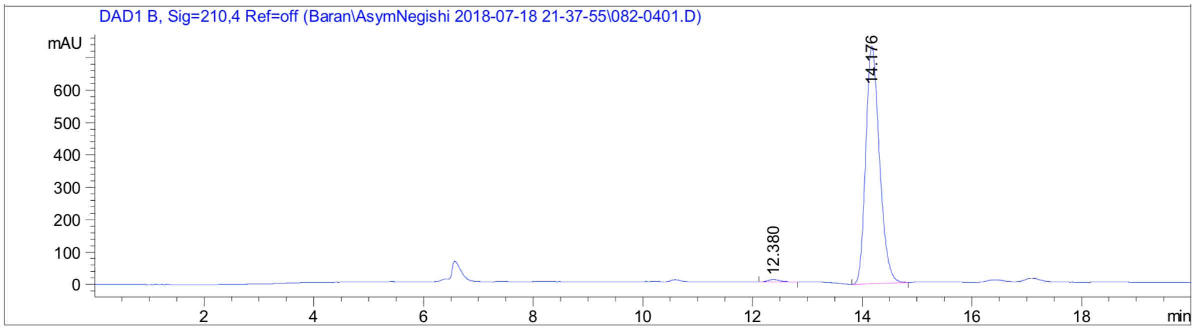

Signal 2: DAD1 B, Sig=210,4 Ref=off

Peak #	RetTime [min]	Type	Width [min]	Area [mAU*s]	Height [mAU]	Area %
1	12.415	BB	0.2379	1.68894e4	1095.56909	49.7838
2	14.243	BB	0.2755	1.70361e4	957.74109	50.2162

Signal 2: DAD1 B, Sig=210,4 Ref=off

Peak #	RetTime [min]	Type	Width [min]	Area [mAU*s]	Height [mAU]	Area %
1	12.380	BB	0.2236	108.97846	7.41083	0.8465
2	14.176	BB	0.2693	1.27643e4	732.58002	99.1535

Compound 61

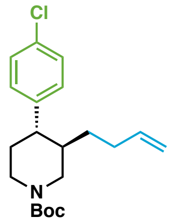

***tert*-butyl (3*R*,4*S*)-3-(but-3-en-1-yl)-4-(4-chlorophenyl)piperidine-1-carboxylate (61)**

Following General Procedure **C2** on 0.1 mmol scale with **B14**, $NiCl_2$•glyme (30 mol%), di*t*BuBipy (40 mol%), and Zinc reagent **SI-25** (2.0 equiv.). Purification by pTLC (silica, 10:1 Hexanes:EtOAc) afforded 26.8 mg (77%) of the title compound **61**.

Physical State: colorless oil

^{1}H NMR (600 MHz, $CDCl_3$, both rotamers): δ 7.29 – 7.24 (m, 2H), 7.07 (d, J = 8.5 Hz, 2H), 5.66 – 5.55 (m, 1H), 4.93 – 4.88 (m, 2H), 4.33 – 4.20 (m, 2H), 2.74 (s, 1H), 2.39 (s, 1H), 2.27 (td, J = 11.6, 3.8 Hz, 1H), 2.06 – 2.00 (m, 1H), 1.91 – 1.82 (m, 1H), 1.72 (d, J = 11.7 Hz, 1H), 1.63 (s, 1H), 1.49 (s, 9H), 1.26 – 1.21 (m, 2H), 0.98 (ddt, J = 13.8, 9.2, 4.6 Hz, 1H) ppm.

^{13}C NMR (151 MHz, $CDCl_3$, both rotamers): δ 154.8, 143.1, 138.3, 132.1, 129.0, 128.8, 115.0, 79.7, 49.0, 48.5, 44.5, 40.5, 34.7, 30.8, 30.5, 28.6 ppm.

HRMS (ESI-TOF): calc'd for $C_{20}H_{29}ClNO_2$ $[M+H]^+$: 350.1887; found 350.1883.

TLC: R_f = 0.4 (8:1 Hexanes:EtOAc).

$[\alpha]_D^{20}$ = 8.9 (c = 0.5, $CHCl_3$).

Chiral HPLC: Chiralpak IC, 4.6 x 250 mm; 10/90 *i*PrOH/hexanes, 0.5 mL/min, 210 nm; t_R (minor) = 9.8 min, t_R (major) = 11.2 min, 98% *ee*.

Racemic:

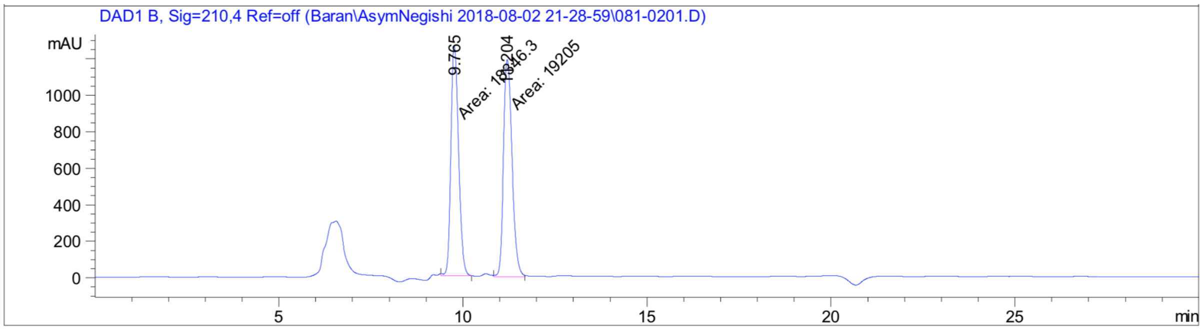

Enantioenriched **61:**

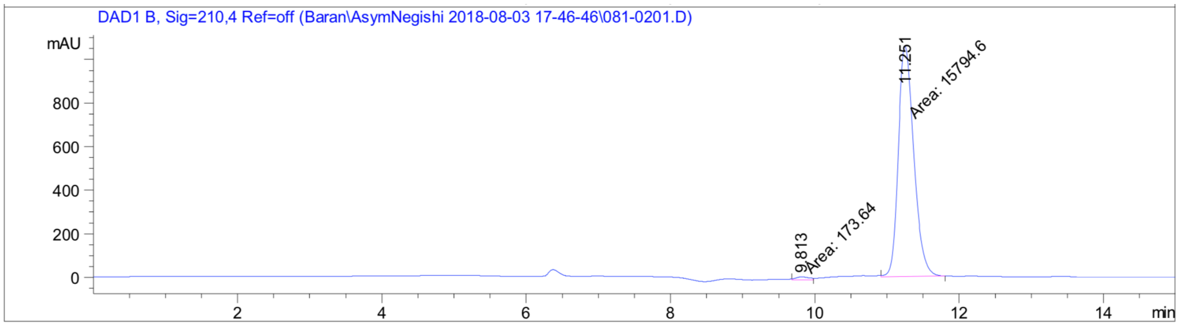

Signal 2: DAD1 B, Sig=210,4 Ref=off

Peak #	RetTime [min]	Type	Width [min]	Area [mAU*s]	Height [mAU]	Area %
1	9.765	MM	0.2432	1.83463e4	1257.40454	48.8567
2	11.204	MM	0.2688	1.92050e4	1190.69885	51.1433

Signal 2: DAD1 B, Sig=210,4 Ref=off

Peak #	RetTime [min]	Type	Width [min]	Area [mAU*s]	Height [mAU]	Area %
1	9.813	MM	0.2122	173.64006	13.63709	1.0874
2	11.251	MM	0.2491	1.57946e4	1056.90015	98.9126

Compound 62

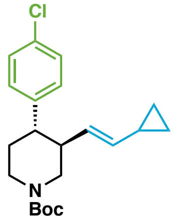

***tert*-butyl (3*R*,4*S*)-4-(4-chlorophenyl)-3-((E)-2-cyclopropylvinyl)piperidine-1-carboxylate (62)**

Following General Procedure **C1** on 0.1 mmol scale with **B14**, $NiCl_2$•glyme (40 mol%), diOMeBipy (40 mol%), **SI-22** (2.0 equiv.). Purification by pTLC (silica, 10:1 Hexanes:EtOAc) afforded 32.8 mg (91%) of the title compound **62**.

Physical State: colorless oil

^{1}H NMR (600 MHz, $CDCl_3$, both rotamers): δ 7.23 (d, J = 8.4 Hz, 2H), 7.04 (d, J = 8.4 Hz, 2H), 5.06 (dd, J = 15.5, 7.6 Hz, 1H), 4.78 (dd, J = 15.5, 8.4 Hz, 1H), 4.19 (s, 2H), 2.75 (s, 1H), 2.54 (s, 1H), 2.37 (td, J = 11.6, 3.8 Hz, 1H), 2.24 (s, 1H), 1.80 – 1.71 (m, 1H), 1.63 – 1.57 (m, 1H), 1.48 (s, 9H), 1.16 – 1.11 (m, 1H), 0.55 – 0.50 (m, 2H), 0.14– 0.08 (m, 2H) ppm.

^{13}C NMR (151 MHz, $CDCl_3$, both rotamers): δ 154.3, 142.3, 135.9, 131.3, 128.6, 127.9, 126.2, 79.1, 48.8, 47.9, 43.9, 33.4, 28.0, 13.0, 6.1, 5.9 ppm.

HRMS (ESI-TOF): calc'd for $C_{16}H_{20}ClN$ [M-Boc+H]$^+$: 262.1363; found 262.1357.

TLC: R_f = 0.4 (8:1 Hexanes:EtOAc).

$[\alpha]_D^{20}$ = 9.2 (c = 0.5, $CHCl_3$).

Chiral HPLC: Chiralpak IC, 4.6 x 250 mm; 5/95 *i*PrOH/hexanes, 0.5 mL/min, 210 nm; t_R (minor) = 14.5 min, t_R (major) = 15.8 min, 94% *ee*.

Racemic:

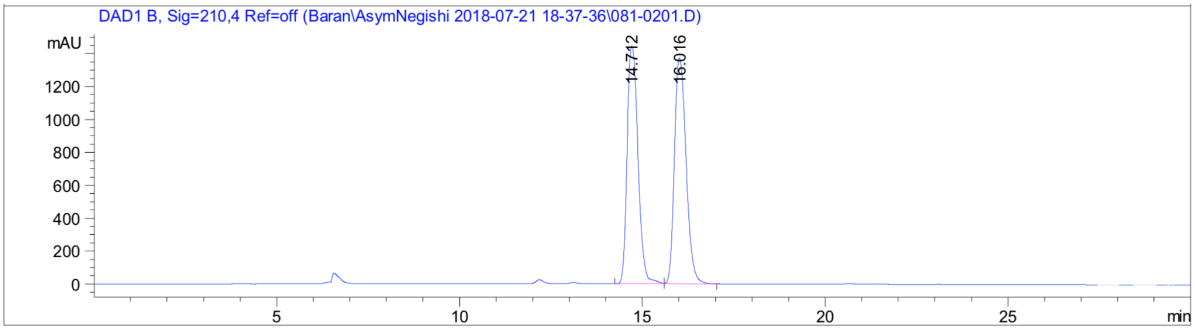

Enantioenriched **62:**

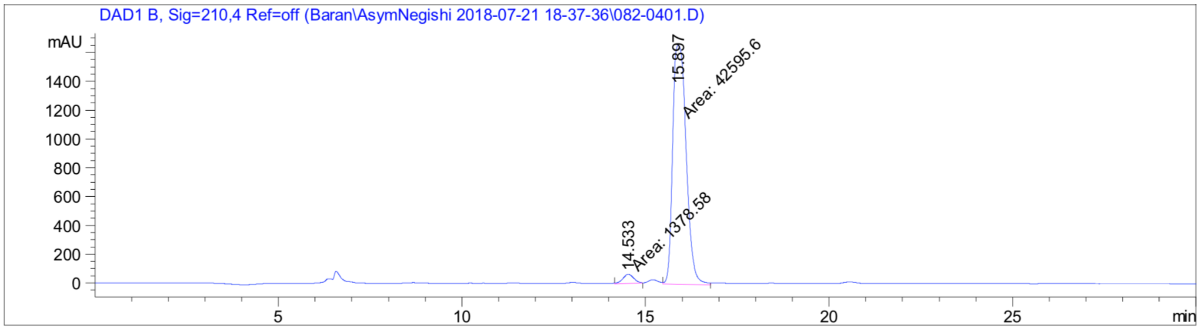

Signal 2: DAD1 B, Sig=210,4 Ref=off

Peak #	RetTime [min]	Type	Width [min]	Area [mAU*s]	Height [mAU]	Area %
1	14.712	BV	0.3251	2.98487e4	1444.40845	49.5122
2	16.016	VB	0.3488	3.04369e4	1371.92883	50.4878

Signal 2: DAD1 B, Sig=210,4 Ref=off

Peak #	RetTime [min]	Type	Width [min]	Area [mAU*s]	Height [mAU]	Area %
1	14.533	MM	0.3551	1378.57922	64.69947	3.1350
2	15.897	MM	0.4274	4.25956e4	1661.17053	96.8650

Compound 63

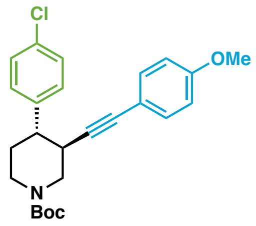

***tert*-butyl (3*S*,4*S*)-4-(4-chlorophenyl)-3-((4-methoxyphenyl)ethynyl)piperidine-1-carboxylate (63)**

Following General Procedure **C6** on 0.1 mmol scale with **B14**.Purification by pTLC (silica, 10:1 Hexanes:EtOAc) afforded 19.5 mg (46%) of the title compound **63**.

Physical State: colorless oil

^{1}H NMR (600 MHz, $CDCl_3$, both rotamers): δ 7.29 (d, J = 8.5 Hz, 2H), 7.20 (d, J = 8.5 Hz, 2H), 7.13 (d, J = 8.3 Hz, 2H), 6.75 (d, J = 8.9 Hz, 2H), 4.46 – 4.27 (m, 2H), 3.77 (s, 3H), 2.82 (s, 2H), 2.67 (d, J = 10.1 Hz, 2H), 1.86 – 1.78 (m, 1H), 1.69 (s, 1H), 1.50 (s, 9H) ppm.

^{13}C NMR (151 MHz, $CDCl_3$, both rotamers): δ 159.4, 154.5, 142.1, 133.0, 132.5, 129.1, 128.6, 115.3, 113.9, 87.3, 83.5, 80.1, 55.4, 49.3, 48.5, 43.9, 36.3, 32.7, 28.6 ppm.

HRMS (ESI-TOF): calc'd for $C_{25}H_{29}ClNO_3$ $[M+H]^+$: 426.1836; found 426.1837.

TLC: R_f = 0.3 (6:1 Hexanes:EtOAc).

$[\alpha]_D^{20}$ = 34.1 (c = 0.25, $CHCl_3$).

Chiral HPLC: Chiralpak IC, 4.6 x 250 mm; 10/90 *i*PrOH/hexanes, 0.5 mL/min, 254 nm; t_R (minor) = 13.8 min, t_R (major) = 15.9 min, 96% *ee*.

Racemic:

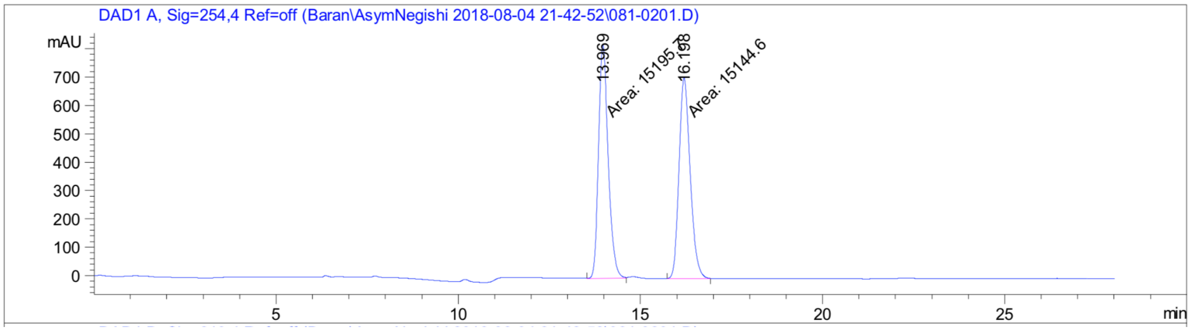

Enantioenriched **63:**

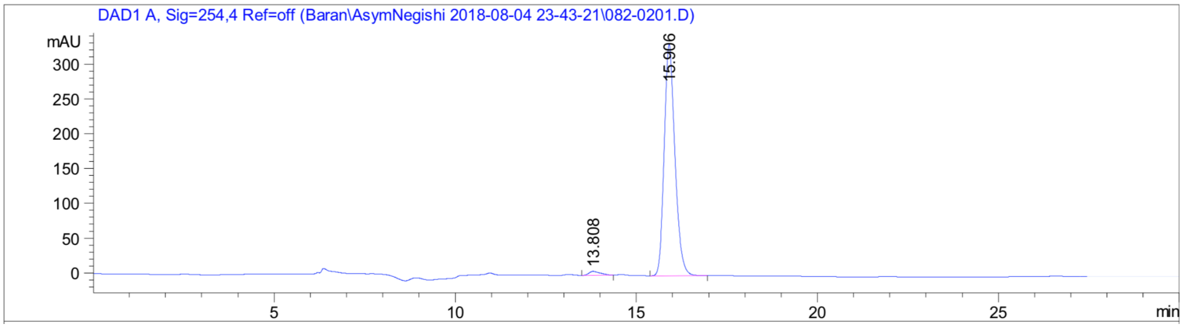

Signal 1: DAD1 A, Sig=254,4 Ref=off

Peak #	RetTime [min]	Type	Width [min]	Area [mAU*s]	Height [mAU]	Area %
1	13.969	MM	0.3073	1.51957e4	824.02411	50.0843
2	16.198	MM	0.3557	1.51446e4	709.60089	49.9157

Signal 1: DAD1 A, Sig=254,4 Ref=off

Peak #	RetTime [min]	Type	Width [min]	Area [mAU*s]	Height [mAU]	Area %
1	13.808	BB	0.3157	129.20750	5.84851	1.8520
2	15.906	BB	0.3166	6847.51367	332.00601	98.1480

Compound 64

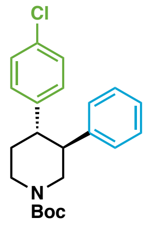

***tert*-butyl (3*S*,4*S*)-4-(4-chlorophenyl)-3-phenylpiperidine-1-carboxylate (64)**

Following General Procedure **C4** on 0.1 mmol scale with **B14**, $NiCl_2$•glyme (30 mol%), di*t*BuBipy (40 mol%), and Zinc reagent **SI-35** (2.0 equiv.). Purification by pTLC (silica, 4:1 Hexanes:EtOAc) afforded 32.8 mg (88%) of the title compound **64.**

Physical State: colorless oil

^{1}H NMR (600 MHz, $CDCl_3$, both rotamers) δ 7.16 (t, *J* = 7.5 Hz, 2H), 7.11 – 7.07 (m, 3H), 7.04 (d, *J* = 7.0 Hz, 2H), 6.95 (d, *J* = 8.3 Hz, 2H), 4.31 (s, 2H), 2.95 – 2.82 (m, 4H), 1.88 (d, *J* = 12.3 Hz, 1H), 1.79 – 1.72 (m, 1H), 1.49 (s, 9H) ppm.

^{13}C NMR (151 MHz, $CDCl_3$, both rotamers) δ 154.8, 142.3, 141.0, 131.8, 128.9, 128.5, 127.9, 126.7, 79.9, 51.0, 48.9, 48.2, 44.3, 34.2, 28.6 ppm.

HRMS (ESI-TOF): calc'd for $C_{22}H_{27}ClNO_2$ $[M+H]^+$: 372.1730; found 372.1734.

TLC: R_f = 0.4 (8:1 Hexanes:EtOAc).

$[\alpha]_D^{20}$ = 41.4 (*c* = 1.0, $CHCl_3$).

Chiral HPLC: Chiralpak IC, 4.6 x 250 mm; 10/90 *i*PrOH/hexanes, 1 mL/min, 210 nm; t_R (minor) = 9.6 min, t_R (major) = 10.4 min, 96% *ee*.

Racemic:

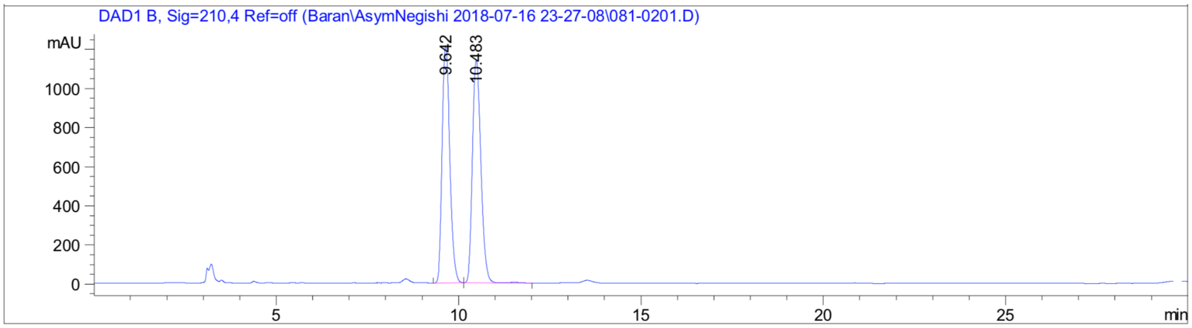

Enantioenriched **64:**

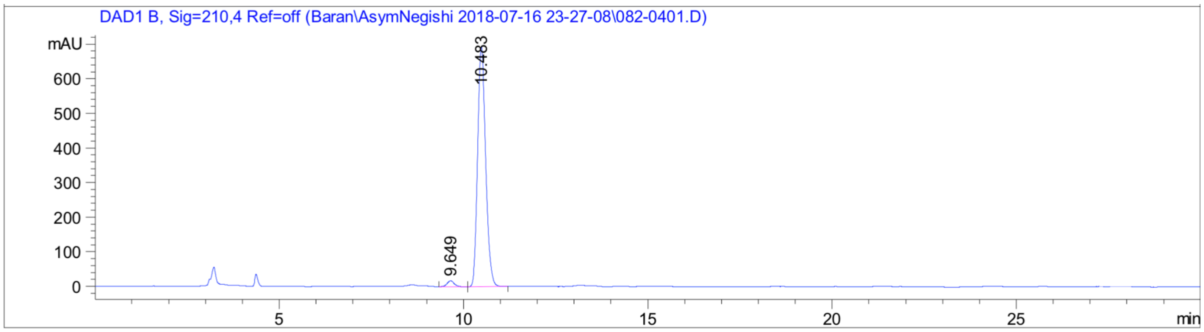

Signal 2: DAD1 B, Sig=210,4 Ref=off

Peak #	RetTime [min]	Type	Width [min]	Area [mAU*s]	Height [mAU]	Area %
1	9.642	BB	0.2290	1.77629e4	1212.16760	49.0036
2	10.483	BV R	0.2547	1.84853e4	1138.40784	50.9964

Signal 2: DAD1 B, Sig=210,4 Ref=off

Peak #	RetTime [min]	Type	Width [min]	Area [mAU*s]	Height [mAU]	Area %
1	9.649	BB	0.2077	236.43112	17.27503	2.1923
2	10.483	BB	0.2356	1.05482e4	692.79028	97.8077

Compound 65

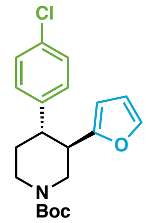

***tert*-butyl (3*R*,4*S*)-4-(4-chlorophenyl)-3-(furan-2-yl)piperidine-1-carboxylate (65)**

Following General Procedure **C1** on 0.1 mmol scale with **B15**, $NiCl_2$•$6H_2O$ (30 mol%), BPhen (30 mol%), and boronic acid **SI-20** (3.0 equiv.). Purification by pTLC (silica, 6:1 Hexanes:EtOAc) afforded 27.1 mg (75%) of the title compound **65**.

Physical State: colorless oil

^{1}H NMR (600 MHz, $CDCl_3$, both rotamers): δ 7.20 (d, J = 1.8 Hz, 1H), 7.17 – 7.15 (m, 2H), 6.99 (d, J = 8.5 Hz, 2H), 6.11 (s, 1H), 5.76 (d, J = 3.2 Hz, 1H), 4.32 (s, 2H), 3.01 – 2.85 (m, 4H), 1.86 – 1.70 (m, 2H), 1.49 (s, 9H) ppm.

^{13}C NMR (151 MHz, $CDCl_3$, both rotamers): δ 154.7, 154.5, 142.4, 141.2, 132.1, 128.7, 128.6, 110.0, 106.5, 80.0, 48.9, 47.0, 44.2, 42.4, 33.7, 28.6 ppm.

HRMS (ESI-TOF): calc'd for $C_{20}H_{25}ClNO_3$ $[M+H]^+$: 362.1523; found 362.1519.

TLC: R_f = 0.4 (6:1 Hexanes:EtOAc).

$[\alpha]_D^{20}$ = 17.1 (c = 0.5, $CHCl_3$).

Chiral HPLC: Chiralpak IC, 4.6 x 250 mm; 10/90 *i*PrOH/hexanes, 0.5 mL/min, 210 nm; t_R (minor) = 12.1 min, t_R (major) = 16.0 min, 96% *ee*.

Racemic:

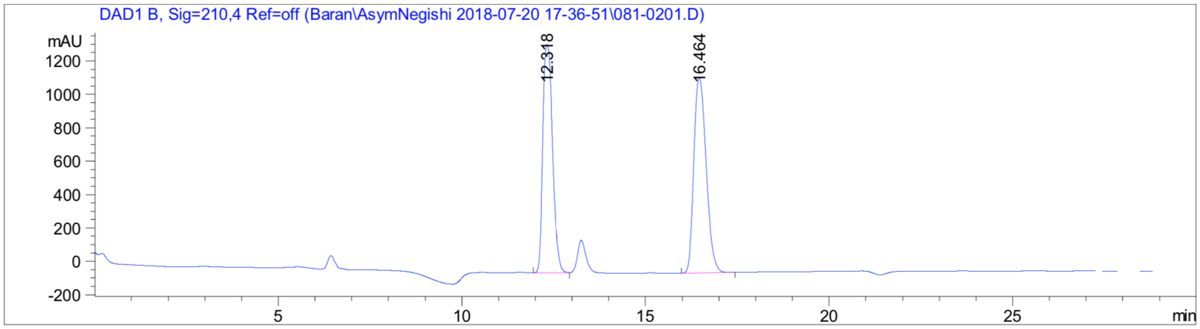

Enantioenriched **65:**

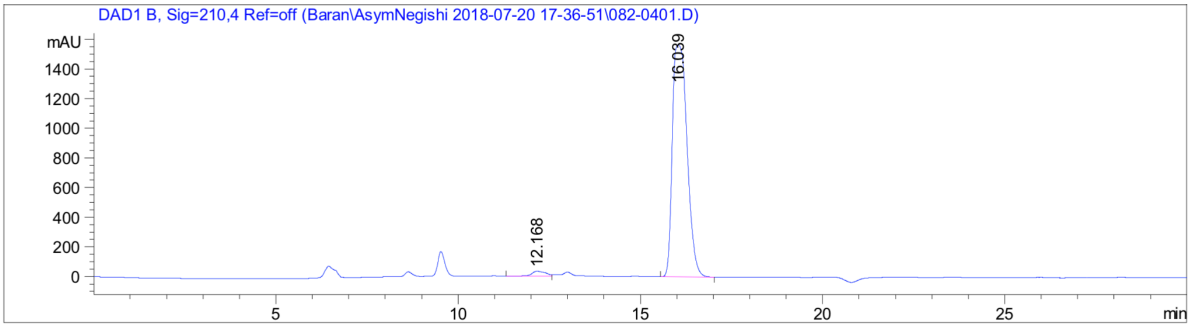

Signal 2: DAD1 B, Sig=210,4 Ref=off

Peak #	RetTime [min]	Type	Width [min]	Area [mAU*s]	Height [mAU]	Area %
1	12.318	BV	0.2909	2.51932e4	1367.85291	48.0279
2	16.464	BB	0.3680	2.72621e4	1169.43884	51.9721

Signal 2: DAD1 B, Sig=210,4 Ref=off

Peak #	RetTime [min]	Type	Width [min]	Area [mAU*s]	Height [mAU]	Area %
1	12.168	BV	0.3780	894.88928	33.45746	1.9937
2	16.039	BB	0.4439	4.39906e4	1565.07227	98.0063

Experimental Procedures and Characterization Data for Morpholine Scaffolds (Figure 3–e)

Compound SI-3

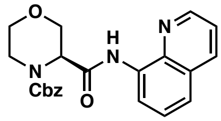

benzyl (*S*)-3-(quinolin-8-ylcarbamoyl)morpholine-4-carboxylate (SI-3)

Following General Procedure **A4** on 10.0 mmol scale. Purification by flash column chromatography (silica, 5:1 Hexanes:EtOAc) afforded 2.89 g (74%) of the title compound **SI-3**.

Physical State: yellow oil.

^{1}H NMR (600 MHz, $CDCl_3$, both rotamers): δ 10.44 (s, 1H), 8.89 – 8.61 (m, 2H), 8.16 (dd, J = 8.2, 1.7 Hz, 1H), 7.56 – 7.42 (m, 7H), 7.37 – 7.13 (m, 2H), 5.39 – 5.25 (m, 2H), 4.91 – 4.69 (m, 2H), 4.15 – 3.92 (m, 2H), 3.65 (d, J = 86.8 Hz, 3H) ppm.

^{13}C NMR (151 MHz, $CDCl_3$, both rotamers): δ 167.6, 156.3, 155.6, 148.6, 138.7, 136.4, 136.1, 134.0, 128.7, 128.3, 128.1, 128.0, 127.4, 122.1, 121.8, 116.8, 68.2, 67.0, 66.6, 56.7, 56.1, 42.2, 41.6 ppm.

HRMS (ESI-TOF): calc'd for $C_{22}H_{22}N_3O_4$ $[M+H]^+$: 392.1610; found 392.1613.

TLC: R_f = 0.3 (3:1 Hexanes:EtOAc).

$[\alpha]_D^{20}$ = -118.7 (c = 1.0, $CHCl_3$).

Chiral HPLC: Chiralpak IA, 4.6 x 250 mm; 20/80 *i*PrOH/hexanes, 1 mL/min, 210 nm; t_R (minor) = 23.5 min, t_R (major) = 15.5 min, 98% *ee*.

Racemic:

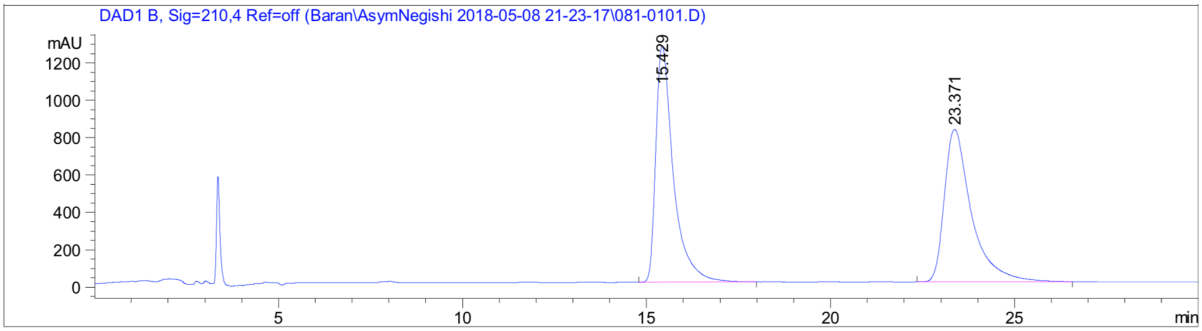

Enantioenriched **SI-3:**

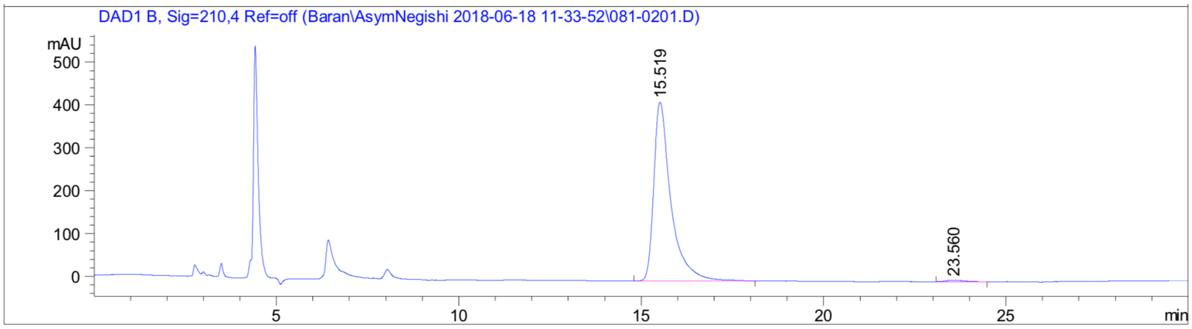

Signal 2: DAD1 B, Sig=210,4 Ref=off

Peak #	RetTime [min]	Type	Width [min]	Area [mAU*s]	Height [mAU]	Area %
1	15.429	BB	0.4923	4.21515e4	1266.32910	50.2469
2	23.371	BB	0.7402	4.17372e4	816.74042	49.7531

Signal 2: DAD1 B, Sig=210,4 Ref=off

Peak #	RetTime [min]	Type	Width [min]	Area [mAU*s]	Height [mAU]	Area %
1	15.519	BB	0.4765	1.36810e4	417.28632	99.0833
2	23.560	BB	0.4666	126.57509	3.24821	0.9167

Compound SI-4

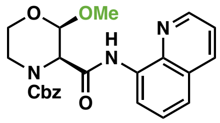

benzyl (2*R*,3*S*)-2-methoxy-3-(quinolin-8-ylcarbamoyl)morpholine-4-carboxylate (SI-5)

Following General Procedure **A4** on 1.68 mmol scale with **SI-3**. Purification by flash column chromatography (silica, 2:1 Hexanes:EtOAc) afforded 297 mg (51%) of the title compound **SI-4**.

Physical State: yellow oil.

^{1}H NMR (600 MHz, $CDCl_3$, both rotamers): δ 10.52 (s, 1H), 8.84 – 8.58 (m, 2H), 8.16 (d, *J* = 8.2 Hz, 1H), 7.55 (d, *J* = 4.4 Hz, 2H), 7.47 – 7.14 (m, 6H), 5.49 – 5.22 (m, 3H), 5.23 – 5.03 (m, 1H)), 4.26 – 3.98 (m, 2H), 3.63 – 3.40 (m, 5H) ppm.

^{13}C NMR (151 MHz, $CDCl_3$, both rotamers): δ 166.2, 156.5, 156.1, 148.8, 148.6, 138.7, 136.4, 136.3, 133.8, 133.7, 128.7, 128.5, 128.3, 128.1, 128.0, 127.7, 127.4, 127.4 – 127.3 (m), 122.3, 122.2, 121.8, 121.8, 116.8, 96.1, 95.6, 68.2, 68.1, 59.9, 59.1, 57.3, 57.2, 55.3, 55.1, 53.6, 41.4, 40.7 ppm.

HRMS (ESI-TOF): calc'd for $C_{23}H_{24}N_3O_5$ [M+H]$^+$: 422.1716; found 422.1714.

TLC: R_f = 0.2 (1:1 Hexanes:EtOAc).

$[\alpha]_D^{20}$ = -31.6 (*c* = 1.0, $CHCl_3$).

Chiral HPLC: Chiralpak IA, 4.6 x 250 mm; 30/70 *i*PrOH/hexanes, 1 mL/min, 254 nm; t_R (minor) = 13.1 min, t_R (major) = 9.2 min, 97% *ee*.

Racemic:

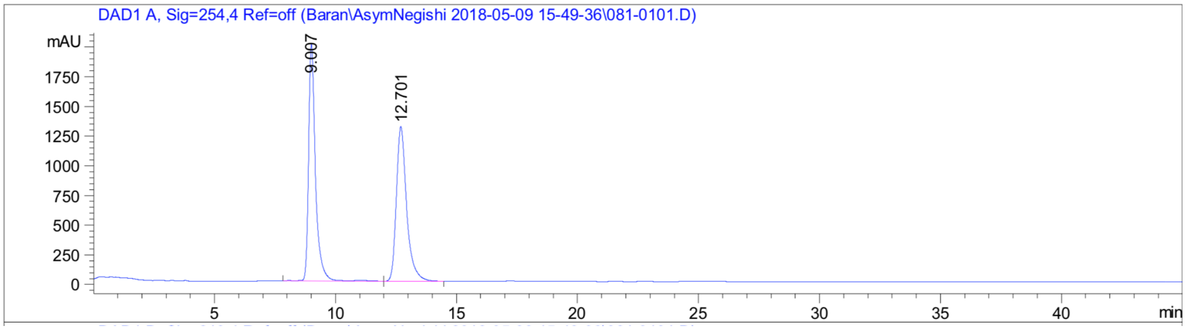

Enantioenriched **SI-4:**

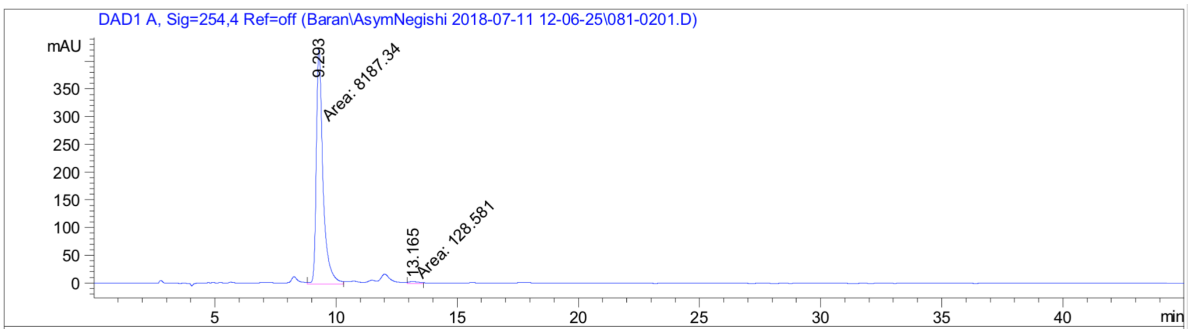

Signal 1: DAD1 A, Sig=254,4 Ref=off

Peak #	RetTime [min]	Type	Width [min]	Area [mAU*s]	Height [mAU]	Area %
1	9.007	VV R	0.2935	3.95174e4	1991.41882	50.6407
2	12.701	BB	0.4452	3.85176e4	1301.78955	49.3593

Signal 1: DAD1 A, Sig=254,4 Ref=off

Peak #	RetTime [min]	Type	Width [min]	Area [mAU*s]	Height [mAU]	Area %
1	9.293	MM	0.3216	8187.34424	424.35239	98.4538
2	13.165	MM	0.5276	128.58076	4.06177	1.5462

Compound B16

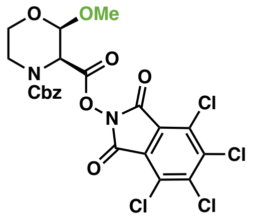

4-benzyl 3-(4,5,6,7-tetrachloro-1,3-dioxoisoindolin-2-yl) (2*R*,3*S*)-2-methoxymorpholine-3,4-dicarboxylate (B16)

Following General Procedure **A4** on 1.73 mmol scale. Purification by flash column chromatography (silica, 2:1 Hexanes:EtOAc) afforded 730 mg (73%) of the title compound **B16**.

Physical State: yellow oil.

^{1}H NMR (600 MHz, $CDCl_3$, both rotamers): δ 7.41 – 7.29 (m, 5H), 5.31 – 5.10 (m, 4H), 4.06 – 3.87 (m, 2H), 3.69 – 3.39 (m, 2H), 3.46 – 3.44 (m, 3H) ppm.

^{13}C NMR (151 MHz, $CDCl_3$, both rotamers): δ 165.2, 165.1, 157.1, 156.0, 155.4, 141.4, 141.3, 136.0, 136.0, 130.8, 130.8, 128.7, 128.4, 128.3, 128.3 128.2, 128.1, 124.7, 95.7, 95.1, 68.3, 68.3, 57.7, 57.5, 56.9, 56.3, 55.4, 55.3, 40.9, 40.2 ppm.

HRMS (ESI-TOF): calc'd for $C_{22}H_{17}Cl_4N_2O_8$ [M+H]$^+$: 576.9739; found 576.9741.

TLC: R_f = 0.3 (1:1 Hexanes:EtOAc).

$[\alpha]_D^{20}$ = -9.2 (*c* = 1.0, $CHCl_3$).

Chiral HPLC: Chiralpak IC, 4.6 x 250 mm; 45/55 *i*PrOH/hexanes, 1 mL/min, 254 nm; t_R (minor) = 22.4 min, t_R (major) = 31.7 min, >99% *ee*.

Racemic:

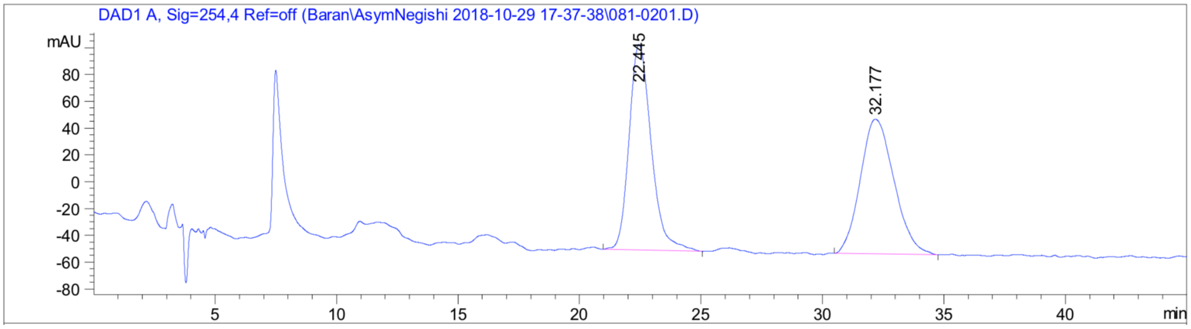

Enantioenriched **B16:**

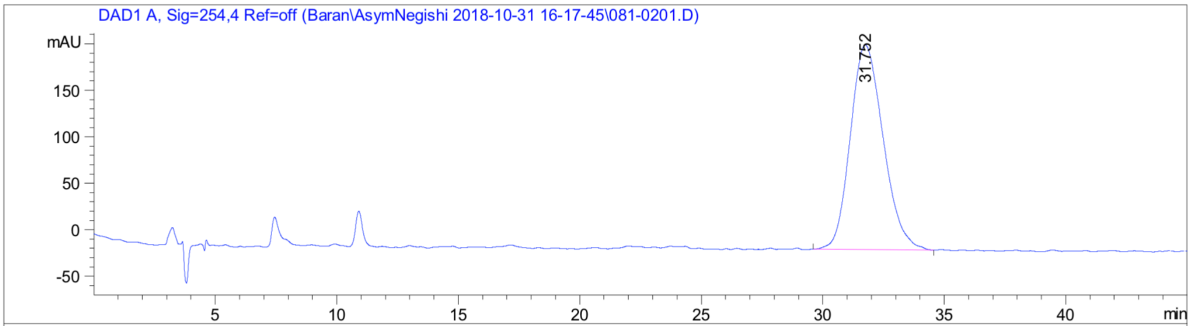

Signal 1: DAD1 A, Sig=254,4 Ref=off

Peak #	RetTime [min]	Type	Width [min]	Area [mAU*s]	Height [mAU]	Area %
1	22.445	BB	0.9743	9761.04199	153.40486	50.2138
2	32.177	BB	1.4082	9677.91309	100.43898	49.7862

Signal 1: DAD1 A, Sig=254,4 Ref=off

Peak #	RetTime [min]	Type	Width [min]	Area [mAU*s]	Height [mAU]	Area %
1	31.752	BB	1.4007	2.07725e4	220.18013	100.0000

Compound 66

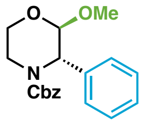

benzyl (2*R*,3*S*)-2-methoxy-3-phenylmorpholine-4-carboxylate (66)

Following General Procedure **C4** on 0.1 mmol scale with **B16**, $NiCl_2$•glyme (30 mol%), di^{t}BuBipy (40 mol%), and Zinc reagent **SI-35** (2.0 equiv.). Purification by pTLC (silica, 4:1 Hexanes:EtOAc) afforded 19.9 mg (61%) of the title compound **66**.

Physical State: colorless oil

^{1}H NMR (600 MHz, $CDCl_3$, both rotamers) δ 7.52 – 7.28 (m, 10H), 5.25 – 5.13 (m, 3H), 4.98 (s, 1H), 4.04 (t, J = 11.6 Hz, 1H), 3.88 (s, 1H), 3.60 (d, J = 9.3 Hz, 1H), 3.44 (s, 3H), 3.20 (t, J = 14.4 Hz, 1H) ppm.

^{13}C NMR (151 MHz, $CDCl_3$, both rotamers) δ 156.0, 137.4, 136.7, 128.7, 128.6, 128.2, 128.1, 128.0, 127.8, 98.0, 67.6, 58.3, 57.1, 54.8, 38.9 ppm.

HRMS (ESI-TOF): calc'd for $C_{19}H_{22}NO_4$ $[M+H]^+$: 328.1549; found 328.1544.

TLC: R_f = 0.3 (4:1 Hexanes:EtOAc).

$[\alpha]_D^{20}$ = -28.6 (c = 1.0, $CHCl_3$).

Chiral HPLC: Chiralpak IC, 4.6 x 250 mm; 10/90 *i*PrOH/hexanes, 1 mL/min, 210 nm; t_R (minor) = 9.6 min, t_R (major) = 10.4 min, 96% *ee*.

Racemic:

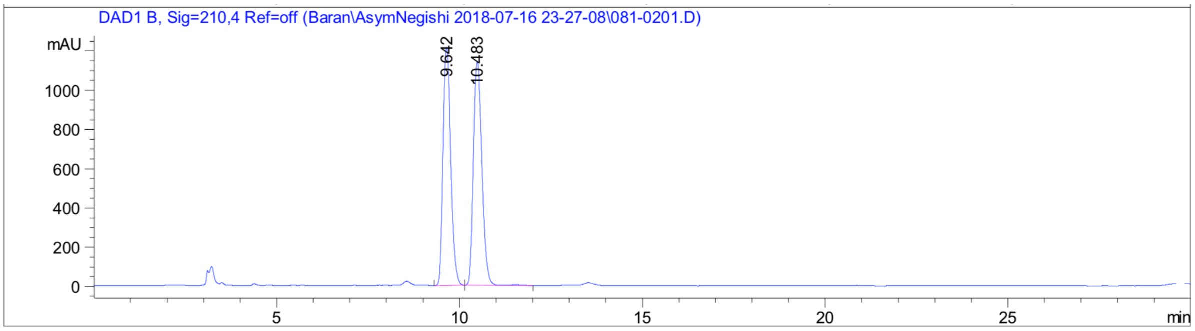

Enantioenriched **66:**

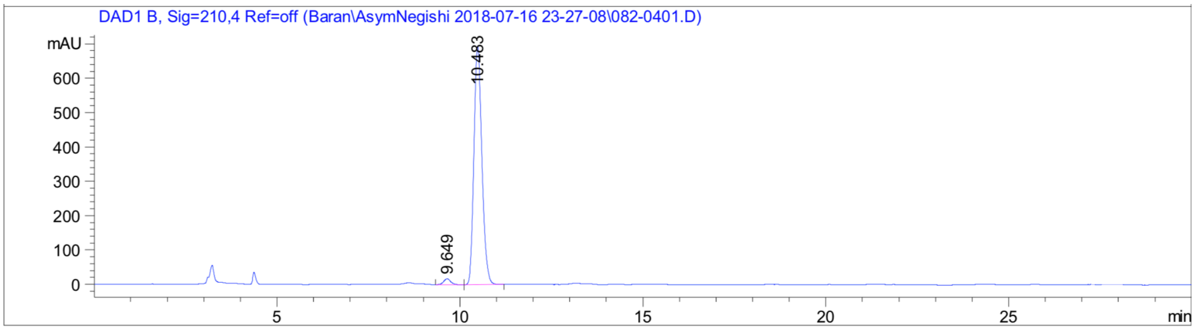

Signal 2: DAD1 B, Sig=210,4 Ref=off

Peak #	RetTime [min]	Type	Width [min]	Area [mAU*s]	Height [mAU]	Area %
1	9.642	BB	0.2290	1.77629e4	1212.16760	49.0036
2	10.483	BV R	0.2547	1.84853e4	1138.40784	50.9964

Signal 2: DAD1 B, Sig=210,4 Ref=off

Peak #	RetTime [min]	Type	Width [min]	Area [mAU*s]	Height [mAU]	Area %
1	9.649	BB	0.2077	236.43112	17.27503	2.1923
2	10.483	BB	0.2356	1.05482e4	692.79028	97.8077

Compound 67

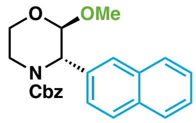

benzyl (2*R*,3*S*)-2-methoxy-3-(naphthalen-2-yl)morpholine-4-carboxylate (67)

Following General Procedure **C1** on 0.088 mmol scale with **B16**, $NiCl_2$•$6H_2O$ (30 mol%), BPhen (30 mol%), and boronic acid **SI-11** (3.0 equiv.). Purification by pTLC (silica, 6:1 Hexanes:EtOAc) afforded 20.1 mg (60%) of the title compound **67**.

Physical State: colorless oil

^{1}H NMR (600 MHz, $CDCl_3$, both rotamers): δ 7.94 (s, 1H), 7.81 (d, *J* = 19.5 Hz, 3H), 7.56 (s, 1H), 7.49 (d, *J* = 9.3 Hz, 2H), 7.36 – 7.31 (m, 5H), 5.40 (s, 1H), 5.30 – 5.08 (m, 3H), 4.09 (t, *J* = 11.8 Hz, 1H), 3.92 (s, 1H), 3.65 (d, *J* = 9.6 Hz, 1H), 3.49 (s, 3H), 3.24 (td, *J* = 13.4, 3.7 Hz, 1H) ppm.

^{13}C NMR (151 MHz, $CDCl_3$, both rotamers): δ 156.0, 136.6, 134.8, 133.2, 132.9, 128.6, 128.4, 128.3, 128.2, 128.1, 127.7, 127.1, 126.3, 126.3, 97.9, 67.7, 58.4, 57.0, 54.9, 39.0 ppm.

HRMS (ESI-TOF): calc'd for $C_{23}H_{24}NO_4$ $[M+H]^+$: 378.1705; found 378.1704.

TLC: R_f = 0.4 (4:1 Hexanes:EtOAc).

$[\alpha]_D^{20}$ = -68.9 (*c* = 1.0, $CHCl_3$).

Chiral HPLC: Chiralpak IC, 4.6 x 250 mm; 10/90 *i*PrOH/hexanes, 1 mL/min, 210 nm; t_R (minor) = 12.1 min, t_R (major) = 13.2 min, 97% *ee*.

Racemic:

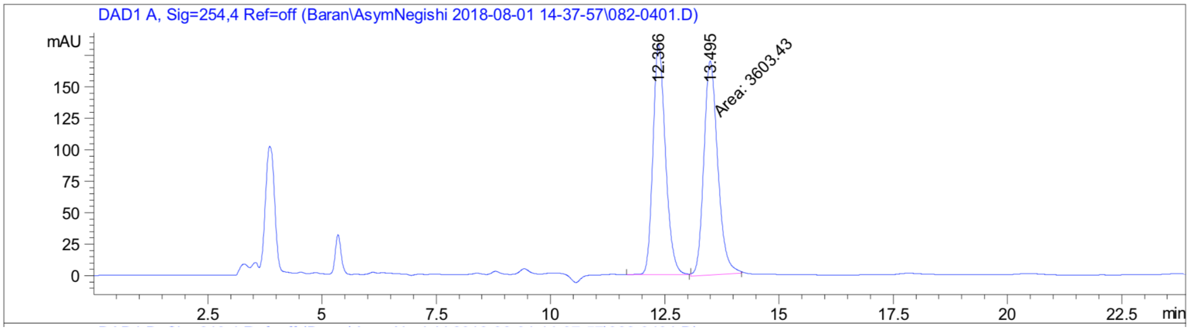

Enantioenriched **67:**

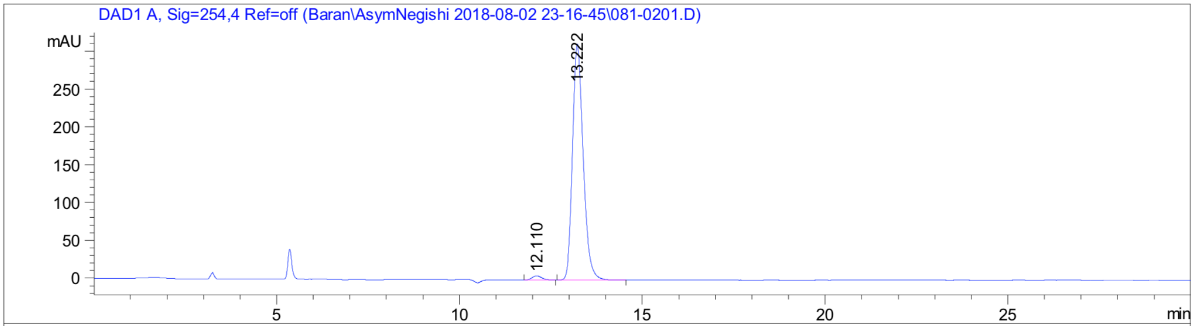

Signal 1: DAD1 A, Sig=254,4 Ref=off

Peak #	RetTime [min]	Type	Width [min]	Area [mAU*s]	Height [mAU]	Area %
1	12.366	BV	0.2919	3479.83203	182.94949	49.1275
2	13.495	MM	0.3530	3603.42822	170.14580	50.8725

Signal 1: DAD1 A, Sig=254,4 Ref=off

Peak #	RetTime [min]	Type	Width [min]	Area [mAU*s]	Height [mAU]	Area %
1	12.110	BB	0.2719	98.36753	5.57500	1.5255
2	13.222	BB	0.3139	6349.83545	311.41013	98.4745

Compound 68

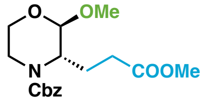

benzyl (2*R*,3*S*)-2-methoxy-3-(3-methoxy-3-oxopropyl)morpholine-4-carboxylate (68)

Following General Procedure **C8** on 0.095 mmol scale with methyl acrylate. Purification by pTLC (silica, 3:1 Hexanes:EtOAc) afforded 12.5 mg (39%) of the title compound **68**.

Physical State: colorless oil

^{1}H NMR (600 MHz, $CDCl_3$, both rotamers): δ 7.37 – 7.31 (m, 5H), 5.16 – 5.11 (m, 2H), 4.44 – 4.38 (m, 1H), 4.18 – 4.09 (m, 1H), 3.94 – 3.80 (m, 2H), 3.64 – 3.47 (m, 4H), 3.35 (s, 3H), 3.22 – 3.12 (m, 1H), 2.37 – 2.28 (m, 2H), 2.21 – 2.12 (m, 1H), 1.87 – 1.81 (m, 1H) ppm.

^{13}C NMR (151 MHz, $CDCl_3$, both rotamers): δ 173.6, 173.3, 156.1, 155.9, 136.7, 128.6, 128.2, 128.1, 127.9, 98.6, 98.3, 67.5, 57.9, 57.8, 54.9, 54.7, 54.2, 53.6, 51.8, 38.6, 37.9, 30.4, 30.2, 23.8 ppm.

HRMS (ESI-TOF): calc'd for $C_{17}H_{24}NO_6$ $[M+H]^+$: 338.1604; found 338.1603.

TLC: R_f = 0.3 (4:1 Hexanes:EtOAc).

$[\alpha]_D^{20}$ = 16.2 (*c* = 0.33, $CHCl_3$).

Chiral HPLC: Chiralpak IA, 4.6 x 250 mm; 10/90 *i*PrOH/hexanes, 0.5 mL/min, 210 nm; t_R (minor) = 25.4 min, t_R (major) = 26.7 min, 97% *ee*.

Racemic:

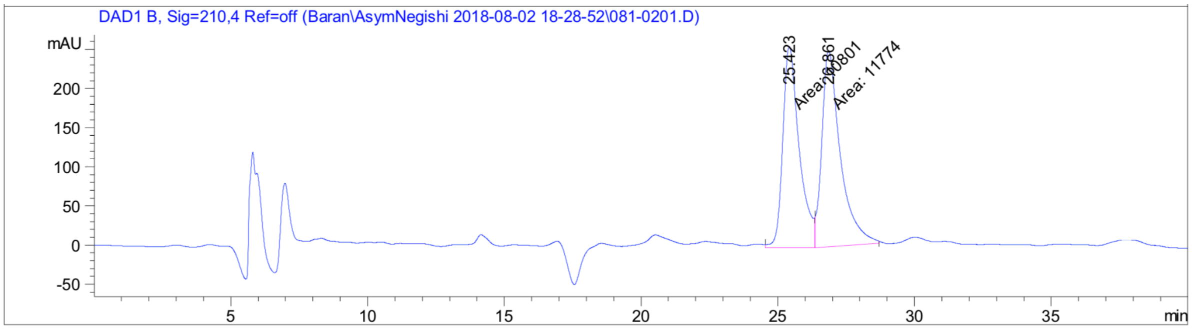

Enantioenriched **68:**

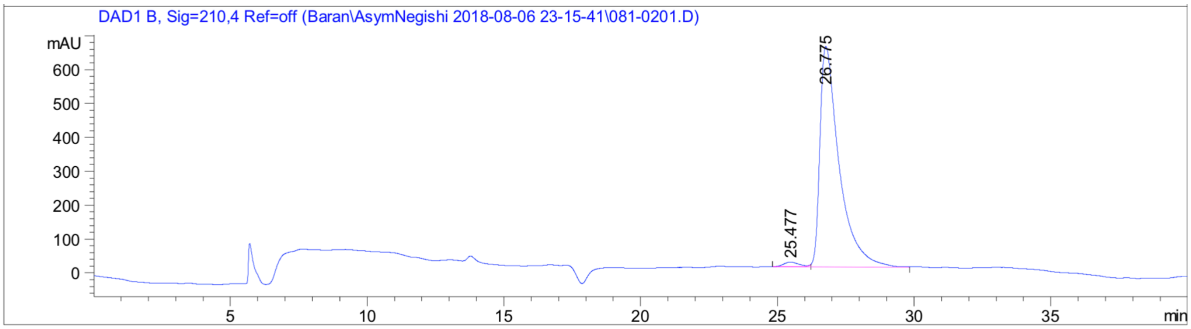

Signal 2: DAD1 B, Sig=210,4 Ref=off

Peak #	RetTime [min]	Type	Width [min]	Area [mAU*s]	Height [mAU]	Area %
1	25.423	MM	0.6993	1.08010e4	257.40671	47.8450
2	26.861	MM	0.7828	1.17740e4	250.69632	52.1550

Signal 2: DAD1 B, Sig=210,4 Ref=off

Peak #	RetTime [min]	Type	Width [min]	Area [mAU*s]	Height [mAU]	Area %
1	25.477	BV E	0.4636	526.85687	13.85538	1.6308
2	26.775	VB R	0.7064	3.17800e4	650.05908	98.3692

Experimental Procedures and Characterization Data for hit-to-lead candidates and late-stage intermediates (Figure 4)

Compound SI-42

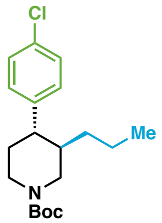

***tert*-butyl (3*R*,4*S*)-4-(4-chlorophenyl)-3-propylpiperidine-1-carboxylate (SI-42)**

Following General Procedure **C2** on 0.2 mmol scale with **B14**, $NiCl_2$•glyme (30 mol%), di^{t}BuBipy (40 mol%), and Zinc reagent **SI-51** (2.0 equiv.). Purification by pTLC (silica, 10:1 Hexanes: EtOAc) afforded 41.1 mg (61%) of the title compound **SI-42**.

Physical State: colorless oil

^{1}H NMR (600 MHz, $CDCl_3$, both rotamers): δ δ 7.27 (d, J = 5.5 Hz, 2H), 7.08 (d, J = 8.4 Hz, 2H), 4.28 – 4.19 (m, 2H), 2.75 (s, 1H), 2.38 (s, 1H), 2.26 (td, J = 11.6, 3.8 Hz, 1H), 1.71 (d, J = 12.0 Hz, 1H), 1.61 – 1.59 (m, 2H), 1.49 (s, 9H), 1.34 – 1.28 (m, 1H), 1.14 – 1.05 (m, 2H), 0.93 – 0.84 (m, 1H), 0.75 (t, J = 7.0 Hz, 3H) ppm.

^{13}C NMR (151 MHz, $CDCl_3$, both rotamers): δ 154.9, 143.3, 132.0, 129.0, 128.8, 79.6, 49.4, 49.0, 44.6, 40.9, 34.8, 33.5, 28.6, 19.6, 14.3 ppm.

HRMS (ESI-TOF): calc'd for $C_{14}H_{19}ClN$ [M-Boc + H]$^+$: 238.1363; found 238.1362.

TLC: R_f = 0.3 (6:1 hexanes:EtOAc).

$[\alpha]_D^{20}$ = 11.6 (c = 0.5, $CHCl_3$).

Chiral HPLC: Chiralpak IC, 4.6 x 250 mm; 10/90 *i*PrOH/hexanes, 0.5 mL/min, 210 nm; *t*R (minor) = 9.7 min, *t*R (major) = 11.0 min, 97% *ee*.

Racemic:

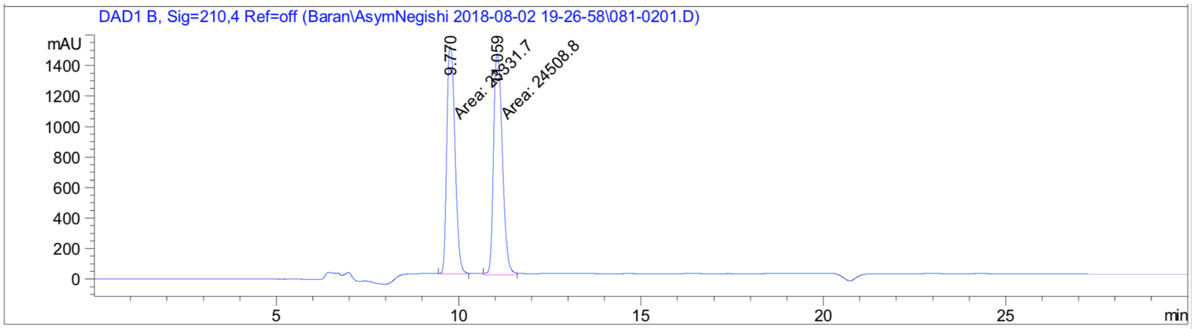

Enantioenriched **SI-42**:

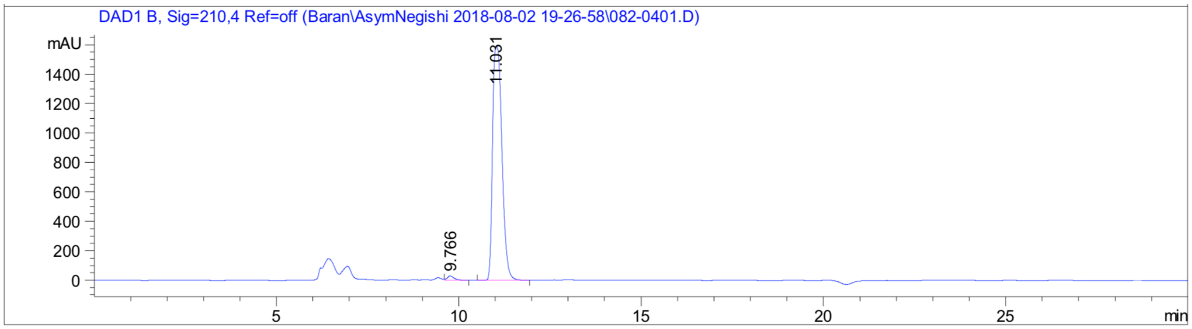

Signal 2: DAD1 B, Sig=210,4 Ref=off

Peak #	RetTime [min]	Type	Width [min]	Area [mAU*s]	Height [mAU]	Area %
1	9.770	MM	0.2609	2.33317e4	1490.33911	48.7698
2	11.059	MM	0.2817	2.45088e4	1450.12830	51.2302

Signal 2: DAD1 B, Sig=210,4 Ref=off

Peak #	RetTime [min]	Type	Width [min]	Area [mAU*s]	Height [mAU]	Area %
1	9.766	VB	0.1911	383.43439	29.21444	1.3378
2	11.031	BB	0.2818	2.82785e4	1587.65710	98.6622

Compound 69

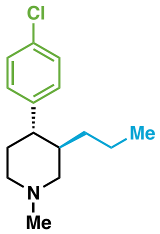

(3*R*,4*S*)-4-(4-chlorophenyl)-1-methyl-3-propylpiperidine (69)

To a culture tube was added **SI-42** (29.0 mg, 0.086 mmol, 1 equiv.), evacuated and backfilled with argon three times and followed by adding 2 mL HCl in dioxane (4 M). Then the solution was stirred at room temperature for 6 hours. Upon completion, the solvent was removed *in vacuo*. To the same culture were added 36.5% HCHO in H_2O (10.7 mg, 0.129 mmol, 1.5 equiv.), $NaBH(OAc)_3$ (36.5 mg, 0.172 mmol, 2 equiv.), HOAc (10.3 mg, 0.172 mmol, 2 equiv.) and CH_2Cl_2 (2 mL). Then the resulting mixture was stirred at room temperature for 12h. Upon completion, a saturated solution of aq. $NaHCO_3$ (10 mL) was added to quench the reaction. The mixture was then extracted with EtOAc (3x). The combined organic layers were dried over Na_2SO_4, filtered and purified by PTLC (silica, 10:1 EtOAc:MeOH) afforded 16.2 mg (75%) of the title compound **69**.

Physical State: colorless oil

^{1}H NMR (600 MHz, $CDCl_3$) δ 7.25 (d, J = 8.2 Hz, 2H), 7.10 (d, J = 8.1 Hz, 2H), 3.07 (ddd, J = 11.3, 3.7, 1.7 Hz, 1H), 2.94 (ddt, J = 11.4, 4.0, 2.1 Hz, 1H), 2.33 (s, 3H), 2.09 (td, J = 11.3, 4.5 Hz, 1H), 1.99 (td, J = 11.5, 3.2 Hz, 1H), 1.84 – 1.74 (m, 3H), 1.67 (t, J = 11.1 Hz, 1H), 1.32 – 1.26 (m, 1H), 1.10 – 1.03 (m, 2H), 0.91 – 0.85 (m, 1H), 0.73 (t, J = 7.1 Hz, 3H) ppm.

^{13}C NMR (151 MHz, $CDCl_3$) δ 143.9, 131.8, 129.1, 128.7, 61.7, 56.4, 48.3, 46.6, 40.9, 35.1, 34.1, 19.8, 14.3 ppm.

HRMS (ESI-TOF): calc'd for $C_{15}H_{23}ClN$ $[M+H]^+$: 252.1519; found 252.1517.

TLC: R_f = 0.3 (10:1 EtOAc:MeOH).

$[\alpha]_D^{20}$ = 31.8 (c = 0.5, $CHCl_3$).

Compound SI-43

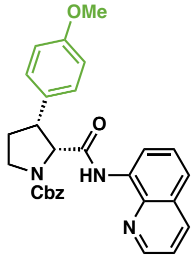

Benzyl (2*R*,3*R*)-3-(4-methoxyphenyl)-2-[(quinolin-8-yl)carbamoyl]pyrrolidine-1-carboxylate, Compound (SI-43)

A reaction tube was charged with benzyl (2*R*)-2-[(quinolin-8-yl)carbamoyl]pyrrolidine-1-carboxylate 1 (1.89 g, 5.00 mmol, 1.0 equiv), AgOAc (1.51 g, 9.00 mmol 1.8 equiv), 4-iodoanisole (2.12 g, 9.00 mmol, 1.8 equiv) and $Pd(OAc)_2$ (55.3 mg, 19.7 µmol, 5 mol%). The reaction vessel was purged with argon and sealed, then placed in an oil bath (preheated to 110 °C) for 14 hours. The reaction mixture was then allowed to cool to room temperature and EtOAc (30 mL) was added. The resulting solution was filtered through a pad of Celite®, eluting with further EtOAc (2 x 30 mL). The solvent was removed under reduced pressure, and the crude material was purified by flash column chromatography (silica, 7:3 Et_2O:Hexanes), affording arylated compound **SI-43** (2.22 g, 92%) as a white solid.

Physical state: White solid

m.p.:115-117 °C

^{1}H NMR (600 MHz, $CDCl_3$, both rotamers): δ 9.51 (s, 1H), 8.63 (s, 1H), 8.52 (d, *J* = 8.1 Hz, 1H), 8.08 (d, *J* = 8.3 Hz, 1H), 7.52 – 7.29 (m, 5H), 7.18 (m, 3H), 6.95 (t, *J* = 7.5 Hz, 2H), 6.60 (d, *J* = 8.2 Hz, 2H), 5.39 – 4.95 (m, 2H), 4.70 (d, *J* = 8.4 Hz, 1H), 4.11 (t, *J* = 7.3 Hz, 1H), 3.88 – 3.59 (m, 2H), 3.48 (s, 3H), 2.73 (m, 1H), 2.20 (m, 1H) ppm.

^{13}C NMR (151 MHz, $CDCl_3$, both rotamers): δ 168.9, 168.8, 158.2, 155.1, 154.7, 148.1, 148.0, 138.4, 136.9, 136.5, 136.1, 136.0, 134.0, 133.9, 129.1, 128.6, 128.5, 128.1, 128.1, 128.0, 127.8, 127.7, 127.6, 127.3, 127.2, 121.6, 121.6, 121.4, 116.5, 113.9, 67.4, 67.2, 66.7, 66.5, 66.2, 55.1, 48.3, 47.4, 46.8, 45.4, 28.8, 28.2 ppm.

TLC: R_f = 0.85 (100% EtOAc)

HRMS (ESI-TOF) calc'd for $C_{29}H_{28}N_3O_4$ $[M+H]^+$ 482.2080; Found 482.2077.

$[\alpha]_D^{20}$ = 10.3° (c 2.0, $CHCl_3$);

Compound A13

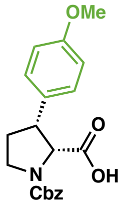

(2*R*,3*R*)-1-((benzyloxy)carbonyl)-3-(4-methoxyphenyl)pyrrolidine-2-carboxylic acid (A13)

A flame-dried reaction tube was charged with benzyl (2*R*,3*R*)-3-(4-methoxyphenyl)-2-[(quinolin-8-yl)carbamoyl]pyrrolidine-1-carboxylate (964 mg, 2.00 mmol, 1 equiv.), followed by di-*tert*-butyl dicarbonate (2.18 g, 10.0 mmol, 5 equiv.) and 4-(dimethylamino)pyridine (DMAP, 48.7 mg, 0.4 mmol, 0.2 equiv.). The reaction vessel was sealed and purged with argon, then MeCN (3.3 mL, 0.6 M) was added by syringe. The reaction tube was then placed in an oil bath and gradually warm up to 70 ºC. The reaction mixture was then stirred at 70 °C for 24 h. The reaction mixture was then allowed to cool to room temperature and sat. aq. NH_4Cl (6 mL) and CH_2Cl_2 (6 mL) were added. The phases were separated, and the aqueous layer was extracted with CH_2Cl_2 (3× 6 mL). The combined organic extracts were dried over Na_2SO_4 and filtered. The solvent was removed *in vacuo* to afford the crude N-Boc protected pyrrolidine derivative. A solution of H_2O_2 (0.4 mL, 4.0 mmol, 30 wt.% in H_2O, 4 equiv.) in THF (1 mL) was added to a solution of $LiOH{\cdot}H_2O$ (135 mg, 3.2 mmol, 1.6 equiv.) in H_2O (3 mL) at 0 °C under argon. The resulting mixture was added dropwise to a solution of crude *N*-Boc protected piperidine in THF (4 mL) at 0 °C under argon. The reaction was then stirred at 45 °C for 24 h. The reaction mixture was then allowed to cool down to room temperature and sat. aq. $Na_2S_2O_3$ (3 mL), EtOAc (3 mL) and 1 M NaOH (3 mL) were added. The aqueous layer was collected, and the organic layer extracted with 1 M NaOH (3 ×7 mL). The aqueous layers were combined, washed with EtOAc (20 mL), and acidified to pH 2–3 with conc. HCl. The acidic layer was extracted with EtOAc (5 × 25 mL). The combined organic extracts were washed with brine (35 mL), dried over Na_2SO_4 and filtered. Solvent was removed under reduced pressure. Et_2O (35 mL) and pentane (35 mL) were added and the solvent was removed under reduced pressure to afford the pure **A13** (548 mg, 77% over 2 steps, single diastereoisomer).

Physical state: White solid

m.p.: 185-187 °C

^{1}H NMR (600 MHz, $CDCl_3$, both rotamers): δ 7.44 – 7.36 (m, 2H), 7.34 – 7.26 (m, 3H), 7.16 (t, *J* = 8.4 Hz, 2H), 6.85 (dd, *J* = 8.8, 2.4 Hz, 2H), 5.26 – 4.99 (m, 2H), 4.57 (dd, *J* = 30.3, 8.7 Hz, 1H), 3.91 – 3.87 (m, 1H), 3.77 (s, 3H), 3.70 – 3.63 (m, 1H), 3.57 – 3.50 (m, 1H), 2.60 – 2.48 (m, 1H), 2.11 – 2.18 (m, 1H).

^{13}C NMR (151 MHz, $CDCl_3$, both rotamers): δ 175.8, 175.5, 159.1, 159.0, 155.4, 155.1, 136.6, 136.4, 129.0, 129.0, 128.6, 128.5, 128.4, 128.2, 128.0, 127.7, 114.0, 67.4, 67.3, 64.1, 63.8, 55.3, 47.5, 46.6, 46.4, 46.1, 28.7, 27.8

HRMS (ESI$^+$) calc'd for $C_{20}H_{22}NO_5$ $[M+H]^+$ 356.1492; Found 356.1490.

TLC: R_f = 0.21 (4% MeOH/DCM)

$[\alpha]_D^{20}$ = 24.3° (c 2.0, $CHCl_3$);

Compound 5

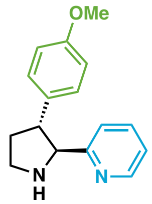

2-((2*S*,3*R*)-3-(4-methoxyphenyl)pyrrolidin-2-yl)pyridine (5).

A culture tube was charged with (2*R*,3*R*)-1-((benzyloxy)carbonyl)-3-(4-methoxyphenyl)pyrrolidine-2-carboxylic acid (89 mg, 0.25 mmol, 1.0 equiv.) and HATU (148 mg , 0.25 mmol, 1.0 equiv.). The tube was then evacuated and backfilled with argon from a balloon. DMF (2.5 mL) and Et_3N (35 µL, 0.25 mmol, 1.0 equiv.) were added. The mixture was stirred for 30 minutes. A solution of $NiCl_2$•glyme (26 mg, 0.13 mmol, 0.5 equiv.), and di^{t}BuBipy (35 mg, 0.25 mmol, 1 equiv.) in DMF (2.5 mL) was added, and the mixture was stirred for 5 minutes. A 0.42 M solution of 2-pyridine zinc reagent (1.8 mL, 0.75 mmol, 3.0 equiv.) in THF was then added. The argon balloon was removed, and the culture tube was sealed with Teflon™ tape and electrical tape. The resulting mixture was allowed to stir overnight at room temperature. The reaction mixture was quenched with 0.1 M HCl and extracted with EtOAc (2x 10 mL). The organic layer was washed with water (2x 10 mL), LiCl (1x 10 mL) and dried over $MgSO_4$. The organic layer was concentrated *in vacuo*. The crude product was then diluted in 1 mL of EtOAc and Pd/C (10 wt%, 2.50 mg, 0.005 mmol) was added. The reaction vessel was purged three times with hydrogen, charged to 50 psi. The reaction was heated under microwave irradiation to 80 °C and held for 5 minutes. Upon cooling to room temperature, the reaction mixture was filtered through Celite®, washed with EtOAc and condensed. The crude mixture was then purified by silica gel flash column chromatography (4:96 MeOH:DCM) to yield the pure compound **5** as a colorless oil (36.8 mg, 58% over 3 steps).

Physical state: colorless oil

^{1}H NMR (600 MHz, $CDCl_3$): δ 8.56 (d, J = 4.5 Hz, 1H), 7.57 (t, J = 7.7 Hz, 1H), 7.28 (dd, J = 7,7, 4.8 Hz, 1H), 7.20 (t, J = 7.7 Hz, 2H), 6.89 – 6 .88 (m, 2H), 6.80 (d, 7.1 Hz, 1H), 3.90 (t, J = 7.7 Hz, 2H), 3.80 (s, 3H), 3.57 – 3.53 (m, 1H), 3.30 – 3.26 (m, 1H), 3.54 – 3.42 (m, 2H).

HRMS (ESI$^+$) calc'd for $C_{14}H_{19}N_2O$ $[M+H]^+$ 356.1492; Found 356.1490.

TLC: R_f = 0.21 (4% MeOH/DCM)

$[\alpha]_D^{20}$ = 87° (c 1.0, $CHCl_3$);

Chiral SFC: IG column (3 μm, 4.6x250 mm) under isocratic conditions [3% MeOH / CO_2 (4 mL/min), 1600 psi backpressure] at 30 °C. The enantiomers were detected by UV light (260 nm). t_R (major) = 5.735 min, t_R (minor) = 6.149 min, >99 % *ee*.

Data are in accordance with those published in the literature.[17]

Racemic:

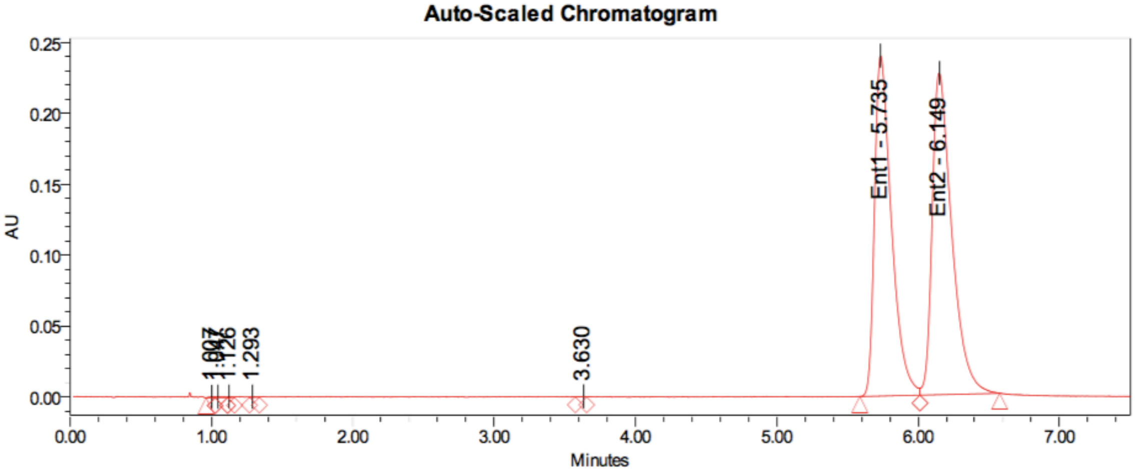

Enantioenriched **5**:

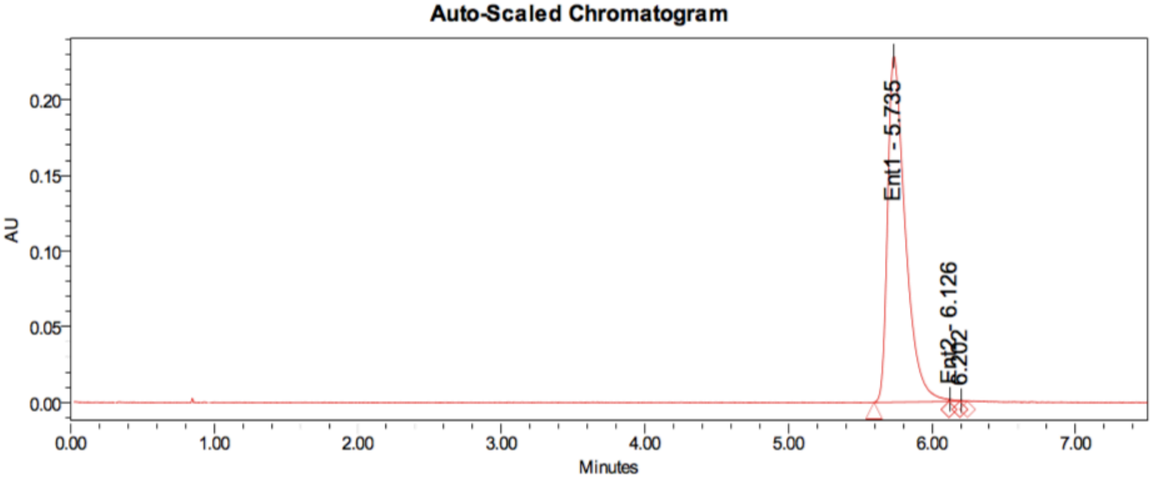

KSF-JV-1	47.74	52.26	-4.52	1999311	2188759
KSF-JV-2	99.77	0.23	99.54	1953645	4485

Unsuccessful or Challenging Substrates

Morpholine Scaffold: C-H sp^3 Arylation

Table S2: 8-*Aminoquinoline as directing group*: Screening of Bases and solvents using $Pd(OAc)_2$ as catalyst.

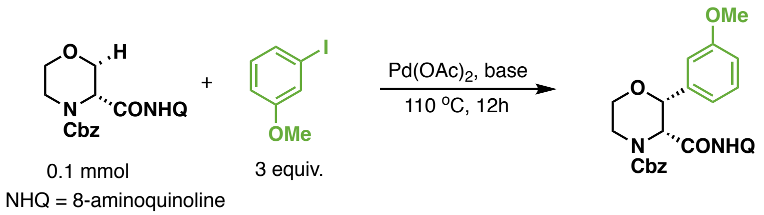

entry	base	solvent	yield[a]
1	K_2CO_3	t-Am-OH	n.d.
2	K_3PO_4	t-Am-OH	n.d.
3	KOAc	t-Am-OH	n.d.
4	Cs_2CO_3	t-Am-OH	n.d.
5	K_3PO_4	t-Am-OH:H_2O = 4:1	n.d.
6	KH_2PO_4	t-Am-OH	n.d.
7	AgTFA	toluene	n.d.
8	AgOTf	toluene	n.d.
9	K_2CO_3	HFIP	n.d.
10	Cs_2CO_3	HFIP	n.d.
11[b]	AgOAc	toluene[c]	15%
12[b]	AgOAc	toluene	12%
13[b]	AgOAc	toluene[d]	10%
14[e]	AgOAc	neat	12%
15[b]	Ag_2CO_3	toluene	9%
16[b]	Ag_2O	toluene	6%

Conditons: $Pd(OAc)_2$ (10 mol%), base (2 equiv), solvent (1 mL).[a] yield determined by NMR
[b] reaction carried out with 4-Iodoanisole [c] 90 °C [d] 130 °C [e] 4-Iodoanisole (10 equiv).

Table S3: *Boc as protecting group*: Screening of Bases and solvents using $Pd(OAc)_2$ as catalyst.

Morpholine 3-amide:

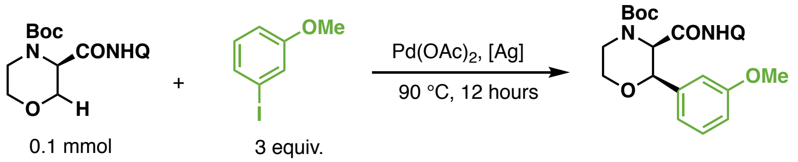

entry	[Ag]	additive	solvent	yield
1	AgOAc	-	toluene	n.d.
2[b]	AgOAc	-	toluene	n.d.
3[c]	AgOAc	-	toluene	n.d.
4[b]	AgOAc	-	t-BuOH	n.d.
5[b]	Ag_2CO_3	$(BnO)_2PO_2H$ (0.2 eq)	t-AmOH	n.d.
6[b]	Ag_2CO_3	$(BnO)_2PO_2H$ (0.2 eq)	DCE	n.d.

Conditons: $Pd(OAc)_2$ (10 mol%), [Ag] (2 equiv), toluene (0.5 mL).[a] yield determined by NMR [b] 110 °C. [c] $Pd(OAc)_2$ (50 mol%), $(BnO)PO_2H$ (20 mol%) as additive, Ar.

Morpholine 2-amide:

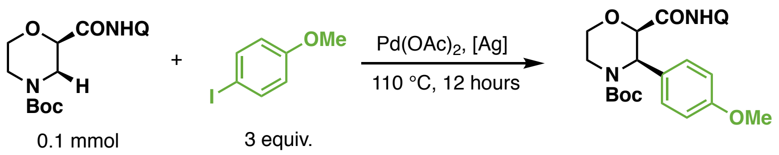

entry	[Ag]	solvent	yield[a]
1	AgOAc	toluene	10%
2	AgOAc	dioxane	8%
3	AgOAc	DCE	6%
4	AgOAc	t-AmOH	traces
5	Ag_2CO_3	MeCN	traces
6	Ag_2CO_3	toluene	n.r.
7	Ag_2O	toluene	n.r
8[b]	Ag_2CO_3	t-AmOH	n.r

Conditons: $Pd(OAc)_2$ (10 mol%), [Ag] (2 equiv), solvent (1 mL).[a] yield determined by NMR [b] $(BnO)PO_2H$ (20 mol%) as additive, Ar.

Table S4*: PIP as directing group*: Screening of Bases and solvents using $Pd(OAc)_2$ as catalyst.

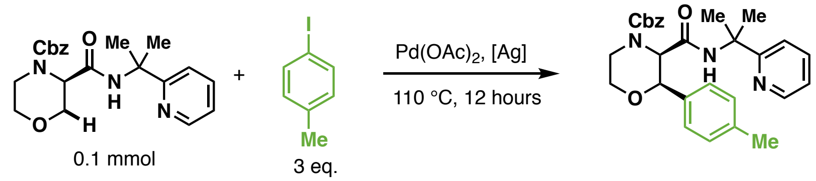

entry	[Ag]	additive	solvent	yield[a]
1	AgOAc	—	toluene	n.d.
2	AgOAc	—	t-BuOH	n.d.
3	AgOAc	PivOH (0.5 eq)	toluene	n.d.
4	Ag_2CO_3	$(BnO)_2PO_2H$ (0.2 eq)	t-AmOH	n.d.
5[b]	AgOAc	—	toluene	n.d.
6	Ag_2CO_3	$(BnO)_2PO_2H$ (0.2 eq)	DCE:^{t}BuOH	n.d.

Conditons: $Pd(OAc)_2$ (10 mol%), [Ag] (1.8 equiv), solvent (1 mL).[a] yield determined by NMR [b] 130 °C

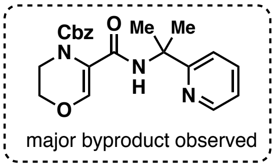

Morpholine Scaffold: C-H sp^3 Methylation and Alkynylation

Table S5*:* Screening of conditions for C-H sp^3 Methylation

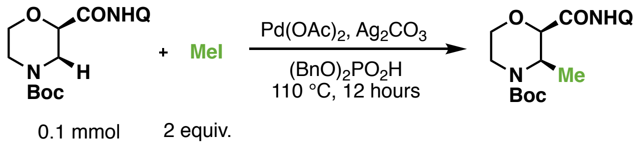

entry	solvent	yield[a]
1	t-amy-OH	traces
2[b]	t-amy-OH	traces
3	toluene	nd

Conditons: $Pd(OAc)_2$ (10 mol%), $(BnO)_2PO_2H$ (20 mol%), Ag_2CO_3 (2 equiv.), solvent (0.5 mL).[a] yield determined by LC-MS. [b] without $(BnO)_2PO_2H$.

Table S6*:* Screening of conditions for C-H sp^3 Alkynylation

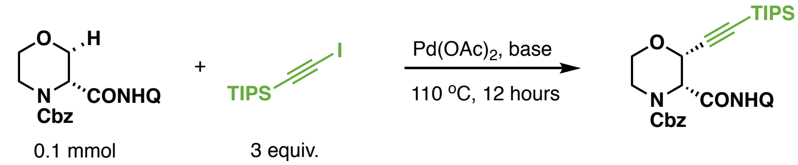

entry	base	solvent	yield[a]
1	AgOAc	toluene	n.d.
2	Ag_2CO_3	toluene	n.d.
3	AgTFA	toluene	n.d.
4[b]	AgOAc	HFIP	n.d.
5	AgOAc	DCE	n.d.
6	AgOAc	dioxane	n.d.
7	AgOAc	MeCN	n.d.
8	AgOAc + K_2CO_3	toluene	n.d.
9	AgOAc + CsF	toluene	n.d.

Conditons: $Pd(OAc)_2$ (10 mol%), base (2 equiv.), solvent (1 mL). [a] yield determined by NMR [b] byproduct observed.

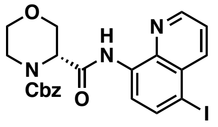

Piperazine Scaffold: C-H sp^{3}alkoxylation and arylation

Table S7: Screening of conditions for C-H sp^3 Alkoxylation

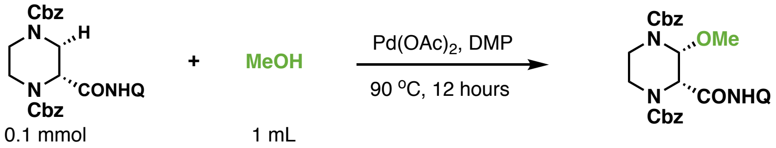

entry	$Pd(OAc)_2$ (mol%)	DMP (eq)	additive	yield[a]
1	30	4	–	15%
2	50	4	–	23%
3[b]	20	4	–	<5%
4	20	4	–	<5%
5	20	4	$PhI(OAc)_2$ (1 eq)	<5%
6	20	4	HOAc (1 eq)	10%
**7**	20	–	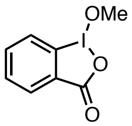	11%

Conditons: [a] yield determined by NMR. [b] 70 °C.

Table S8: ***8-Aminoquinoline as directing group*****: Screening of conditions for arylation.**

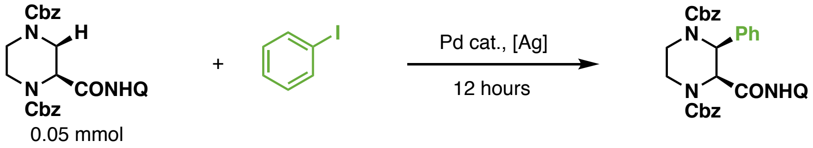

entry	PhI (eq)	[Pd]	[Ag]	solvent	temperature	additive	yield[a]
1	3	$Pd(OAc)_2$	AgOAc	Toluene	110 °C	–	nd
2	3	$Pd(TFA)_2$	AgOAc	Toluene	110 °C	–	nd
3	10	$Pd(OAc)_2$	Ag_2CO_3	neat	110 °C	PivOH	nd
4	3	$Pd(OAc)_2$	Ag_2CO_3	Toluene	110 °C	–	nd
5	3	$Pd(OAc)_2$	AgOAc	Toluene	110 °C	PivOH	nd
6	10	$Pd(OAc)_2$	AgOAc	neat	110 °C	–	nd
7	3	$Pd(OAc)_2$	$AgBF_4$	t-BuOH	75 °C	–	traces
8	20	$Pd(OAc)_2$	AgOAc	Toluene	110 °C	–	nd
9[b]	3	$Pd(OAc)_2$	AgOAc	Toluene	90 °C	–	nd
10[b]	3	$Pd(OAc)_2$	AgOAc	neat	110 °C	–	nd
11[b,c]	3	$Pd(OAc)_2$	AgOAc	Toluene	125 °C	–	nd

Conditons: $Pd(OAc)_2$ (10 mol%), [Ag] (2 equiv.), additive (0.2 equiv.), solvent (0.5 mL).[a] yield determined by LC-MS. [b] reaction carried out with 4-Iodoanisole. [c] reaction carried out using a microwave reactor for 20 minutes.

Table S9: ***2-thiomethylaniline as directing group:*** **Screening of conditions for arylation.**

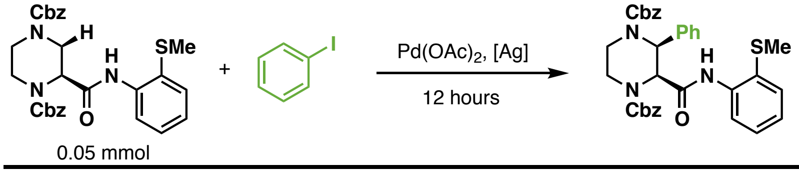

entry	PhI (eq)	[Ag]	solvent	temperature	additive	yield
1	3	AgOAc	toluene	110 °C	–	traces
2	10	Ag_2CO_3	–	110 °C	PivOH	nd
3	3	Ag_2CO_3	t-BuOH	75 °C	–	nd
4	3	$AgBF_4$	t-BuOH	75 °C	–	nd

Conditons: $Pd(OAc)_2$ (10 mol%), [Ag] (2 equiv.), additive (0.2 equiv.), solvent (0.5 mL).
[a] yield determined by LC-MS

Experimental Procedures and Characterization Data for BRD8468 (74) (Figure 5-a)

Synthetic route to compound BRD8468 (74)

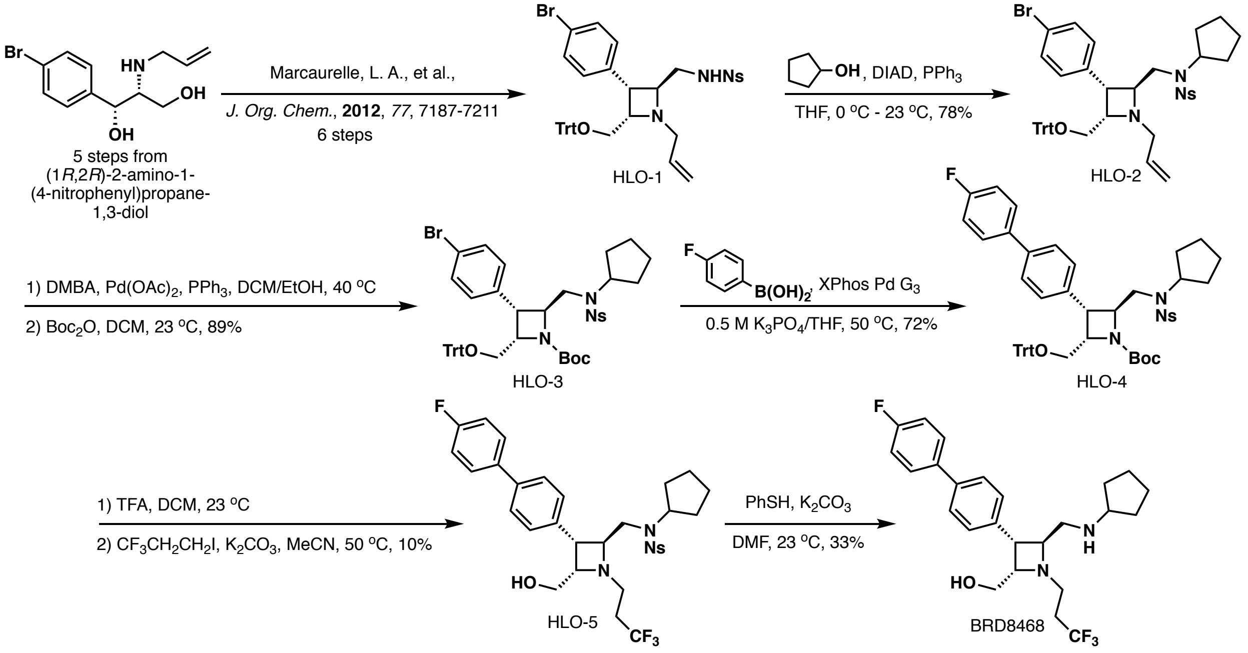

Compound BRD8468

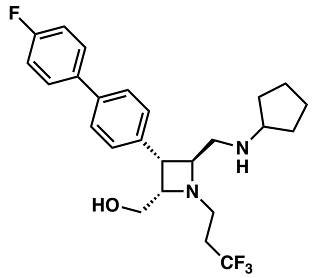

((2*S*,3*R*,4*S*)-4-((cyclopentylamino)methyl)-3-(4'-fluoro-[1,1'-biphenyl]-4-yl)-1-(3,3,3-trifluoropropyl)azetidin-2-yl)methanol (72)

Physical State: colorless oil

^{1}H NMR (600 MHz, CDCl$_3$): δ 7.54 – 7.49 (m, 4H), 7.32 (d, *J* = 7.9 Hz, 2H), 7.12 (t, *J* = 8.6 Hz, 2H), 4.10 (s, 1H), 3.99 (s, 1H), 3.78 (s, 1H), 3.65 (d, *J* = 3.6 Hz, 1H), 3.54 (dd, *J* = 12.1, 3.9 Hz, 1H), 3.21 (s, 1H), 2.88 (dd, *J* = 12.5, 4.9 Hz, 1H), 2.82 – 2.76 (m, 1H), 2.40 – 2.36 (m, 1H), 2.34 (t, *J* = 7.5 Hz, 3H), 1.92 (d, *J* = 23.4 Hz, 1H) ppm.

^{13}C NMR (151 MHz, CDCl$_3$): δ 128.1, 128.1, 127.6, 127.5, 127.1, 126.8, 126.3, 115.3, 115.1, 70.1, 65.2, 37.0, 36.7, 35.5, 34.0, 33.3, 31.5, 31.4, 27.0, 26.3, 25.1, 22.7, 22.2, 13.7 ppm.

^{19}F NMR (376 MHz, CDCl$_3$): δ -65.10, -115.86 ppm.

LRMS-ESI: (*m/z*) 451.6 [M+H]$^+$

HRMS (ESI-TOF): calc'd for $C_{25}H_{31}F_4N_2O$ [M+H]$^+$: 451.2373, found: 451.2373.

TLC: R$_f$ = 0.47 (9:1 CH_2Cl_2:MeOH).

Note: *An LCMS of BRD8468 provided by the Broad Institute was run alongside a LCMS of the synthesized BRD8468 (**74**) as well as a mixture of both provided/synthesized BRD8468*

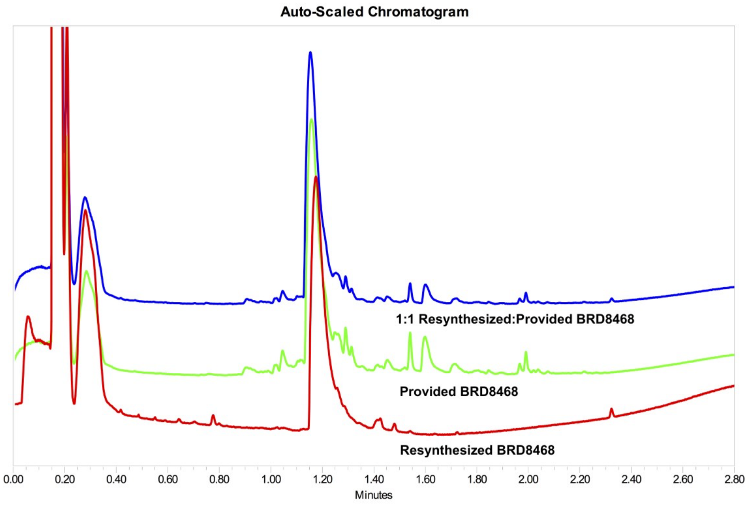

Experimental Procedures and Characterization Data for the synthesis of BRD8468 analogs (Figure 5-b)

Compound SI- 44[16]

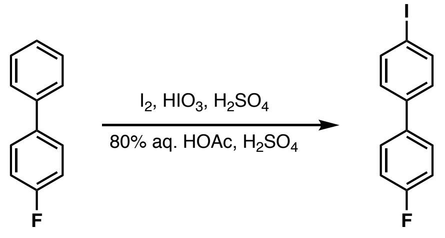

SI-44

4-fluoro-4'-iodo-1,1'-biphenyl (SI-44)

A round-bottom flask equipped with a condenser and a stir bar was charged with 4-fluoro-1,1'-biphenyl (17.5 g, 172 mmol, 1 equiv.) and 80% aqueous HOAc (246 mL), concentrated H_2SO_4 (2.19 mL), I_2 (17.1 g, 20.3 mmol, 0.7 equiv.), HIO_3 (4.66 g, 26.4 mmol, 0.26 equiv.) were added to the flask. The suspension was heated at 80 °C until full conversion of starting material, followed by GC-MS (typical time was 8 hours). Then, the reaction mixture was cooled to room temperature and diluted with a sat. aq. $NaHSO_3$ solution. Additional solid $NaHSO_3$ was added, and the suspension was stirred for 3 hours to remove solid I_2. The solid was filtered, washed with water, and dried in vacuo to afford the desired product as a white solid. The solid was then recrystallized in EtOH affording 29.2 g (97%) of the title compound **SI-44.**

Physical State: white crystalline solid.
m.p.: 88-89 °C.
^{1}H NMR (600 MHz, $CDCl_3$): δ 7.76 (d, J = 8.5 Hz, 2H), 7.53 – 7.48 (m, 2H), 7.29 – 7.26 (m, 2H), 7.13 (t, J = 8.7 Hz, 2H) ppm.
^{19}F NMR (376 MHz, $CDCl_3$): δ -115.17 ppm.
^{13}C NMR (151 MHz, $CDCl_3$): δ 162.8 (d, J_{C-F} = 247.1 Hz), 139.9, 138.0, 136.3 (d, J_{C-F} = 3.3 Hz), 129.0, 128.6 (d, J_{C-F} = 8.0 Hz), 115.9 (d, J_{C-F} = 21.5 Hz), 93.1 ppm.
TLC: R_f = 0.5 (9: 1 Hexanes:Ether).
*Note: no HRMS could be recorded for **SI-44***

Compound 74

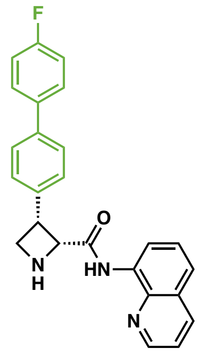

(2*R*,3*S*)-3-(4'-fluoro-[1,1'-biphenyl]-4-yl)-N-(quinolin-8-yl)azetidine-2-carboxamide (74)

Following General Procedure **A2.2** on 10.5 mmol scale. Purification by flash column chromatography (silica, 6:4 Hexanes:EtOAc) afforded 3.28 g (79 %) of the title compound **74**.

Physical State: white solid.

m.p.: 185-187 °C.

^{1}H NMR (600 MHz, $CDCl_3$): δ 11.33 (s, 1H), 8.93 (d, J = 4.9 Hz, 1H), 8.48 (d, J = 7.6 Hz, 1H), 8.12 (d, J = 8.2 Hz, 1H), 7.55 (d, J = 8.1 Hz, 2H), 7.47 – 7.36 (m, 3H), 7.32 – 7.23 (m, 4H), 6.99 (t, J = 8.7 Hz, 2H), 4.99 (d, J = 9.4 Hz, 1H), 4.42 – 4.20 (m, 2H), 3.79 (dd, J = 7.9, 4.2 Hz, 1H) ppm.

^{19}F NMR (376 MHz, $CDCl_3$): δ -116.45 ppm.

^{13}C NMR (151 MHz, $CDCl_3$): δ 169.9, 162.3 (d, $J_{C\text{-}F}$ = 246.0 Hz), 148.7, 139.1, 138.8, 138.2, 137.0 (d, $J_{C\text{-}F}$ = 3.2 Hz), 136.2, 133.8, 128.6 - 128.5 (m), 128.1, 127.3, 126.9, 121.7 (d, $J_{C\text{-}F}$ = 43.1 Hz), 116.6, 115.4 (d, $J_{C\text{-}F}$ = 21.4 Hz), 65.0, 50.1, 43.1 ppm.

HRMS (ESI-TOF): calc'd for $C_{25}H_{21}FN_3O$ $[M+H]^+$: 398.1669, found: 398.1668.

TLC: R_f = 0.16 (16:1 Ether:MeOH).

Compound SI-45

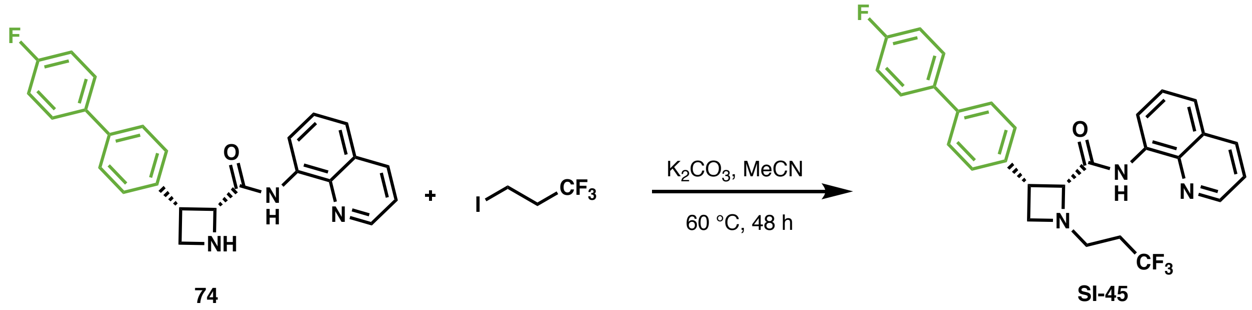

(2*R*,3*S*)-3-(4'-fluoro-[1,1'-biphenyl]-4-yl)-N-(quinolin-8-yl)-1-(3,3,3 trifluoropropyl)azetidine-2-carboxamide (SI-45)

A reaction vessel was charged with (2*R*,3*S*)-3-(4'-fluoro-[1,1'-biphenyl]-4-yl)-N-(quinolin-8-yl)azetidine-2-carboxamide (**74**), (4.29 g, 10.8 mmol, 1 equiv.) and K_2CO_3 (5.96 g, 43.2 mmol, 4 equiv.). The reaction vessel was evacuated and backfilled with argon from a balloon (3x). Acetonitrile was added (21.6 mL, 0.5 M), followed by addition of 1,1,1-Trifluoro-3-iodopropane (5.10 mL, 43.2 mmol, 4 equiv.) and the vessel was placed in an oil bath (preheated to 60 °C) for 12 hours. The reaction mixture was then allowed to cool to room temperature and more 1,1,1-Trifluoro-3-iodopropane (1 mL, 8.56 mmol, 1 equiv.) and acetonitrile (6 mL) were added to the mixture. The reaction vessel was replaced in oil bath for further 12 hours. Then, after 48 hours the reaction vessel was allowed to cool to room temperature and the reaction mixture was quenched with water and extracted with EtOAc (3x). The combined organic layers were dried over Na_2SO_4, filtered and concentrated *in vacuo*. The crude mixture was purified by flash column chromatographic (silica, 3:7 Hexanes:Ether) to afford 2.39 g (48 %) of the title compound **SI-45**.

Physical State: yellow oil.

^{1}H NMR (600 MHz, CDCl$_3$): δ 11.38 (bs, 1H), 9.00 (bs, 1H), 8.57 (bs, 1H), 8.12 (bs, 1H), 7.68 (bs, 2H), 7.55 – 7.22 (m, 7H), 7.03 (bs, 2H), 4.37 (bs, 1H), 4.16 (bs, 1H), 3.98 (bs, 1H), 3.67 (bs, 1H), 3.19 (bs, 1H), 3.04 (bs, 1H), 2.65 (bs, 2H) ppm.

^{19}F NMR (376 MHz, CDCl$_3$): δ -65.28, -116.29 ppm.

^{13}C NMR (151 MHz, CDCl$_3$): δ 168.2, 162.2 (d, J_{C-F} = 246.0 Hz), 148.5, 138.8 (d, J_{C-F} = 33.2 Hz), 137.4, 136.7 (d, J_{C-F} = 2.5 Hz), 136.0, 133.5, 128.6, 128.3 (d, J_{C-F} = 8.0 Hz), 127.9, 127.0, 126.7, 121.7 (d, J_{C-F} = 43.2 Hz), 116.3, 115.3 (d, J_{C-F} = 246.0 Hz), 71.9, 56.5, 50.7, 38.8, 33.0 (q, J_{C-F} = 28.3 Hz) ppm.

HRMS (ESI-TOF): calc'd for $C_{28}H_{24}F_4N_3O$ $[M+H]^+$: 494.1856, found: 494.1859.

TLC: R_f = 0.43 (3:7 Hexanes:Ether).

$[\alpha]_D^{20}$ = -141.30 (c = 1.0, $CHCl_3$).

Compound A15

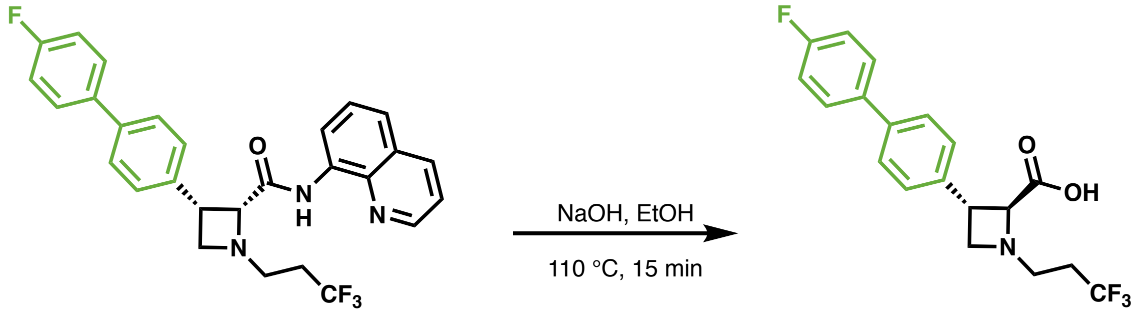

SI-45 A15

(2*S*,3*S*)-3-(4'-fluoro-[1,1'-biphenyl]-4-yl)-1-(3,3,3-trifluoropropyl)azetidine-2-carboxylic acid (A15)

A sealed tube equipped with a stir bar was charged with (2*R*,3*S*)-3-(4'-fluoro-[1,1'-biphenyl]-4-yl)-N-(quinolin-8-yl)-1-(3,3,3 trifluoropropyl) azetidine-2-carboxamide **SI-45** (1 g, 2.03 mmol, 1 equiv.), followed by the addition of EtOH (10.1 mL, 0.2 M). To the mixture NaOH pellets (0.81 g, 20.3 mmol, 10 equiv.) were added. The tube was sealed and placed in an oil bath (preheated to 110°C) for 15 min (*note: reaction time is critical*). The reaction mixture was then allowed to cool to room temperature and water was added and the crude mixture was washed with DCM (2x). The resulting aqueous solution was acidified with 1 M HCl, exhaustively extracted with 3:7 *i*-PrOH:$CHCl_3$ (5x) and dried over Na_2SO_4. Concentration *in vacuo* afforded 0.67 g (90 %) of the title compound **A15**.

Physical State: brown solid.

m.p.:185-186 °C.

^{1}H NMR (400 MHz, MeOD): δ 7.68 – 7.62 (m, 4H), 7.56 (d, *J* = 8.3 Hz, 2H), 7.19 (t, *J* = 8.8 Hz, 2H), 4.86 (d, *J* = 9.2 Hz, 1H), 4.37 (t, *J* = 8.6 Hz, 1H), 4.14 (q, *J* = 9.2 Hz, 1H), 4.04 (t, *J* = 9.4 Hz, 1H), 3.66 – 3.44 (m, 2H), 2.83 – 2.63 (m, 2H) ppm.

^{19}F NMR (376 MHz, MeOD): δ -67.22, -117.94 ppm.

^{13}C NMR (151 MHz, MeOD): δ 172.1, 164.0 (d, $J_{C\text{-}F}$ = 245.0 Hz), 140.9, 138.3, 138.1(d, $J_{C\text{-}F}$ = 3.2 Hz), 129.7 (d, $J_{C\text{-}F}$ = 8.01 Hz), 128.9, 128.3, 116.6 (d, $J_{C\text{-}F}$ = 21.8 Hz), 74.9, 57.5, 40.9, 31.1 (q, $J_{C\text{-}F}$ = 30.3 Hz) ppm.

HRMS (ESI-TOF): calc'd for $C_{19}H_{18}F_4NO2$ $[M+H]^+$: 368.1274, found: 368.1274.

TLC: R_f = 0.17 (9:1 CH_2Cl_2:MeOH).

$[\alpha]_D^{20}$ = 71.4 (*c* = 0.5, MeOH).

Compound A16

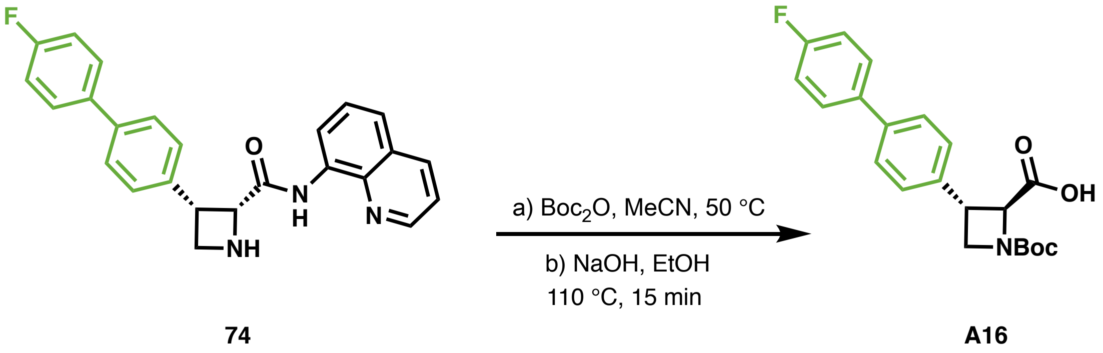

(2*S*,3*S*)-1-(*tert*-butoxycarbonyl)-3-(4'-fluoro-[1,1'-biphenyl]-4-yl)azetidine-2-carboxylic acid (A16)

A a round bottom flask equipped with a stir bar was charged with (2R,3S)-3-(4'-fluoro-[1,1'-biphenyl]-4-yl)-N-(quinolin-8-yl)azetidine-2-carboxamide **(74)** (1.25 g, 3.14 mmol, 1 equiv.) and dissolved in MeCN (7.4 mL, 0.5 M). Boc_2O (0.93 mL, 3.46 mmol, 1.1 equiv.) was added and the reaction mixture was heated to 50 ºC and stirred for 15 minutes. The solvent was removed *in vacuo* and the residue was dissolved in EtOH (15.7 mL, 0.2 M) and transferred to a sealed tube. To the mixture NaOH pellets (1.26 g, 31.4 mmol, 10 equiv.) were added. The tube was sealed and placed in an oil bath (preheated to 110°C) for 15 min (*note: reaction time is critical*). The reaction mixture was then allowed to cool to room temperature and water was added and the crude mixture was washed with DCM (2x). The resulting aqueous solution was acidified with 1 M HCl, exhaustively extracted with 3:7 *i*-PrOH:$CHCl_3$ (5x) and dried over Na_2SO_4. Concentration *in vacuo* afforded 716 mg (62 %) of the title compound **A16**.

Physical State: brown solid.

m.p.: 82-83 °C.

^{1}H NMR (600 MHz, $CDCl_3$): δ 7.53 – 7.45 (m, 4H), 7.36 (d, J = 8.0 Hz, 2H), 7.11 (t, J = 8.5 Hz, 2H), 5.14 – 4.98 (m, 1H), 4.35 – 3.97 (m, 3H), 1.47 (s, 9H) ppm.

^{19}F NMR (376 MHz, DMSO): δ -115.70 ppm.

^{13}C NMR (151 MHz, DMSO): δ 171.6, 162.7, 161.1, 138.2, 136.2 (d, J_{C-F} = 3.0 Hz), 128.6 (d, J_{C-F} = 8.2 Hz), 127.6, 127.0, 115.8 (d, J_{C-F} = 21.3 Hz), 79.3, 72.5, 37.5, 28.0 ppm.

TLC: R_f = 0.17 (CH_2Cl_2:MeOH).

$[\alpha]_D^{20}$ = 43.30 (c = 0.25, $CHCl_3$).

*Note: no HRMS could be recorded for **A17**.*

Compound B9

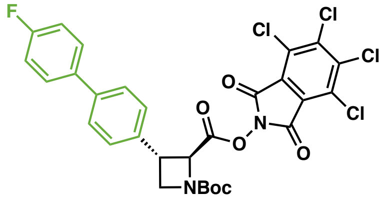

1-(*tert*-butyl) 2-(4,5,6,7-tetrachloro-1,3-dioxoisoindolin-2-yl) (2*S*,3*S*)-3-(4'-fluoro-[1,1'-biphenyl]-4-yl)azetidine-1,2-dicarboxylate (B9)

Following General Procedure **B** on 1 mmol scale with **A16**. Purification by flash column chromatography (silica, DCM), afforded 224 mg (34%) of the title compound **B9**.

Physical State: yellow solid.

m.p.: 98-100 °C.

^{1}H NMR (600 MHz, $CDCl_3$): δ 7.60 – 7.45 (m, 6H), 7.14 (t, J = 8.7 Hz, 2H), 4.94 (d, J = 5.5 Hz, 1H), 4.49 (t, J = 8.4 Hz, 1H), 4.18 (dd, J = 8.1, 5.9 Hz, 1H), 4.04 (dt, J = 8.6, 5.7 Hz, 1H), 1.50 (bs, 9H) ppm.

^{19}F NMR (376 MHz, DMSO): δ -115.61 ppm.

^{13}C NMR (151 MHz, $CDCl_3$): δ 166.8, 162.7 (dd, $J_{C\text{-}F}$ = 246.6, 7.6 Hz), 159.6, 141.4, 140.4, 140.2, 139.7, 137.8, 136.7 (dd, $J_{C\text{-}F}$ = 9.9, 3.2 Hz),130.7, 129.9, 128.8 – 128.7 (m), 127.7 (d, $J_{C\text{-}F}$ = 28.7 Hz), 127.5 (d, $J_{C\text{-}F}$ = 7.1 Hz), 125.1, 124.8, 115.9 (dd, $J_{C\text{-}F}$ = 21.4, 5.6 Hz), 81.9, 68.0, 53.4, 42.9, 39.2, 28.4, 28.3, 23.4 ppm.

HRMS (ESI-TOF): calc'd for $C_{24}H_{14}Cl_4FN_2O_4$[M-Boc+H]$^+$: 552.9692, found: 552.9698

TLC: R_f = 0.23 (CH_2Cl_2).

$[\alpha]_D^{20}$ = 113.0 (c = 0.1, $CHCl_3$).

X-Ray Crystallographic Data Compound B9

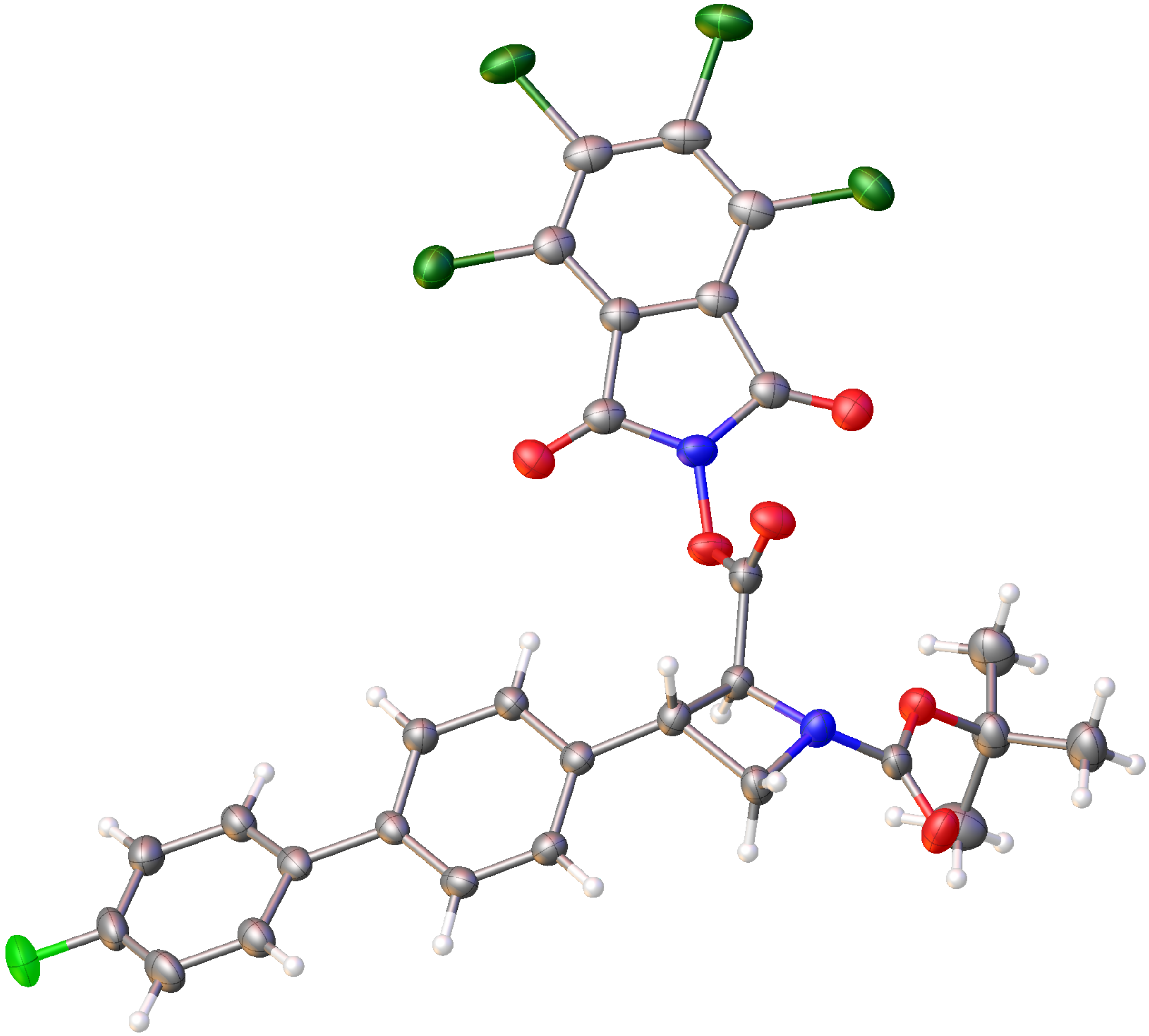

Table S10. Crystal data and structure refinement for Baran705.

Report date	2018-11-09	
Identification code	baran705	
Empirical formula	C29 H21 Cl4 F N2 O6	
Molecular formula	C29 H21 Cl4 F N2 O6	
Formula weight	654.28	
Temperature	100.0 K	
Wavelength	1.54178 Å	
Crystal system	Orthorhombic	
Space group	$P2_12_12_1$	
Unit cell dimensions	a = 7.0574(2) Å	α= 90°.
	b = 13.5533(3) Å	β= 90°.
	c = 30.2887(7) Å	γ = 90°.
Volume	2897.15(12) $Å^3$	
Z	4	
Density (calculated)	1.500 Mg/m^3	
Absorption coefficient	4.176 mm^{-1}	
F(000)	1336	
Crystal size	0.2 x 0.03 x 0.03 mm^3	
Crystal color, habit	colorless needle	
Theta range for data collection	2.918 to 68.236°.	
Index ranges	-8<=h<=8, -16<=k<=16, -33<=l<=36	
Reflections collected	32224	
Independent reflections	5325 [R(int) = 0.0753]	
Completeness to theta = 67.679°	100.0 %	
Absorption correction	Semi-empirical from equivalents	
Max. and min. transmission	0.7533 and 0.5927	
Refinement method	Full-matrix least-squares on F^2	
Data / restraints / parameters	5325 / 0 / 382	
Goodness-of-fit on F^2	1.055	
Final R indices [I>2sigma(I)]	R1 = 0.0423, wR2 = 0.1033	
R indices (all data)	R1 = 0.0507, wR2 = 0.1076	
Absolute structure parameter	0.005(10)	
Largest diff. peak and hole	0.297 and -0.346 e.$Å^{-3}$	

Table S11. Atomic coordinates (x 10^4) and equivalent isotropic displacement parameters ($Å^2$x 10^3)

for Baran705. U(eq) is defined as one third of the trace of the orthogonalized U^{ij} tensor.

	x	y	z	U(eq)
Cl(1)	3981(2)	10745(1)	5693(1)	41(1)
Cl(2)	3472(2)	11665(1)	6626(1)	50(1)
Cl(3)	3837(2)	10368(1)	7464(1)	49(1)
Cl(4)	4991(2)	8138(1)	7382(1)	40(1)
F(1)	6355(6)	8988(2)	2046(1)	56(1)
O(1)	6247(6)	2784(2)	5350(1)	40(1)
O(2)	7090(5)	4118(2)	5770(1)	33(1)
O(3)	3053(5)	5894(2)	5783(1)	38(1)
O(4)	5775(5)	6721(2)	5627(1)	34(1)
O(5)	4578(4)	8567(2)	5341(1)	34(1)
O(6)	5870(4)	6721(2)	6573(1)	36(1)
N(1)	4844(6)	4266(3)	5267(1)	31(1)
N(2)	5120(6)	7460(3)	5905(1)	33(1)
C(1)	4467(6)	5961(3)	5572(1)	30(1)
C(2)	5132(7)	5318(3)	5198(1)	28(1)
C(3)	3717(6)	5248(3)	4802(1)	27(1)
C(4)	3972(7)	4116(3)	4831(1)	30(1)
C(5)	6106(7)	3640(3)	5456(1)	30(1)
C(6)	4202(6)	5787(3)	4382(1)	26(1)
C(7)	4507(6)	6799(3)	4391(1)	28(1)
C(8)	4855(7)	7327(3)	4009(1)	29(1)
C(9)	4958(6)	6847(3)	3599(1)	25(1)
C(10)	4677(6)	5830(3)	3594(1)	28(1)
C(11)	4294(6)	5312(3)	3976(1)	28(1)
C(12)	5338(6)	7412(3)	3187(1)	28(1)
C(13)	6183(7)	8341(3)	3202(1)	32(1)
C(14)	6519(7)	8882(4)	2818(2)	37(1)
C(15)	6026(7)	8464(4)	2423(1)	39(1)
C(16)	5190(8)	7550(4)	2390(2)	40(1)

C(17)	4857(7)	7026(4)	2776(1)	33(1)
C(18)	8752(8)	3654(3)	5982(2)	40(1)
C(19)	10278(8)	3503(5)	5630(2)	53(1)
C(20)	8200(9)	2696(4)	6205(2)	48(1)
C(21)	9366(8)	4435(4)	6314(2)	47(1)
C(22)	5374(6)	7423(3)	6363(2)	30(1)
C(23)	4852(6)	8450(3)	6500(1)	28(1)
C(24)	4676(6)	8846(4)	6918(2)	32(1)
C(25)	4191(6)	9849(4)	6951(2)	35(1)
C(26)	3994(6)	10429(3)	6578(2)	35(1)
C(27)	4170(6)	10014(3)	6155(2)	31(1)
C(28)	4573(6)	9025(3)	6125(1)	28(1)
C(29)	4741(6)	8384(3)	5725(2)	30(1)

Compound SI-46 and 73

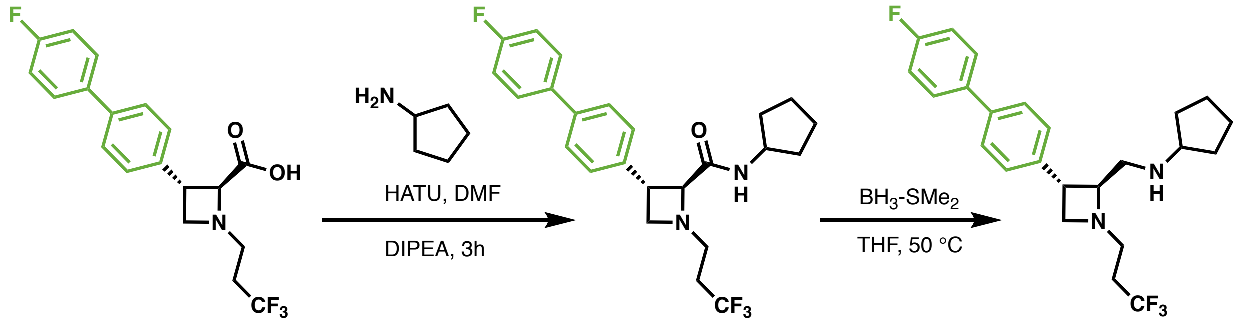

A15 SI-50 73

(2*S*,3*S*)-*N*-cyclopentyl-3-(4'-fluoro-[1,1'-biphenyl]-4-yl)-1-(3,3,3-trifluoropropyl)azetidine-2-carboxamide (SI-46)

To a reaction vessel containing (2*S*,3*S*)-3-(4'-fluoro-[1,1'-biphenyl]-4-yl)-1-(3,3,3-trifluoropropyl)azetidine-2-carboxylic acid **A15** (147 mg, 0.4 mmol, 1.0 equiv.) was added DMF (1.6 mL, 0.25 M) and cooled to 0 °C before the addition of *N,N*-diisopropylethylamine (139 µL, 0.8 mmol, 2.0 equiv.), HATU (152 mg, 0.4 mmol, 1.0 equiv.), and cyclopentylamine (37.5 mg, 0.4 mmol, 1.1 equiv.) in sequence. The ice bath was removed, and the reaction was allowed to warm to room temperature and stirred for 30 minutes. The reaction mixture was then diluted with EtOAc and washed with water (2x) and brine (1x). The resulting organic layer was dried over Na_2SO_4, filtered, and concentrated *in vacuo*. Purification by flash column chromatography (silica, 4:1 Hexanes:EtOAc) afforded 116 mg (67%) of the title compound **SI-46**.

Physical State: colorless sticky oil.

^{1}H NMR (600 MHz, $CDCl_3$): δ 7.54 – 7.47 (m, 4H), 7.38 (d, J = 8.2 Hz, 1H), 7.22 (d, J = 8.2 Hz, 1H), 7.11 (t, J = 8.7 Hz, 2H), 4.26 (q, J = 6.9 Hz, 1H), 3.94 (t, J = 6.7 Hz, 1H), 3.69 – 3.60 (m, 2H), 3.11 (t, J = 7.4 Hz, 1H), 2.86 (dt, J = 12.5, 7.6 Hz, 1H), 2.71 (dt, J = 12.5, 6.2 Hz, 1H), 2.25 – 2.17 (m, 2H), 2.03 (tt, J = 13.5, 6.0 Hz, 2H), 1.77 – 1.60 (m, 5H), 1.45 (dq, J = 27.7, 6.9 Hz, 2H) ppm.

^{19}F NMR (376 MHz, $CDCl_3$): δ -64.85, -116.04 ppm.

^{13}C NMR (151 MHz, $CDCl_3$): δ 171.2, 162.6 (d, J_{C-F} = 246.5 Hz), 139.2 (d, J_{C-F} = 66.6 Hz), 137.1 (d, J_{C-F} = 3.2 Hz), 128.7 (d, J_{C-F} = 8.0 Hz), 127.5, 127.3, 115.7 (d, J_{C-F} = 19.3 Hz), 73.2, 57.5, 51.0 - 50.9 (m), 50.5, 40.9, 32.2 (q, J_{C-F} = 28.1 Hz), 23.9 (d, J_{C-F} = 3.3 Hz) ppm.

HRMS (ESI-TOF): calc'd for $C_{24}H_{27}F_4N_2O$ $[M+H]^+$: 435.2060, found: 435.2062.

TLC: R_f = 0.26 (4:1 Hexanes:EtOAc).

$[\alpha]_D^{20}$ = 53.42 (*c* = 1.12, MeOH).

N-(((2*S*,3*S*)-3-(4'-fluoro-[1,1'-biphenyl]-4-yl)-1-(3,3,3-trifluoropropyl)azetidin-2-yl)methyl)cyclopentanamine (73)

To a reaction vessel containing (2*S*,3*S*)-*N*-cyclopentyl-3-(4'-fluoro-[1,1'-biphenyl]-4-yl)-1-(3,3,3-trifluoropropyl)azetidine-2-carboxamide **SI-46** (96 mg, 0.22 mmol, 1.0 equiv.) was added THF (1.1 mL, 0.2 M). The reaction mixture was cooled to 0 °C before the slow addition of Borane-dimethyl sulfide complex (BH_3-SMe_2, 0.55 mL (2 M), 1.1 mmol, 5 equiv.). The reaction mixture was allowed to warm to room temperature, then was heated to 50 °C, followed by TLC, upon full conversion of starting material. The mixture was then cooled in an ice bath, and MeOH (1 mL) was added dropwise. The mixture was stirred at room temperature for 20 minutes, then Rochelle salt's solution was added and the reaction mixture was stirred overnight. The clear solution was extracted with EtOAc (3x) and the combined organics were washed with brine, dried over Na2SO4 and concentrated *in vacuo*. Purification by flash column chromatography (silica, 1:1 Hexanes:EtOAc) afforded 45 mg (49%) of the title compound **73**.

Physical State: colorless oil

^{1}H NMR (600 MHz, $CDCl_3$): δ 7.52 – 7.45 (m, 4H), 7.36 (d, *J* = 7.5 Hz, 2H), 7.12 (t, *J* = 8.6 Hz, 2H), 3.85 (t, *J* = 7.2 Hz, 1H), 3.71 (bs, 1H), 3.37 (bs, 1H), 3.13 (bs, 1H), 3.05 – 2.92 (m, 2H), 2.82 (dd, *J* = 13.0, 4.1 Hz, 1H), 2.68 (td, *J* = 12.1, 10.8, 5.2 Hz, 1H), 2.41 – 2.16 (m, 2H), 1.94 – 1.82 (m, 2H), 1.79 – 1.68 (m, 3H), 1.65 – 1.36 (m, 5H) ppm.

^{19}F NMR (376 MHz, $CDCl_3$): δ -65.02, -115.93 ppm.

^{13}C NMR (151 MHz, $CDCl_3$): δ 162.6 (d, J_{C-F} = 246.5 Hz), 138.9, 137.0 (d, J_{C-F} = 3.2 Hz), 128.7 (d, J_{C-F} = 8.3 Hz),128.0, 127.2, 115.8 (d, J_{C-F} = 20.9 Hz), 60.4, 58.6, 51.2, 39.1, 32.9 (q, J_{C-F} = 28.1 Hz), 24.1 (d, J_{C-F} = 3.3 Hz) ppm.

HRMS (ESI-TOF): calc'd for $C_{24}H_{29}F_4N_2$ $[M+H]^+$: 421.2267, found: 421.2266.

TLC: R_f = 0.54 (9:1 CH_2Cl_2:MeOH).

$[\alpha]_D^{20}$ = -88.46 (*c* = 0.92, MeOH).

Compound 75

Route A:

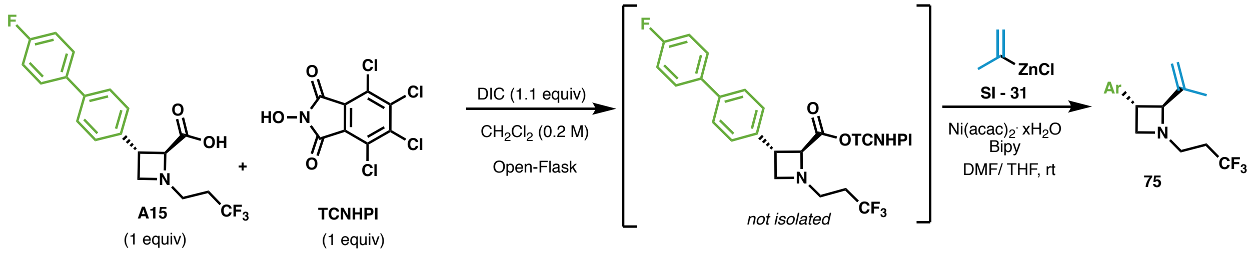

(2*S*,3*S*)-3-(4'-fluoro-[1,1'-biphenyl]-4-yl)-2-(prop-1-en-2-yl)-1-(3,3,3-trifluoropropyl)azetidine (75)

A culture tube was charged with carboxylic acid **A15** (36.7 mg, 0.1 mmol, 1.0 equiv.) and TCNHPI (38 mg, 0.1 mmol, 1.0 equiv.), CH_2Cl_2 (0.5 mL, anhydrous, 0.2 M) was added and DIC (17 µL, 0.11 mmol, 1.1 equiv.) was added dropwise. The reaction was monitored by TLC, after 1 hour all starting material was consumed and the solvent was removed under reduced pressure. The resulting crude Redox Active Ester was then used following the General Decarboxylation Procedure **C3** in the same reaction vessel. Following General Procedure **C3** on 0.1 mmol with the crude RAE, $Ni(acac)_2$•xH_2O (20 mol%), 2,2'-bipyridine (20 mol%), and Zinc reagent **SI-31** (3.0 equiv.). Purification by pTLC (silica, 4:1 Hexanes:EtOAc) afforded 5.1 mg (14%) of the title compound **75**.

Physical State: colorless oil.

^{1}H NMR (600 MHz, $CDCl_3$): δ 7.60 – 7.46 (m, 4H), 7.29 (d, J = 7.9 Hz, 2H), 7.12 (t, J = 8.7 Hz, 2H), 5.04 (s, 1H), 4.87 (s, 1H), 3.81 – 3.79 (m, 1H), 3.54 – 3.47 (m, 2H), 2.95 – 2.93 (m, 1H), 2.87 – 2.83 (m, 1H), 2.70 – 2.65 (m, 1H), 2.30 – 2.19 (m, 2H), 1.76 (s, 3H).

^{19}F NMR (376 MHz, $CDCl_3$): δ -115.86 ppm.

^{13}C NMR (151 MHz, $CDCl_3$): δ 162.6 (d, $J_{C\text{-}F}$ = 246.5 Hz), 139.0, 137.0 (d, $J_{C\text{-}F}$ = 3.3 Hz), 128.7 (d, $J_{C\text{-}F}$ = 8.2 Hz), 127.8, 127.3, 115.8 (d, $J_{C\text{-}F}$ = 21.4 Hz), 112.1, 79.0, 58.0, 51.2, 41.9, 32.6 (q, $J_{C\text{-}F}$ = 28.7 Hz), 18.8.

HRMS (ESI-TOF): calc'd for $C_{21}H_{22}F_4N$ $[M+H]^+$: 364.1688, found: 364.1689.

TLC: R_f = 0.3 (10:1 Hexanes:EtOAc).

$[\alpha]_D^{20}$ = 44.6 (c =0.1, $CHCl_3$).

Chiral SFC: IG column (3 µm, 4.6x250 mm) under isocratic conditions [3% MeOH / CO_2 (4

mL/min), 1600 psi backpressure] at 30 °C. The enantiomers were detected by UV light (260 nm). t_R (major) = 1.31 min, > 99 % *ee*.

Racemic:

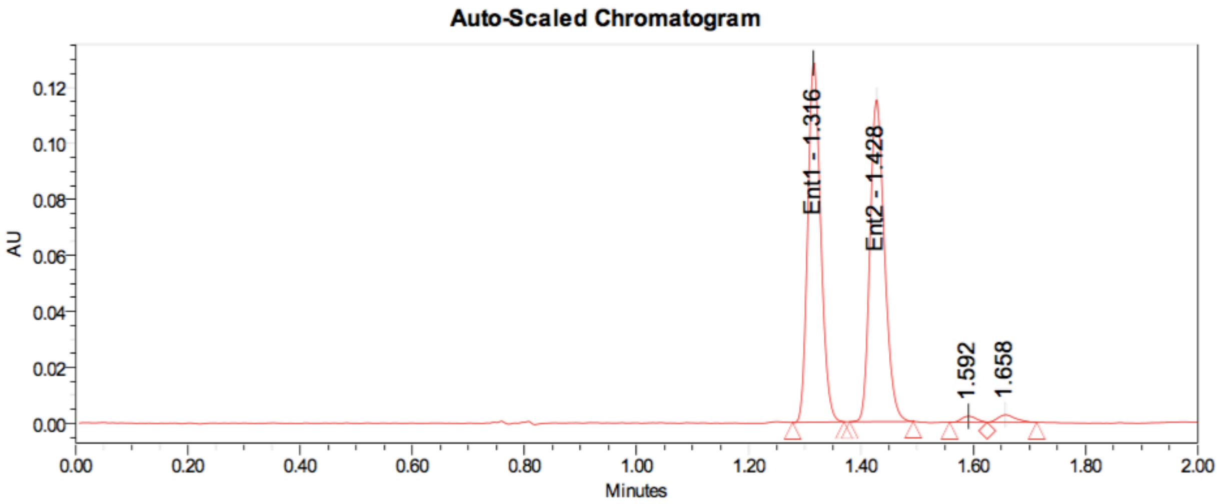

Enantioenriched **75**:

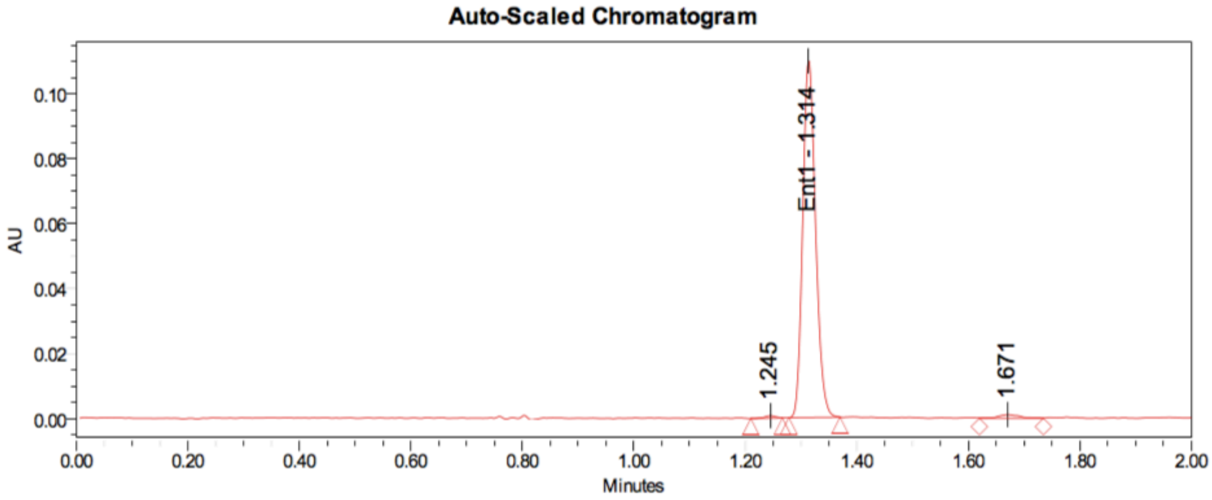

KSF-371	50.03	49.97	0.05	206771	206545
KSF-377	100.00		100.00	175697	

Route B:

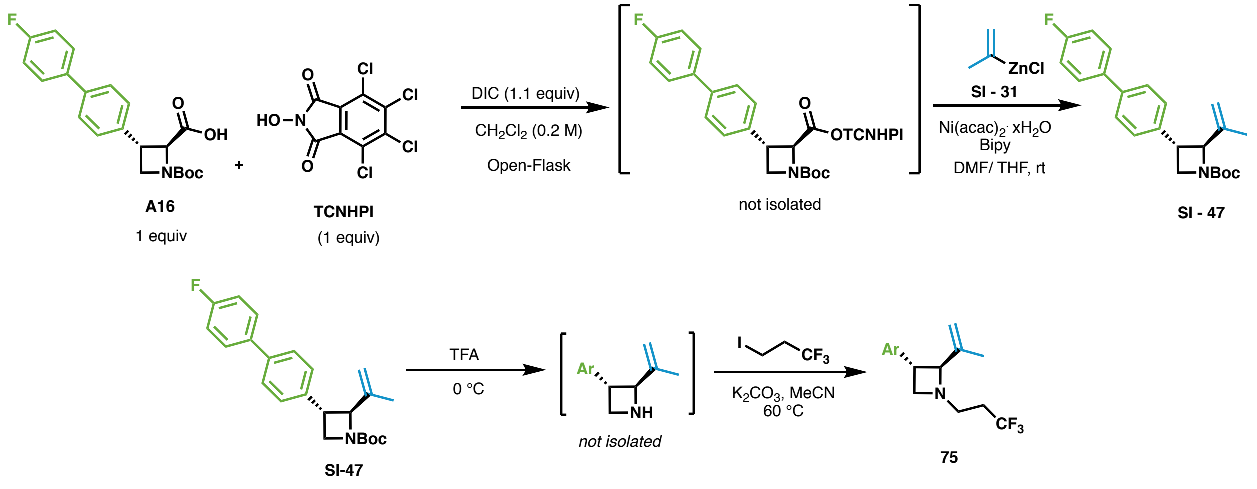

tert-butyl (2*S*,3*S*)-3-(4'-fluoro-[1,1'-biphenyl]-4-yl)-2-(prop-1-en-2-yl)azetidine-1-carboxylate (SI-47)

A culture tube was charged with carboxylic acid **A16** (37.1 mg, 0.1 mmol, 1.0 equiv.) and TCNHPI (38 mg, 0.1 mmol, 1.0 equiv.). CH_2Cl_2 (0.5 mL, anhydrous, 0.2 M) was added, then DIC (17 µL, 0.11 mmol, 1.1 equiv.) was added dropwise. The reaction was monitored by TLC, after 1 hour all starting material was consumed and the solvent was removed under reduced pressure. The resulting crude Redox Active Ester was then used following the General Decarboxylation Procedure **C3** in the same reaction vessel.

Following General Procedure **C3** on 0.1 mmol with the crude RAE, $Ni(acac)_2$•xH_2O (20 mol%), 2,2'-bipyridine (20 mol%), and Zinc reagent **SI-31** (3.0 equiv.). Purification by pTLC (silica, 9:1 Hexanes:EtOAc) afforded 15.6 mg (43%) of the title compound **SI-47**.

Physical State: yellow sticky oil.

^{1}H NMR (600 MHz, $CDCl_3$): δ 7.56 – 7.50 (m, 4H), 7.35 (d, J = 8.2 Hz, 2H), 7.12 (t, J = 8.7 Hz, 2H), 5.01 (s, 1H), 4.92 (s, 1H), 4.60 (d, J = 6.2 Hz, 1H), 4.23 (t, J = 8.6 Hz, 1H), 3.98 (t, J = 7.6 Hz, 1H), 3.55 – 3.27 (m, 1H), 1.81 (s, 3H), 1.46 (s, 9H) ppm.

^{19}F NMR (376 MHz, $CDCl_3$): δ -115.86 ppm.

^{13}C NMR (151 MHz, $CDCl_3$): δ 163.2 (d, $J_{C\text{-}F}$ = 246.5 Hz), 156.8, 143.7, 139.9 (d, $J_{C\text{-}F}$ = 205.8 Hz), 136.9 (d, $J_{C\text{-}F}$ = 3.2 Hz), 128.7 (d, $J_{C\text{-}F}$ = 8.0 Hz), 127.5 (d, $J_{C\text{-}F}$ = 19.3 Hz), 115.8 (d, $J_{C\text{-}F}$ = 21.4 Hz), 111.5, 79.9, 74.2, 66.0, 53.5, 40.6, 28.5, 17.7, 15.4 ppm.

HRMS (ESI-TOF): calc'd for $C_{23}H_{27}FNO_2$ $[M+H]^+$: 368.2026, found: 368.2021.

TLC: R$_f$ = 0.4 (9:1 Hexanes:EtOAc).

[α]$_D^{20}$ = +18.00 (*c* = 1, $CHCl_3$).

(2*S*,3*S*)-3-(4'-fluoro-[1,1'-biphenyl]-4-yl)-2-(prop-1-en-2-yl)-1-(3,3,3-trifluoropropyl)azetidine (75)

A culture tube equipped with a stir bar was charged with *tert*-butyl-(2*S*,3*S*)-3-(4'-fluoro-[1,1'-biphenyl]-4-yl)-2-(prop-1-en-2-yl)azetidine-1-carboxylate **(SI-47)** (36.7 mg, 0.1 mmol, 1.0 equiv.) and TFA (2.37 mL, 31 mmol) was added dropwise at 0 °C. The reaction was followed by TLC upon completion of starting material by TLC, 40 minutes. The crude mixture was concentrated *in vacuo* to remove the excess of TFA. Then, the crude mixture was diluted with EtOAc, quenched with saturated $NaHCO_3$ solution and extracted with EtOAc (3x). The organic layers were combined, dried over Na_2SO_4, filtered and concentrated *in vacuo*. The crude mixture was transferred to a culture tube and dried under high vacuum for at least 30 minutes, followed by the addition of K_2CO_3 (41.4 mg, 0.3 mmol, 3 equiv.). Then evacuated and backfilled with argon from a balloon (3x). Acetonitrile was added (5 mL, 0.02 M) followed by the addition of 1,1,1-Trifluoro-3-iodopropane (0.23 mL, 2 mmol, 20 equiv.). The tube was placed in an oil bath (preheated to 60 °C) for 12 hours. The reaction mixture was then allowed to cool to room temperature and more 1,1,1-Trifluoro-3-iodopropane (0.23 mL, 2 mmol, 20 equiv.) was added to the mixture. The tube was replaced in oil bath for further 12 hours. Then, after 48 hours the reaction was allowed to cool to room temperature and quenched with water. The resulting mixture was extracted with EtOAc (3x), the combined organic phases were dried over Na_2SO_4, filtered and concentrated *in vacuo*. The crude mixture was purified by flash column chromatographic (10:3 Hexanes:EtOAc) to afforded 3.4 mg (9.3 %) of the title compound **75**.

Compound 76

Route A:

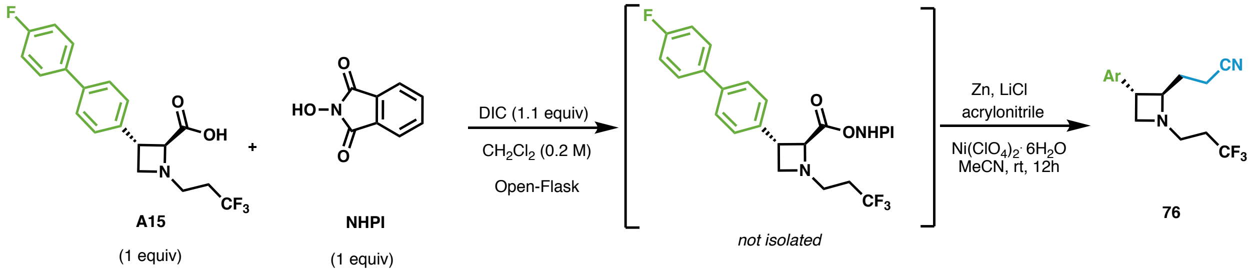

3-((2*R*,3*S*)-3-(4'-fluoro-[1,1'-biphenyl]-4-yl)-1-(3,3,3-trifluoropropyl)azetidin-2-yl)propanenitrile (76)

A culture tube was charged with carboxylic acid **A15** (36.7 mg, 0.1 mmol, 1.0 equiv.) and TCNHPI (38 mg, 0.1 mmol, 1.0 equiv.). CH_2Cl_2 (0.5 mL, anhydrous, 0.2 M) was added, then DIC (17 µL, 0.11 mmol, 1.1 equiv.) was added dropwise. The reaction was monitored by TLC, after 1 hour all starting material was consumed and the solvent was removed under reduced pressure. The resulting crude Redox Active Ester was then used in Giese Conjugate Addition reaction (General Procedure **C8**) in the same reaction vessel.

Following General Procedure **C8** on 0.1 mmol scale with the crude RAE and acrylonitrile. Purification by flash column chromatography (silica, 7:3 Hexanes:EtOAc) afforded 7.5 mg (20%) of the title compound **76**.

Physical State: colorless oil

^{1}H NMR (400 MHz, CDCl$_3$): δ 7.55 – 7.49 (m, 4H), 7.34 – 7.30 (m, 2H), 7.13 (t, J = 8.7 Hz, 2H), 3.85 (t, J = 6.8 Hz, 1H), 3.46 – 3.33 (m, 2H), 2.99 – 2.88 (m, 2H), 2.66 (ddd, J = 11.8, 9.0, 5.6 Hz, 1H), 2.51 – 2.34 (m, 2H), 2.31 – 2.16 (m, 2H), 2.05 – 2.00 (m, 2H) ppm.

^{19}F NMR (376 MHz, CDCl$_3$): δ -65.37, -115.77 ppm.

^{13}C NMR (151 MHz, CDCl$_3$): δ 162.65 (d, J_{C-F} = 246.5 Hz), 139.3, 139.1, 136.8 (d, J_{C-F} = 3.2 Hz), 128.7 (d, J_{C-F} = 8.0 Hz), 128.0, 127.4, 119.7, 115.8 (d, J_{C-F} = 21.4 Hz), 71.7, 58.8, 51.1 – 51.0 (m), 41.2, 32.8 (q, J_{C-F} = 28.2 Hz), 30.4, 13.1 ppm.

HRMS (ESI-TOF): calc'd for $C_{21}H_{20}F_4N_2$ $[M+H]^+$: 377.1641, found: 377.1642.

TLC: R_f = 0.3 (7:3 Hexanes:EtOAc).

$[\alpha]_D^{20}$ = -5.40 (c = 1, CHCl$_3$).

Route B:

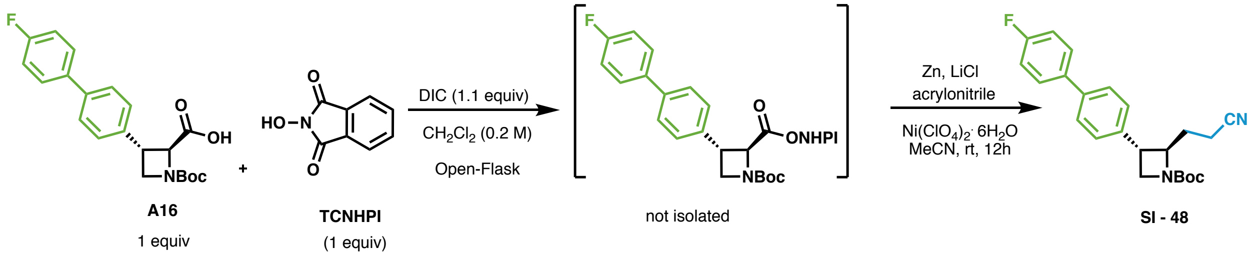

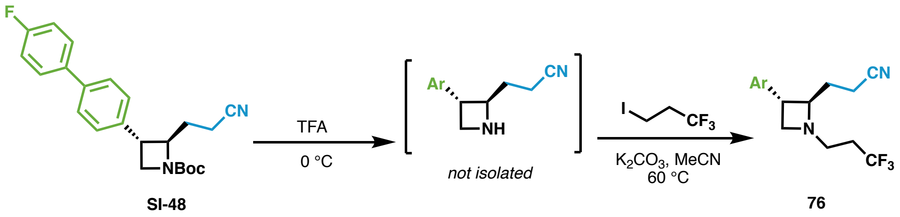

tert-butyl (2*R*,3*S*)-2-(2-cyanoethyl)-3-(4'-fluoro-[1,1'-biphenyl]-4-yl)azetidine-1-carboxylate (SI-48)

A culture tube was charged with carboxylic acid **A16** (184 mg, 0.5 mmol, 1.0 equiv.) and NHPI (89.7 mg, 0.55 mmol, 1.1 equiv.). CH_2Cl_2 (1.25 mL, anhydrous, 0.4 M) was added, then DIC (85 μL, 0.55 mmol, 1.1 equiv.) was added dropwise. The reaction was monitored by TLC, after 1 hour all starting material was consumed and the solvent was removed under reduced pressure. The resulting crude Redox Active Ester was then used in Giese Conjugate Addition reaction (General Procedure **C8**) in the same reaction vessel.

Following General Procedure **C8** on 0.5 mmol scale with the crude RAE and acrylonitrile. Purification by flash column chromatography (silica, 4:1 Hexanes:EtOAc) afforded 53.1 mg (28%) of the title compound **SI-48**.

Physical State: colorless oil.

^{1}H NMR (400 MHz, $CDCl_3$): δ 7.55 – 7.50 (m, 4H), 7.33 (d, J = 8.2 Hz, 2H), 7.16 – 7.10 (m, 2H), 4.31 (q, J = 6.2 Hz, 1H), 4.21 (t, J = 8.7 Hz, 1H), 3.95 (t, J = 7.6 Hz, 1H), 3.46 – 3.38 (m, 1H), 2.53 (dq, J = 16.7, 8.0, 7.4 Hz, 2H), 2.32 – 2.10 (m, 2H), 1.50 (bs, 9H) ppm.

^{19}F NMR (376 MHz, $CDCl_3$): δ -115.67 ppm.

^{13}C NMR (151 MHz, $CDCl_3$): δ 163.49, 161.86, 139.59, 139.48, 136.8 (d, $J_{C\text{-}F}$ = 3.2 Hz), 128.75, 128.70, 127.61, 127.52, 119.67, 115.92, 115.78, 80.63, 68.53, 54.27, 40.13, 28.54, 19.62, 13.98 ppm.

HRMS (ESI-TOF): calc'd for $C_{23}H_{26}FN_2O_2$ $[M+H]^+$: 381.1978, found: 381.1971.

TLC: R_f = 0.24 (4:1 Hexanes:EtOAc).

$[\alpha]_D^{20}$ = 19.37 (c = 0.95, $CHCl_3$).

3-((2*R*,3*S*)-3-(4'-fluoro-[1,1'-biphenyl]-4-yl)-1-(3,3,3-trifluoropropyl)azetidin-2-yl)propanenitrile (76)

A culture tube equipped with a stir bar was charged with *tert*-butyl-(2*R*,3*S*)-2-(2-cyanoethyl)-3-(4'-fluoro-[1,1'-biphenyl]-4-yl)azetidine-1-carboxylate **(SI-48)** (37.4 mg, 0.1 mmol, 1.0 equiv.) and TFA (2.37 mL, 31 mmol) was added dropwise at 0 °C. The reaction was followed by TLC upon completion of starting material by TLC, 40 minutes. The crude mixture was concentrated *in vacuo* to remove the excess of TFA. Then, the crude mixture was diluted with EtOAc, quenched with saturated $NaHCO_3$ solution and extracted with EtOAc (3x). The organic layers were combined and dried over Na_2SO_4, filtered and concentrated *in vacuo*. The crude mixture was transferred to a culture tube and dried under high vacuum for at least 30 minutes, followed by the addition of K_2CO_3 (41.4 mg, 0.3 mmol,3 equiv.).Then, the tube was evacuated and backfilled with argon from a balloon (3x). Acetonitrile was added (5 mL, 0.02 M) followed addition of 1,1,1-Trifluoro-3-iodopropane (0.23 mL, 2 mmol, 20 equiv.). The tube was placed in an oil bath (preheated to 60 °C) for 12 hours. The reaction mixture was then allowed to cool to room temperature and more 1,1,1-Trifluoro-3-iodopropane (0.23 mL, 2 mmol, 20 equiv.) were added to the mixture. The tube was replaced in oil bath for further 12 hours. Then, after 48 hours the reaction was allowed to cool to room temperature and quenched with water. The resulting mixture was extracted with EtOAc (3x). The combined organic layers were dried over Na_2SO_4, filtered and concentrated *in vacuo*. The crude mixture was purified by flash column chromatographic (silica, 7:3 Hexanes:EtOAc) to afforded 14.2 mg (42 %) of the title compound **76**.

Compound 77

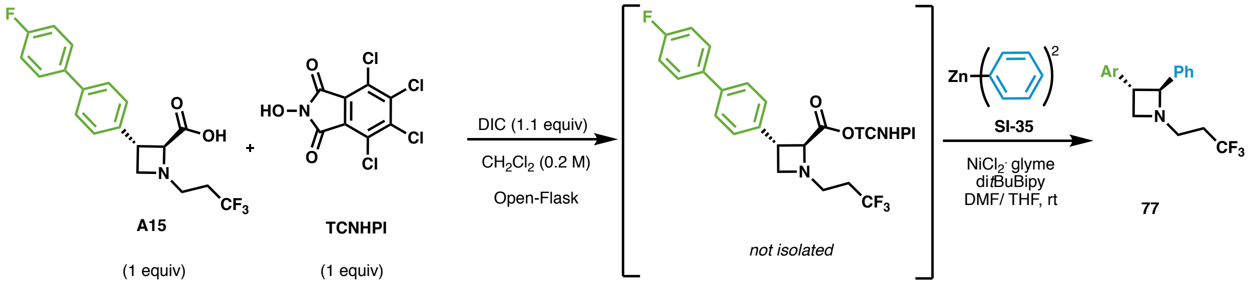

(2*S*,3*S*)-3-(4'-fluoro-[1,1'-biphenyl]-4-yl)-2-phenyl-1-(3,3,3-trifluoropropyl)azetidine (77)

A culture tube was charged with carboxylic acid **A15** (18.4 mg, 0.05 mmol, 1.0 equiv.) and TCNHPI (19 mg, 0.05 mmol, 1.0 equiv.), CH_2Cl_2 (1 mL, anhydrous, 0.05 M) was added, then DIC (17 µL, 0.11 mmol, 1.1 equiv.) was added dropwise. The reaction was monitored by TLC, after 1 hour all starting material was consumed and the solvent was removed reduced pressure. The resulting crude Redox Active Ester was then used following the General Decarboxylation Procedure **C4** in the same reaction vessel.

Following General Procedure **C4** on 0.1 mmol with the crude RAE, $NiCl_2$•glyme (20 mol%), di*t*BuBipy (40 mol%), and Zinc reagent **SI-35** (3.0 equiv.). Purification by pTLC (silica, 2% EtOAc in Hexanes) afforded 3.2 mg (16 %) of the title compound **77**.

Physical State: colorless oil

^{1}H NMR (600 MHz, $CDCl_3$): δ 7.54 – 7.50 (m, 2H), 7.48 (d, J = 8.1 Hz, 2H), 7.43 (d, J = 7.5 Hz, 2H), 7.36 (t, J = 7.4 Hz, 2H), 7.31 – 7.27 (m, 3H), 7.12 (t, J = 8.7 Hz, 2H), 4.05 (d, J = 8.3 Hz, 1H), 3.91 (t, J = 6.9 Hz, 1H), 3.57 (d, J = 8.6 Hz, 1H), 3.11 (t, J = 7.7 Hz, 1H), 2.89 (q, J = 9.6, 8.5 Hz, 1H), 2.77 (q, J = 10.6, 9.3 Hz, 1H), 2.18 (s, 2H) ppm.

^{19}F NMR (376 MHz, $CDCl_3$): δ -65.52, -116.01ppm.

^{13}C NMR (151 MHz, $CDCl_3$): δ 162.4 (d, J_{C-F} = 246.2 Hz), 141.8, 139.6, 138.9, 137.0 (d, J_{C-F} = 3.2 Hz), 128.7, 128.7, 127.9, 127.9, 127.4, 127.2, 126.9, 126.5 (d, J_{C-F} = 276.3 Hz), 123.8, 115.8 (d, J_{C-F} = 21.3 Hz), 57.6, 51.2, 46.0, 32.9 (q, J_{C-F} = 28.3 Hz).

TLC: R_f = 0.2 (9:1 Hexanes: EtOAc).

$[\alpha]_D^{20}$ = 4.78 (c = 0.23, $CHCl_3$).

*Note: no HRMS could be recorded for **80**.*

Route B:

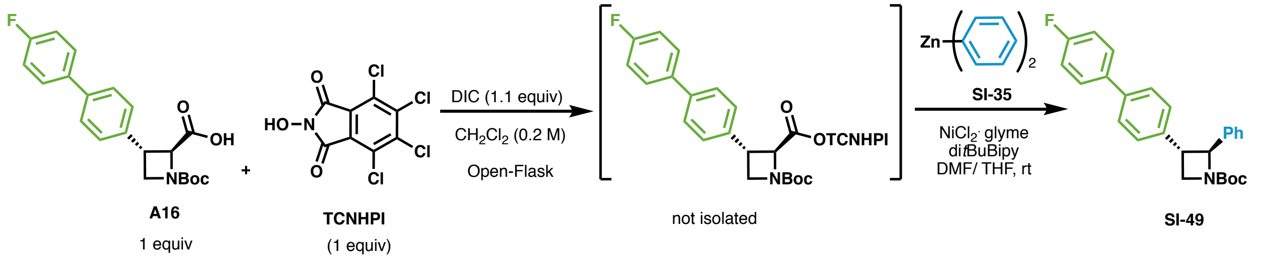

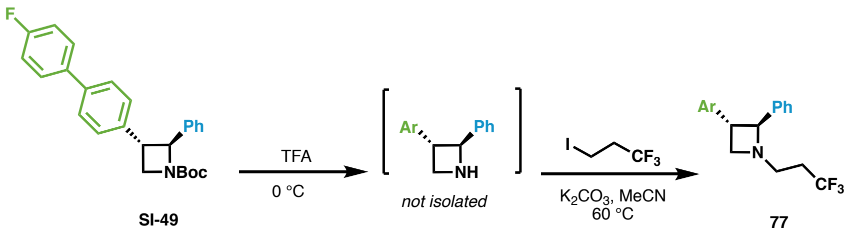

***tert*-butyl (2*S*,3*S*)-3-(4'-fluoro-[1,1'-biphenyl]-4-yl)-2-phenylazetidine-1-carboxylate (SI-49)**

A culture tube was charged with carboxylic acid **A16** (37.1 mg, 0.1 mmol, 1.0 equiv.) and TCNHPI (38 mg, 0.1 mmol, 1.0 equiv). CH_2Cl_2 (0.5 mL, anhydrous, 0.2 M) was added, then DIC (17 µL, 0.11 mmol, 1.1 equiv.) was added dropwise. The reaction was monitored by TLC, after 1 hour all starting material was consumed and the solvent was removed under reduced pressure. The resulting crude Redox Active Ester was then used following the General Decarboxylation Procedure **C4** in the same reaction vessel.

Following General Procedure **C4** on 0.1 mmol with the crude RAE, $NiCl_2$•glyme (20 mol%), di*t*BuBipy (40 mol%), and Zinc reagent **SI-35** (3.0 equiv.). Purification by flash column chromatography (silica, 9:1 Hexanes:EtOAc) afforded 22.3 mg (55 %) of the title compound **SI-49**.

Physical State: colorless sticky oil.

^{1}H NMR (600 MHz, $CDCl_3$): δ 7.57 – 7.51 (m, 4H), 7.38 (dd, J = 6.0, 3.6 Hz, 6H), 7.31 (dt, J = 5.8, 2.8 Hz, 1H), 7.16 – 7.08 (m, 2H), 5.20 – 5.07 (m, 1H), 4.40 (t, J = 8.6 Hz, 1H), 4.20 – 4.03 (m, 1H), 3.62 (dt, J = 8.7, 6.5 Hz, 1H), 1.36 (s, 9H) ppm.

^{19}F NMR (376 MHz, $CDCl_3$): δ -115.81 ppm.

^{13}C NMR (151 MHz, $CDCl_3$): δ 162.6 (d, J_{C-F} = 249.3 Hz), 156.9, 141.6, 140.1, 139.3, 136.9 (d, J_{C-F} = 3.3 Hz), 128.8 – 128.6 (m), 127.8 - 127.4 (m), 125.97, 115.8 (d, J_{C-F} = 21.5 Hz), 80.0, 72.6, 53.6, 44.2, 28.4 ppm.

HRMS (ESI-TOF): calc'd for $C_{26}H_{27}FNO_2$ [M-Boc+H]$^+$: 304.1502, found: 304.1506.

TLC: R_f = 0.45 (9:1 Hexanes:EtOAc).

(2*S*,3*S*)-3-(4'-fluoro-[1,1'-biphenyl]-4-yl)-2-phenyl-1-(3,3,3-trifluoropropyl)azetidine (77)

A culture tube equipped with stir bar was charged with *tert*-butyl-(2*S*,3*S*)-3-(4'-fluoro-[1,1'-biphenyl]-4-yl)-2-phenylazetidine-1-carboxylate **(SI-49)** (40.3 mg, 0.1 mmol, 1.0 equiv.) and TFA (2.37 mL) was added dropwise at 0°C. The reaction was followed by TLC upon completion of starting material by TLC, 40 minutes. The crude mixture was concentrated *in vacuo* to remove the excess of TFA. Then, the crude mixture was diluted with EtOAc, quenched with saturated $NaHCO_3$ solution and extracted with EtOAc (3x). The organic layers were combined and dried over Na_2SO_4, filtered and concentrated *in vacuo*. The crude mixture was transferred to a culture tube and dried under high vacuum for at least 30 minutes, followed by the addition of K_2CO_3 (41.4 mg, 0.3 mmol, 3 equiv.). Then, the tube was evacuated and backfilled with argon from a balloon (3x). Acetonitrile was added (5 mL, 0.02 M) followed addition of 1,1,1-Trifluoro-3-iodopropane (0.23 mL, 2 mmol, 20 equiv.). The tube was placed in an oil bath (preheated to 60 °C) for 12 hours. The reaction mixture was then allowed to cool to room temperature and more 1,1,1-Trifluoro-3-iodopropane (0.23 mL, 2 mmol, 20 equiv.) were added to the mixture. The tube was replaced in oil bath for further 12 hours. Then, after 48 hours the reaction was allowed to cool to room temperature and quenched with water. The resulting mixture was extracted with EtOAc (3x). The combined organic layers were dried over Na_2SO_4, filtered and concentrated *in vacuo*. The crude mixture was purified by flash column chromatographic (silica, 1:9 EtOAc:Hexanes) to afforded 3.6 mg (9 %) of the title compound **77**.

Compound SI-50

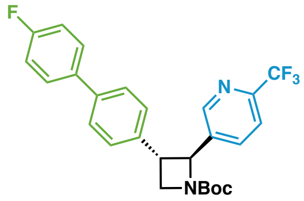

***tert*-butyl (2*S*,3*S*)-3-(4'-fluoro-[1,1'-biphenyl]-4-yl)-2-(6-(trifluoromethyl)pyridin-3-yl)azetidine-1-carboxylate (SI-50)**

Following General Procedure **C1** on 0.15 mmol scale with **B9**, $NiCl_2$•$6H_2O$ (40 mol%), BPhen (40 mol%), and boronic acid **SI-14** (3.0 equiv.). Purification by flash column chromatography (silica, 5:1 Hexanes:EtOAc) afforded 26.1mg (37%) of the title compound **SI-50**.

Physical State: colorless sticky oil.

^{1}H NMR (600 MHz, $CDCl_3$): δ 8.71 (bs, 1H), 7.90 (dd, *J* = 8.3, 1.7 Hz, 1H), 7.71 (d, *J* = 8.1 Hz, 1H), 7.58 – 7.51 (m, 4H), 7.36 (d, *J* = 8.3 Hz, 2H), 7.16 – 7.11 (m, 2H), 5.26 (d, *J* = 6.1 Hz, 1H), 4.38 (t, *J* = 8.6 Hz, 1H), 4.21 (t, *J* = 8.3 Hz, 1H), 3.63 (q, *J* = 7.2 Hz, 1H), 1.40 (s, 9H) ppm.

^{19}F NMR (376 MHz, $CDCl_3$): -68.01, -115.46 ppm.

^{13}C NMR (151 MHz, $CDCl_3$): δ 162.8 (d, J_{C-F} = 246.8 Hz), 156.9, 148.0, 140.1 (d, J_{C-F} = 33.7 Hz), 138.5, 136.7 (d, J_{C-F} = 3.1 Hz), 134.8, 128.8 (d, J_{C-F} = 8.1 Hz), 127.7 (d, J_{C-F} = 20.5 Hz), 120.6, 115.9 (d, J_{C-F} = 21.6 Hz), 81.0, 69.7, 43.9, 29.8, 28.4 ppm.

HRMS (ESI-TOF): calc'd for $C_{26}H_{25}F_4N_2O_2$ [M+H]: 473.1852, found: 473.1852.

TLC: R_f = 0.41 (4:1 Hexanes:EtOAc).

$[\alpha]_D^{20}$ = 60.27 (*c* = 0.37, $CHCl_3$).

Compound 78

Route B:

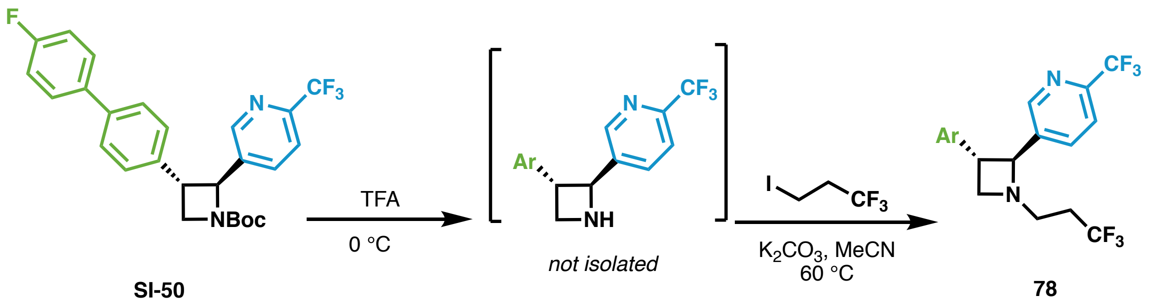

5-((2*S*,3*S*)-3-(4'-fluoro-[1,1'-biphenyl]-4-yl)-1-(3,3,3-trifluoropropyl)azetidin-2-yl)-2-(trifluoromethyl)pyridine (78)

A culture tube equipped with a stir bar was charged *tert*-butyl (2*S*,3*S*)-3-(4'-fluoro-[1,1'-biphenyl]-4-yl)-2-(6-(trifluoromethyl)pyridin-3-yl)azetidine-1-carboxylate **SI-50** (15.5 mg, 0.03 mmol, 1.0 equiv.) and TFA (0.78 mL) was added dropwise at 0 °C. The reaction was followed by TLC upon completion of starting material by TLC, 40 minutes. The crude mixture was concentrated *in vacuo* to remove the excess of TFA. Then, the crude mixture was diluted with EtOAc, quenched with saturated $NaHCO_3$ solution and extracted with EtOAc (3x). The organic layers were combined, dried over Na_2SO_4, filtered and concentrated *in vacuo*. The crude mixture was transferred to a culture tube and dried under high vacuum for at least 30 minutes, followed by the addition of K_2CO_3 (13.8 mg, 0.1 mmol, 3 equiv.). Then, the tube was evacuated and backfilled with argon from a balloon (3x). Acetonitrile was added (1.6 mL, 0.02 M) followed addition of 1,1,1-Trifluoro-3-iodopropane (80 µL, 0.66 mmol, 20 equiv.). The tube was placed in an oil bath (preheated to 60 °C) for 12 hours. The reaction mixture was then allowed to cool to room temperature and more 1,1,1-Trifluoro-3-iodopropane (80 µL, 0.656 mmol, 20 equiv.) were added to the mixture. The tube was replaced in oil bath for further 12 hours. Then, after 48 hours the reaction was allowed to cool to room temperature and quenched with water. The resulting mixture was extracted with EtOAc (3x). The combined organic layers were dried over Na_2SO_4, filtered and concentrated *in vacuo*. The crude mixture was purified by pTLC (silica, 9:1 Hexanes:EtOAc) to afforded 2.6 mg (17 %) of the title compound **78**.

Physical State: colorless sticky oil

^{1}H NMR (600 MHz, $CDCl_3$): δ 8.74 – 8.69 (m, 1H), 7.98 (s, 1H), 7.70 (d, J = 8.0 Hz, 1H), 7.55 – 7.47 (m, 4H), 7.27 (s, 2H), 7.13 (t, J = 8.7 Hz, 2H), 4.17 (d, J = 7.3 Hz, 1H), 3.97 (m, 1H), 3.53 (d, J = 7.2 Hz, 1H), 3.22 (m, 1H), 2.88 (m, 1H), 2.79 (m, 1H), 2.20 (m, 2H) ppm.

^{13}C NMR (151 MHz, $CDCl_3$): δ 162.1 (d, $J_{C\text{-}F}$ = 246.5 Hz), 148.1, 138.9, 137.5, 136.1 (d, $J_{C\text{-}F}$ = 3.3 Hz), 135.2, 128.1 (d, $J_{C\text{-}F}$ = 8.1 Hz), 127.2, 126.9, 120.1, 115.3 (d, $J_{C\text{-}F}$ = 21.5 Hz), 73.6, 57.1, 50.2, 45.6, 32.53 - 31.9 (m) ppm.

^{19}F NMR (376 MHz, $CDCl_3$): δ -65.38, -68.01, -115.65 ppm.

HRMS (ESI-TOF): calc'd for $C_{24}H_{20}F_7N_2$ $[M+H]^+$: 469.1515, found: 469.1516.

TLC: R_f = 0.2 (9:1 Hexanes:EtOAc).

$[\alpha]_D^{20}$ = 61.36 (c = 0.22, $CHCl_3$).

Compound 80

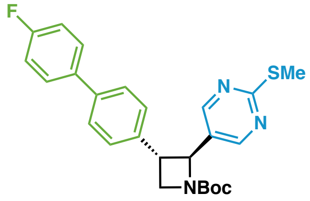

tert-butyl (2S,3S)-3-(4'-fluoro-[1,1'-biphenyl]-4-yl)-2-(2-(methylthio)pyrimidin-5-yl)azetidine-1-carboxylate (80)

Following General Procedure **C1** on 0.038 mmol scale with **B9**, $NiCl_2•6H_2O$ (40 mol%), BPhen (40 mol%), and boronic acid **SI-18** (3.0 equiv.). Purification by pTLC (silica, 3:3:14 Hexanes: Ether:EtOAc) afforded 8.6 mg (50%) of the title compound **80**.

Physical State: white solid.

^{1}H NMR (600 MHz, $CDCl_3$): δ 8.56 (s, 2H), 7.58 – 7.49 (m, 4H), 7.34 (d, J = 8.2 Hz, 2H), 7.14 (t, J = 8.7 Hz, 2H), 5.09 (d, J = 5.7 Hz, 1H), 4.36 (t, J = 8.5 Hz, 1H), 4.18 (t, J = 7.8 Hz, 1H), 3.66 (q, J = 7.1 Hz, 1H), 2.59 (s, 3H), 1.41 (s, 9H) ppm.

^{19}F NMR (376 MHz, $CDCl_3$): δ -115.51 ppm.

^{13}C NMR (151 MHz, $CDCl_3$): δ 171.8, 162.1 (d, J_{C-F} = 247.1 Hz), 156.7, 156.3, 154.9, 139.3, 137.9, 136.0 (d, J_{C-F} = 3.2 Hz), 128.7, 128.2 (d, J_{C-F} = 7.7 Hz), 127.0 (d, J_{C-F} = 24.2 Hz), 115.4, 115.3 (d, J_{C-F} = 21.5 Hz), 80.3, 43.0, 27.8, 13.7 ppm.

HRMS (ESI-TOF): calc'd for $C_{25}H_{27}FN_3O_2S$ $[M+H]^+$: 452.1808, found: 452.1809.

TLC: R_f = 0.35 (3:3:14 Hexanes:Ether:EtOAc)

$[\alpha]_D^{20}$ = +93.61 (c = 0.36, $CHCl_3$).

Compound 79

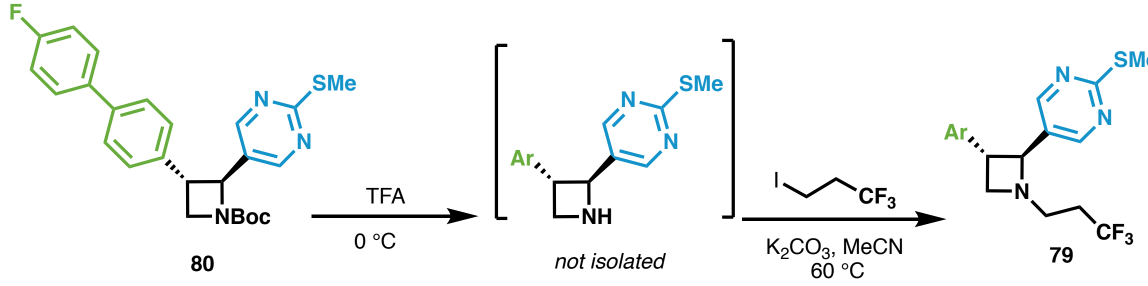

5-((2*S*,3*S*)-3-(4'-fluoro-[1,1'-biphenyl]-4-yl)-1-(3,3,3-trifluoropropyl)azetidin-2-yl)-2-(methylthio)pyrimidine (79)

A culture tube equipped with a stir bar was charged with *tert*-butyl-(2*S*,3*S*)-3-(4'-fluoro-[1,1'-biphenyl]-4-yl)-2-phenylazetidine-1-carboxylate **80** (1 mg, 0.0022 mmol, 1.0 equiv.) and TFA (0.1 mL) was added dropwise at 0 °C. The reaction was followed by TLC upon completion of starting material by TLC, 40 minutes. The crude mixture was concentrated *in vacuo* to remove the excess of TFA. Then, the crude mixture was diluted with EtOAc, quenched with saturated $NaHCO_3$ solution and extracted with EtOAc (3x). The organic layers were combined and dried over Na_2SO_4, filtered and concentrated *in vacuo*. The crude mixture was transferred to a culture tube and dried under high vacuum for at least 30 minutes, followed by the addition of K_2CO_3 (41.4 mg, 0.3 mmol,3 equiv.). Then, the tube was evacuated and backfilled with argon from a balloon (3x). Acetonitrile was added (5 mL, 0.02 M) followed addition of 1,1,1-Trifluoro-3-iodopropane (0.23 mL, 2 mmol, 20 equiv.). The tube was placed in an oil bath (preheated to 60 °C) for 12 hours. The reaction mixture was then allowed to cool to room temperature and more 1,1,1-Trifluoro-3-iodopropane (0.23 mL, 2 mmol, 20 equiv.) were added to the mixture. The tube was replaced in oil bath for further 12 hours. Then, after 48 hours the reaction was allowed to cool to room temperature and quenched with water. The resulting mixture was extracted with EtOAc (3x). The combined organic layers were dried over Na_2SO_4, filtered and concentrated *in vacuo*. The crude mixture was purified by flash column chromatographic (silica, 5:4 Hexanes:EtOAc) to afforded 1.4 mg 61 %) of the title compound **79**.

Physical State: white solid.

^{1}H NMR (600 MHz, $CDCl_3$): δ 8.58 (s, 2H), 7.53 – 7.43 (m, 4H), 7.24 (d, *J* = 8.2 Hz, 2H), 7.10 (t, *J* = 8.7 Hz, 2H), 3.98-3.93 (m, 2H), 3.54 (d, *J* = 12 Hz, 1H), 3.17-3.15 (m, 1H), 2.87-2.83 (m, 1H), 2.77-2.72 (m, 1H), 2.56 (s, 3H), 2.19-2.16 (m, 2H) ppm.

^{19}F NMR (376 MHz, $CDCl_3$): δ -65.43, -115.71 ppm.

Compound 81

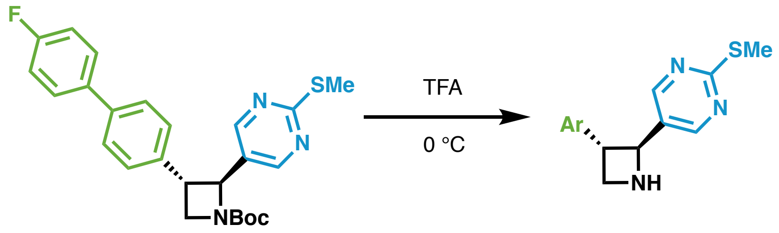

80 81

5-((2*S*,3*S*)-3-(4'-fluoro-[1,1'-biphenyl]-4-yl)azetidin-2-yl)-2-(methylthio)pyrimidine (81)

A culture tube equipped with a stir bar was charged with *tert-butyl* (2*S*,3*S*)-3-(4'-fluoro-[1,1'-biphenyl]-4-yl)-2-(2-(methylthio)pyrimidin-5-yl)azetidine-1-carboxylate **80** (2 mg, 4.4 µmol, 1.0 equiv.) and TFA (0.2 mL) was added dropwise at 0 °C. The reaction was followed by TLC upon completion of starting material by TLC, 40 minutes. The crude mixture was concentrated *in vacuo* to remove the excess of TFA. Then, the crude mixture was diluted with EtOAc (1 mL), quenched with saturated $NaHCO_3$ solution and extracted with EtOAc (3x 1 mL). The organic layers were combined and dried over Na_2SO_4, filtered and concentrated *in vacuo* to afford the desired compound **81** (1.5 mg, 98%) as a colorless oil.

Physical State: colorless oil.

^{1}H NMR (600 MHz, $CDCl_3$): δ 8.33 (s, 2H), 7.47 (dd, *J* = 8.3, 5.1 Hz, 4H), 7.16 – 7.10 (m, 4H), 5.75 (s, 1H), 5.44 (d, *J* = 10.7 Hz, 1H), 3.77 (t, *J* = 11.7 Hz, 1H), 3.72 – 3.62 (m, 1H), 3.37 (dt, *J* = 11.0, 5.5 Hz, 1H), 2.50 (s, 3H).

^{19}F NMR (376 MHz, $CDCl_3$): δ -115.05 ppm.

^{13}C NMR (151 MHz, $CDCl_3$): δ 173.2, 162.9 (d, J_{C-F} = 247.1 Hz), 155.9, 153.3, 140.7, 136.1 (d, J_{C-F} = 3.3 Hz), 133.9, 128.9, 128.8, 128.8, 128.7, 128.1, 125.7, 116.0 (d, J_{C-F} = 21.7 Hz), 79.5, 46.4, 44.6, 14.3.

HRMS (ESI-TOF): calc'd for $C_{20}H_{19}FN_3S$ $[M+H]^+$: 352.1284, found: 352.1287.

TLC: R_f = 0.2 (pure EtOAc).

$[\alpha]_D^{20}$ = 71.1 (*c* =0.2, $CHCl_3$).

Compound 82

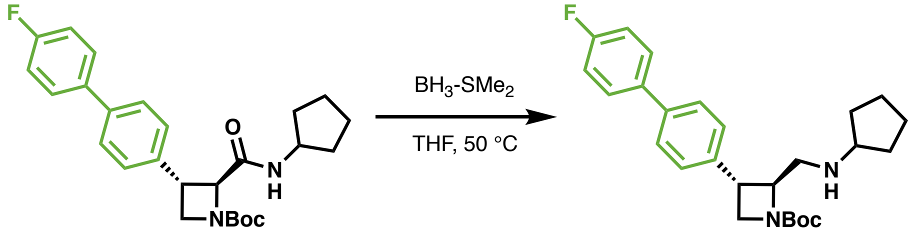

82

***tert*-butyl (2*S*,3*S*)-2-((cyclopentylamino)methyl)-3-(4'-fluoro-[1,1'-biphenyl]-4-yl)azetidine-1-carboxylate (82)**

To a reaction vessel containing *tert*-butyl (2*S*,3*S*)-2-(cyclopentylcarbamoyl)-3-(4'-fluoro-[1,1'-biphenyl]-4-yl)azetidine-1-carboxylate (44 mg, 0.1 mmol, 1.0 equiv.) was added THF (0.5 mL, 0.2 M). The reaction mixture was cooled to 0 °C before the slow addition of a 2 M solution of Borane-dimethyl sulfide complex in THF (0,15 mL, 0.3 mmol, 3 equiv.). The reaction mixture was allowed to warm to room temperature, followed by TLC, upon full conversion of starting material. The mixture was then cooled in an ice bath, and MeOH (1 mL) was added dropwise. The mixture was stirred at room temperature for 20 minutes, then Rochelle salt's solution (3 mL) was added and the reaction mixture was stirred overnight. The clear solution was extracted with EtOAc (3x 2 mL) and the combined organics were washed with brine (1x 8 mL), dried over Na2SO4 and concentrated *in vacuo*. Purification by flash column chromatography (silica, 1:2 Hexanes:EtOAc) afforded 36 mg (84%) of the title compound **82** as a colorless oil.

Physical State: colorless oil.

^{1}H NMR (600 MHz, CDCl$_3$): δ 7.59 – 7.41 (m, 4H), 7.34 (d, *J* = 8.1 Hz, 2H), 7.12 (t, *J* = 8.7 Hz, 2H), 4.40 (s, 1H), 4.18 (t, *J* = 8.6 Hz, 1H), 3.94 (t, *J* = 7.4 Hz, 1H), 3.59 (q, *J* = 6.7 Hz, 1H), 3.17 – 3.08 (m, 1H), 3.04 (m, 1H), 2.96 (m, 1H), 1.89 – 1.80 (m, 2H), 1.76 – 1.63 (m, 4H), 1.54 (d, *J* = 4.3 Hz, 1H), 1.48 (s, 9H), 1.37 (s, 2H) ppm.

^{19}F NMR (376 MHz, CDCl$_3$): δ -115.99 ppm.

^{13}C NMR (151 MHz, CDCl$_3$): δ 162.62 (d, *J* = 246.4 Hz), 139.11, 136.97 (d, *J* = 3.3 Hz), 128.71 (d, *J* = 8.1 Hz), 127.63, 127.39, 115.80 (d, *J* = 21.4 Hz), 80.15, 59.99, 52.64, 37.98, 33.20, 29.85, 28.61, 28.55, 24.16 ppm.

HRMS (ESI-TOF): calc'd for $C_{20}H_{19}FN_3S$ $[M+H]^+$: 425.2604, found: 425.2607.

TLC: R_f = 0.33 (1:2 Hexanes:EtOAc).

$[\alpha]_D^{20}$ = 59.2 (*c* =0.1, CHCl$_3$).

Compound 83

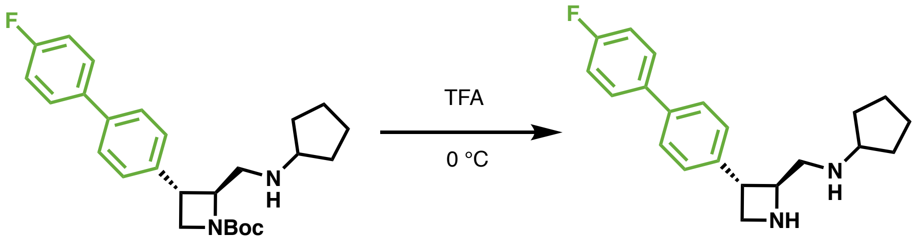

82 **83**

***N*-(((2*S*,3*S*)-3-(4'-fluoro-[1,1'-biphenyl]-4-yl)azetidin-2-yl)methyl)cyclopentanamine (83)**

A culture tube equipped with a stir bar was charged with *tert*-butyl (2*S*,3*S*)-2-((cyclopentylamino)methyl)-3-(4'-fluoro-[1,1'-biphenyl]-4-yl)azetidine-1-carboxylate **82** (5 mg, 12 µmol, 1.0 equiv.) and TFA (0.6 mL) was added dropwise at 0 °C. The reaction was followed by TLC upon completion of starting material by TLC, 40 minutes. The crude mixture was concentrated *in vacuo* to remove the excess of TFA. Then, the crude mixture was diluted with EtOAc (2 mL), quenched with saturated $NaHCO_3$ solution and extracted with EtOAc (3x 1 mL). The organic layers were combined and dried over Na_2SO_4, filtered and concentrated *in vacuo* to afford the desired compound **83** (1.5 mg, 98%) as a colorless oil.

Physical State: colorless oil.

^{1}H NMR (600 MHz, $CDCl_3$): δ 7.61 (d, *J* = 7.4 Hz, 2H), 7.57 – 7.51 (m, 2H), 7.37 (d, *J* = 7.7 Hz, 2H), 7.17 (t, *J* = 7.6 Hz, 2H), 5.16 (s, 1H), 4.29 (d, *J* = 46.1 Hz, 1H), 4.07 (s, 1H), 3.94 (m, 1H), 3.47 (m, 1H), 3.16 (d, *J* = 12.6 Hz, 1H), 2.12 – 1.84 (m, 6H), 1.78 (m, 2H), 1.66 (m, 1H).

^{13}C NMR (151 MHz, $CDCl_3$): δ 162.3 (d, *J* = 247.6 Hz), 140.7, 135.6 (d, *J* = 3.2 Hz), 133.6, 128.2 (d, *J* = 8.1 Hz), 127.6, 127.0, 115.5 (d, *J* = 21.6 Hz), 61.8, 60.67, 49.0, 45.4, 41.3, 29.96 – 28.47 (m), 23.33.

^{19}F NMR (376 MHz, $CDCl_3$): δ -115.81 ppm.

HRMS (ESI-TOF): calc'd for $C_{20}H_{19}FN_3S$ $[M+H]^+$: 325.2080, found: 325.2078.

TLC: R_f = 0.25 (2:8 MeOH:DCM).

$[\alpha]_D^{20}$ = 64.3 (*c* =0.1, $CHCl_3$).

Biological assays: Comments, Procedures and Results (Figure 5-c)

Importance of the trans-relationship between the biaryl substituent and the vicinal group

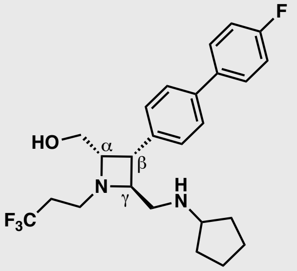

BRD8468 (**74**)
PubChem CID 60182543

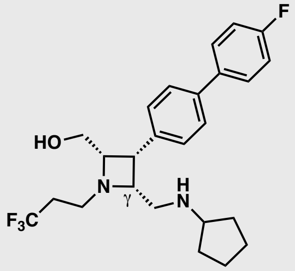

BRD5530
PubChem CID 60182511

Pf **EC_{50}** 0.11–0.49 μM (Dd2 strain)
0.26 μM (3D7-*Sc*DHODH)
0.24 μM (NITD609-resistant)

Pf **EC_{50}** 2.51 μM (Dd2 strain)

HepG2 CC_{50} 21 μM

HepG2 CC_{50} 16.49 μM

BRD8468 is a more potent *P. falciparum* inhibitor than its γ-epimer BRD5530, and remains equipotent against *Pf* 3D7-*Sc*DHODH and NITD609-resistant *Pf*. All data were retrieved from PubChem (https://pubchem.ncbi.nlm.nih.gov/) using the CID compound identifiers shown above.

Example of tolerated diversity at azetidine nitrogen

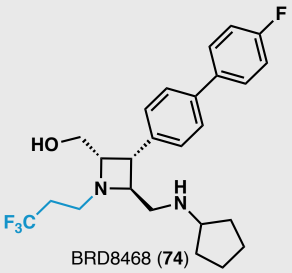

BRD8468 (**74**)
PubChem CID 60182543
Pf **EC_{50}** 0.11–0.49 μM

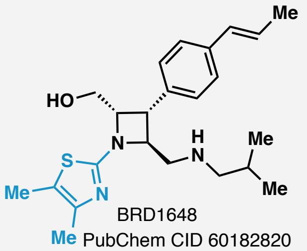

BRD1648
PubChem CID 60182820
Pf **EC_{50}** 0.23 μM

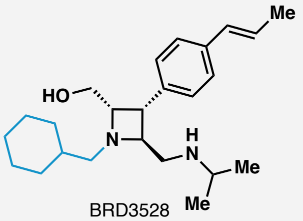

BRD3528
PubChem CID 60182585
Pf **EC_{50}** 0.23 μM

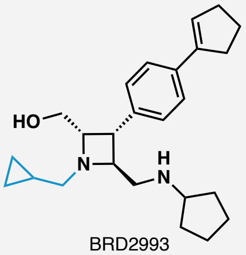

BRD2993
PubChem CID 60182795
Pf **EC_{50}** 0.84 μM

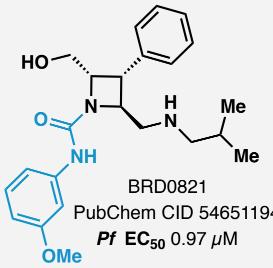

BRD0821
PubChem CID 54651194
Pf **EC_{50}** 0.97 μM

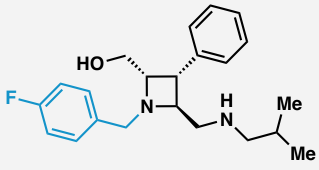

BRD1484
PubChem CID 60182696
Pf **EC_{50}** 0.41 μM

Azetidine amine antimalarial chemotype tolerates modifications at azetidine nitrogen. Selected examples of BRD8468 analogs exhibiting submicromolar *in vitro* EC_{50} against *P. falciparum* (Dd2 strain). All data were retrieved from PubChem (https://pubchem.ncbi.nlm.nih.gov/) using the CID compound identifiers shown above.

Materials

Reagents

RPMI Medium 1640 (without phenol red, Cat. # 11835-055), gentamicin (Cat. # 15710-072), sodium bicarbonate (Cat. # H9377), Hepes (Cat. # H4034), Glucose (Cat. # G5400), and sodium hydroxide (Cat. # S8045) are from Sigma and together with human serum (O^+, Interstate Bank) comprise the "complete" media used for routine maintenance of parasite cultures or alternatively together with Albumax II (Cat. # 11021-045, Invitrogen) comprises the "screening" media used for *in vitro* efficacy testing in 384-well format. Lysis buffer and detection components include EDTA (Cat. # E7889) and Triton X-100 (Cat. #T8787) from Sigma, Tris-HCl (Cat. # BP1756) from Fisher, Saponin (Cat. # AC41923) from Acros Organics and SYBR Green I (Cat. # S7567) from Invitrogen. Human serum whole blood (O+) is from Interstate Blood Bank. Giemsa stain is from Ricca Chemical Company, Cat. # 3250-4.

Parasites

Plasmodium falciparum strains Dd2L, Dd2-PI4K GFP over-expression line, and Dd2 dual-resistant line (to GNF156 and NITD609) were obtained from the laboratory of David Fidock at Columbia University. Both the D10 (Cat. # MRA-201 from MR4) and D10-ScDHODH expression line were obtained from the laboratory of Akhil Vaidya at Drexel University.

Methods

Instrumentation

Parasitized erythrocytes in screening media, and SYBR Green lysis buffer are dispensed using a MultiFlo from BioTek. Compound transfer is carried out by using the Labcyte ECHO Acoustic Liquid Handler. SYBR Green fluorescence is read on an Envision Multimode Reader, and parasitemia is determined using a light microscope (Primovert) with a Zeiss A-plan 100×/1.25 Oil Immersion objective.

Plasmodium falciparum propagation and parasitemia determination

Parasitized erythrocytes from each respective strain are passaged when parasitemia levels reach 8−10% via dilution into fresh culture medium to a final concentration of 1% parasitemia. Specifically, parasitized erythrocytes are transferred into prewarmed complete media containing RPMI 1640 (no phenol red) with L-glutamine, 0.05mg/mL gentamicin, 0.014 mg/mL hypoxanthine, 38.4 mM Hepes, 0.20% sodium bicarbonate, 0.20% Glucose, 3.4 nM NaOH, 5%

human serum and 1.25% albumax. Uninfected fresh erythrocytes are added to achieve a final concentration of 5% hematocrit for subsequent rounds of parasite invasion and proliferation. Cultures are maintained in 10 mL volumes in 25 cm^2 tissue culture flasks (Fisher Cat. # 10-126-39). The freshly diluted culture is gassed with a low oxygen mixture (96% nitrogen, 3% carbon dioxide, 1% oxygen) and incubated at 37˚C. Complete media is either added or exchanged every 1–2 days and parasitemia is monitored every 1–3 days, as needed, depending on the growth kinetics. The percent of parasitemia is estimated by obtaining a 1 μL blood smear. The smear is fixed onto the slide by placing in methanol for 30 seconds, stained in 10% Geimsa stain, and the percent of parasitized erythrocytes vs uninfected erythrocytes is determined by microscopy with a light microscope.

Plasmodium falciparum culture conditions

Routine maintenance of parasites

Using traditional *P. falciparum* protocols established by Trager and Jensen [1976], 10 mL cultures are maintained in 25 cm^2 flasks (Fisher Cat. # 10-126-39) at 5% hematocrit and parasitemia ranging between 1–8%. Once parasitemia reaches 8–10% cultures are diluted to 1% parasitemia. Maintenance requires daily media changes and fresh blood every two weeks. Cultures are gassed for approximately 30 seconds to 1 minute using a blood gas mixture to maintain a gas composition of 96% nitrogen, 3% carbon dioxide and 1% oxygen and incubated at 37°C.

Plasmodium falciparum cultured in 1536-format

A MultiFlo (BioTek) is used to dispense parasitized erythrocytes, uninfected erythrocytes and RPMI screening media into 1536-well plates (789092-A by Griener Bio-One) in a final volume of 8 μL containing 0.3% parasitemia and 2.5% hematocrit. The plates are incubated for 72 hours at 37˚C in a gas chamber in the presence of the low oxygen blood gas mixture.

Assay Protocol

In brief, cultures of the respective *P. falciparum* strains to be used for screening are prepared with screening media (complete media without human serum but supplemented with 0.5% Albumax II) and fresh erythrocytes. Compounds are transferred via the Labcyte ECHO Acoustic Liquid Handler into the assay plates. Parasitized erythrocytes and fresh erythrocytes are prepared with screening media and dispensed into the assay plates containing compound for a final parasitemia of 0.3% and 2.5% hematocrit. The assay plates are directly transferred and cultured in a gas chamber at 37˚C in the presence of the low oxygen blood gas. After 72 hours and daily gas exchanges, the assay plates are removed from the incubator and SYBR Green lysis buffer is added

to each well using the MultiFlo. Plates are incubated for an additional 24 hours at room temperature for fluorescence signal development. Fluorescence intensity is read on an Envision Multimode Reader.

Preparation of compound stocks and dilutions

Compounds are solubilized from powders in 100% DMSO at a final concentration of 10 mM and stored at room temperature by the Calibr Compound Management Group (CMG). Compound dose-response activity plates were prepared by adding 30 μL 10 mM stock solution to columns 1 and 13 in a 384-well compound plate. Compounds were transformed into 12-point , 1:3 dilutions by serially transferring 10 μL of the prior well to 20 μL DMSO.

Treatment of parasitized human erythrocytes with compound

Compounds are transferred directly into the 1536-well assay plates using the Labcyte ECHO Acoustic Liquid Handler, followed by the addition of a mixture of parasitized human erythrocytes and fresh uninfected human erythrocyte in screening media.

Plasmodium falciparum invasion of human erythrocytes

Based on the measured parasitemia levels, each culture is added to screening media to yield a final parasitemia of 0.3% and 2.5% hematocrit blood. The dispense step is performed with a MultiFlo and the assay plates are immediately covered using a custom metal lid and placed in a gas chamber purged with a low-oxygen gas mix and incubated at 37°C. The total assay volume is 8 μL.

Determination of Plasmodium falciparum proliferation by nucleic acid detection

After a 72-hour incubation (equivalent to 1–2 cycles during the blood stage) at 37°C under 95% humidity, a prepared mixture of the lysis buffer (5 mM EDTA, 1.6% Triton X-100, 20 mM Tris-HCl, 0.16% Saponin) in water and SYBR Green detection (0.1%) reagent is dispensed at 2 μL per well using the MultiFlo. Cultures are incubated for an additional 24 hours at 25°C before measuring fluorescence intensity using the Envision with a 505 Dichroic Bottom Mirror. Excitation and emission filters are 485 nm and 530 nm, respectively.

Data analysis

Data are normalized based on maximum fluorescence signal values for DMSO-treated wells (no inhibition by compound) and the minimum fluorescence signal values for wells containing the highest concentration of inhibitor control compounds, for example, atovaquone at a final concentration of 12.5 μM. Data are analyzed on a plate-by-plate basis and compared to reference compounds that are always included on every plate, typically artemisinin, mefloquine and

atovaquone. The half-maximal effective concentration (EC50) values are obtained using the GeneData curve fitting model. The standard logistic regression model is applied for curve fitting. The quality of the assay run is assessed by the performance of the reference compounds where the EC50 must be within 3-fold of the standard reference values for the assay plate to pass requisite data quality needs. The z-factor for this assay ranges from 0.5–0.8. Additionally, all compounds are typically assayed in duplicate (independent assay plates) and EC50 values ideally must not vary by more than two-fold between plates.

Table S12. ***In vitro*** **antimalarial potency and cytotoxicity of synthesized di- and tri-substituted azetidines (s.d. = standard deviation, n = number of biological replicates).**

**compound**	**EC50 (μM)**						**CC50 (μM)**	
	***Pf* Dd2L**			***Pf* D10attB**			**HEK293T**	
	average	s.d.	n	average	s.d.	n	average	n
BRD8468 (**72**)	0.465	0.094	3	0.382		2	>20.00	2
BRD6596 (**73**)	4.081	1.758	3	1.620		2	16.70	2
**75**	>12.50		3	11.746		2	>20.00	2
**76**	12.443	0.091	3	11.129		2	>20.00	2
**77**	11.633	1.493	3	10.521		2	>20.00	2
**78**	11.694	1.144	4	>12.50		3	>20.00	3
**79**	12.371	0.215	3	10.351		2	>20.00	2
**80**	0.173	0.044	4	0.269	0.125	3	>20.00	3
**81**	>12.50		3	>12.50		2	>20.00	2
**82**	1.771	0.629	3	2.251		2	11.01	2
**83**	8.390	0.627	3	8.916		2	>20.00	2

compound	EC50 (µM) *Pf* Dd2L			*Pf* Dd2-PI4K over-expr. line				*Pf* Dd2-mutant PfATP4/PfCARL				*Pf* D10attB			*Pf* D10 - *Sc*DHOD			CC50 (µM) HEK293T	
	average	s.d.	n	average	s.d.	n	*fold-shift (vs. Dd2L)*	average	s.d.	n	*fold-shift (vs. Dd2L)*	average	s.d.	n	average	n	*fold-shift (vs. D10)*	average	n
BRD8468 (**72**)	0.465	0.094	3	0.360		2	*0.77*	0.299		2	*0.64*	0.382		2	0.421	2	*1.10*	>20	2
BRD6596 (**73**)	4.081	1.758	3	3.419		2	*0.84*	3.987		2	*0.98*	1.620		2	1.526	2	*0.94*	16.70	2
**80**	0.173	0.044	4	0.235	0.094	3	*1.36*	0.280	0.063	3	*1.62*	0.269	0.125	3	0.187	2	*0.70*	>20	3
KDU691	0.063		2	0.608		2	***9.68***	0.044		2	*0.70*	0.050		2	0.016	1	*0.33*	>20	2
GNF156	0.008	0.004	3	0.009		2	*1.08*	4.122		2	***497.7***	0.009		2	0.005	1	*0.53*	>20	2
ELQ300	0.017		2	0.033		2	*1.94*	0.027		2	*1.60*	0.019		2	>12.5	1	***>641***	>20	2
atovaquone	0.001	0.001	3	0.004		2	*3.34*	0.003		2	*2.68*	0.002		2	1.357	1	***696.54***	>20	2
mefloquine	0.004		2	0.003		2	*0.79*	0.003		2	*0.83*	0.008		2	0.005	1	*0.65*	4.81	2
NITD609	0.00021		2	0.00021		2	*1.00*	0.001		2	*3.98*	0.000		2	0.000	1	*1.08*	>20	2

Table S13. *In vitro* antimalarial potency and cytotoxicity of selected azetidines and reference compounds. Compound 80 remains equipotent against *Pf* drug-resistant strains (s.d. = standard deviation, n = number of biological

NMR Spectra

Compound B1 ^{1}H NMR

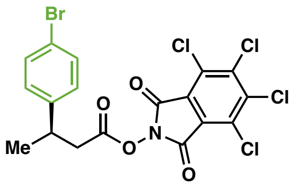

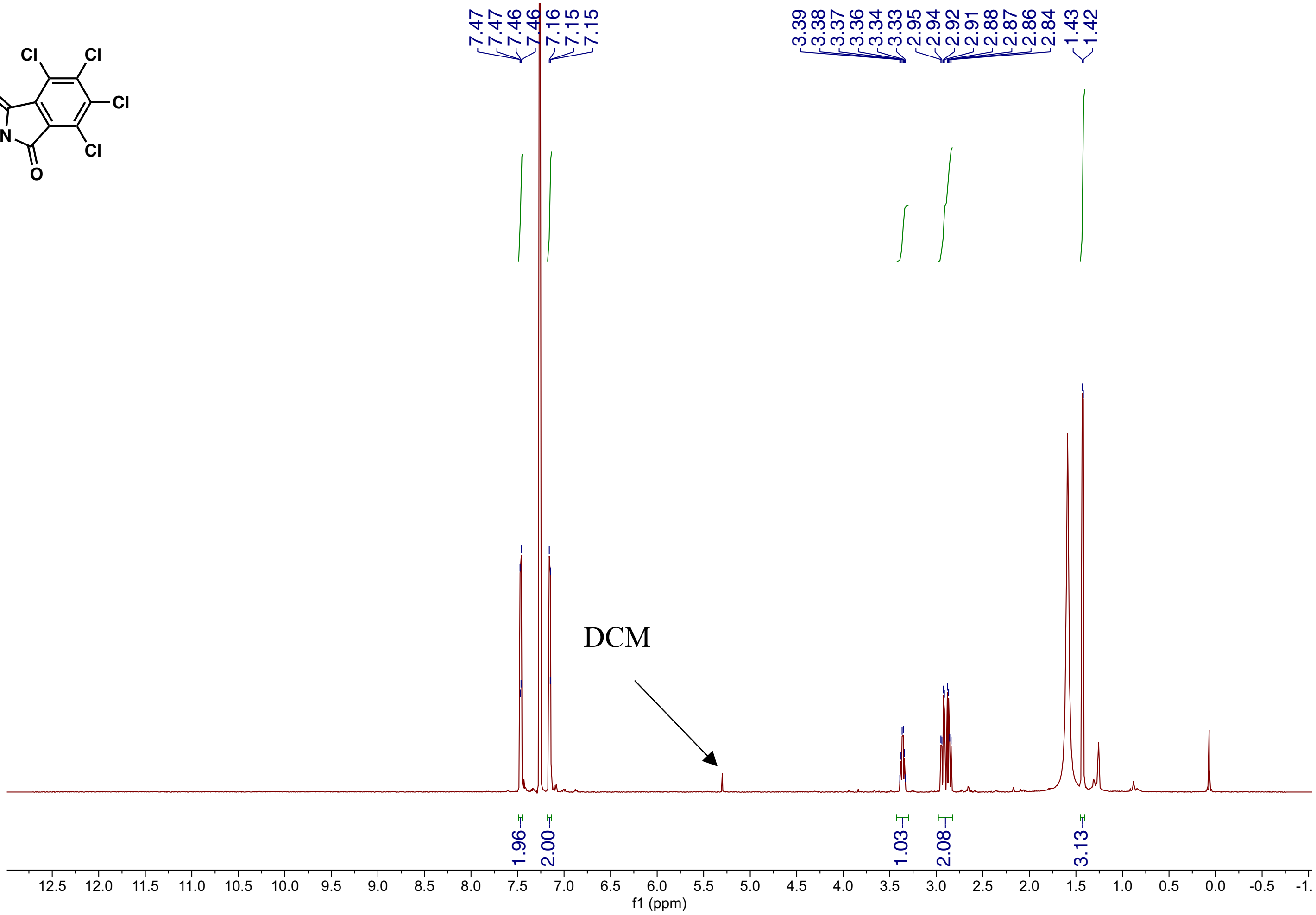

Compound B1 ^{13}C NMR

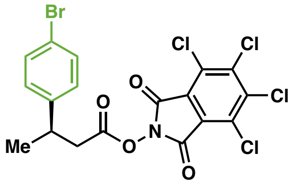

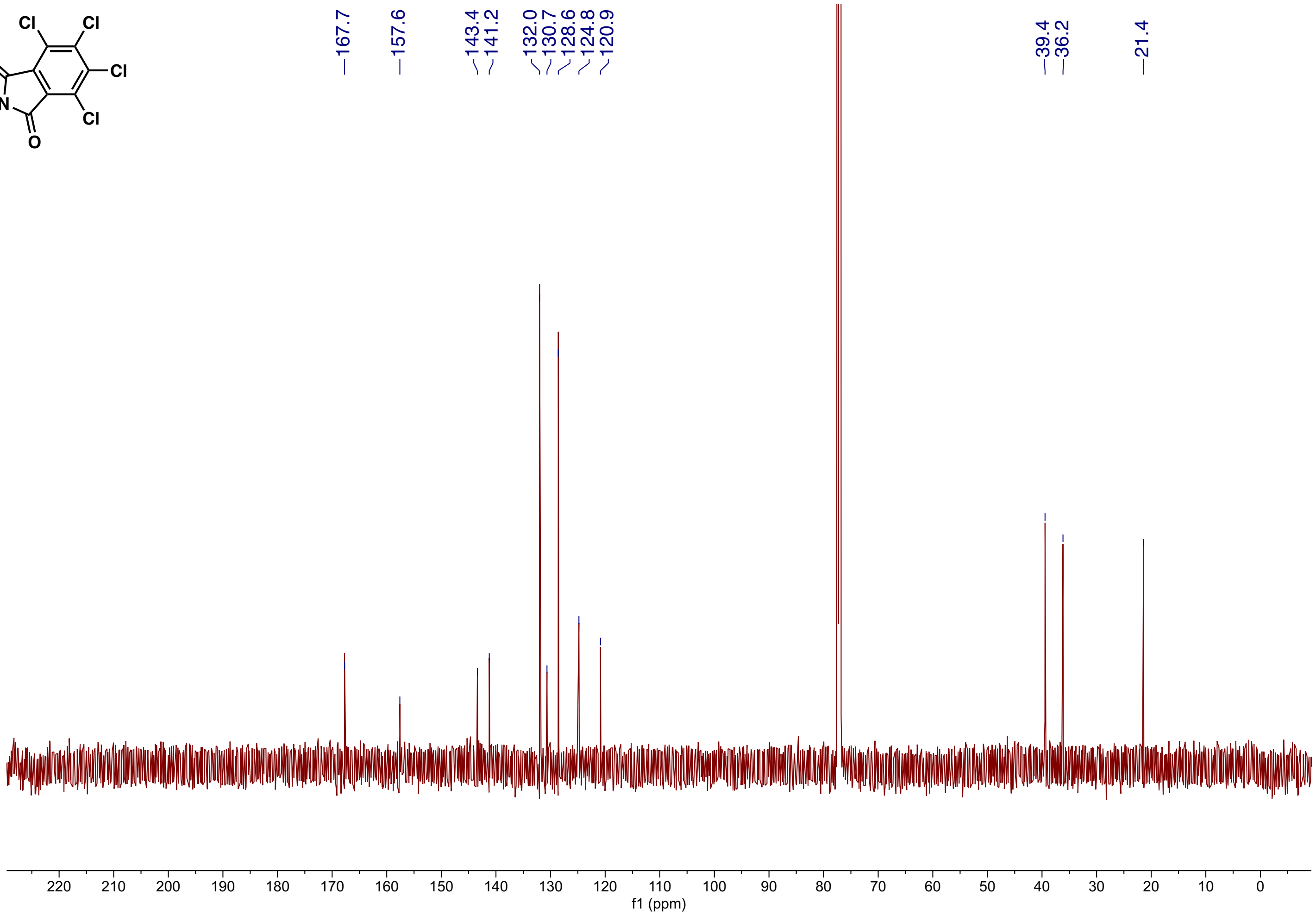

Compound B2 ^{1}H NMR

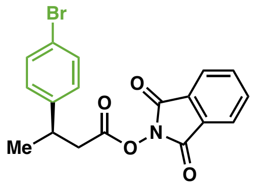

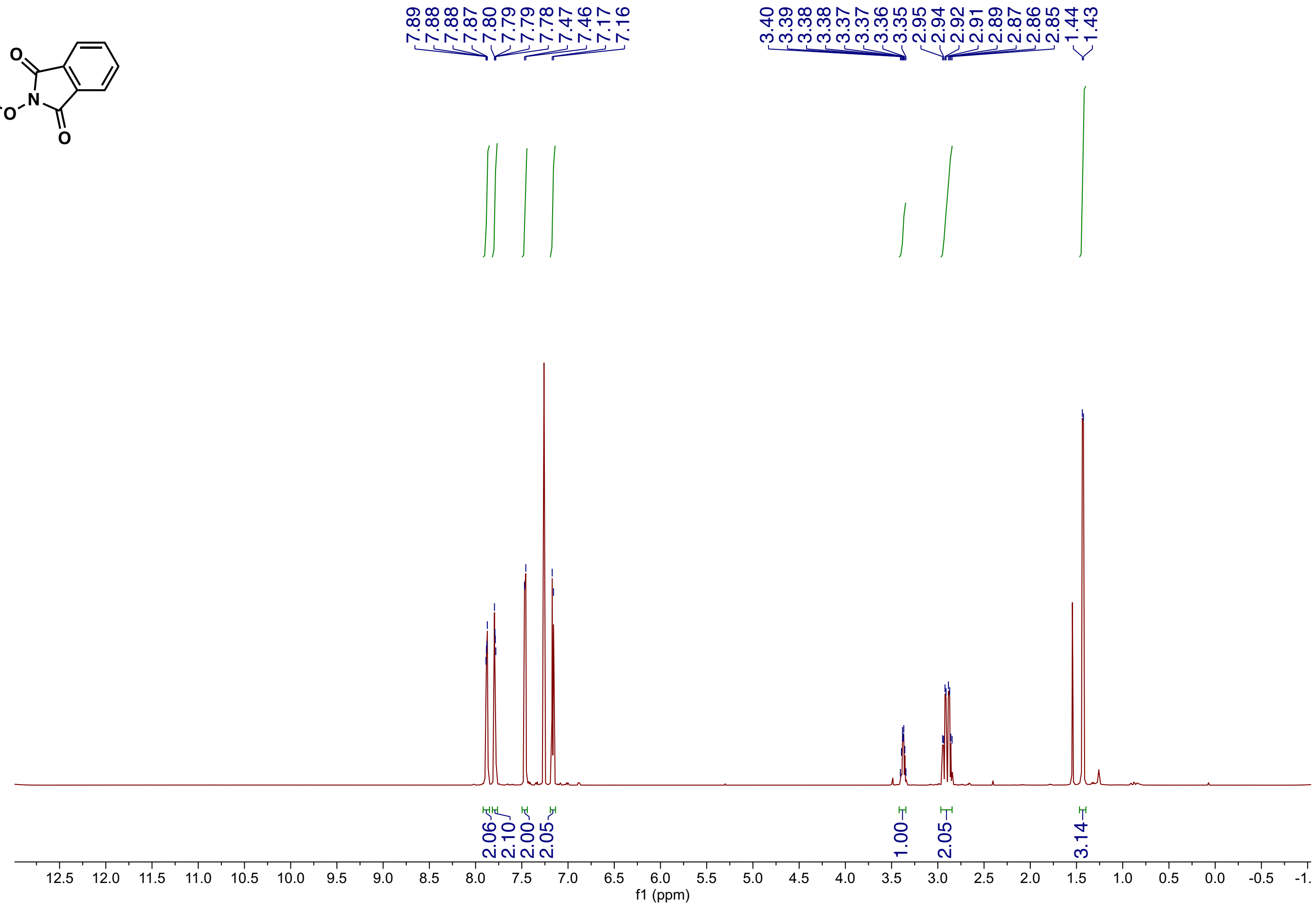

Compound B2 13C NMR

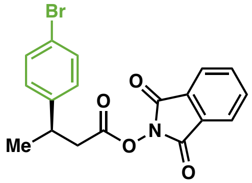

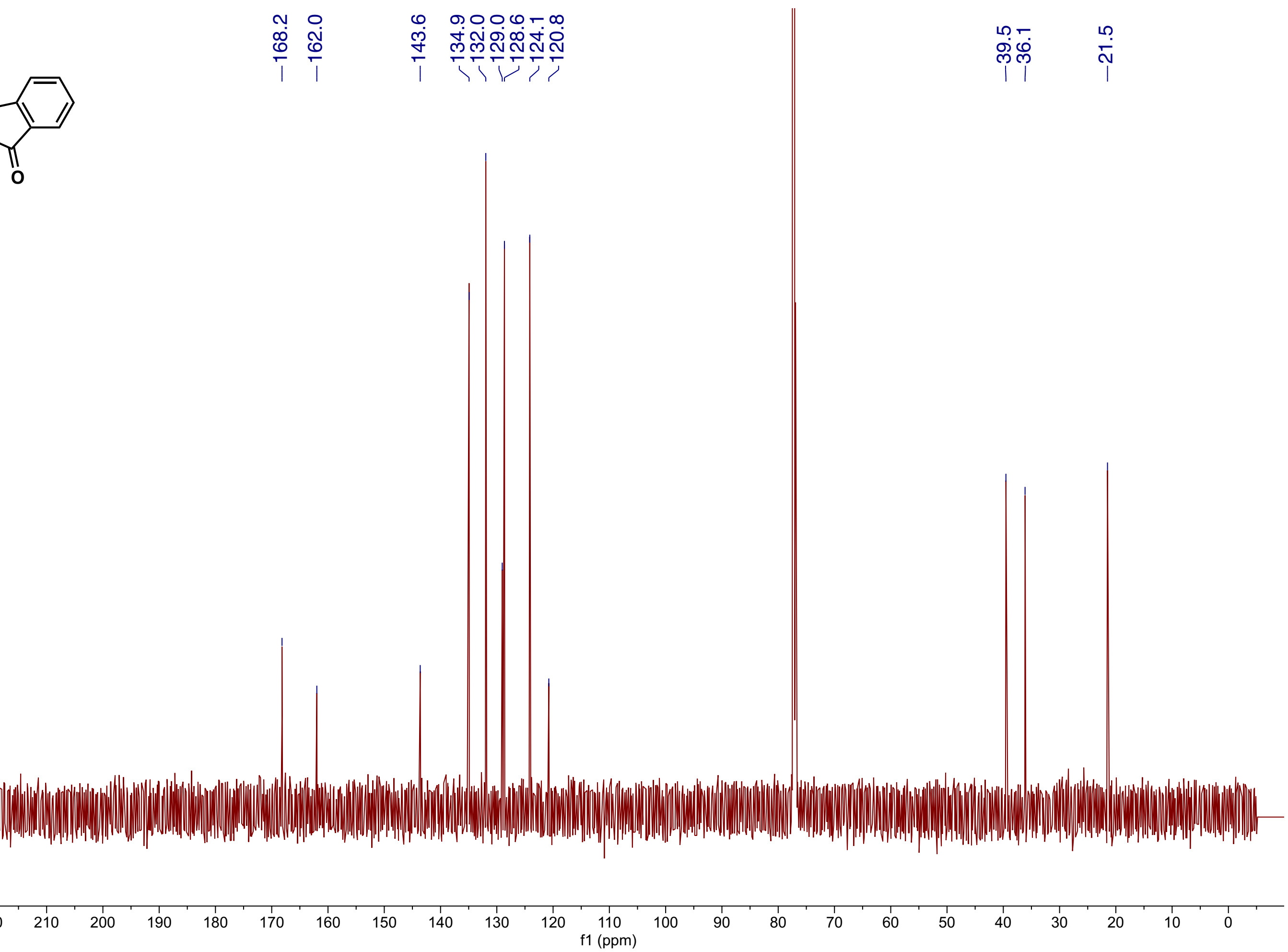

Compound B3 ¹H NMR

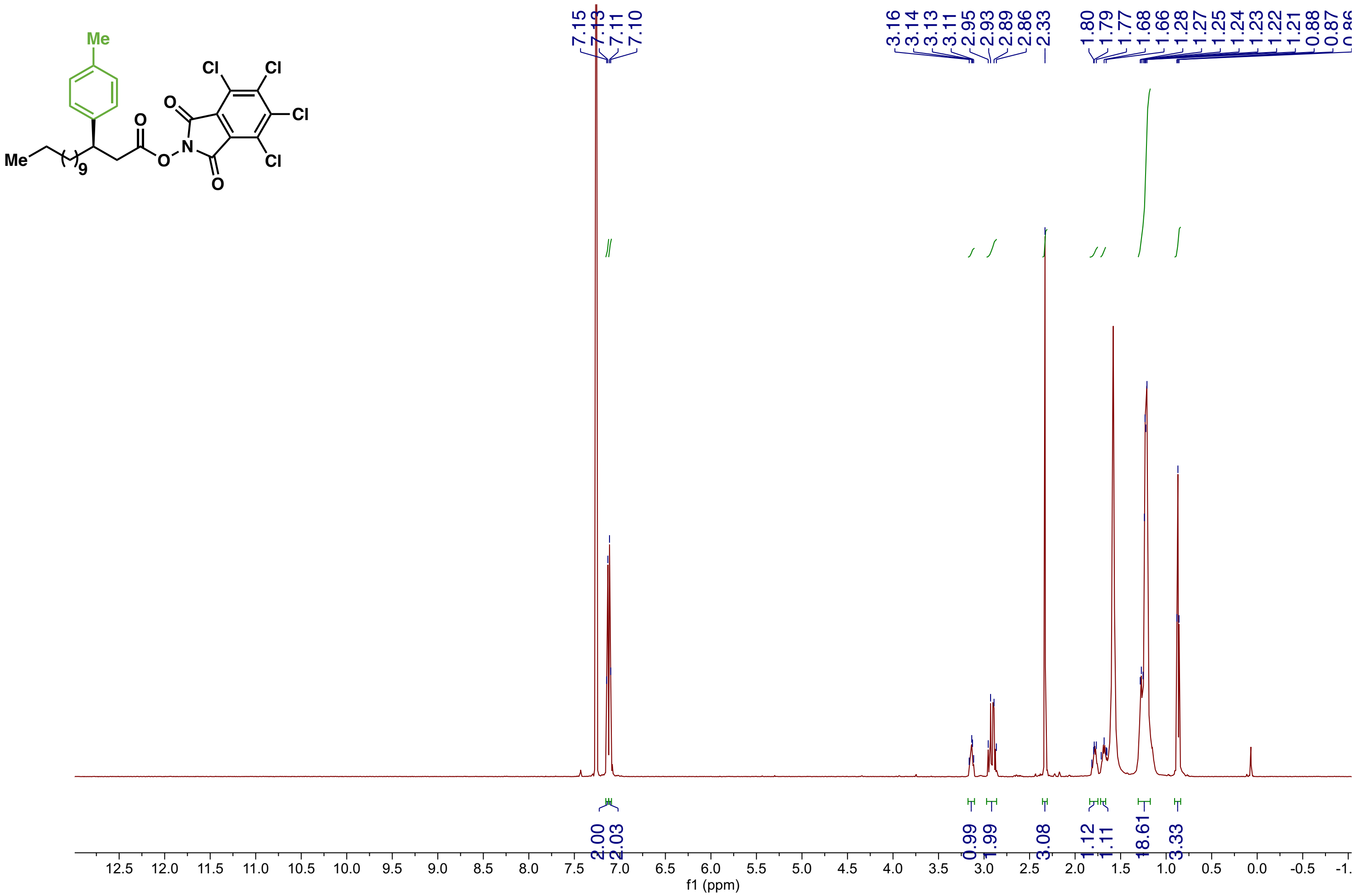

Compound B3 ^{13}C NMR

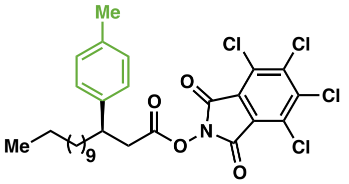

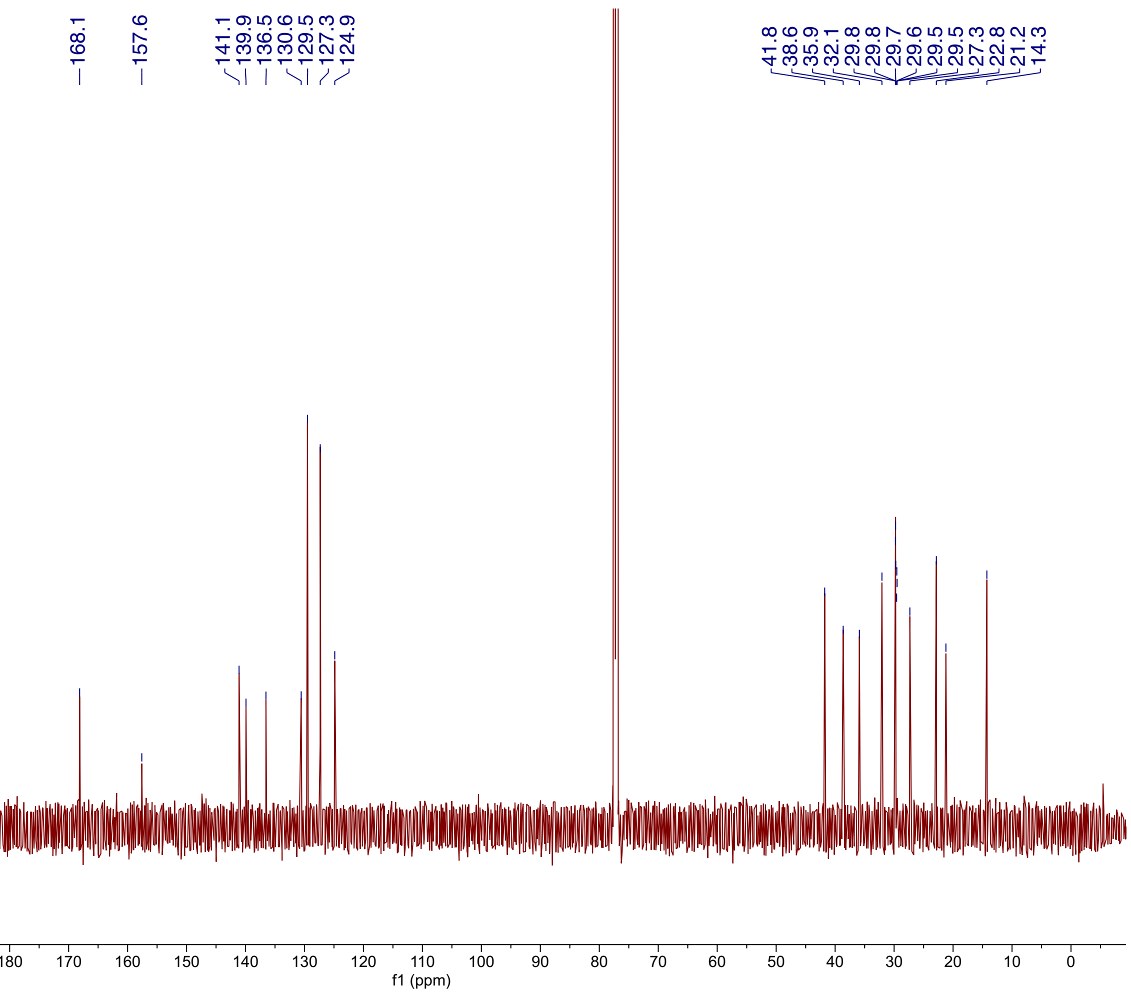

Compound B4 ^{1}H NMR

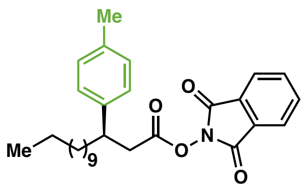

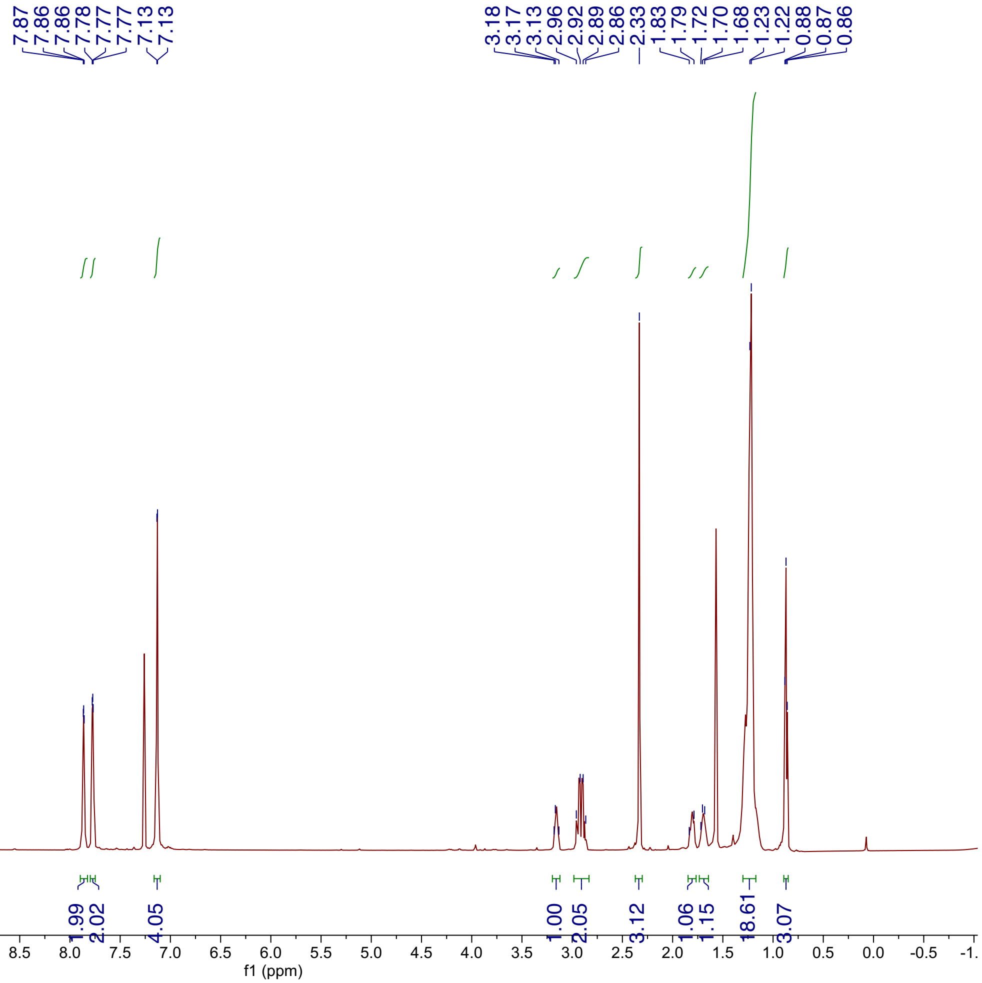

Compound B4 ^{13}C NMR

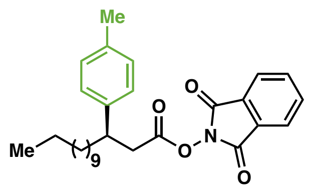

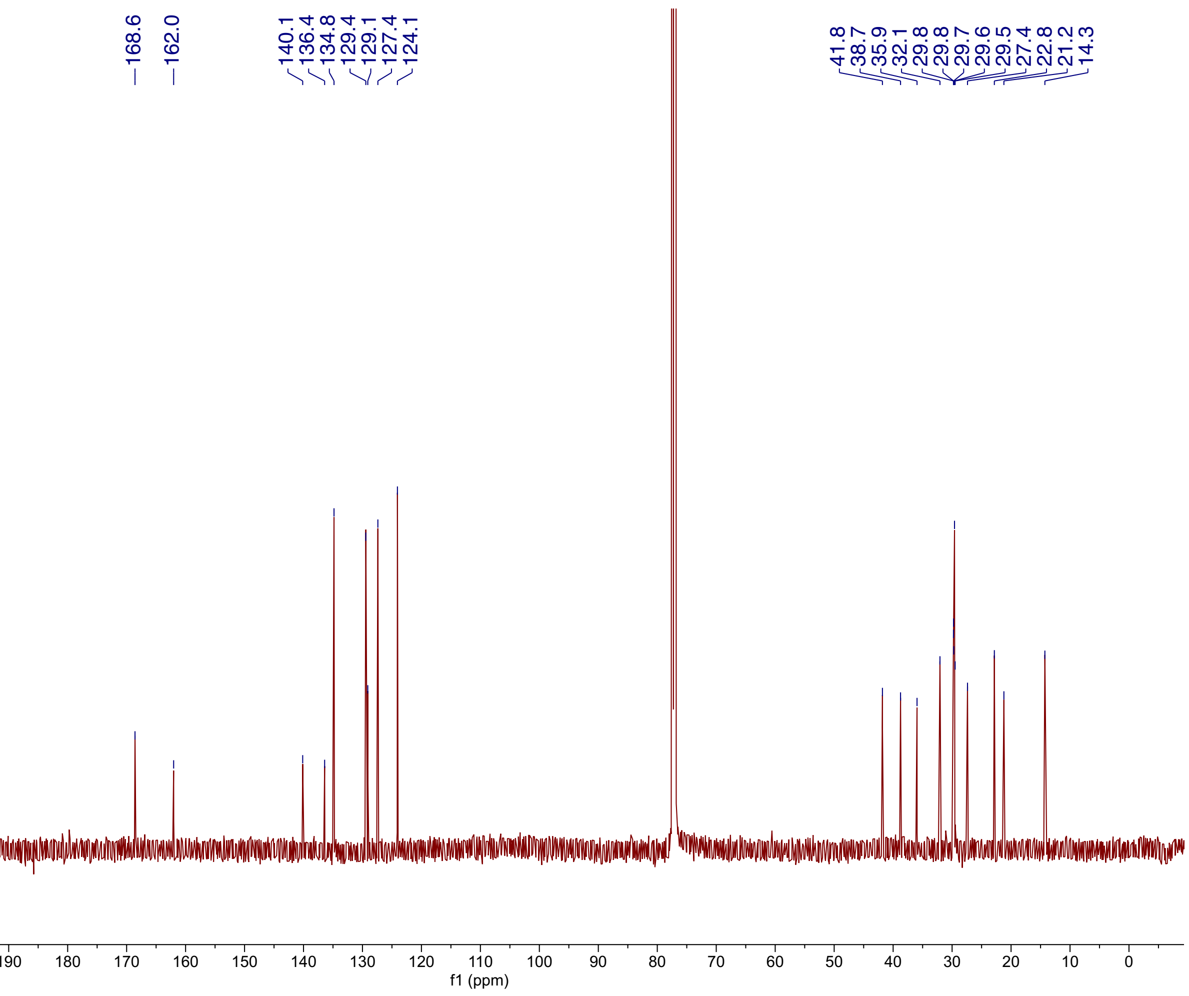

Compound B5 ^{1}H NMR

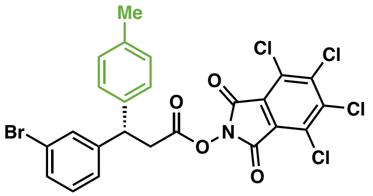

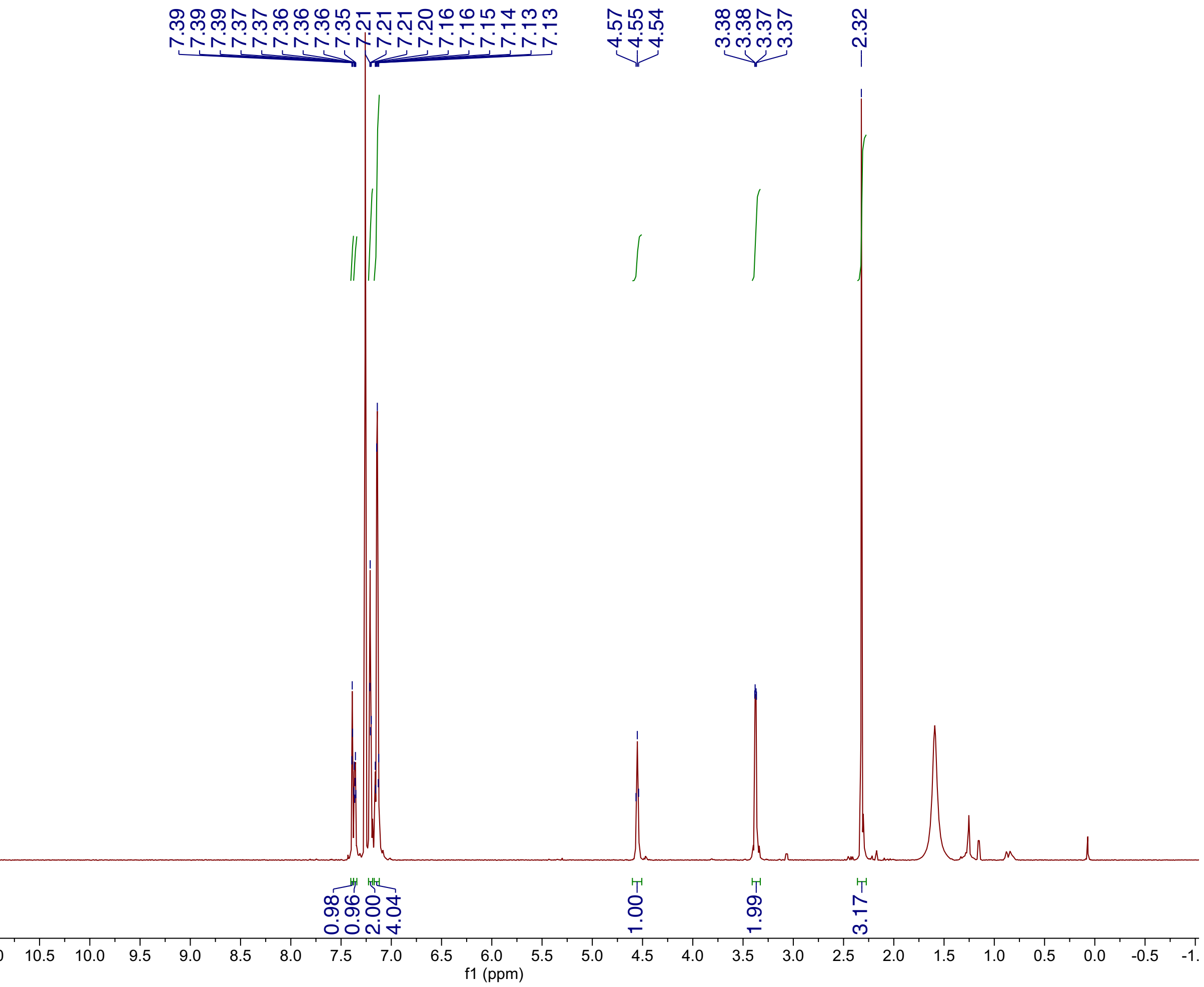

Compound B5 ^{13}C NMR

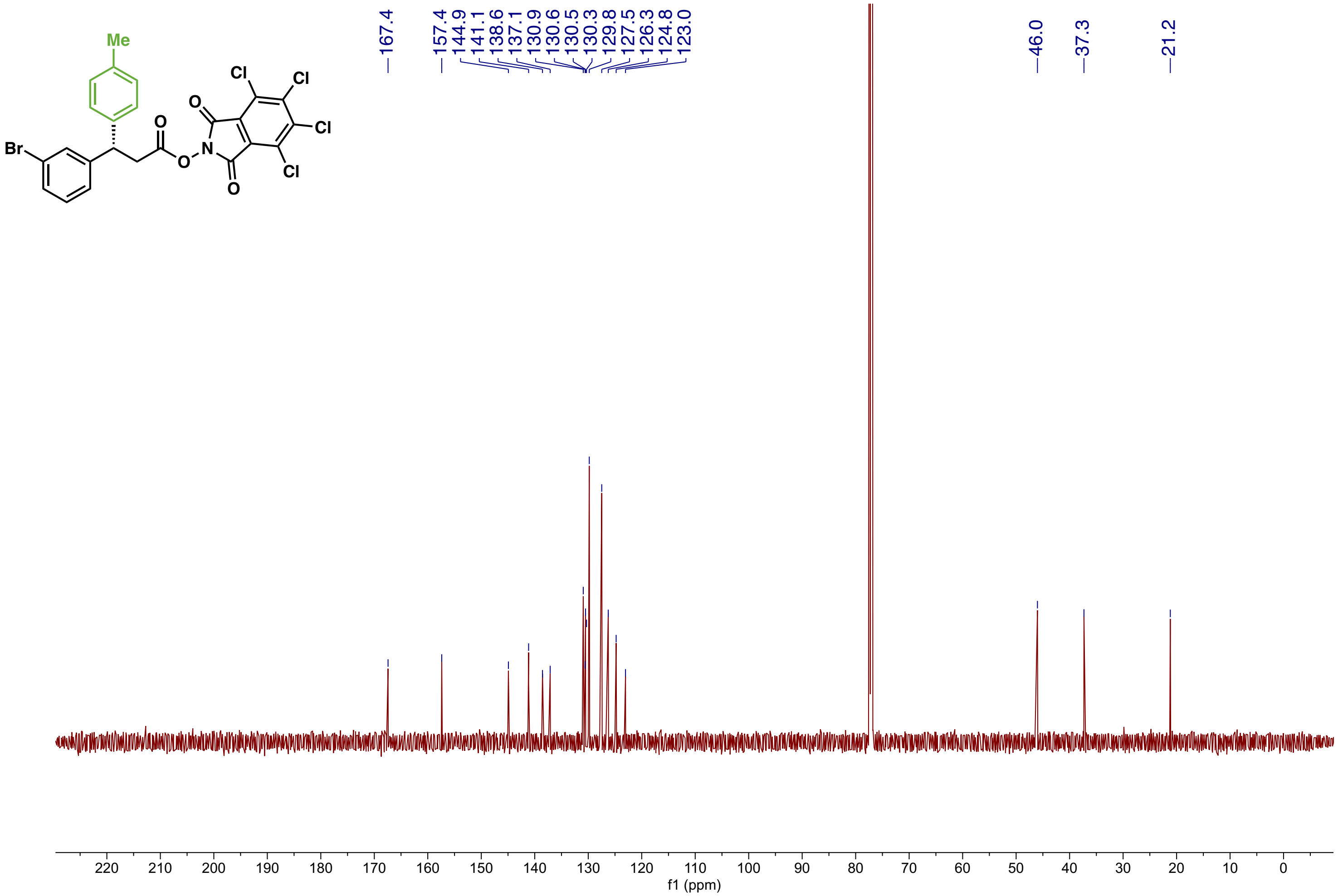

Compound B6 ^{1}H NMR

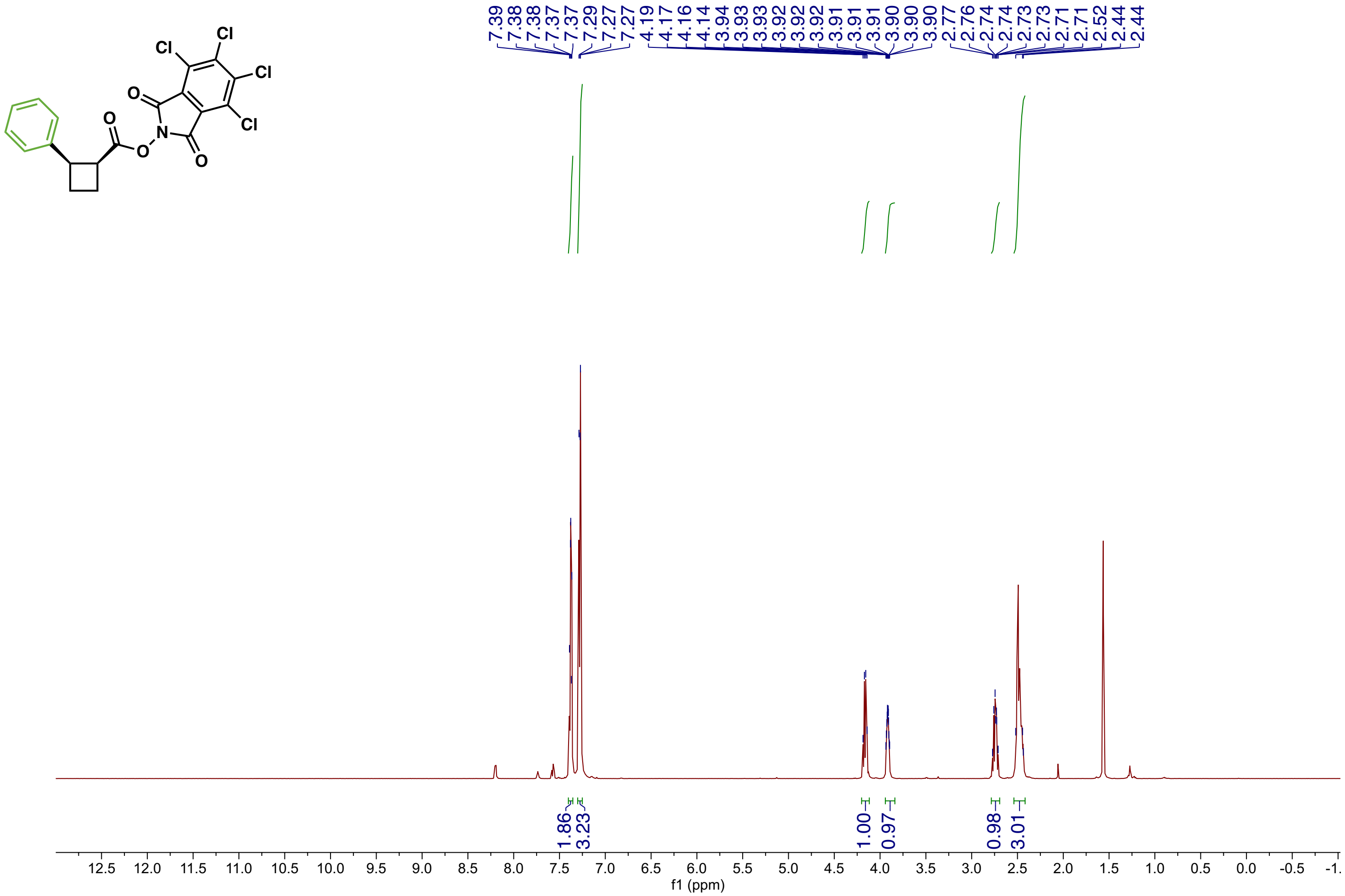

Compound B6 ^{13}C NMR

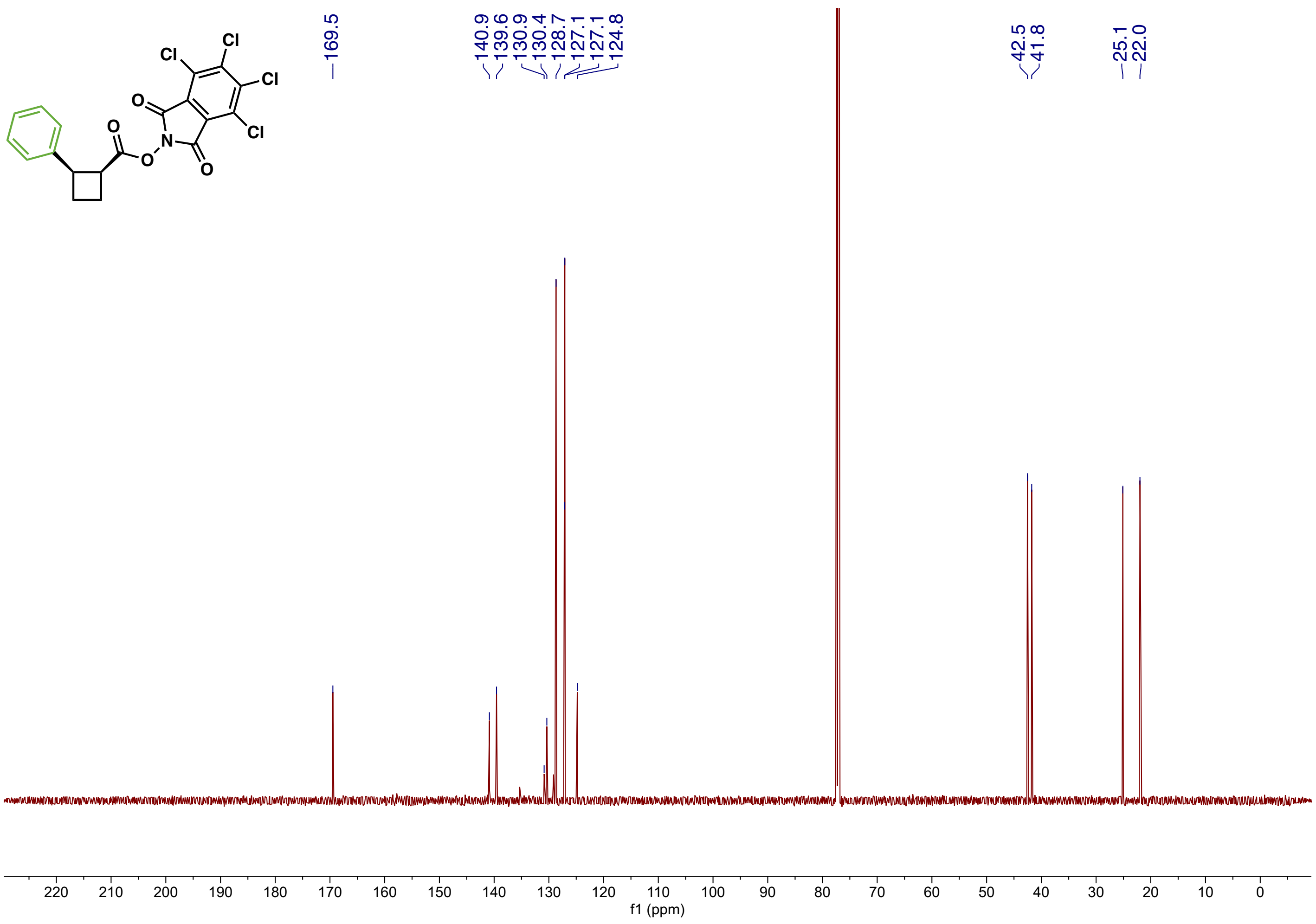

Compound B7 ^{1}H NMR

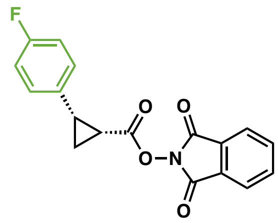

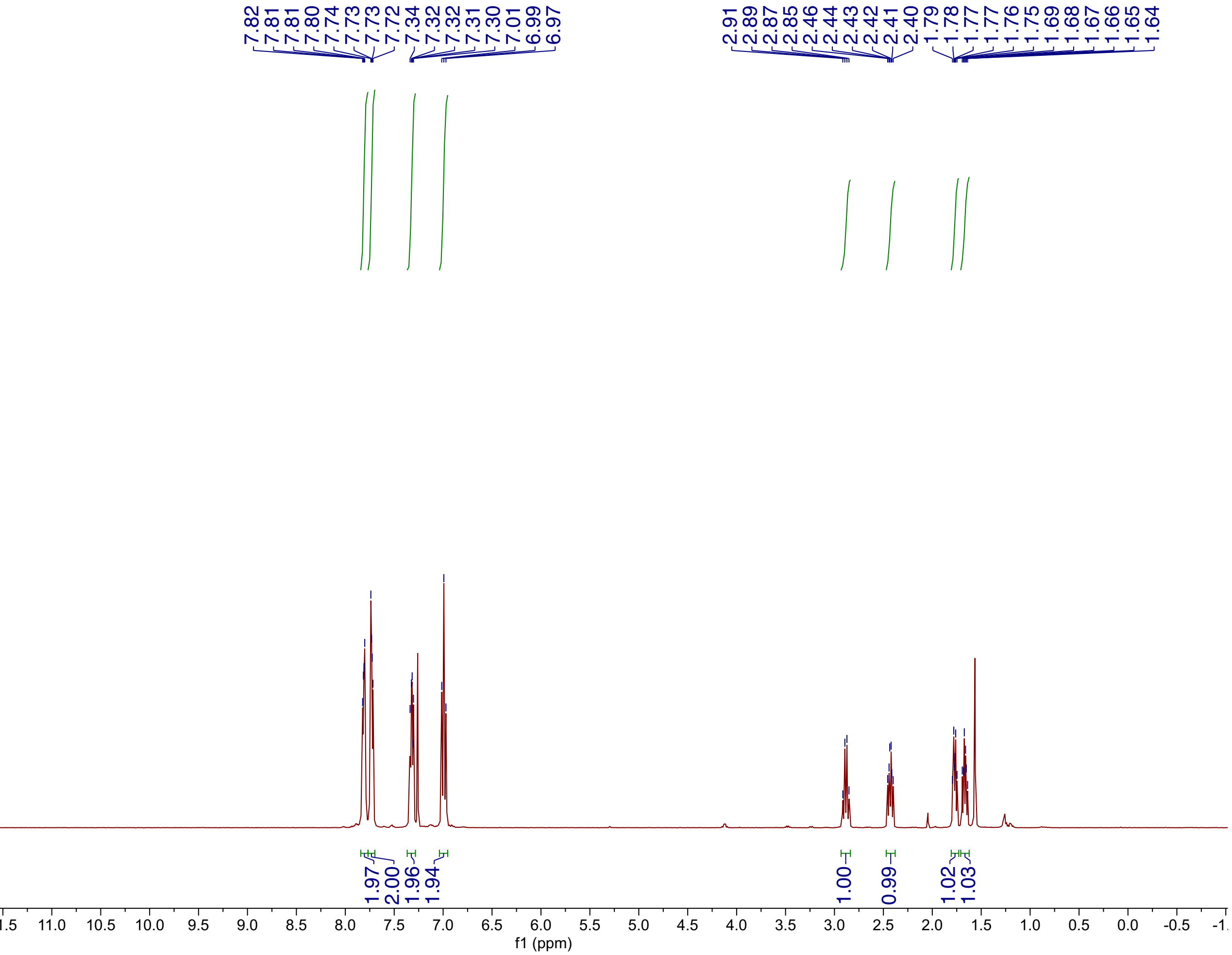

Compound B7 ^{19}F NMR

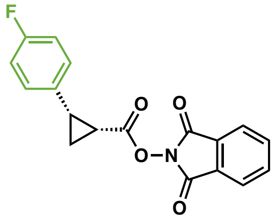

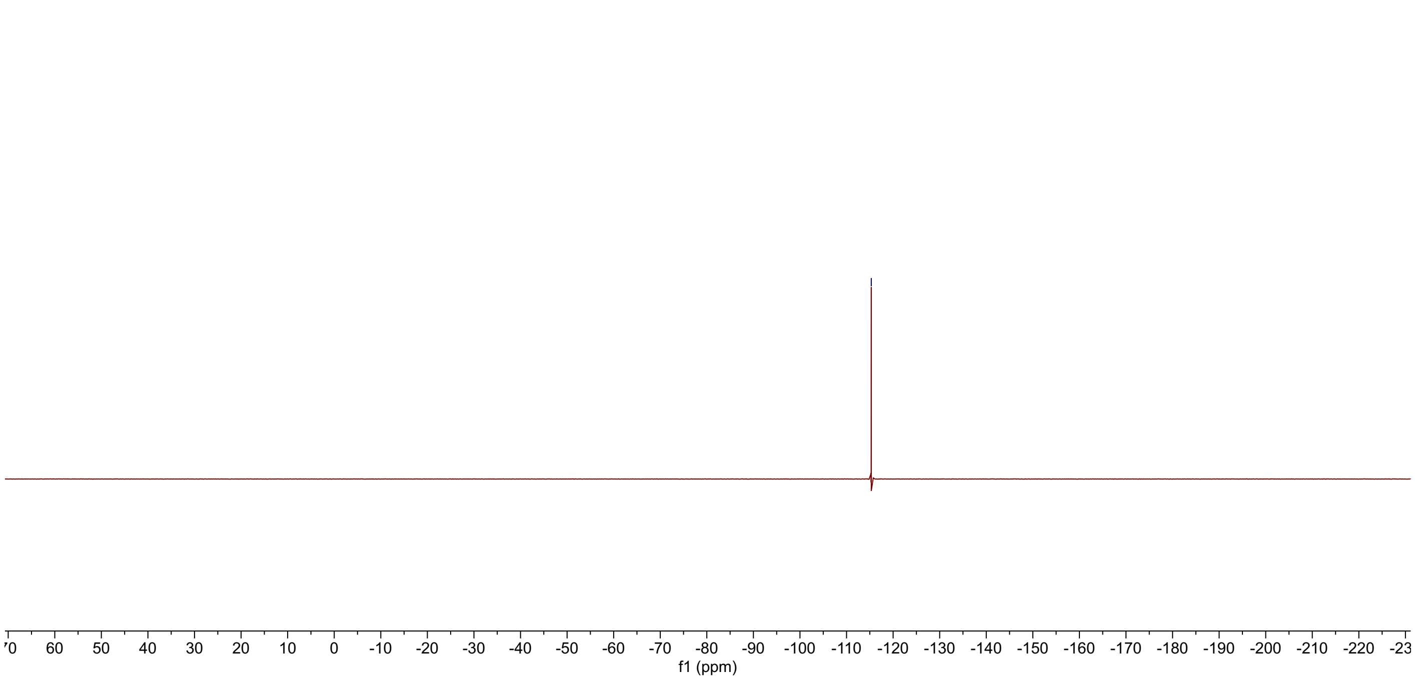

Compound B7 ^{13}C NMR

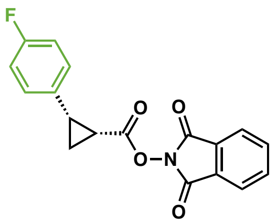

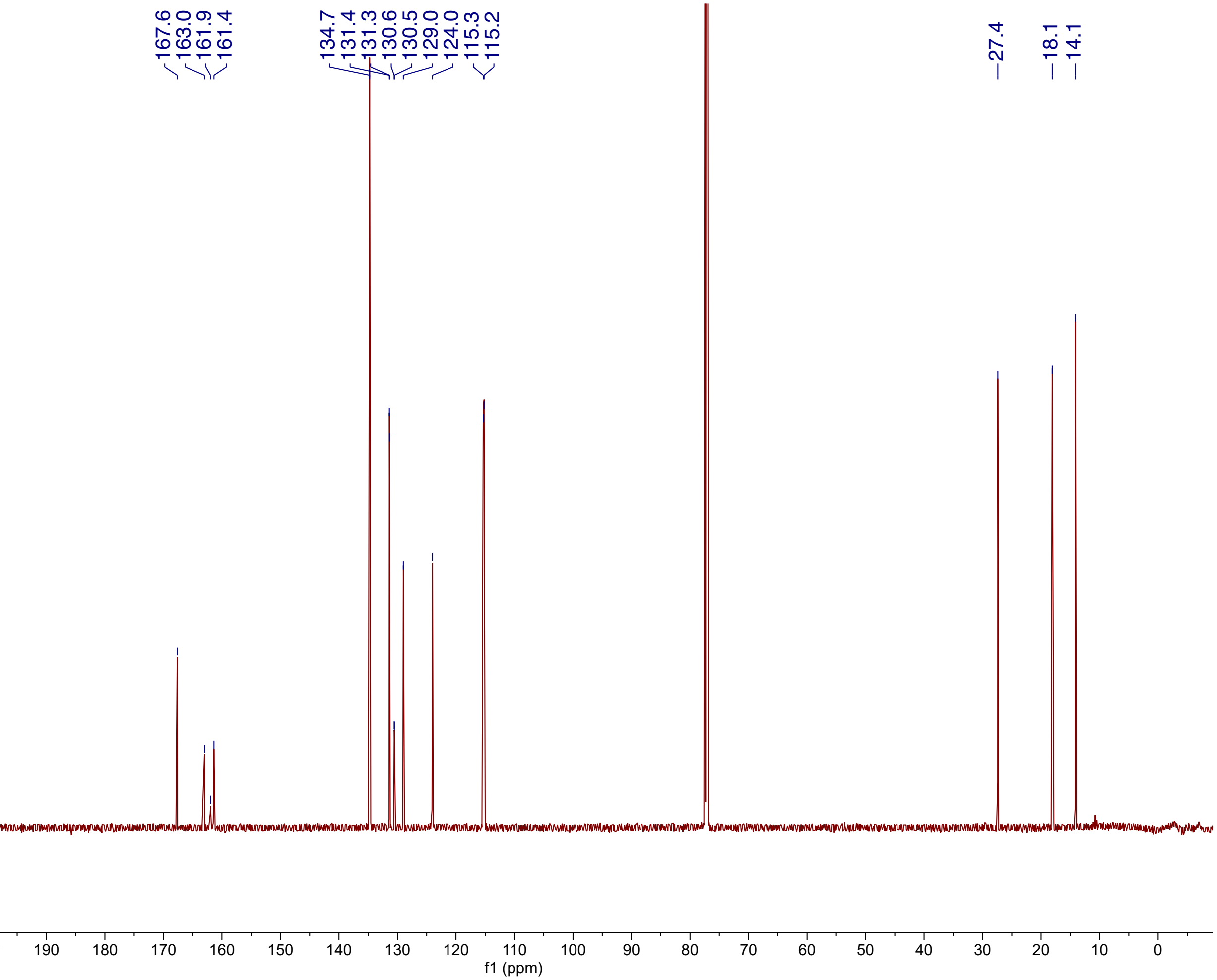

Compound 14 ^{1}H NMR

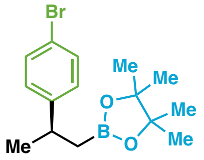

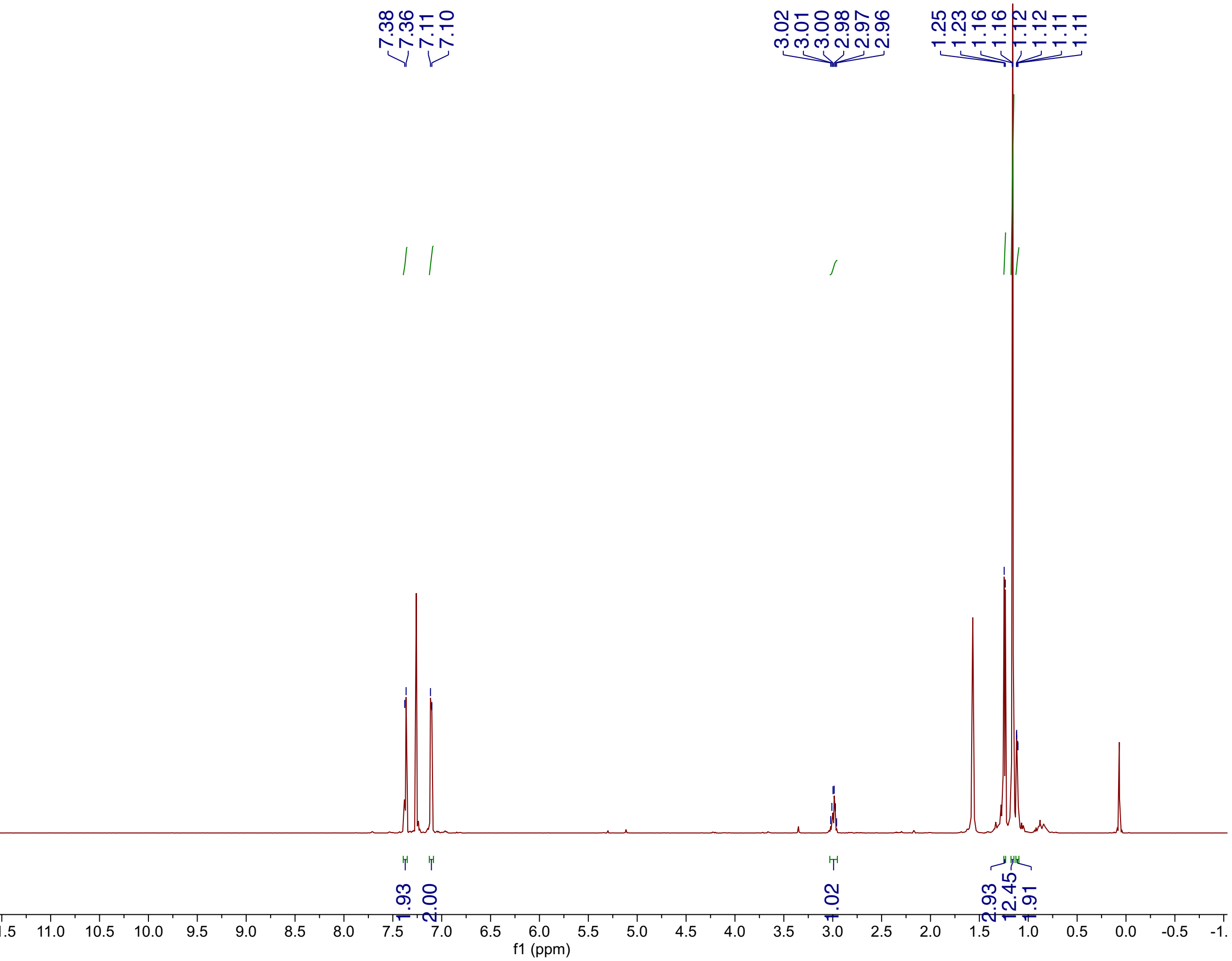

Compound 14 ^{13}C NMR

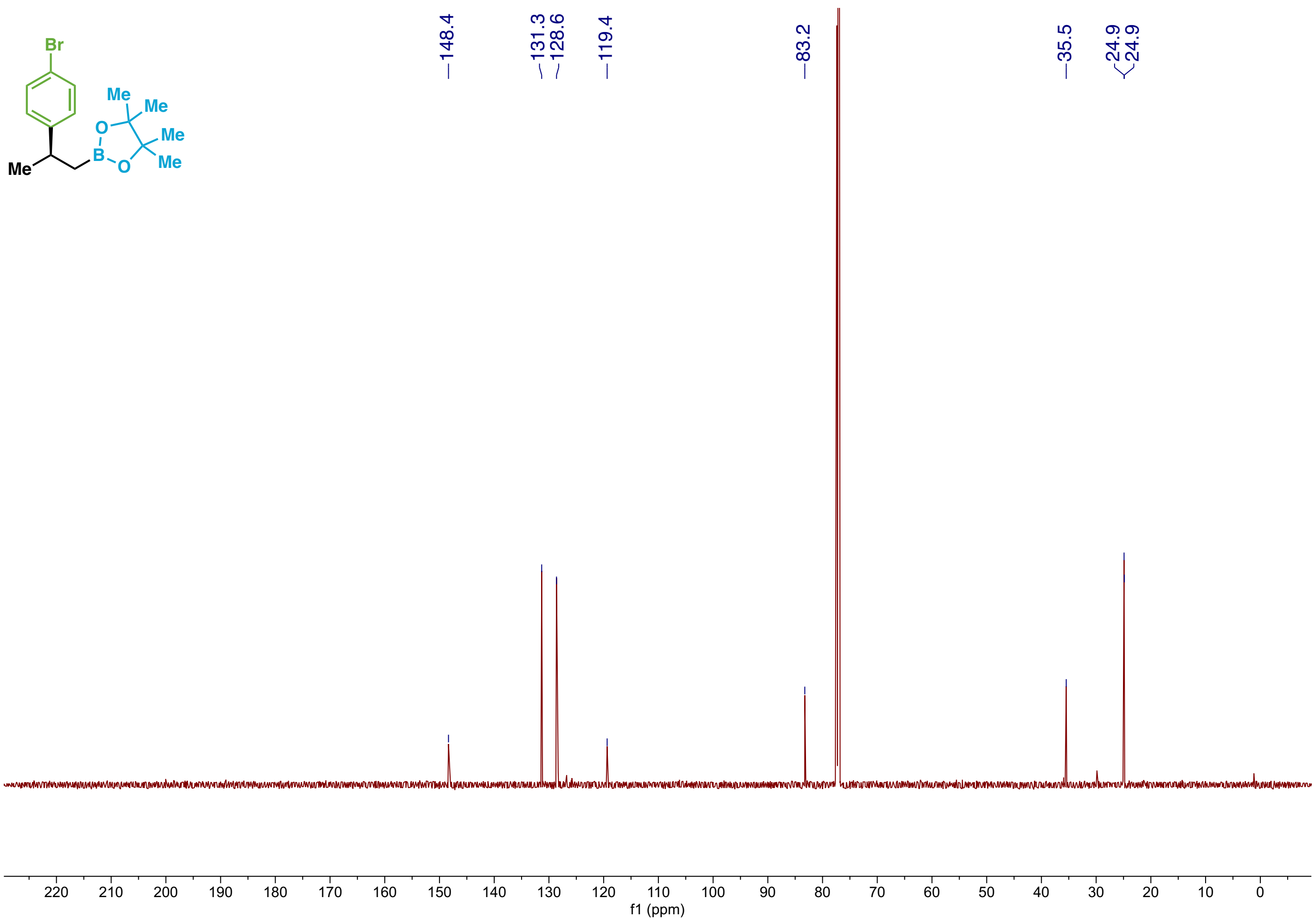

Compound 15 ^{1}H NMR

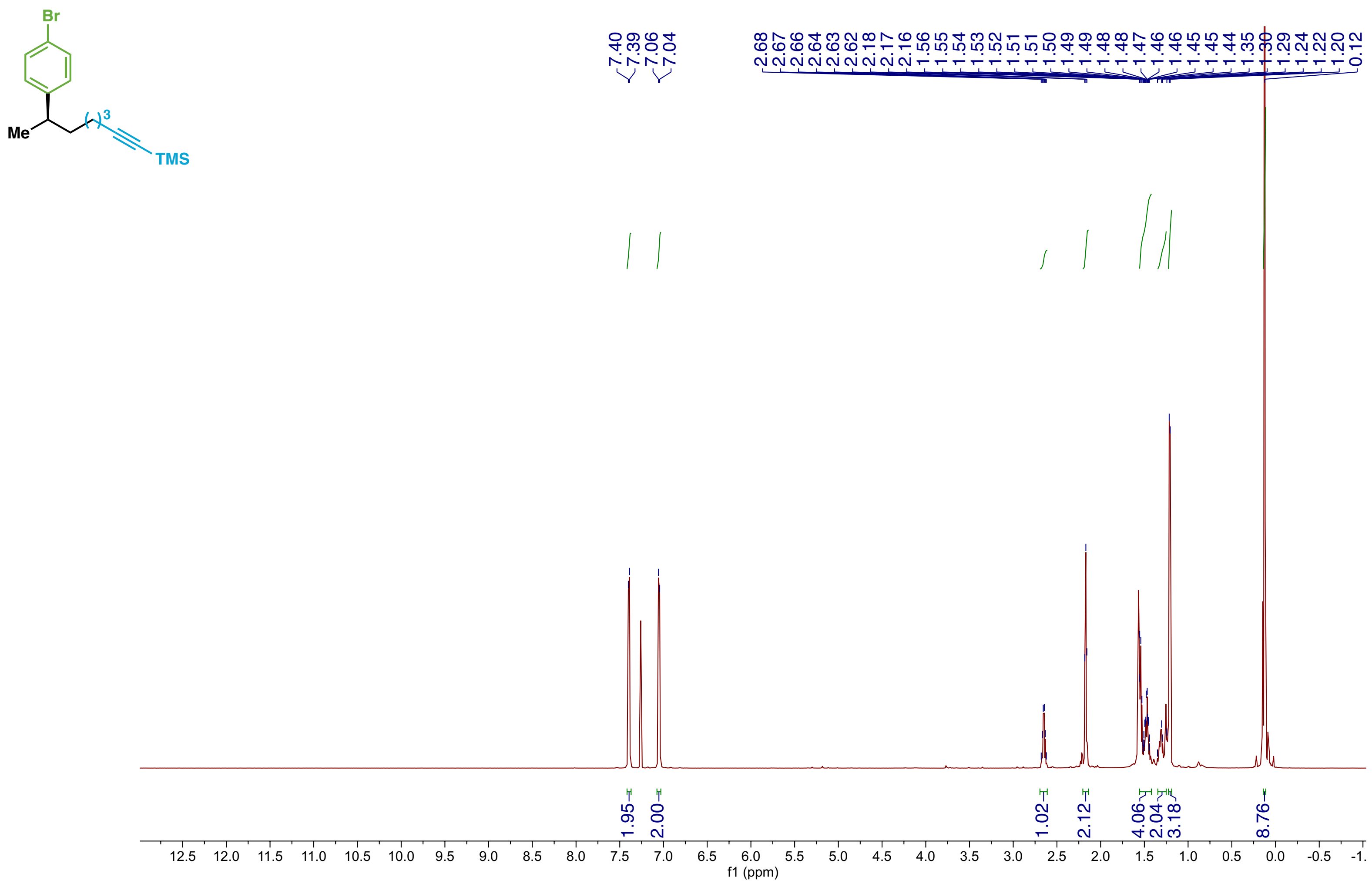

Compound 15 ^{13}C NMR

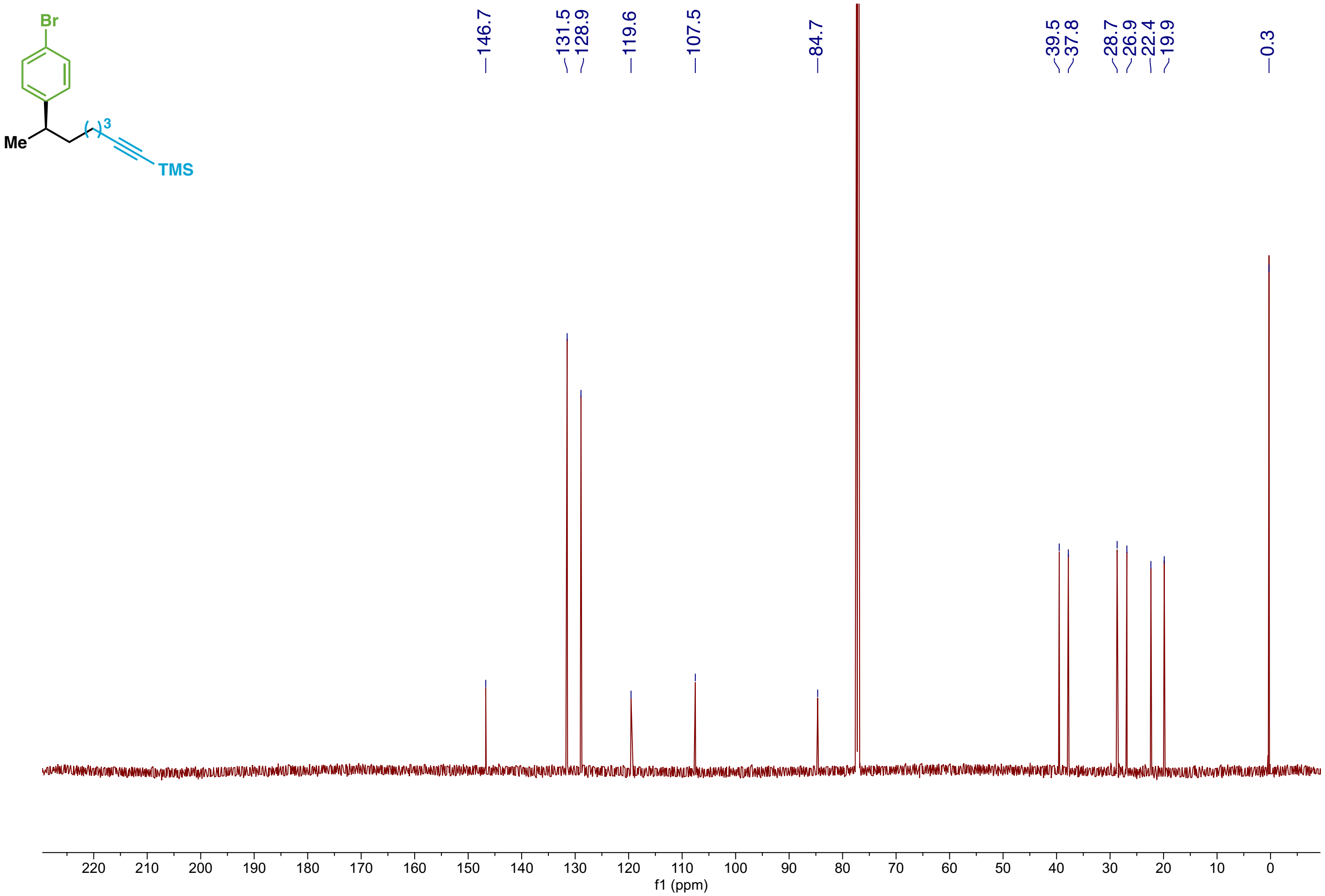

Compound 16 ^{1}H NMR

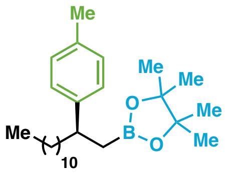

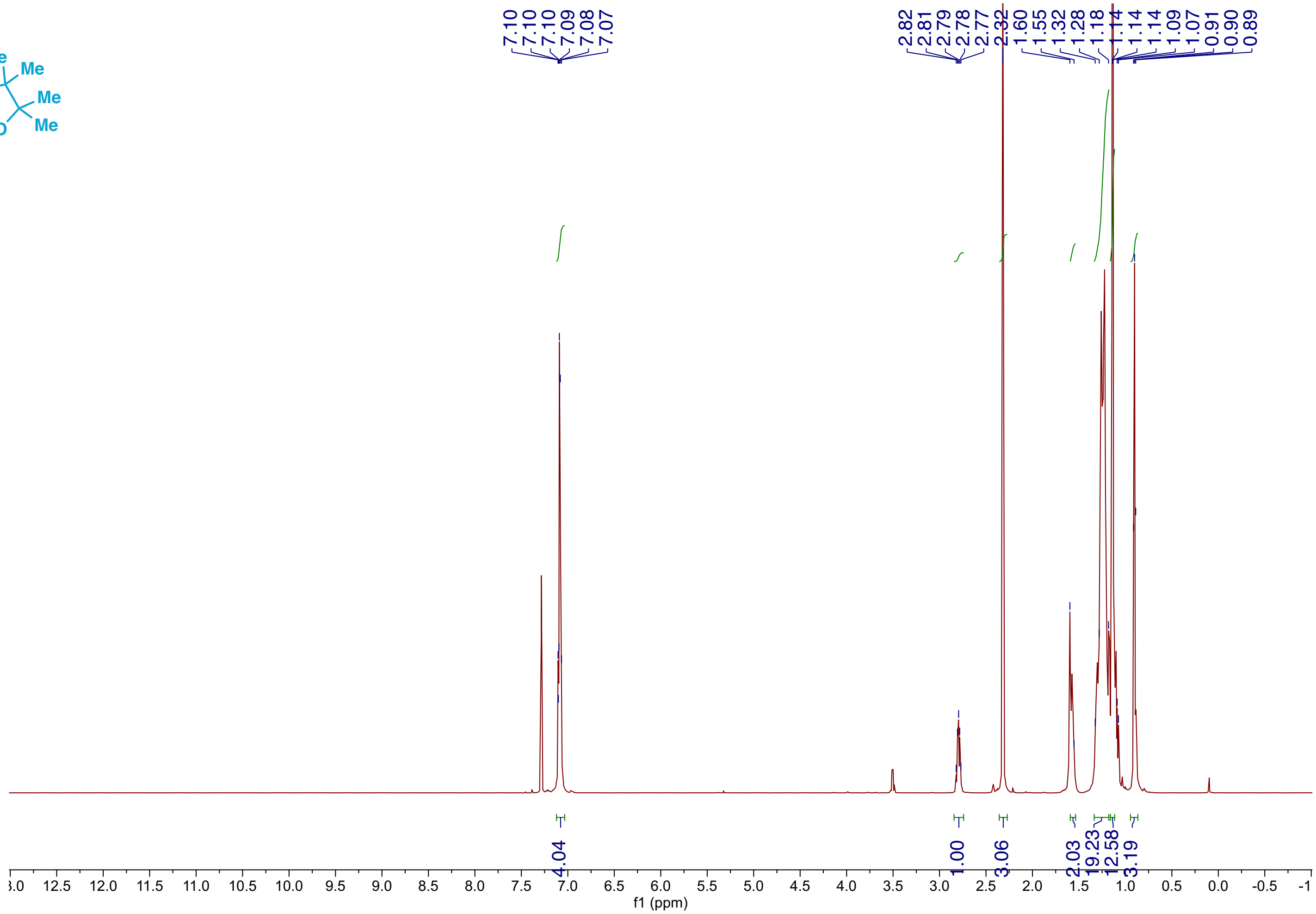

Compound 16 ^{13}C NMR

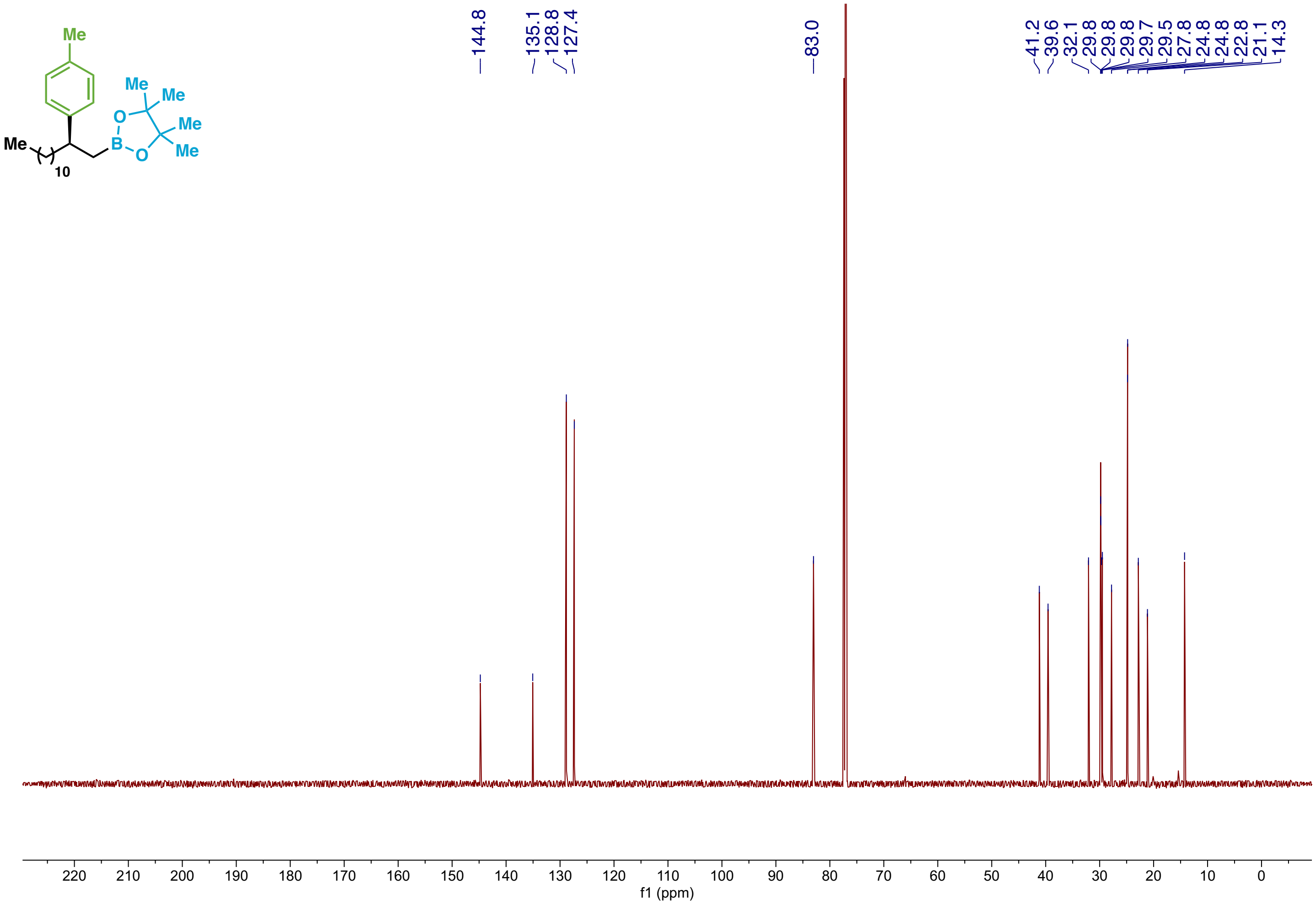

Compound 17 ^{1}H NMR

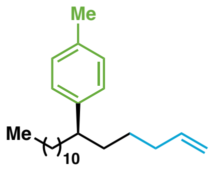

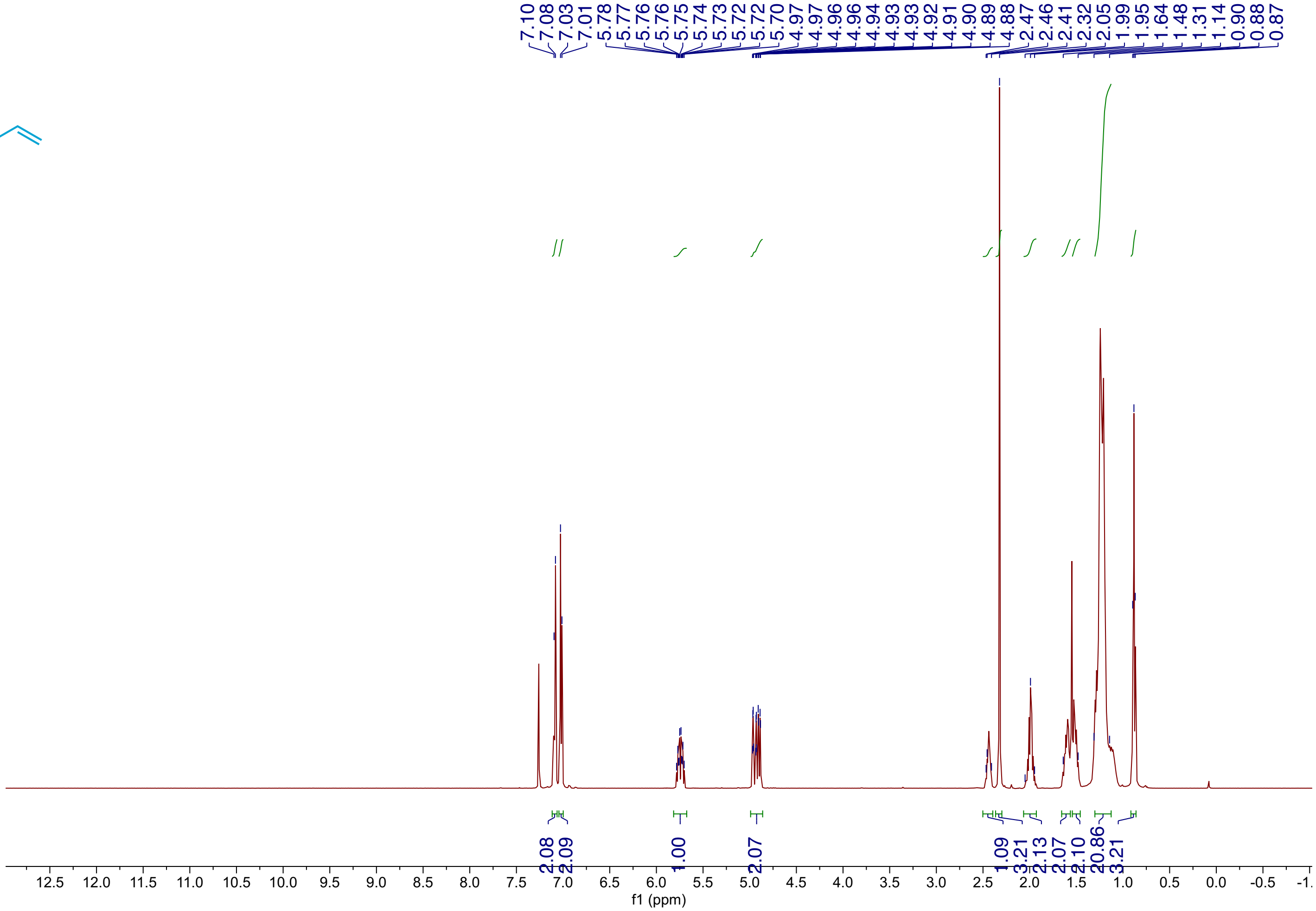

Compound 17 ^{13}C NMR

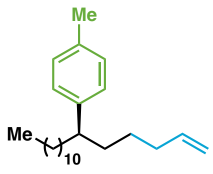

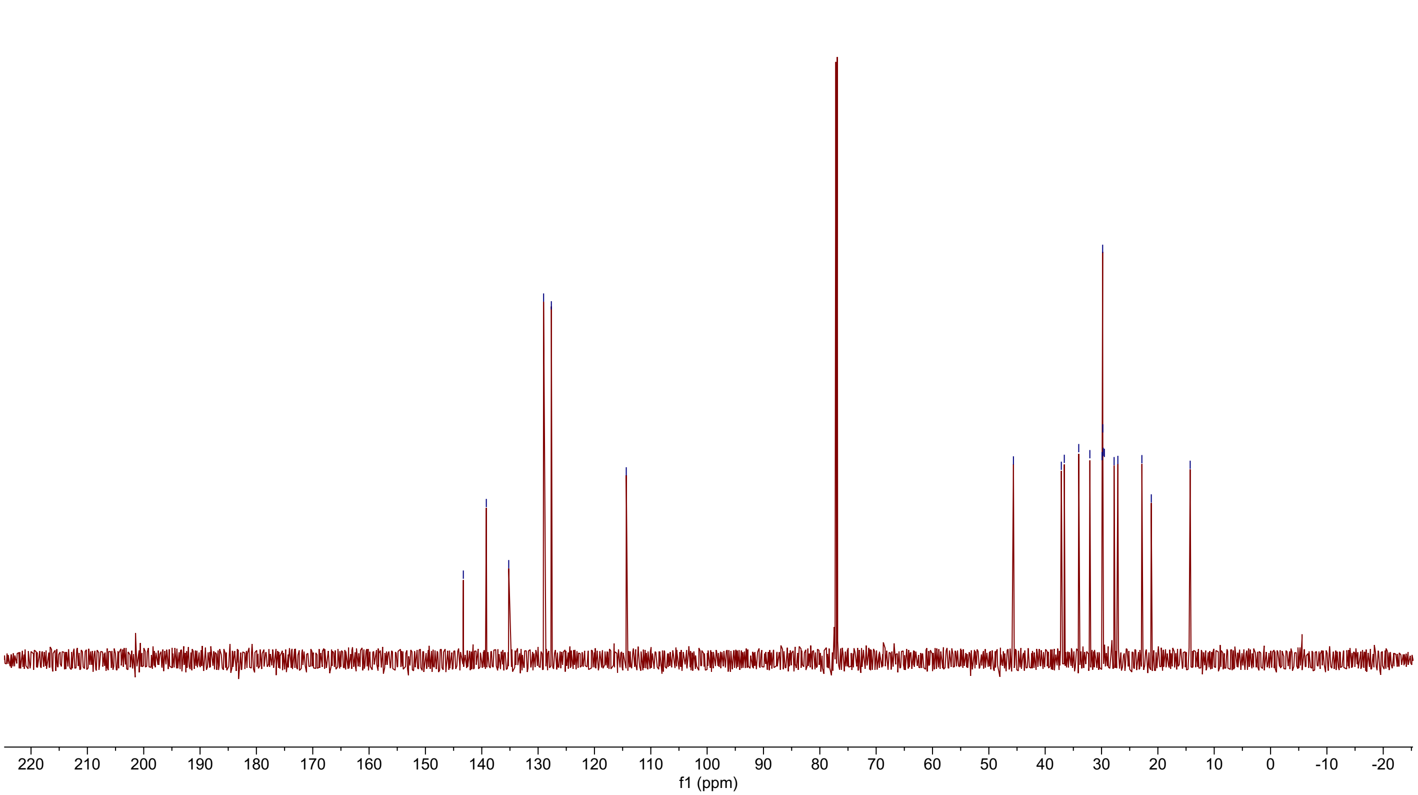

Compound 18 ^{1}H NMR

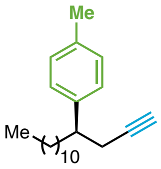

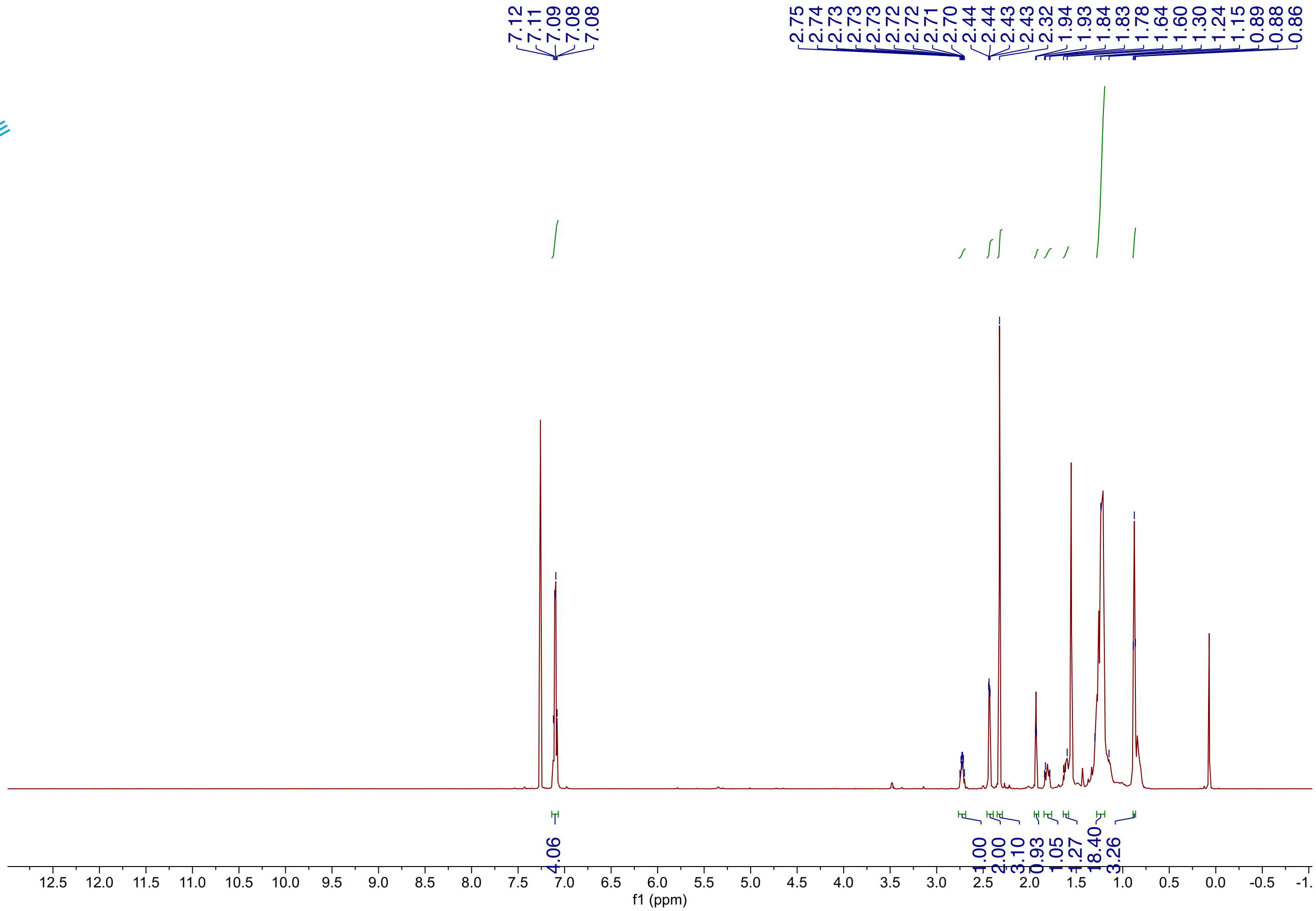

Compound 18 13C NMR

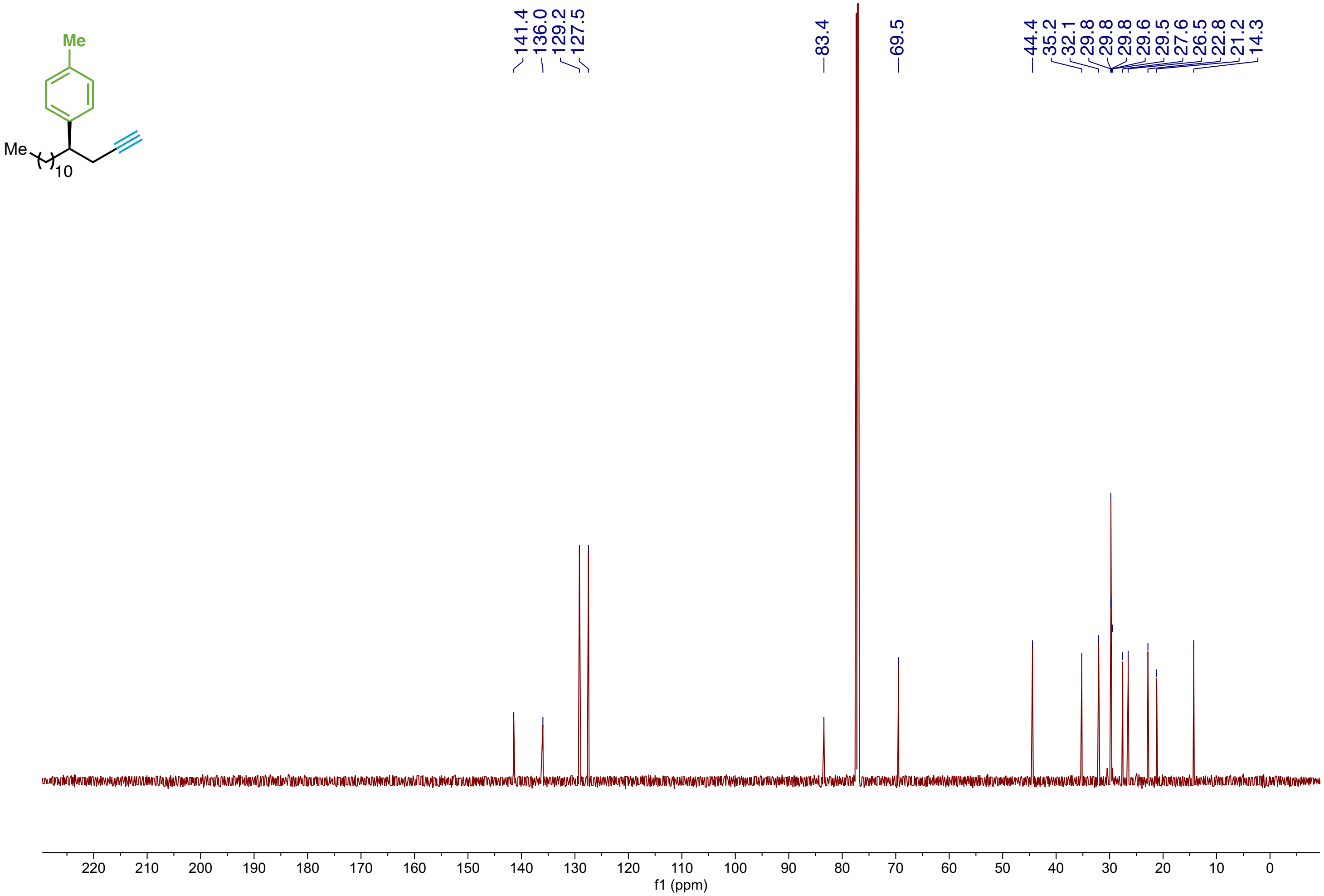

Compound 19 ^{1}H NMR

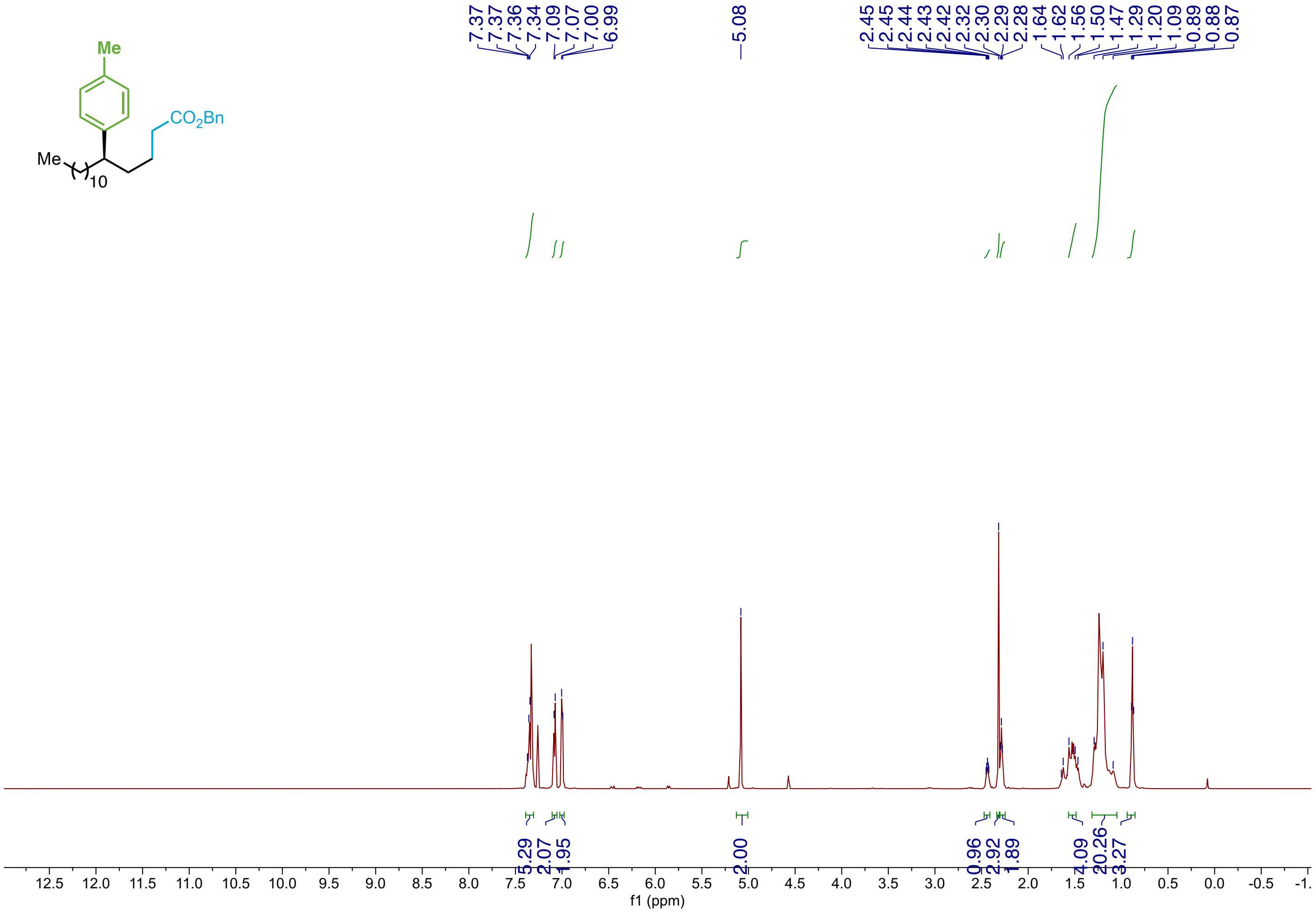

Compound 19 ^{13}C NMR

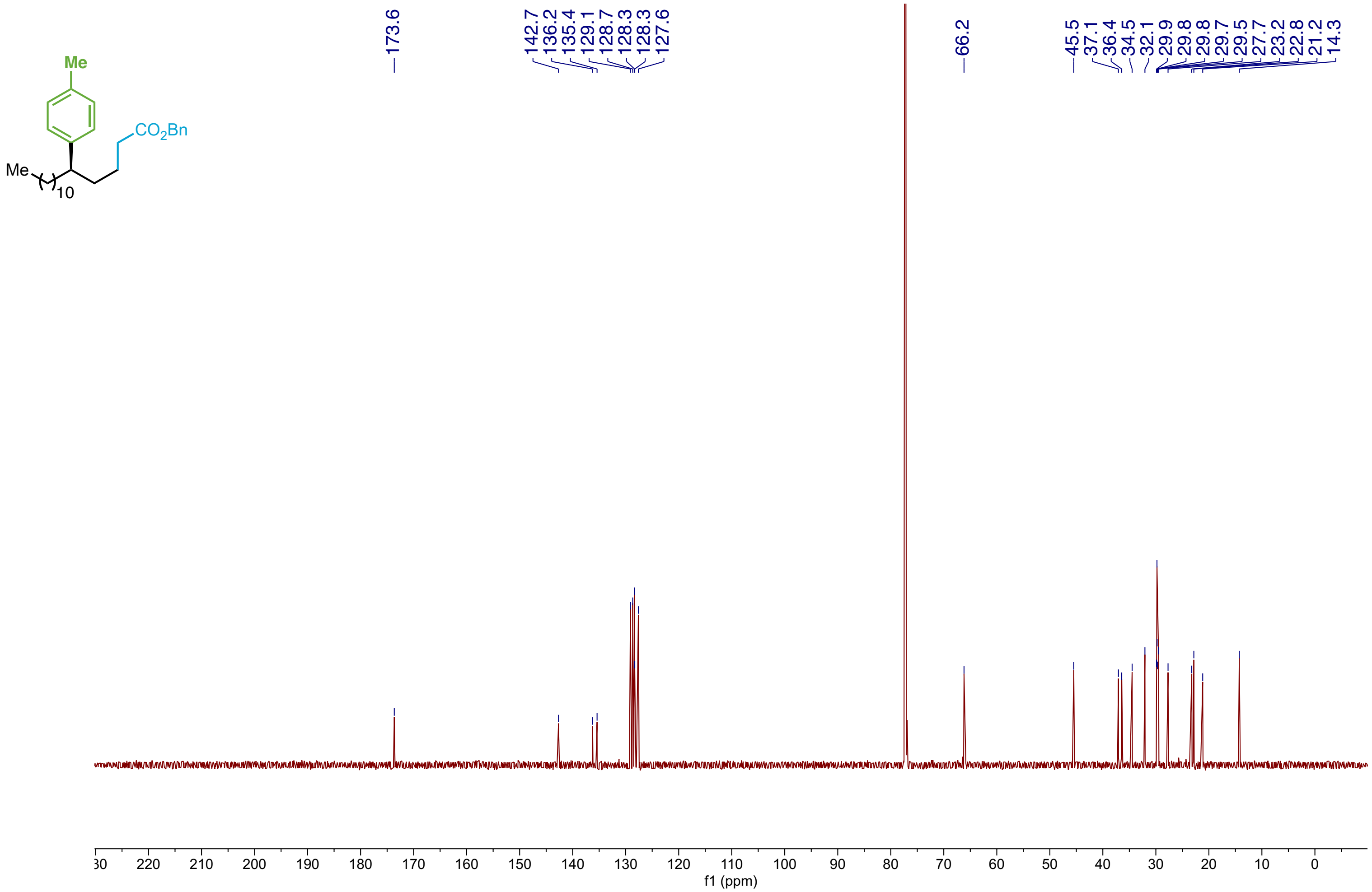

Compound 20 ^{1}H NMR

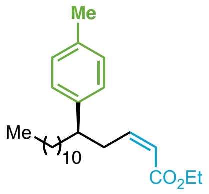

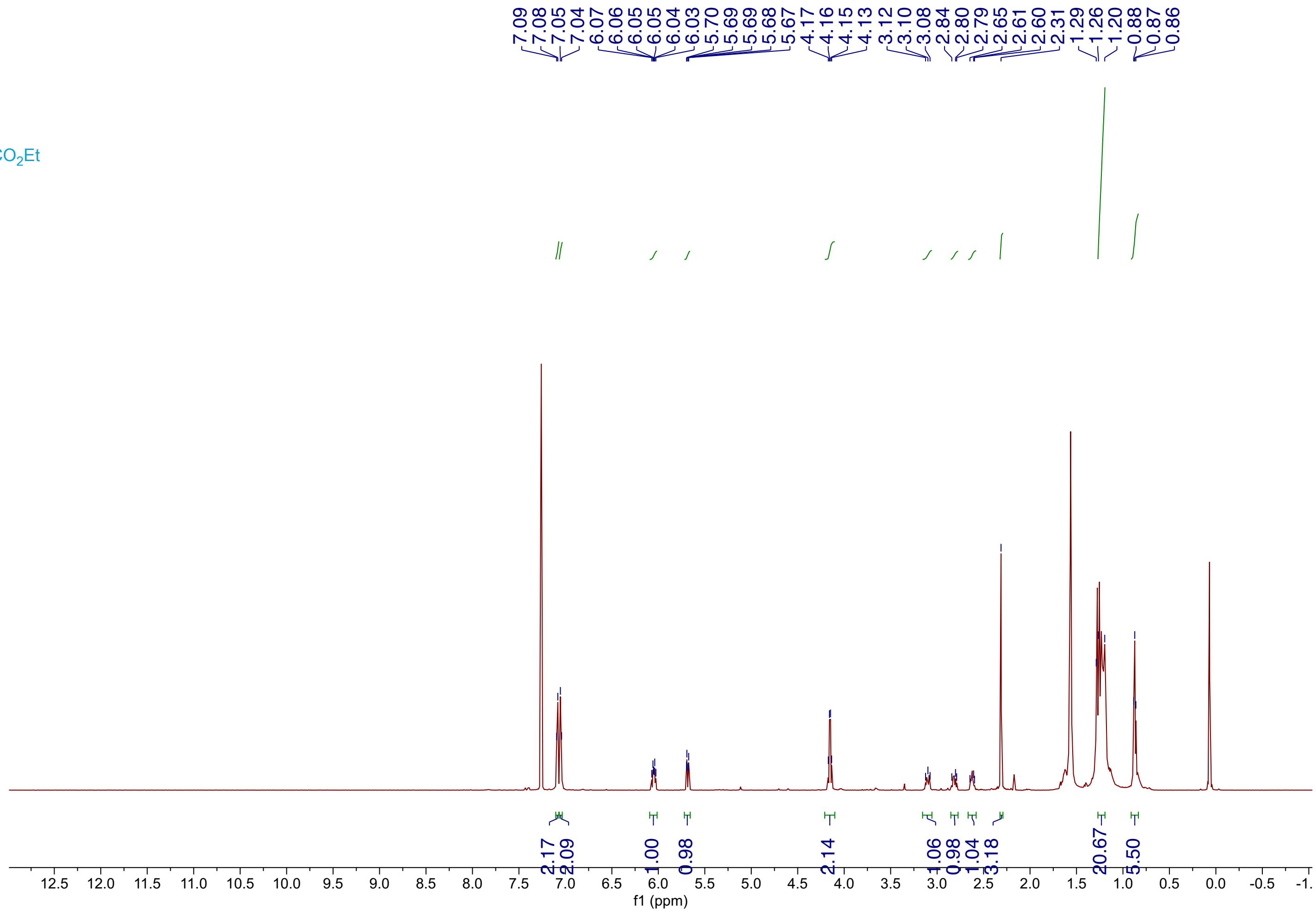

Compound 20 ^{13}C NMR

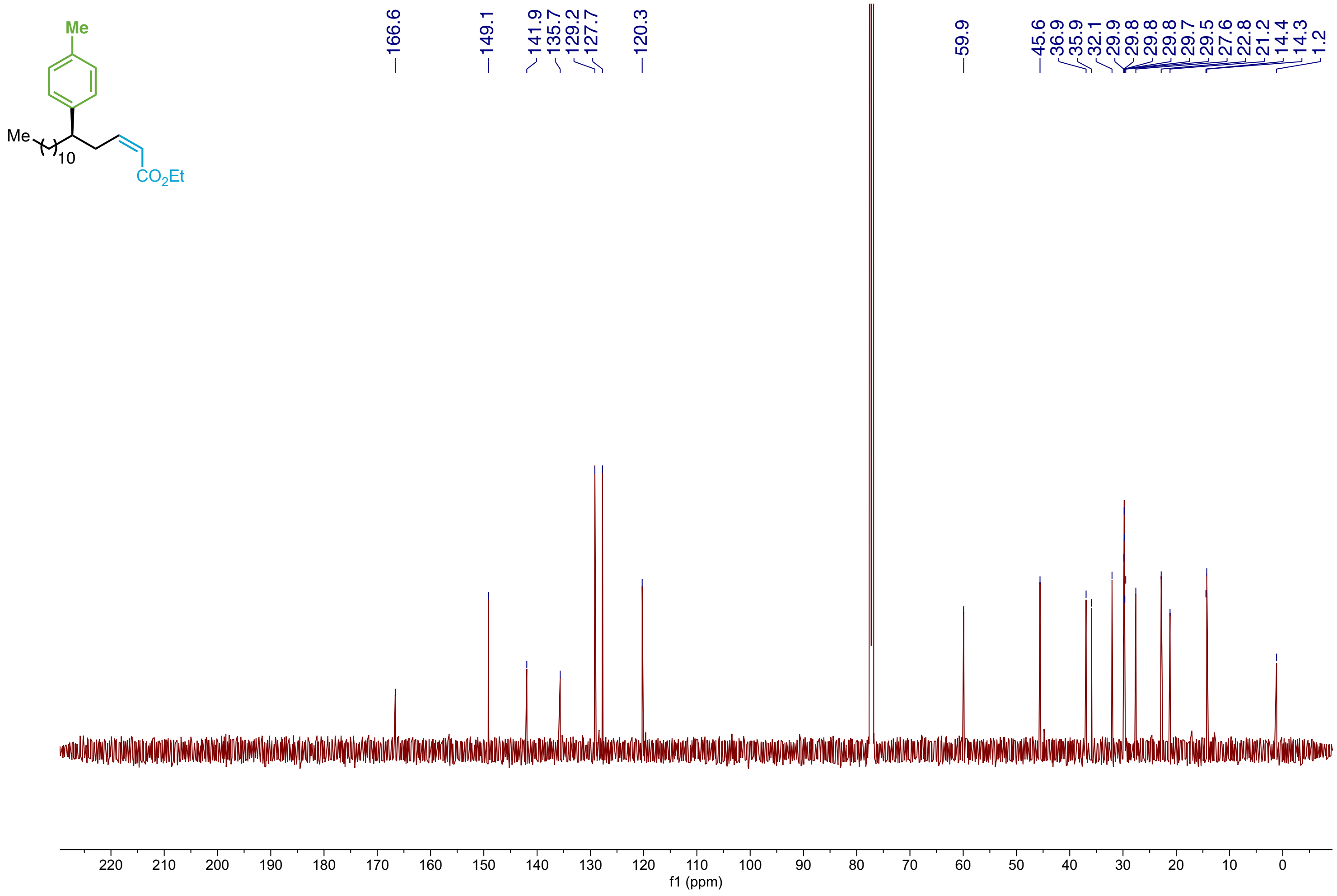

Compound 21 ^{1}H NMR

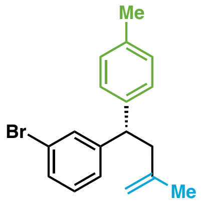

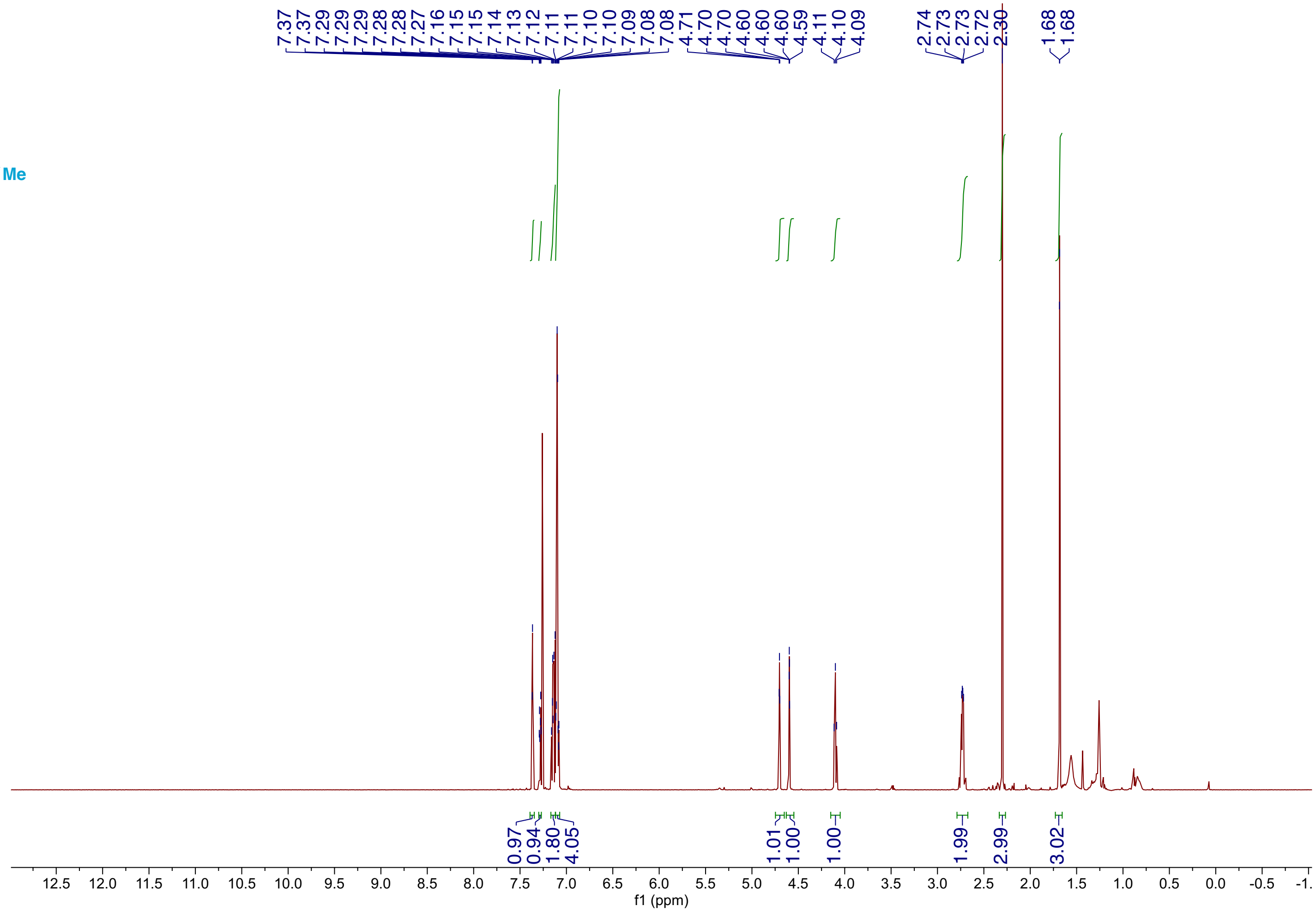

Compound 21 ^{13}C NMR

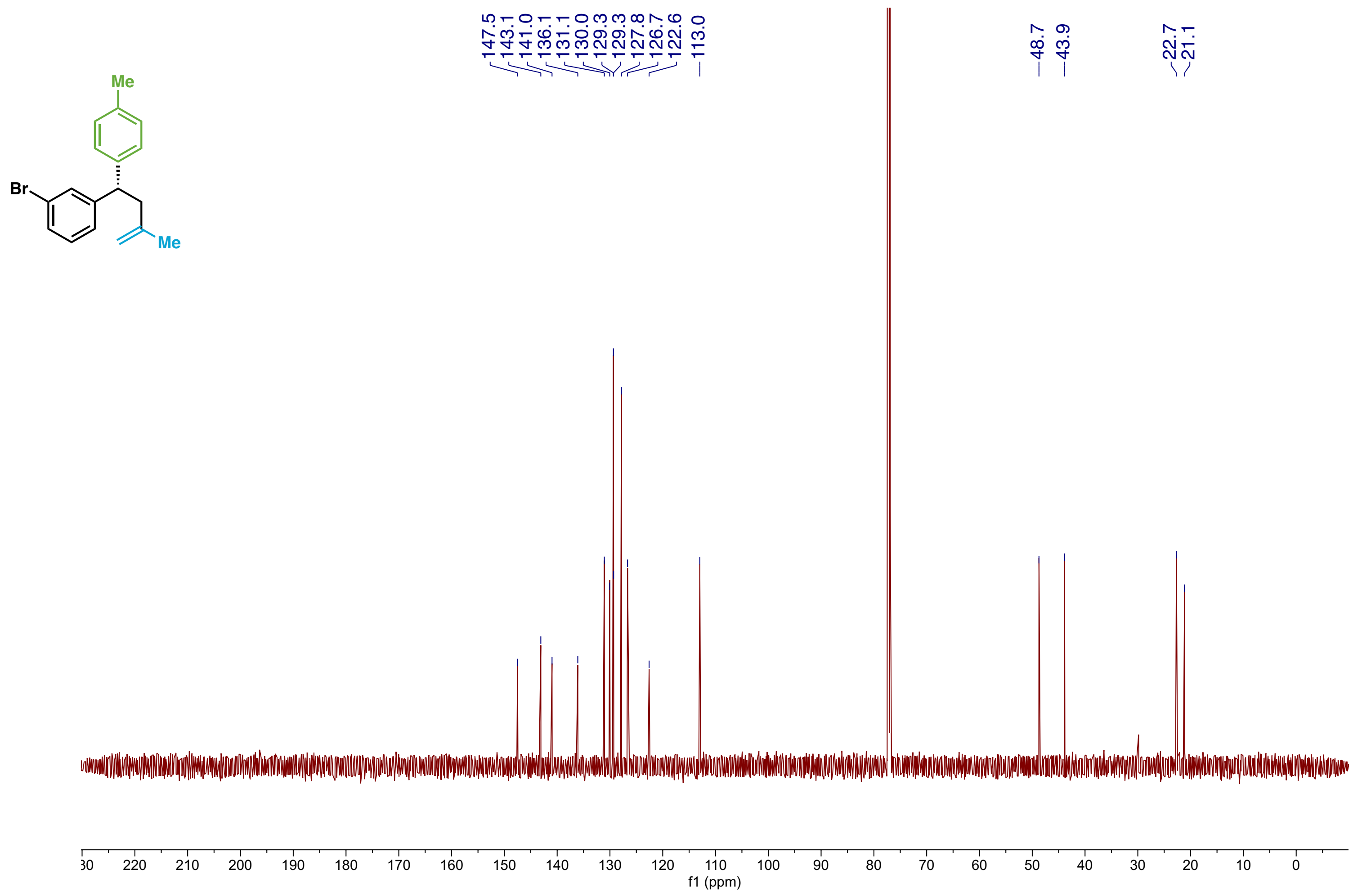

Compound 22 ^{1}H NMR

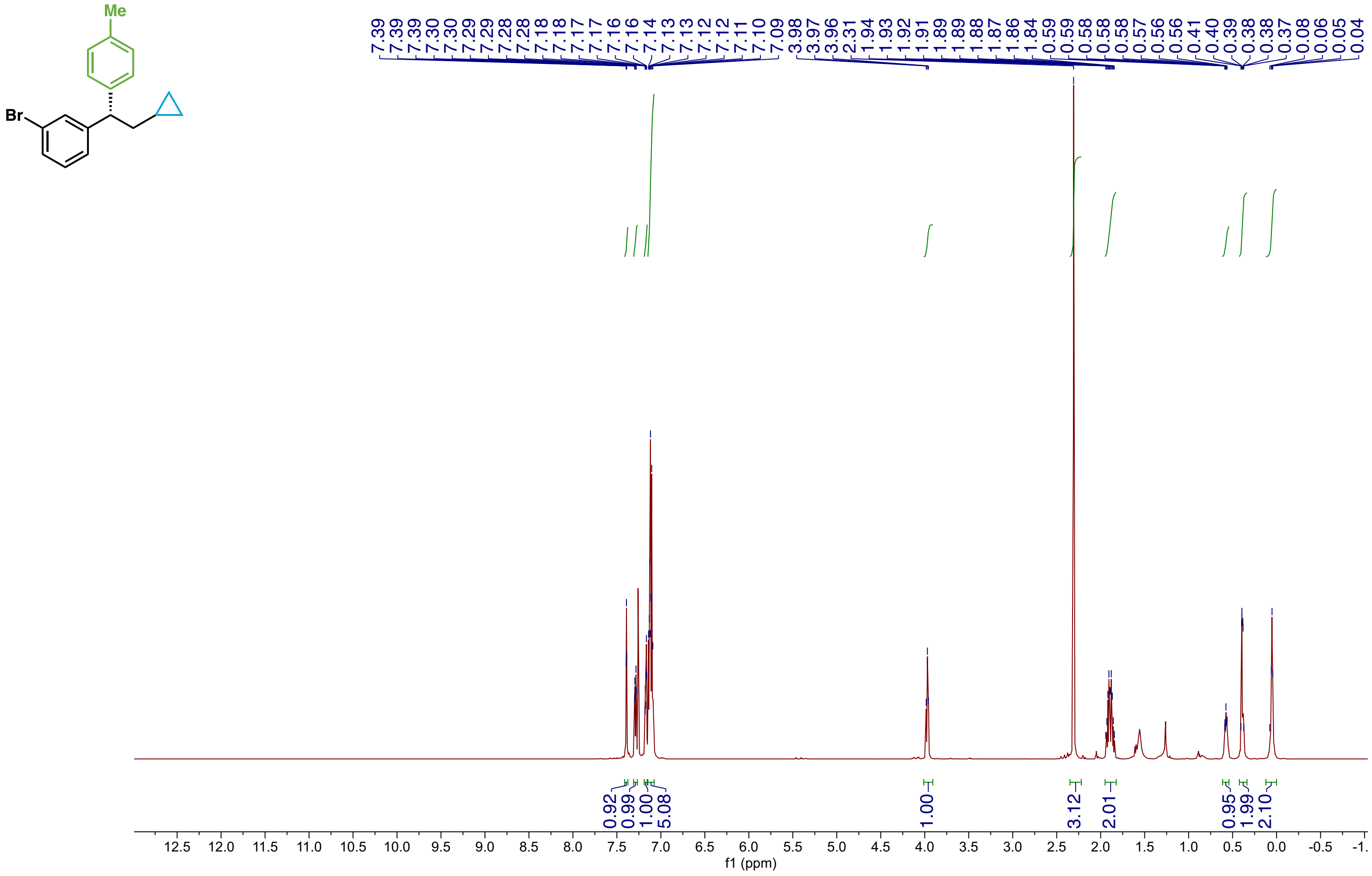

Compound 22 ^{13}C NMR

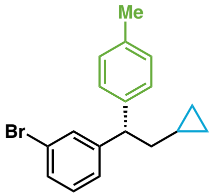

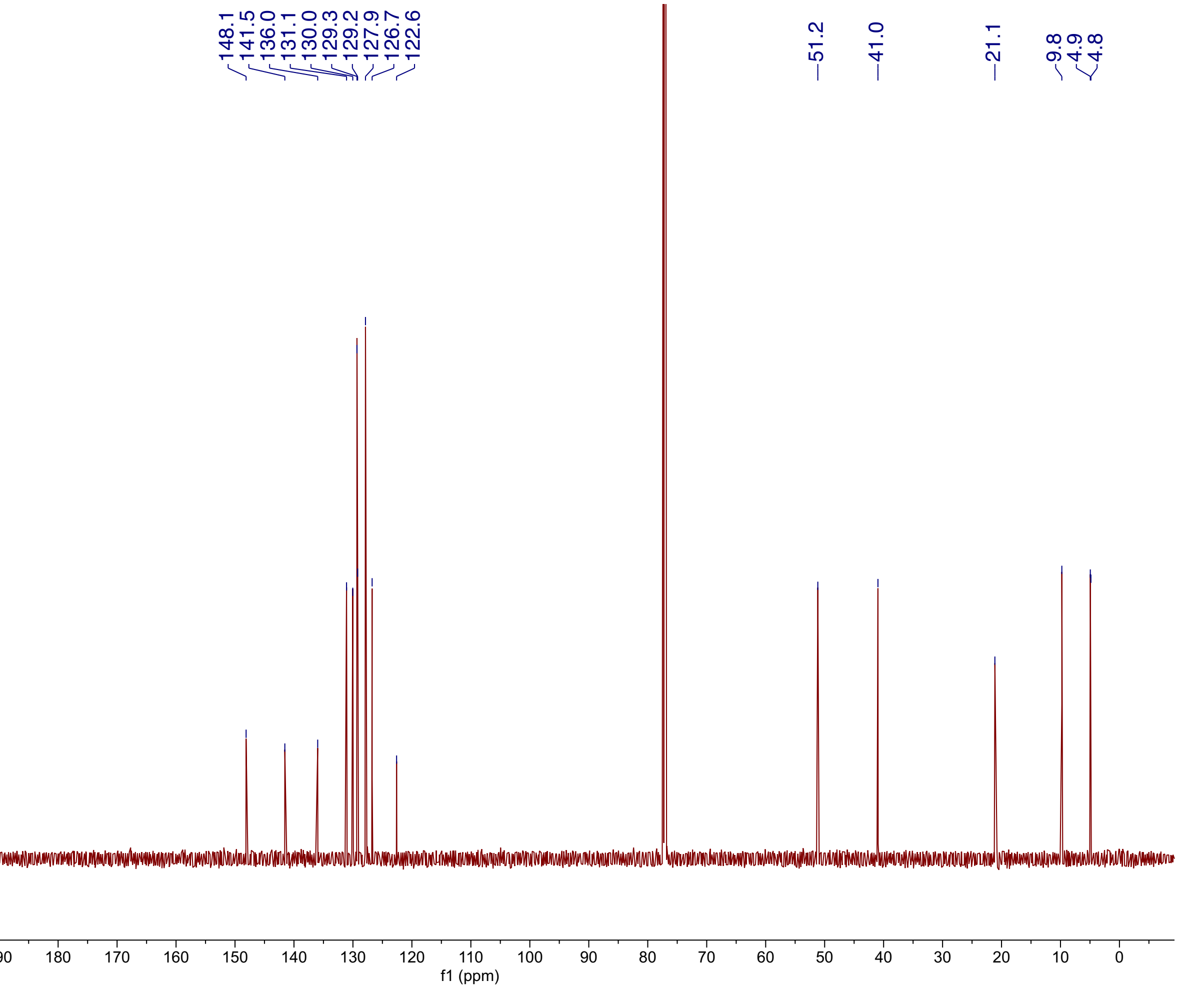

Compound 23 ^{1}H NMR

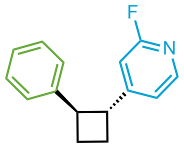

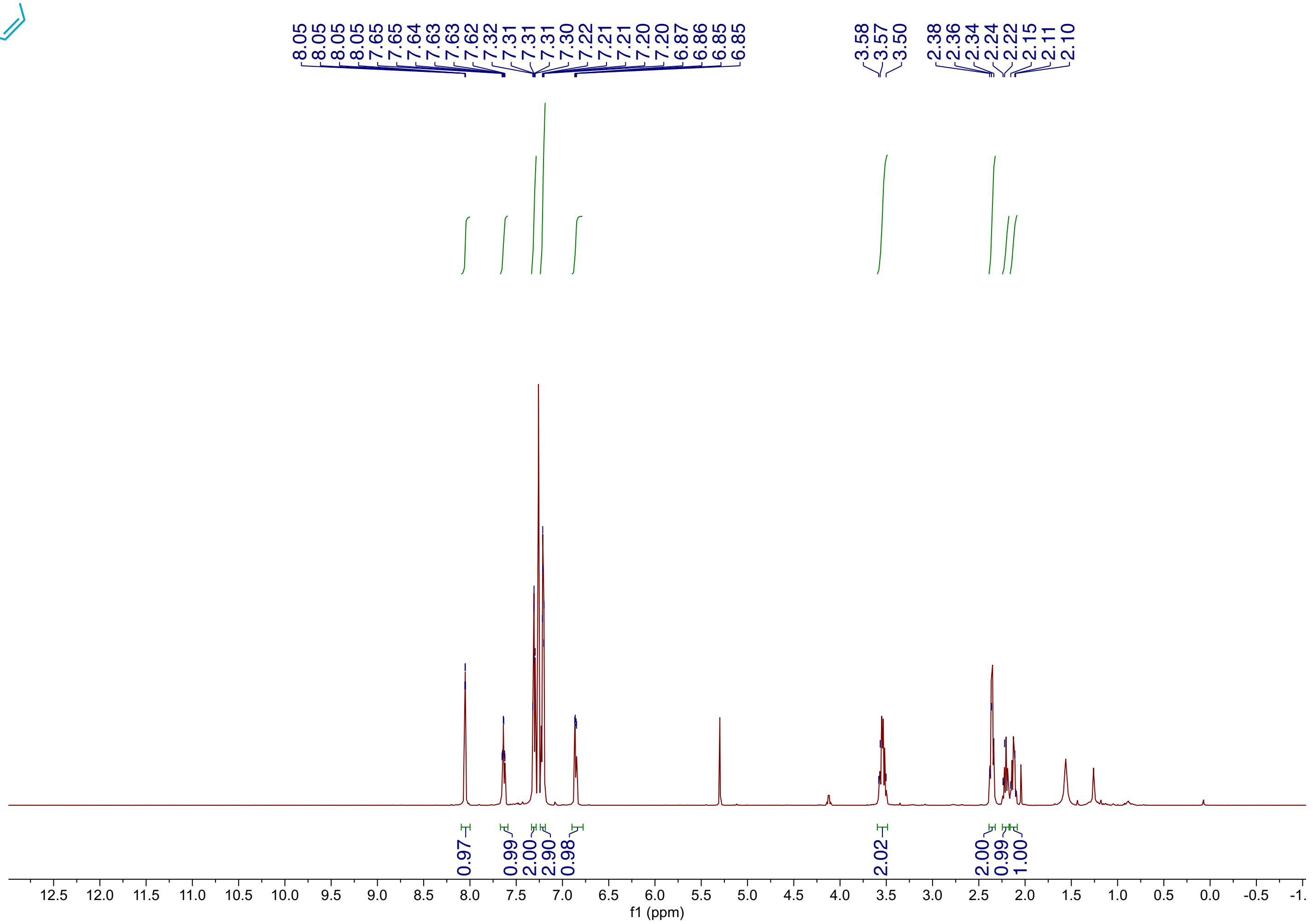

Compound 23 ^{19}F NMR

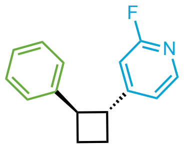

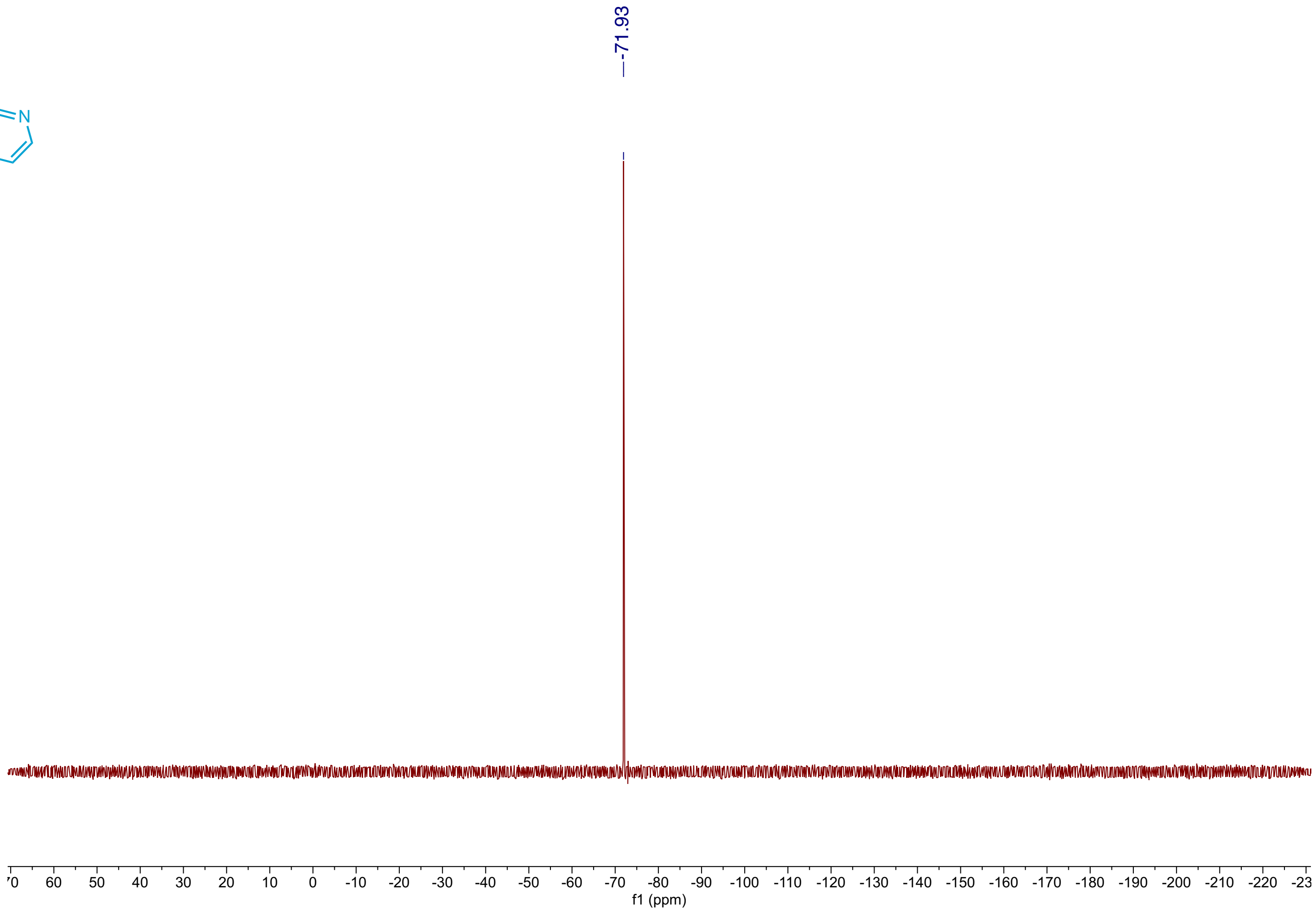

Compound 23 ^{13}C NMR

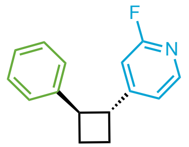

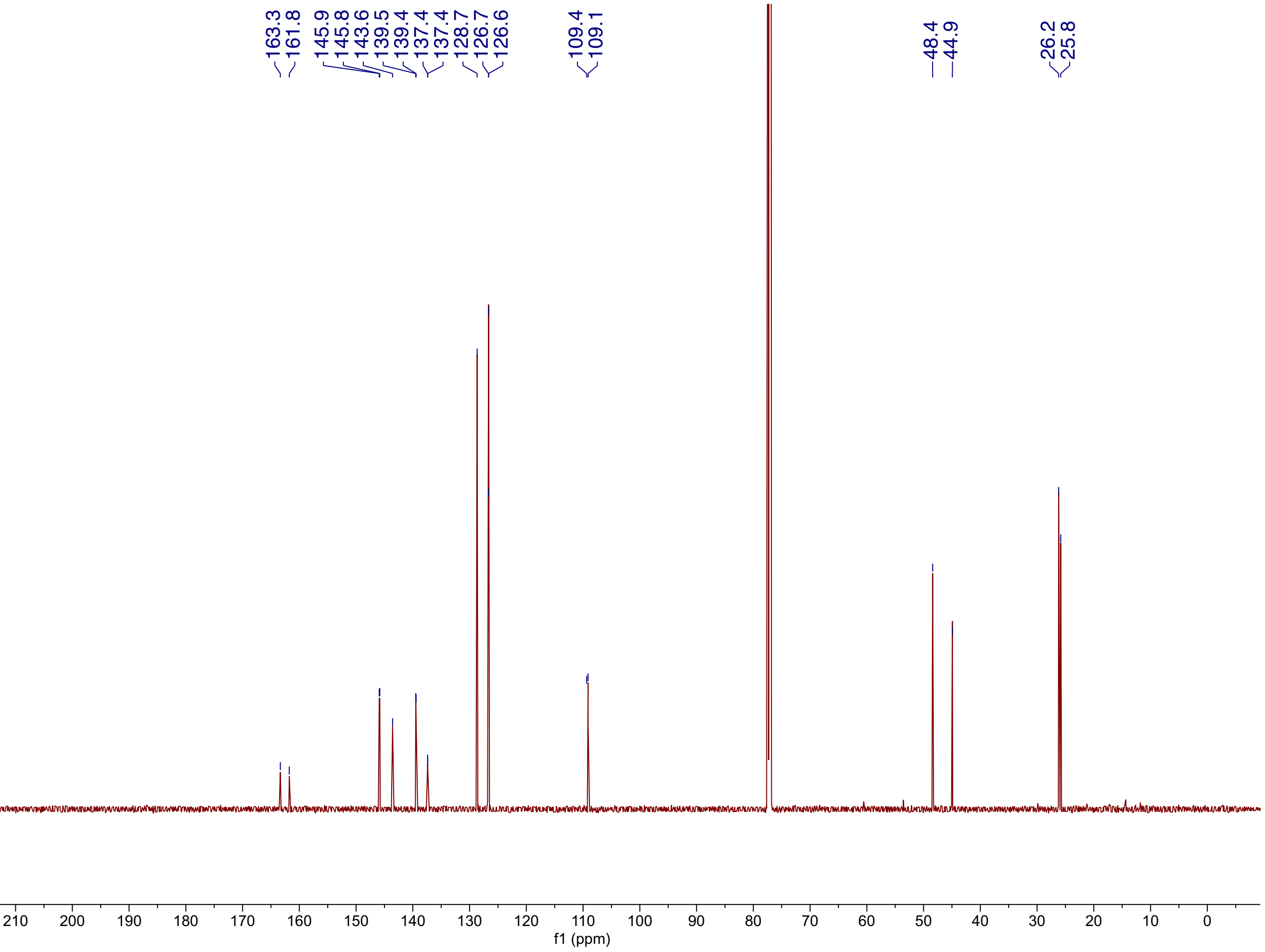

Compound 24 ^{1}H NMR

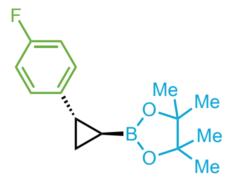

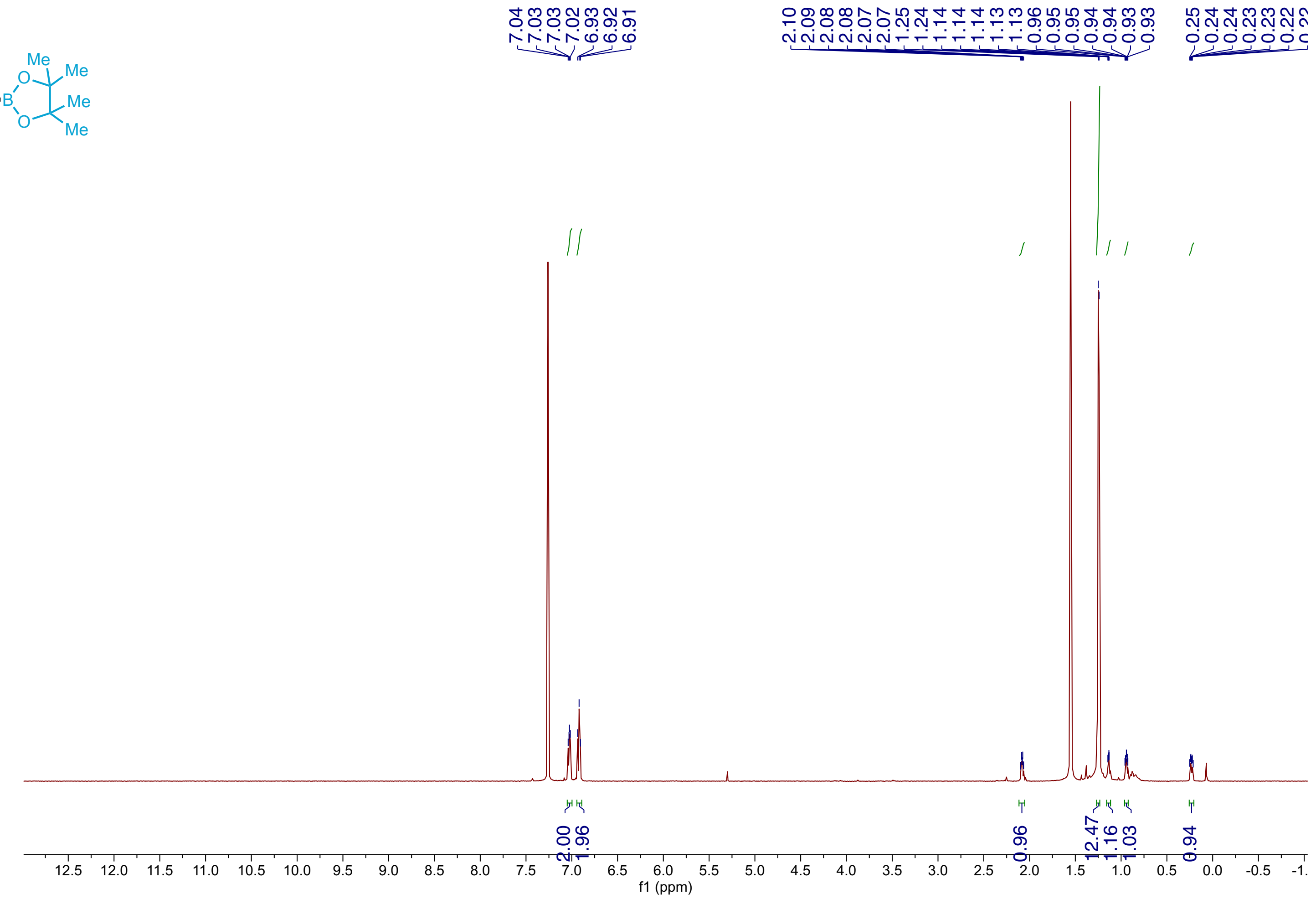

Compound 24 ^{19}F NMR

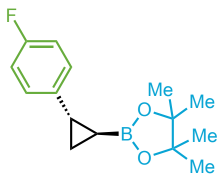

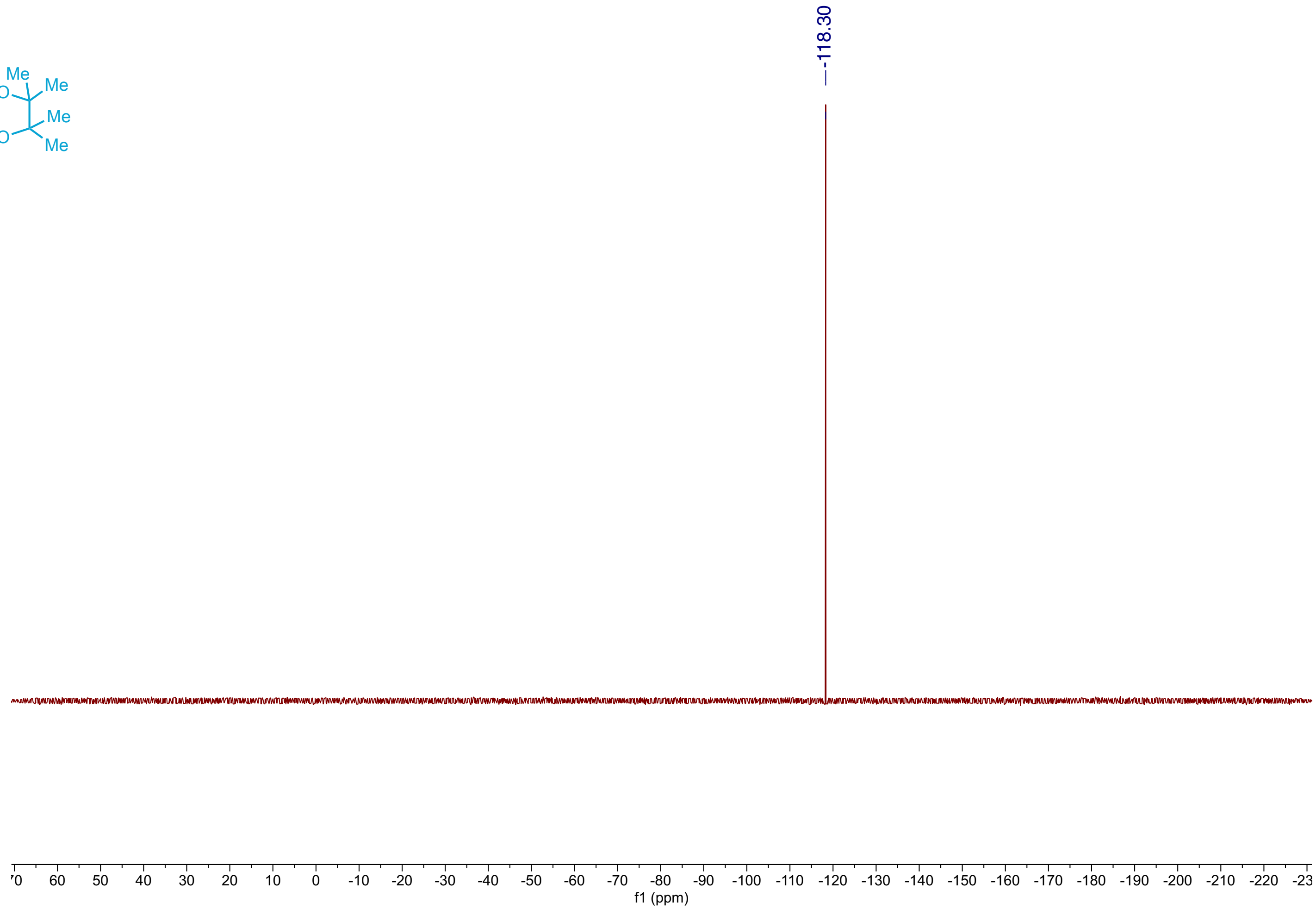

Compound 24 ^{13}C NMR

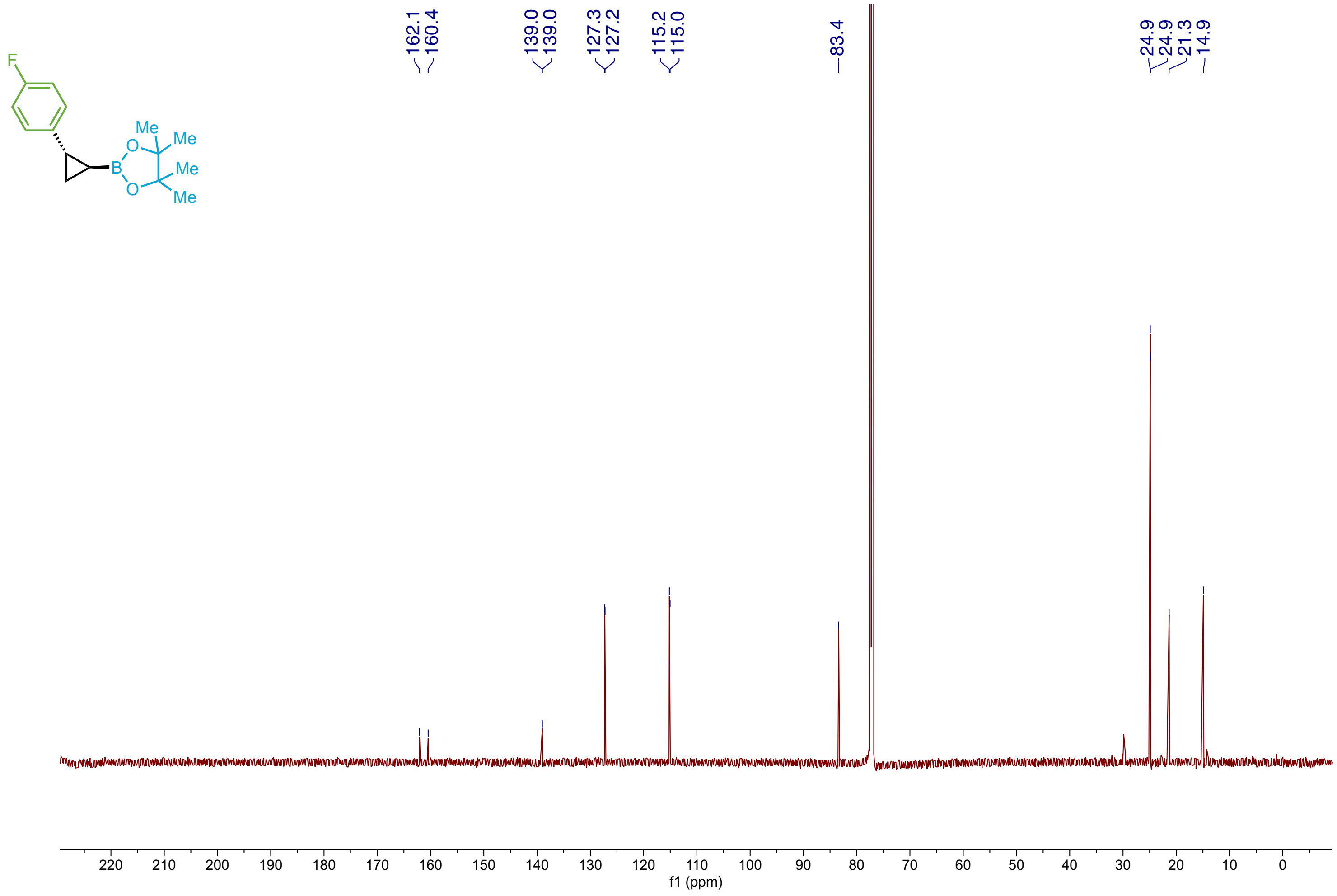

Compound B8 ^{1}H NMR

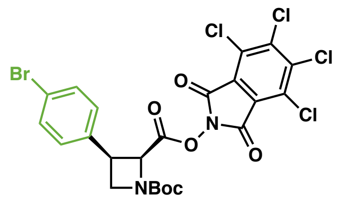

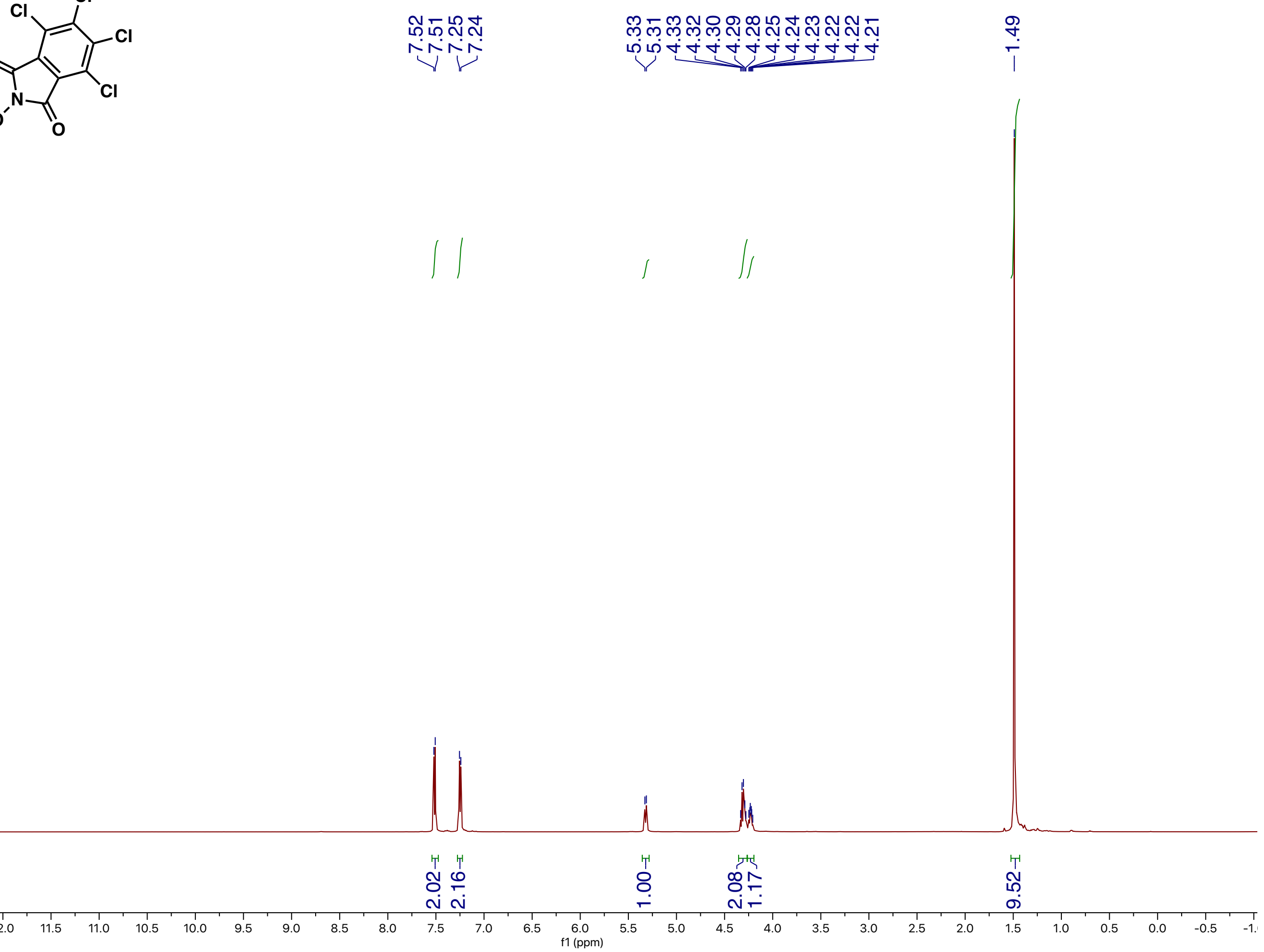

Compound B8 ^{13}C NMR

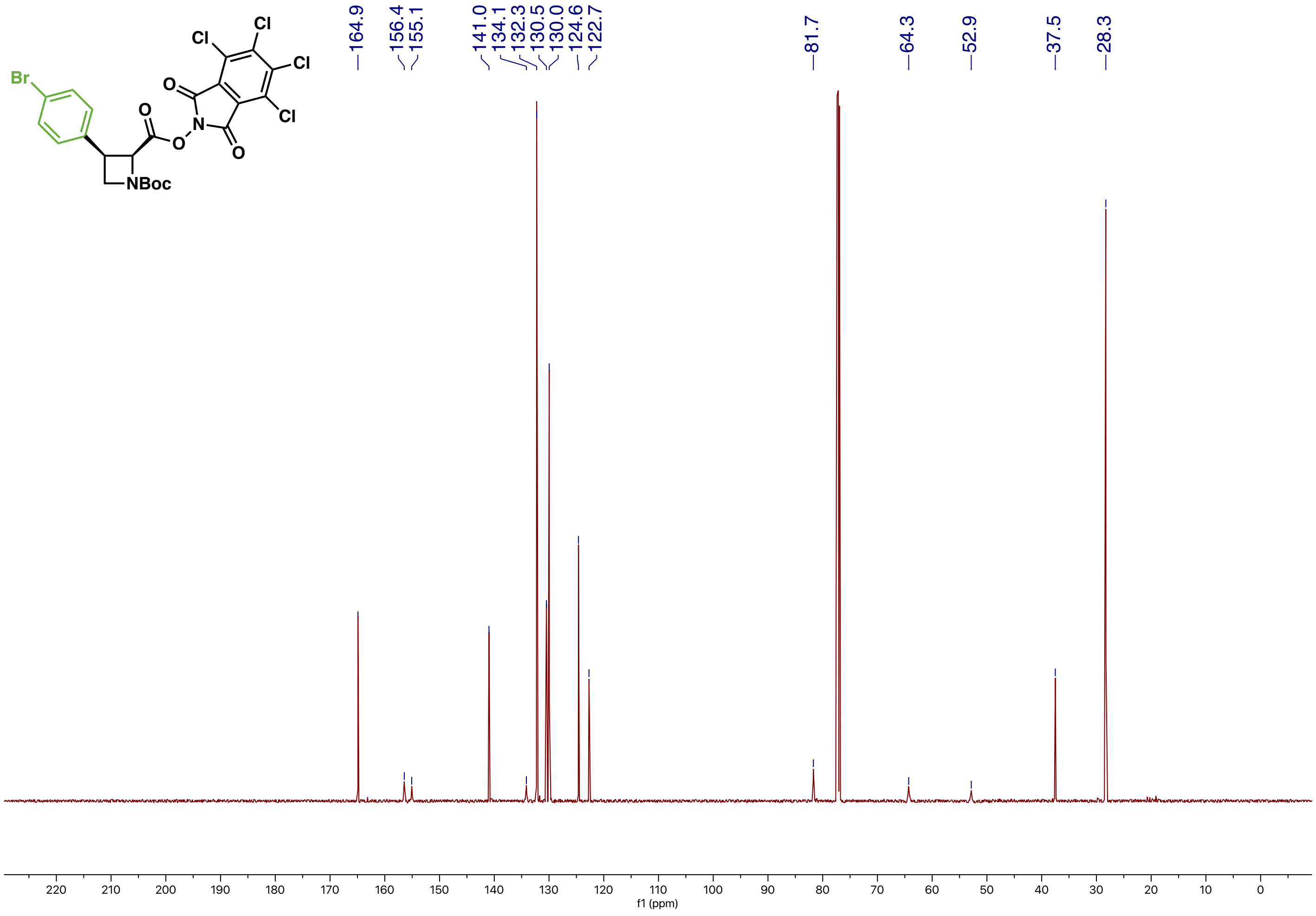

Compound 26 ^{1}H NMR

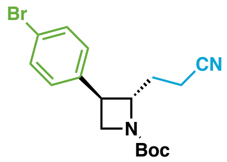

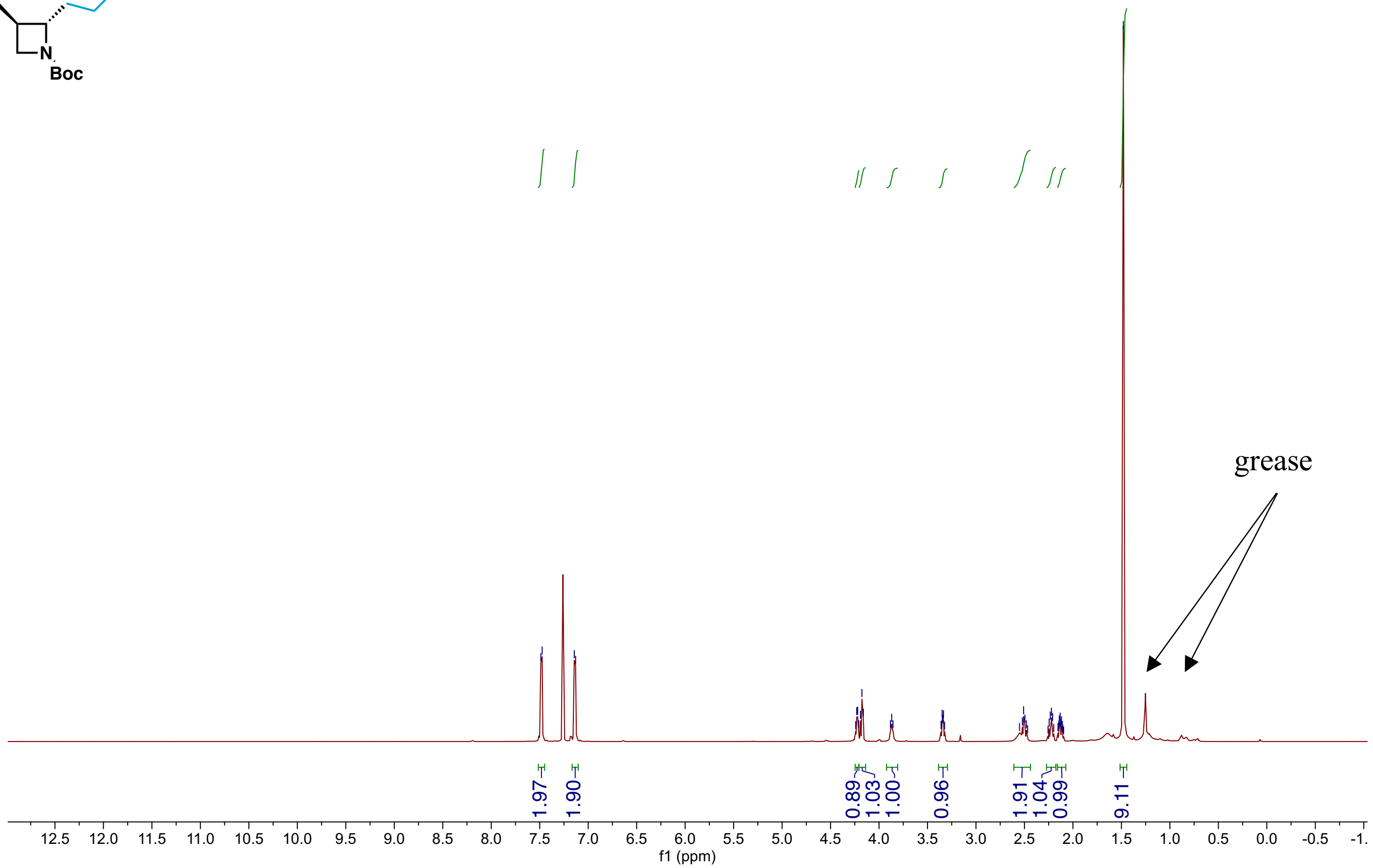

Compound 26 ^{13}C NMR

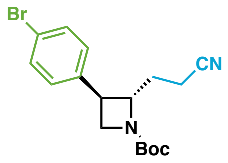

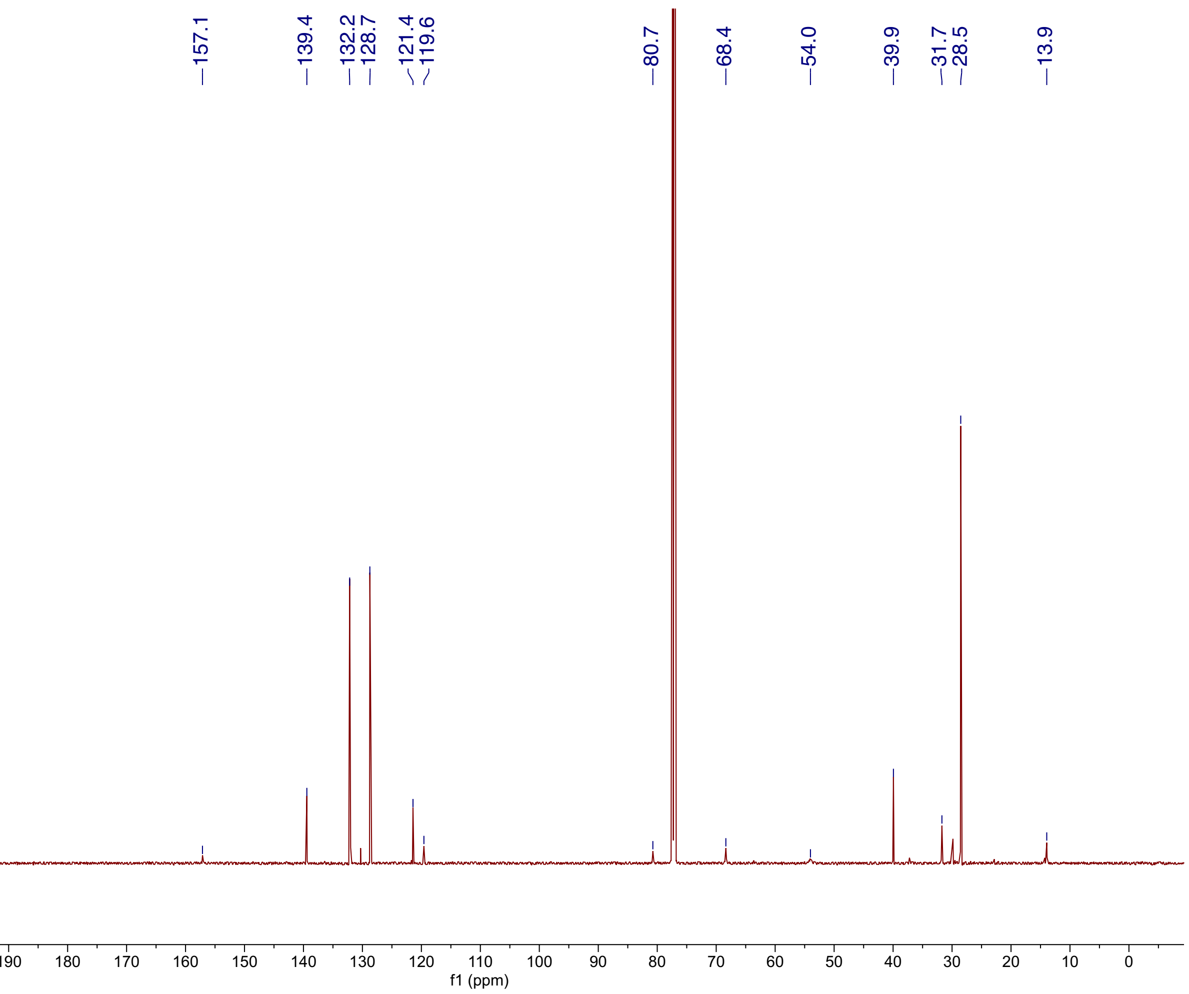

Compound 27 ^{1}H NMR

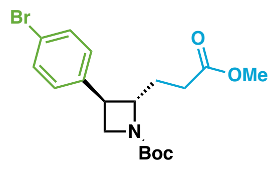

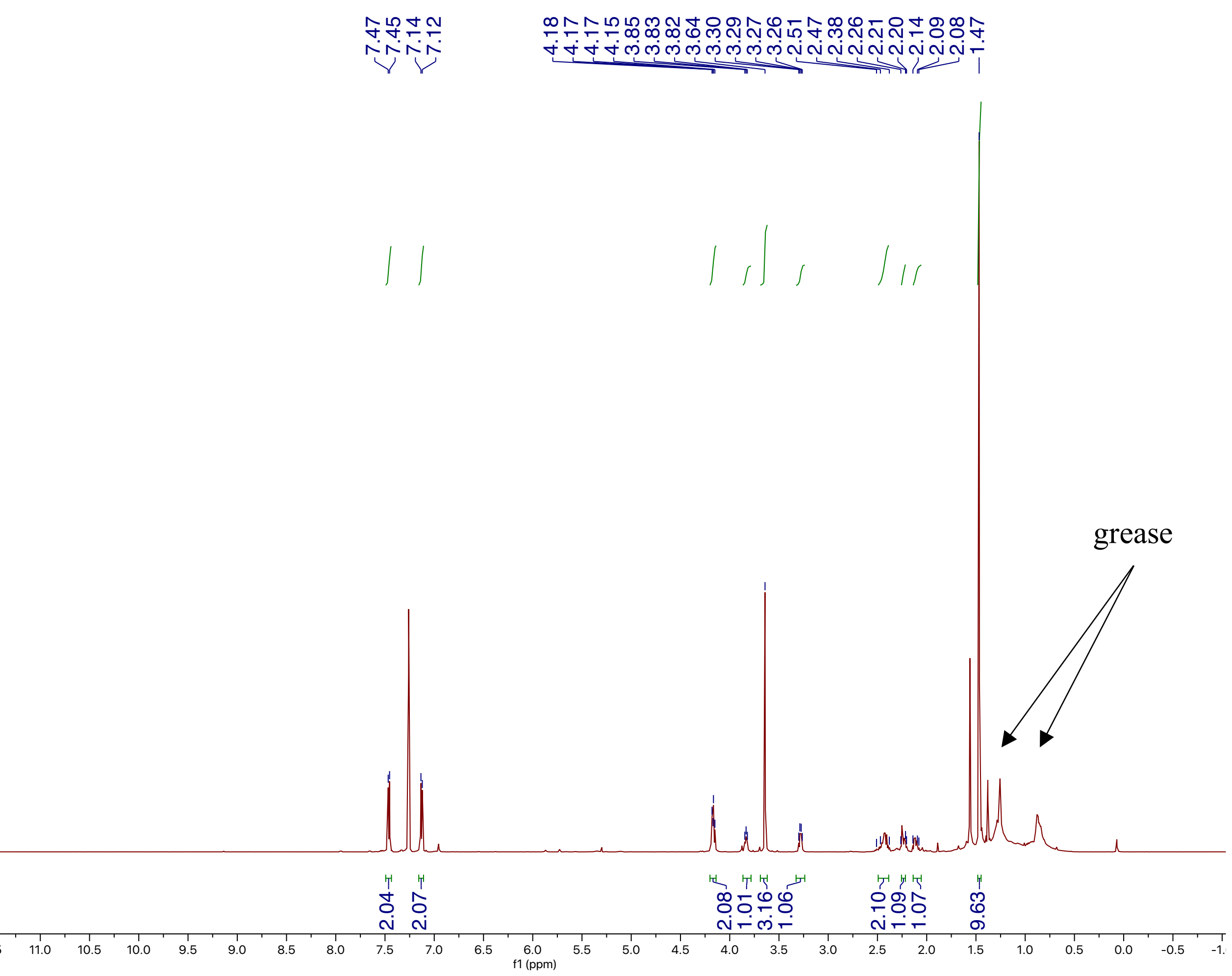

Compound 27 ^{13}C NMR

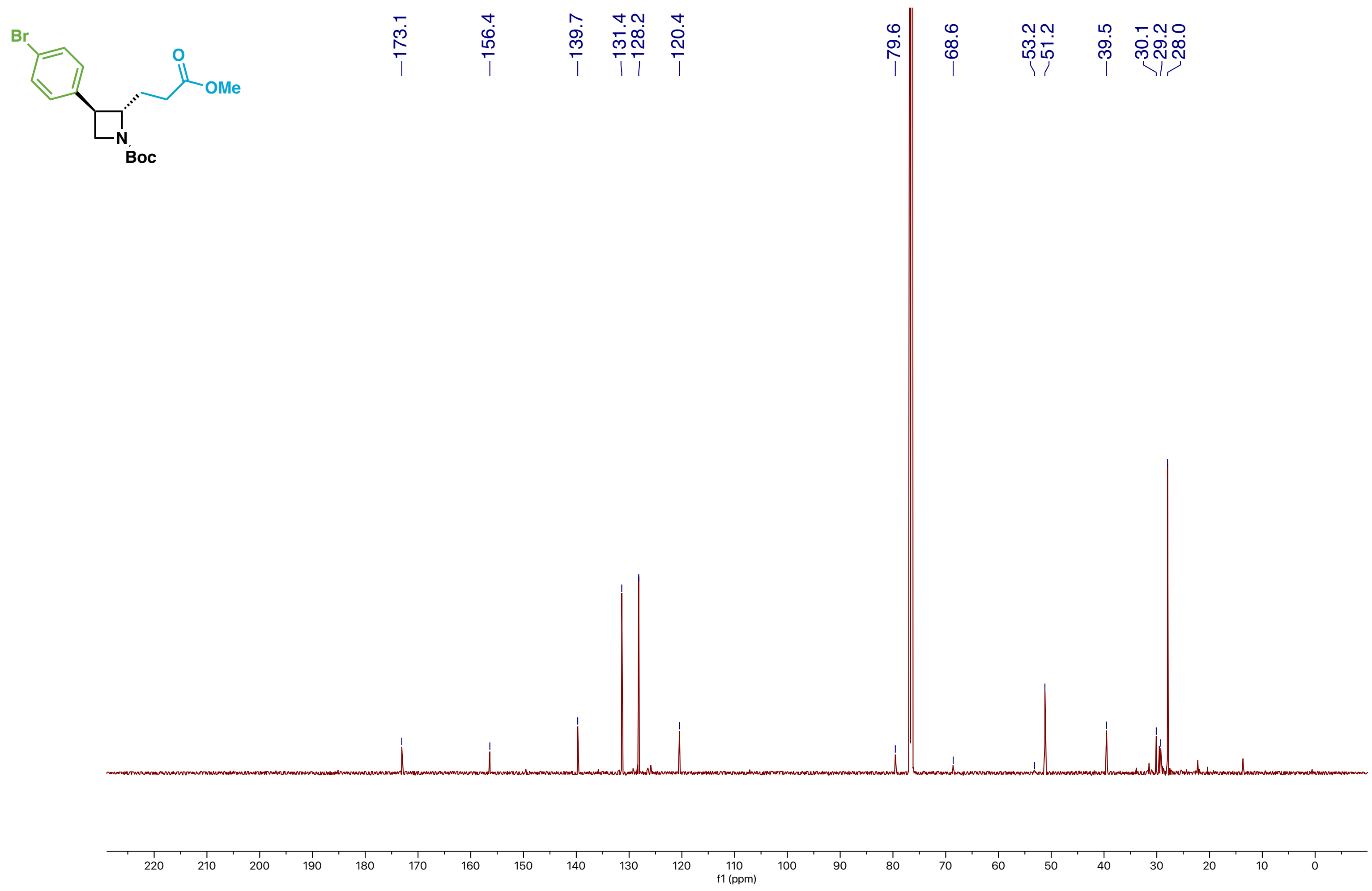

Compound 28 ^{1}H NMR

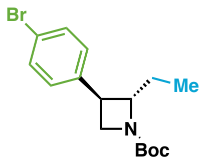

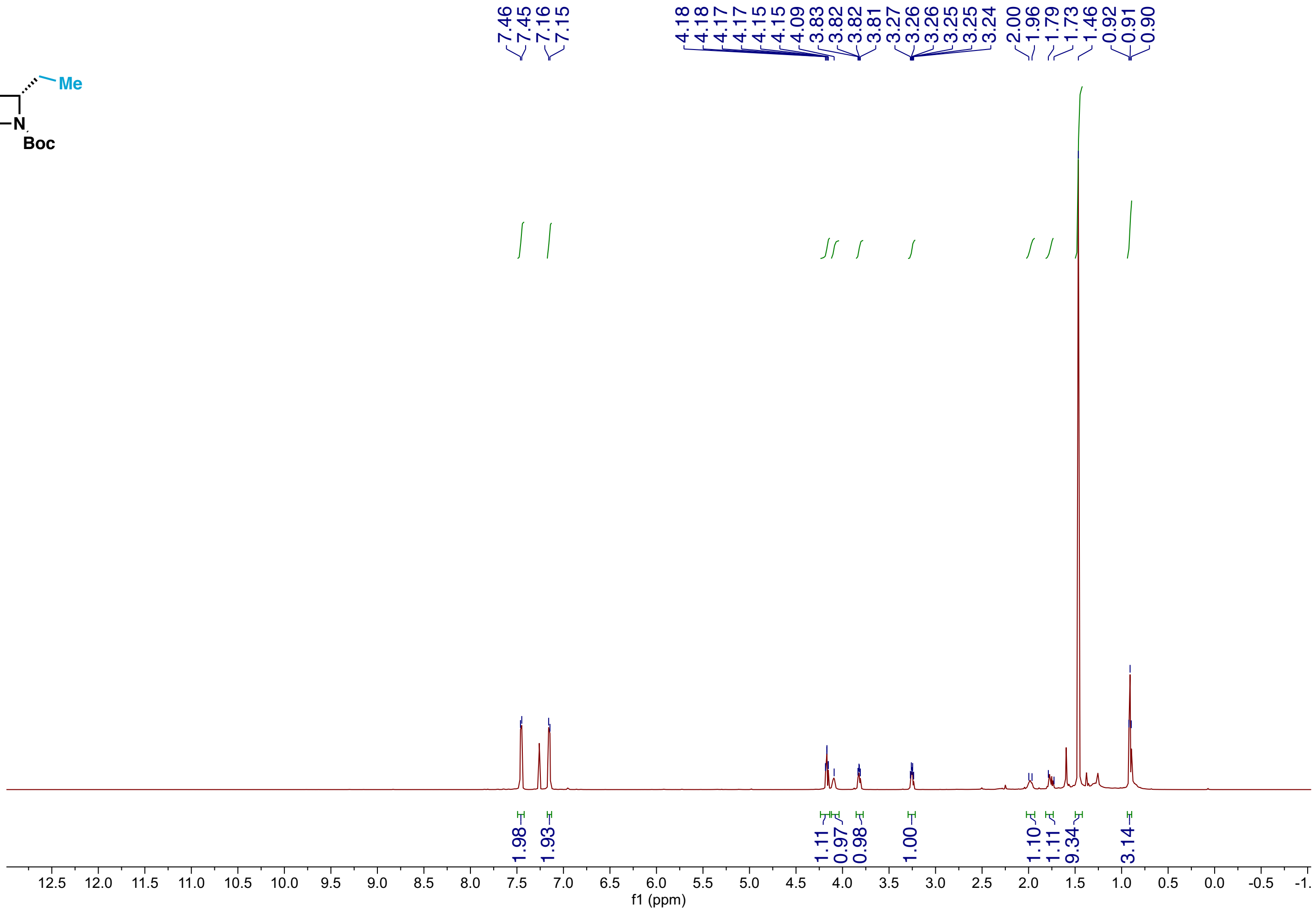

Compound 28 ^{13}C NMR

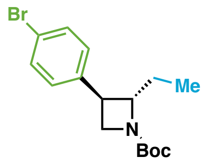

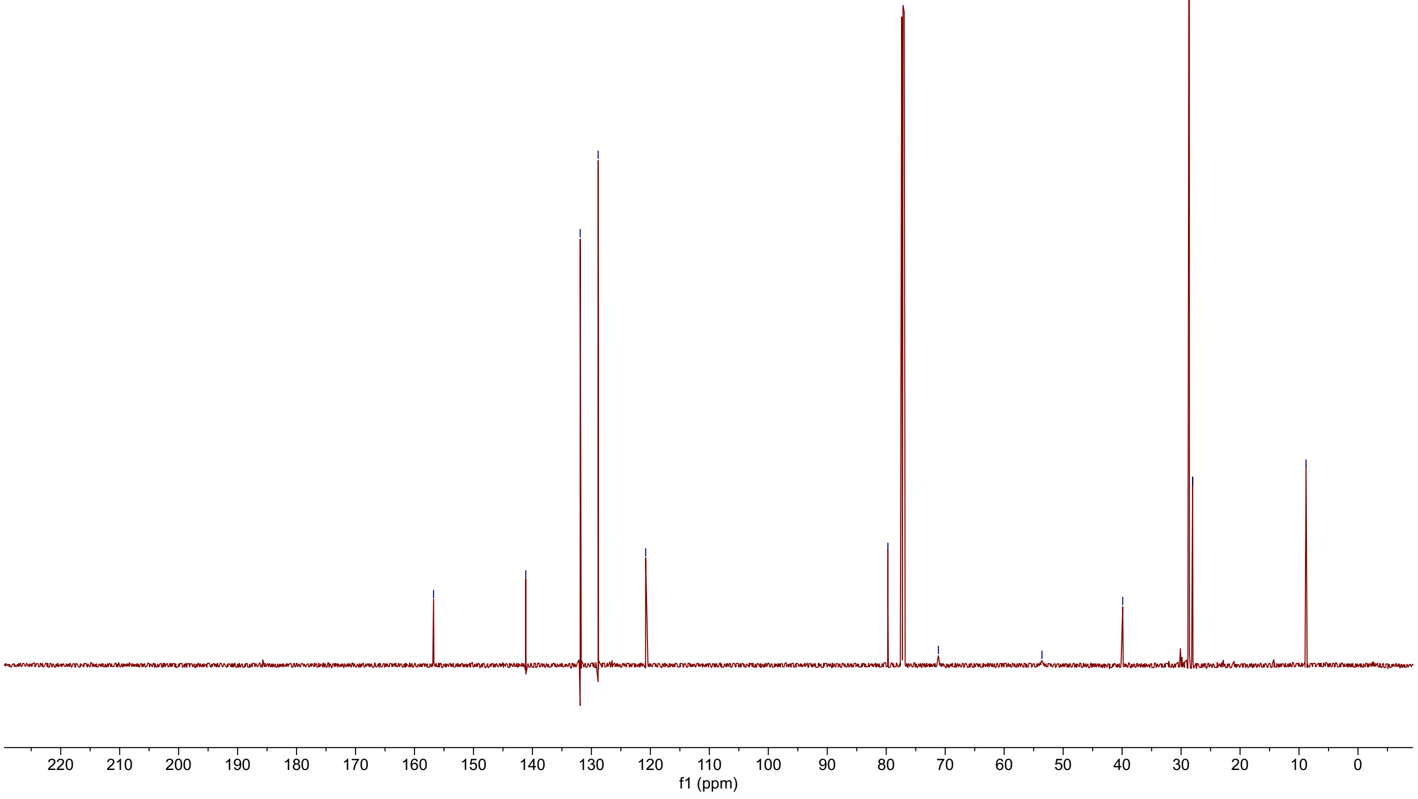

Compound 29 ^{1}H NMR

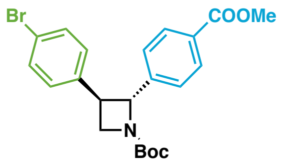

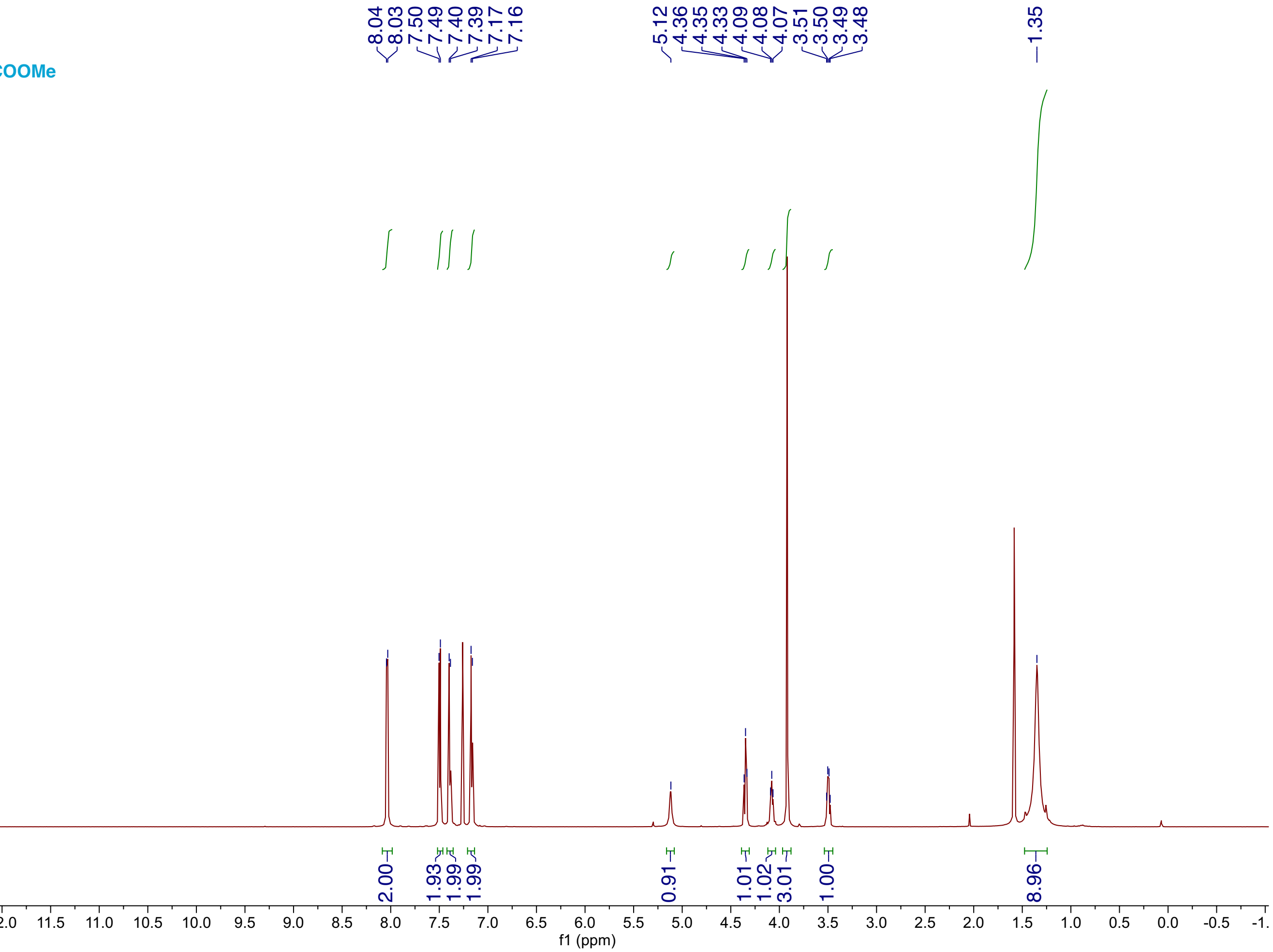

Compound 29 ^{13}C NMR

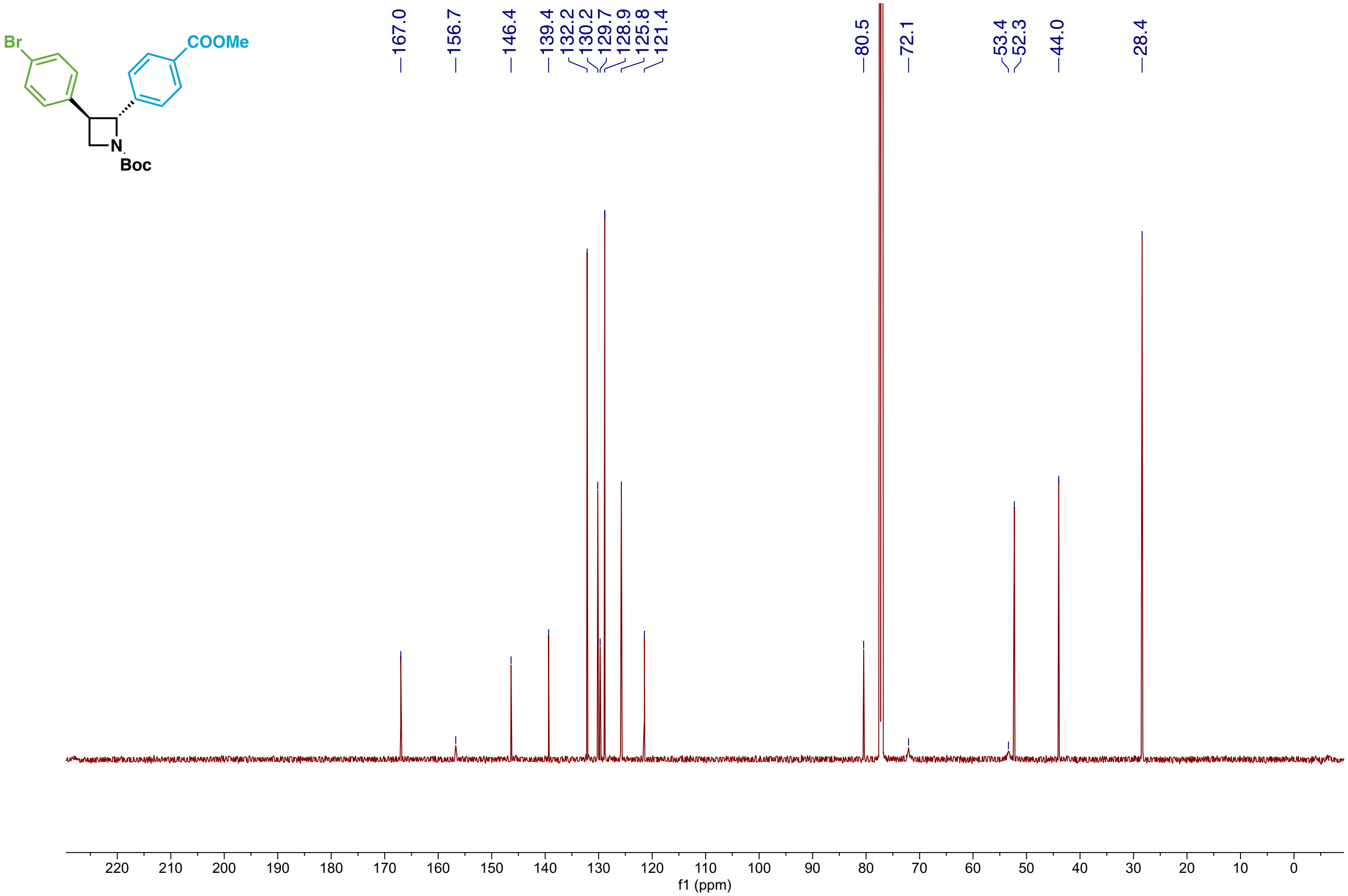

Compound 30 ^{1}H NMR

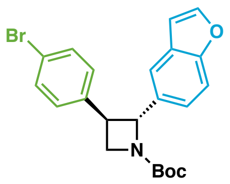

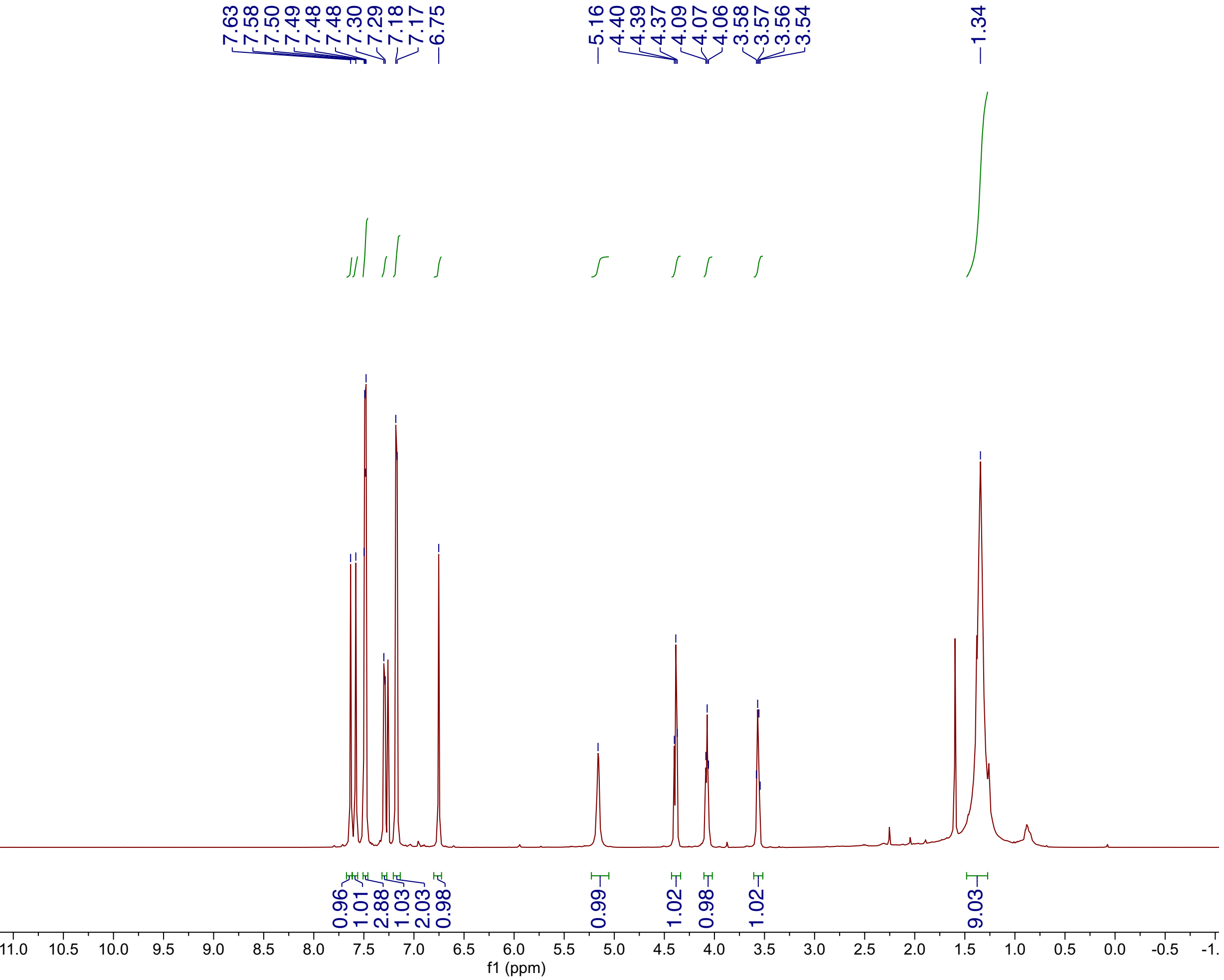

Compound 30 ^{13}C NMR

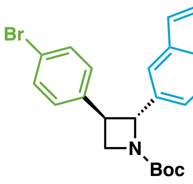

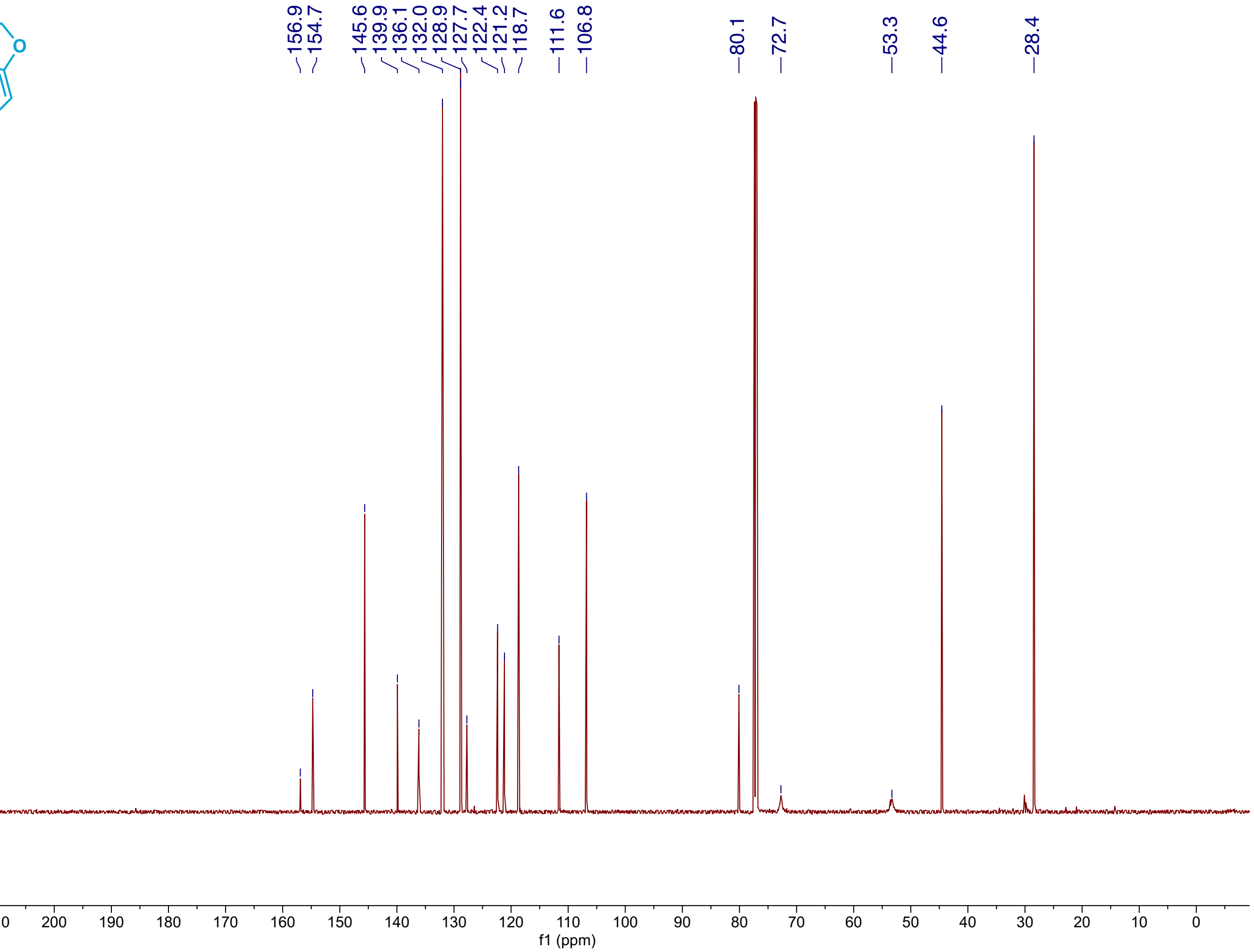

Compound 31 ^{1}H NMR

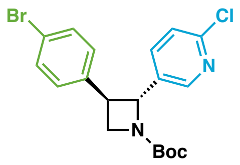

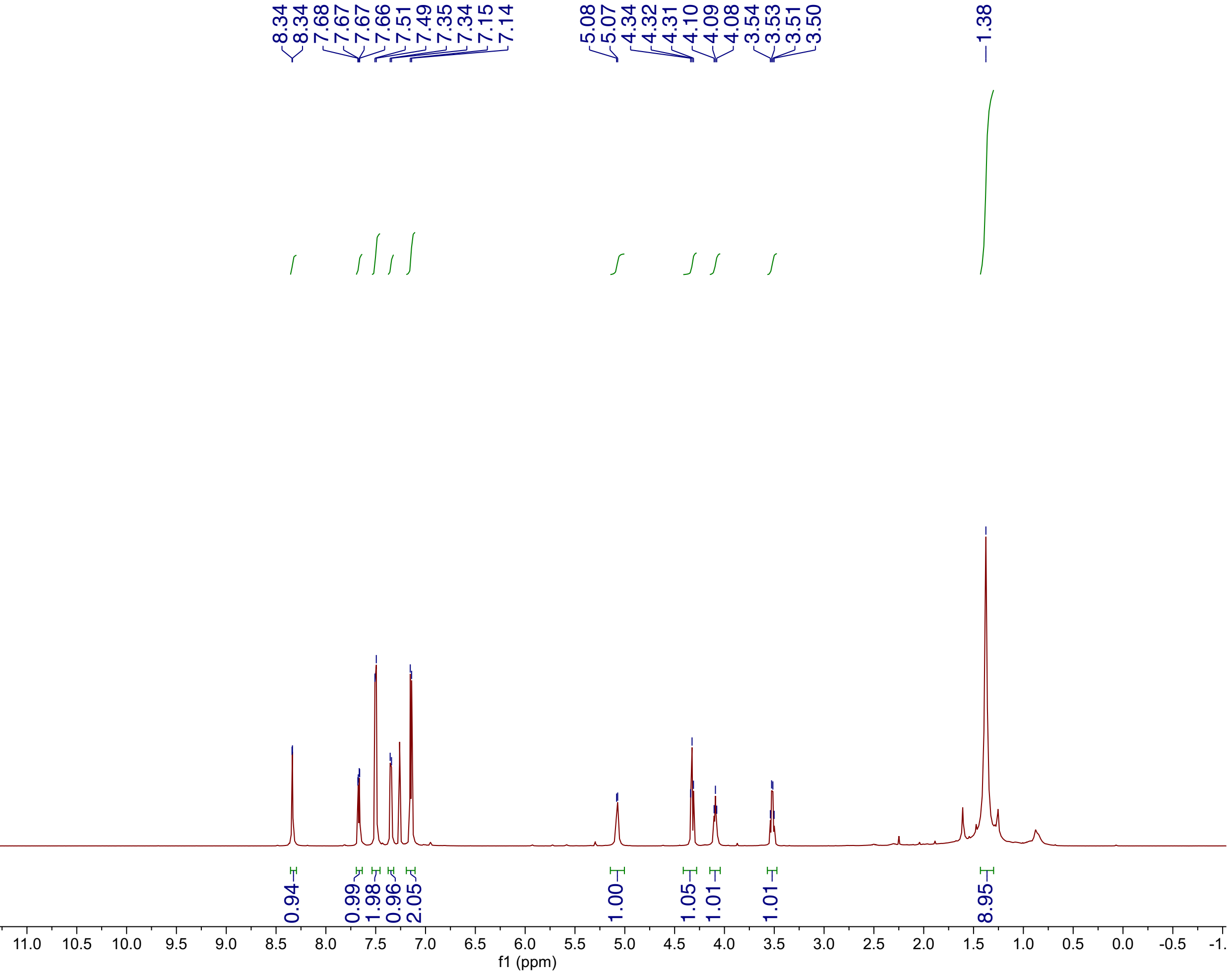

Compound 31 ^{13}C NMR

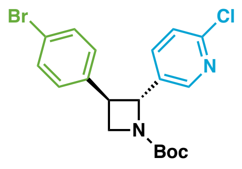

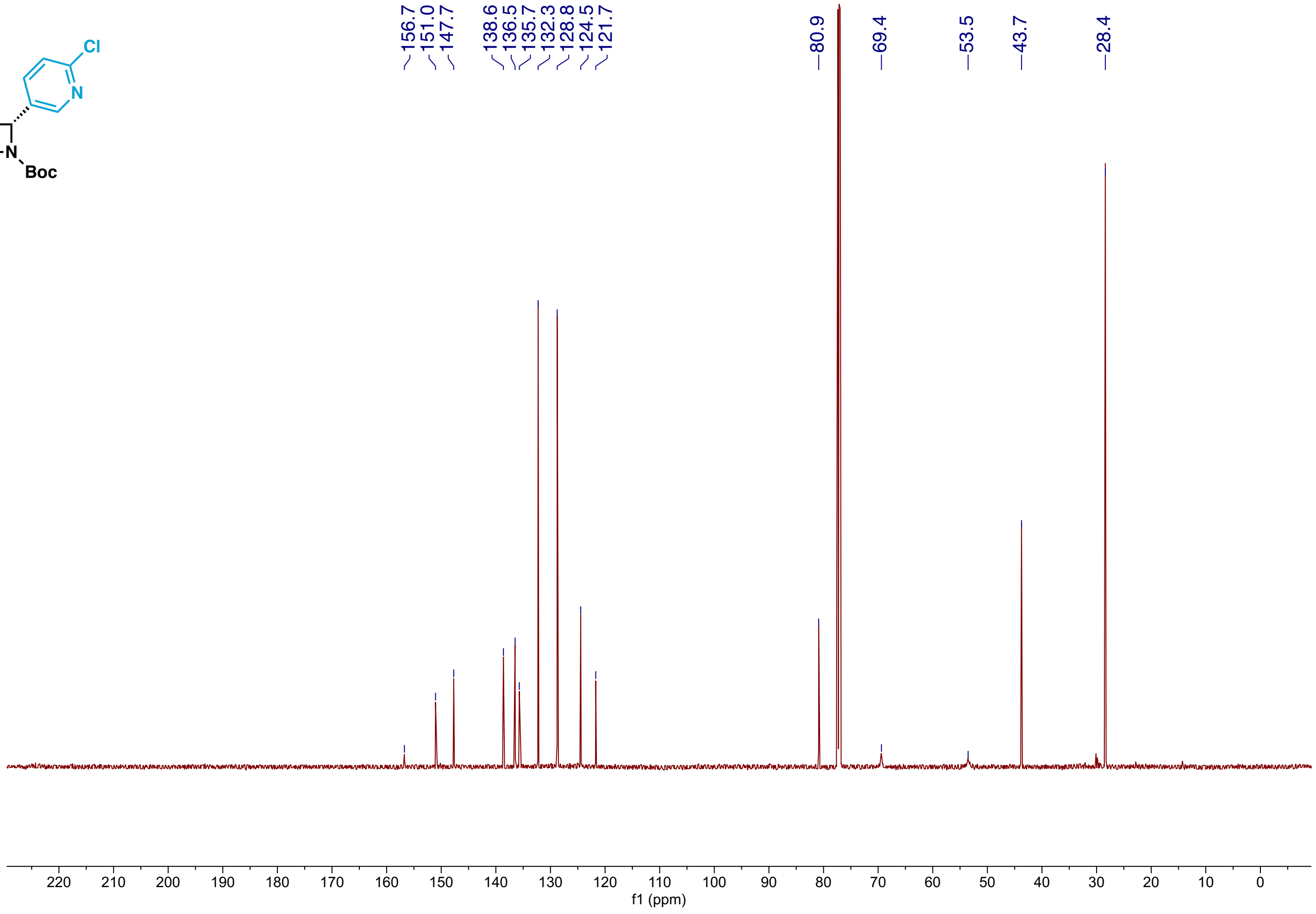

Compound 32 ^{1}H NMR

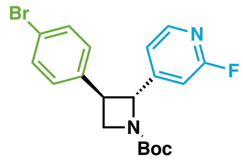

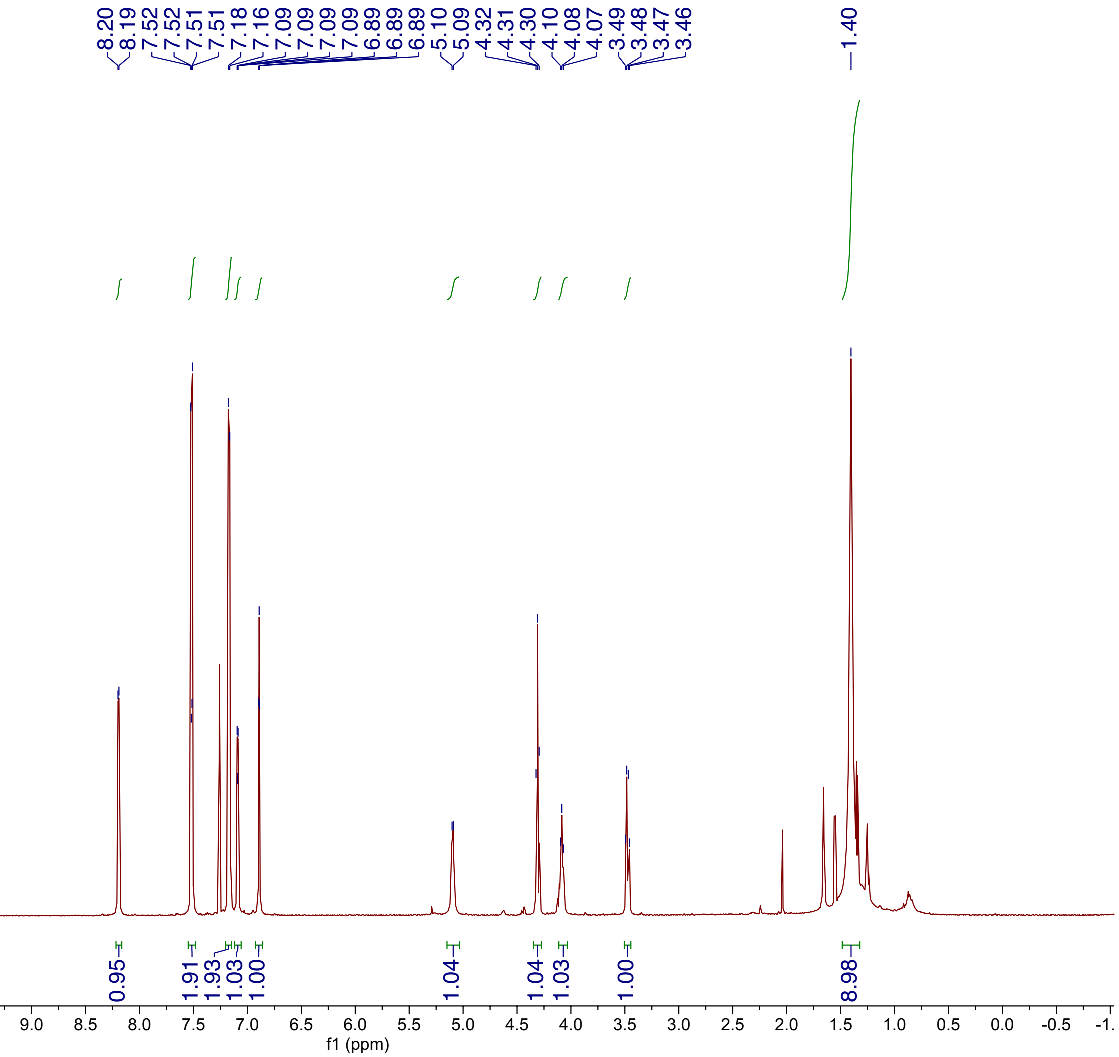

Compound 32 ^{19}F NMR

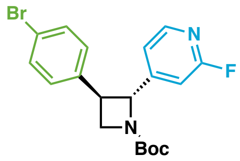

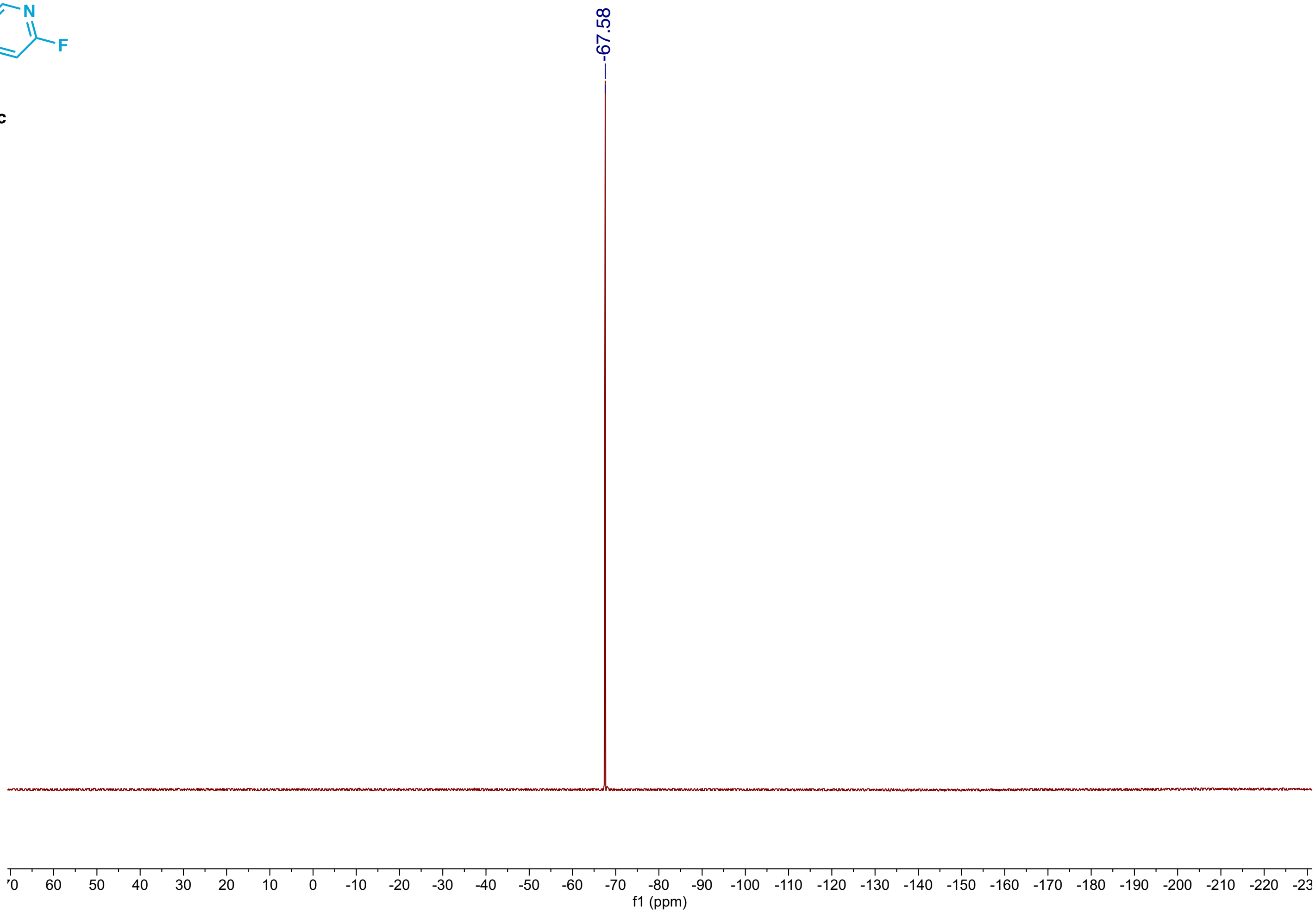

Compound 32 ^{13}C NMR

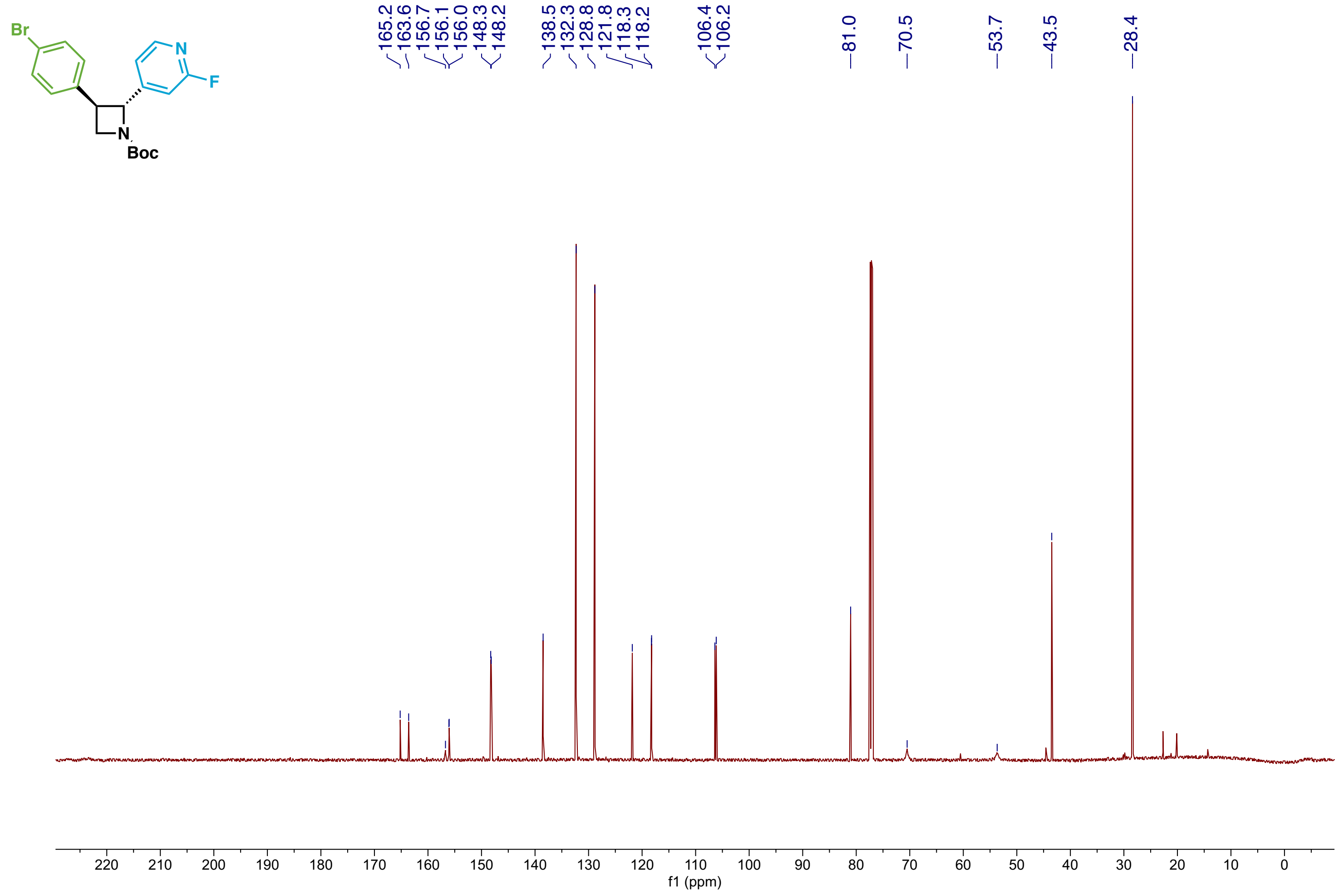

Compound B10 ^{1}H NMR

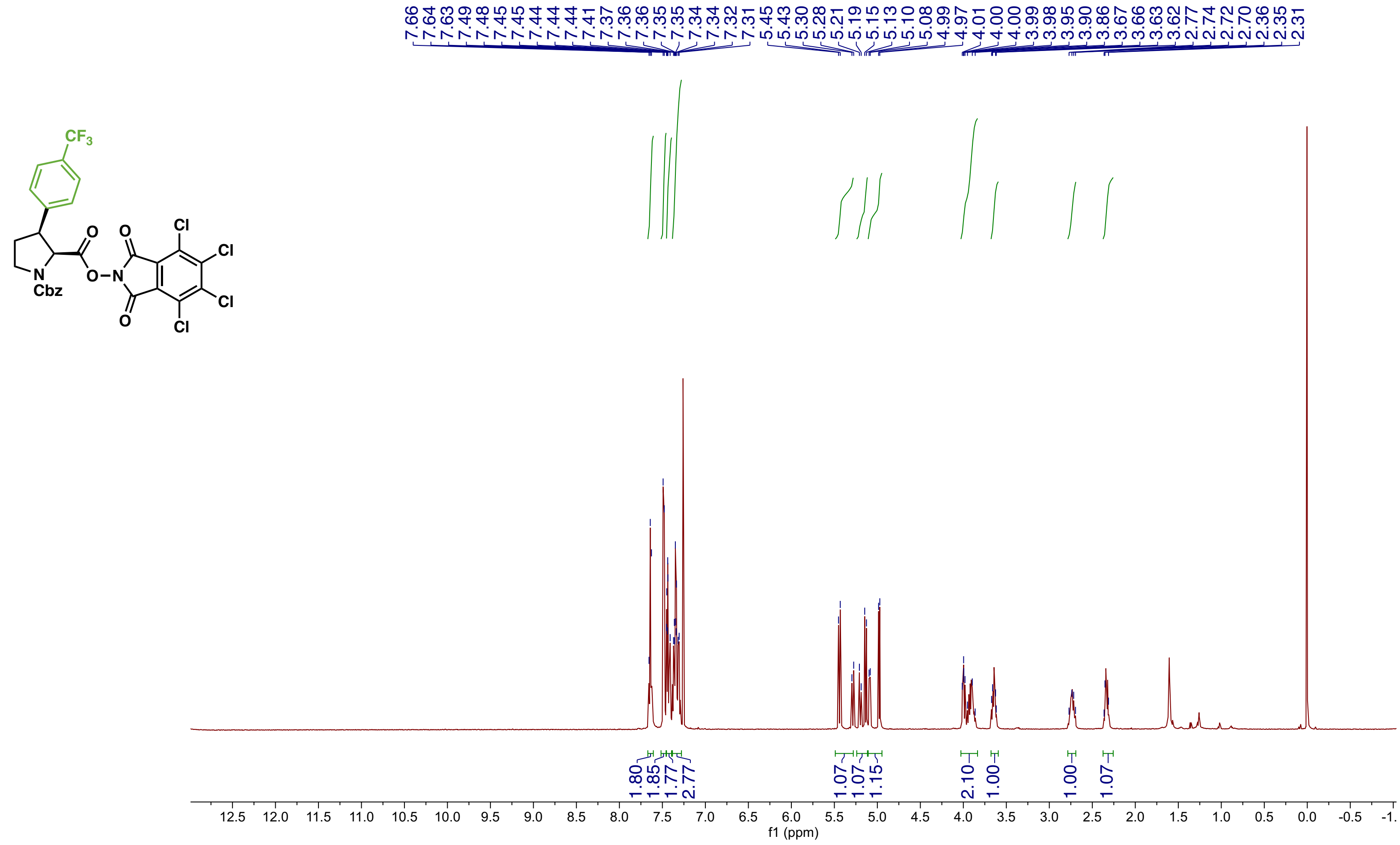

Compound B10 19F NMR

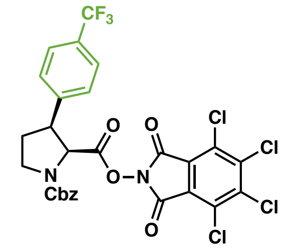

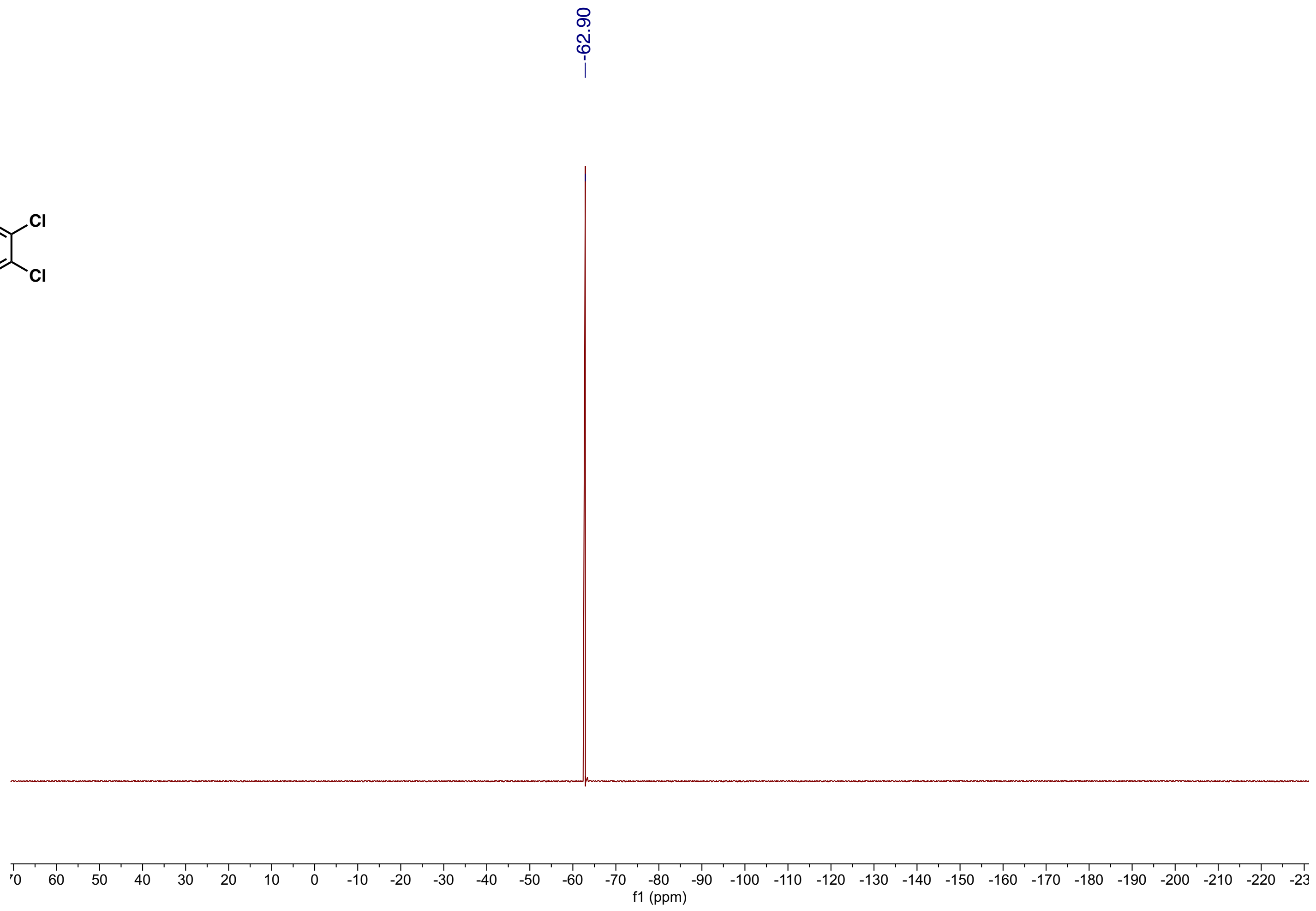

Compound B10 ^{13}C NMR

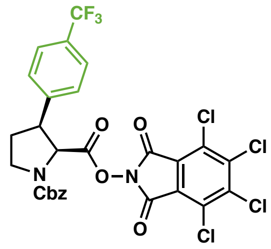

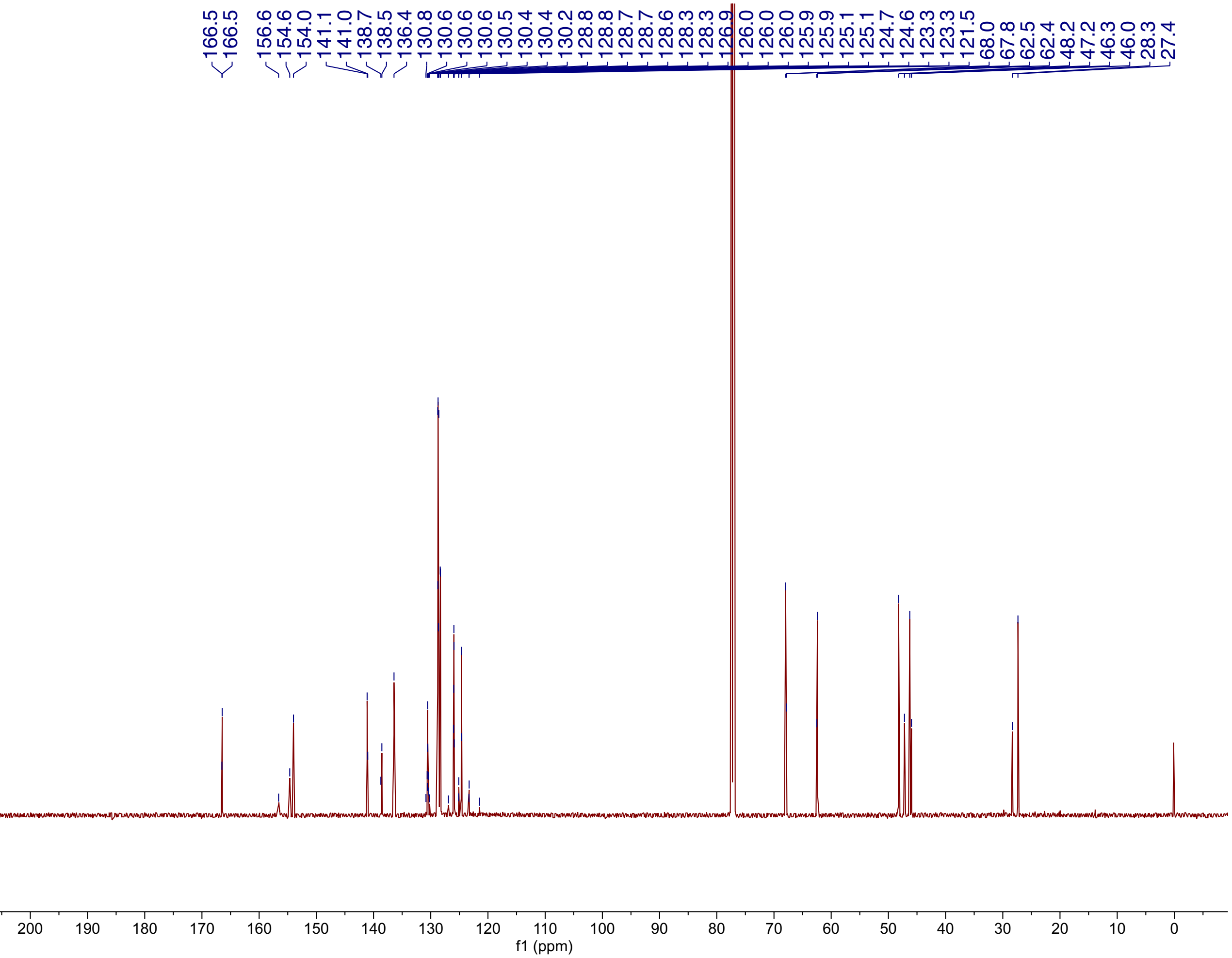

Compound 33 ^{1}H NMR

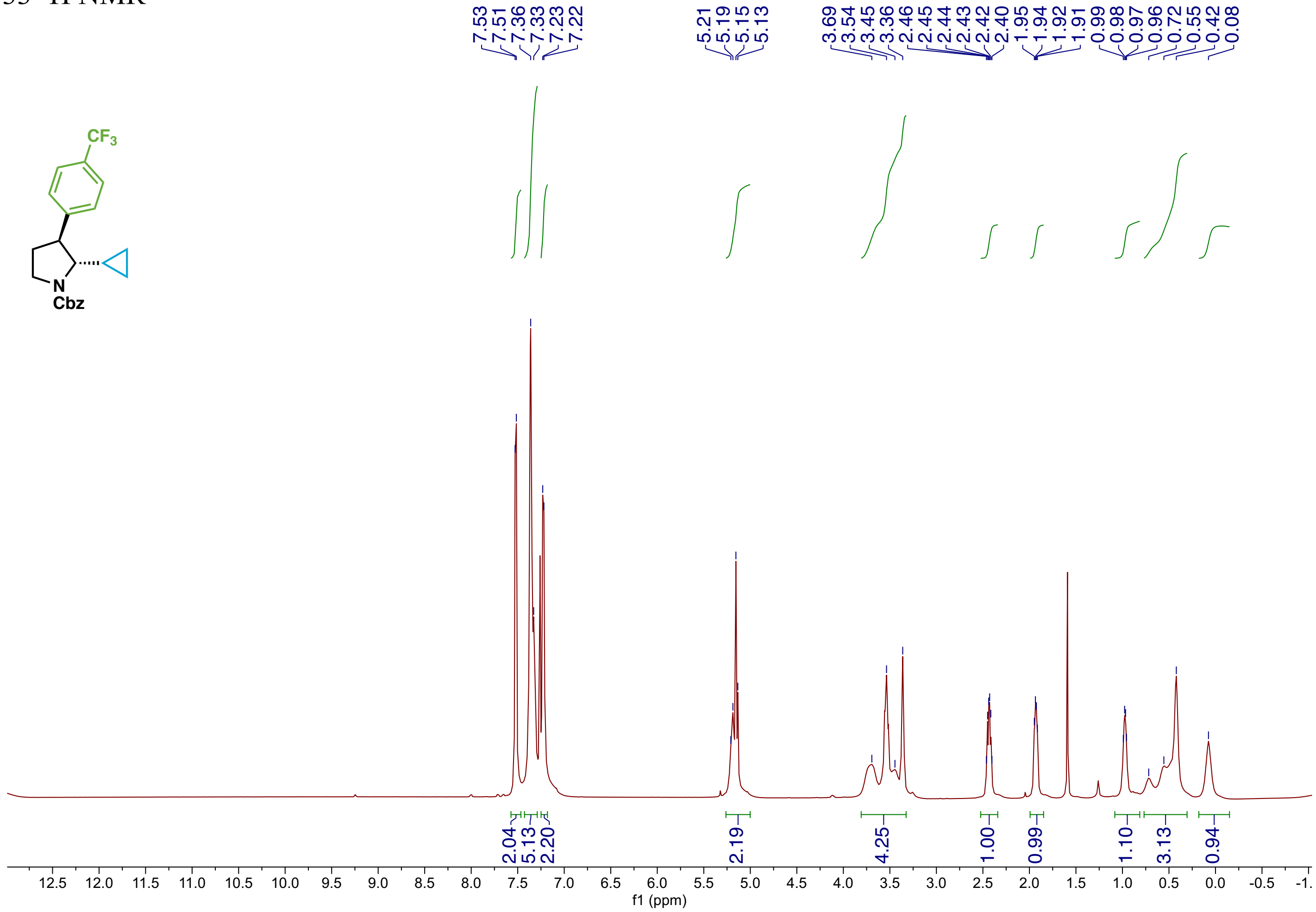

Compound 33 ^{19}F NMR

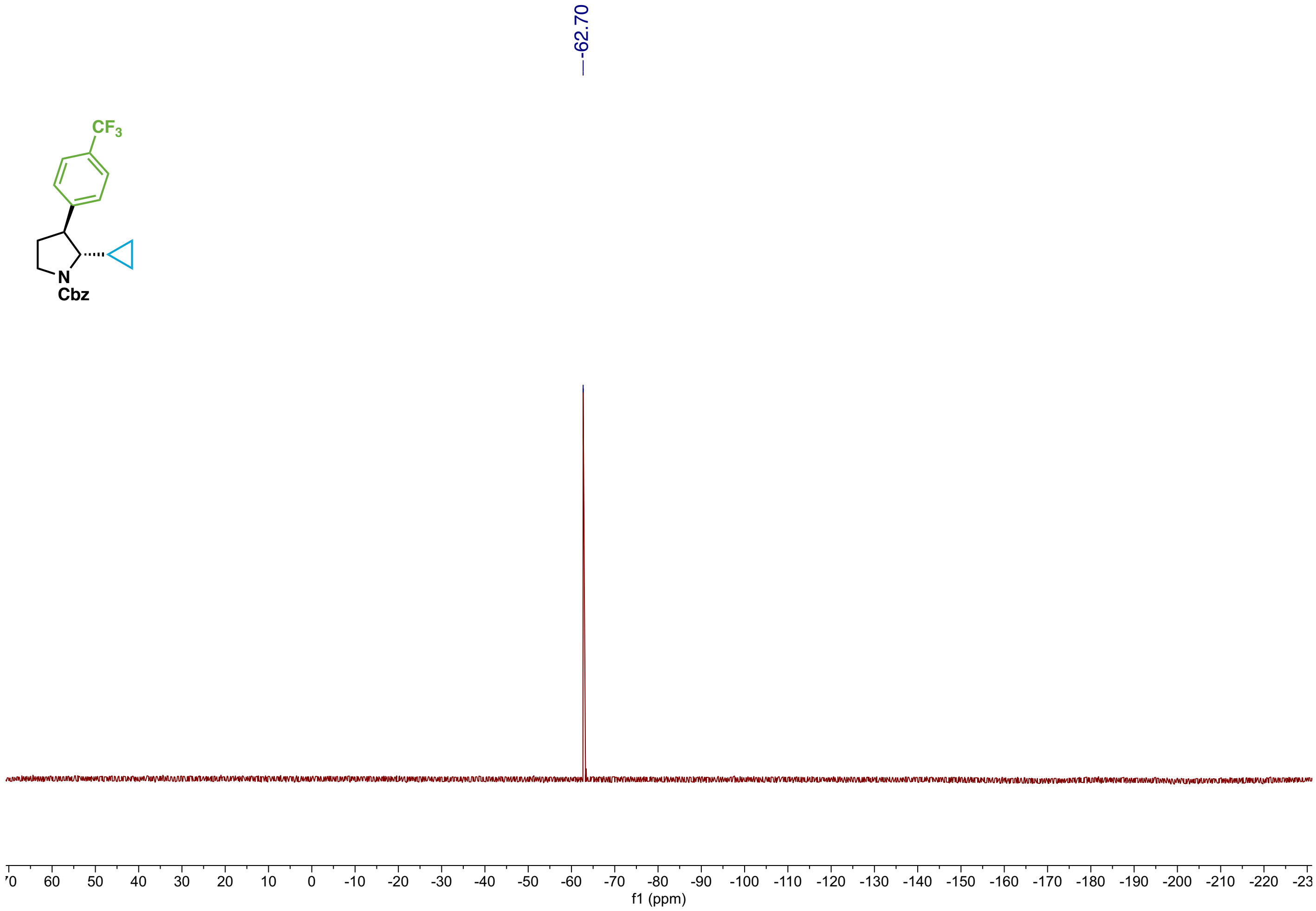

Compound 33 ^{13}C NMR

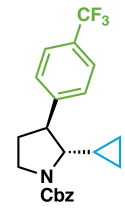

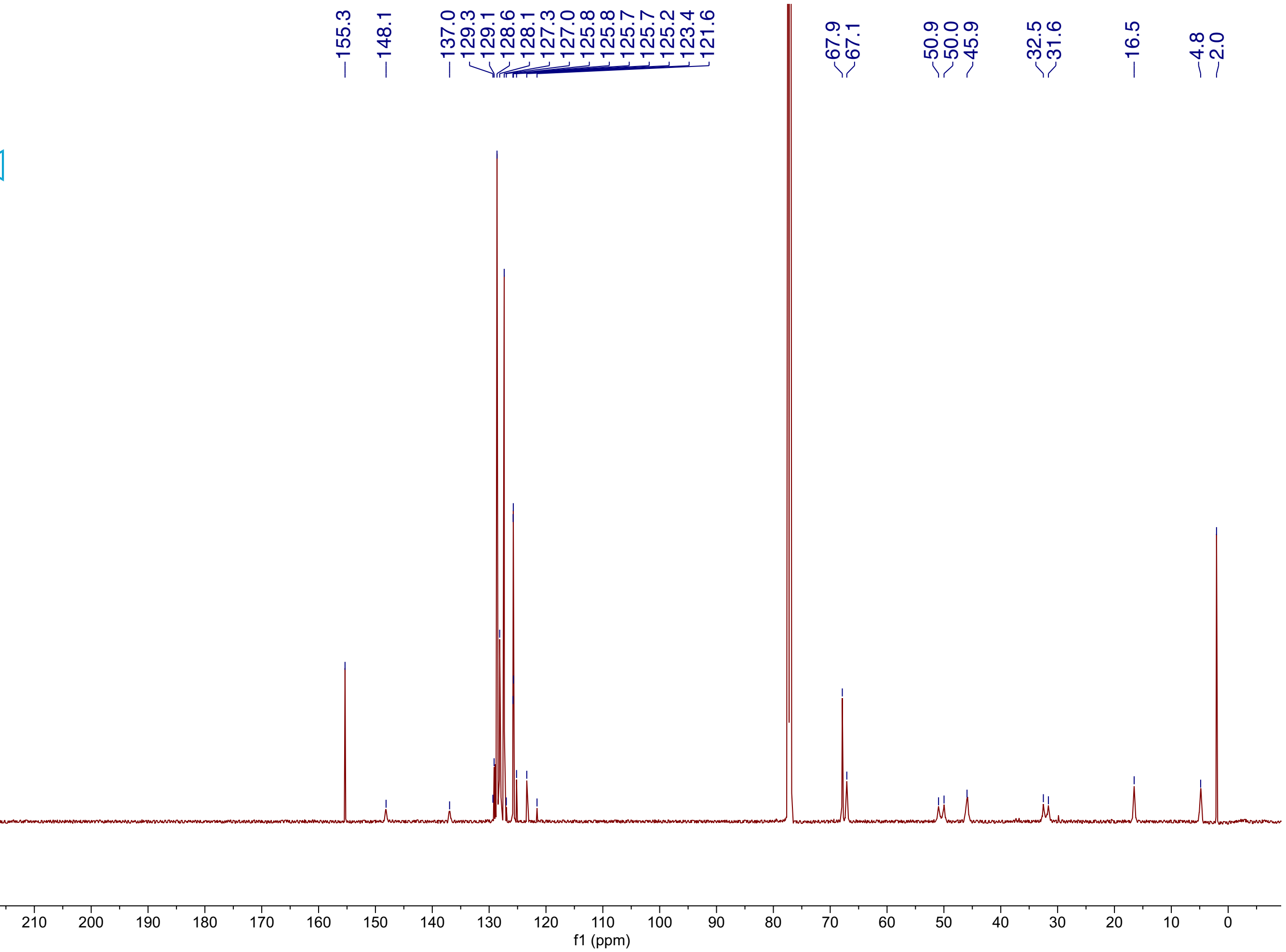

Compound 34 ^{1}H NMR

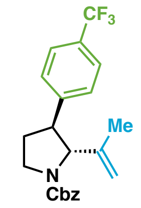

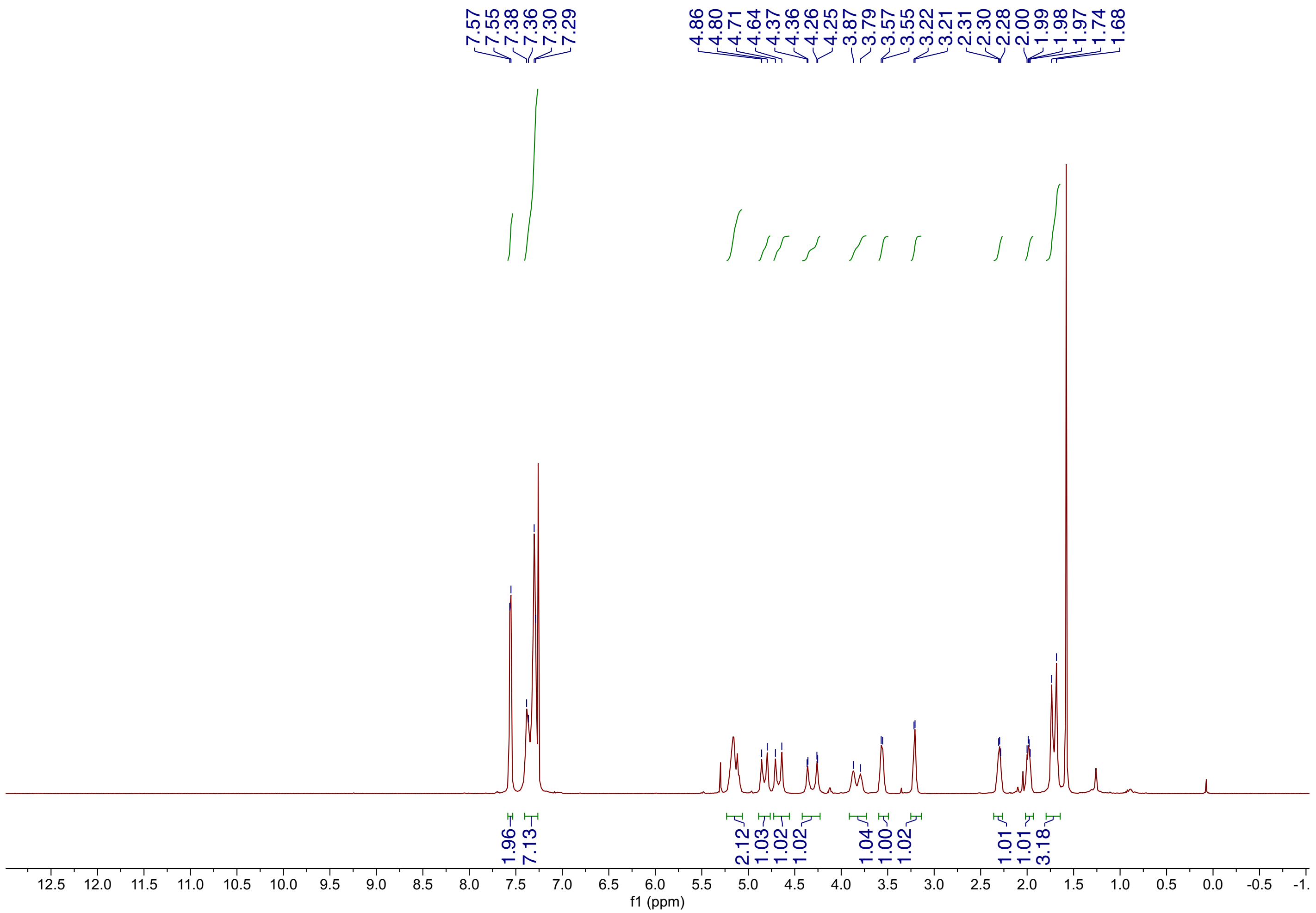

Compound 34 ^{19}F NMR

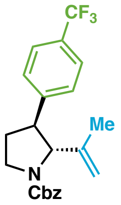

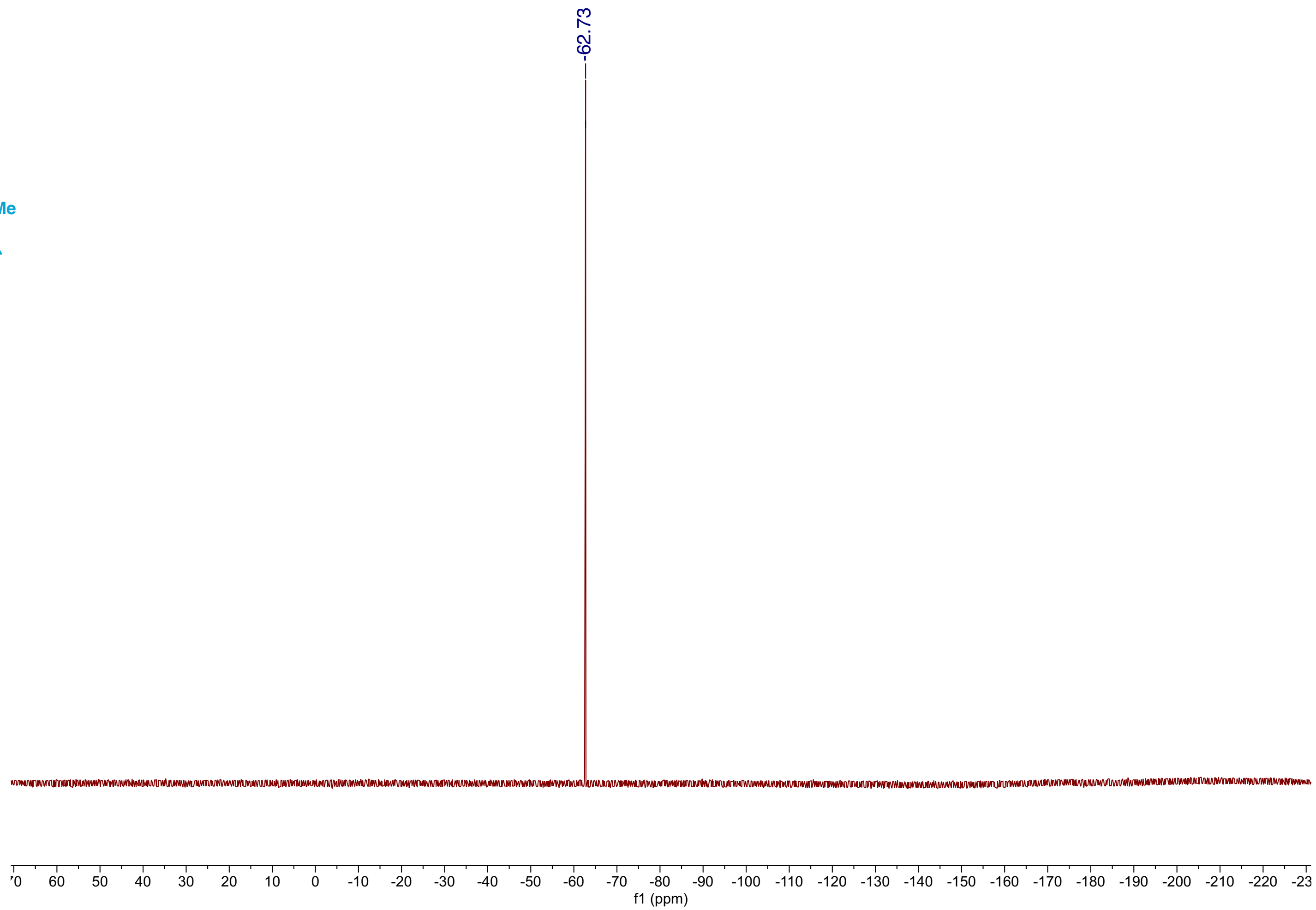

Compound 34 ^{13}C NMR

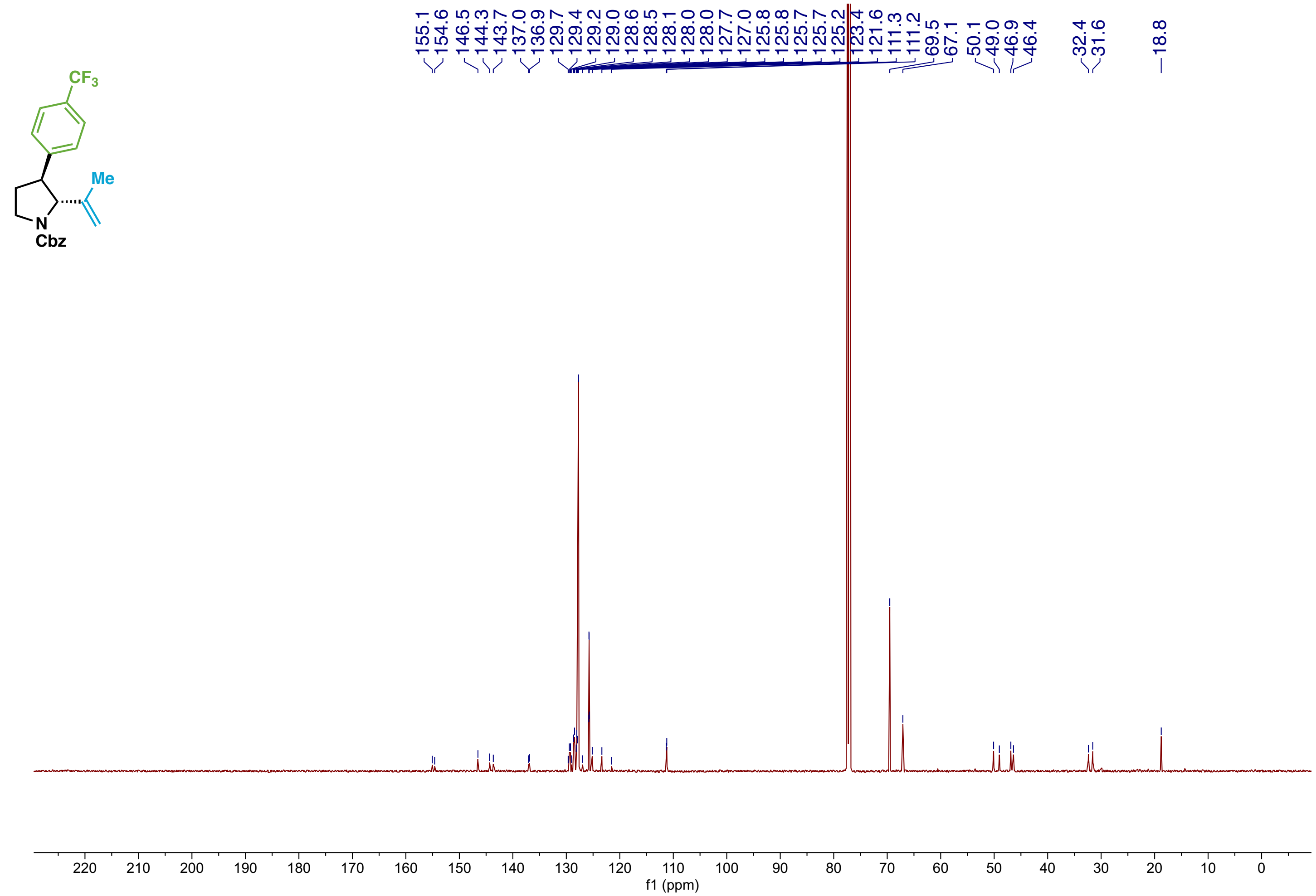

Compound 35 ^{1}H NMR

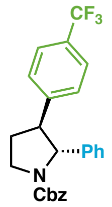

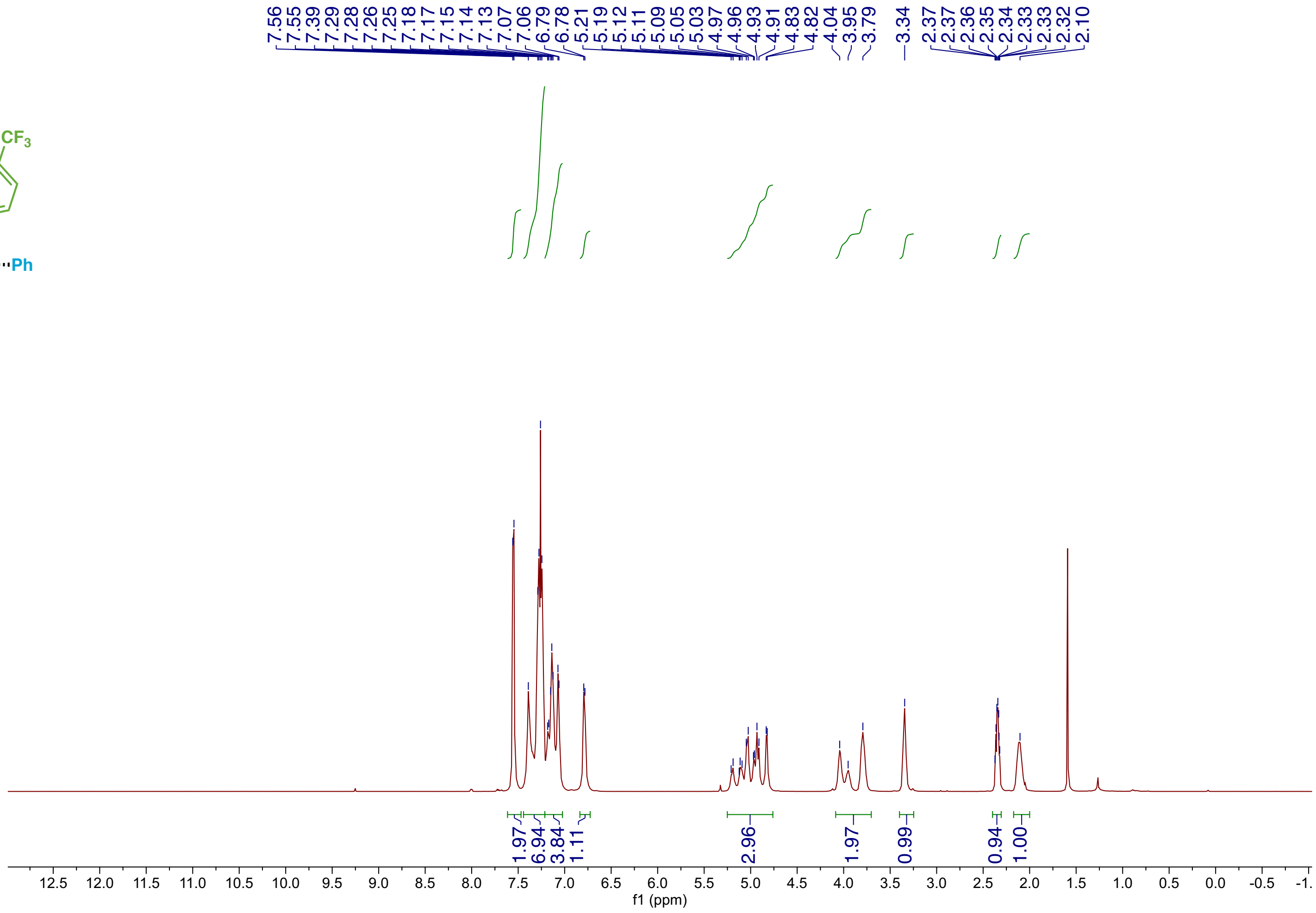

Compound 35 ^{19}F NMR

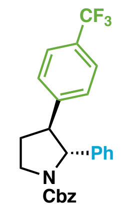

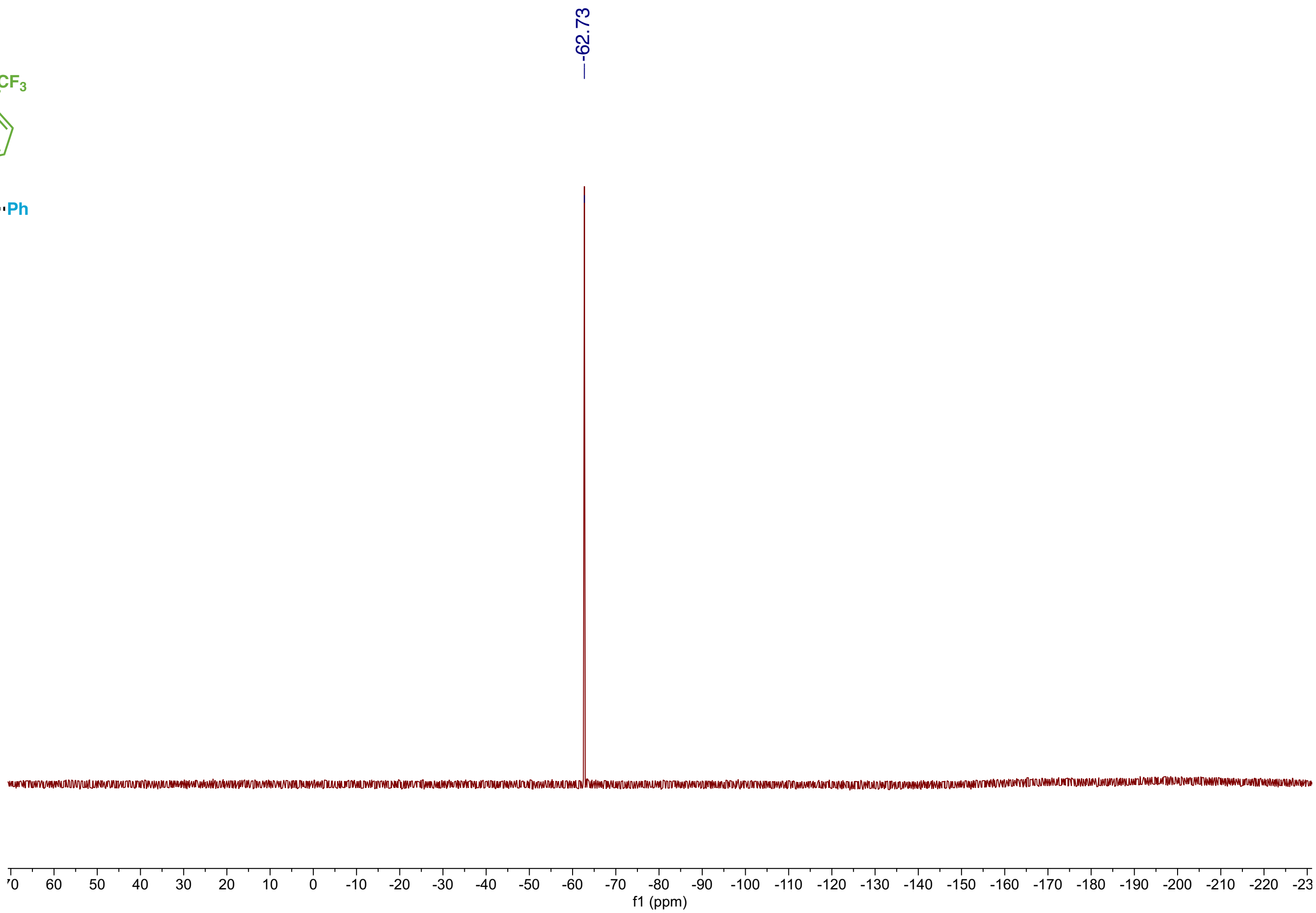

Compound 35 ^{13}C NMR

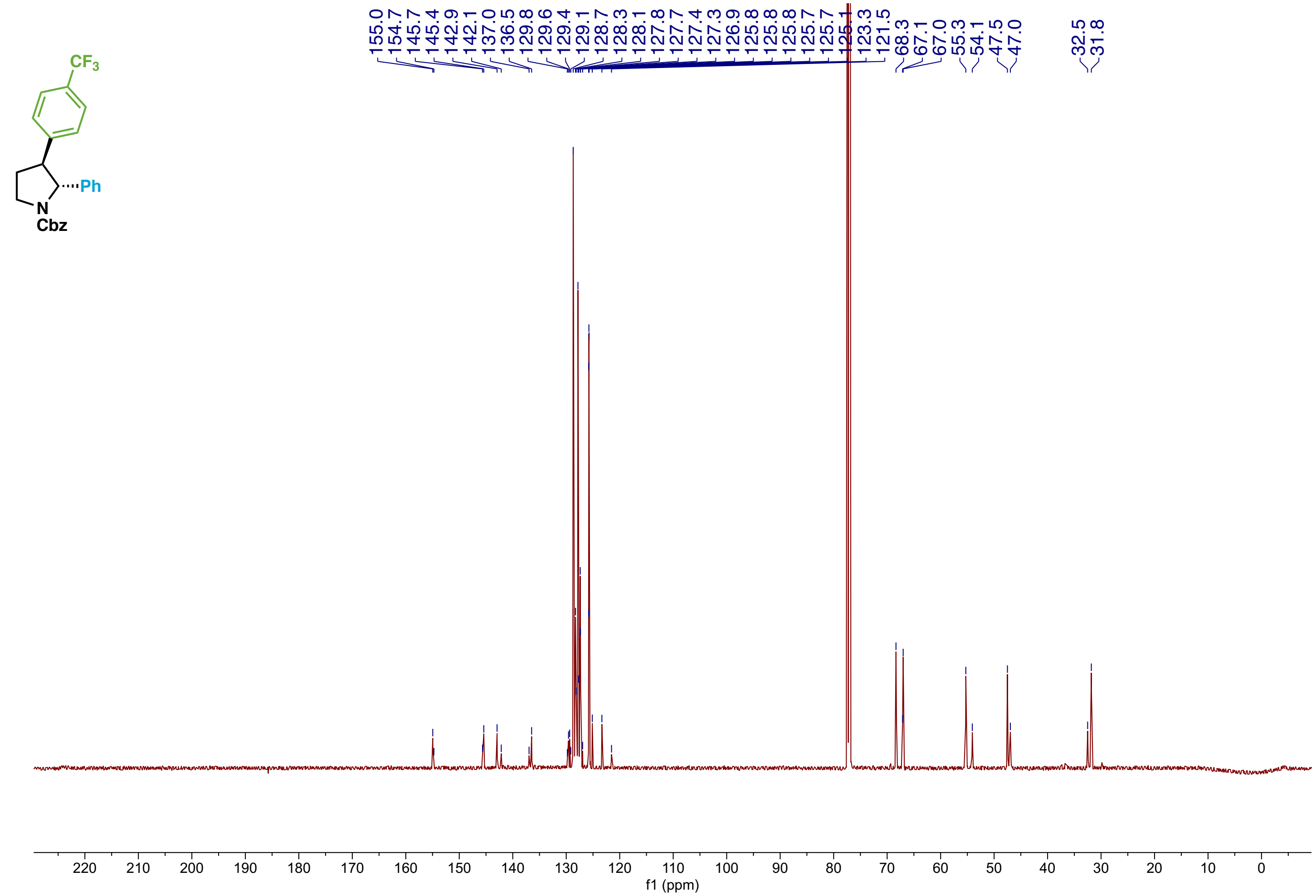

Compound 36 ^{1}H NMR

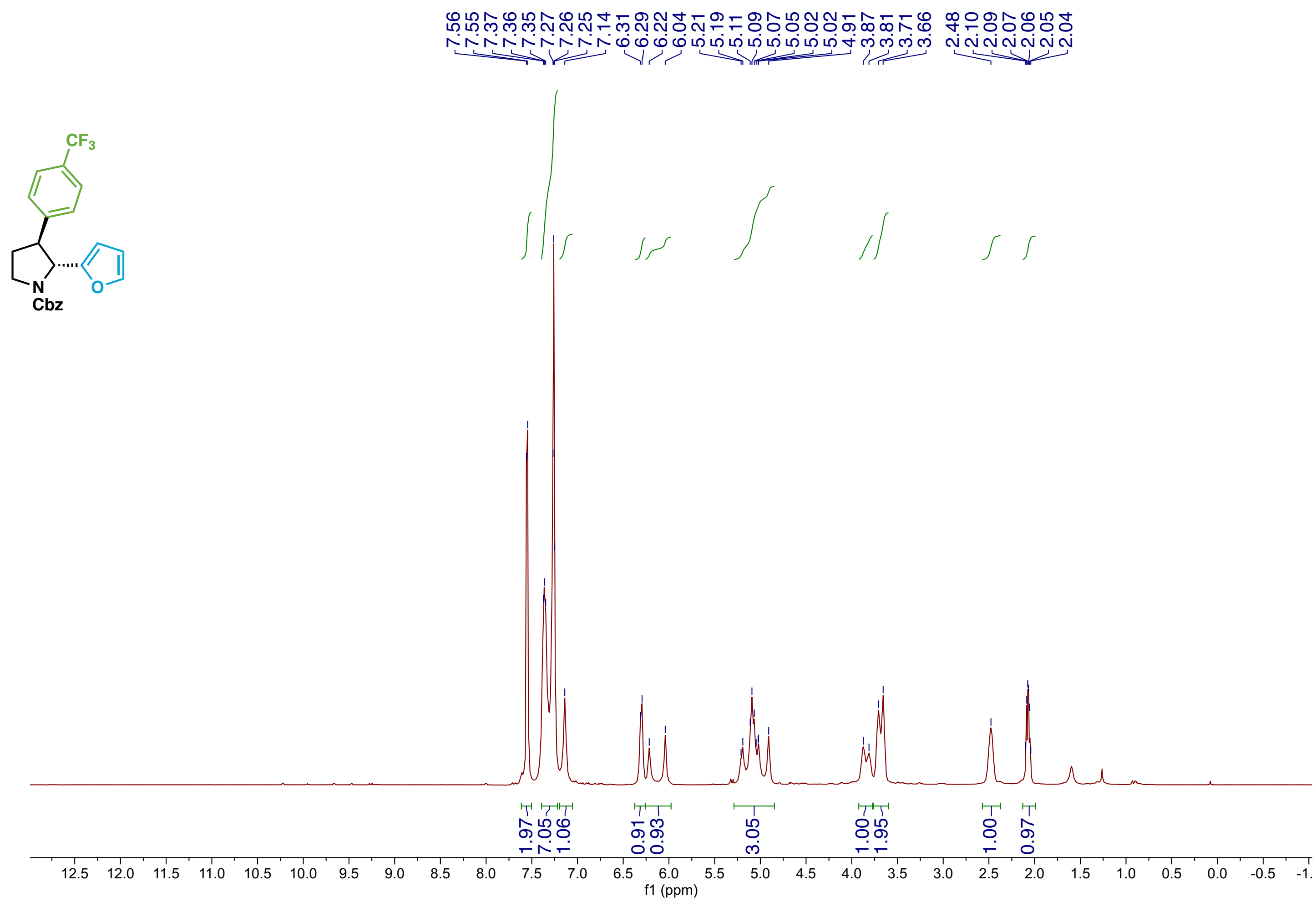

Compound 36 ^{19}F NMR

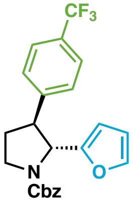

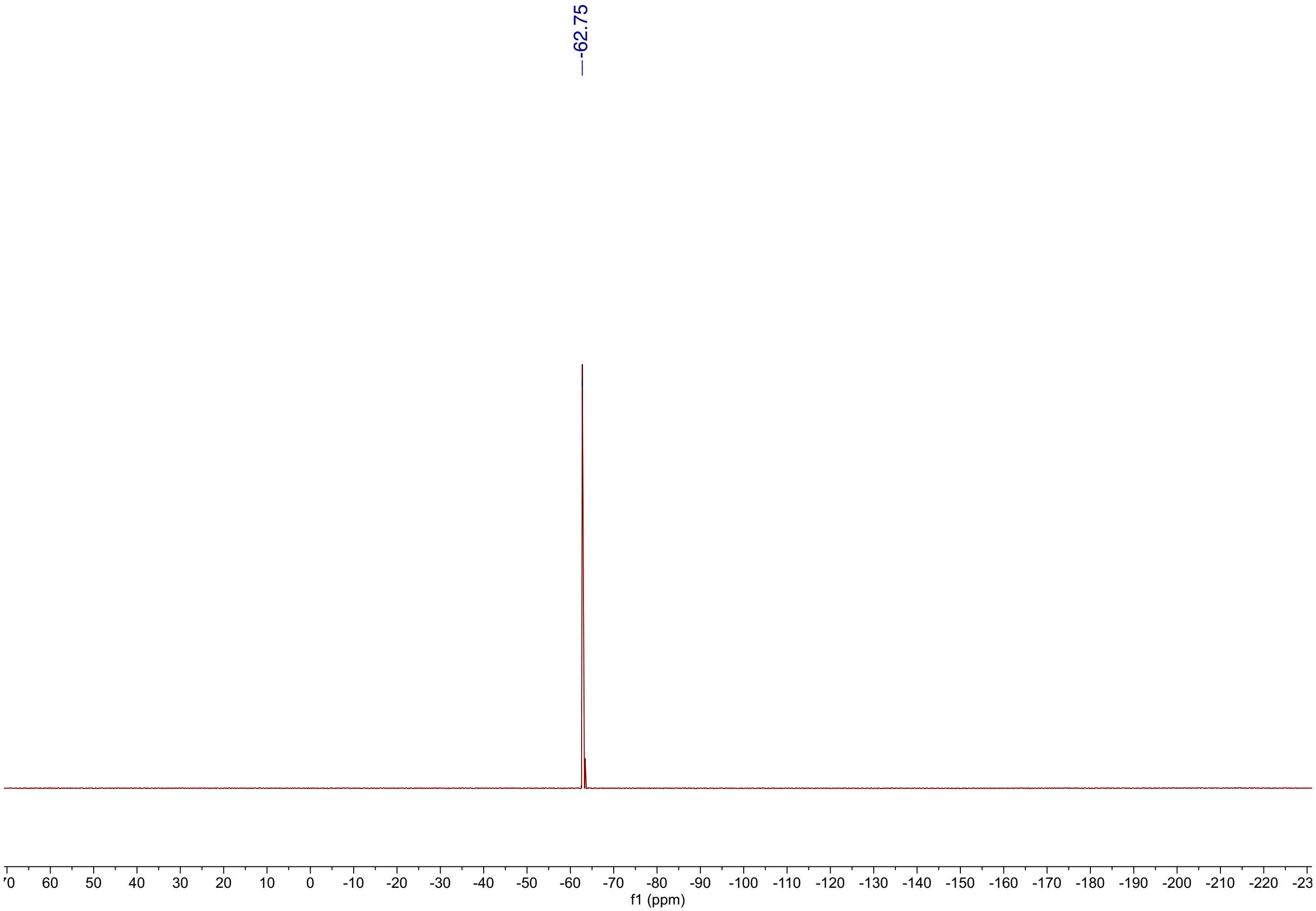

Compound 36 ^{13}C NMR

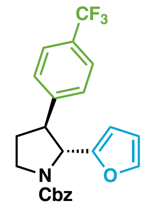

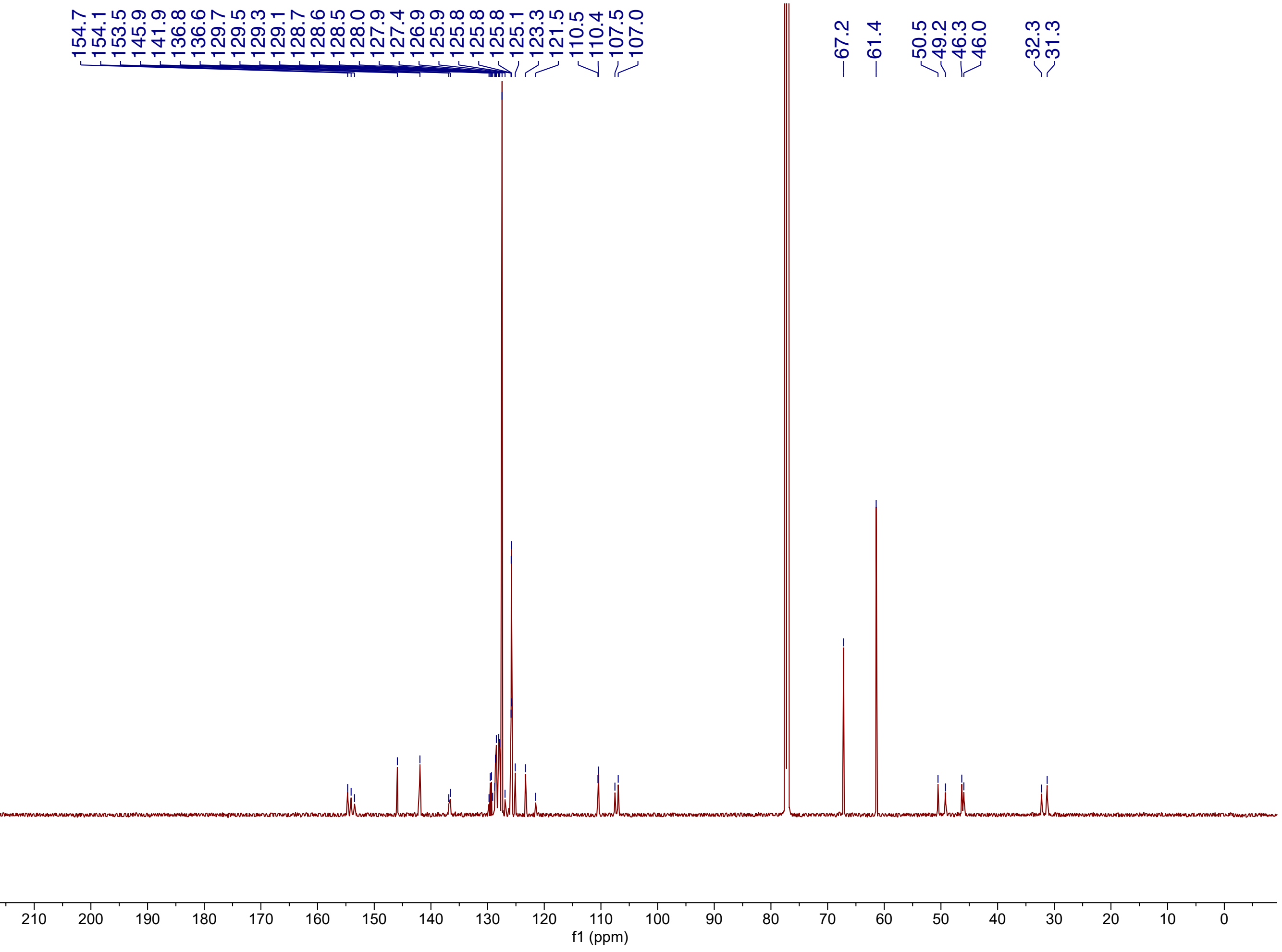

Compound 37 ^{1}H NMR

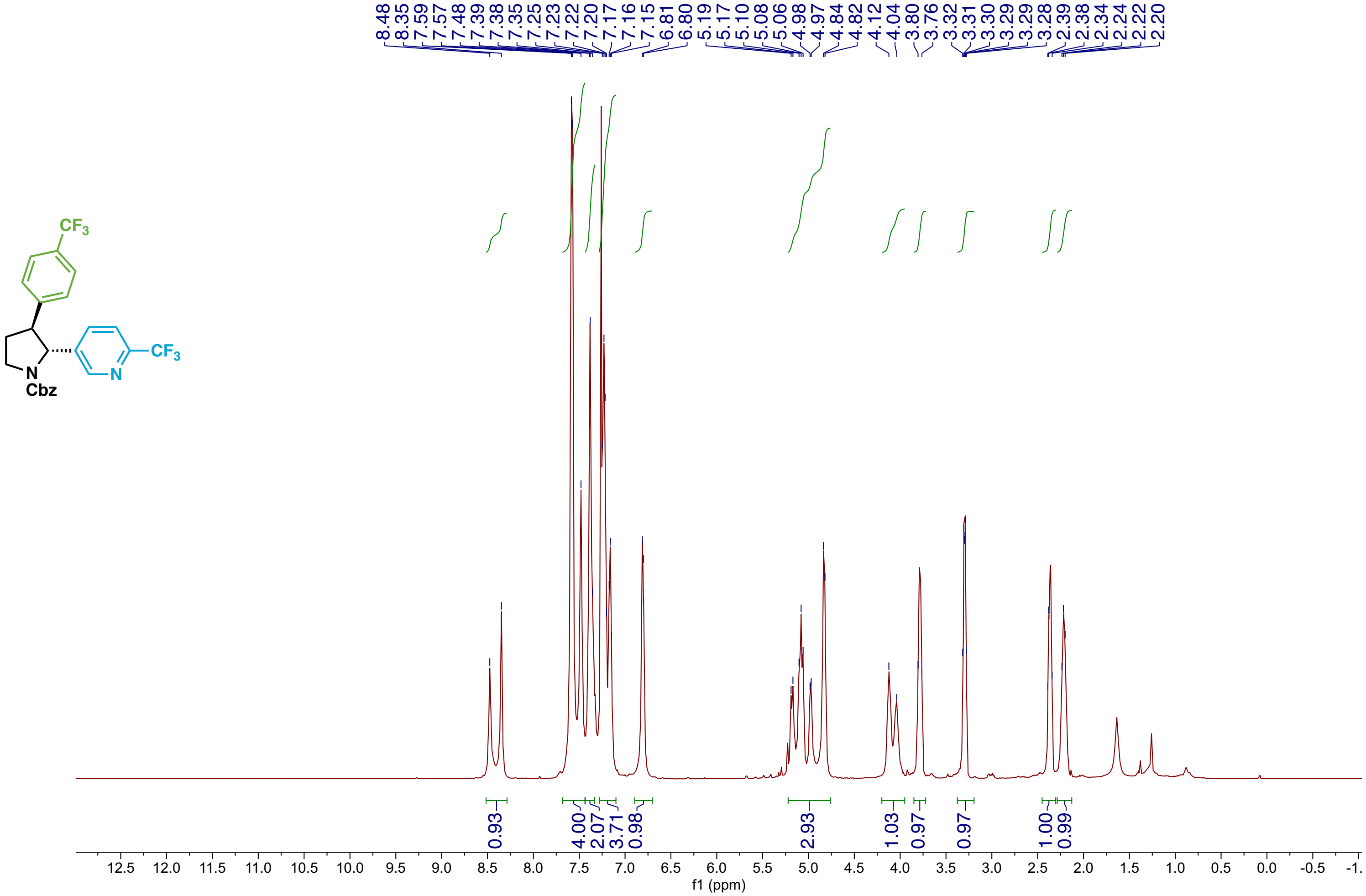

Compound 37 ^{19}F NMR

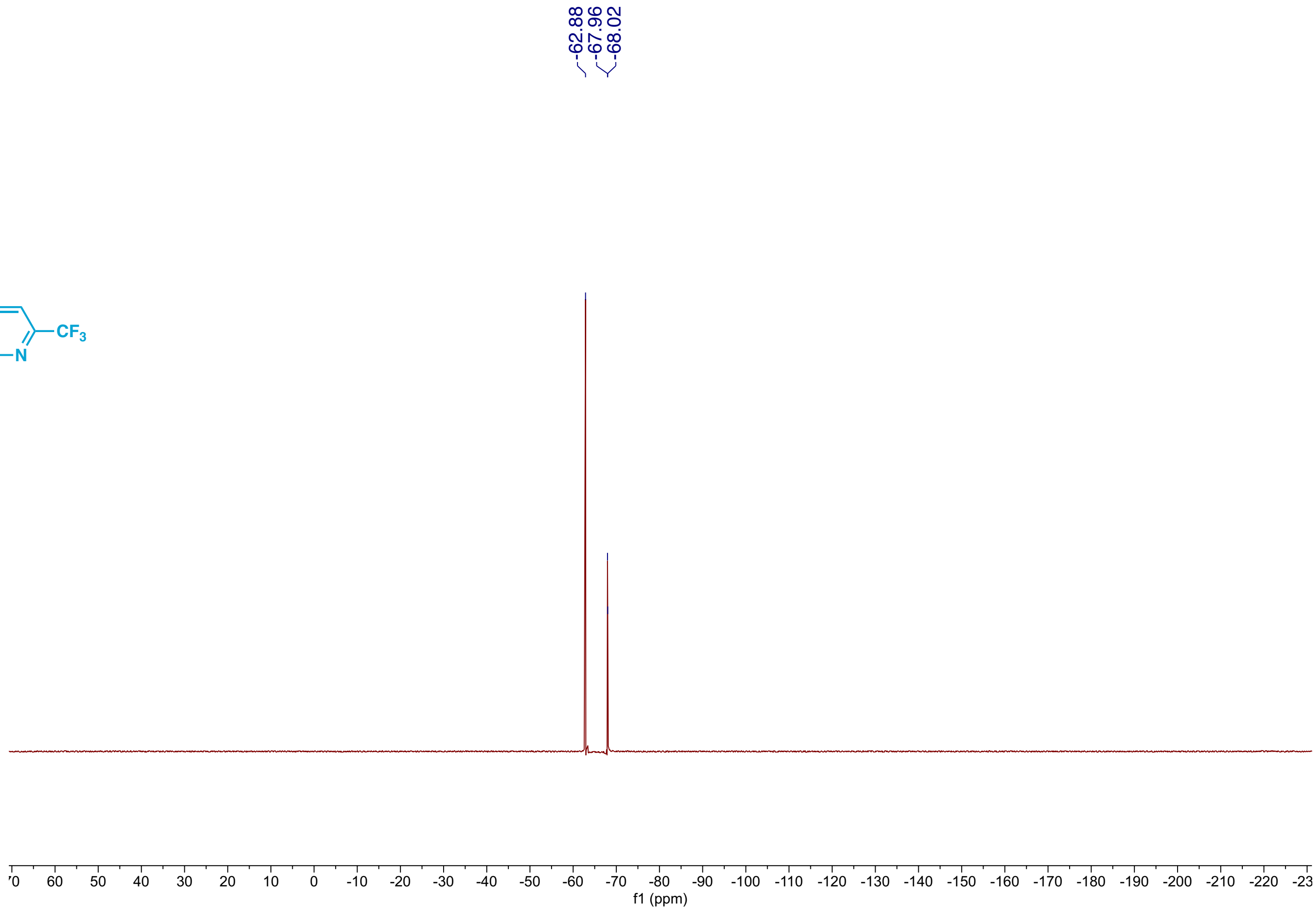

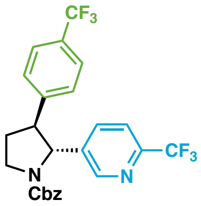

Compound 37 ^{13}C NMR

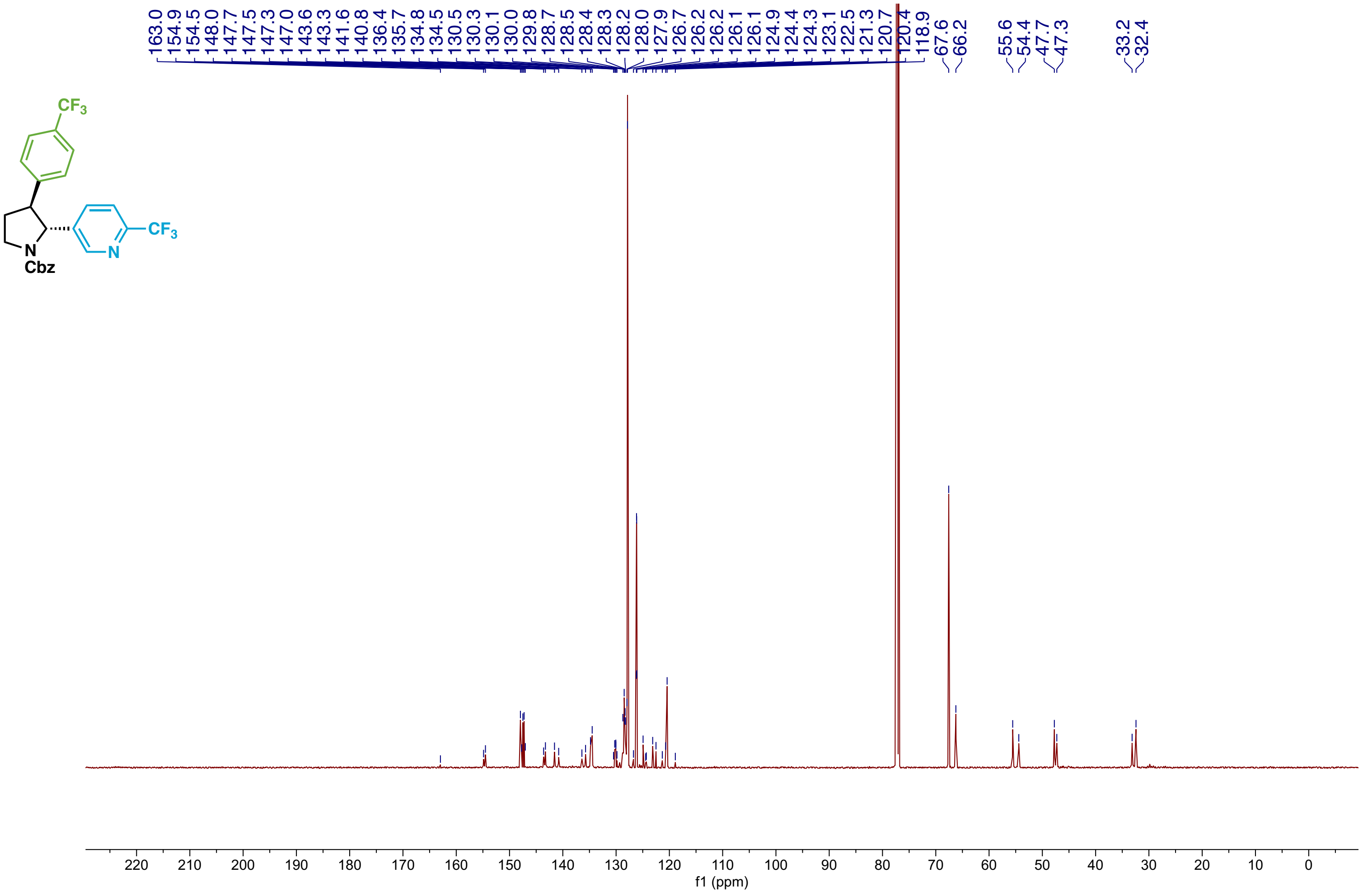

Compound 38 ^{1}H NMR

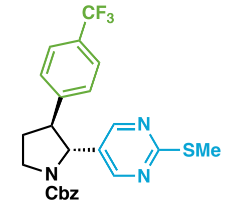

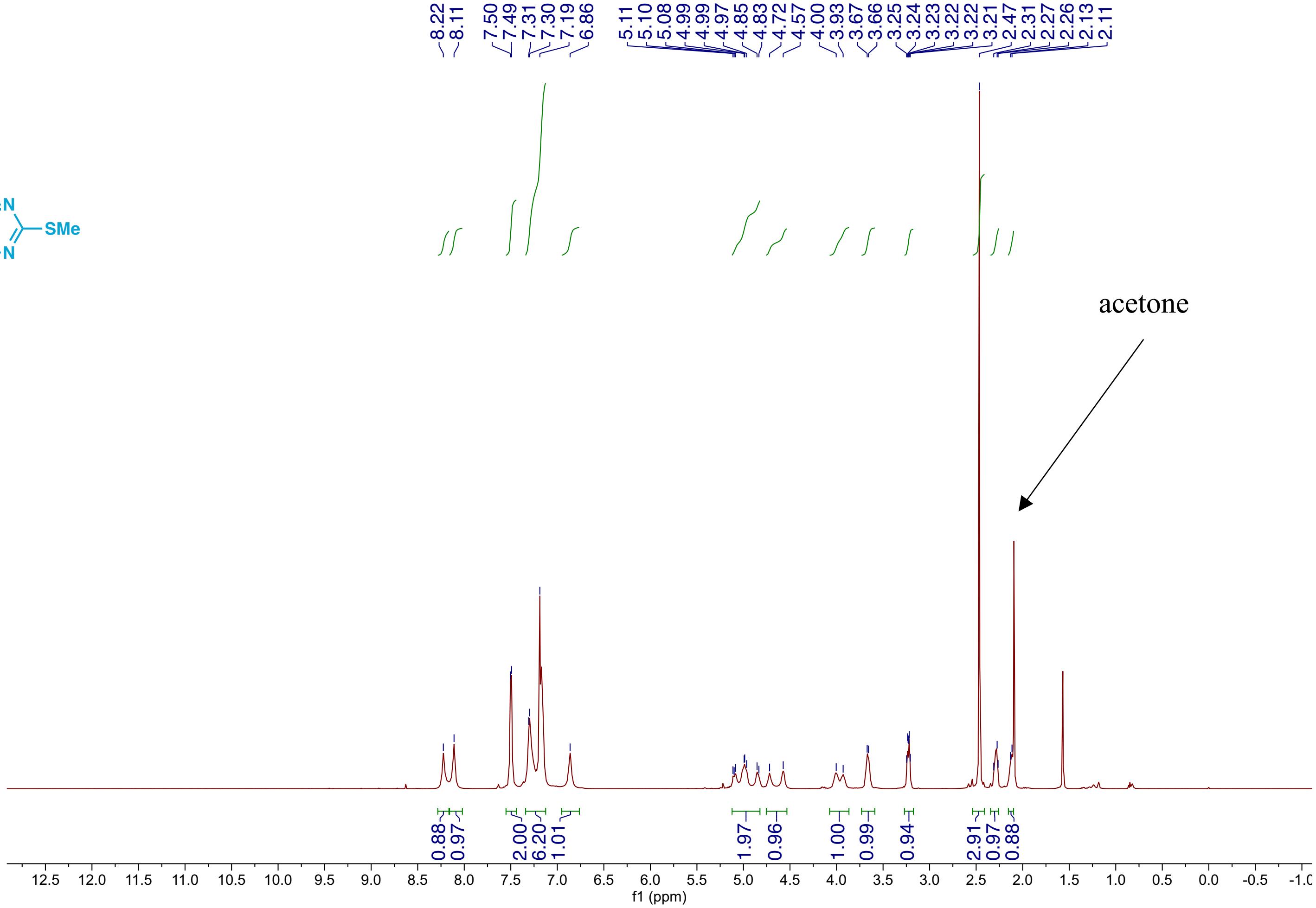

Compound 38 ^{19}F NMR

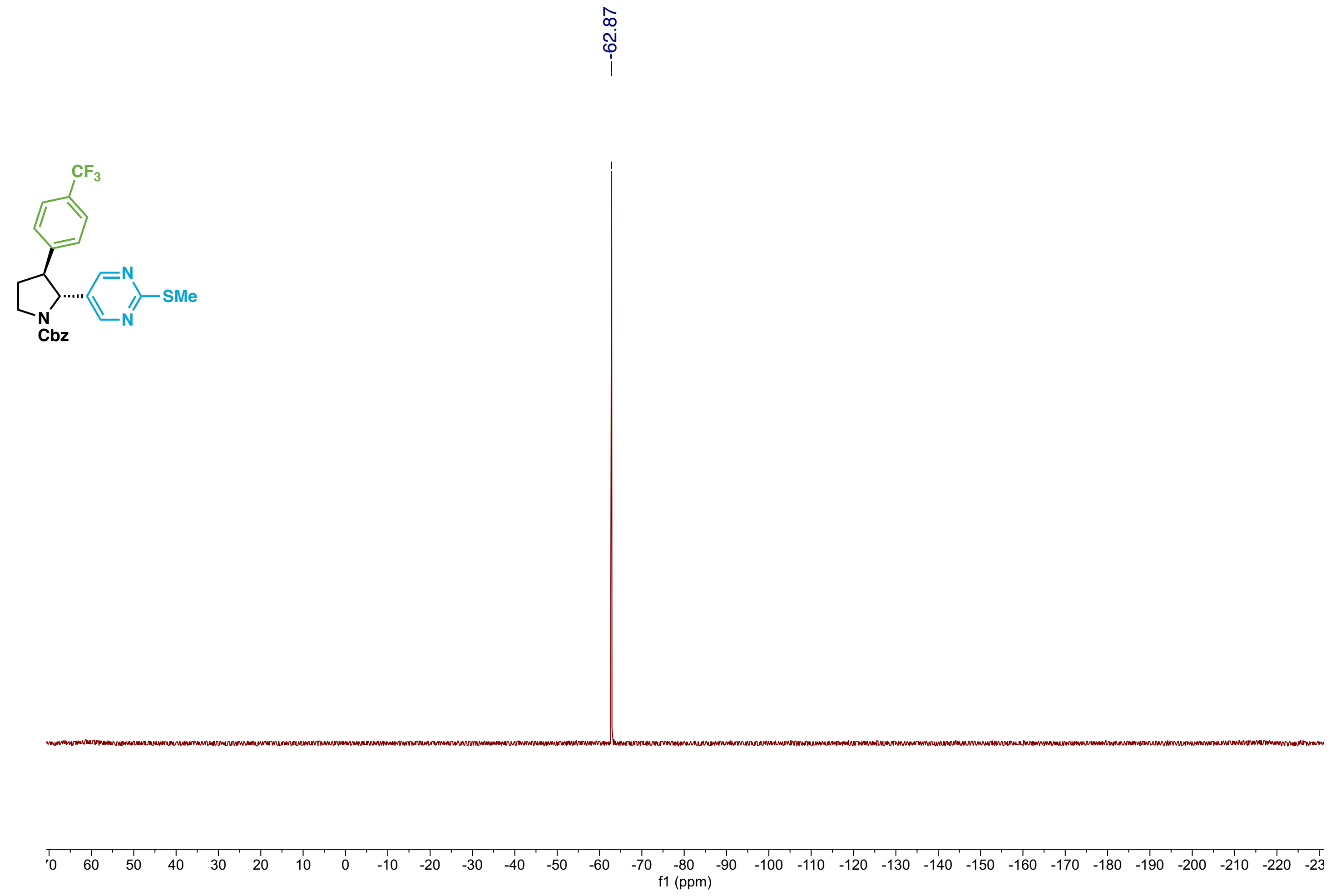

Compound 38 ^{13}C NMR

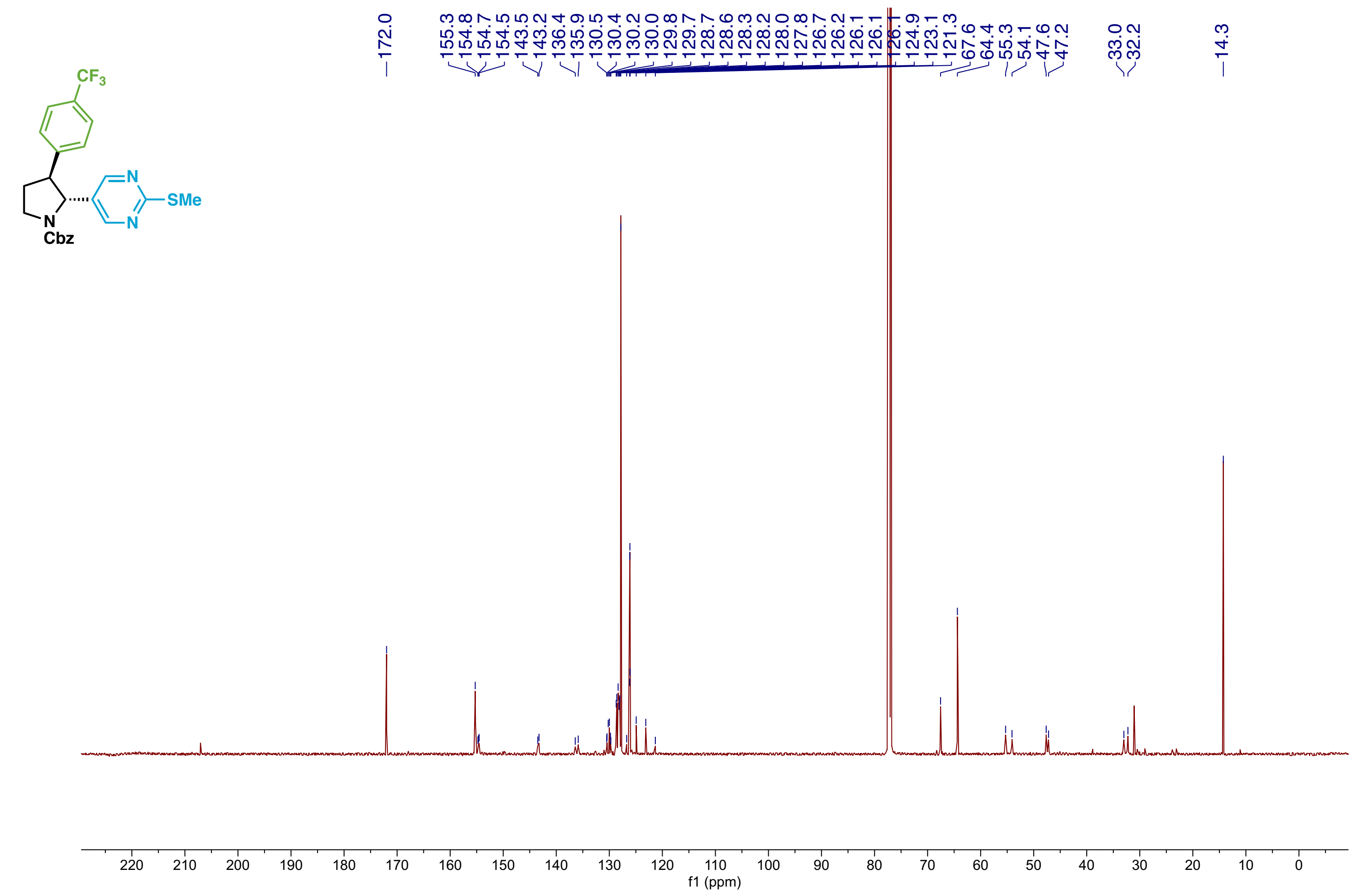

Compound 39 ^{1}H NMR

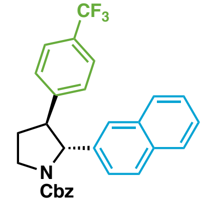

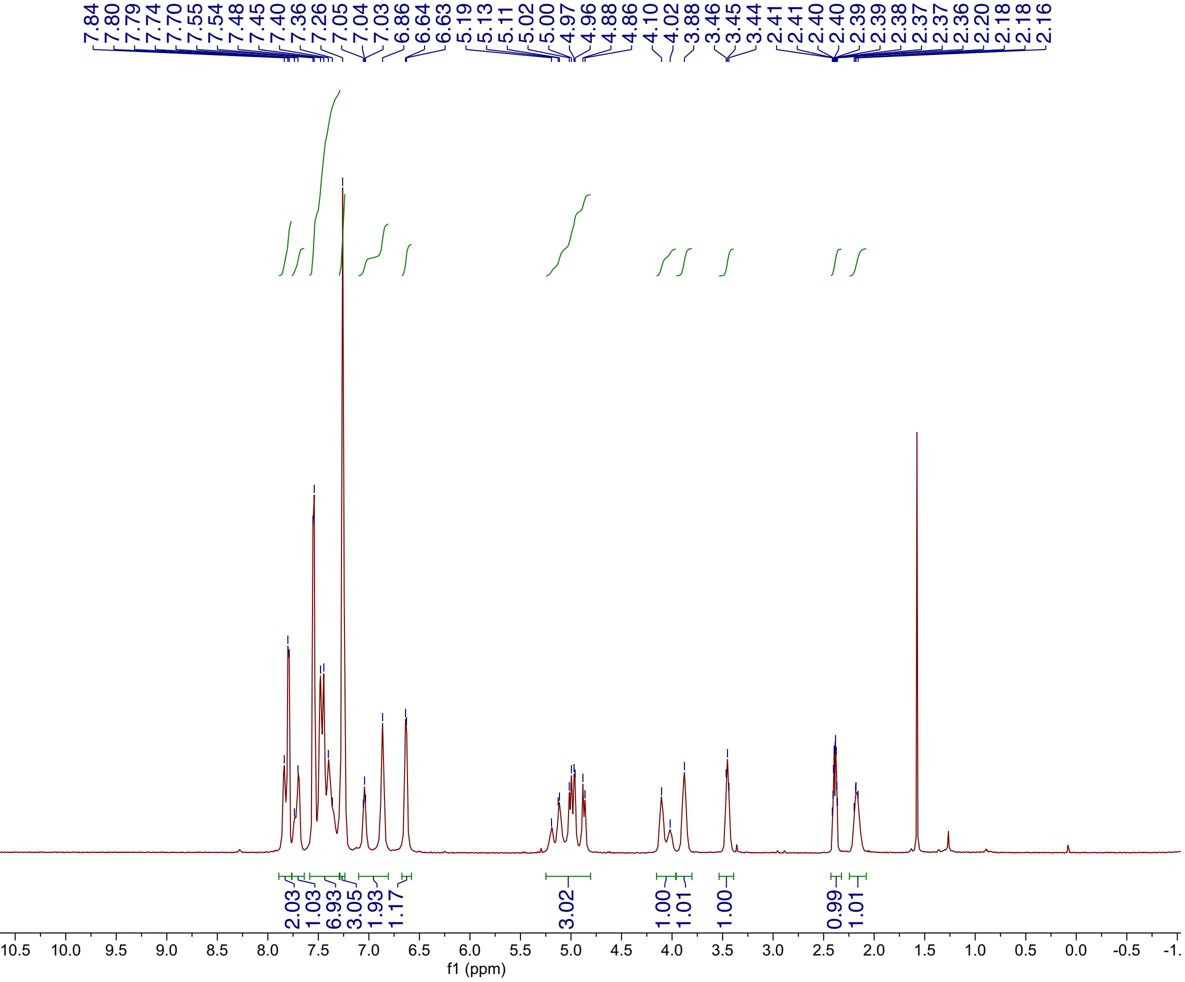

Compound 39 ^{19}F NMR

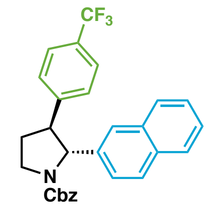

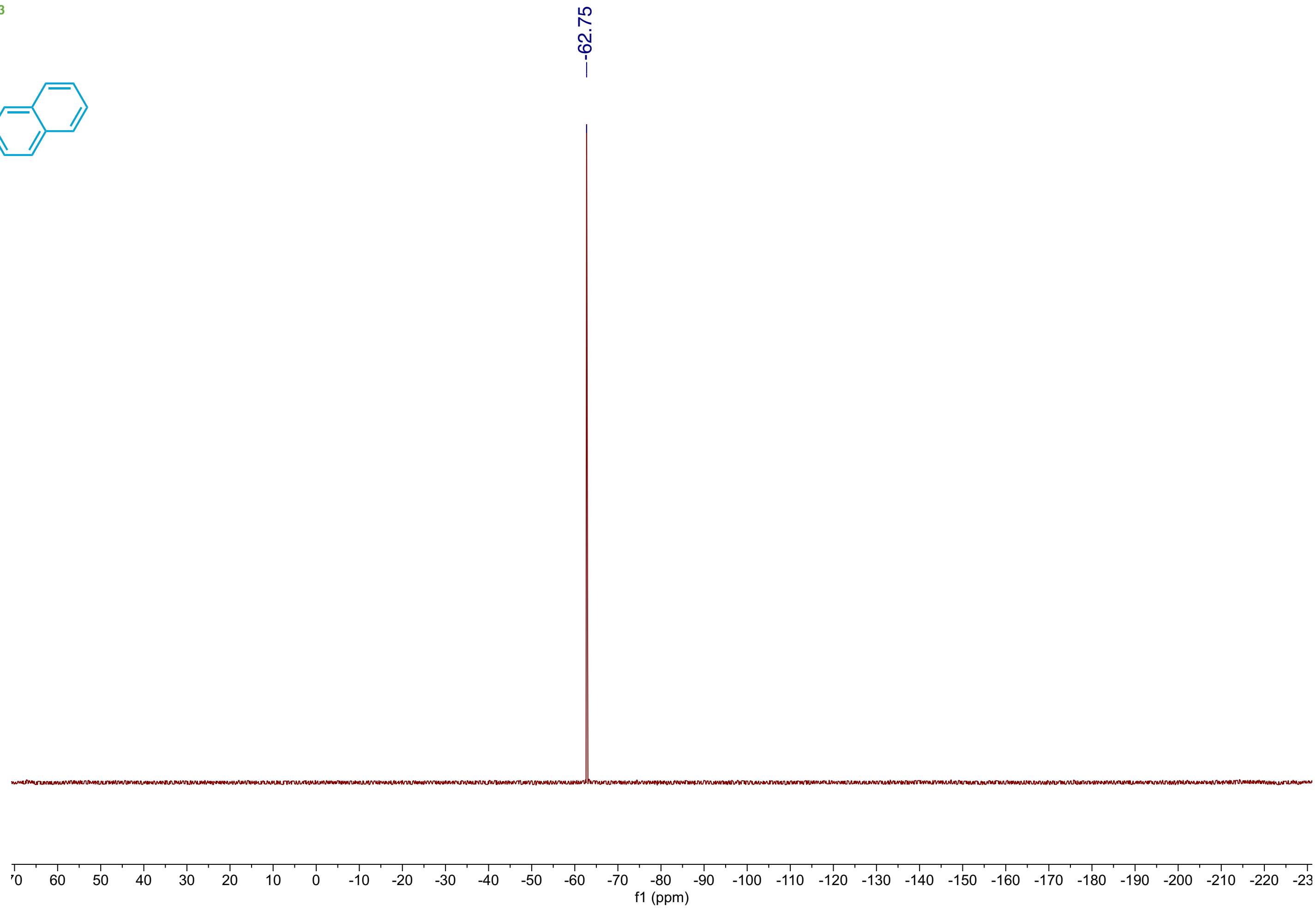

Compound 39 ^{13}C NMR

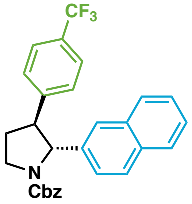

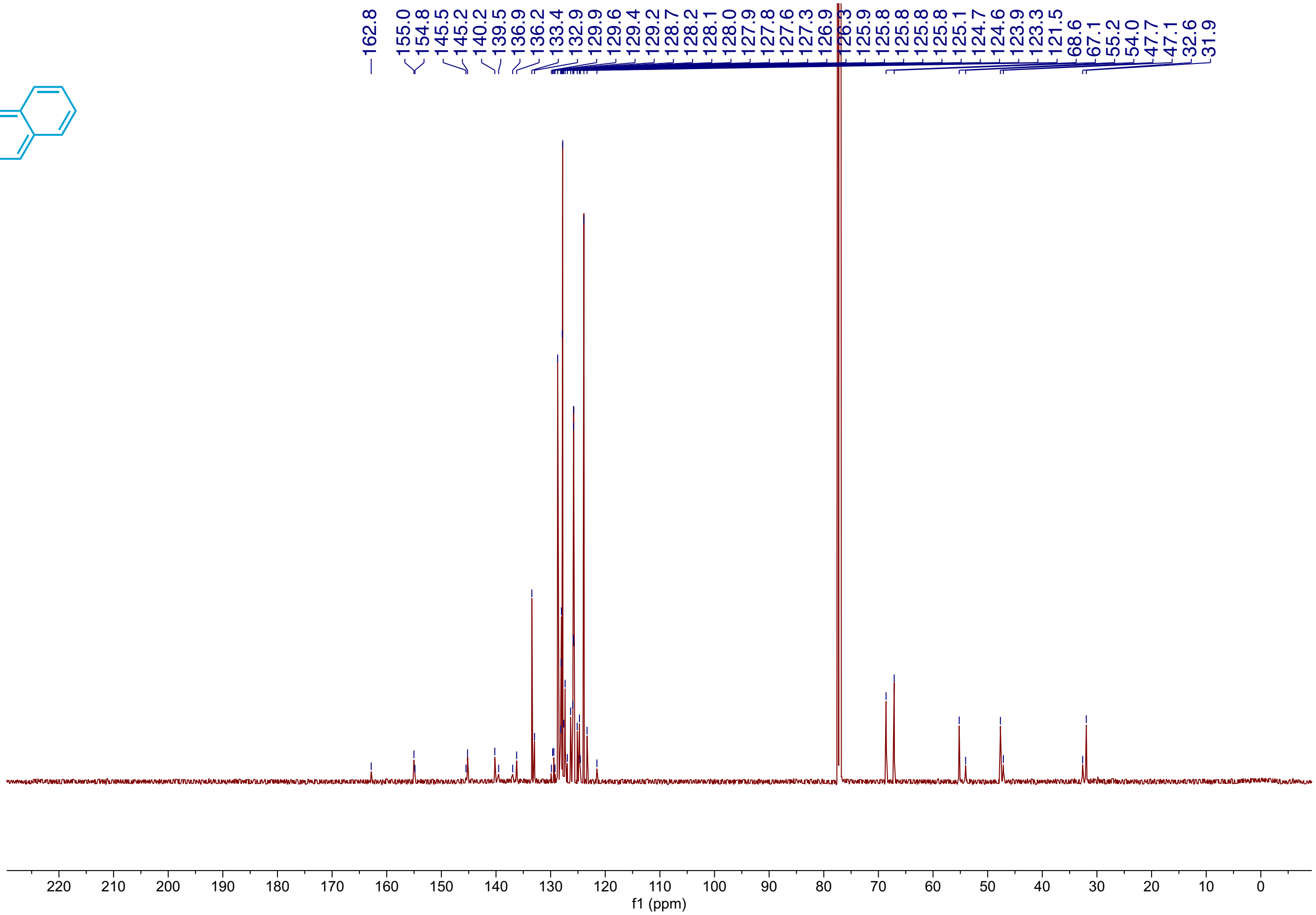

Compound 40 ^{1}H NMR

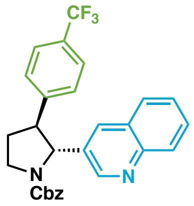

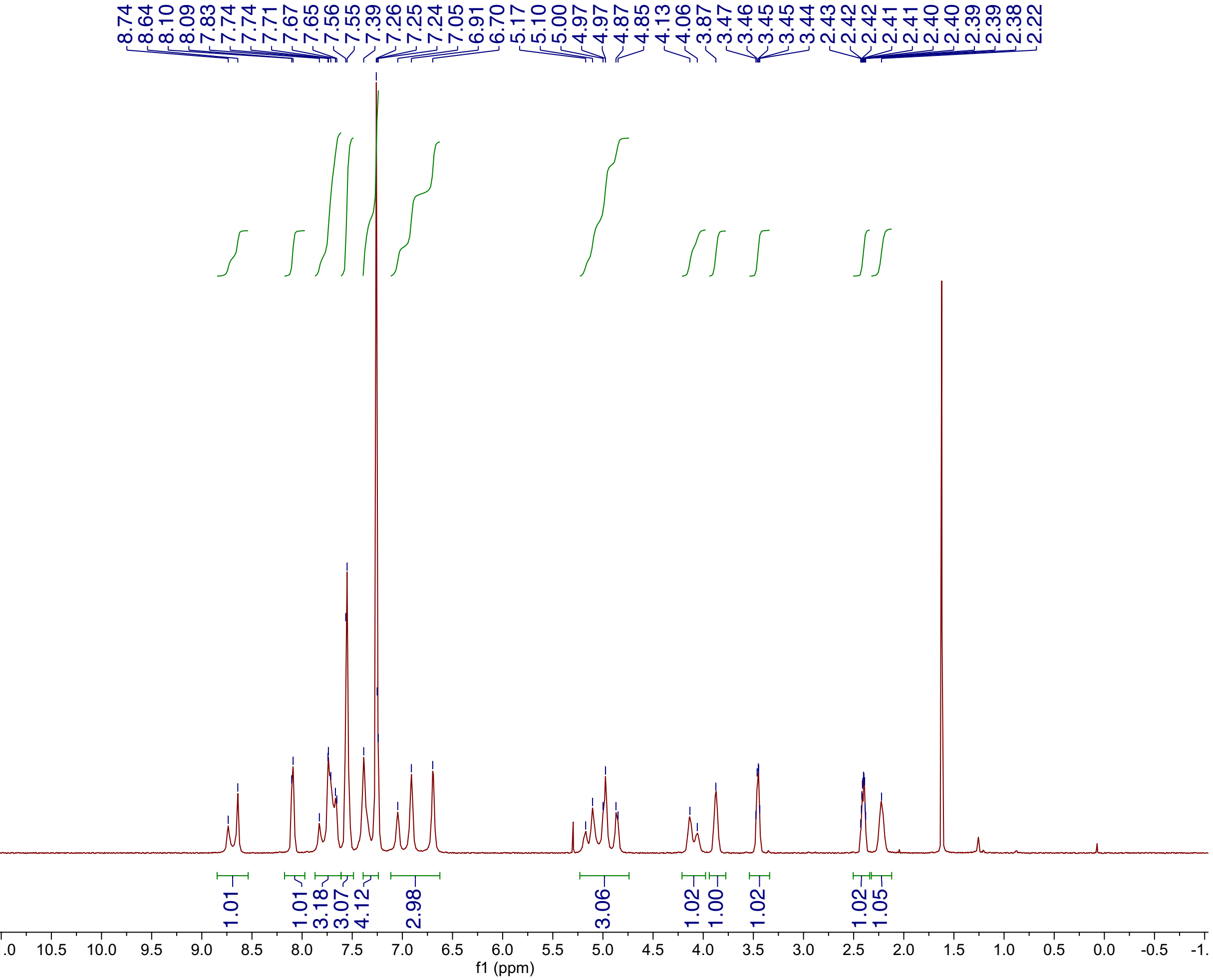

Compound 40 ^{19}F NMR

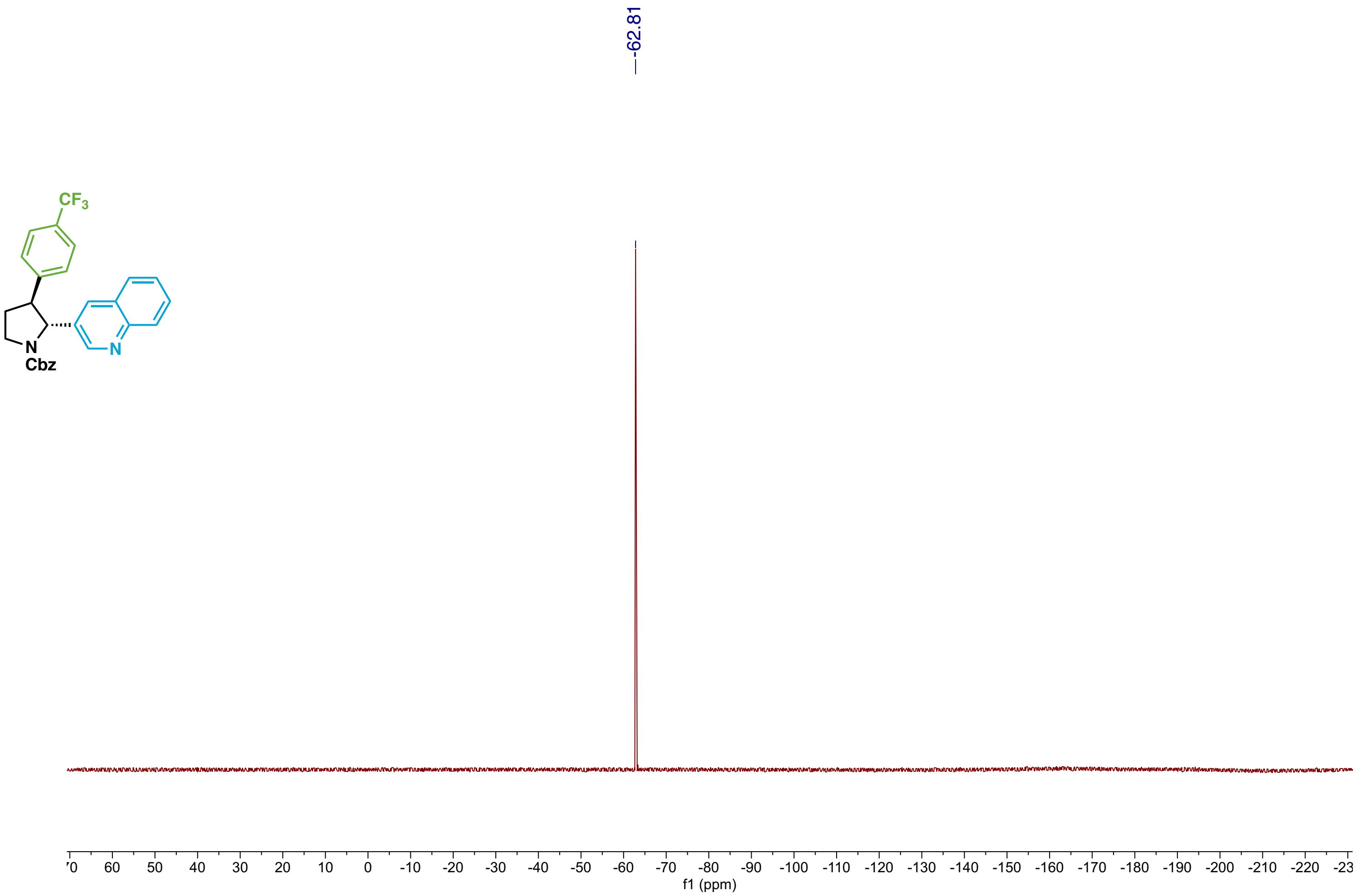

Compound 40 ^{13}C NMR

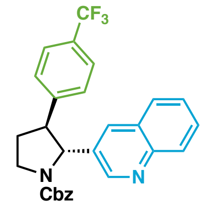

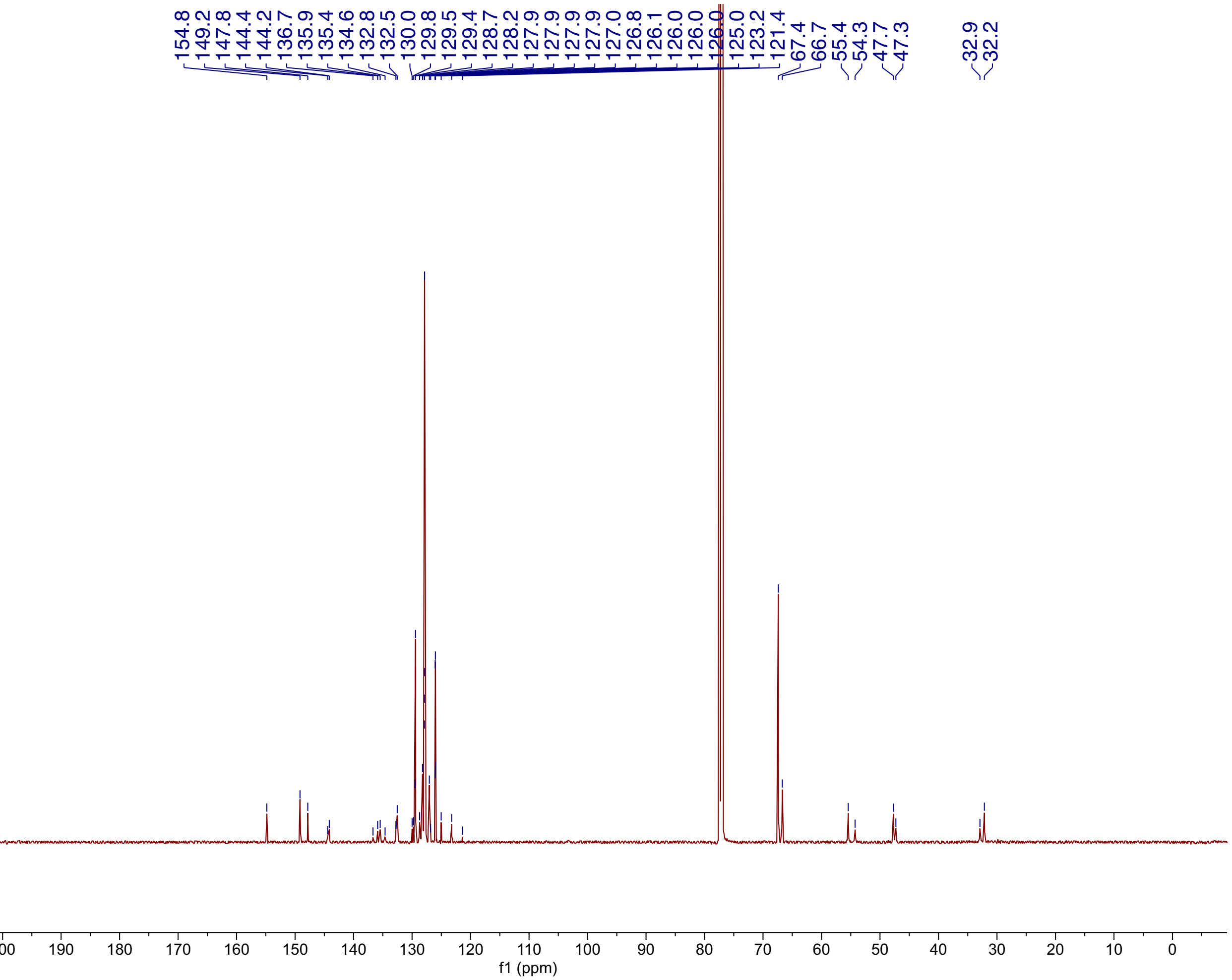

Compound B11 ^1H NMR

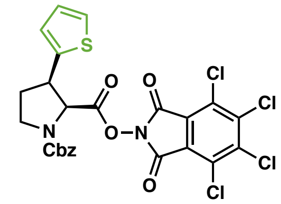

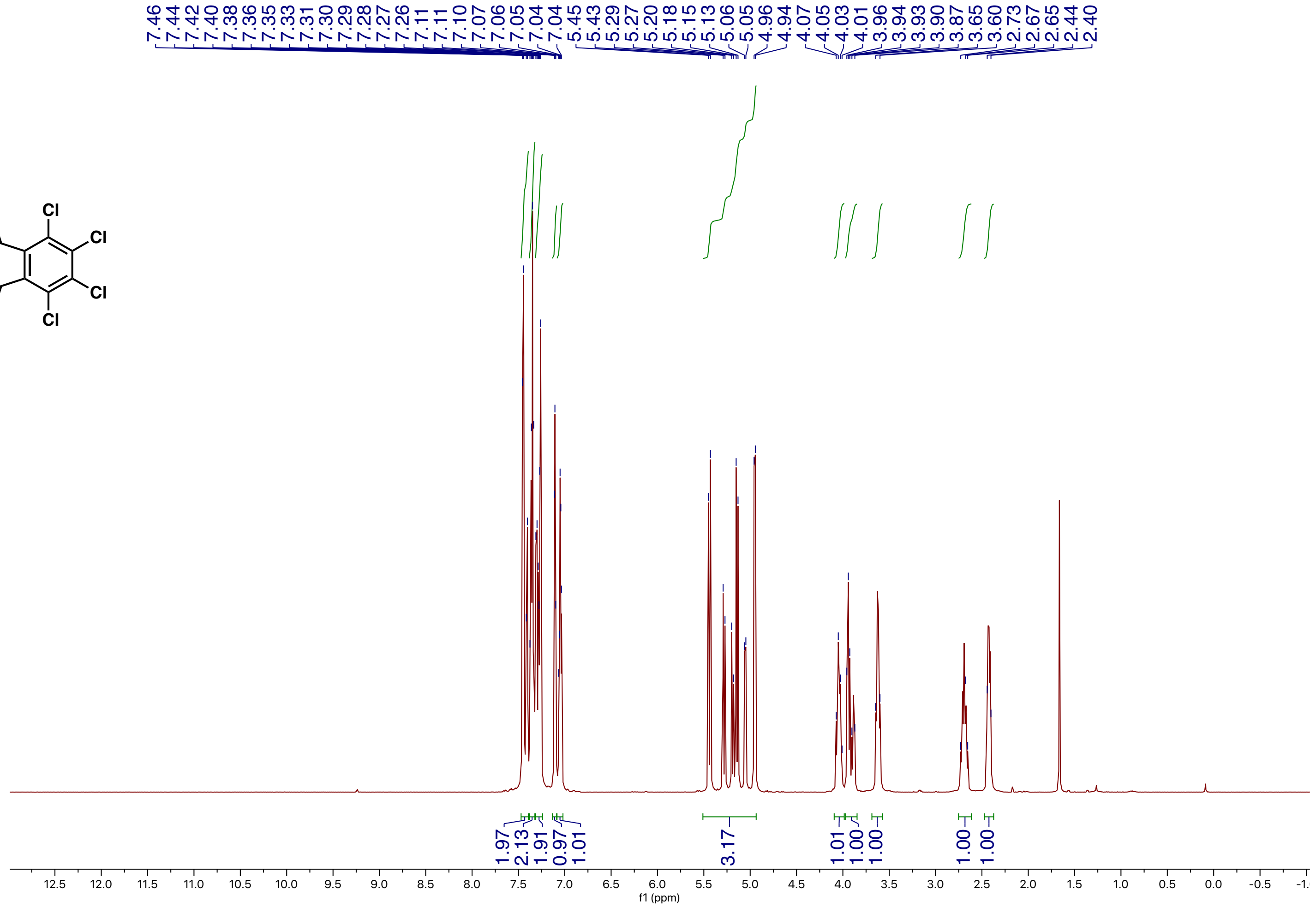

Compound B11 ^{13}C NMR

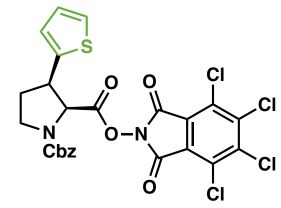

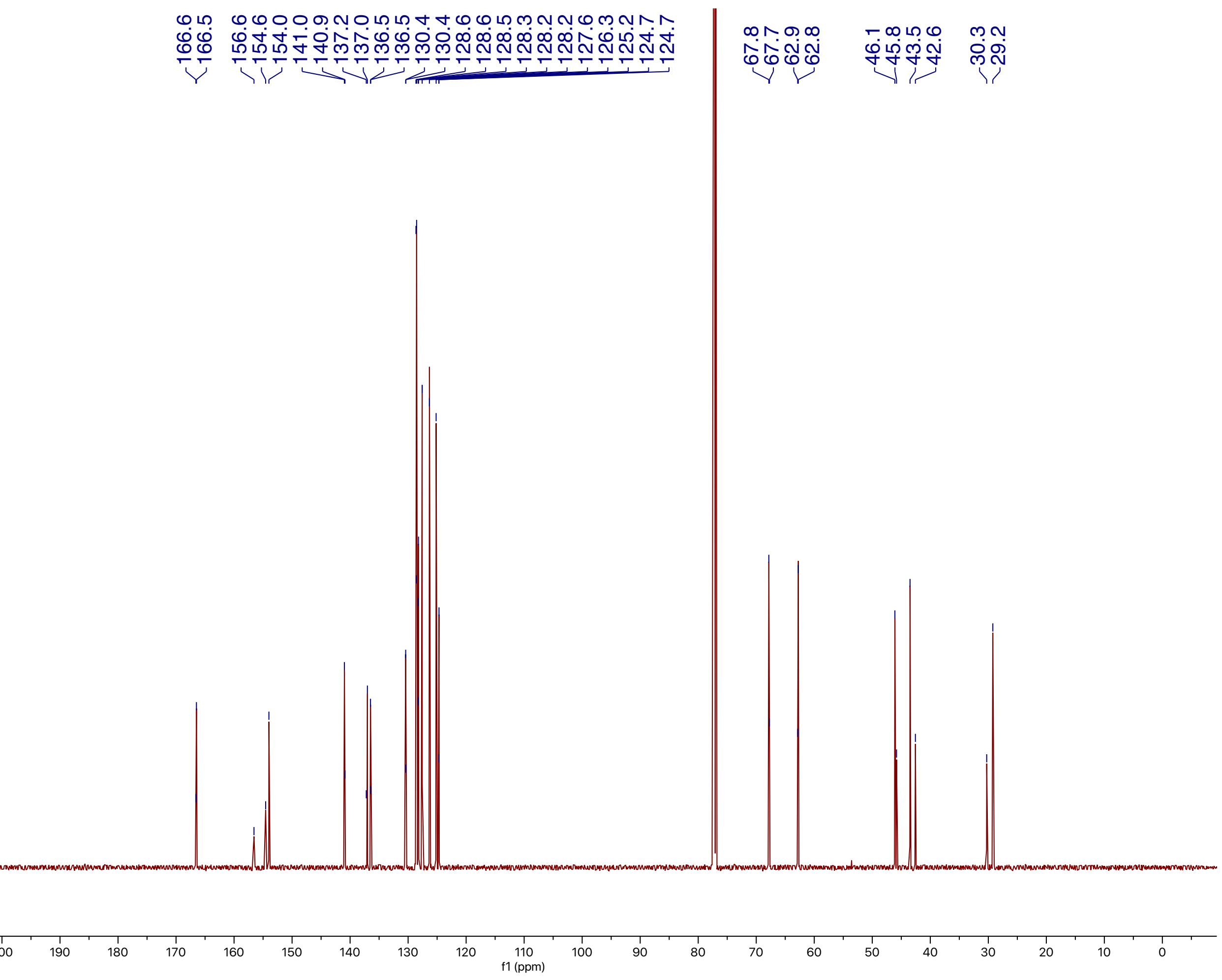

Compound 41 ^{1}H NMR

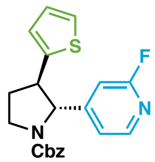

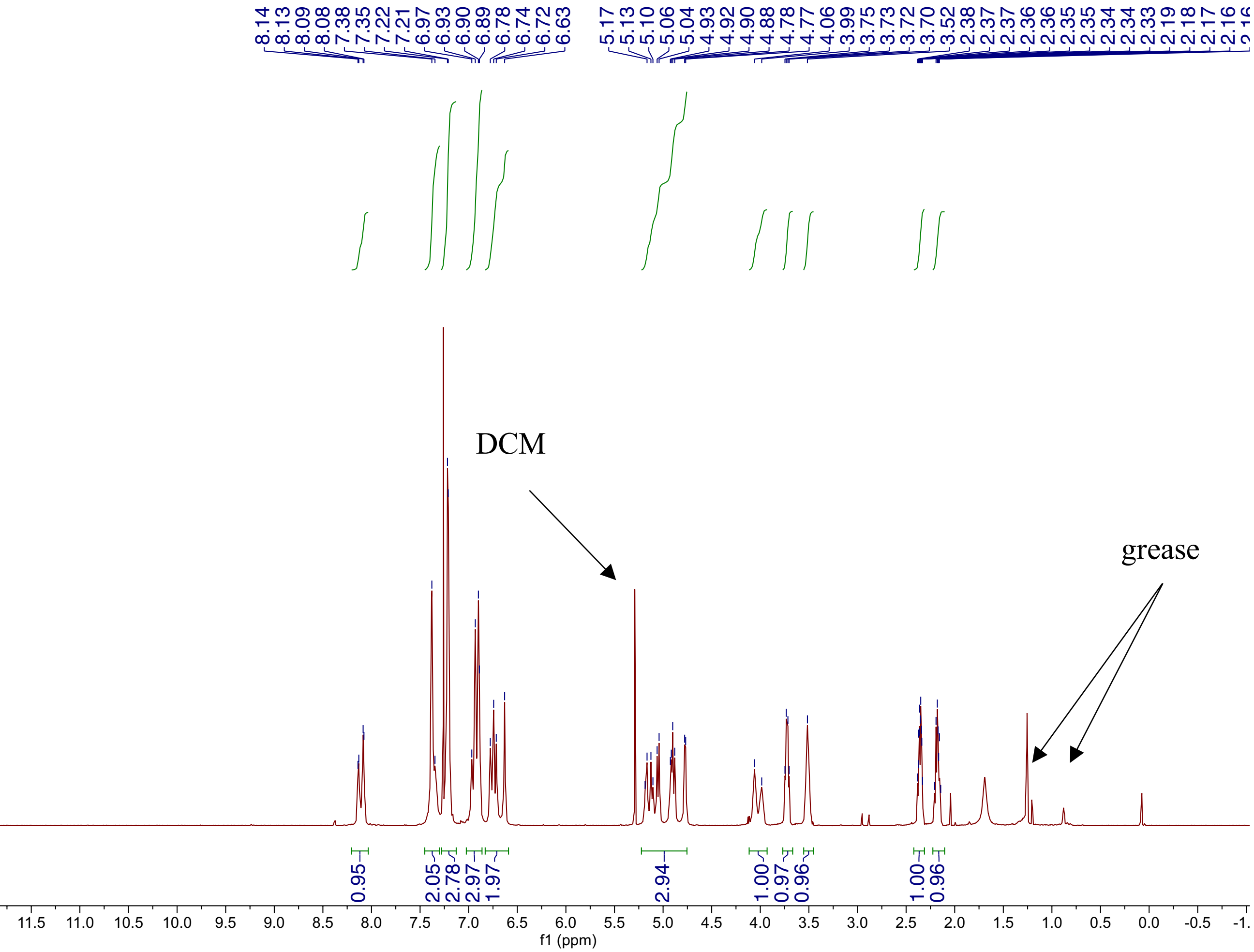

Compound 41 ^{19}F NMR

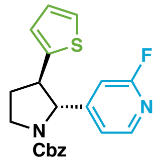

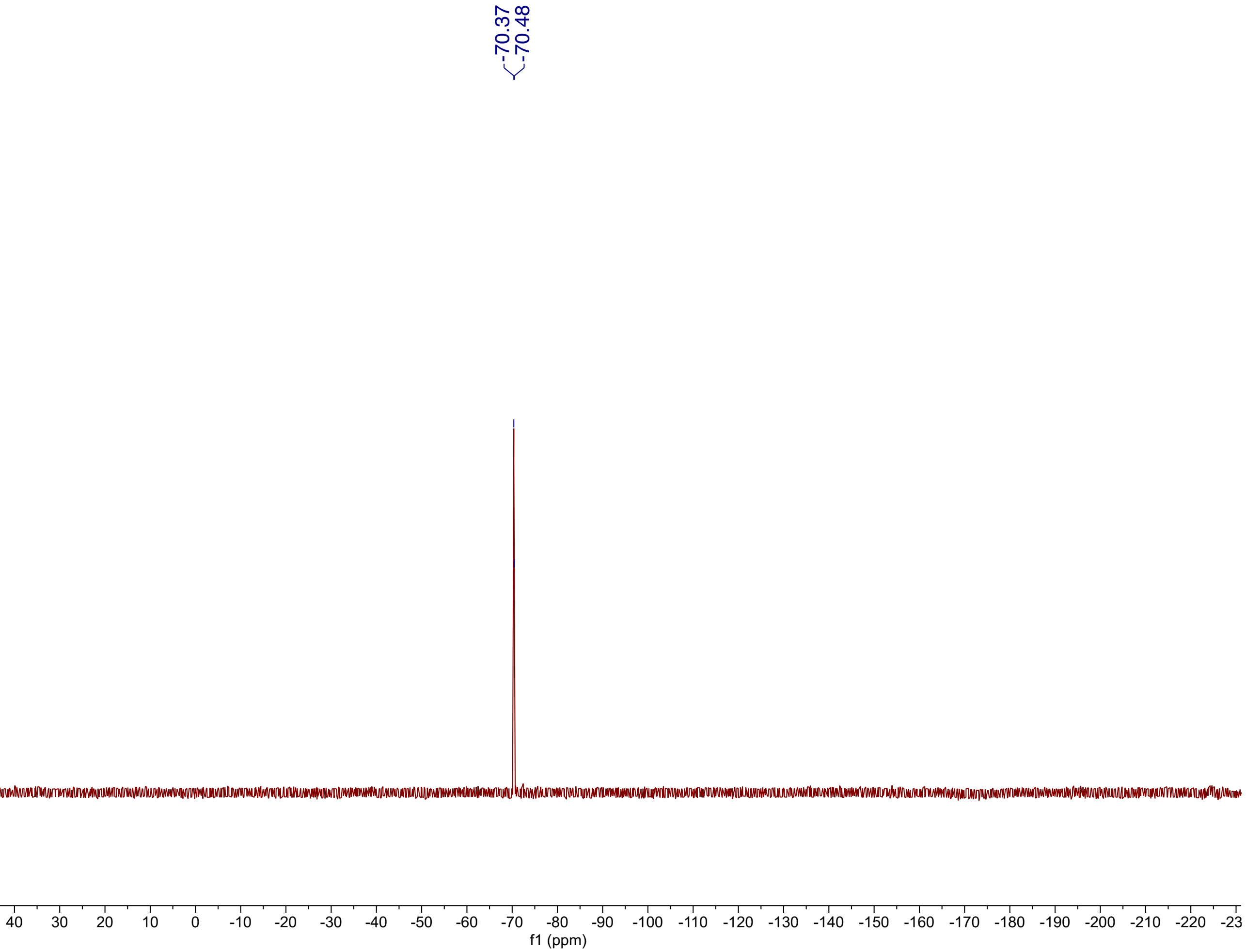

Compound 41 ^{13}C NMR

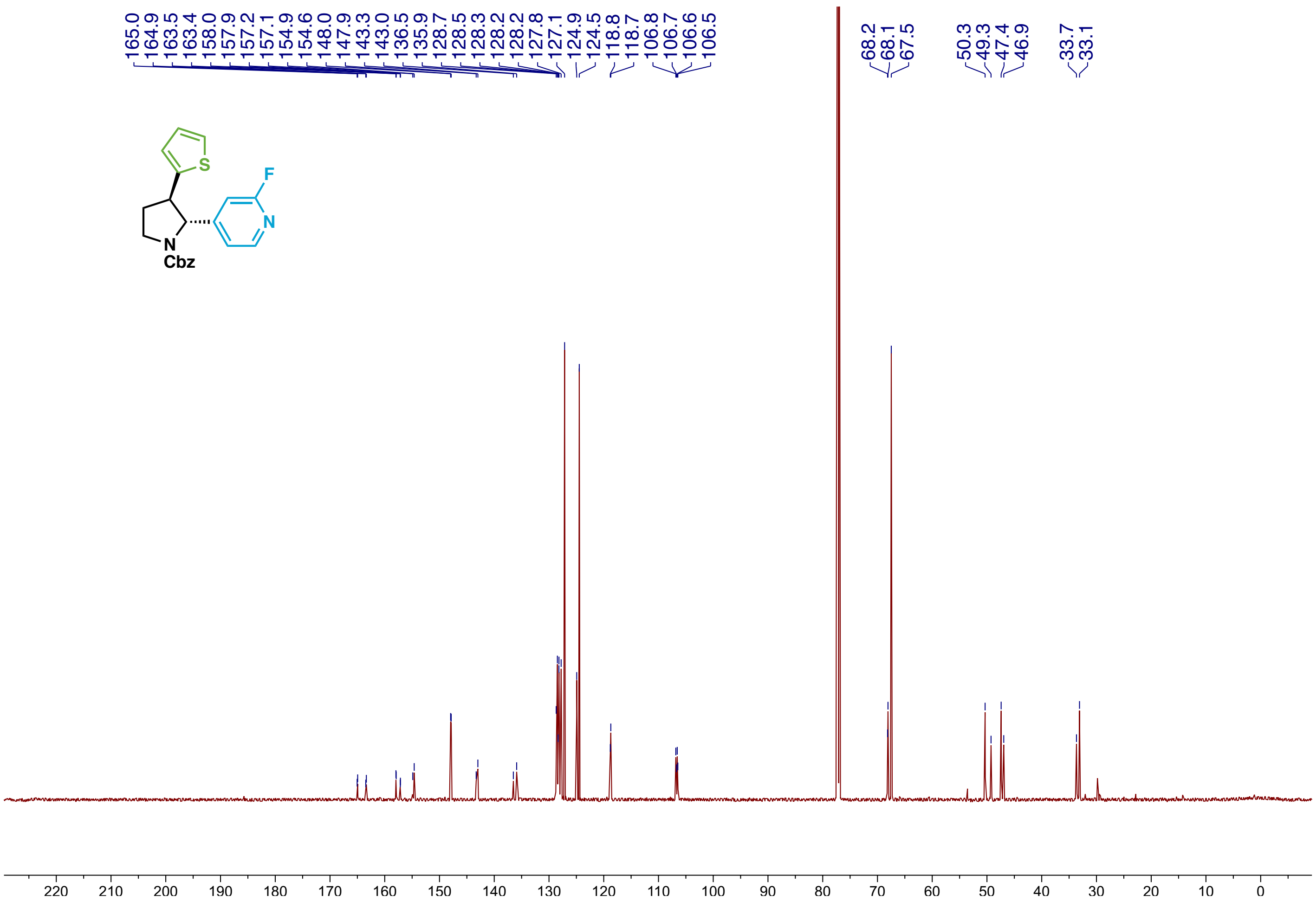

Compound 42 ^{1}H NMR

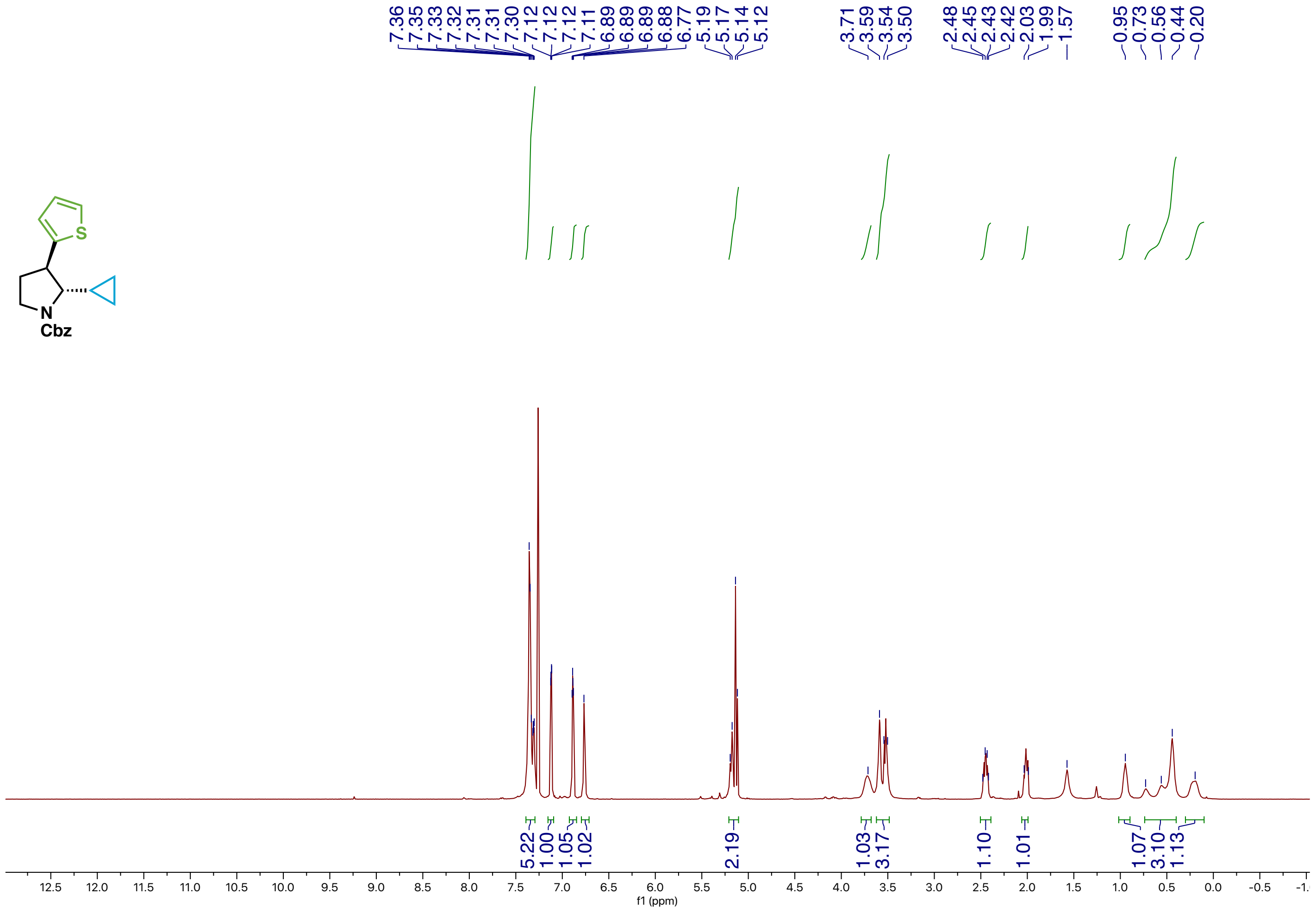

Compound 42 ^{13}C NMR

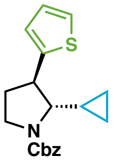

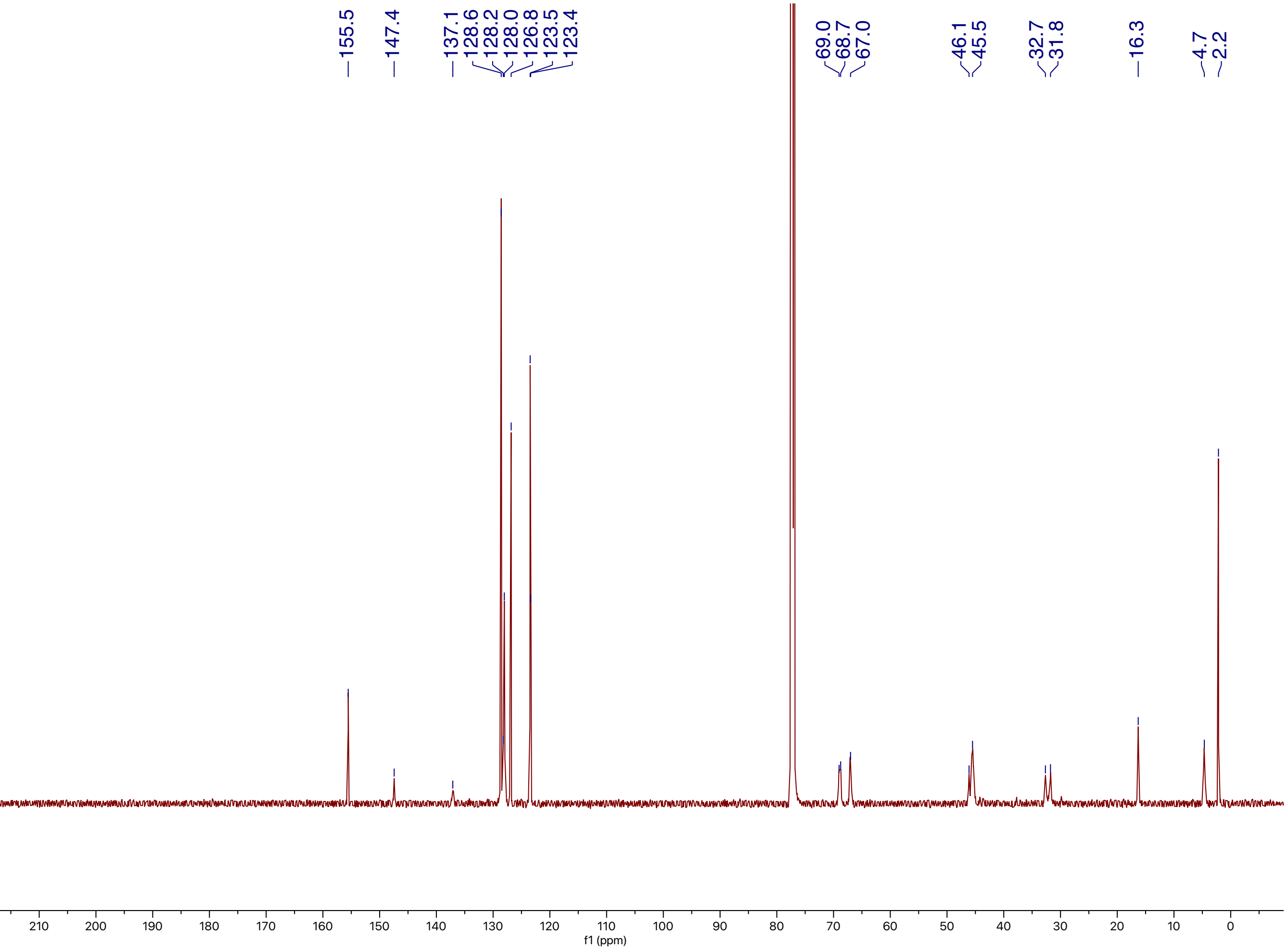

Compound 43 ^{1}H NMR

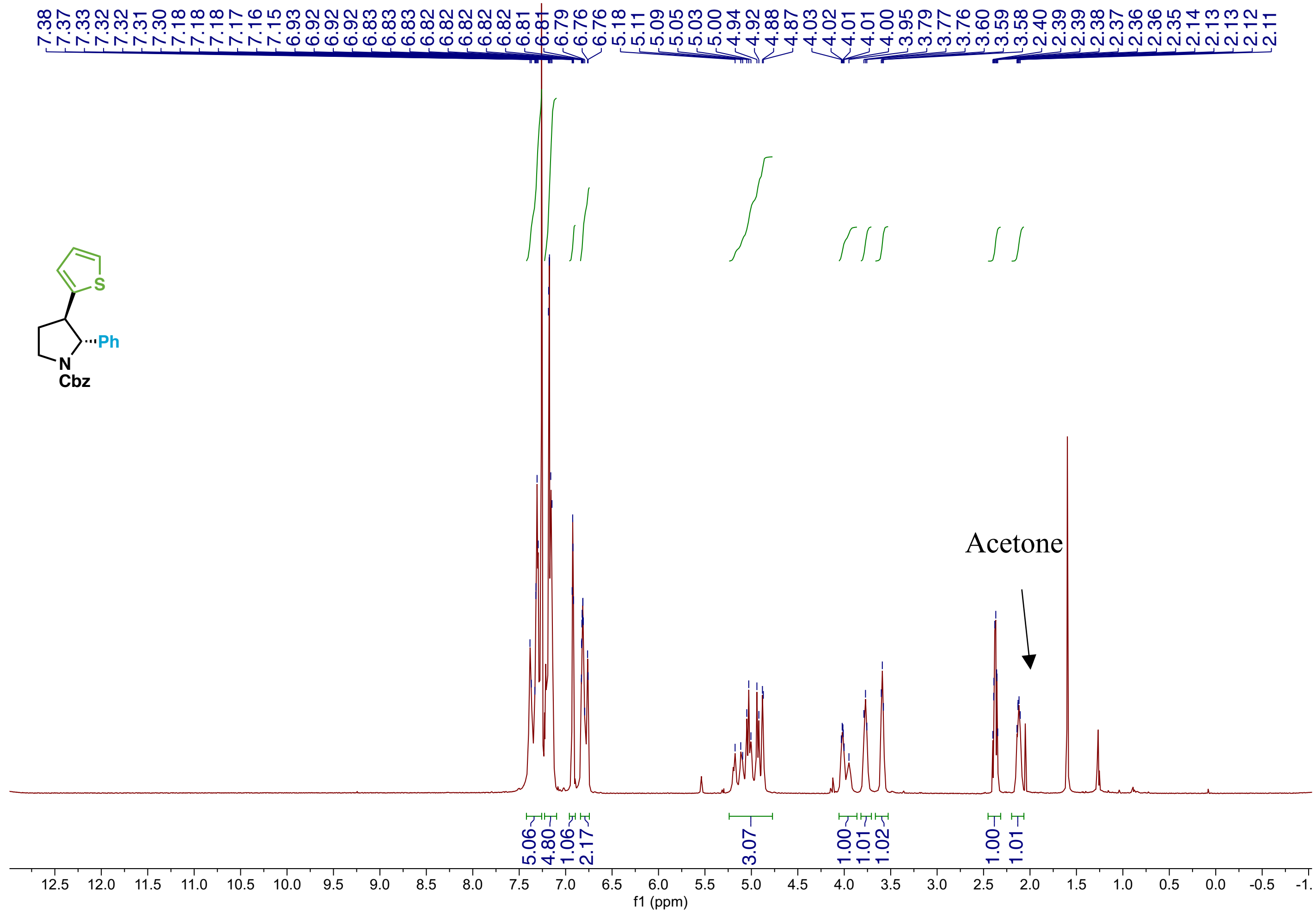

Compound 43 ^{13}C NMR

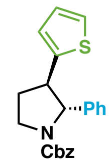

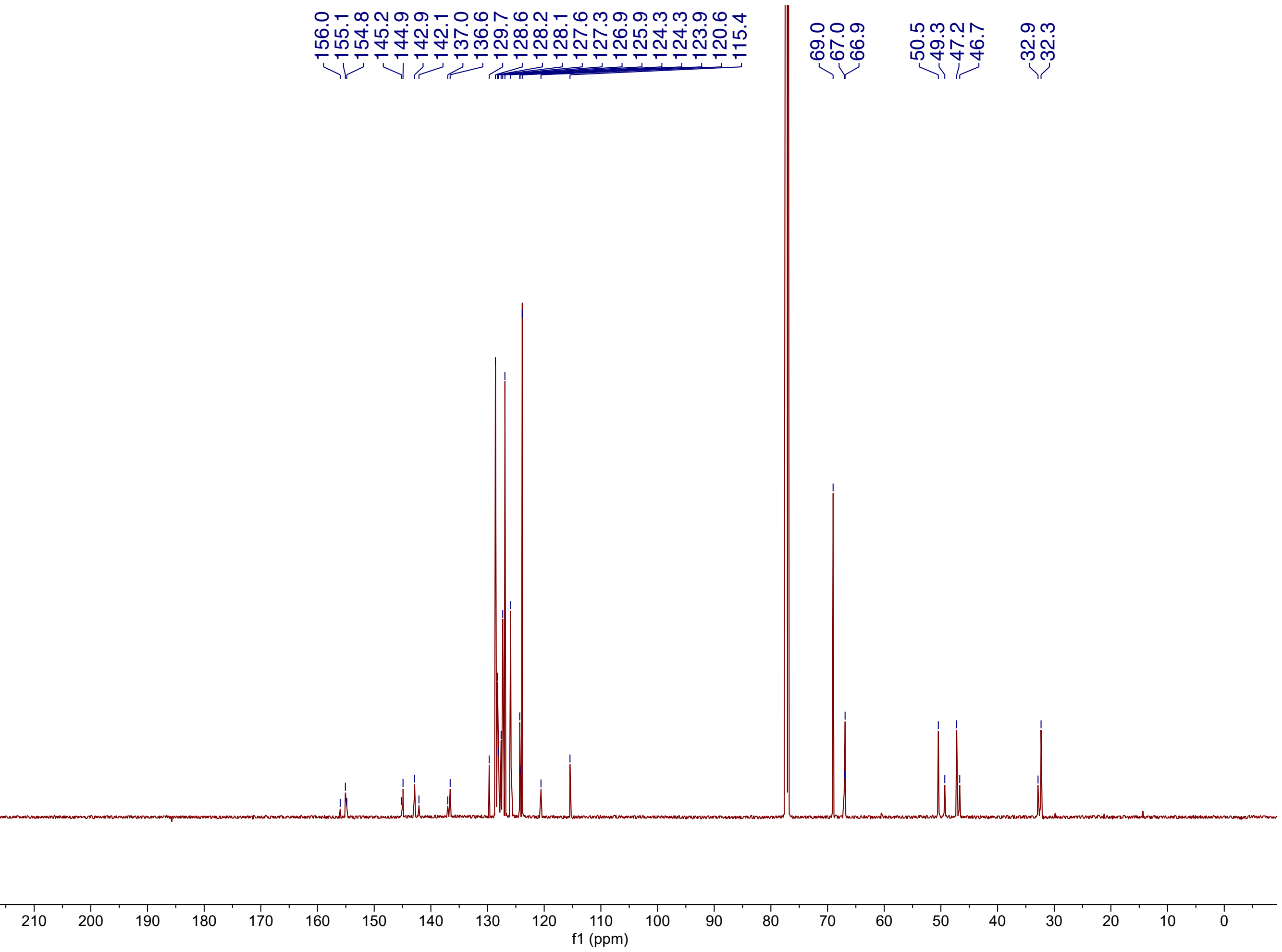

Compound 44 ^{1}H NMR

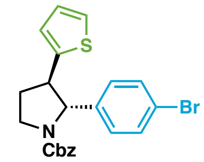

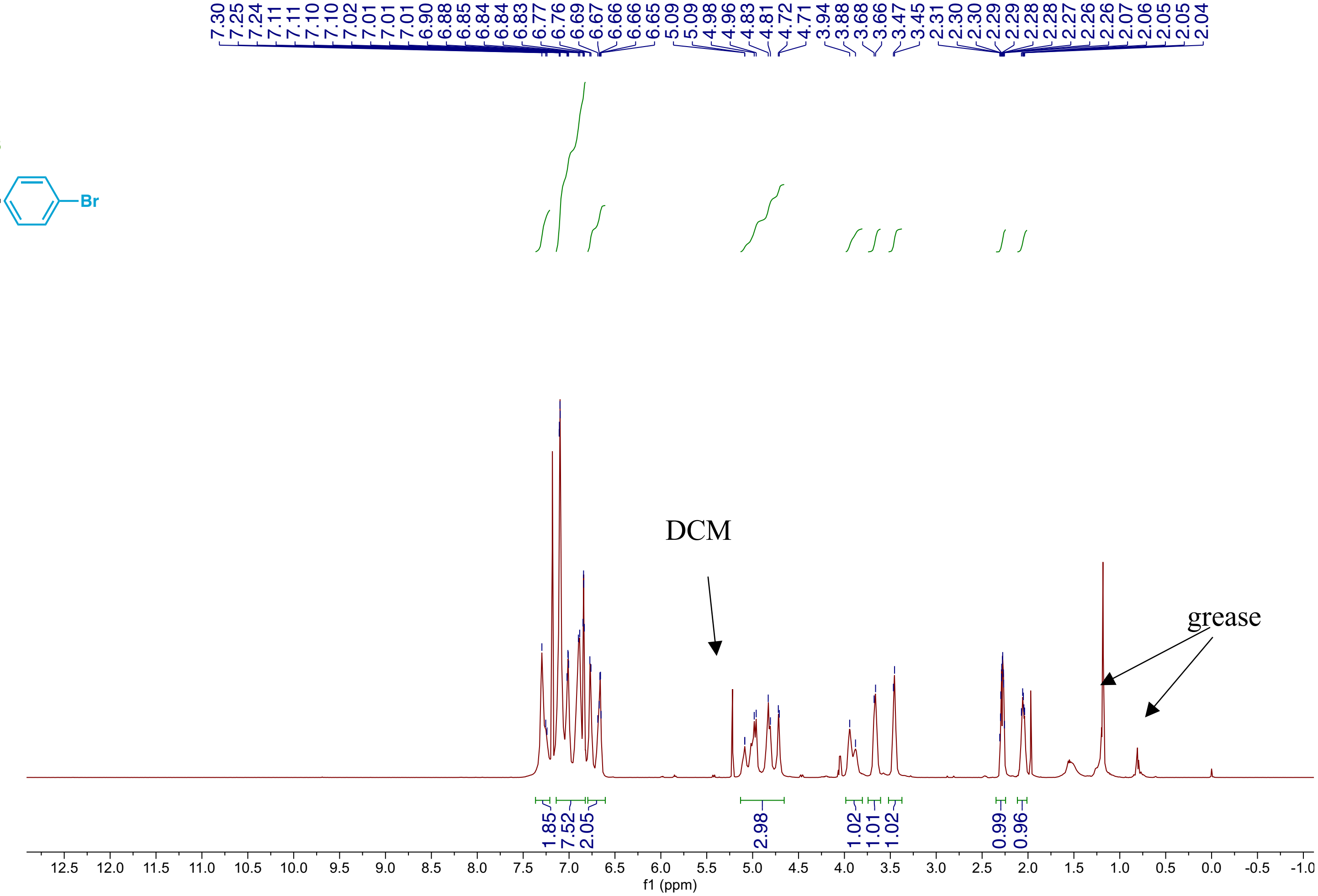

Compound 44 ^{13}C NMR

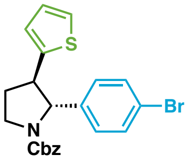

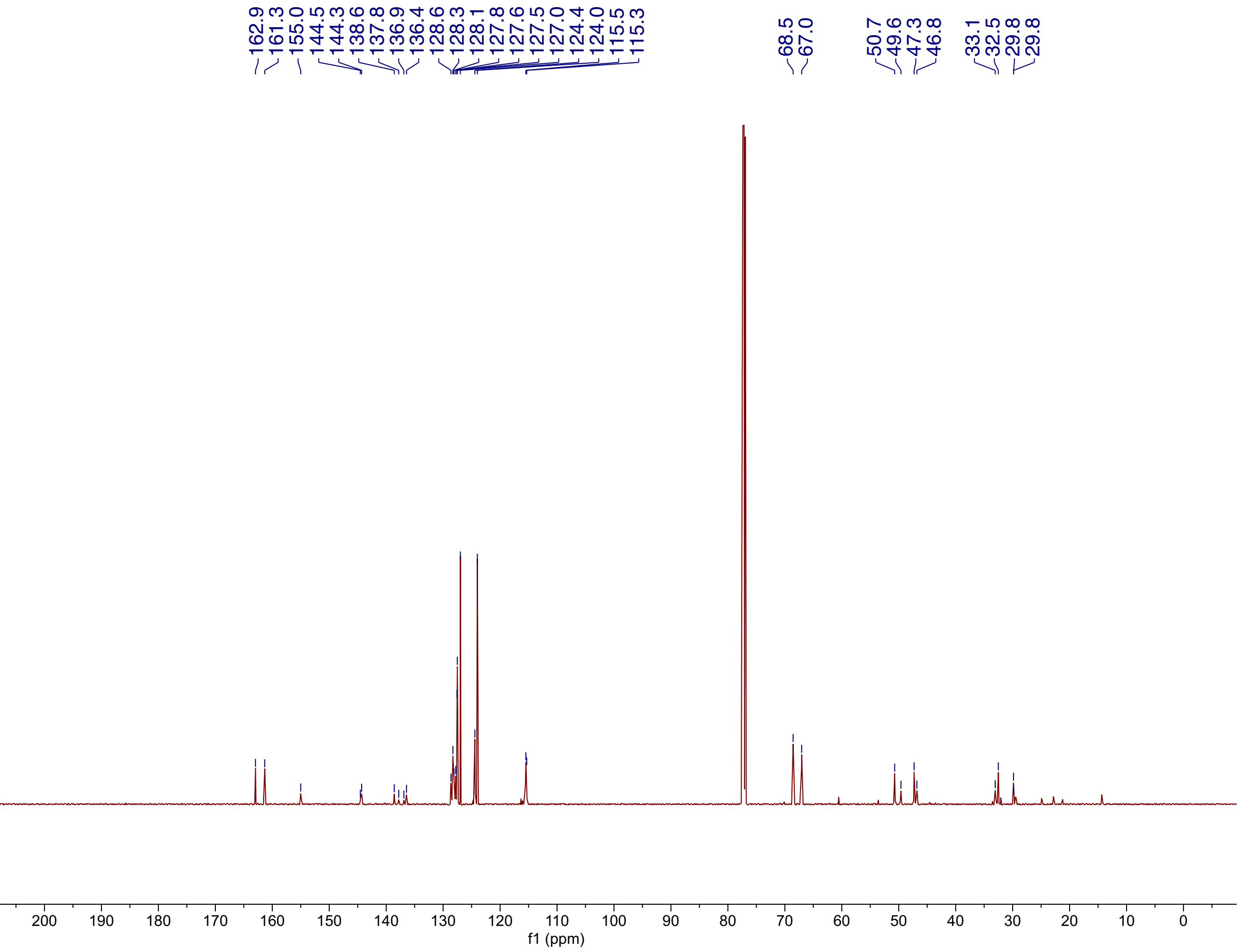

Compound B15 ^{1}H NMR

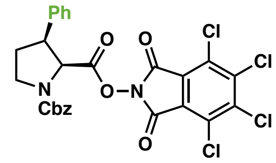

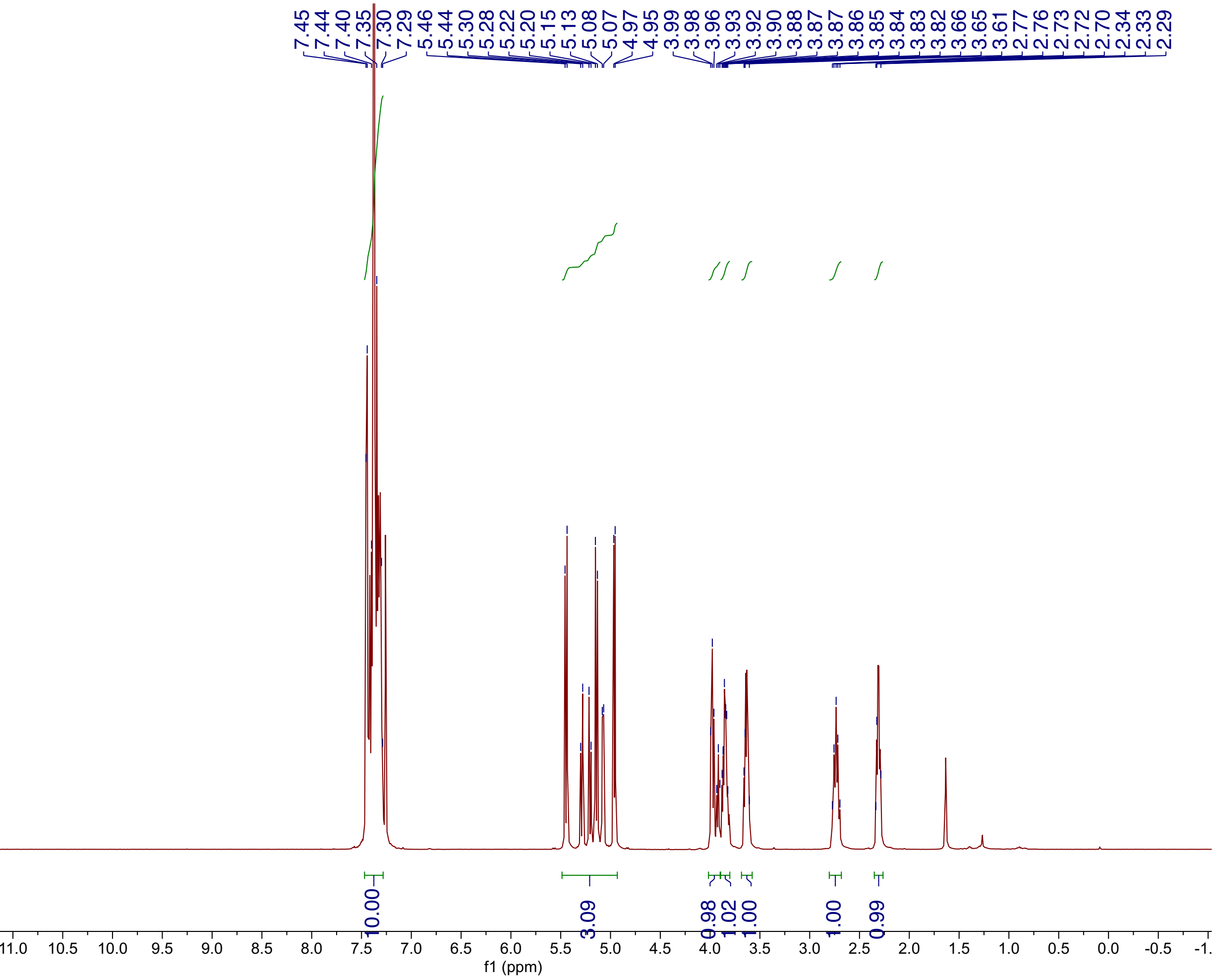

Compound B15 ^{13}C NMR

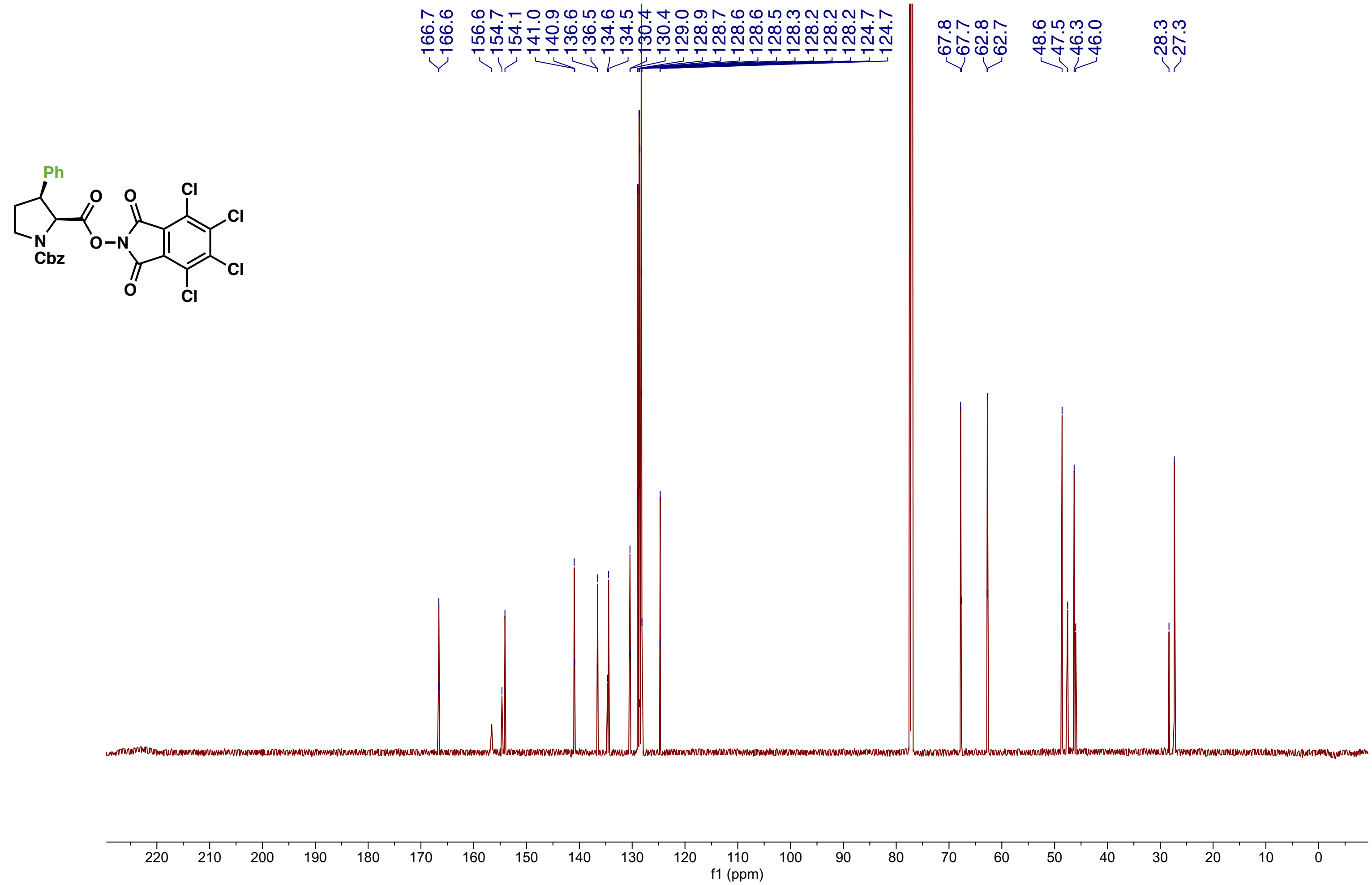

Compound 45 ^{1}H NMR

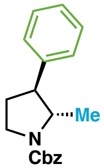

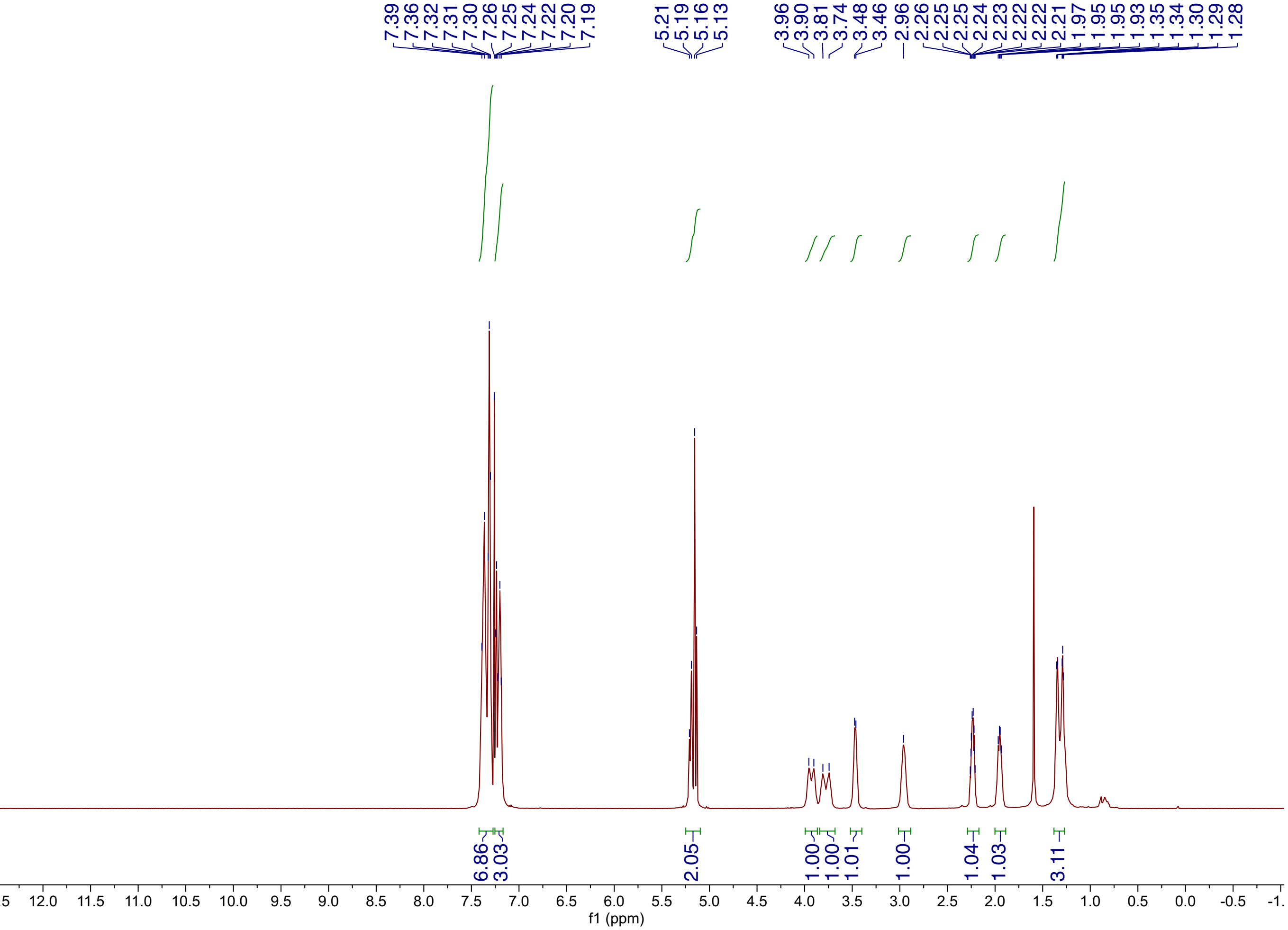

Compound 45 ^{13}C NMR

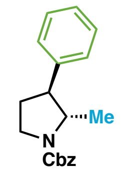

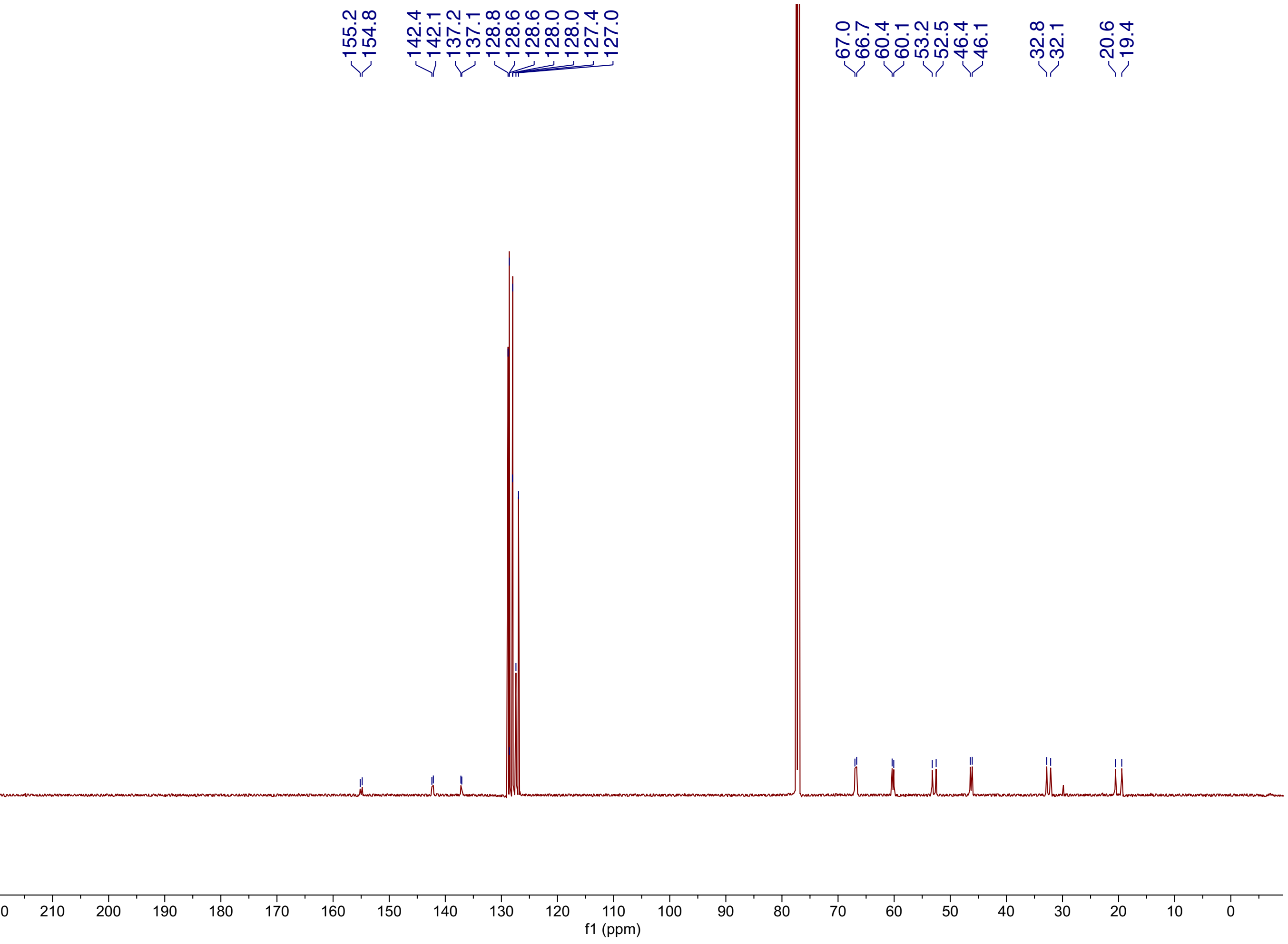

Compound SI-40 ^{1}H NMR

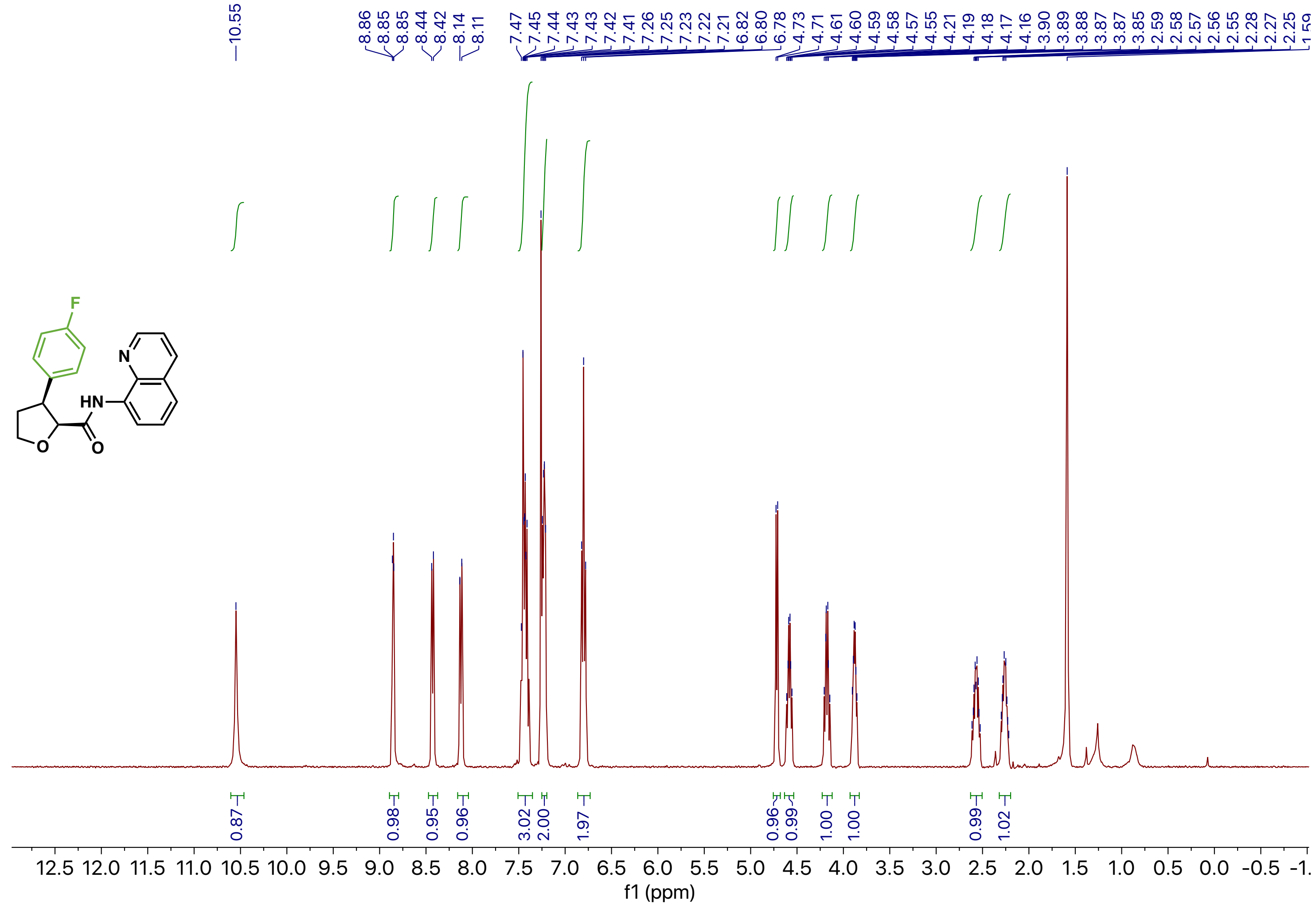

Compound SI-40 ^{19}F NMR

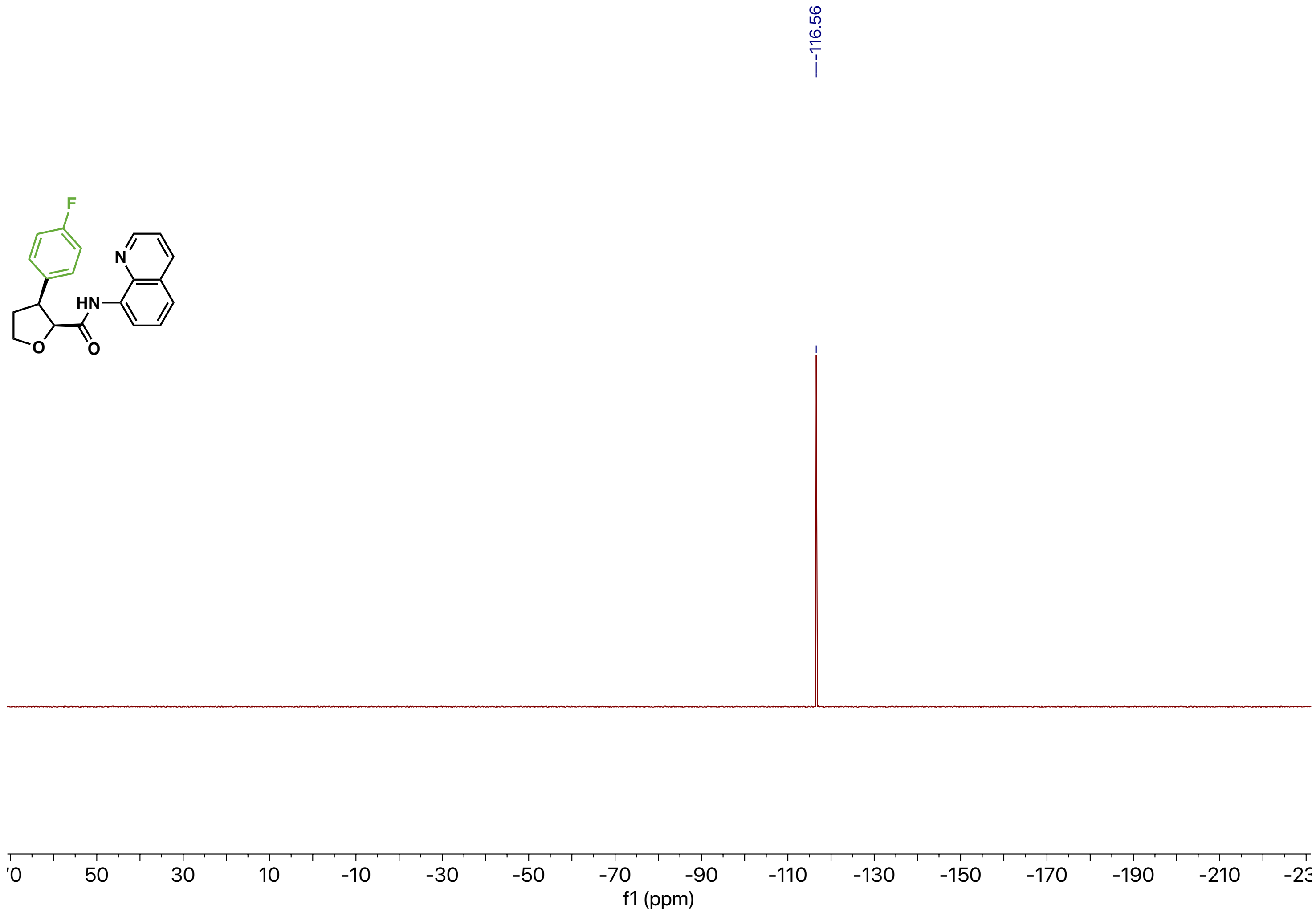

Compound SI-40 ^{13}C NMR

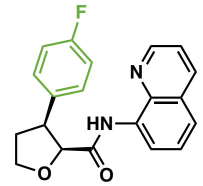

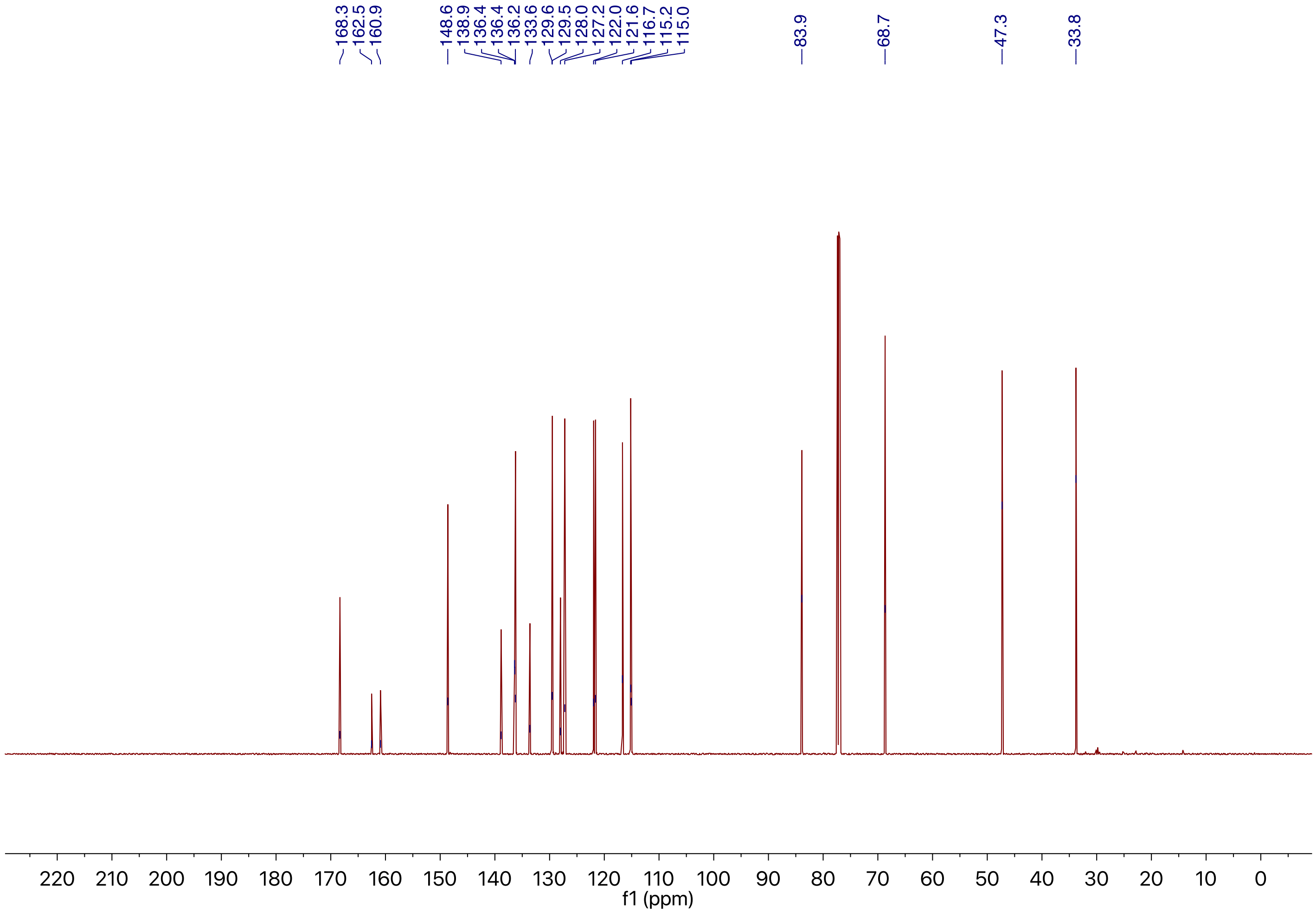

Compound SI-41 ^{1}H NMR

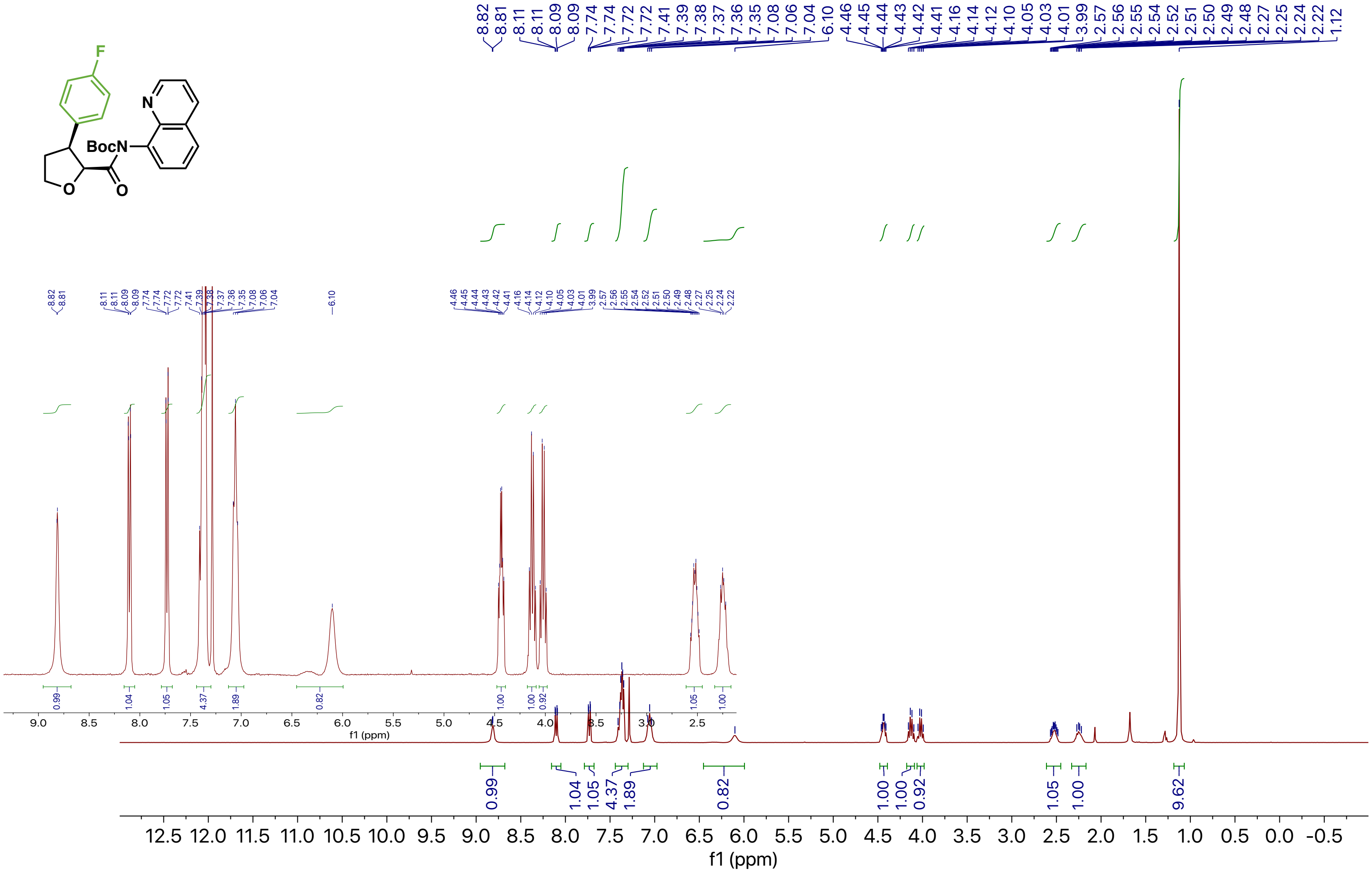

Compound SI-41 ^{19}F NMR

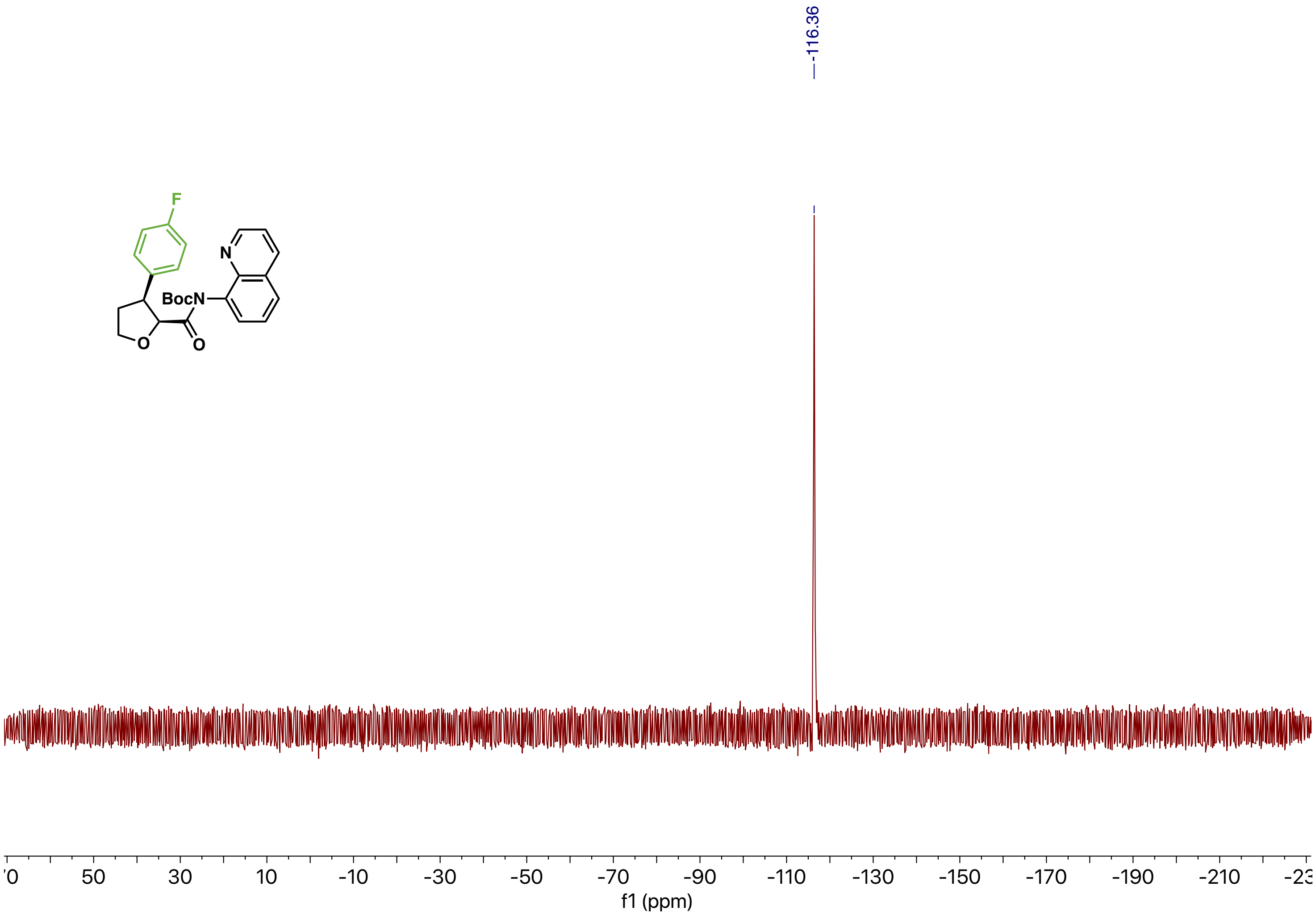

Compound SI-41 ^{13}C NMR

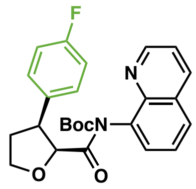

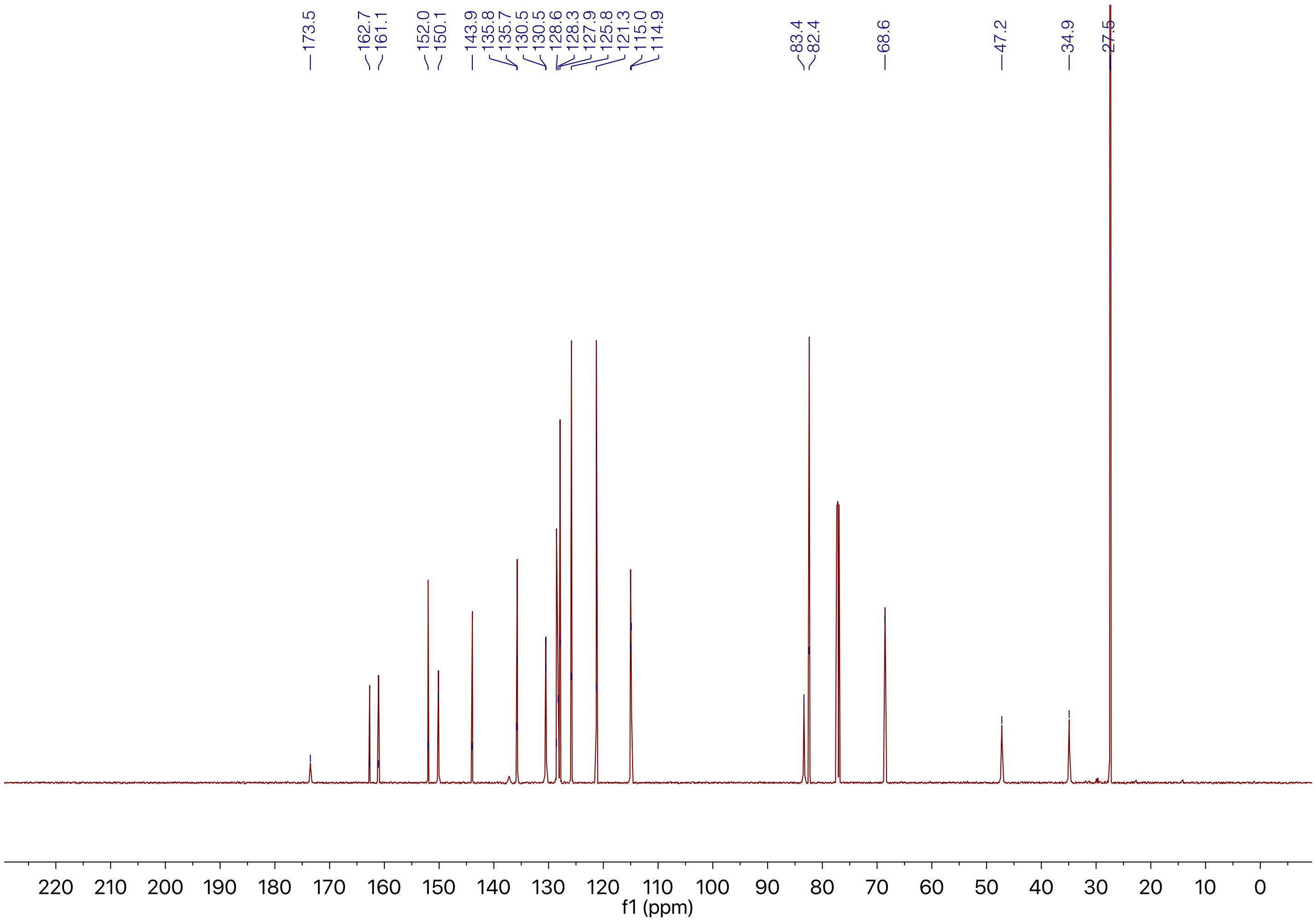

Compound A9 ^{1}H NMR

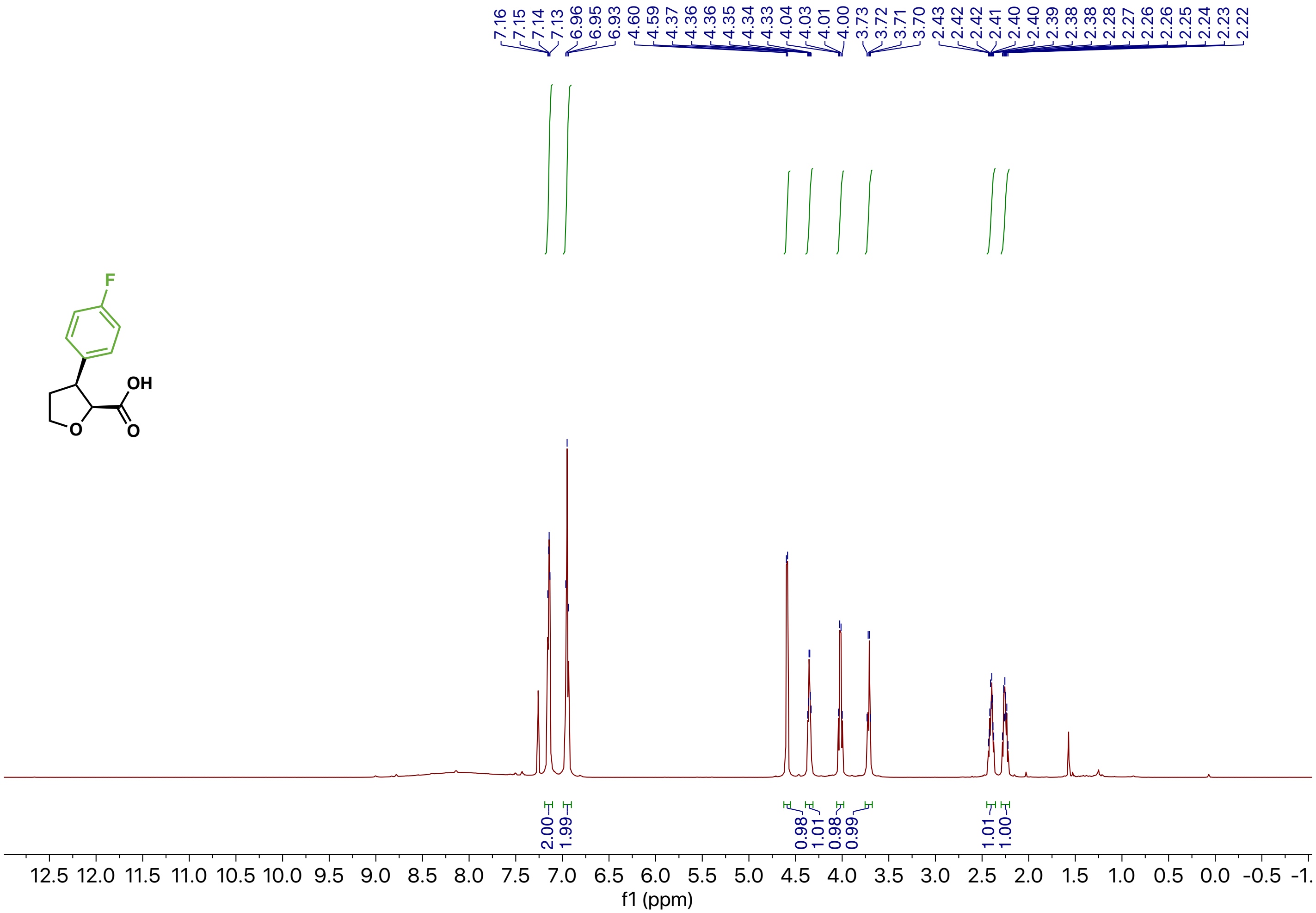

Compound A9 ^{19}F NMR

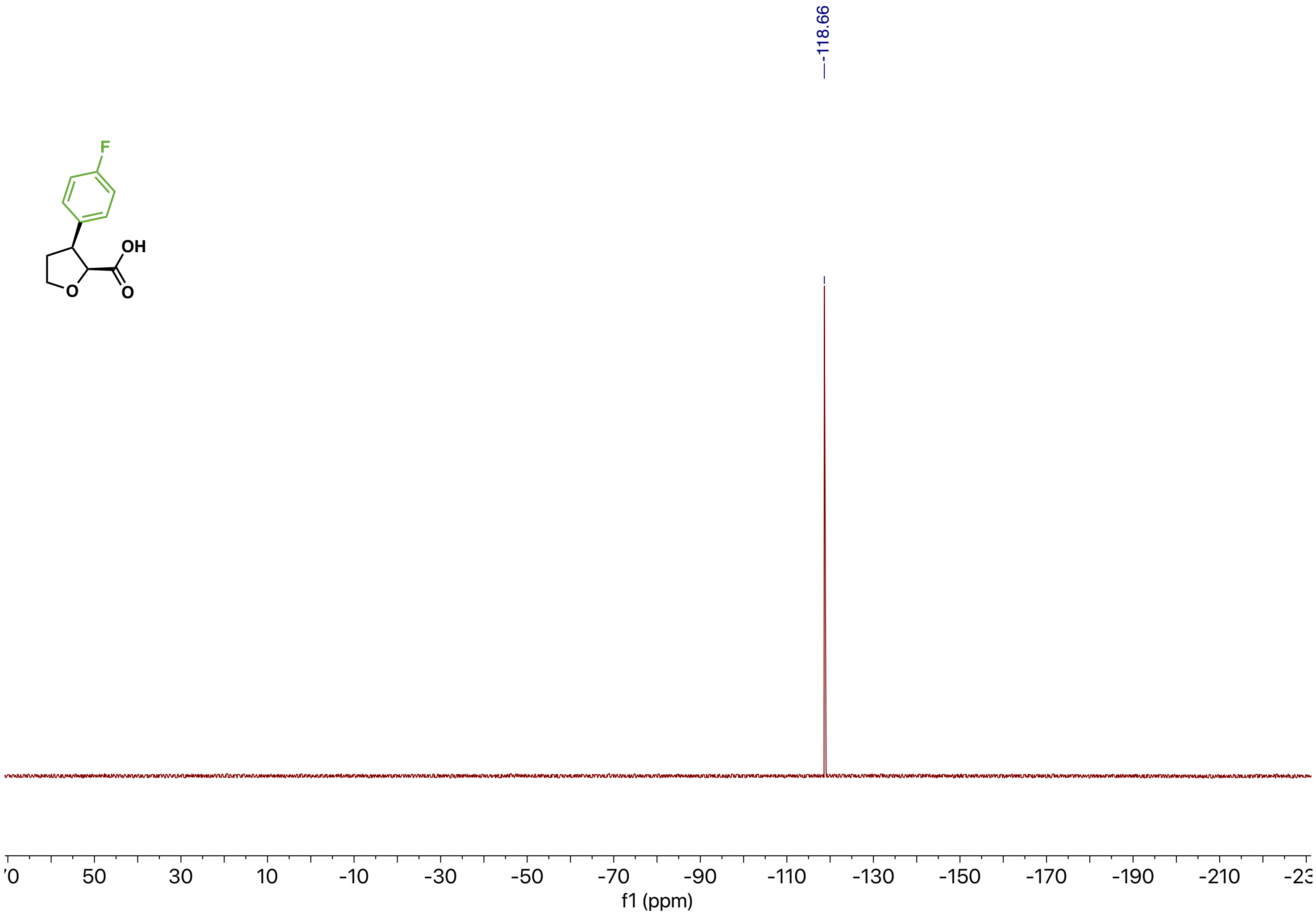

Compound A9 ^{13}C NMR

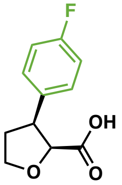

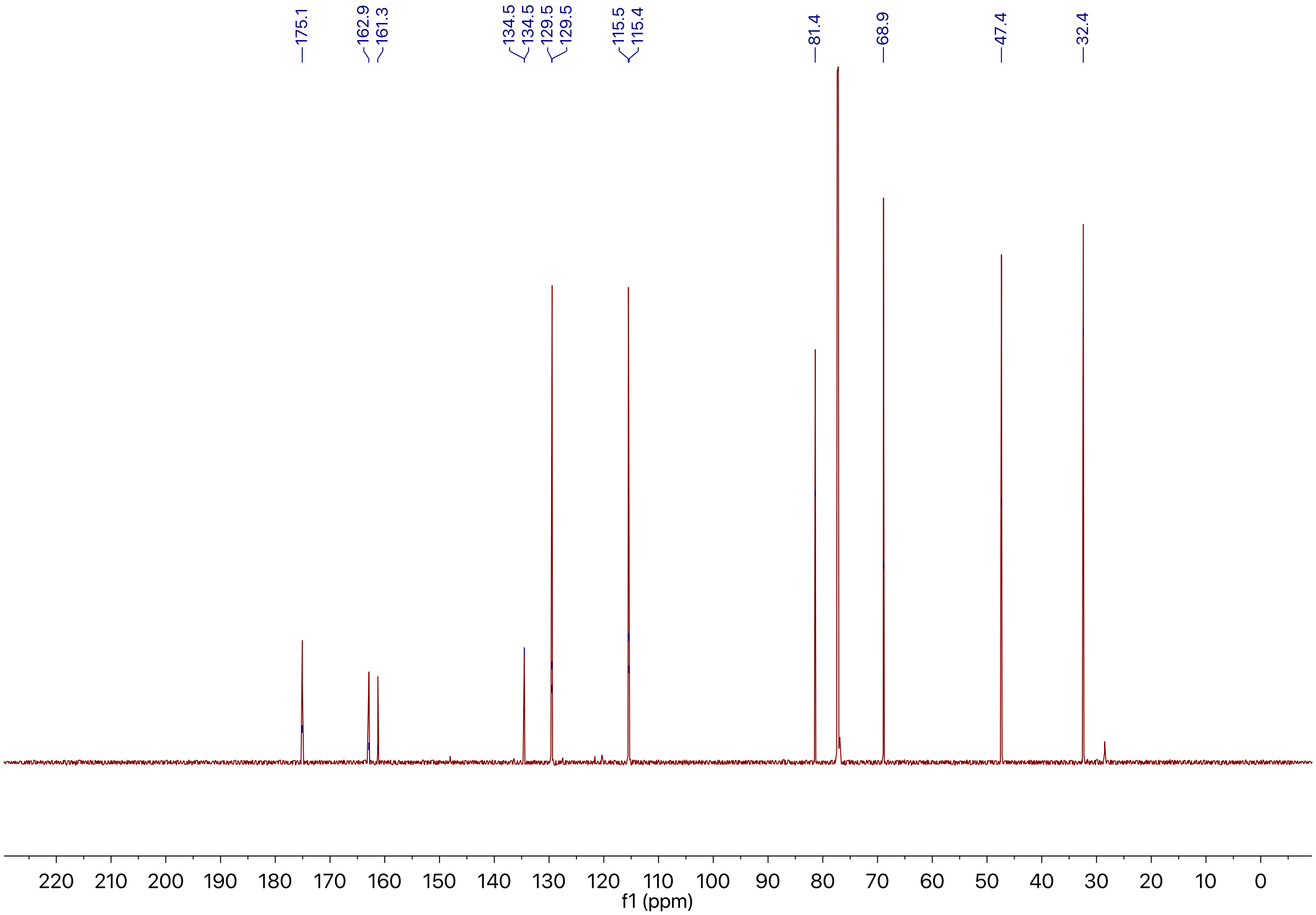

Compound B12 ^{1}H NMR

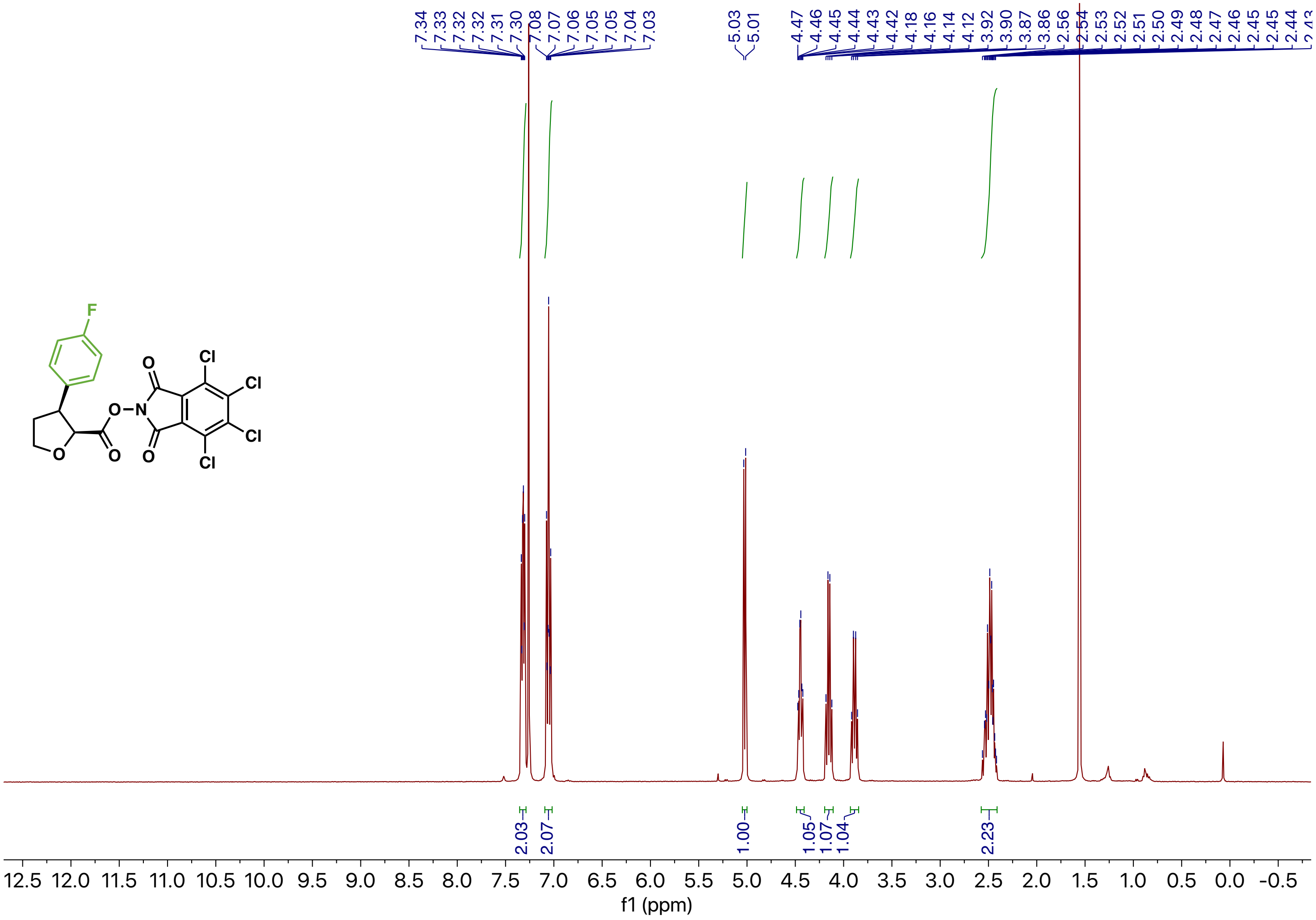

Compound B12 ^{19}F NMR

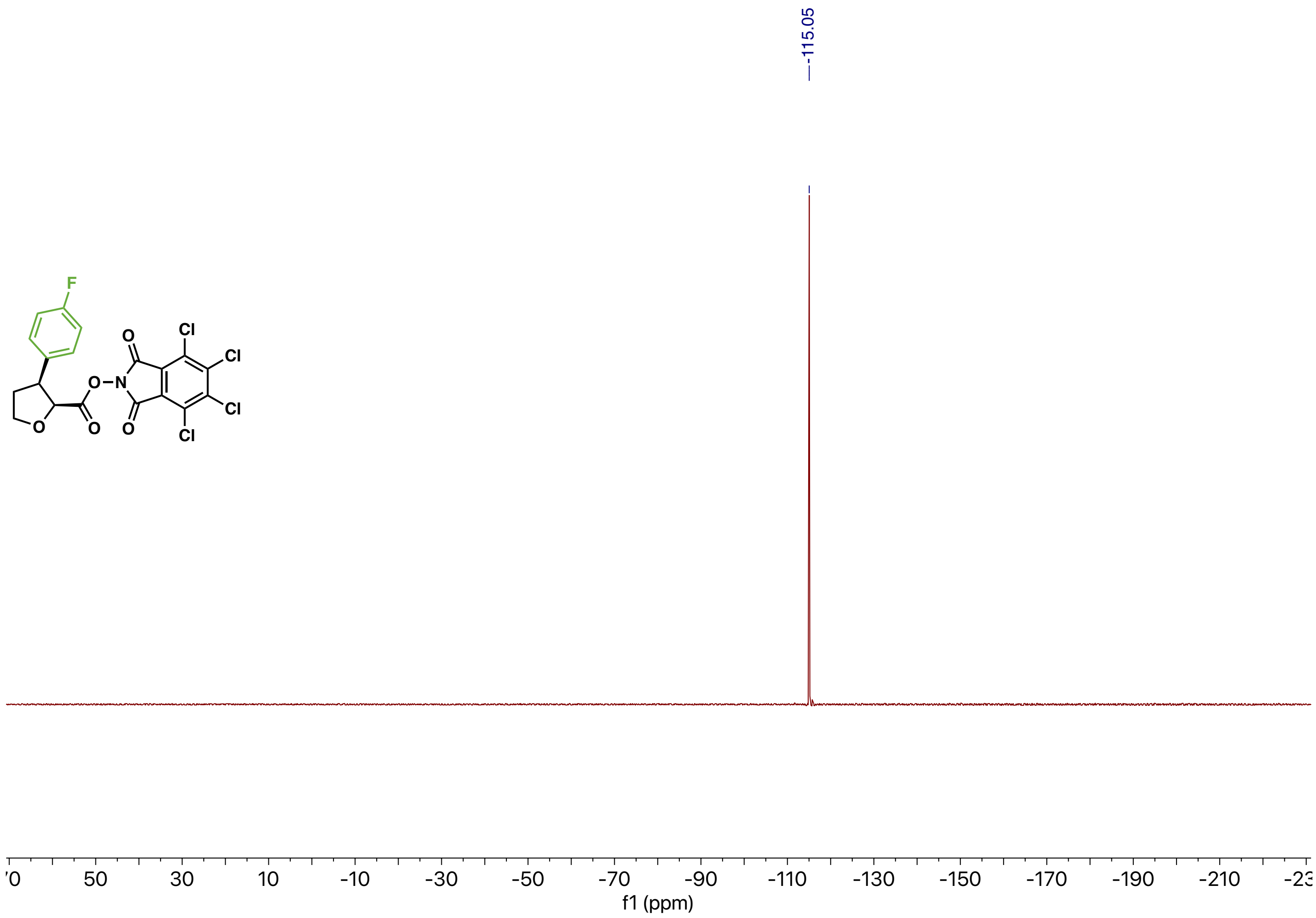

Compound B12 ^{13}C NMR

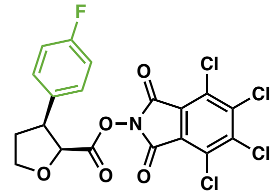

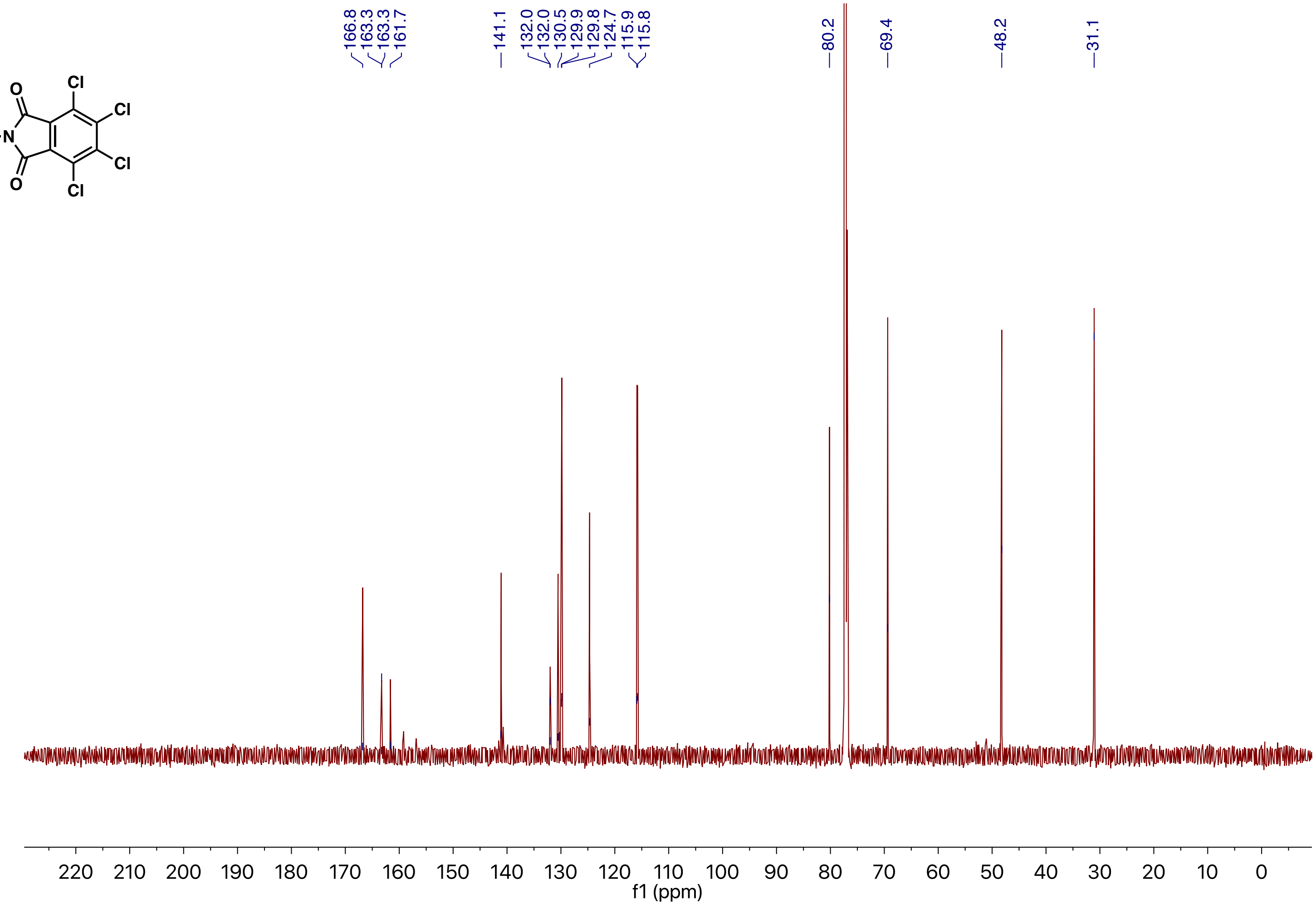

Compound 46 ^{1}H NMR

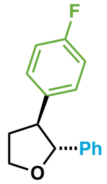

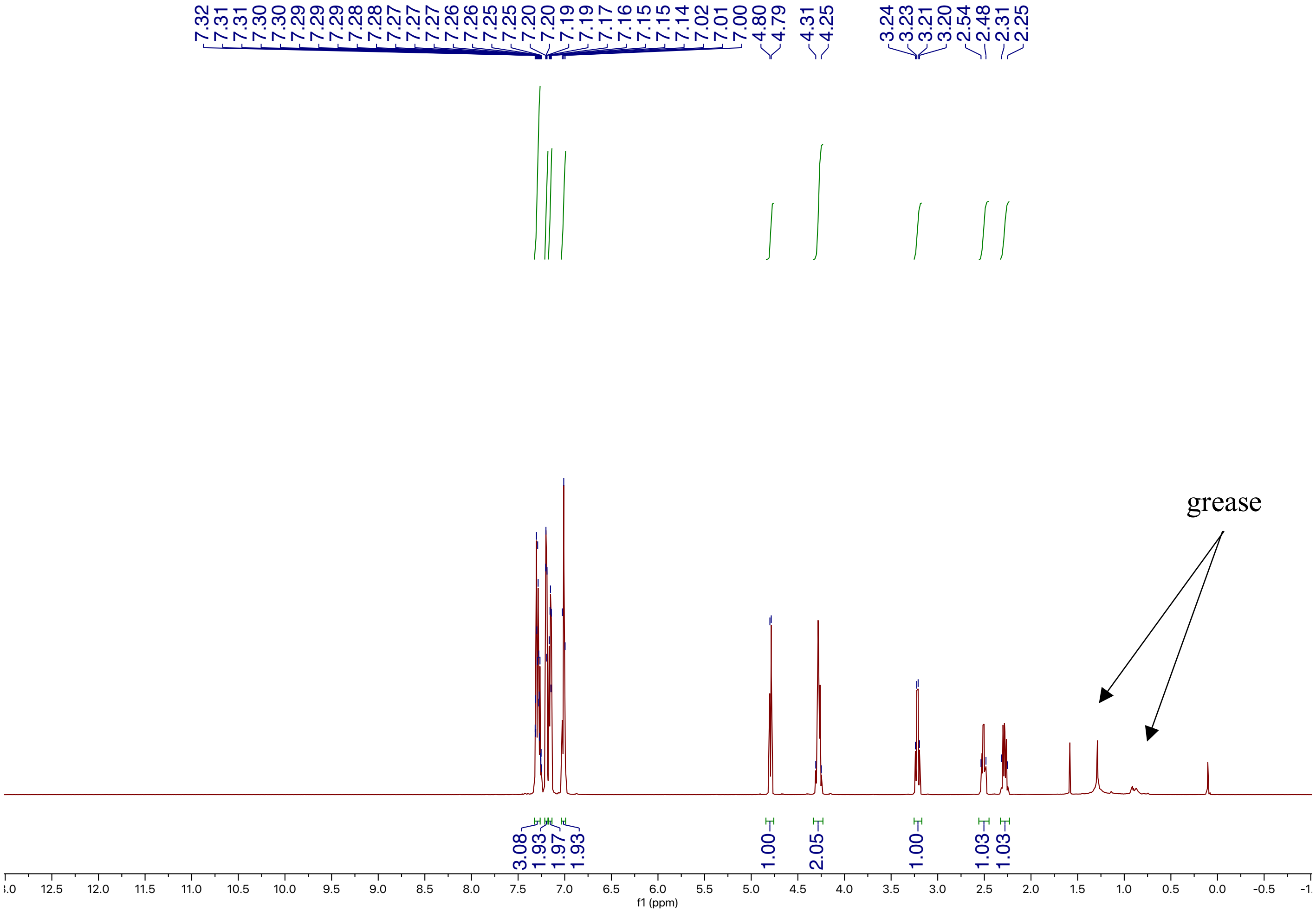

Compound 46 ^{19}F NMR

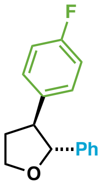

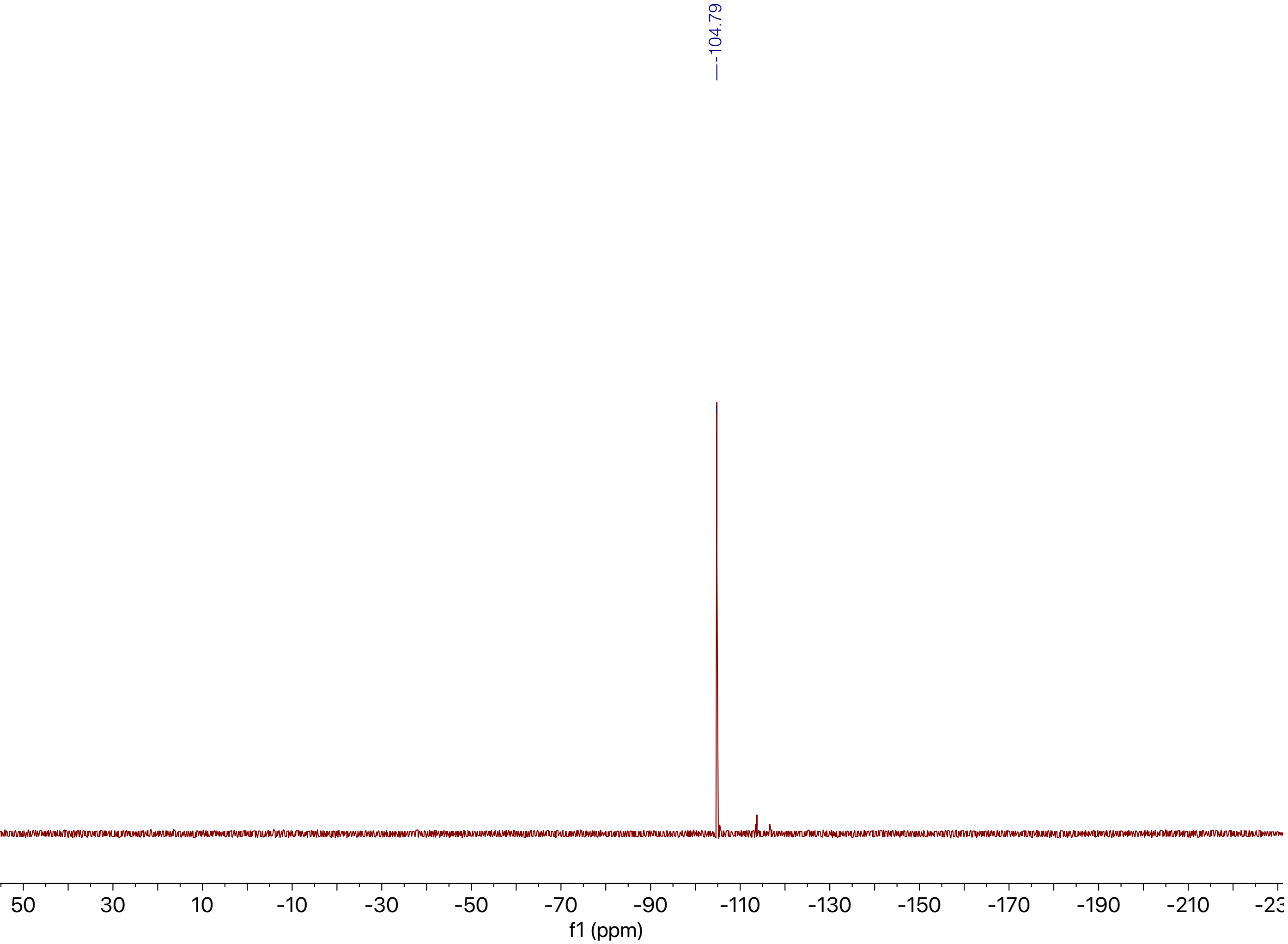

Compound 46 ^{13}C NMR

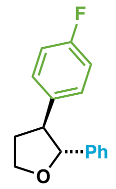

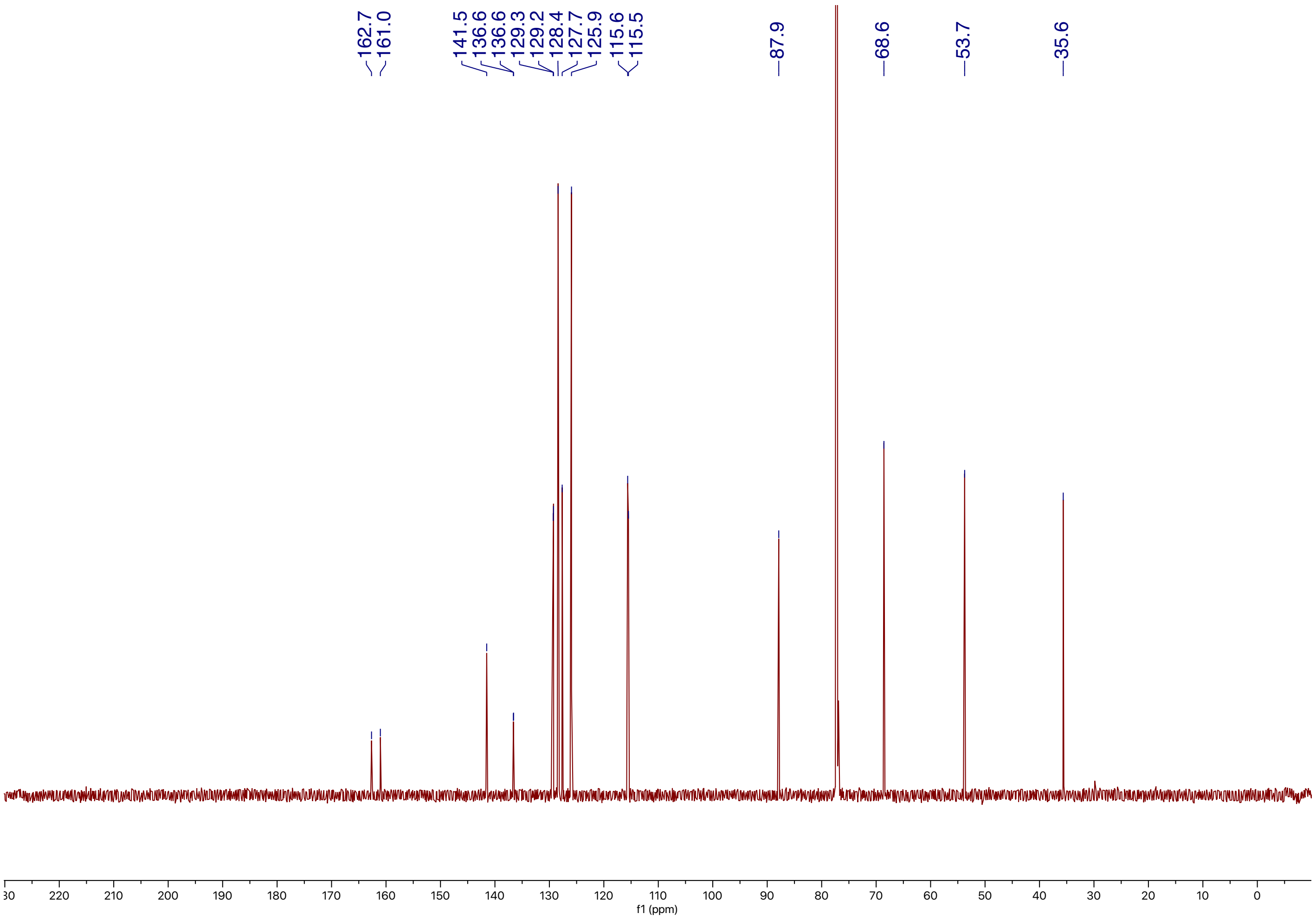

Compound 47 ^{1}H NMR

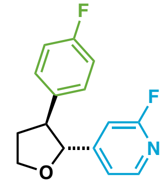

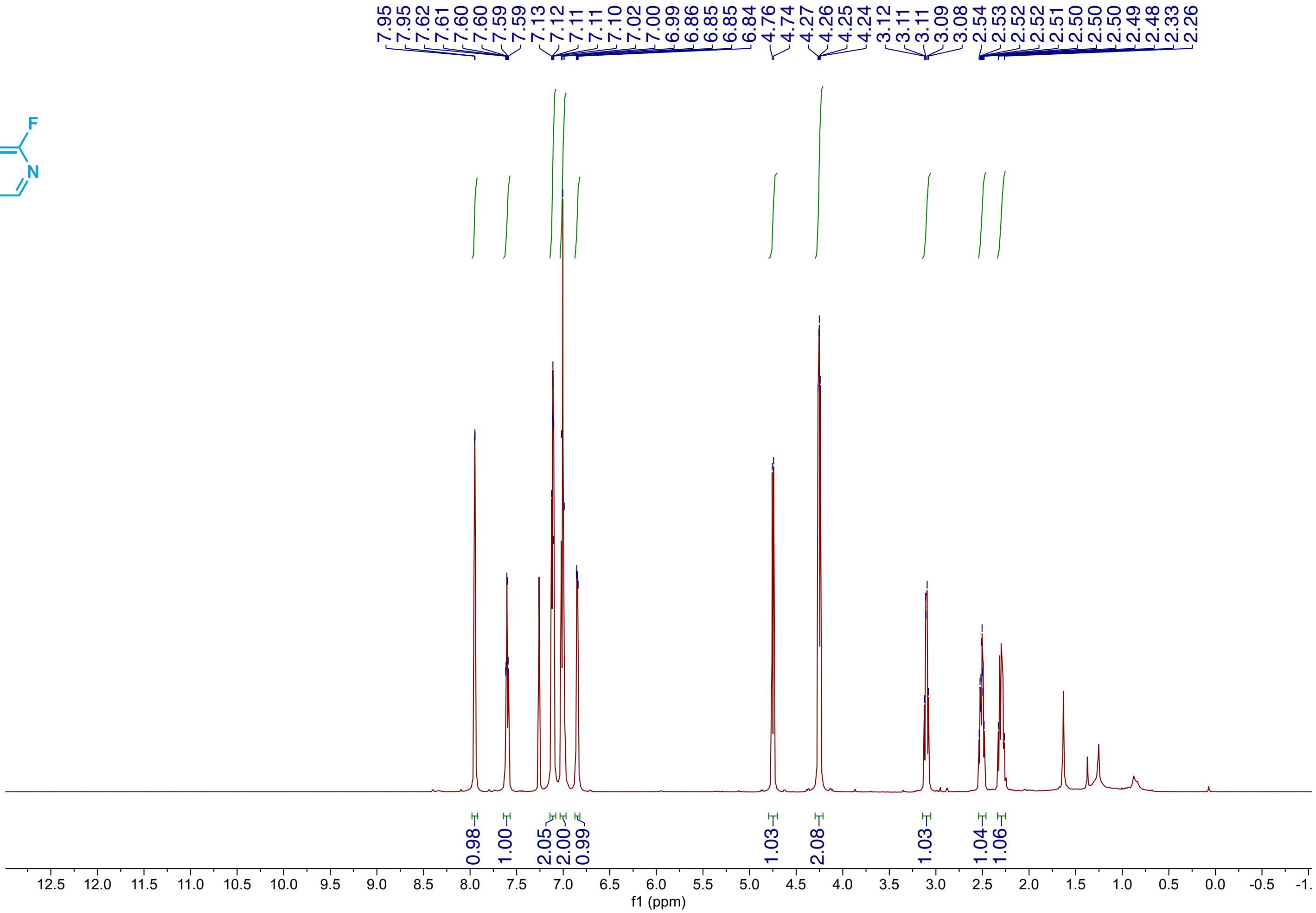

Compound 47 ^{19}F NMR

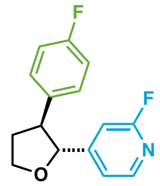

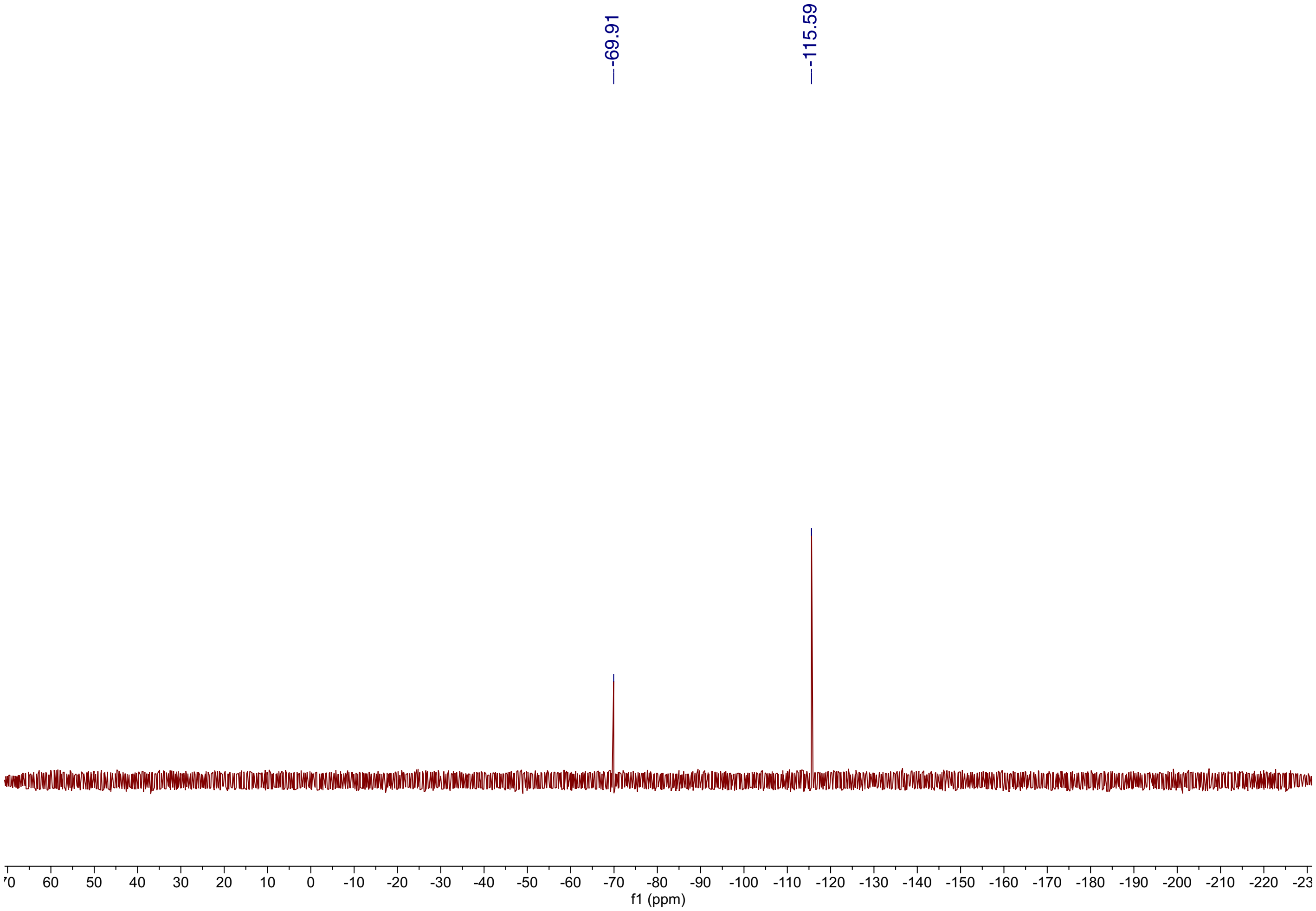

Compound 47 ^{13}C NMR

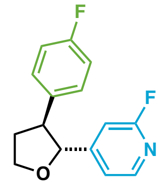

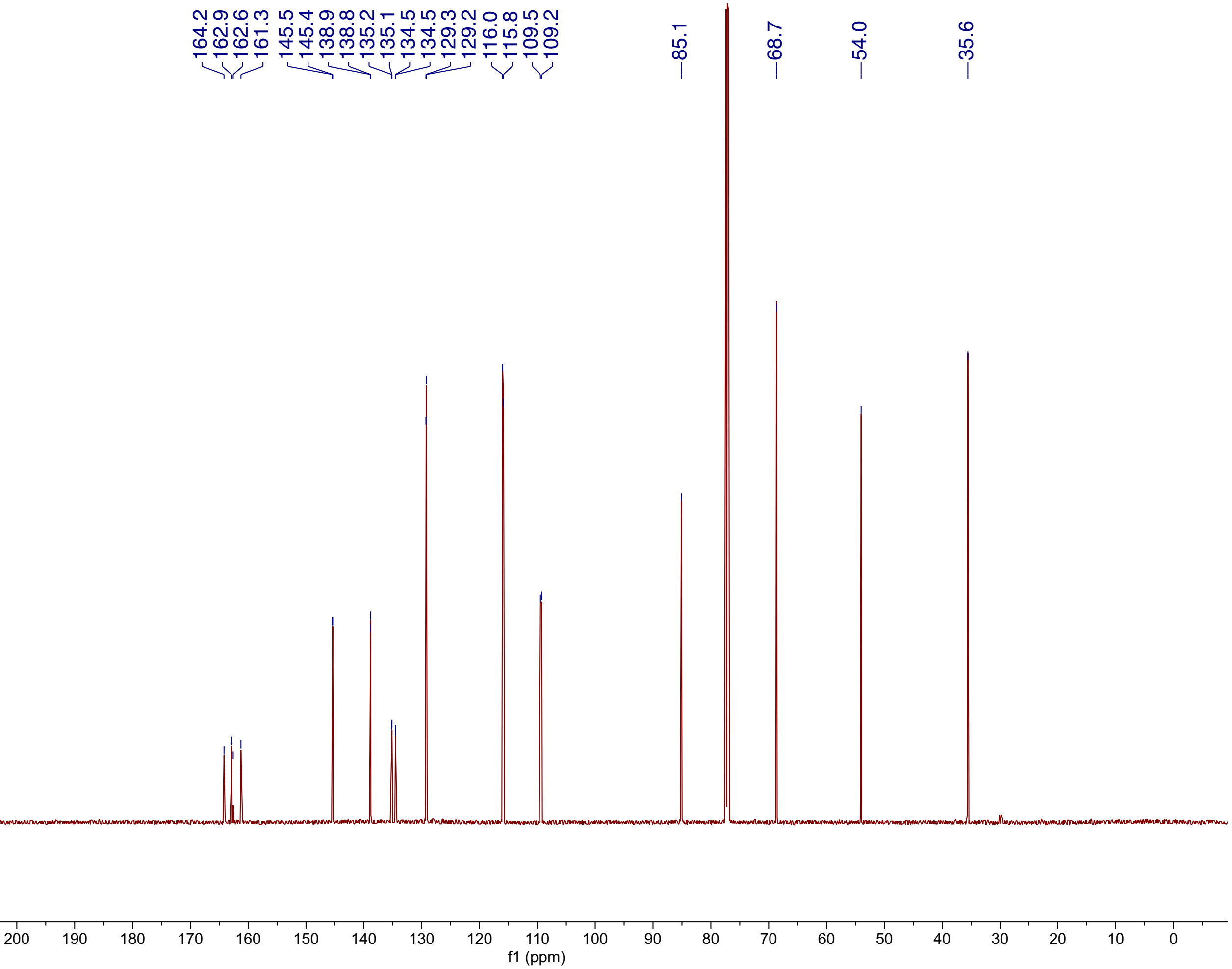

Compound 48 ^{1}H NMR

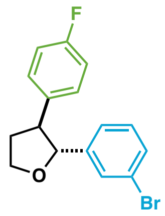

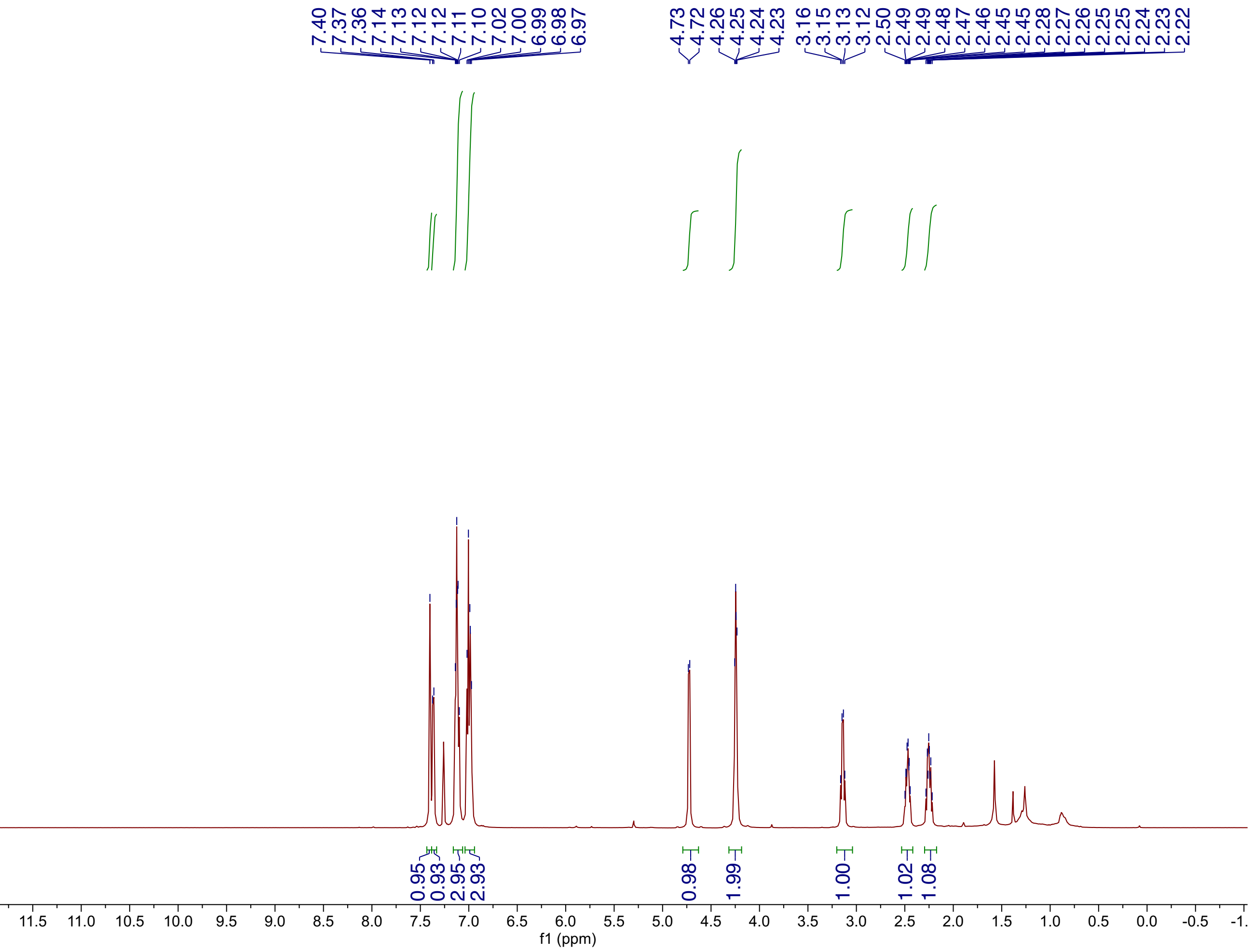

Compound 48 ^{19}F NMR

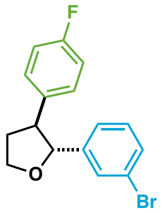

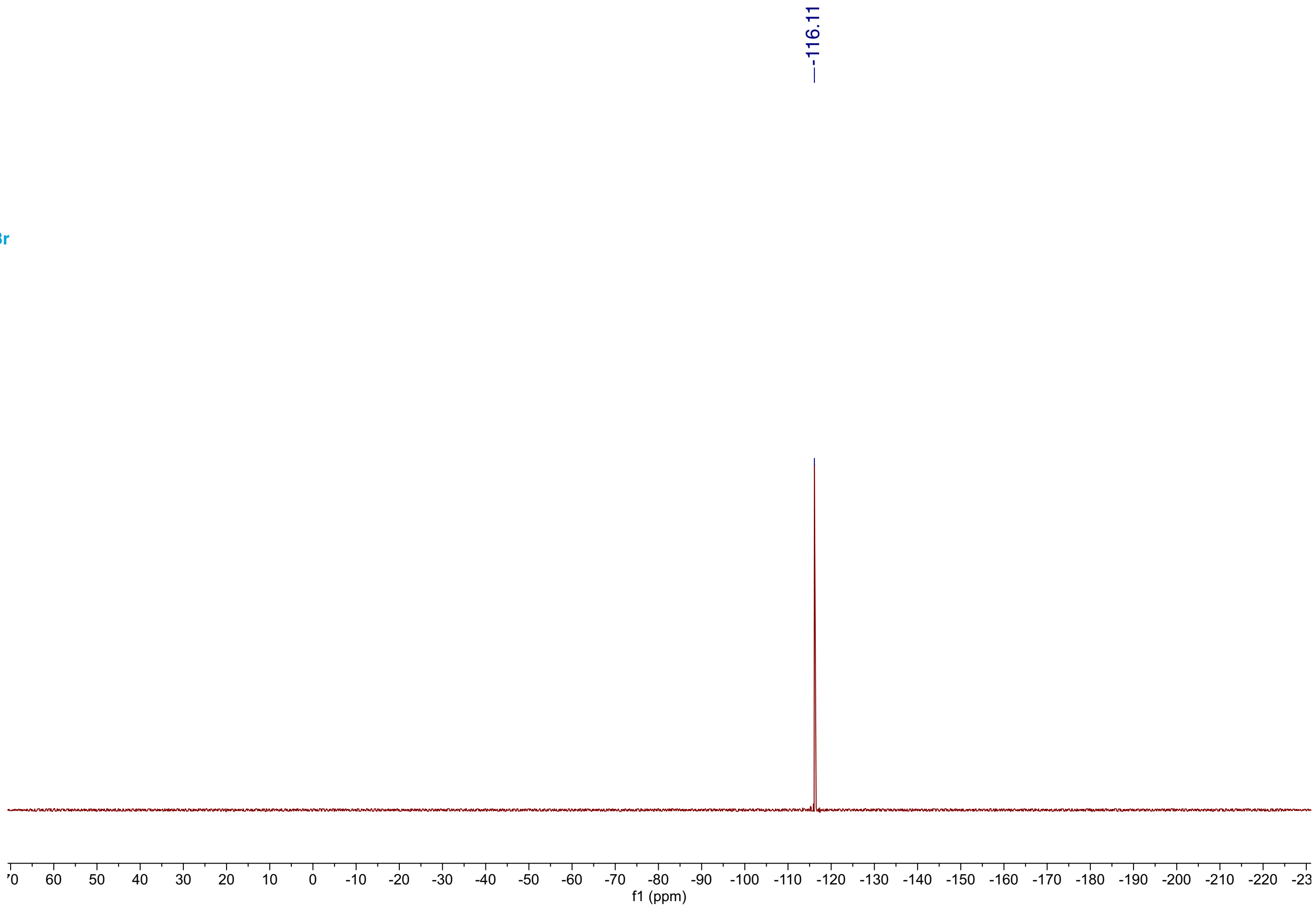

Compound 48 ^{13}C NMR

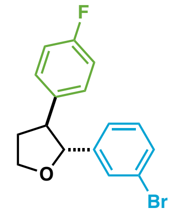

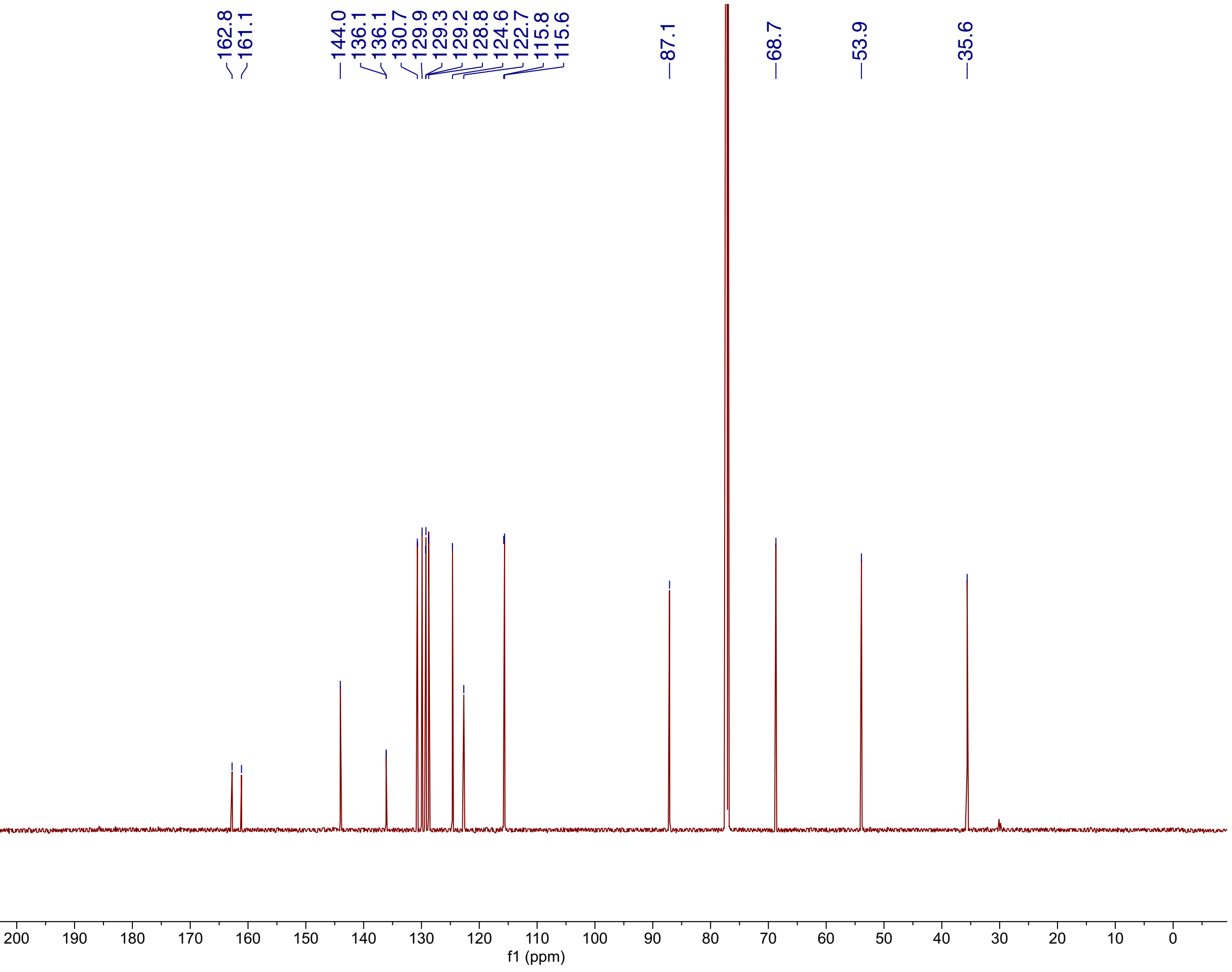

Compound 49 ^{1}H NMR

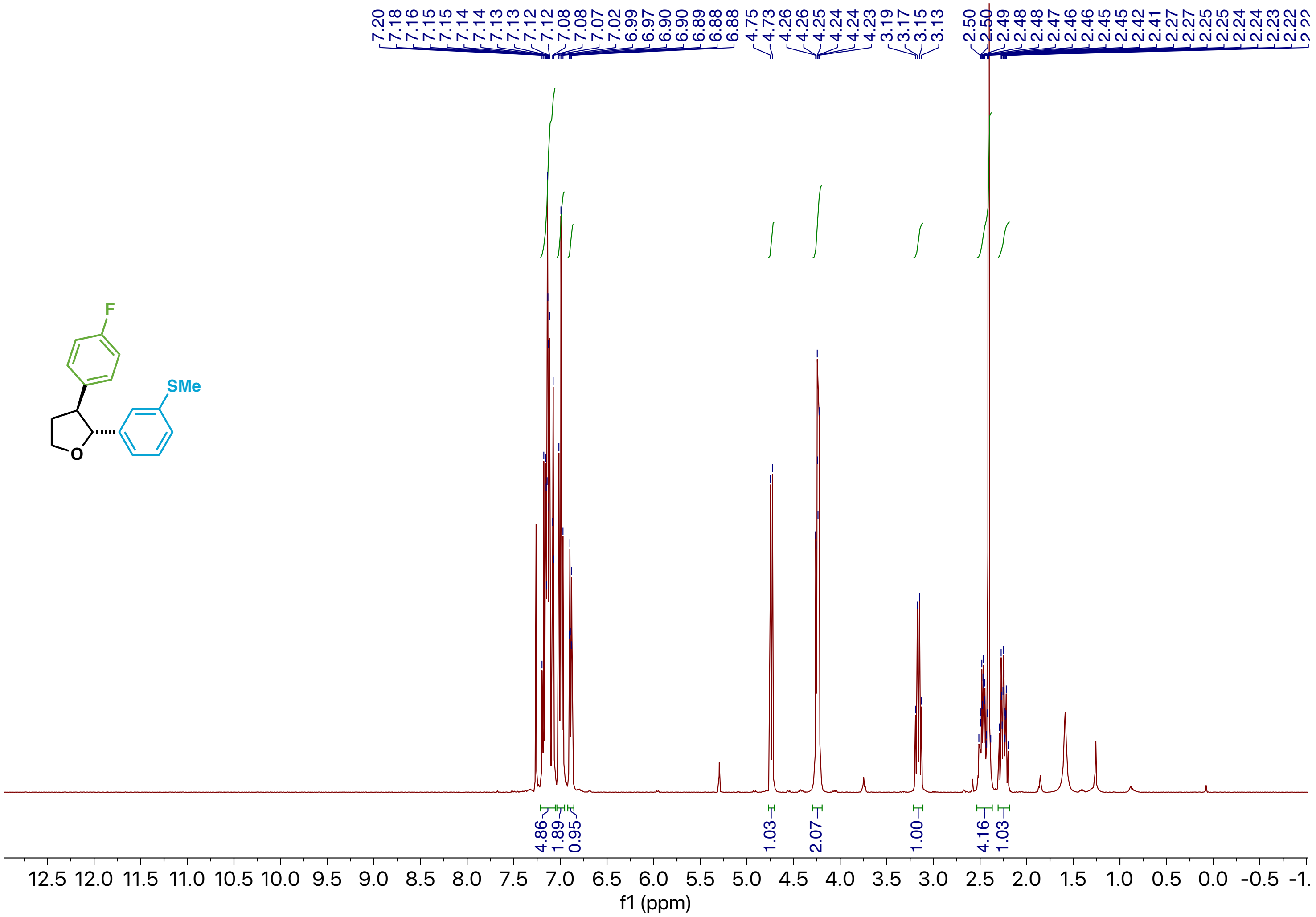

Compound 49 ^{19}F NMR

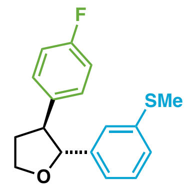

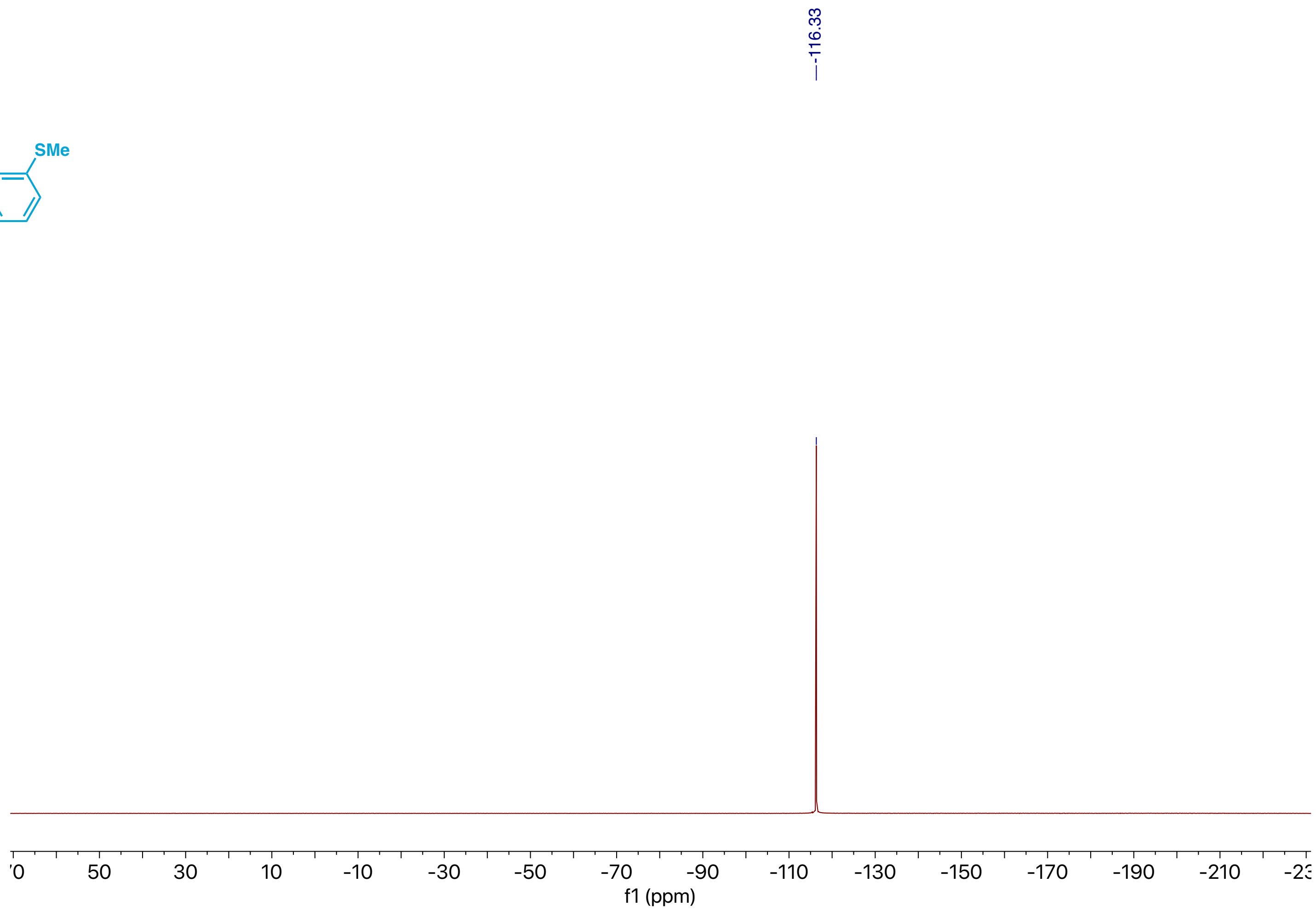

Compound 49 ^{13}C NMR

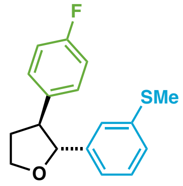

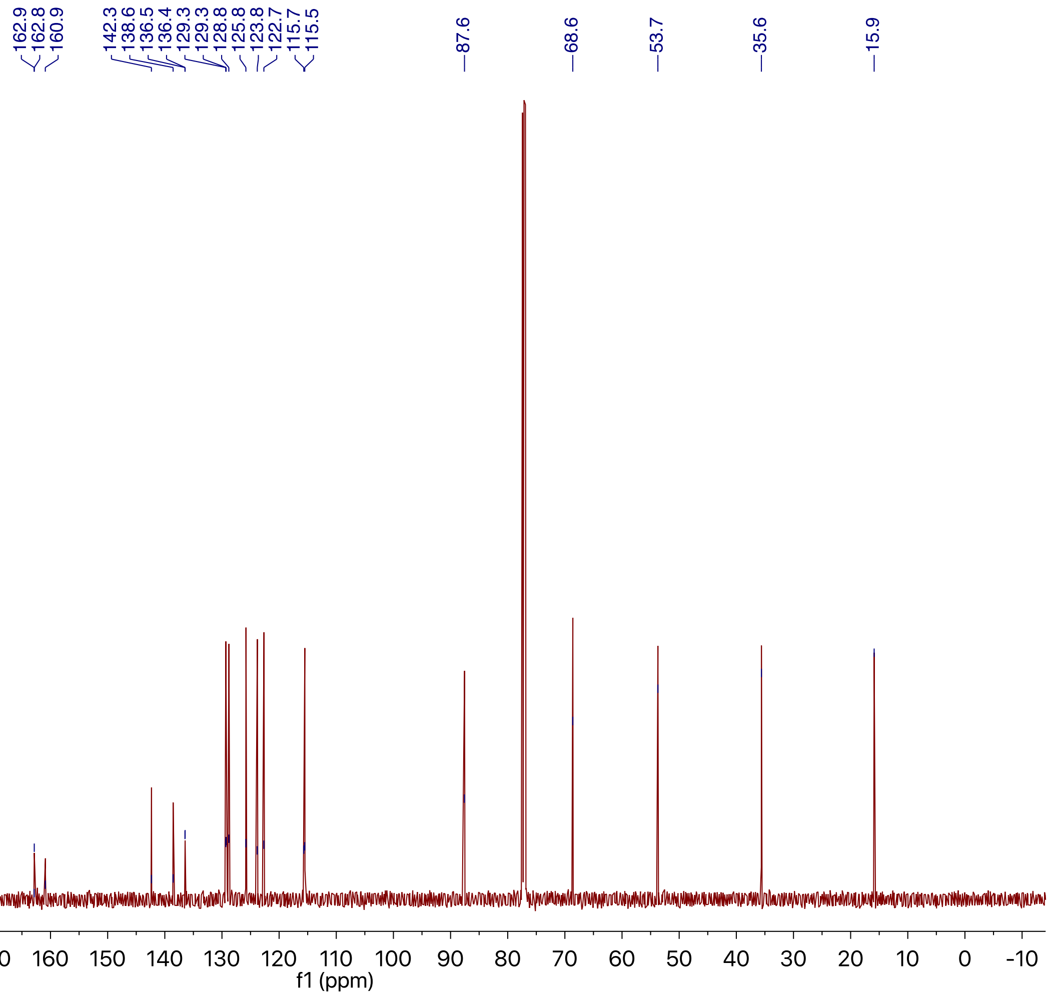

Compound 50 ^{1}H NMR

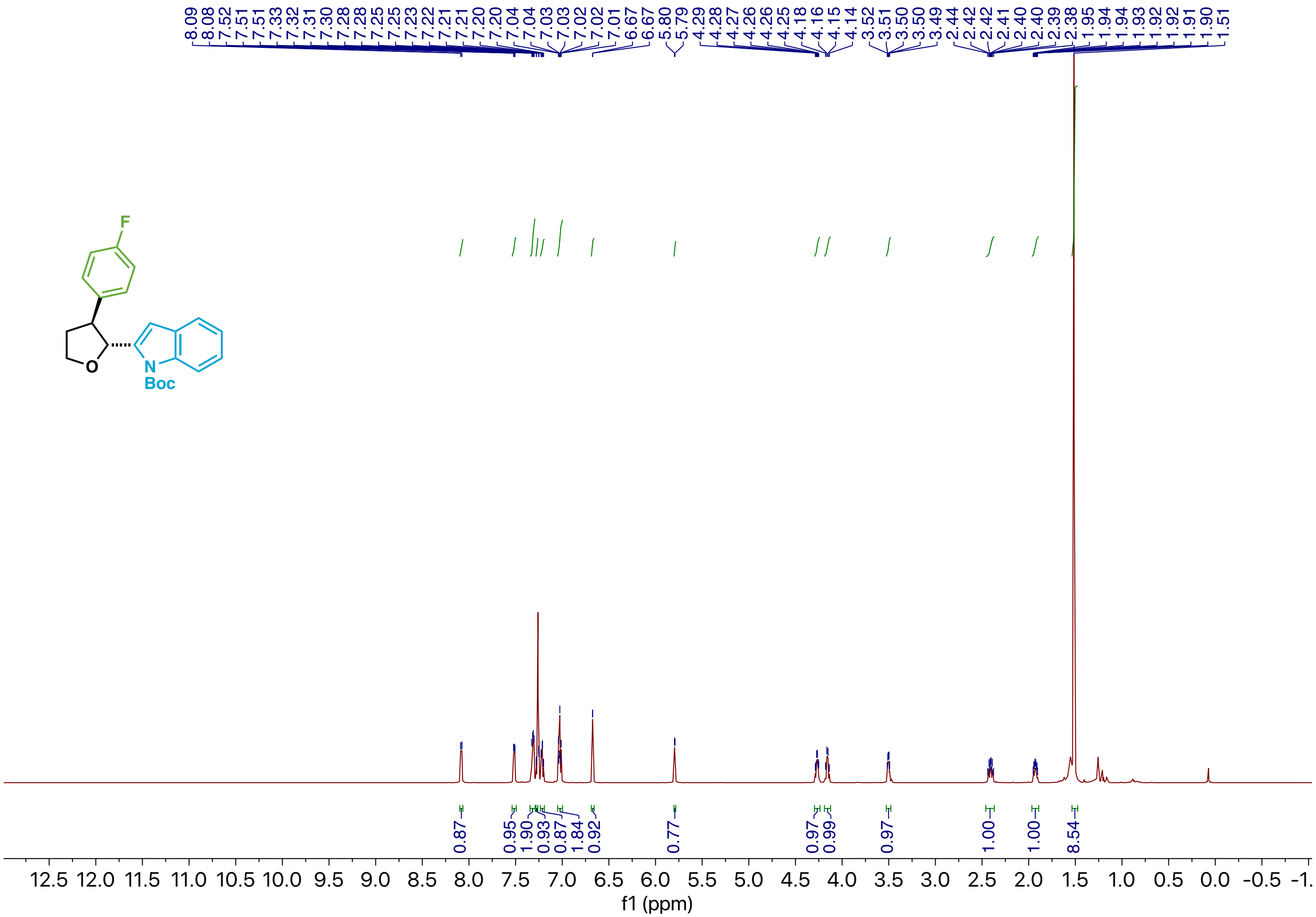

Compound 50 ^{19}F NMR

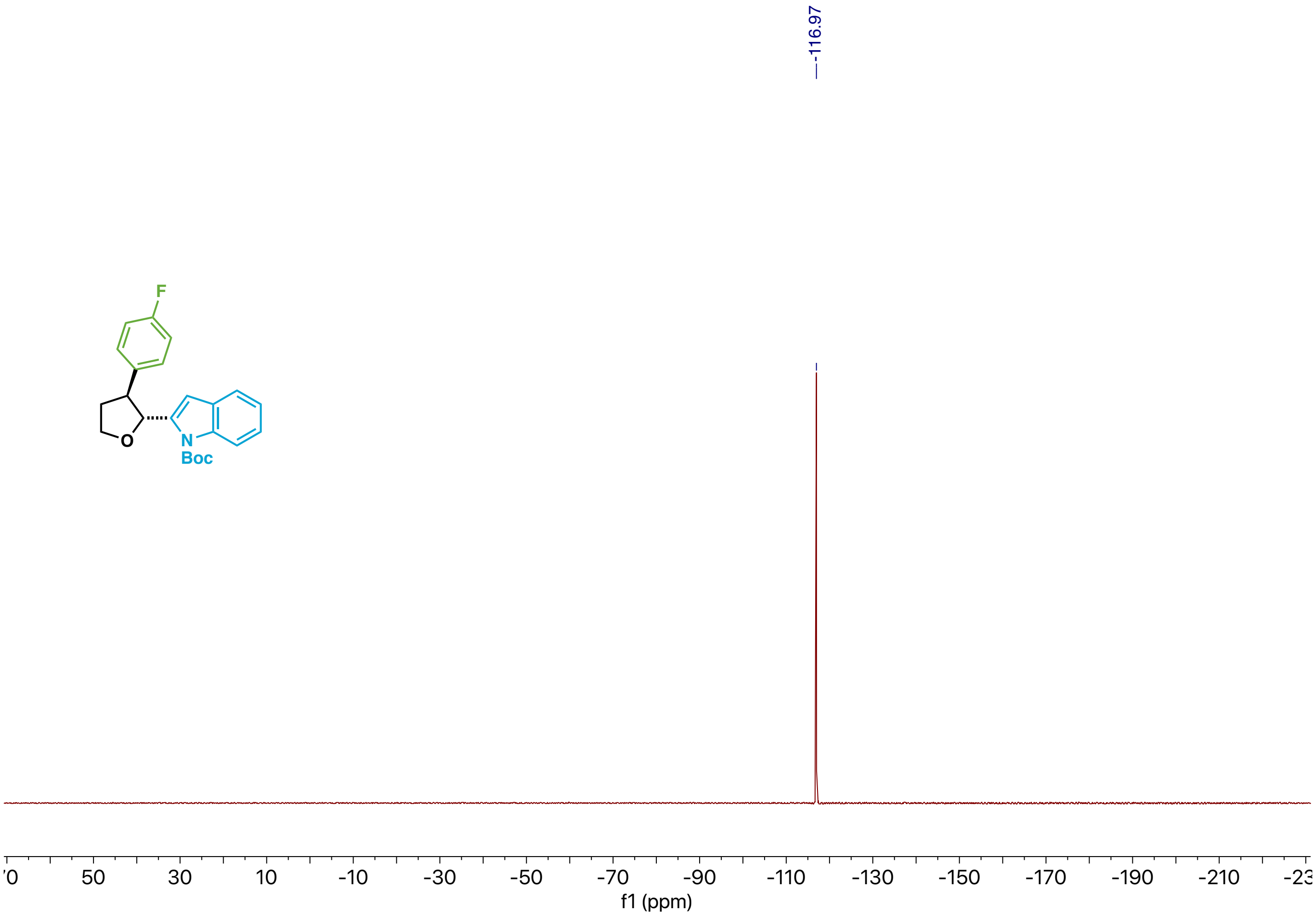

Compound 50 ^{13}C NMR

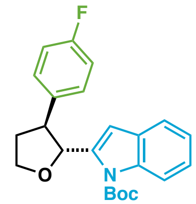

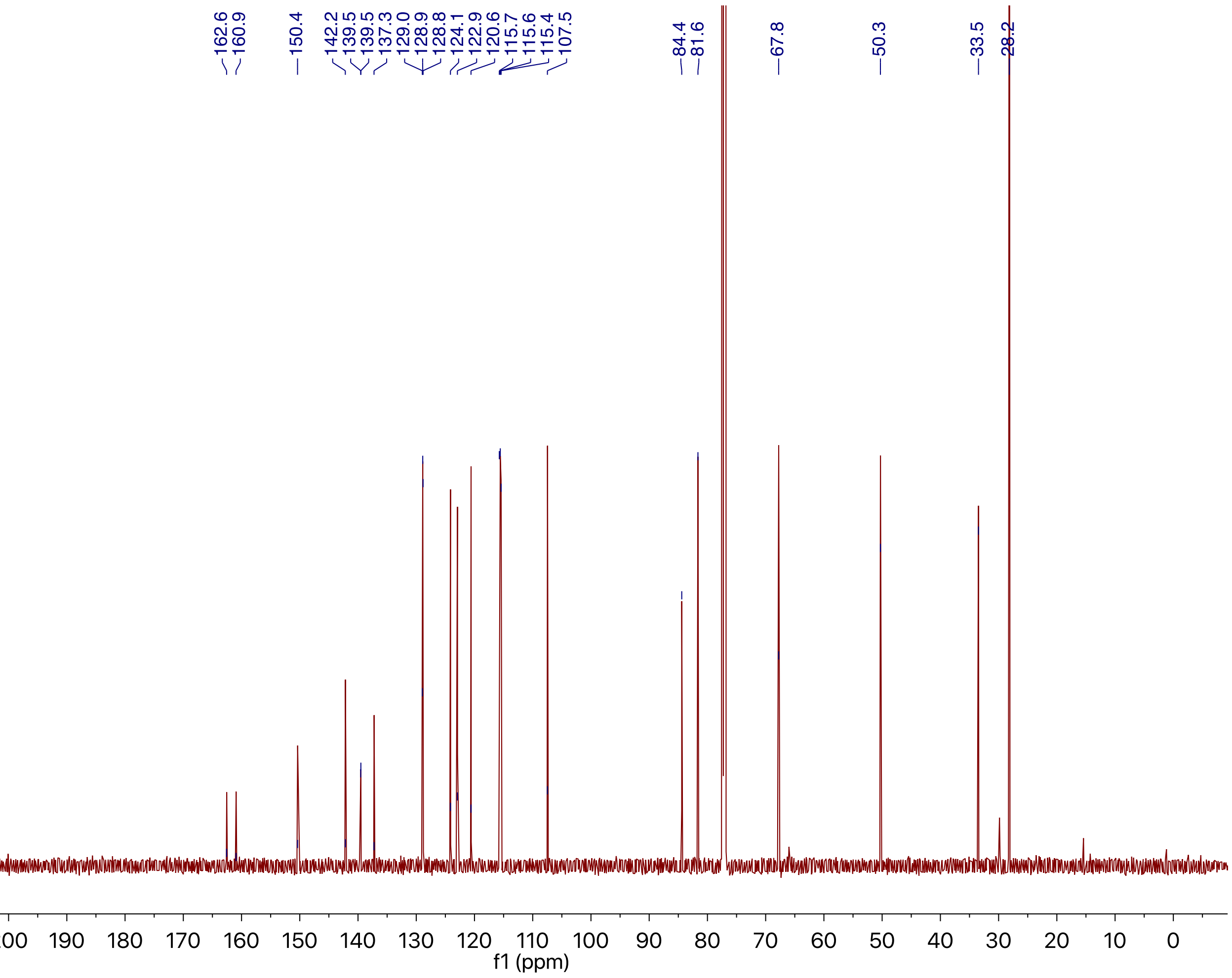

Compound 51 ^{1}H NMR

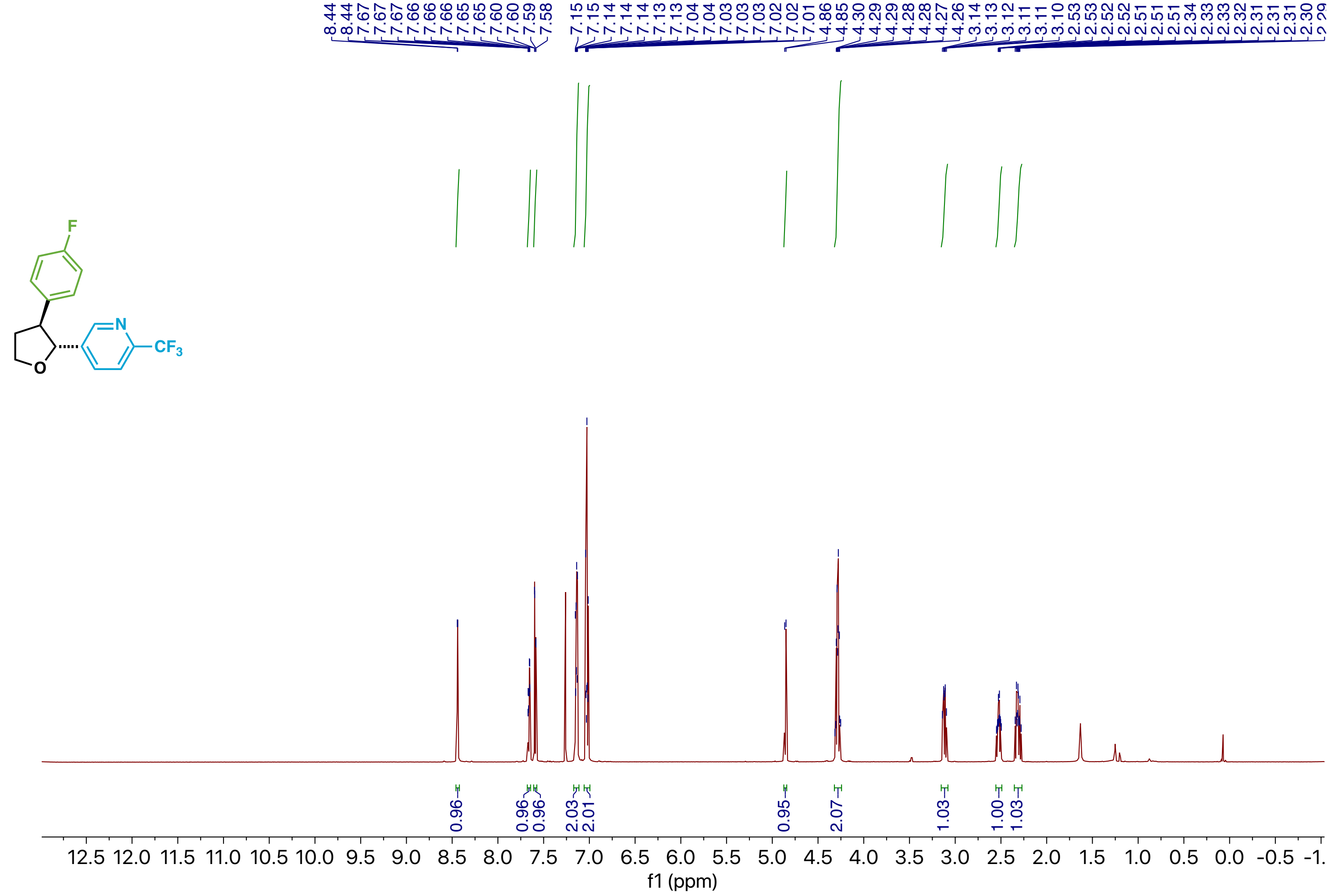

Compound 51 ^{19}F NMR

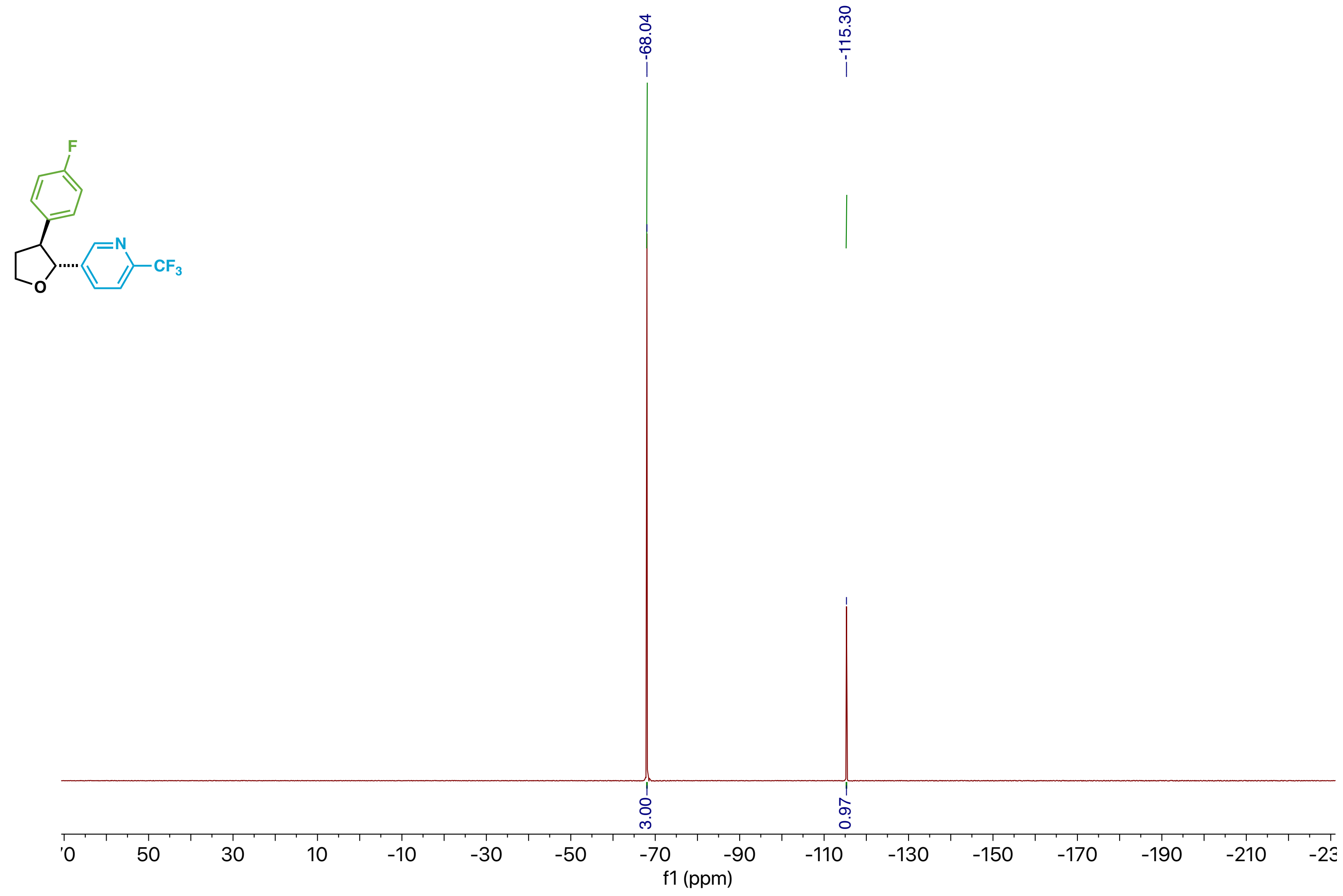

Compound 51 ^{13}C NMR

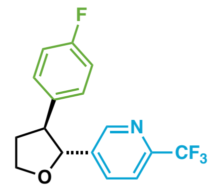

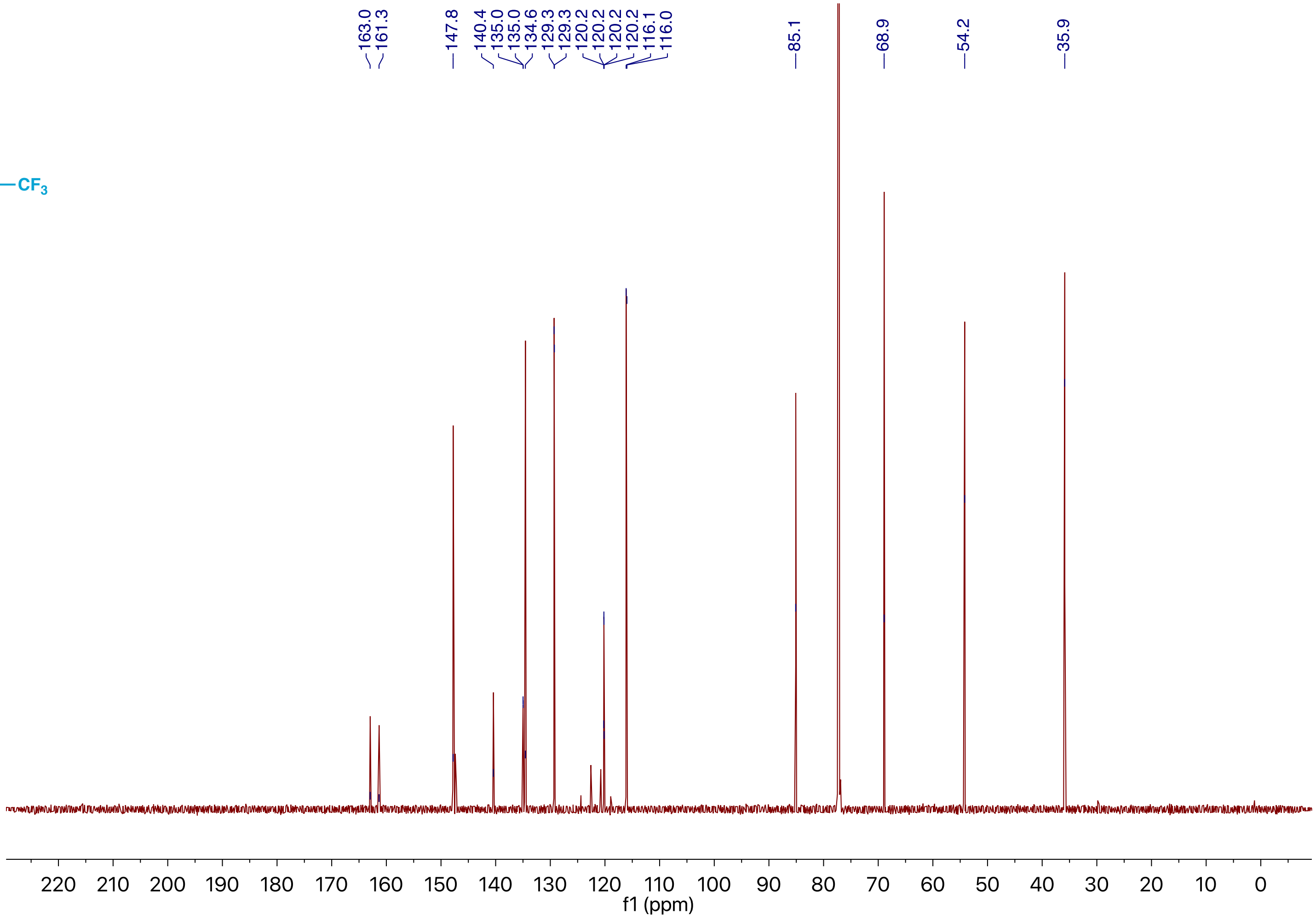

Compound 52 ^{1}H NMR

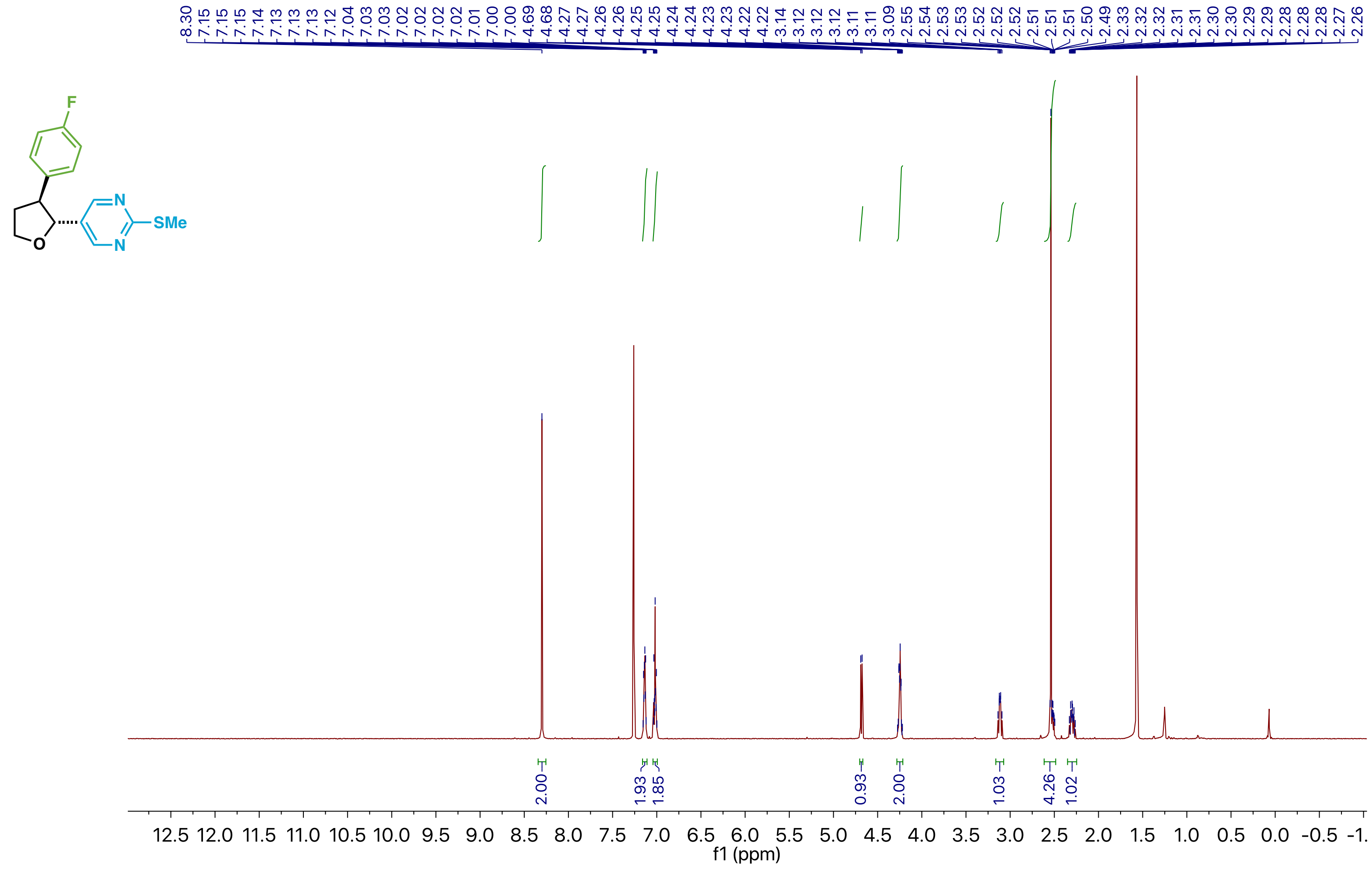

Compound 52 ^{19}F NMR

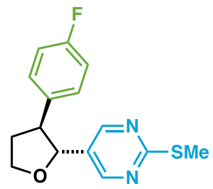

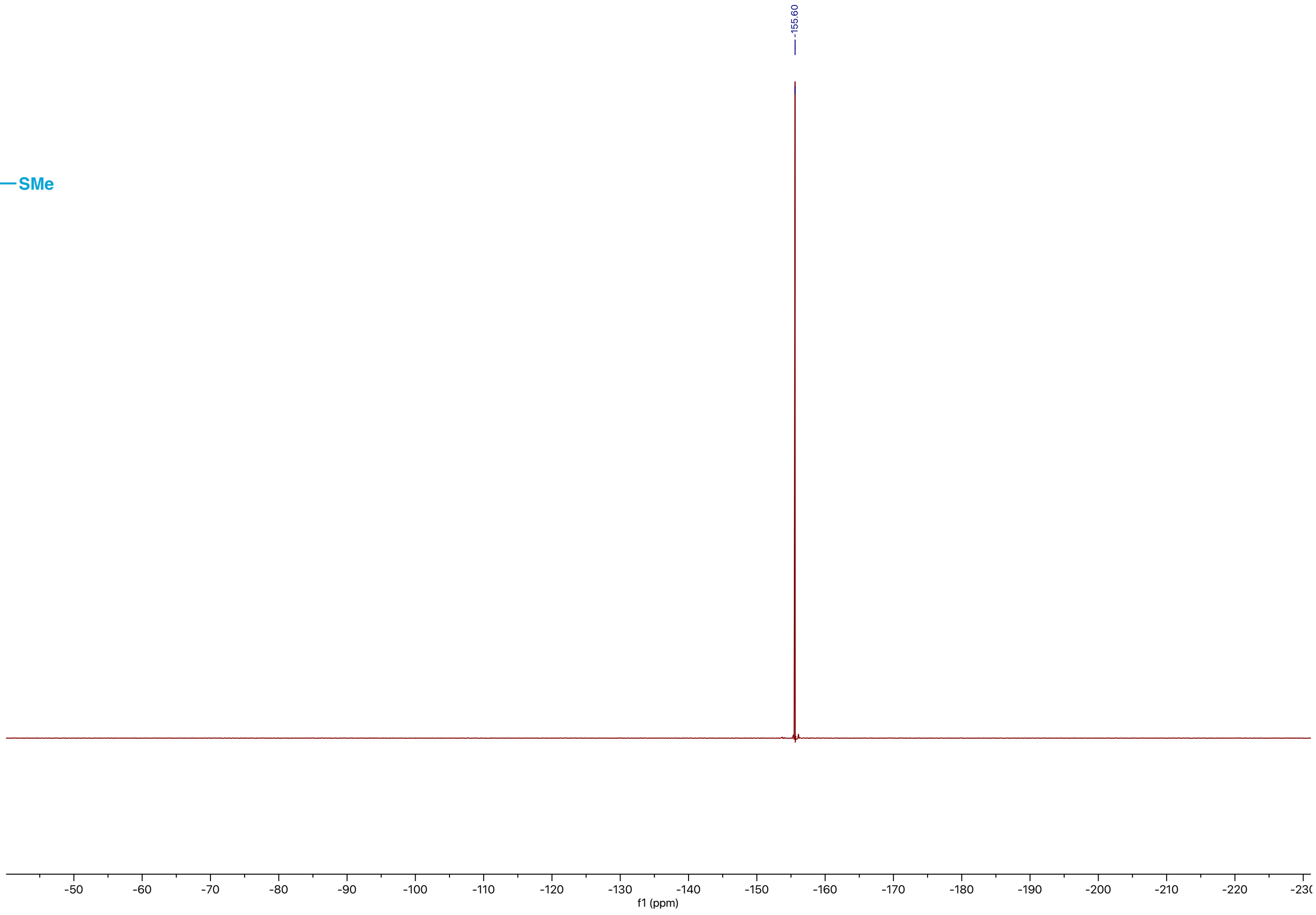

Compound 52 ^{13}C NMR

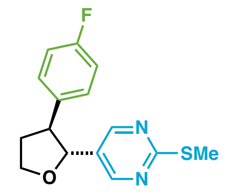

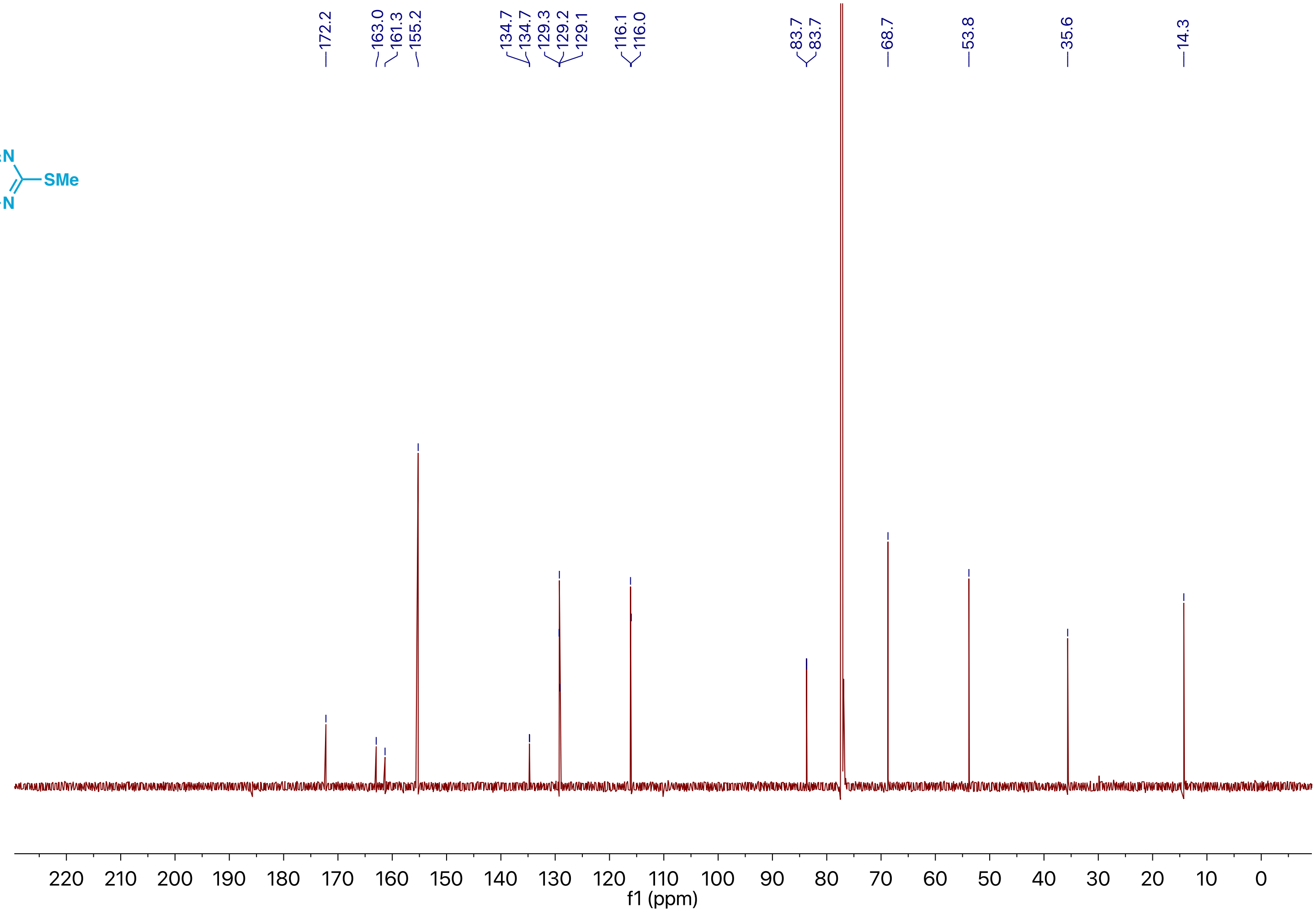

Compound B13 ^{1}H NMR

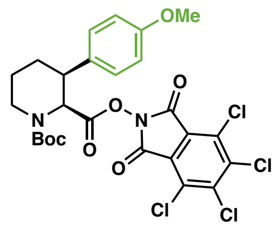

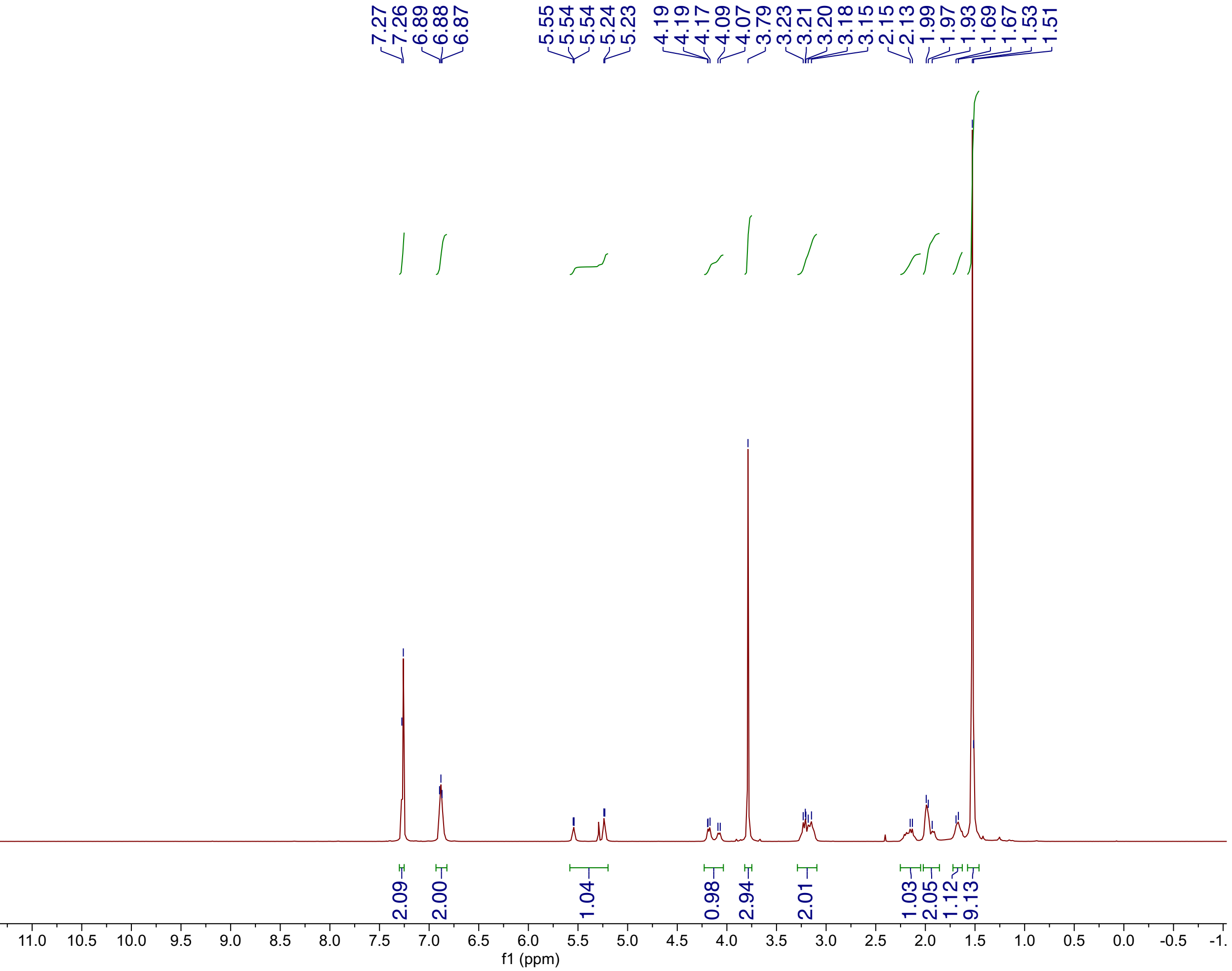

Compound B13 ^{13}C NMR

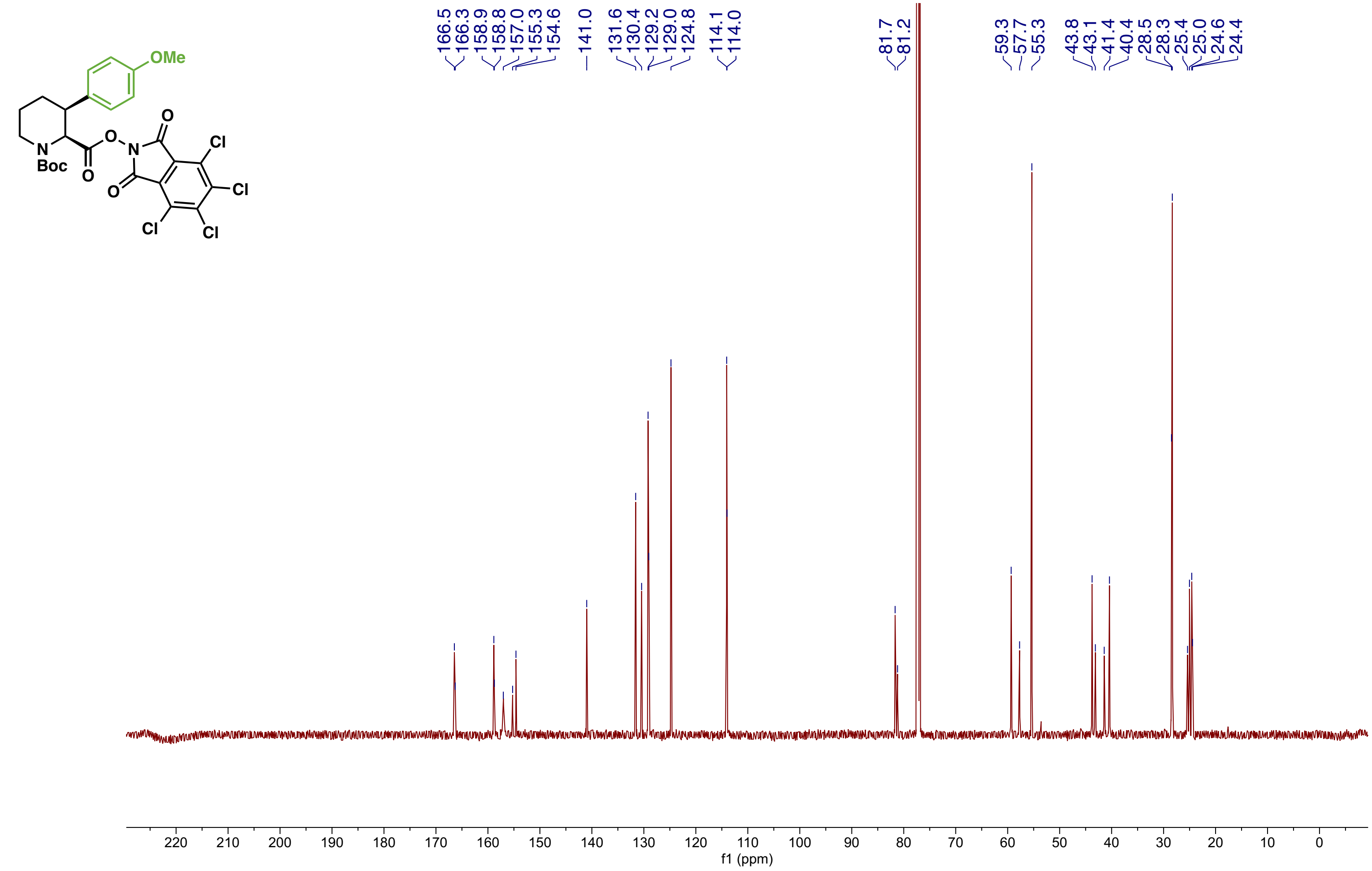

Compound 53 ^{1}H NMR

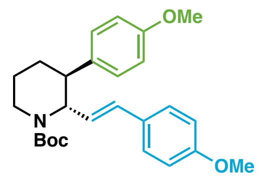

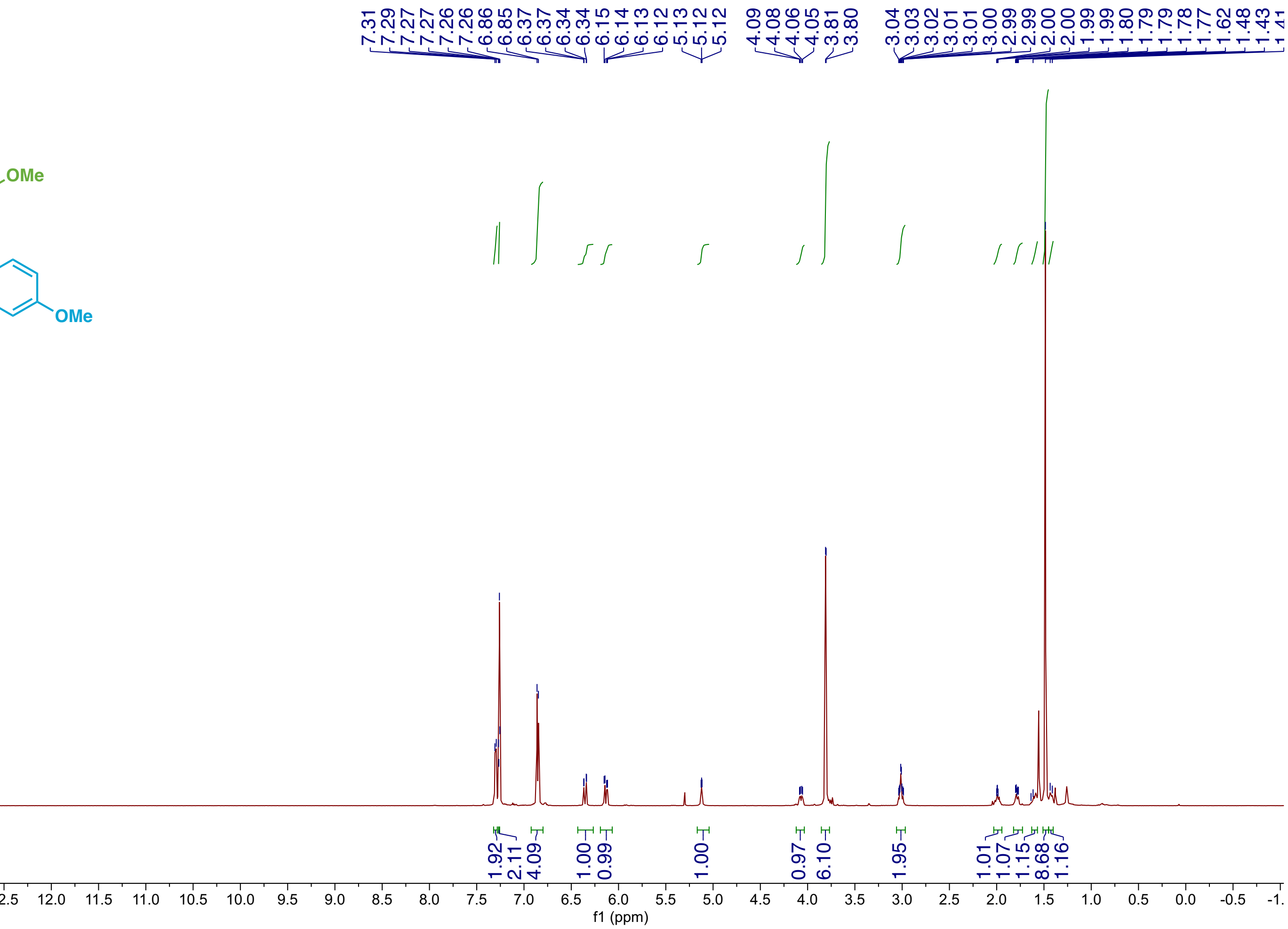

Compound 53 ^{13}C NMR

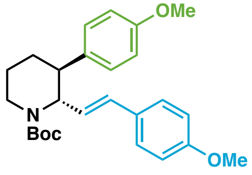

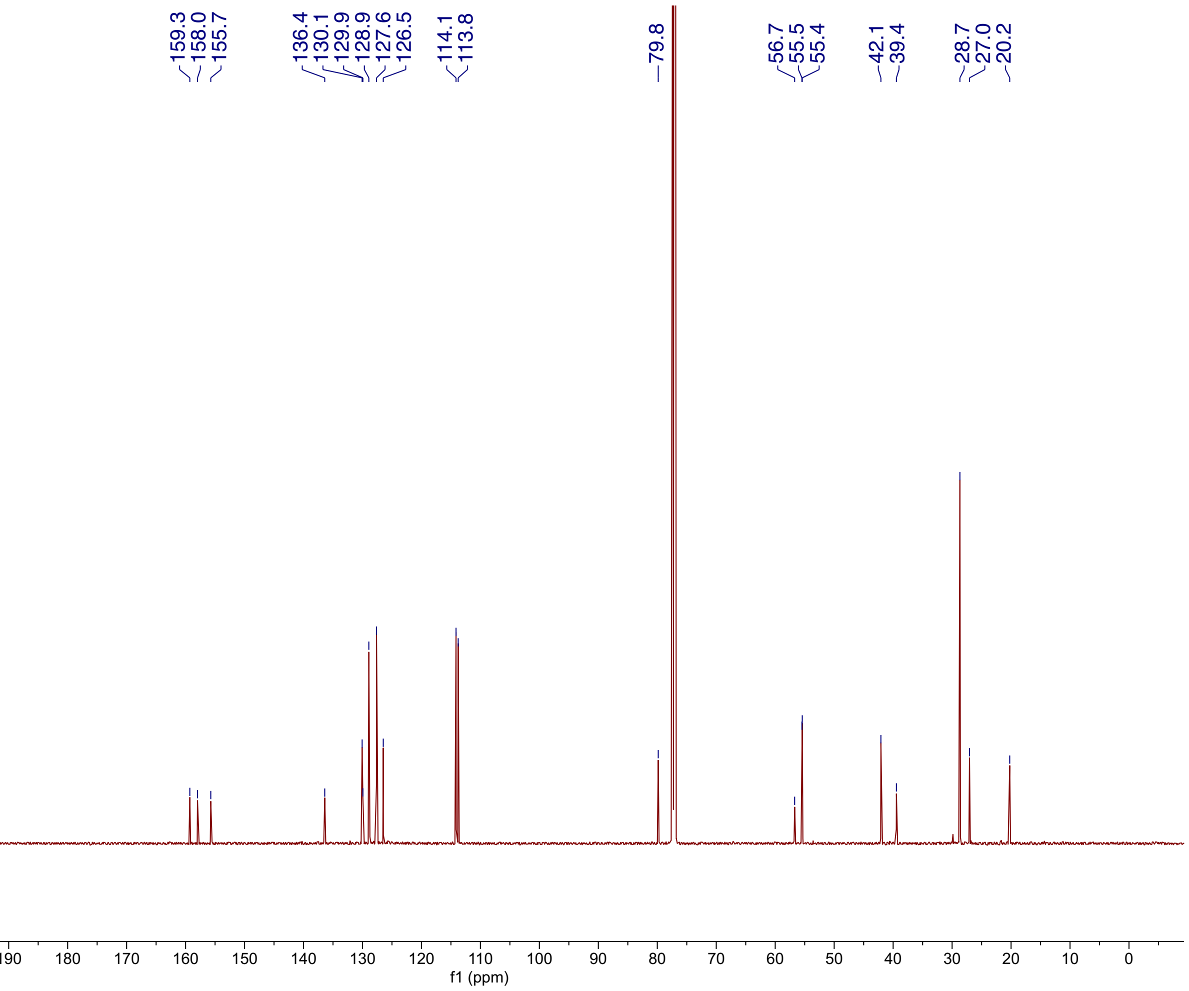

Compound 54 ^{1}H NMR

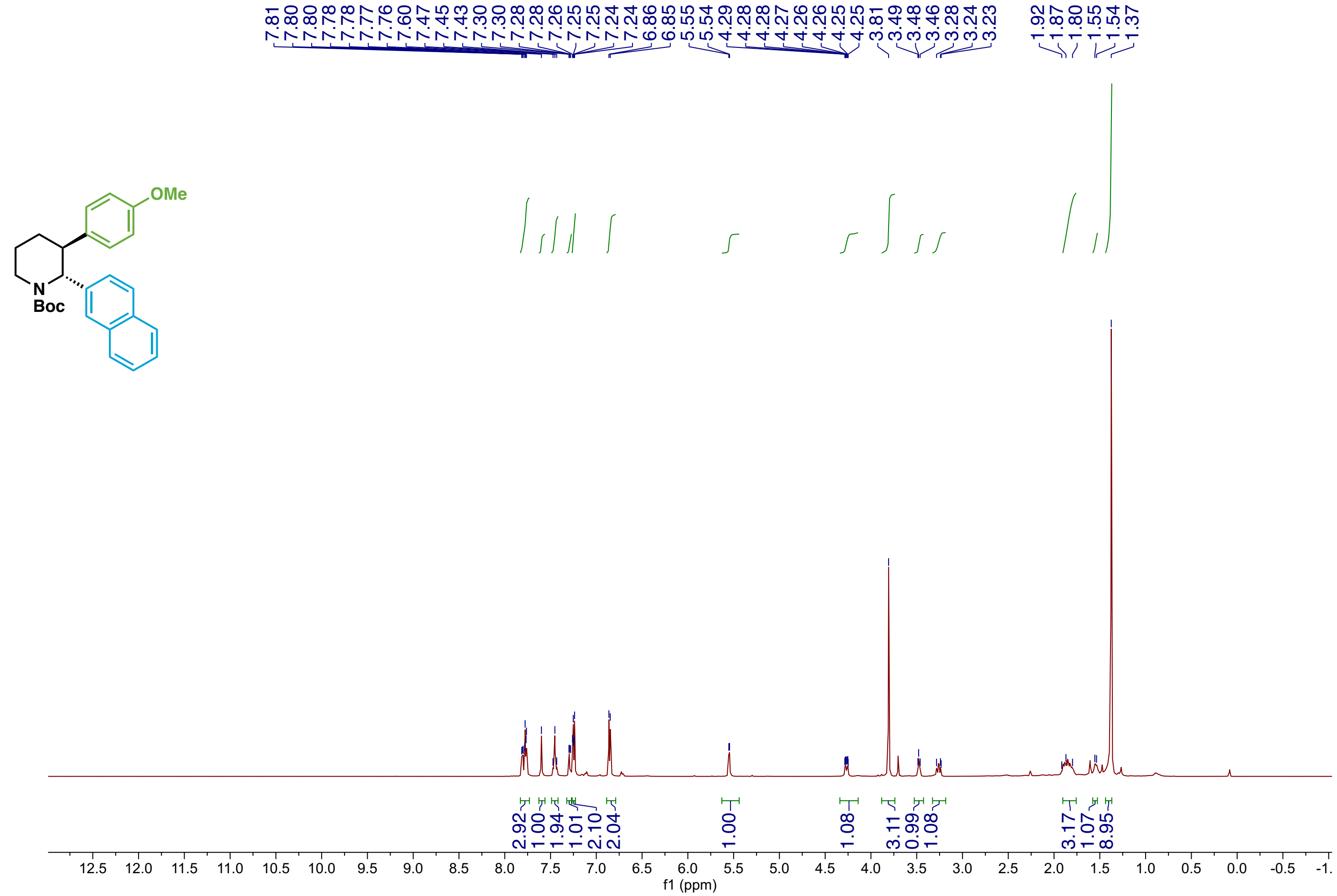

Compound 54 ^{13}C NMR

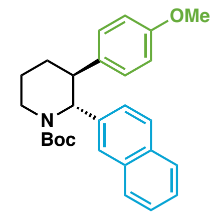

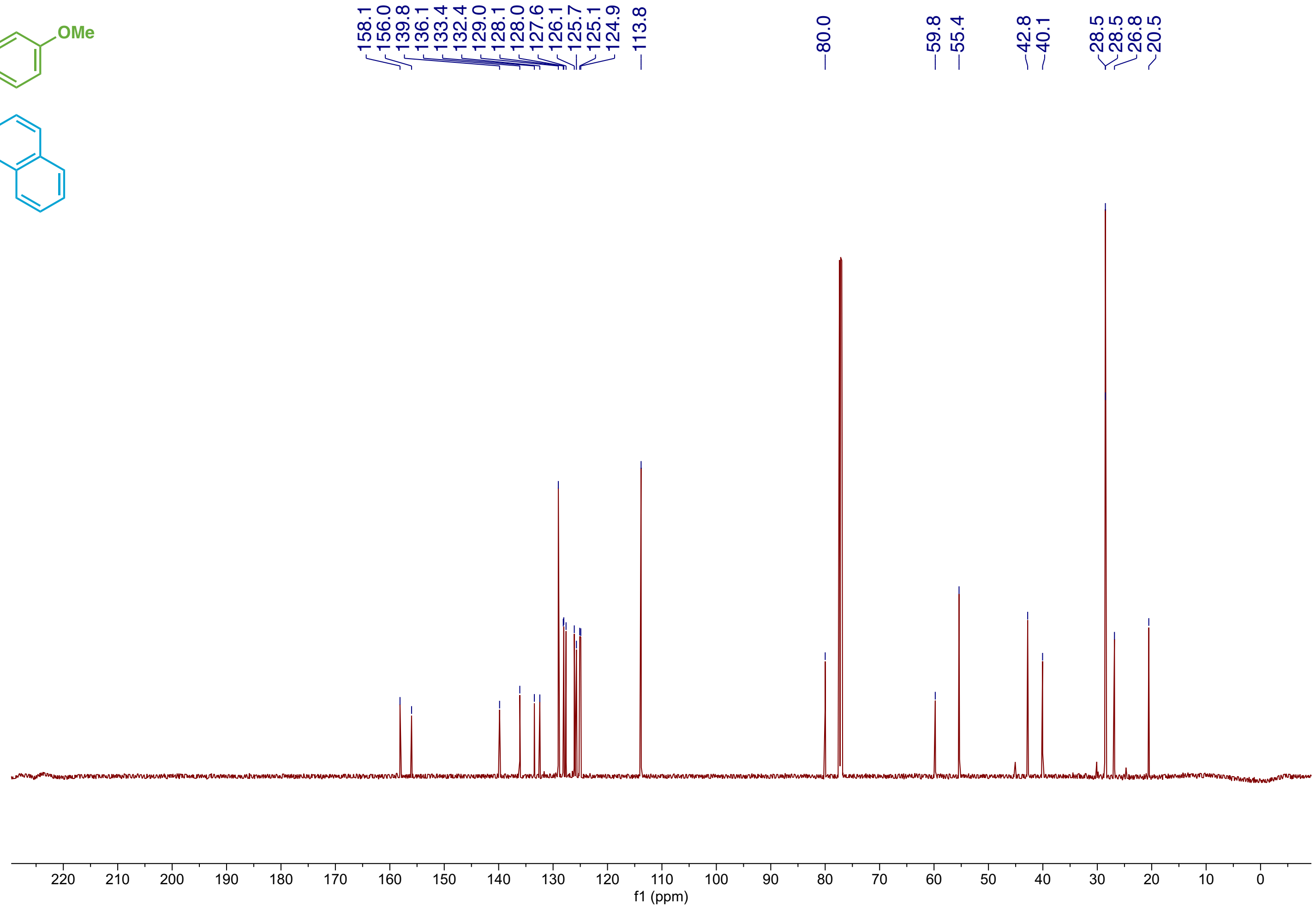

Compound 55 ^{1}H NMR

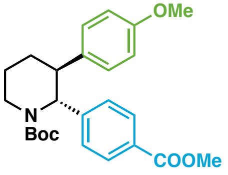

`

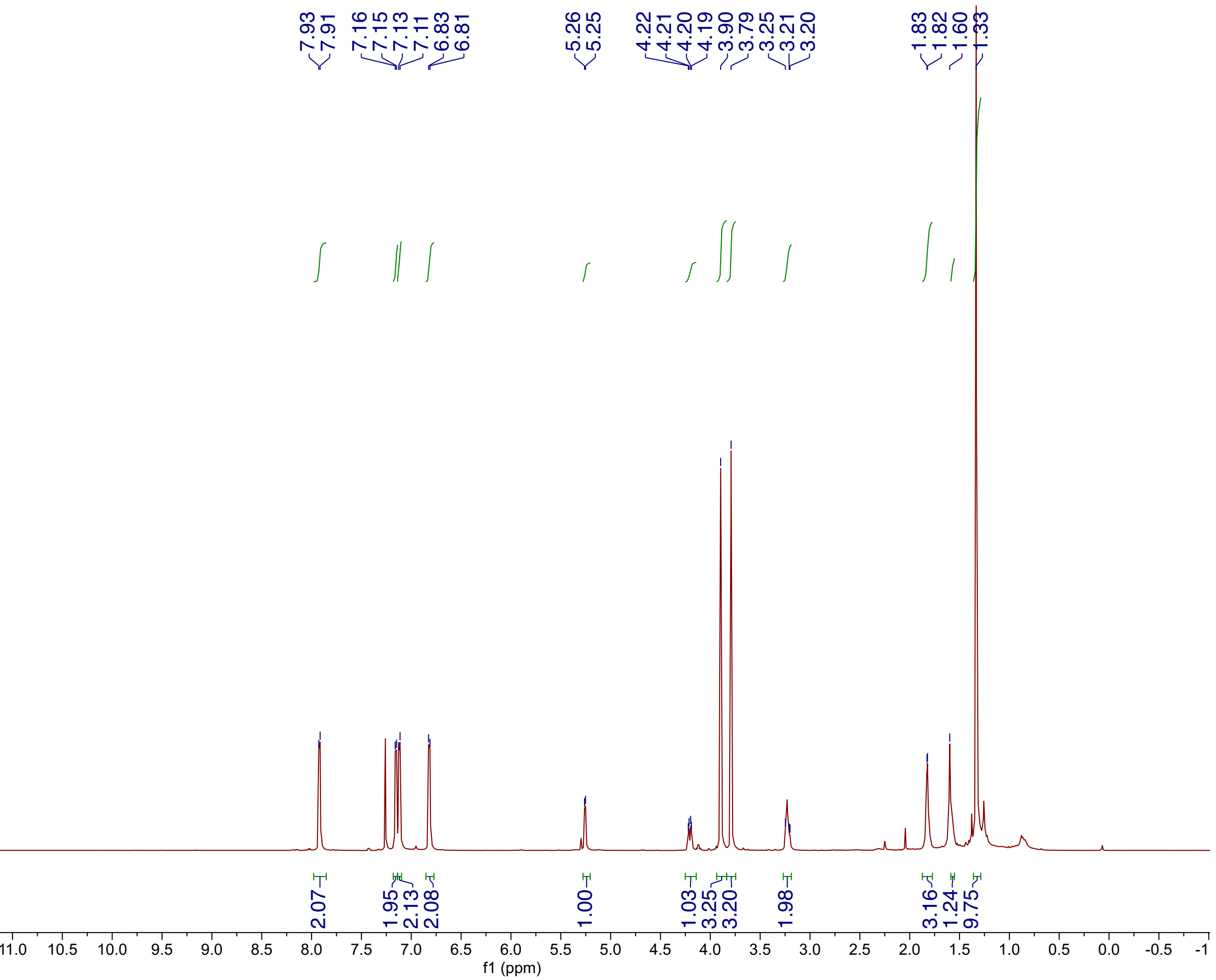

Compound 55 ^{13}C NMR

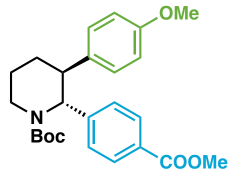

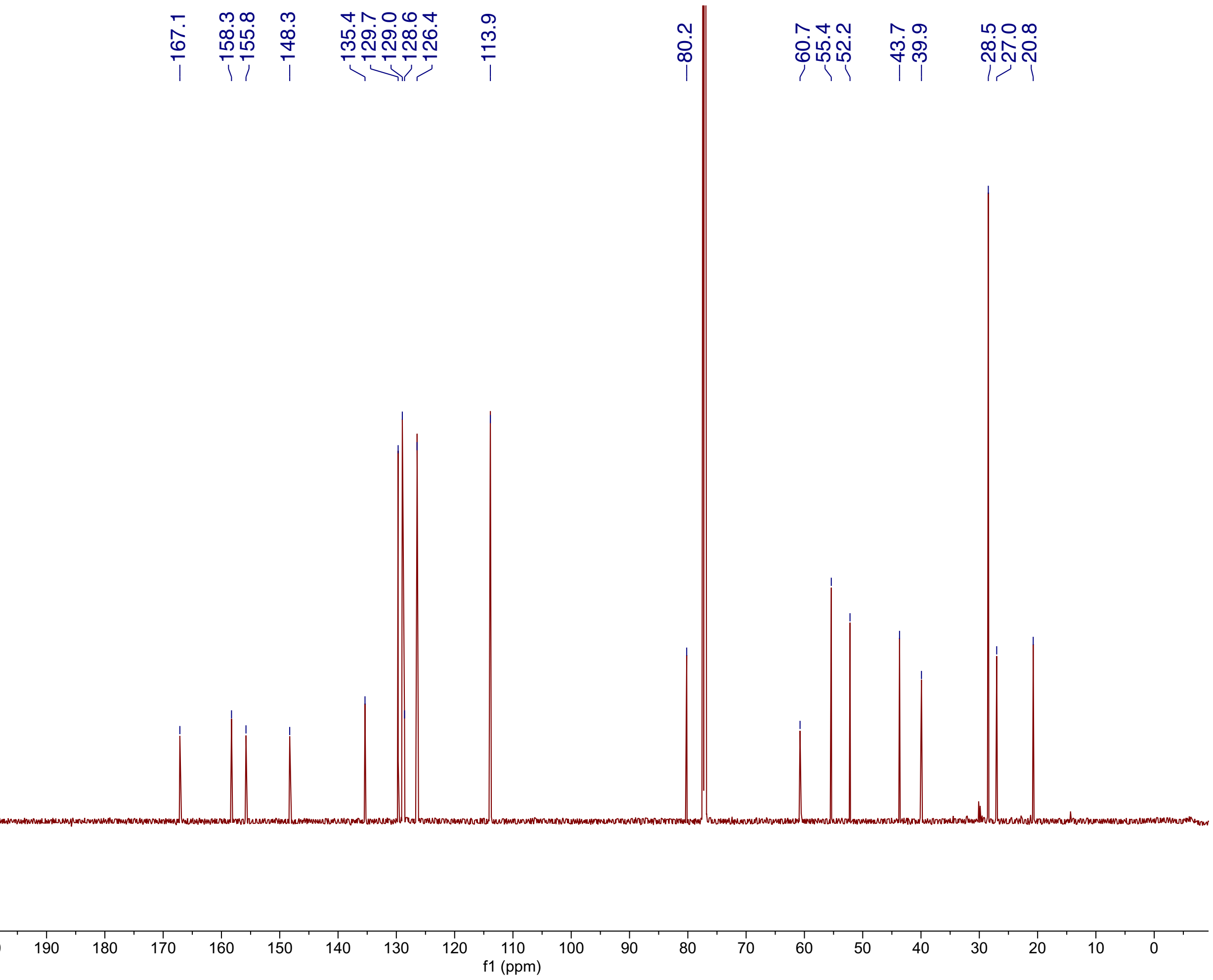

Compound 56 ^{1}H NMR

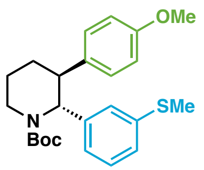

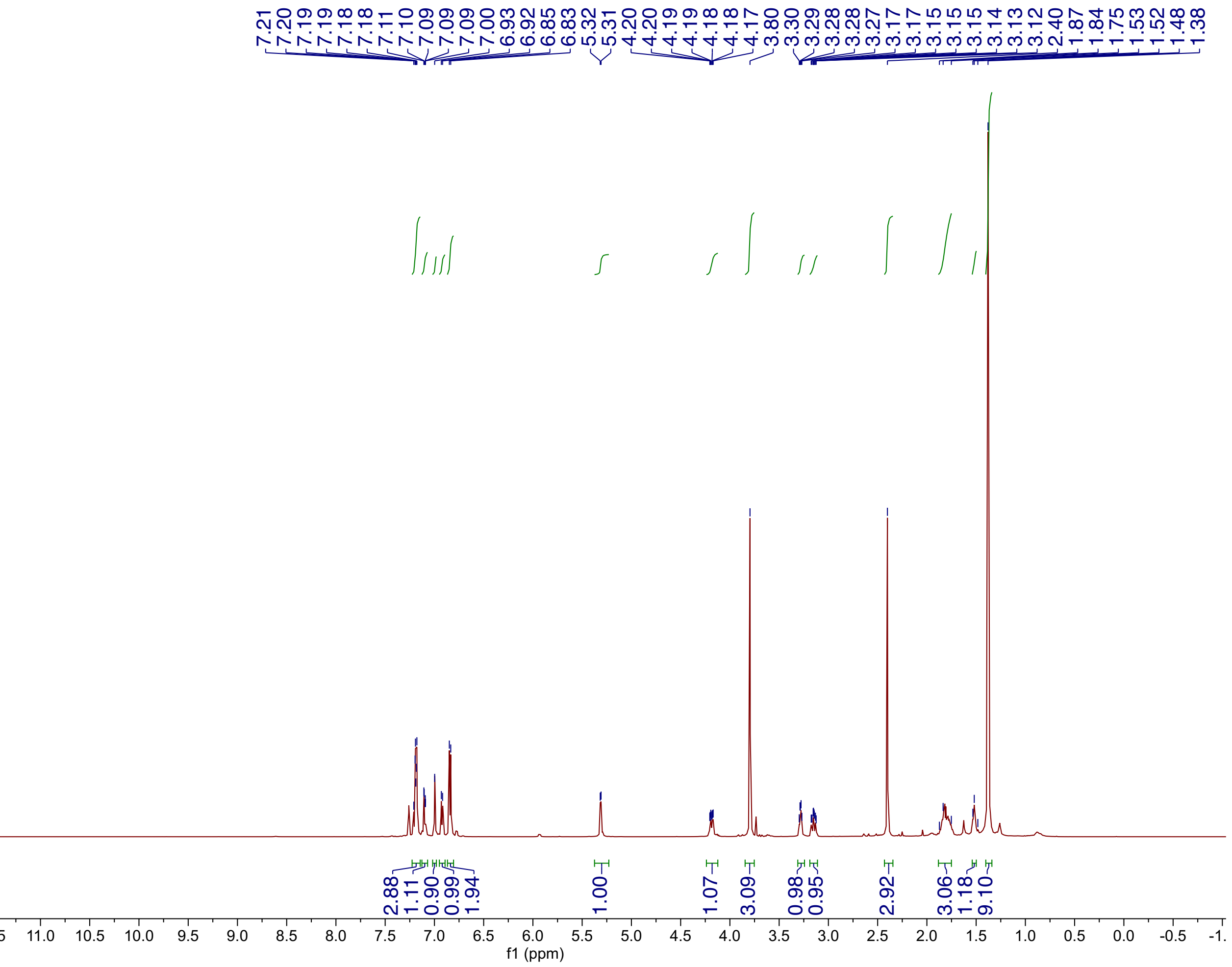

Compound 56 ^{13}C NMR

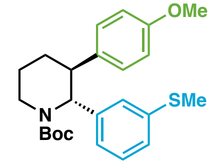

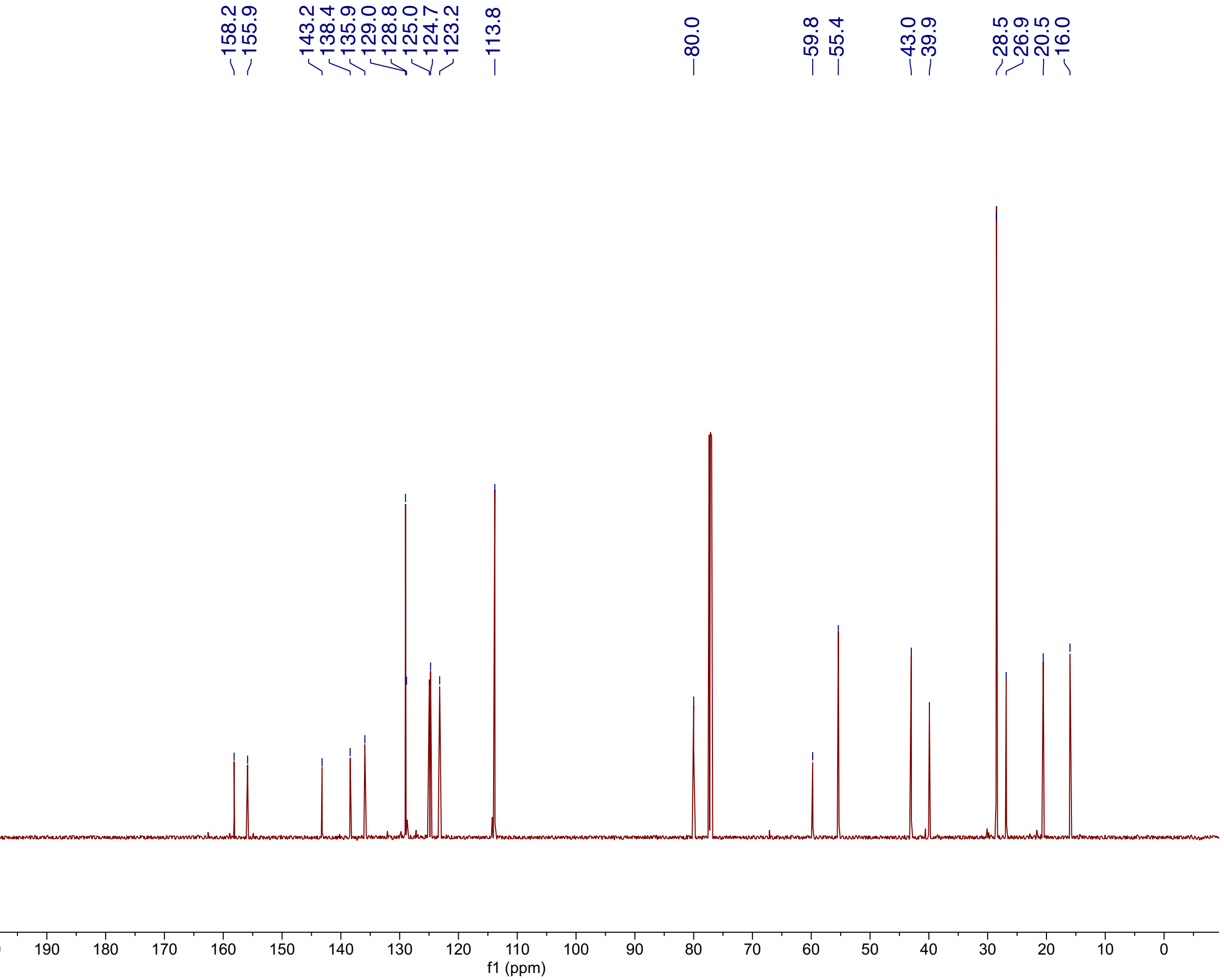

Compound 57 ^{1}H NMR

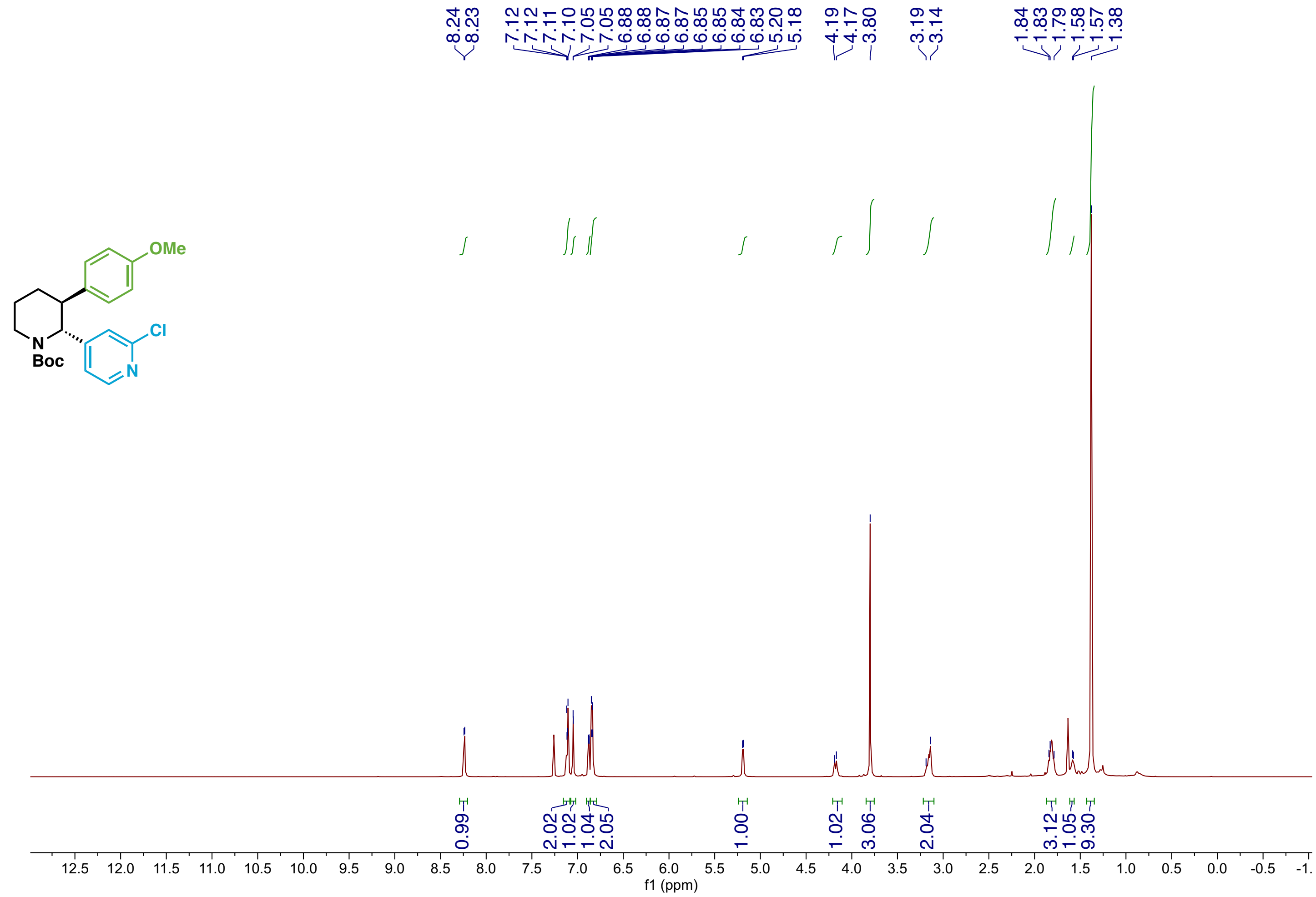

Compound 57 ^{13}C NMR

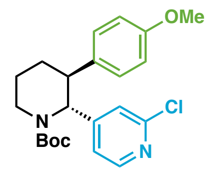

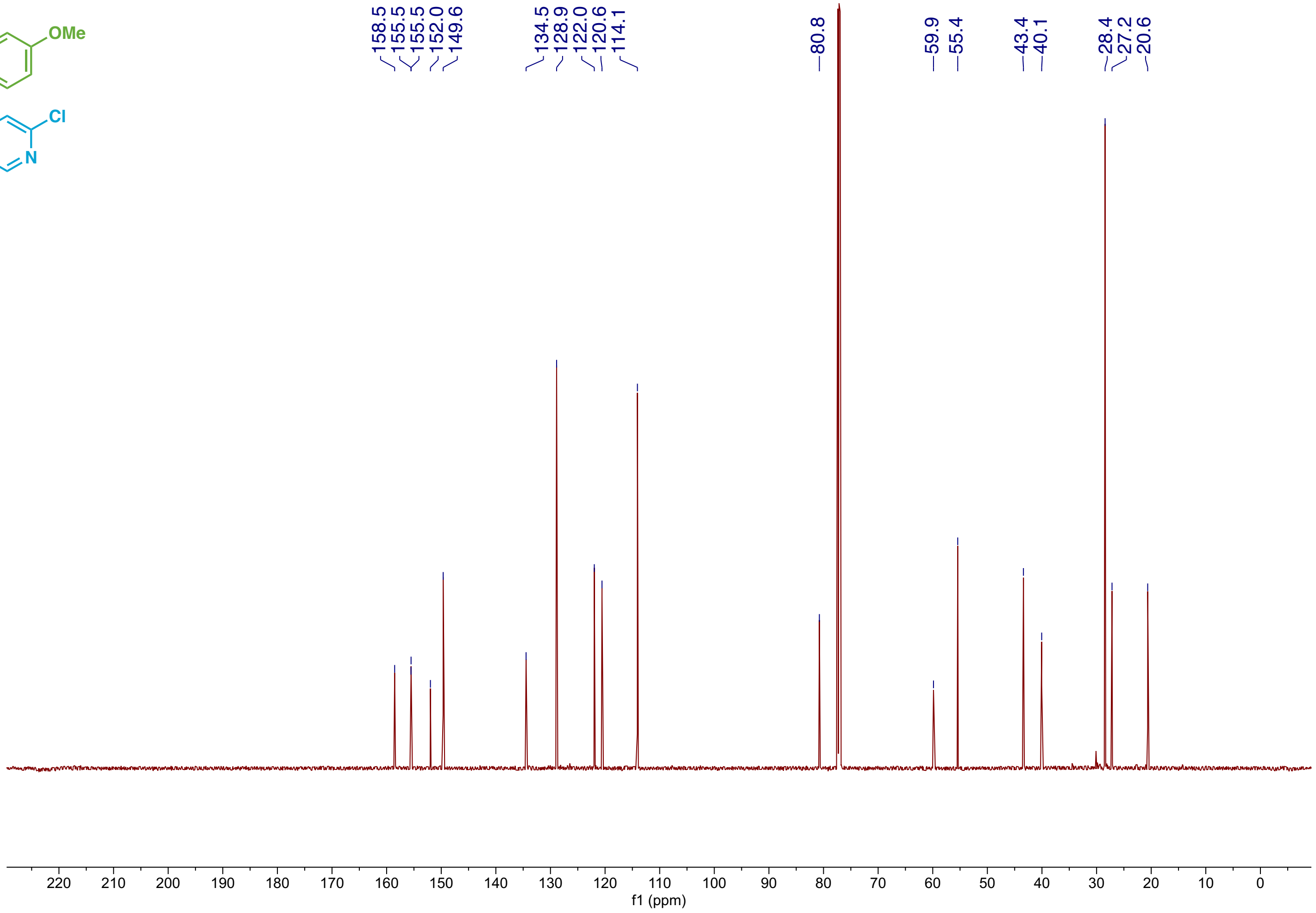

Compound 58 ^{1}H NMR

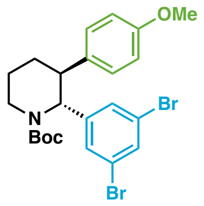

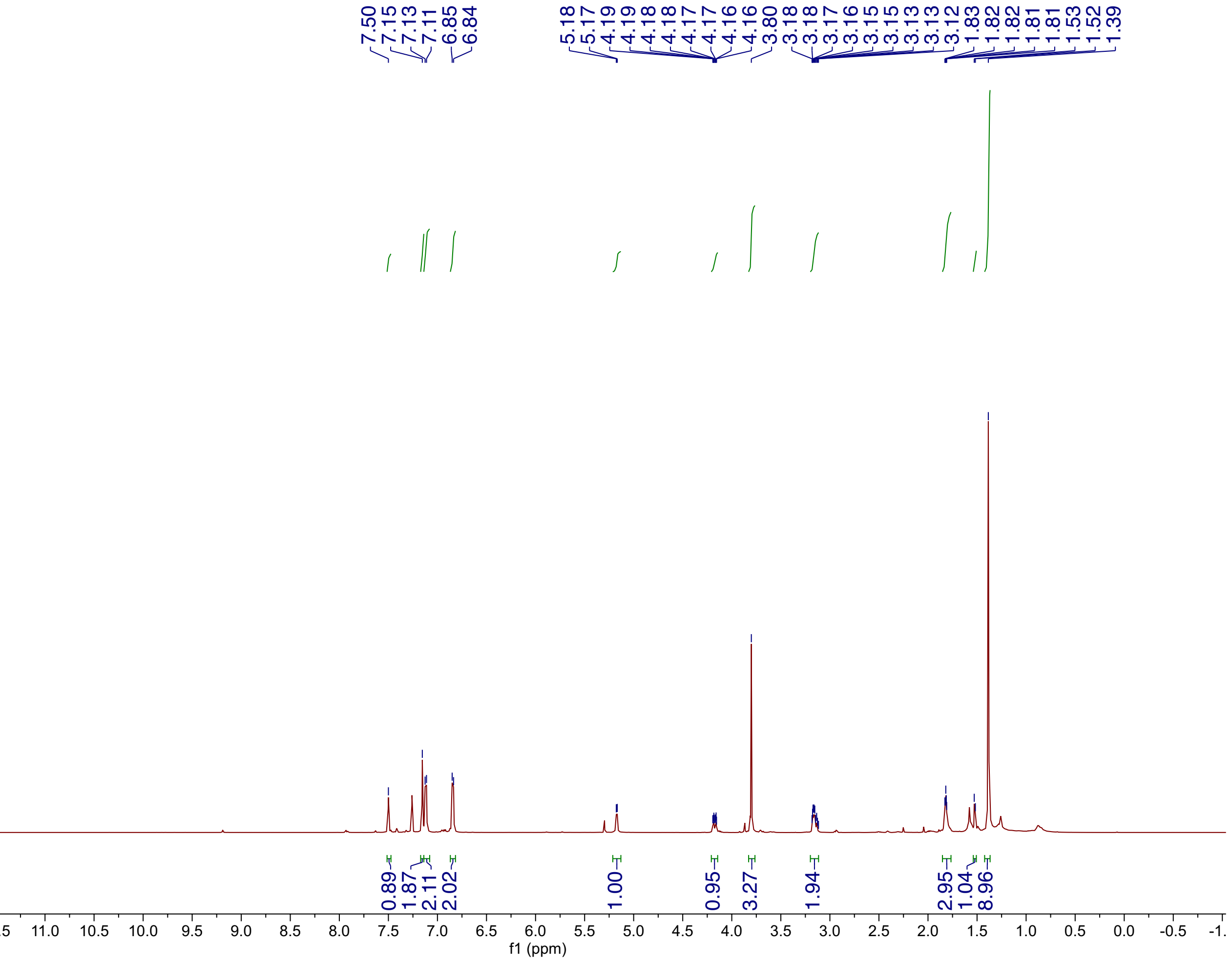

Compound 58 ^{13}C NMR

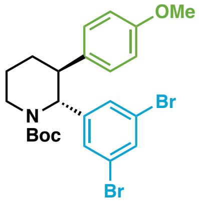

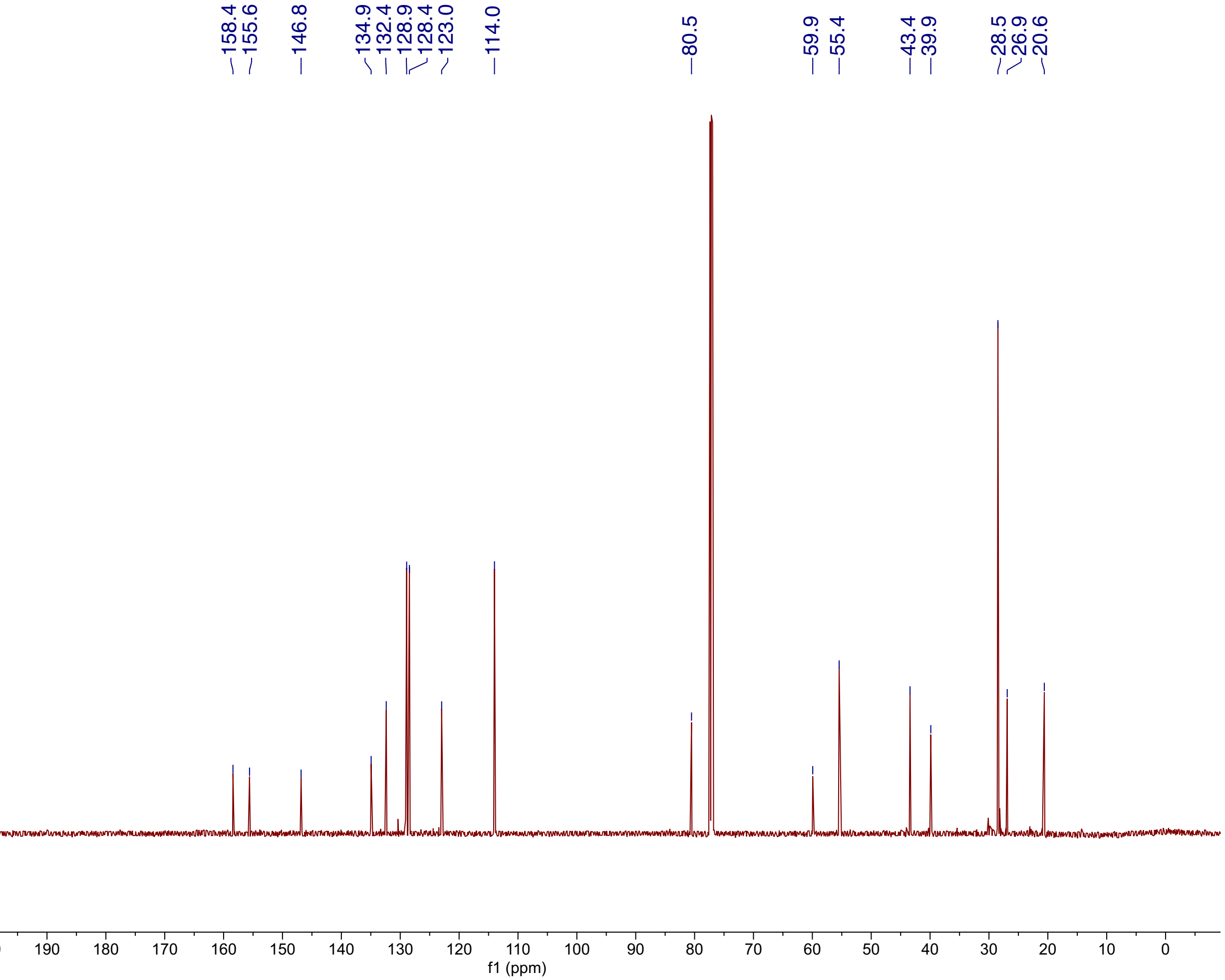

Compound 59 ^{1}H NMR

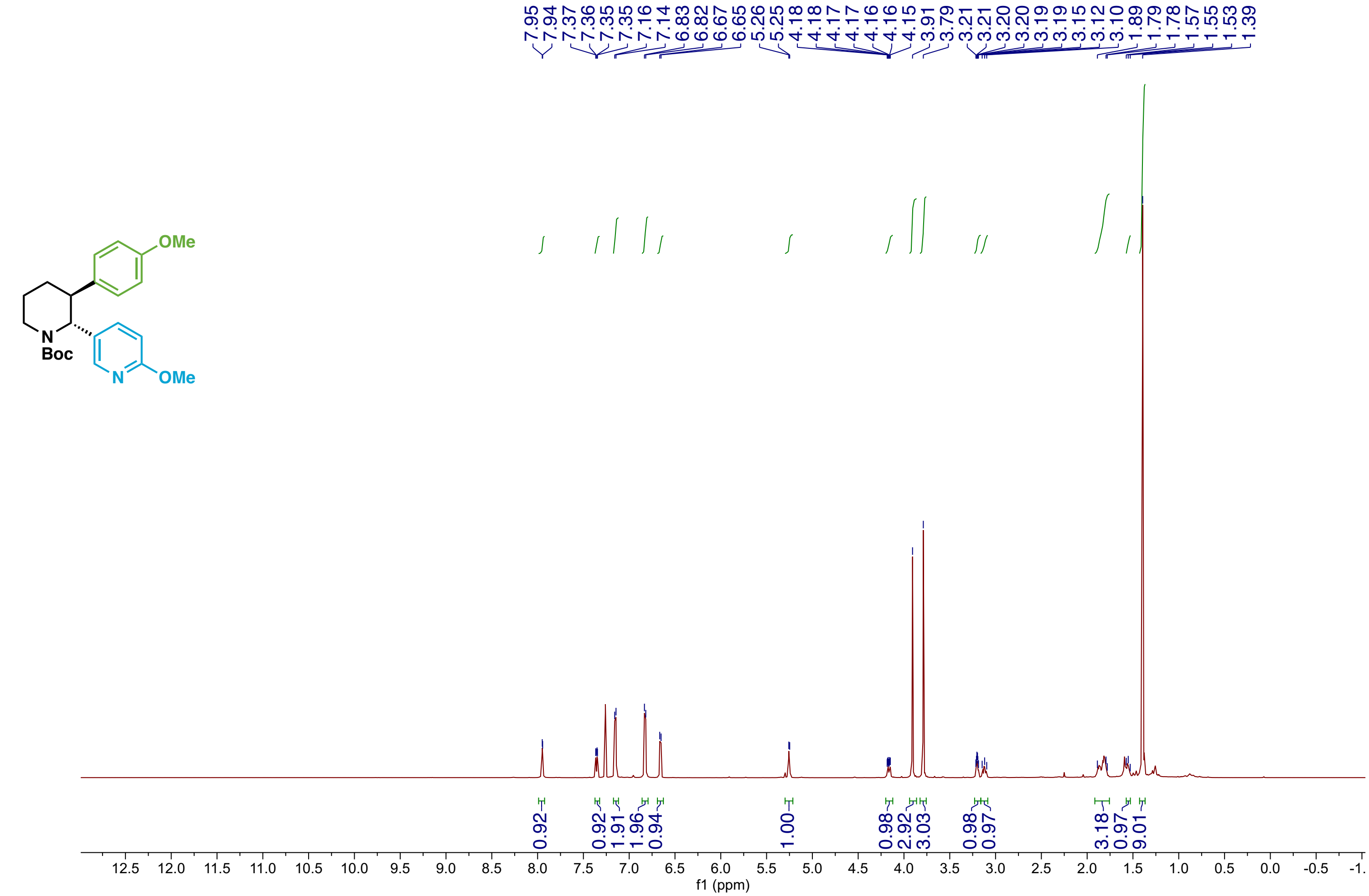

Compound 59 ^{13}C NMR

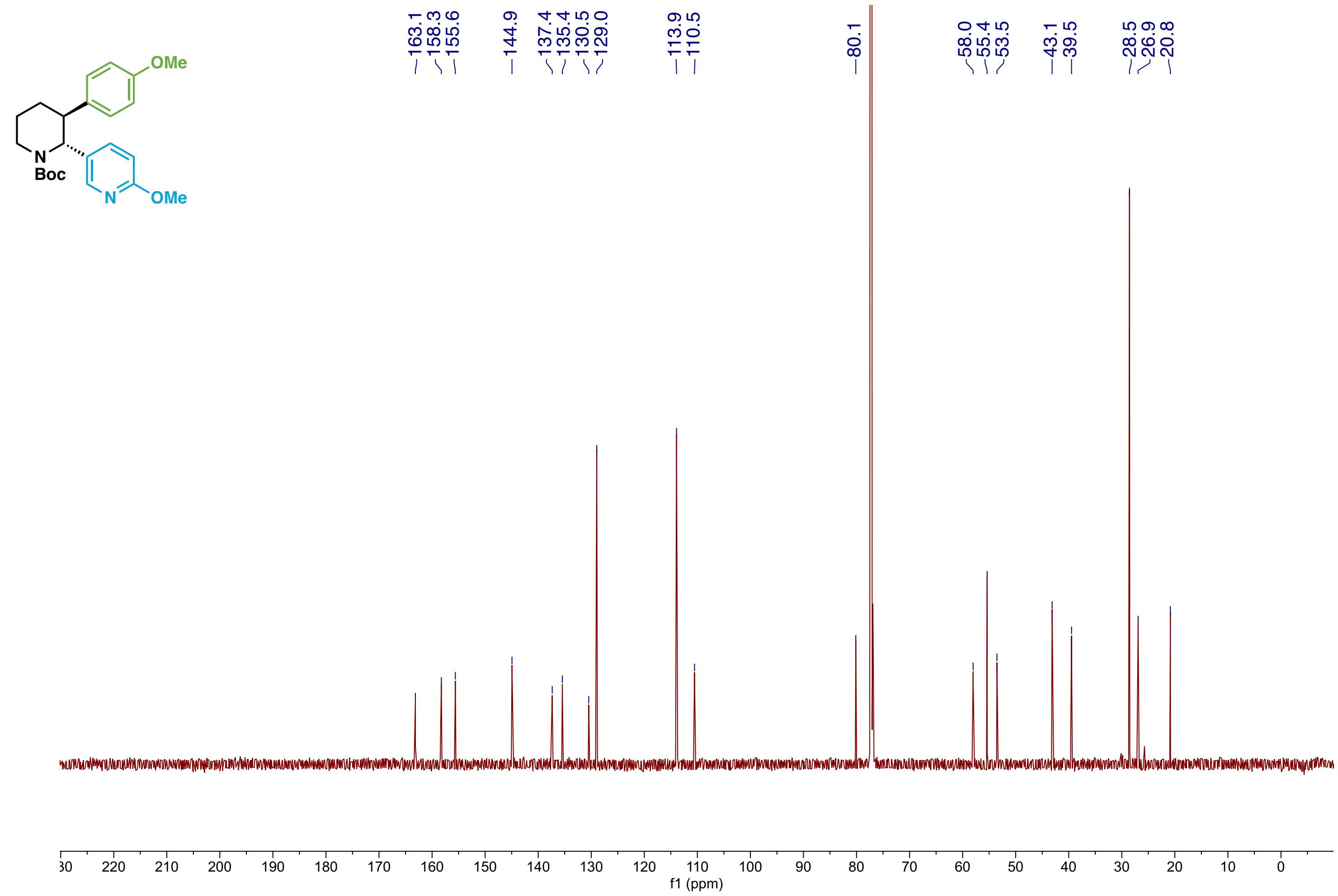

Compound B14 ^{1}H NMR

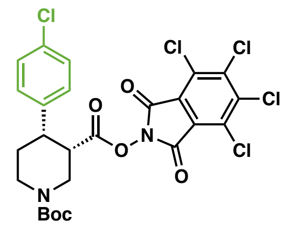

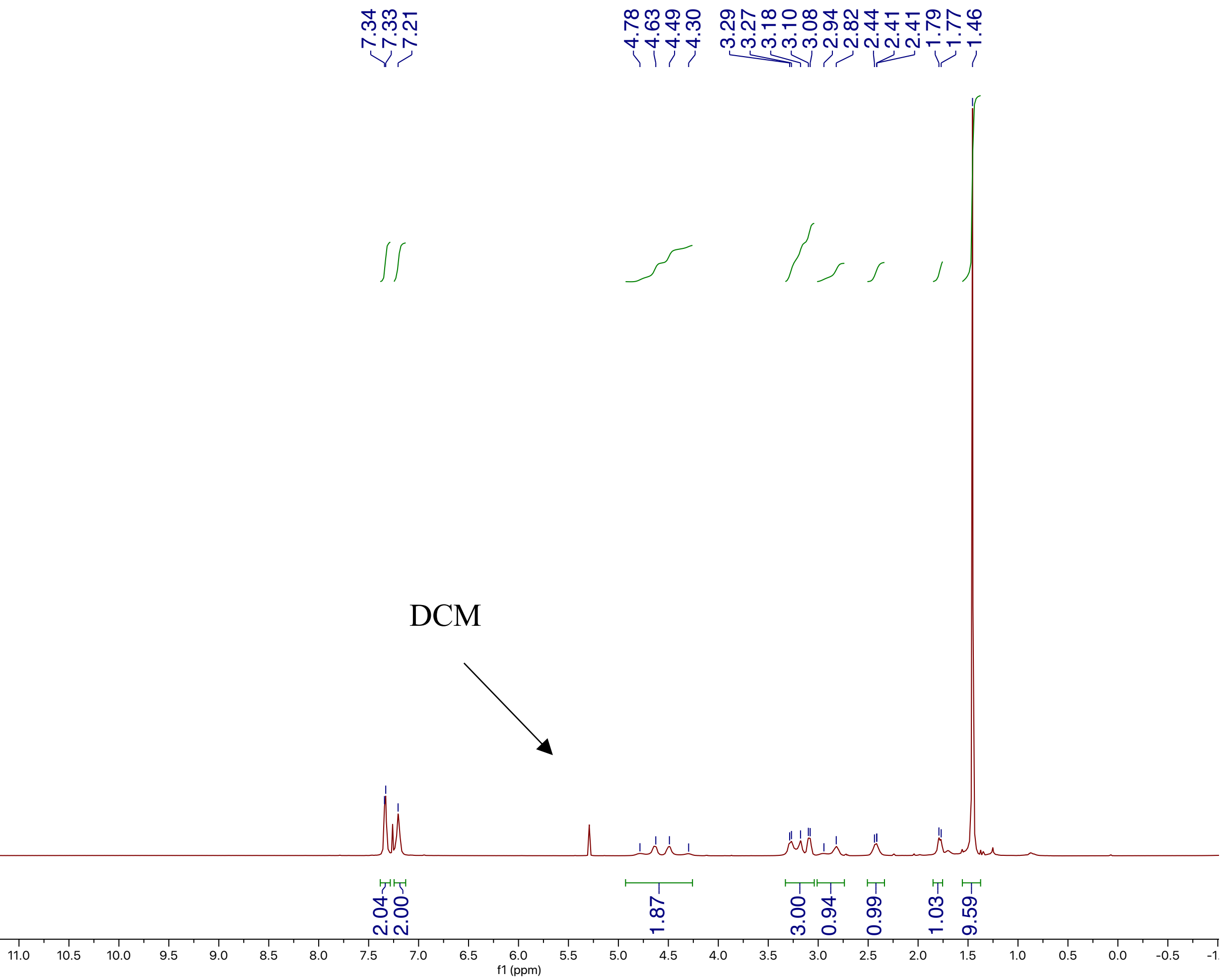

Compound B14 ^{13}C NMR

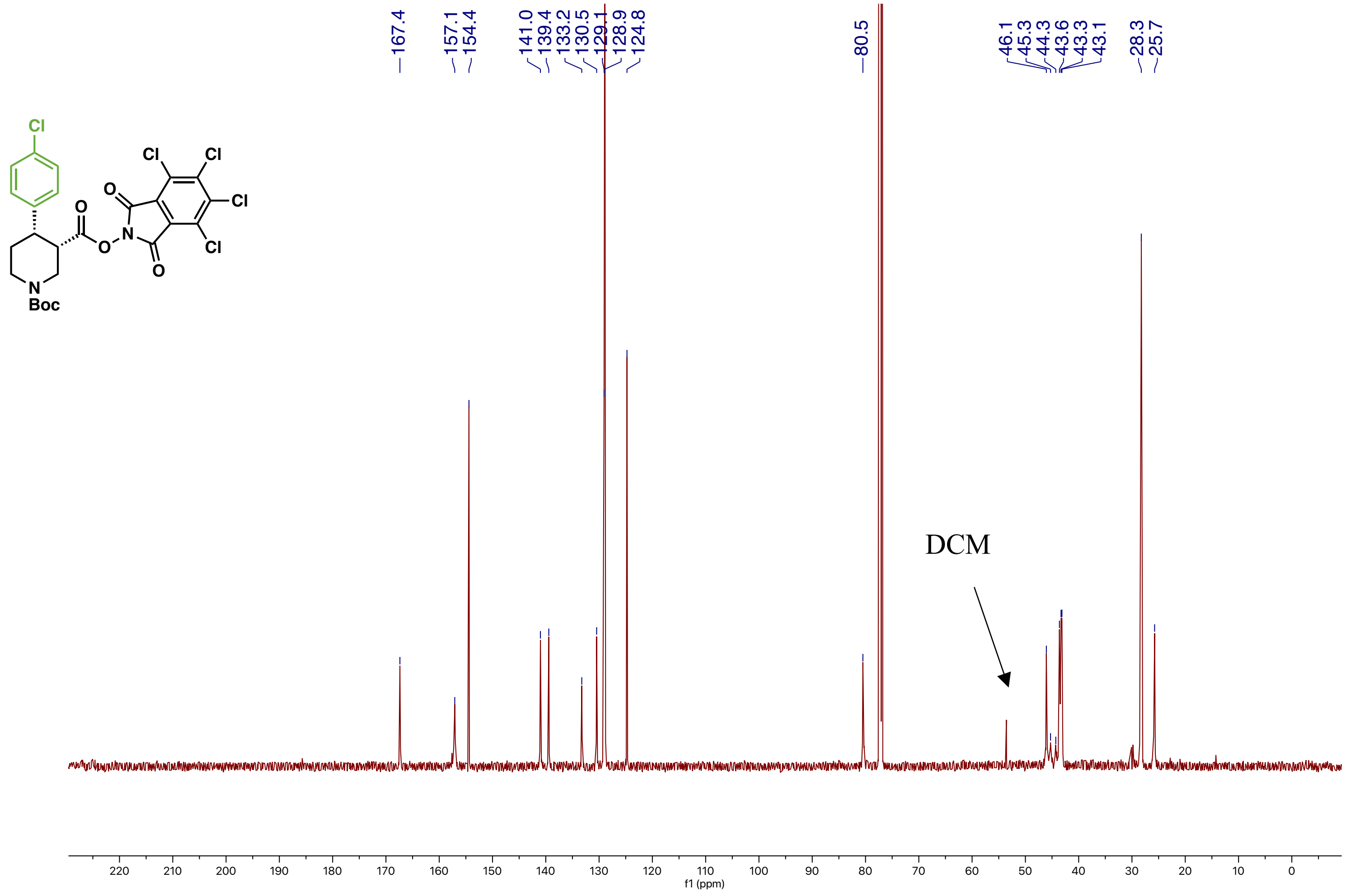

Compound 60 ^{1}H NMR

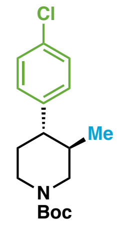

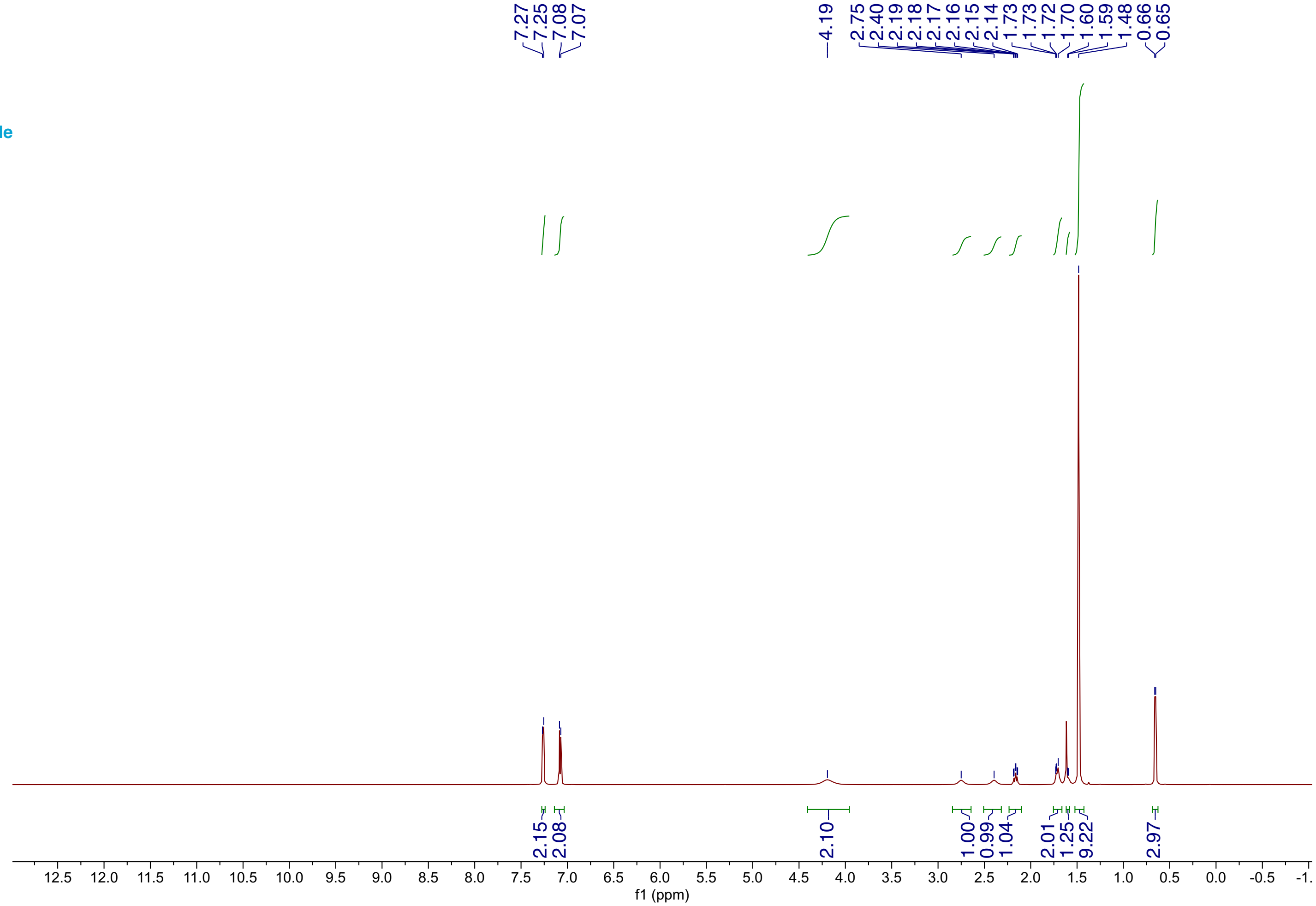

Compound 60 ^{13}C NMR

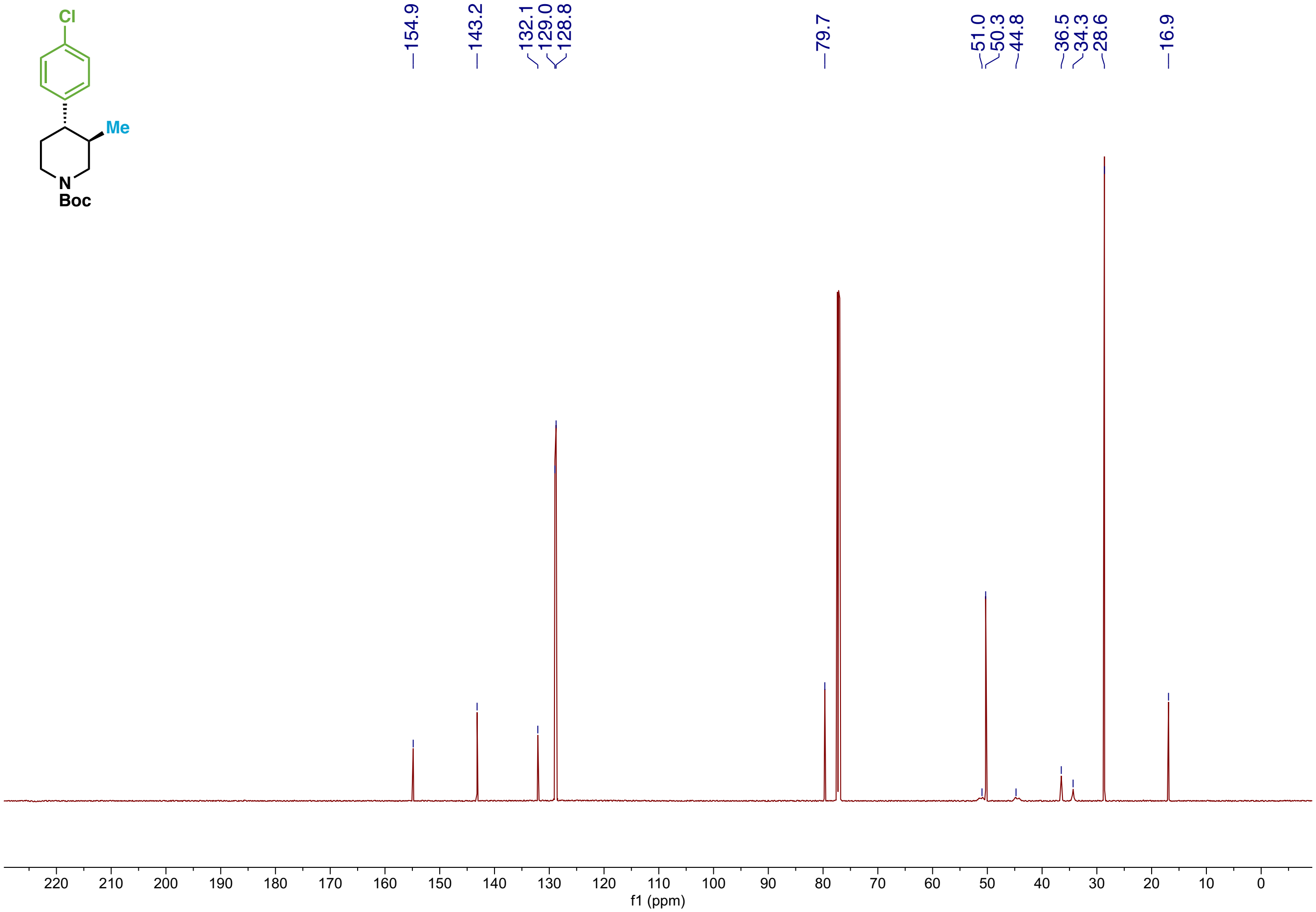

Compound 61 ^{1}H NMR

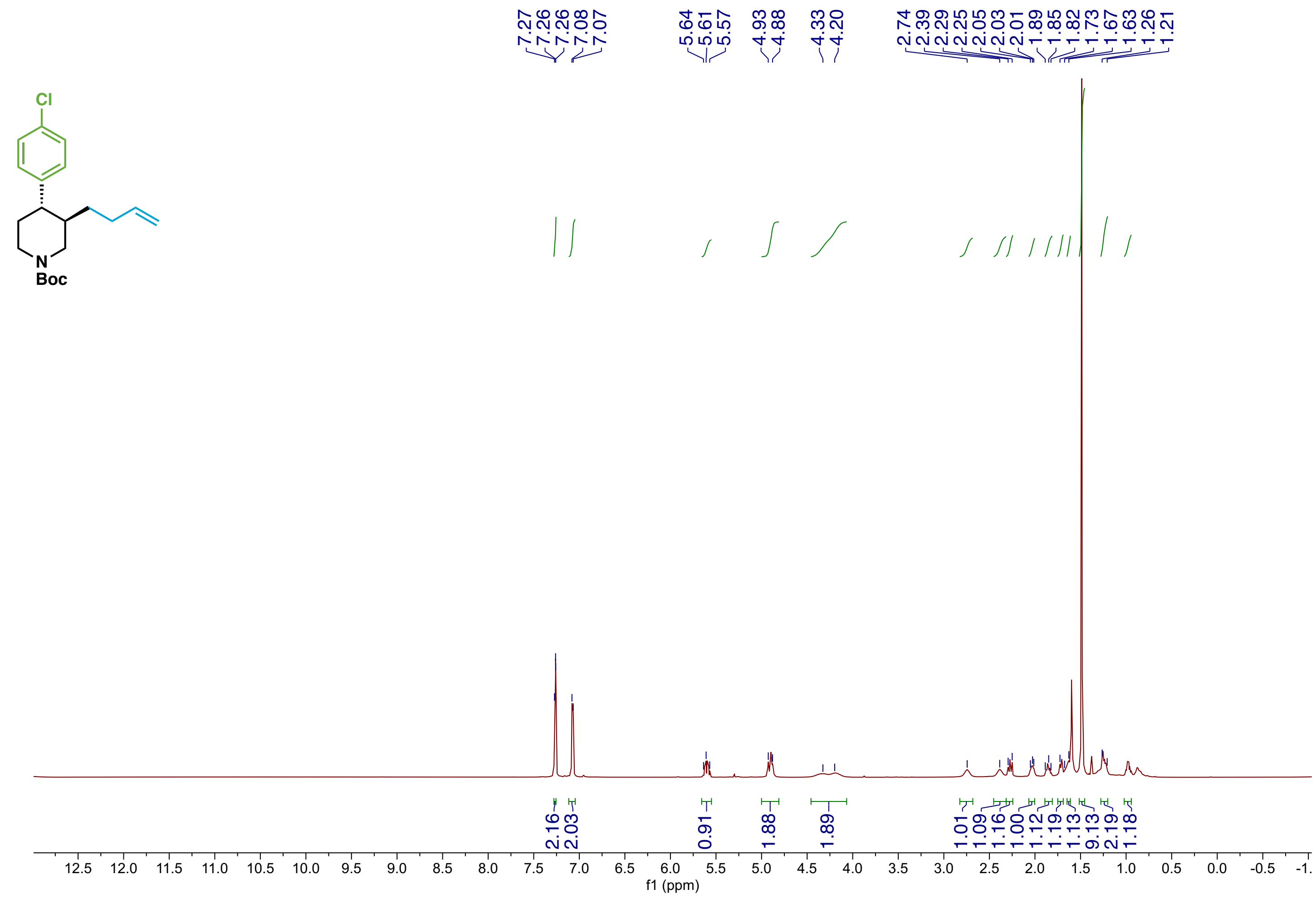

Compound 61 ^{13}C NMR

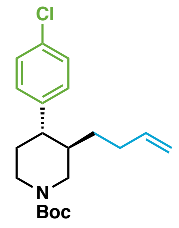

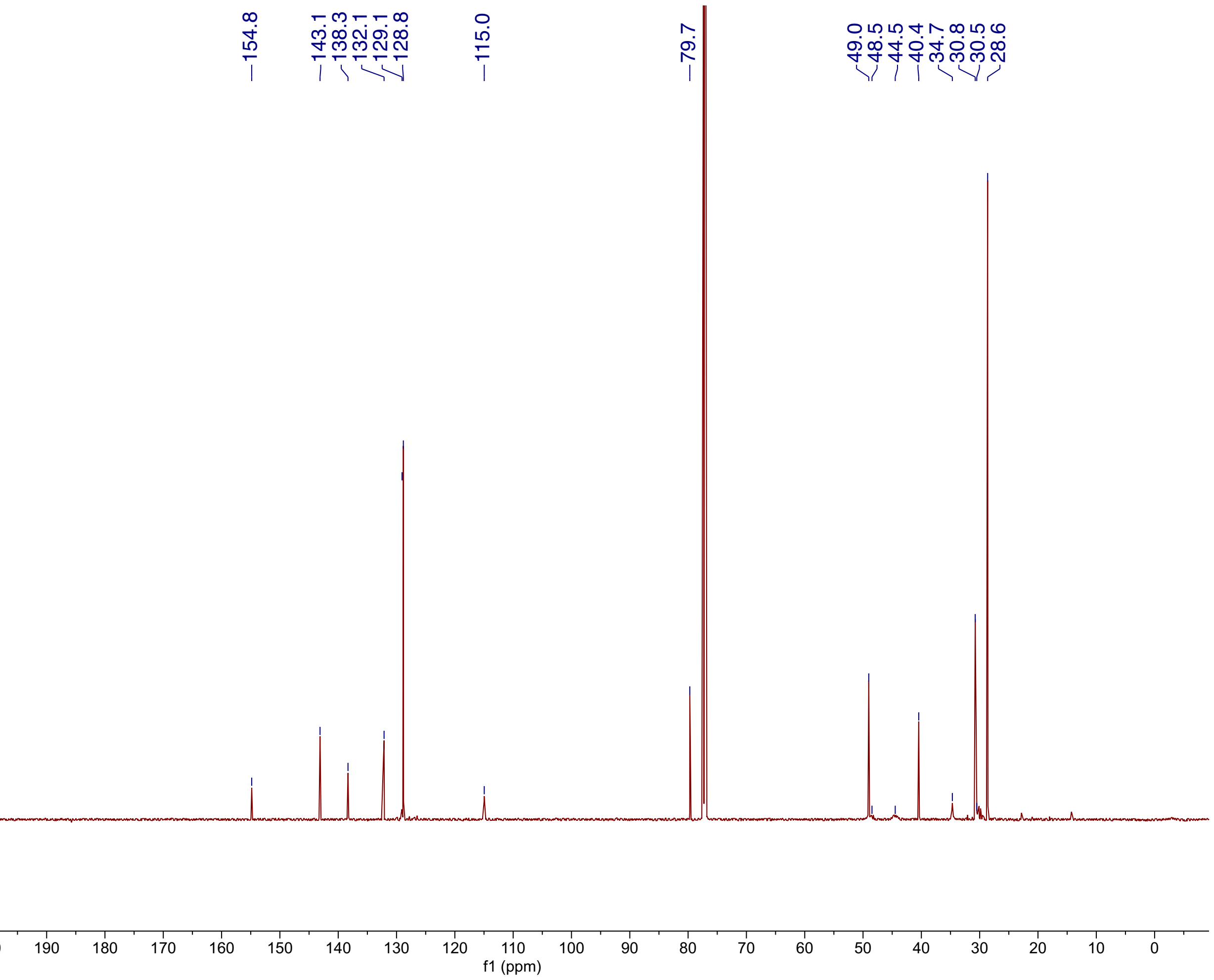

Compound 62 ^{1}H NMR

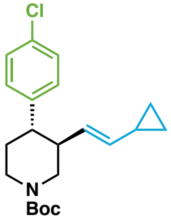

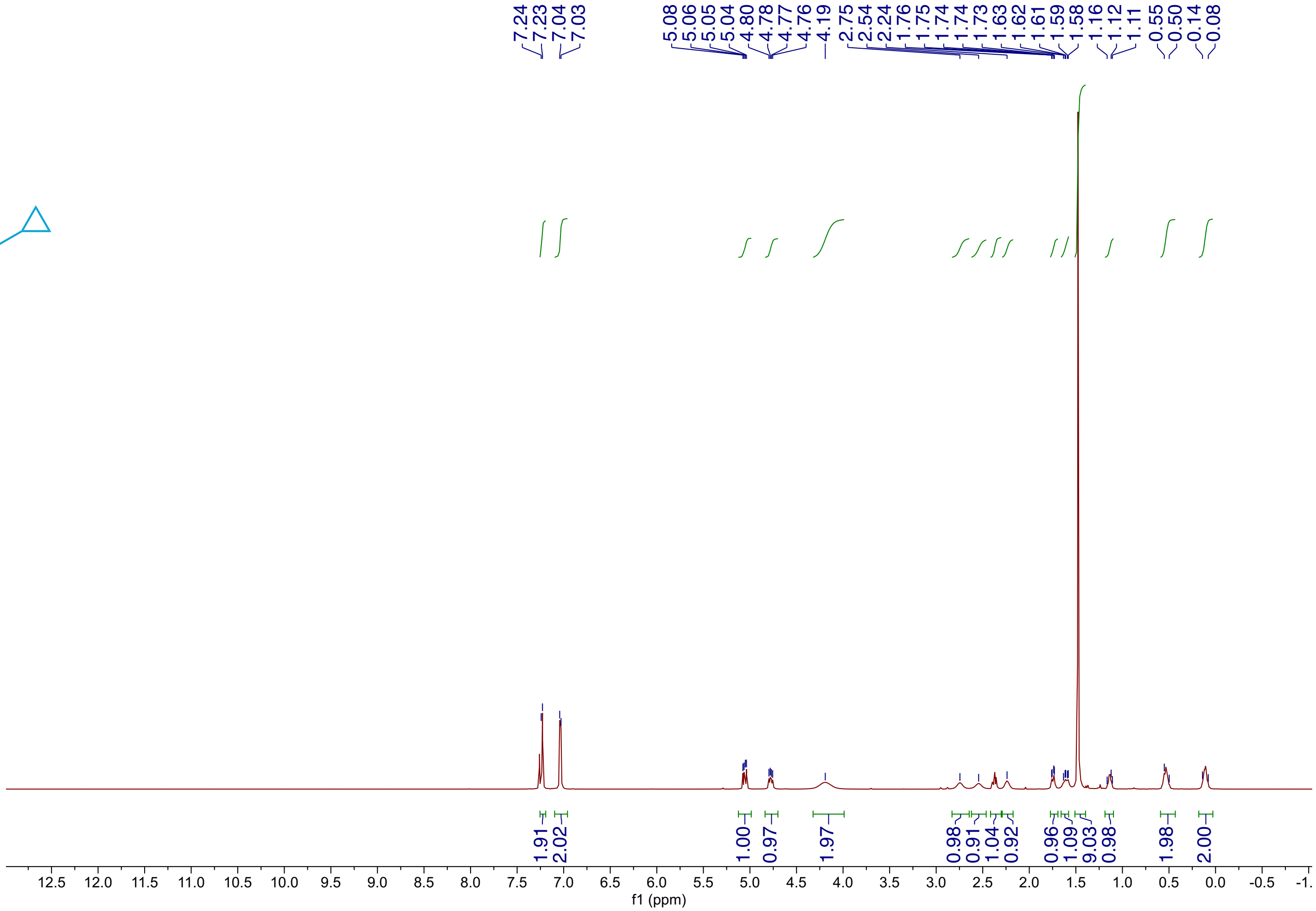

Compound 62 ^{13}C NMR

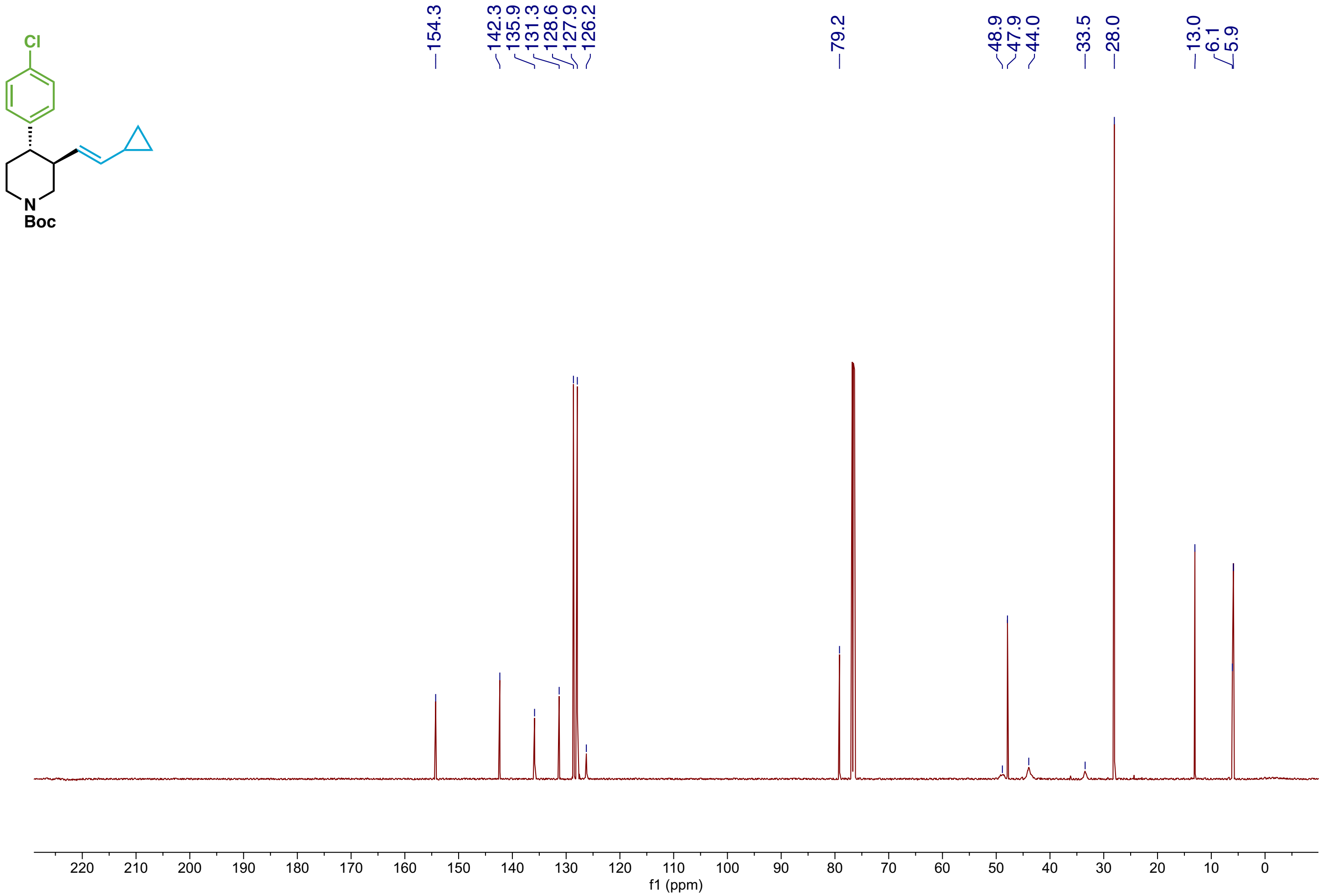

Compound 63 ^{1}H NMR

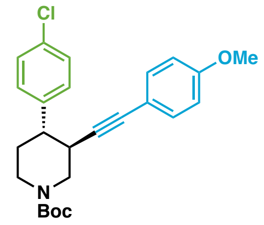

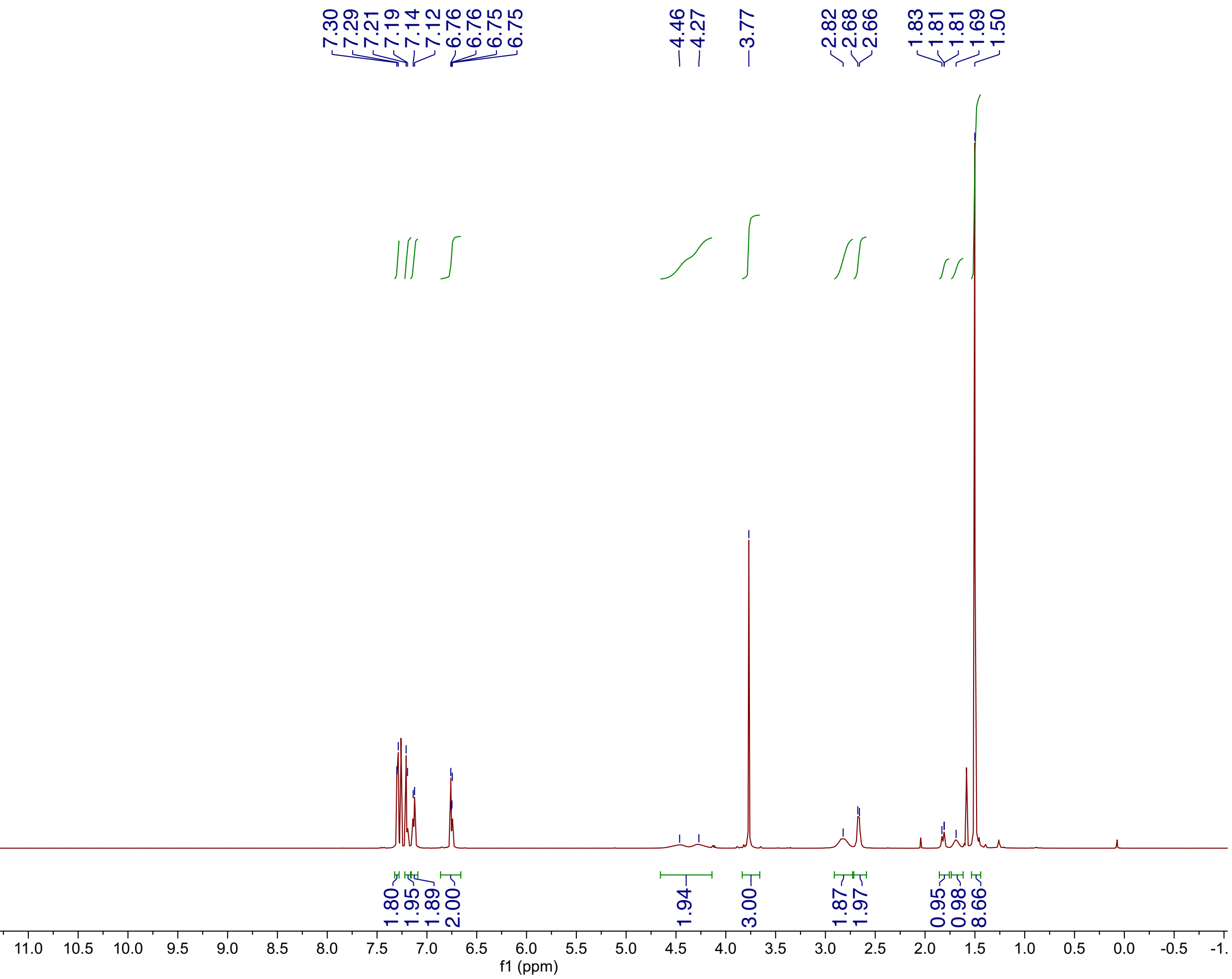

Compound 63 ^{13}C NMR

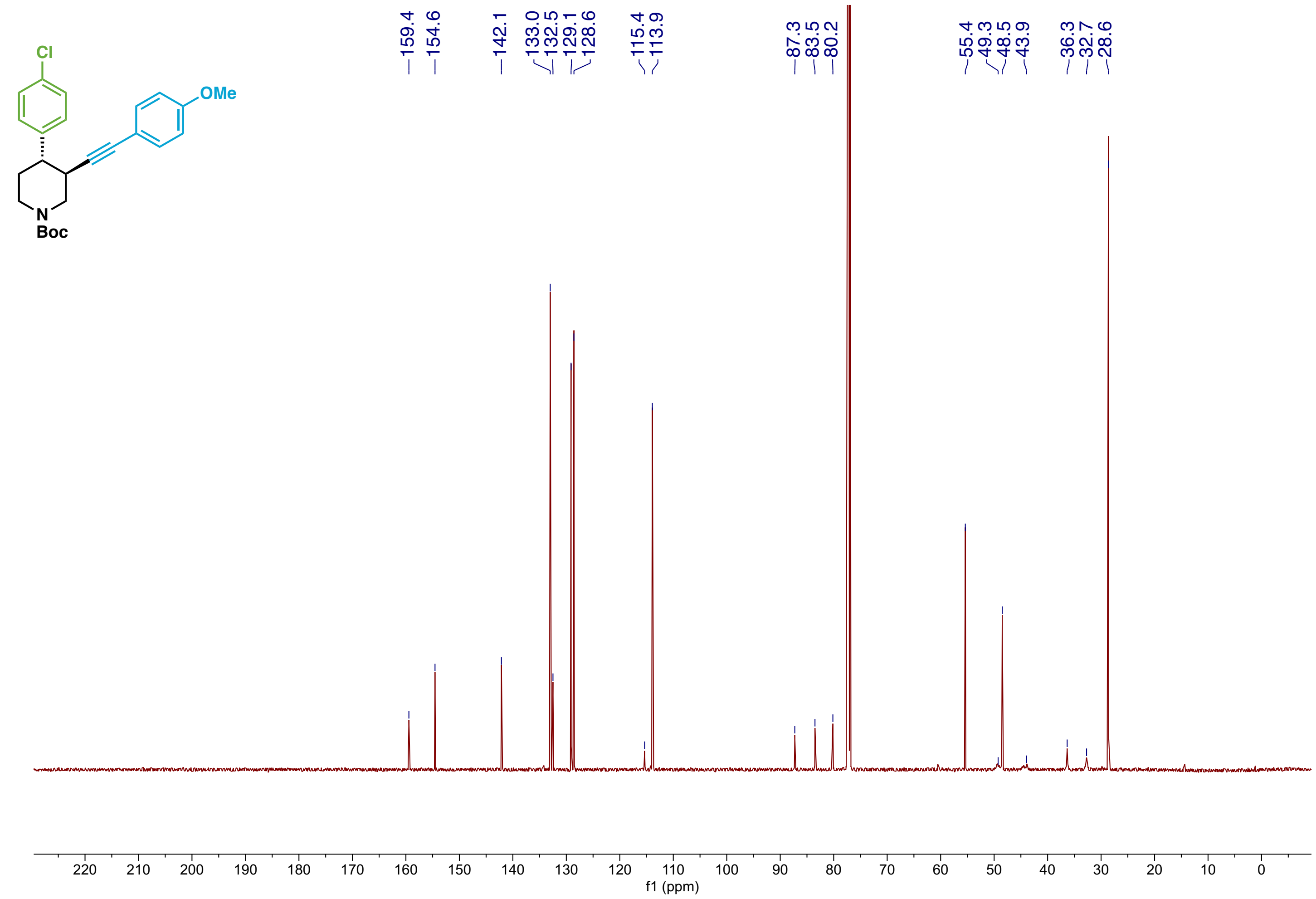

Compound 64 ^{1}H NMR

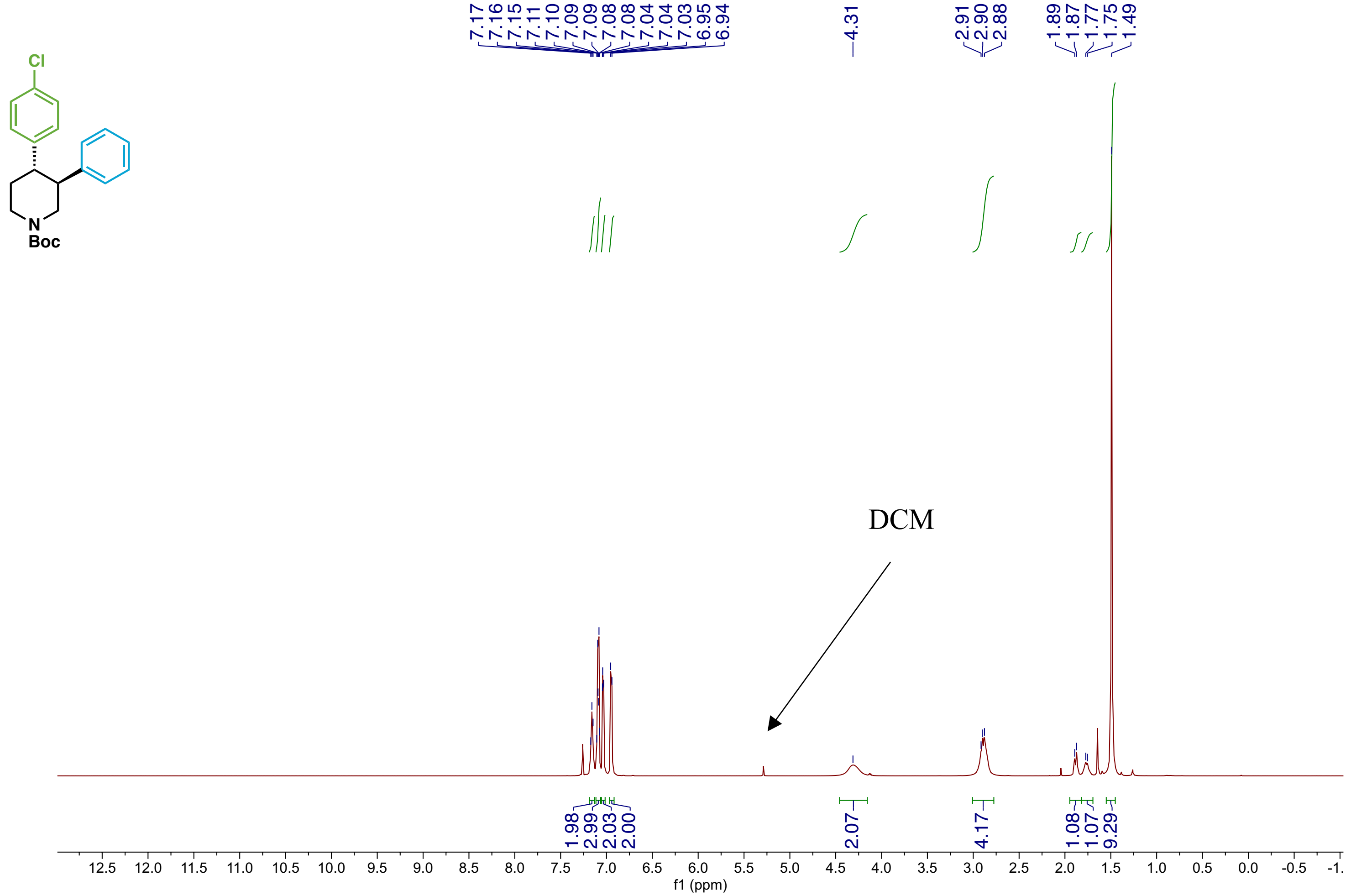

Compound 64 ^{13}C NMR

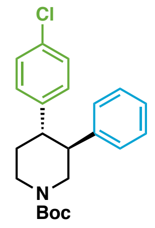

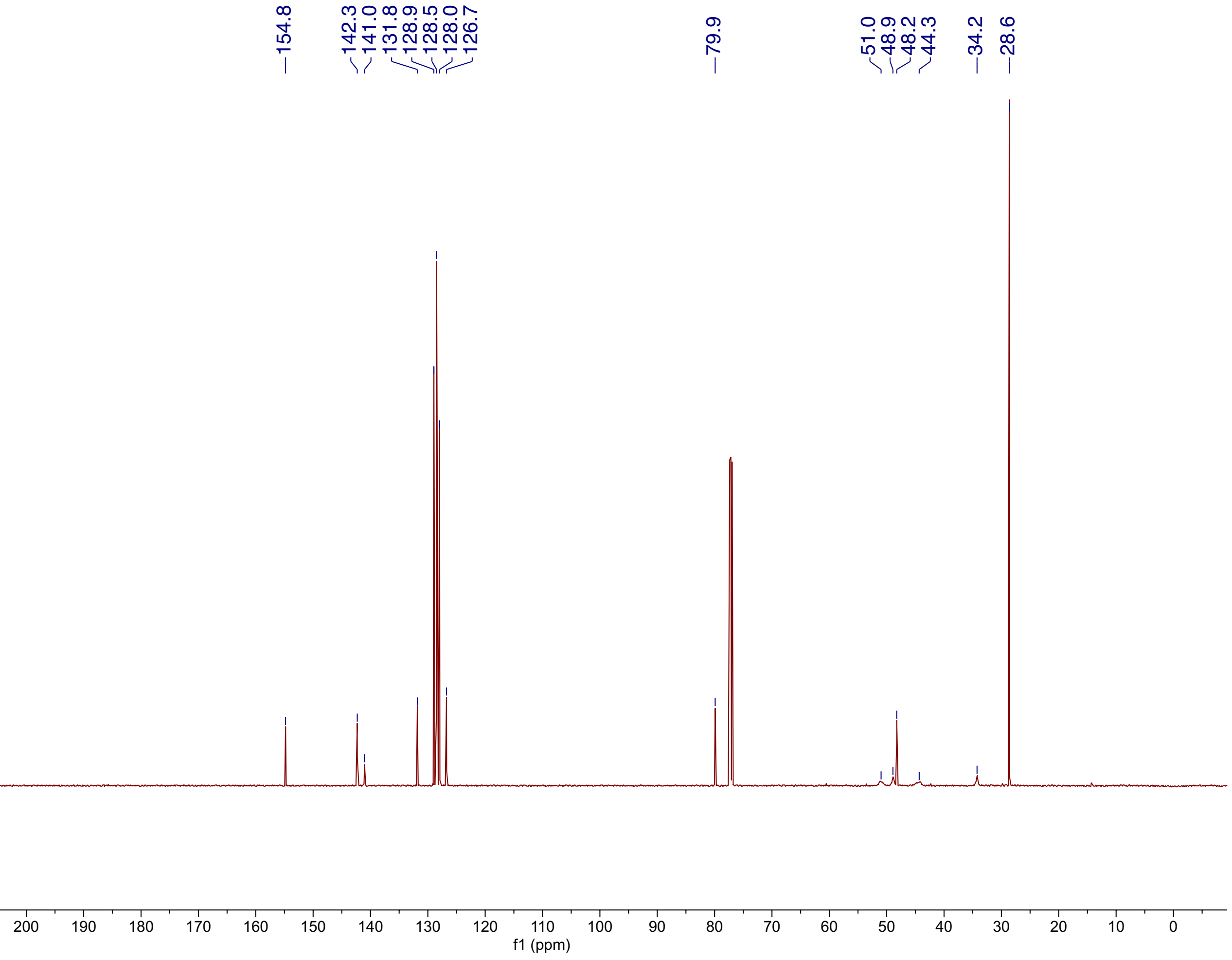

Compound 65 ^{1}H NMR

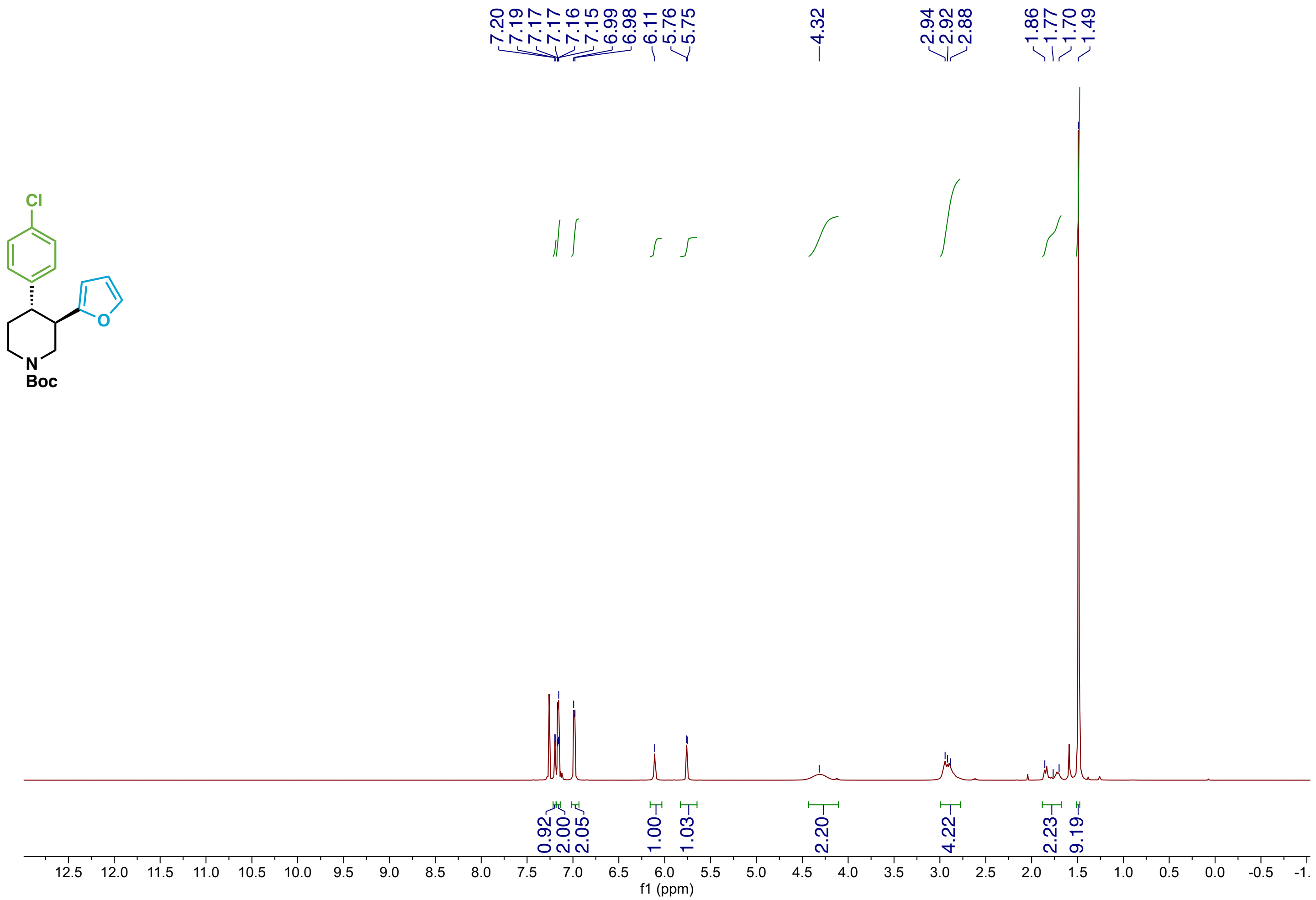

Compound 65 ^{13}C NMR

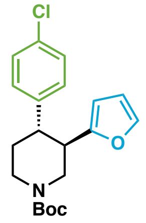

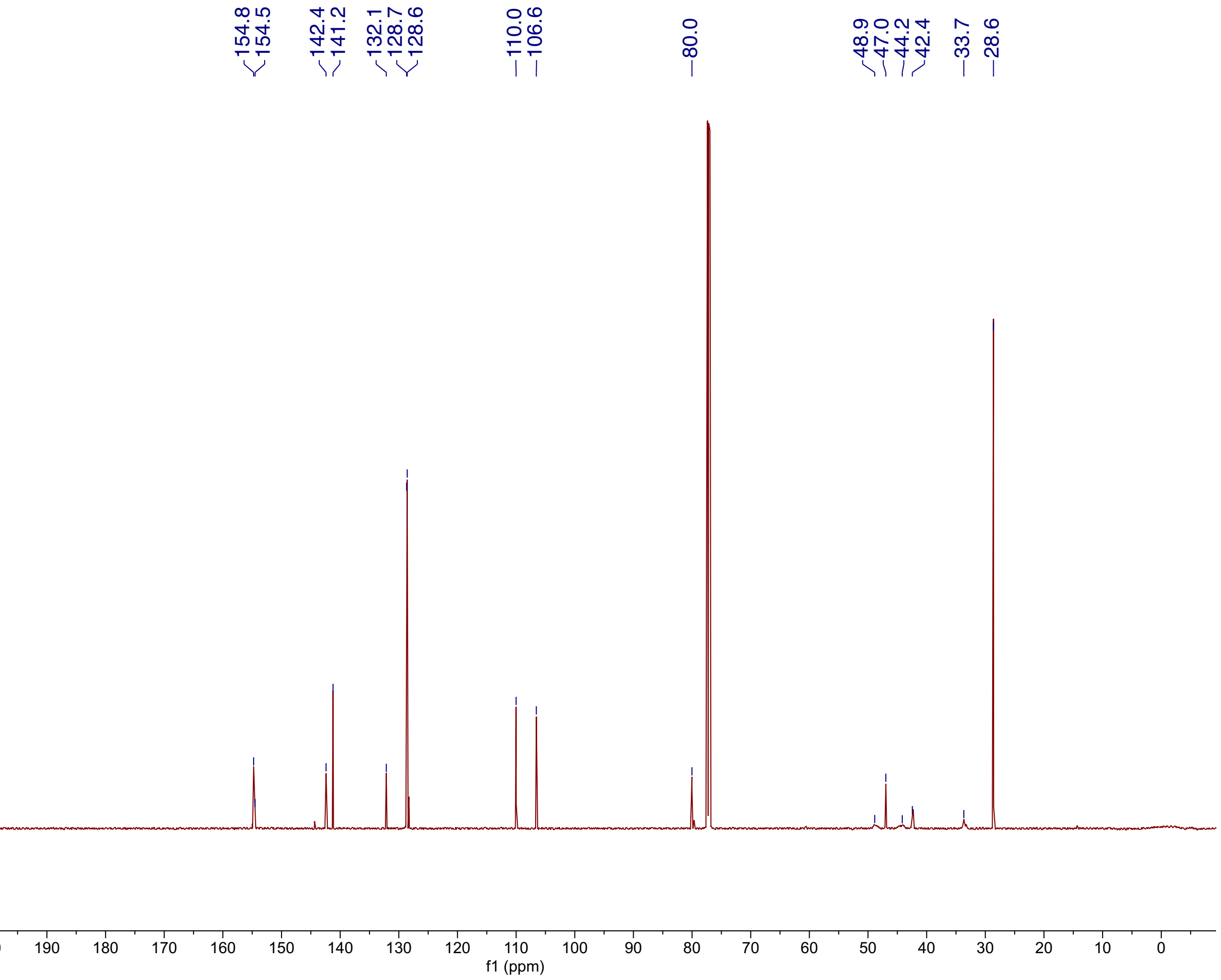

Compound SI-3 ^{1}H NMR

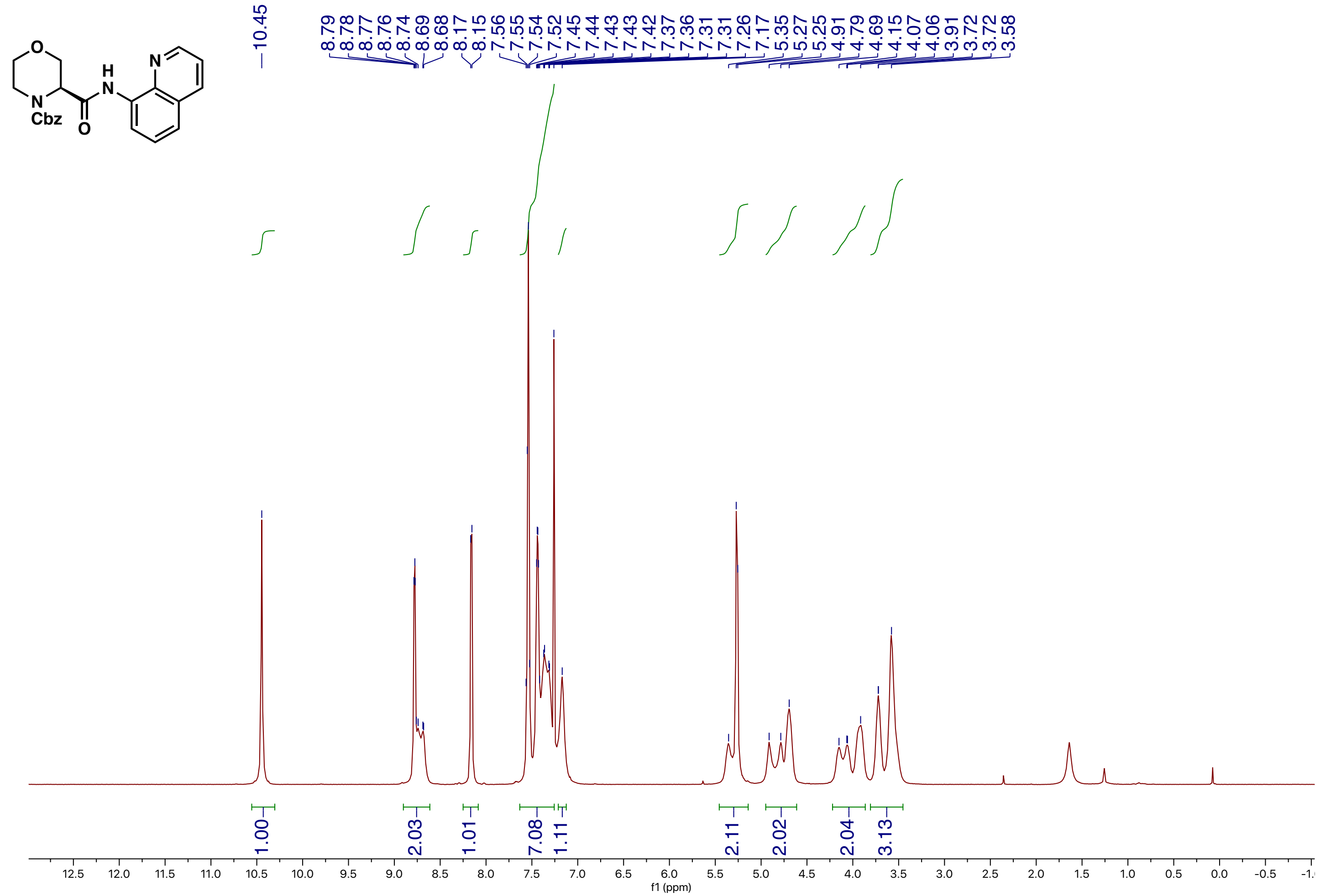

Compound SI-3 ^{13}C NMR

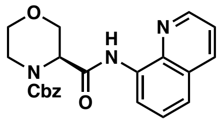

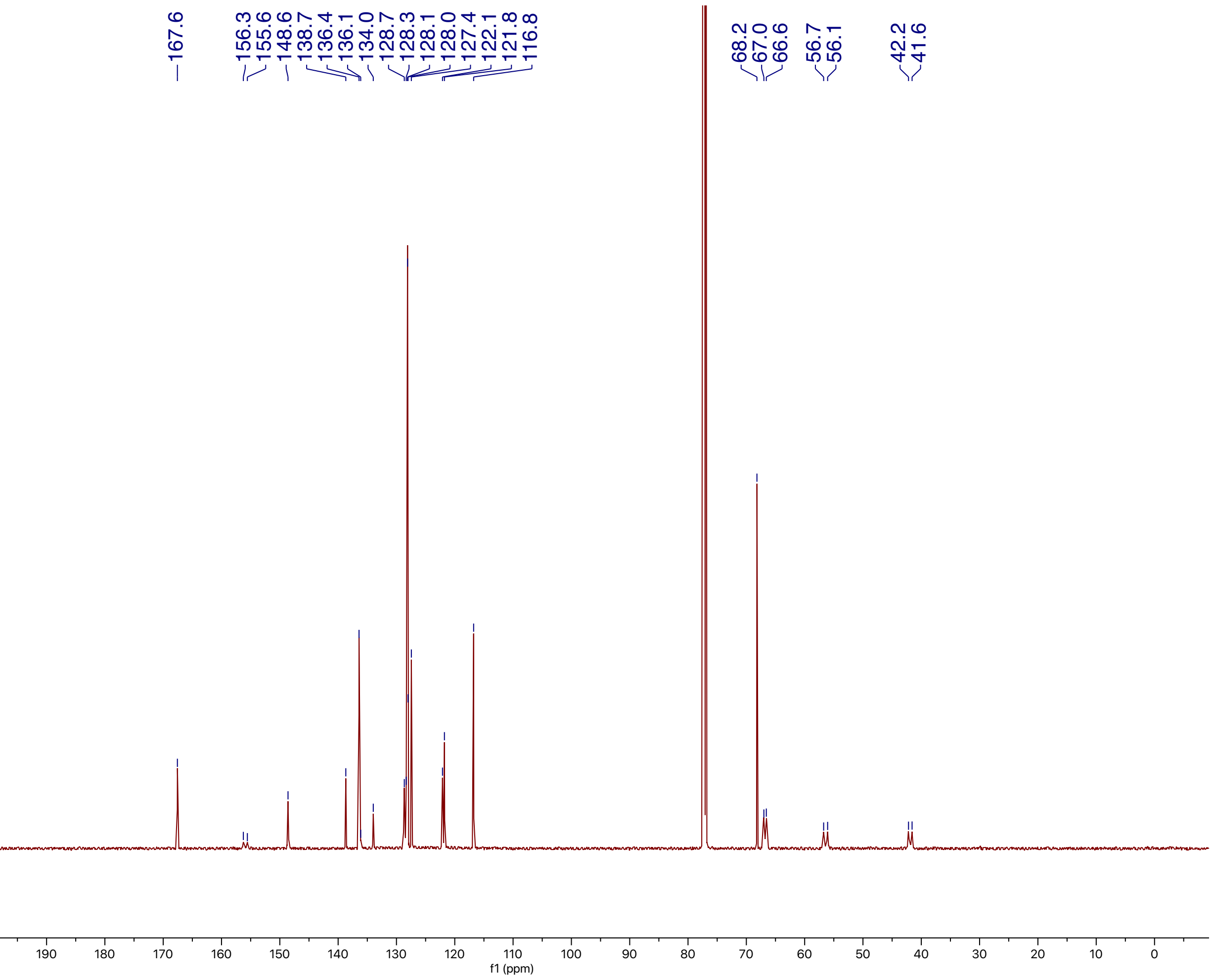

Compound SI-4 ^{1}H NMR

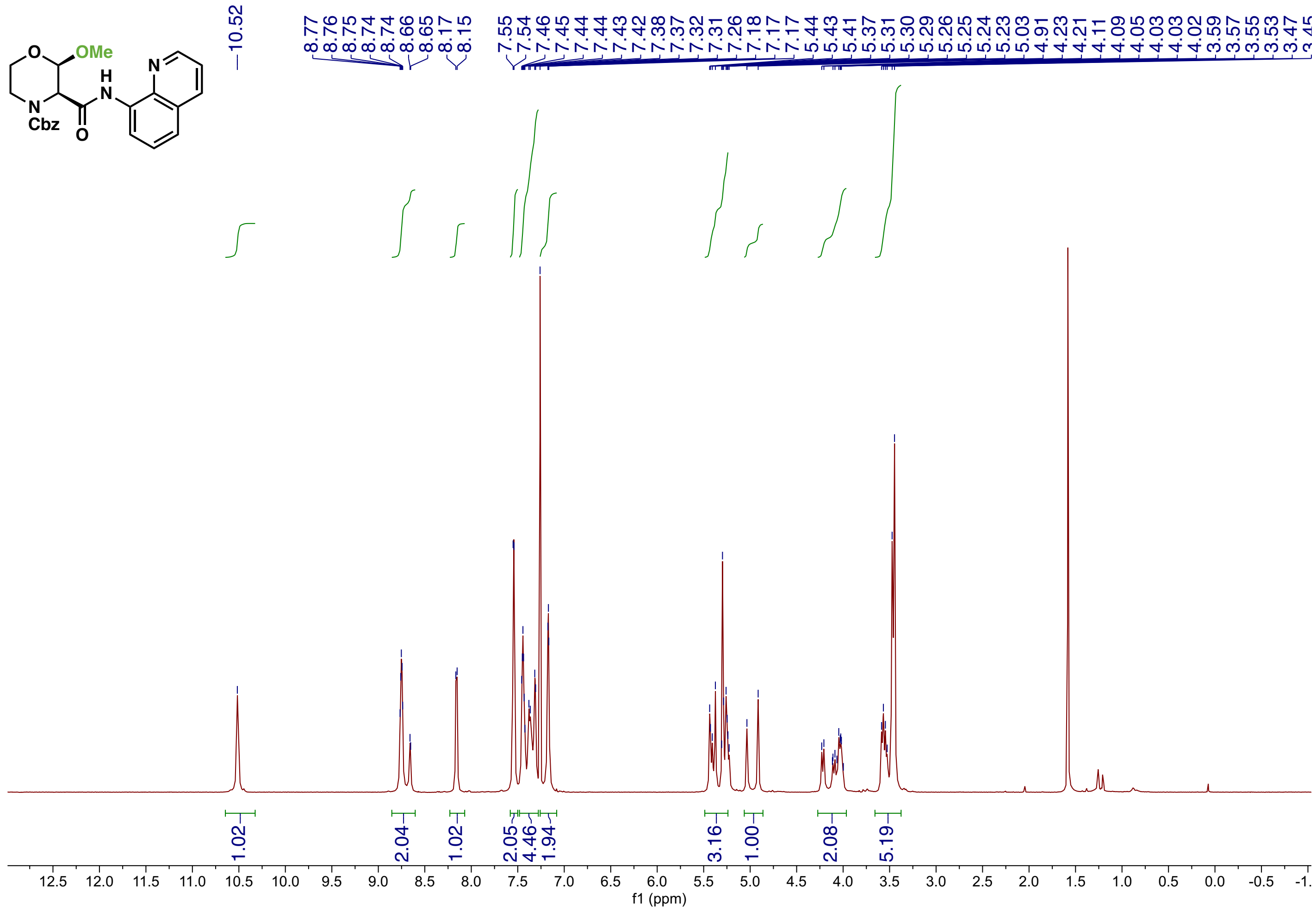

Compound SI-4 ^{13}C NMR

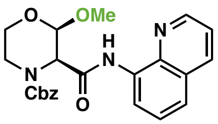

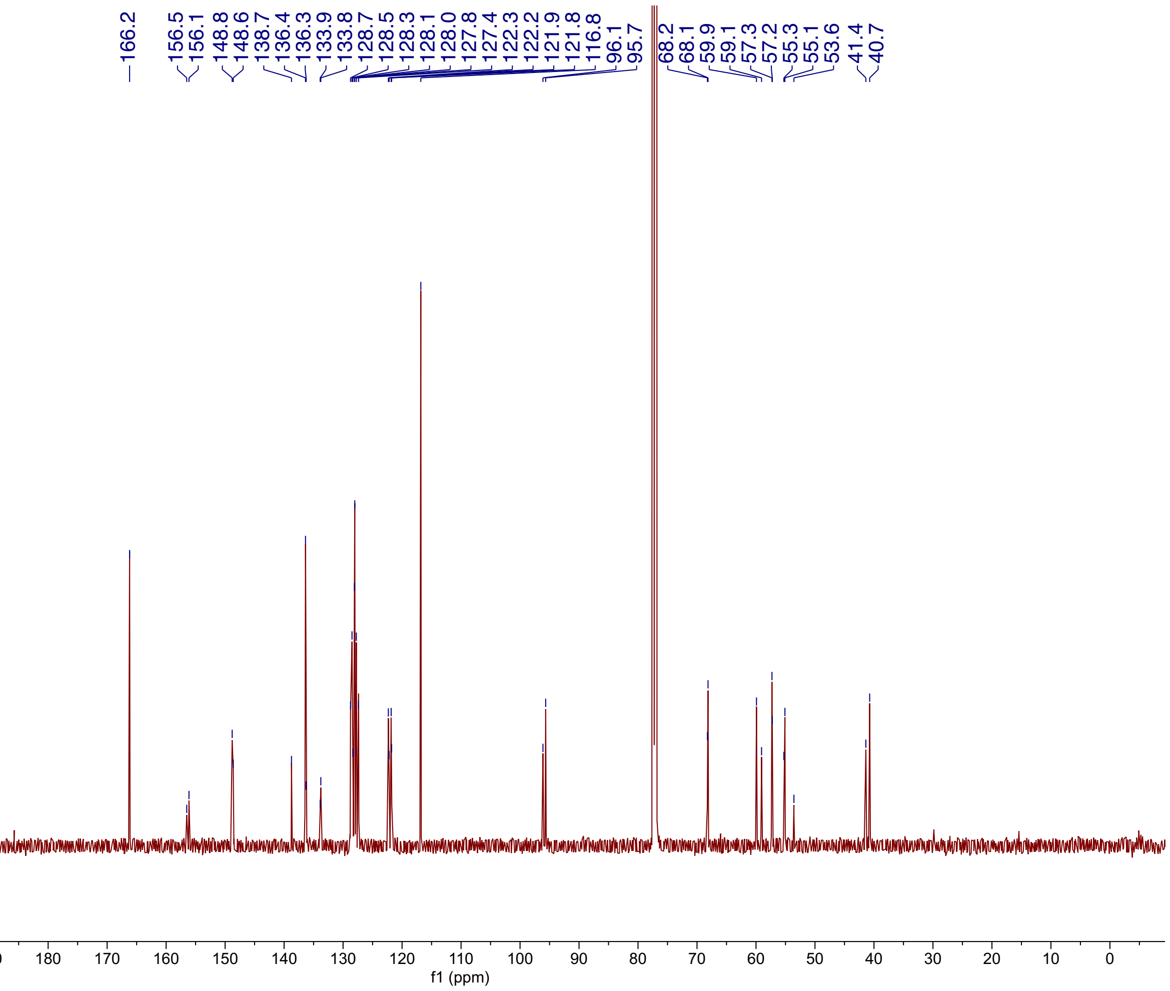

Compound B16 ^{1}H NMR

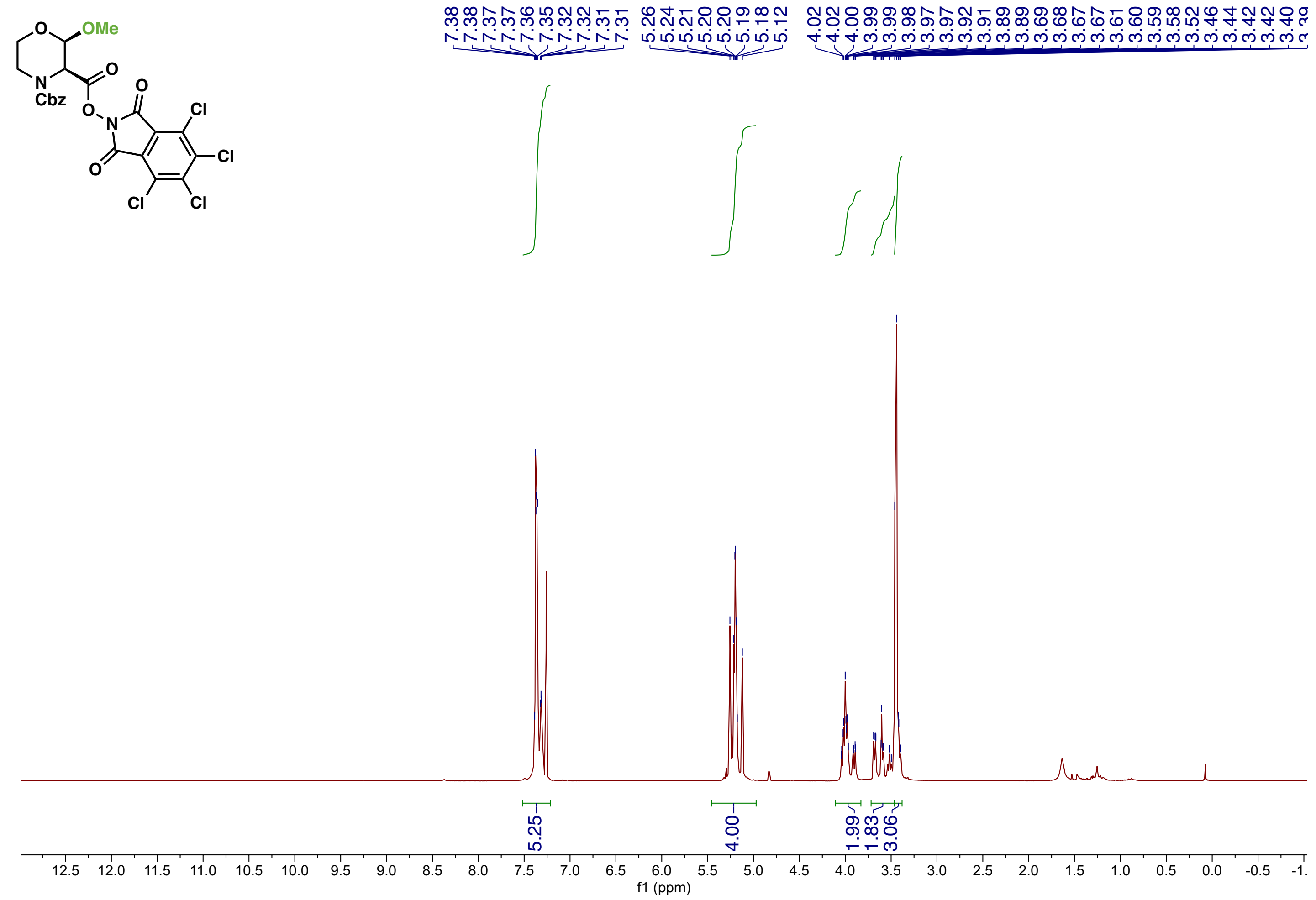

Compound B16 ^{13}C NMR

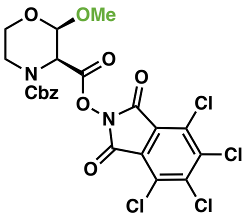

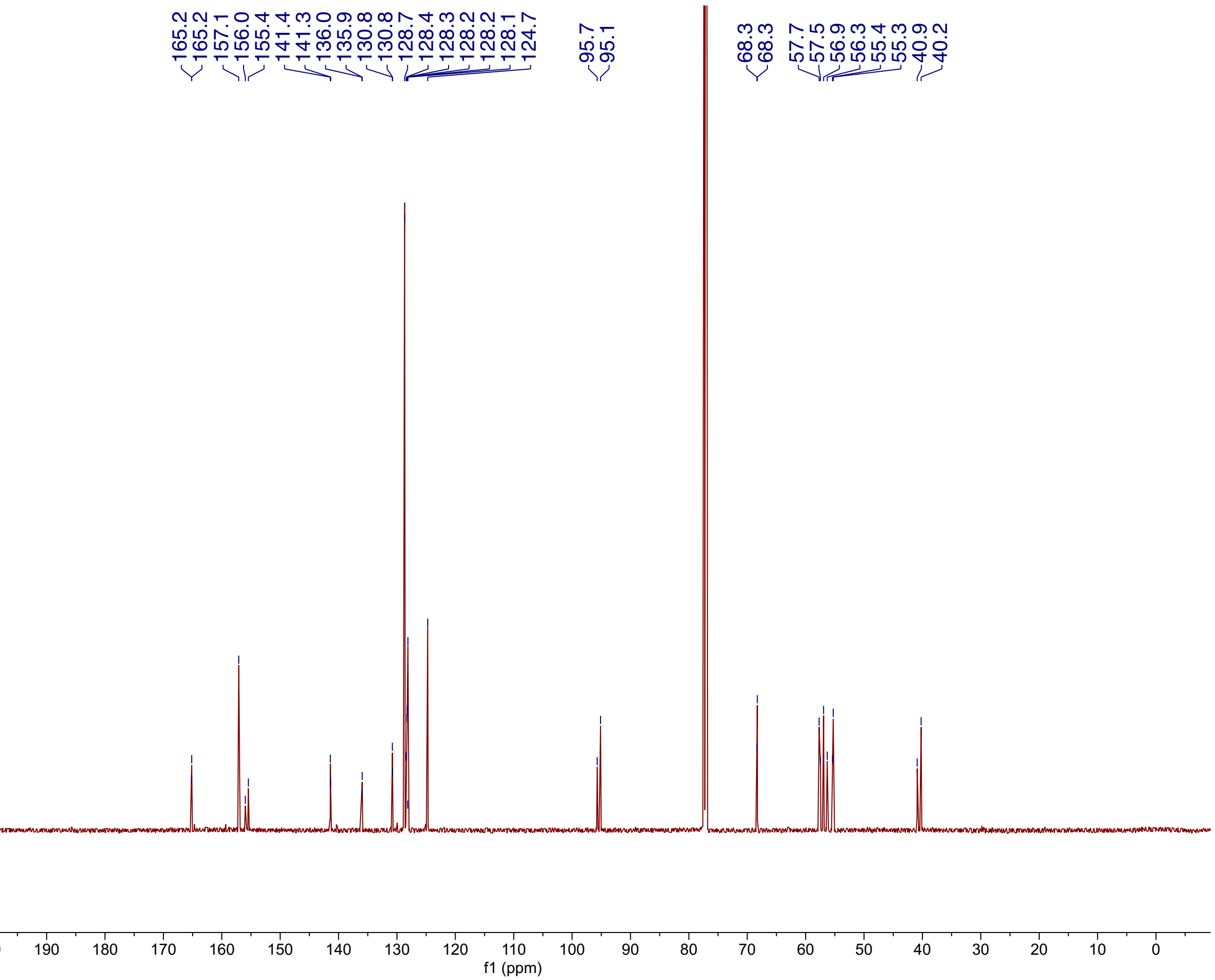

Compound 66 ^{1}H NMR

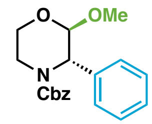

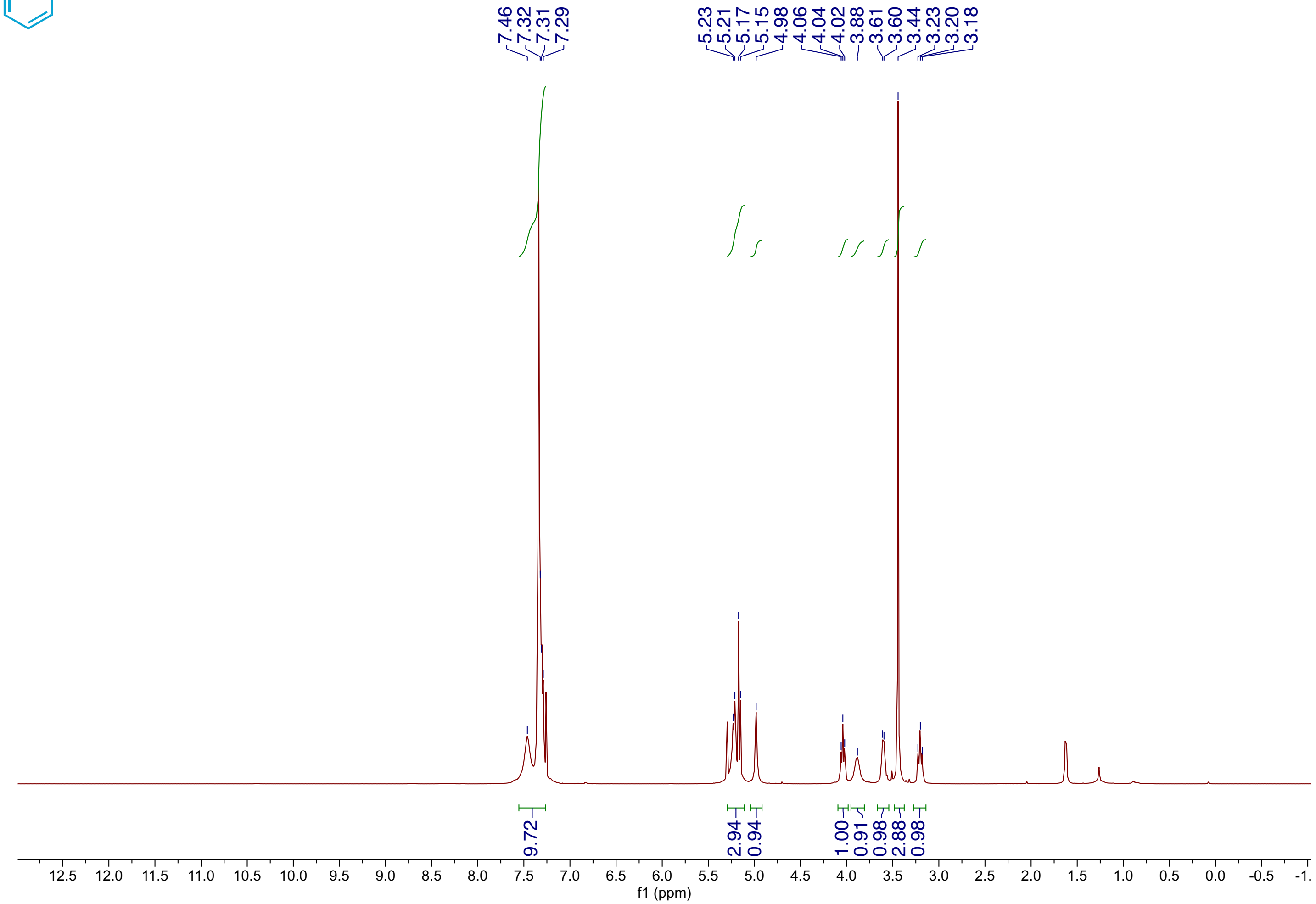

Compound 66 ^{13}C NMR

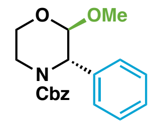

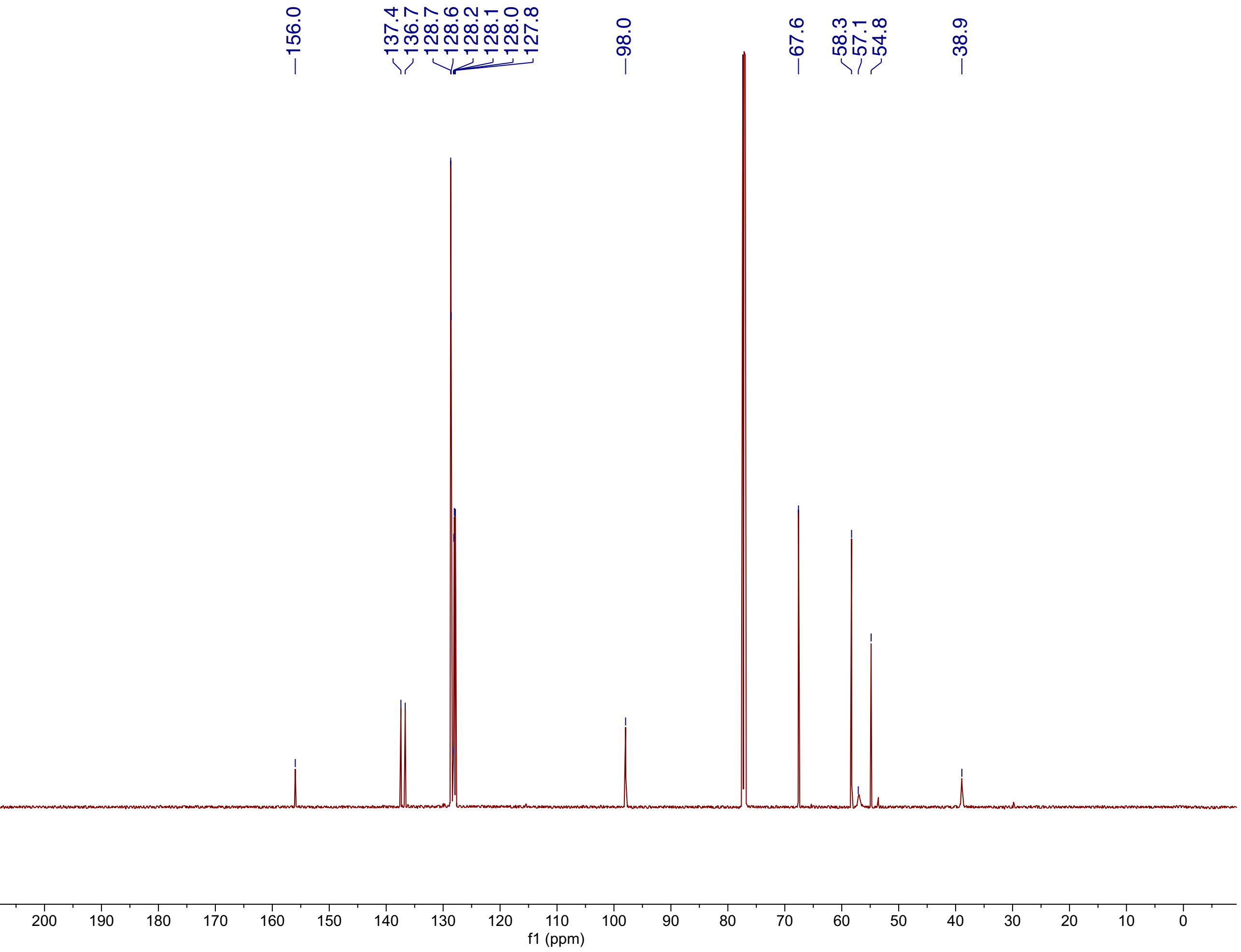

Compound 67 ^{1}H NMR

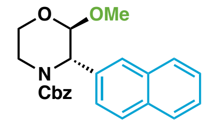

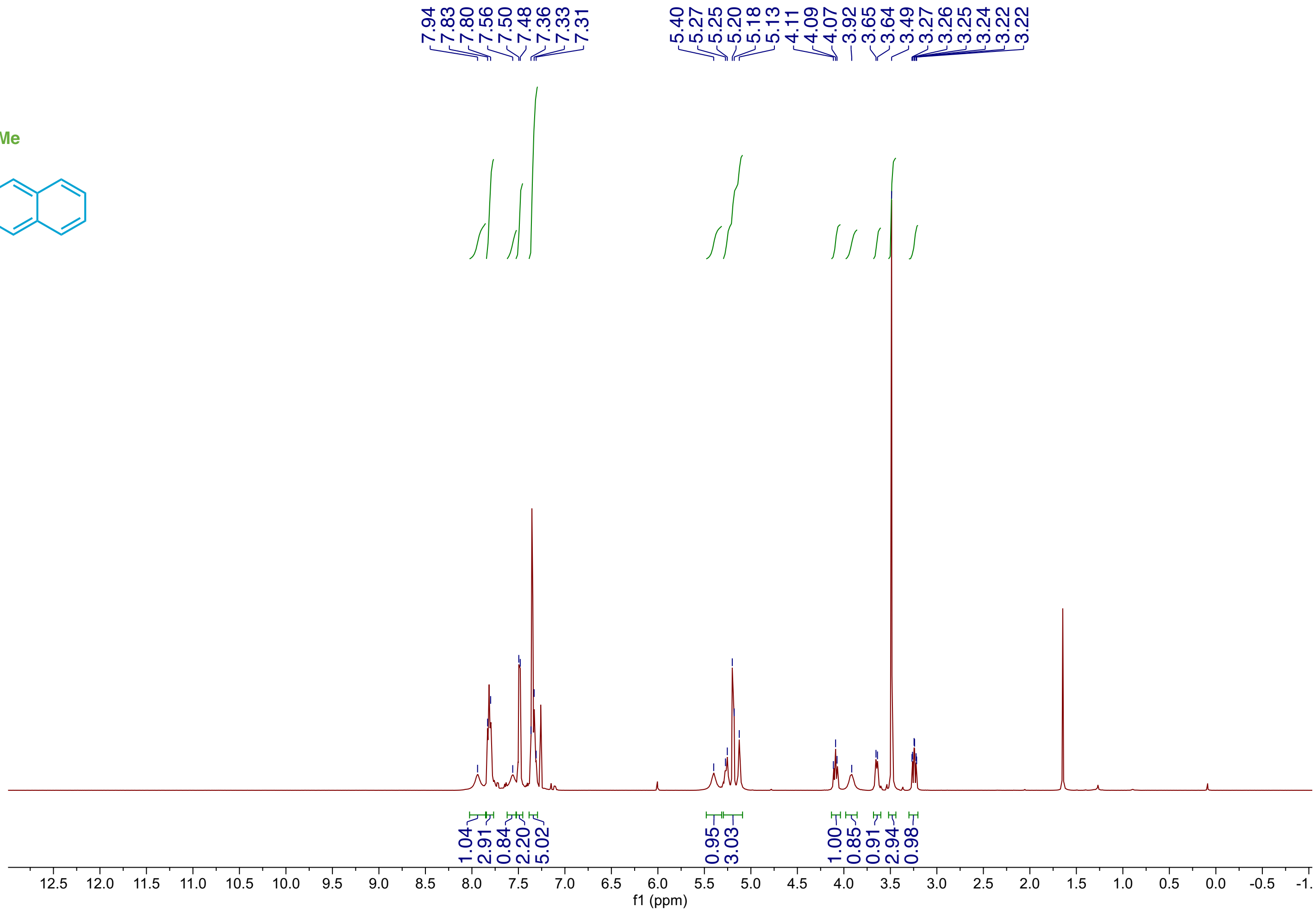

Compound 67 ^{13}C NMR

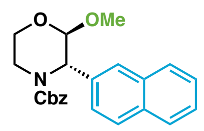

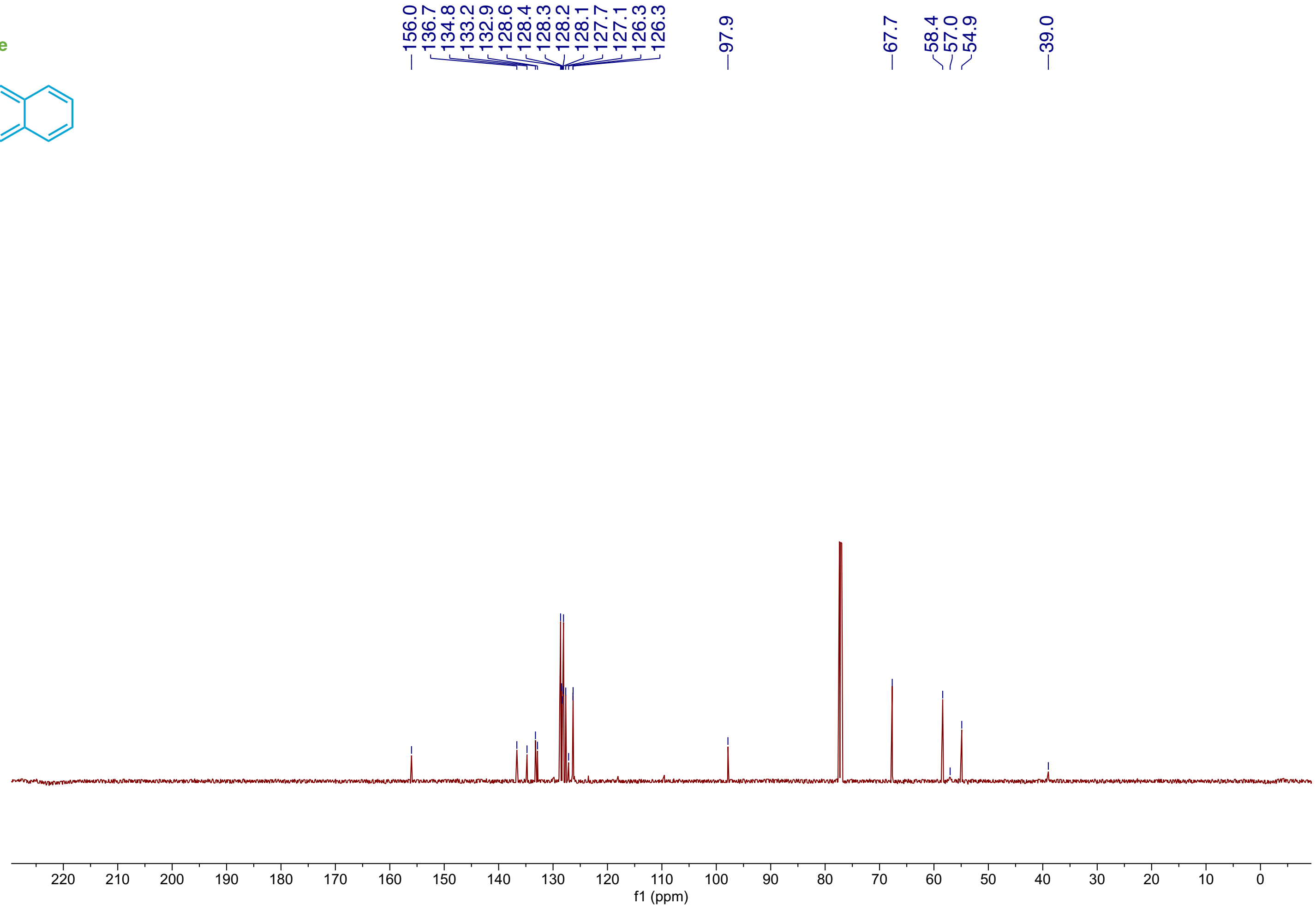

Compound 68 ^{1}H NMR

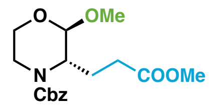

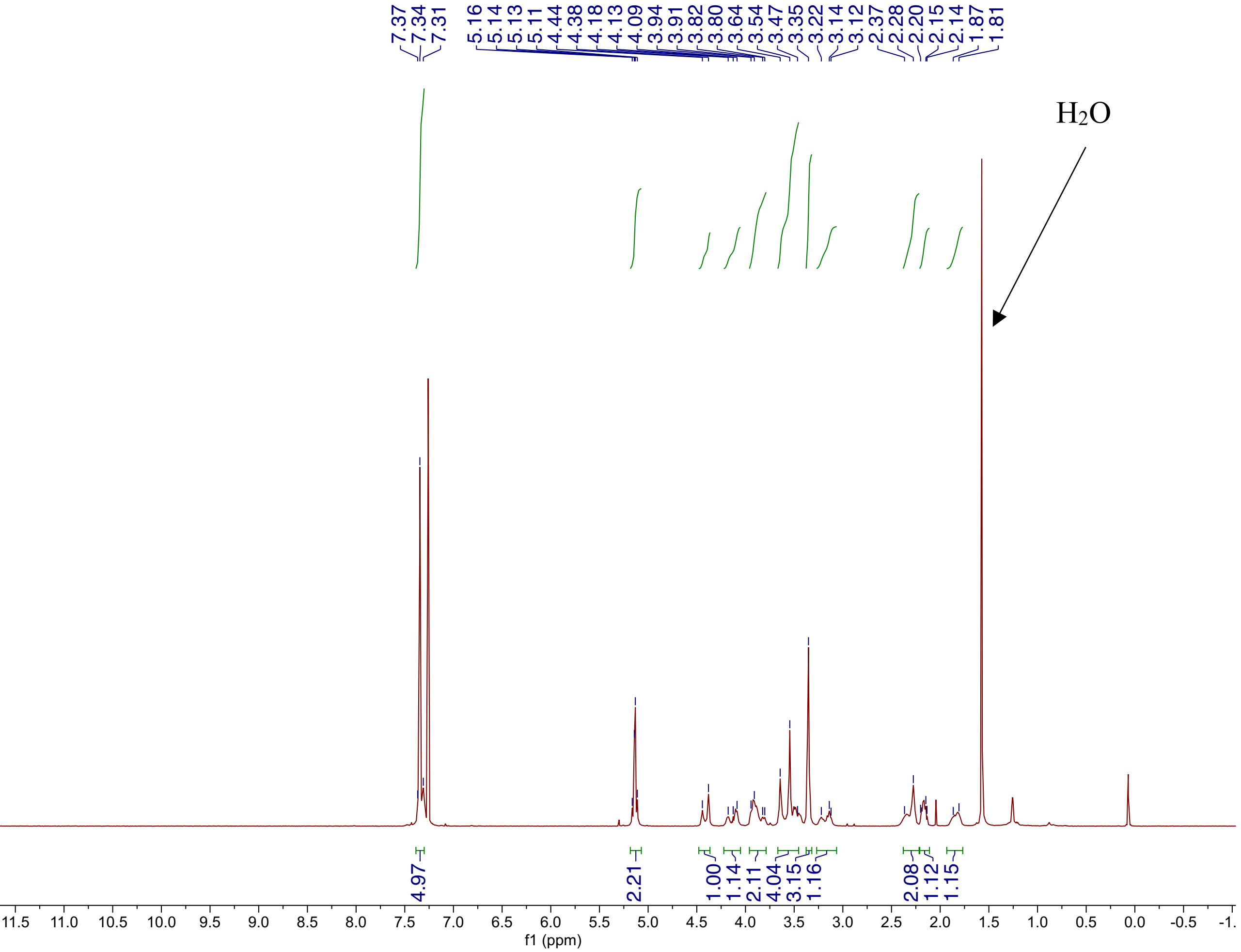

Compound 68 ^{13}C NMR

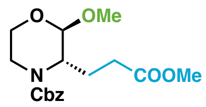

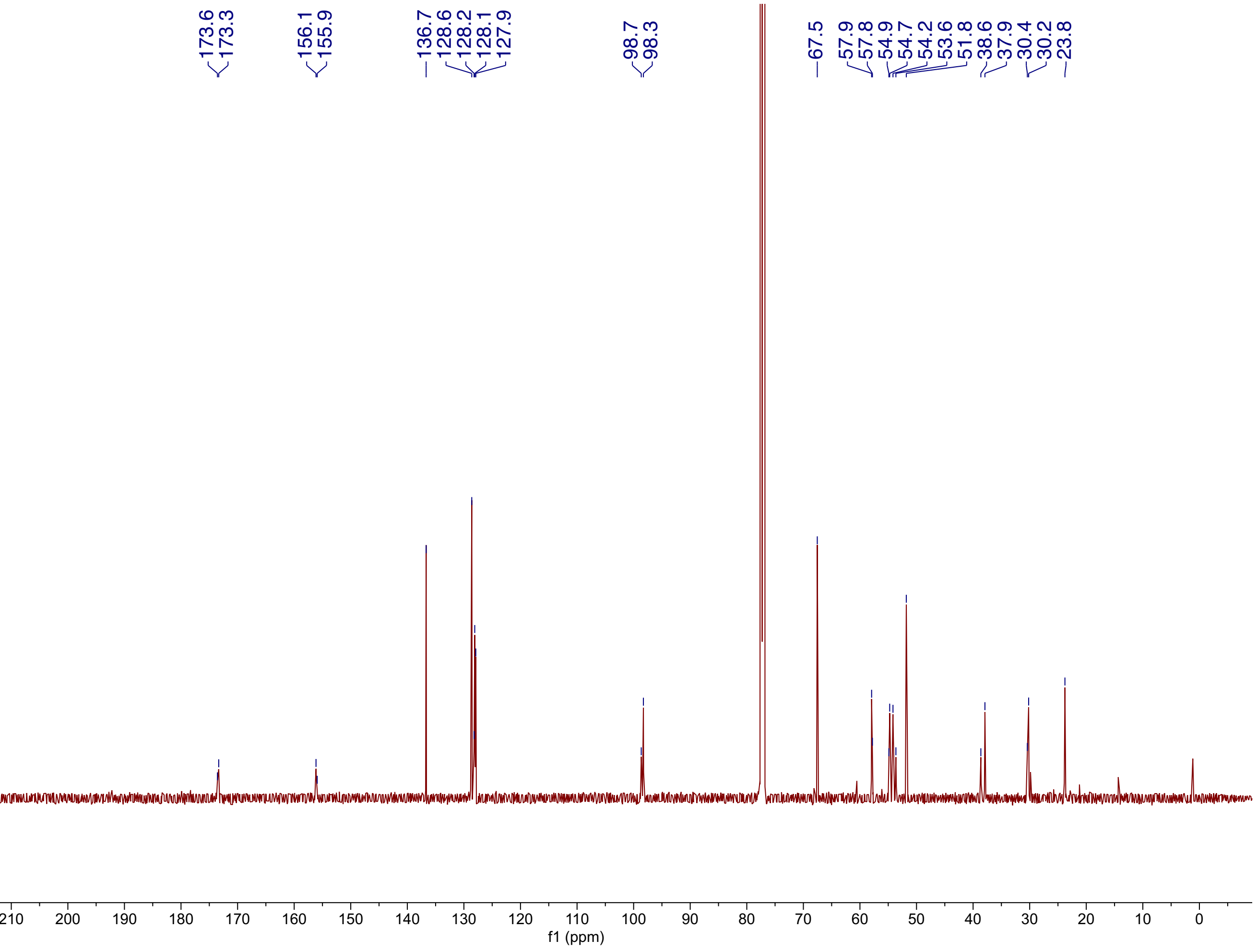

Compound SI-42 ^{1}H NMR

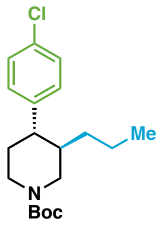

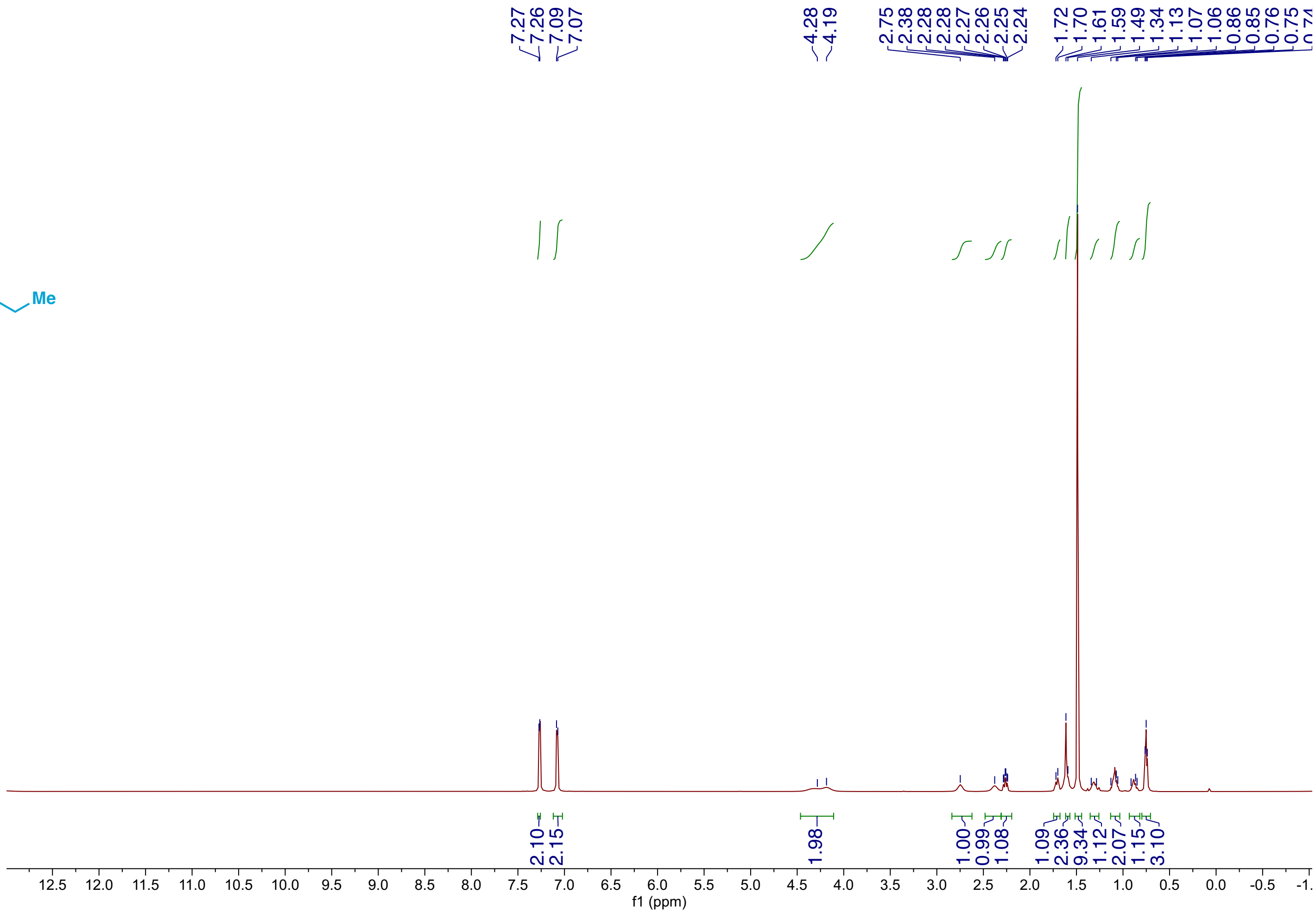

Compound SI-42 ^{13}C NMR

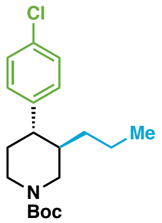

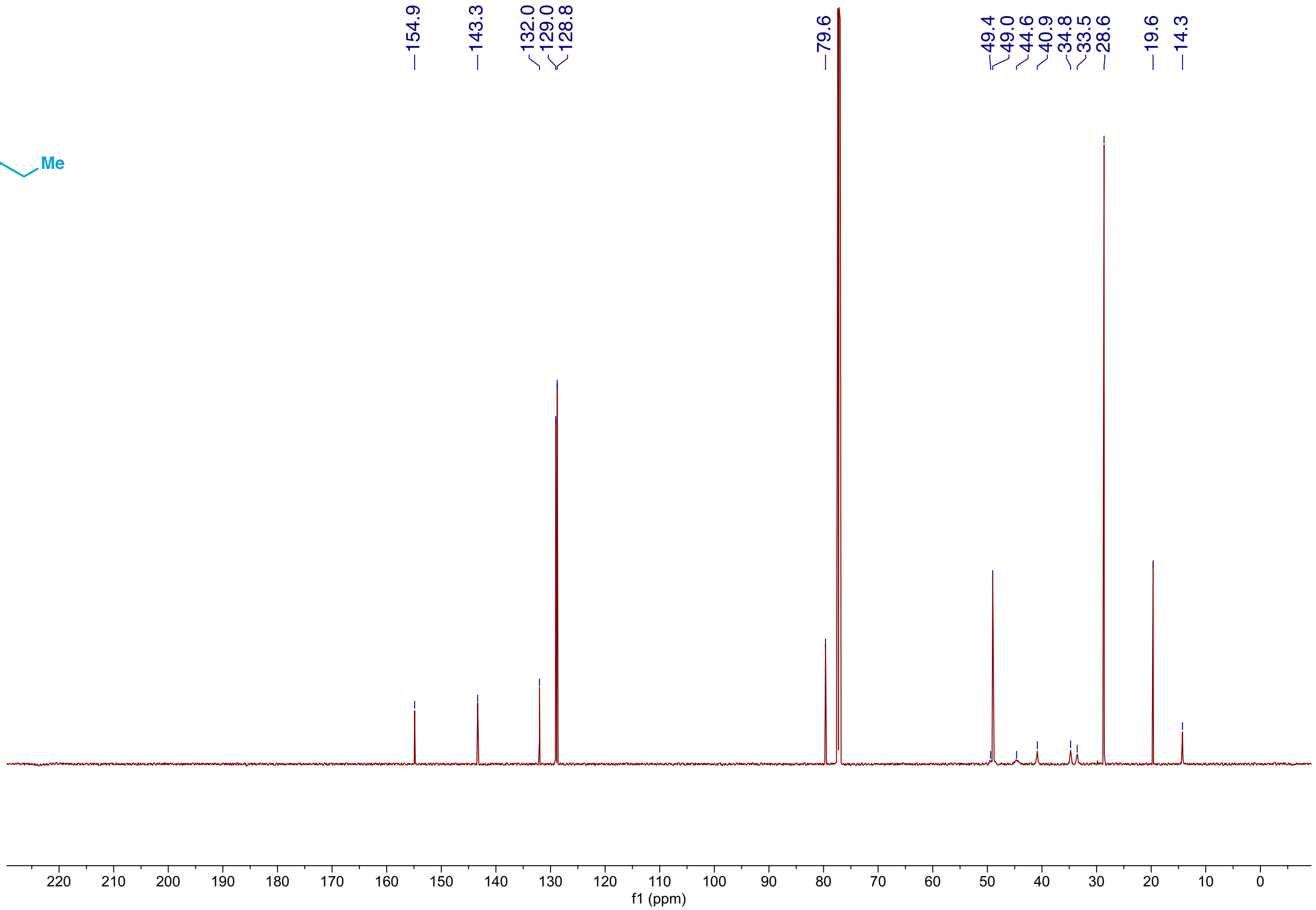

Compound 69 ¹H NMR

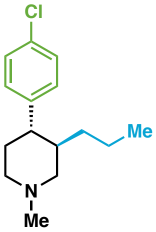

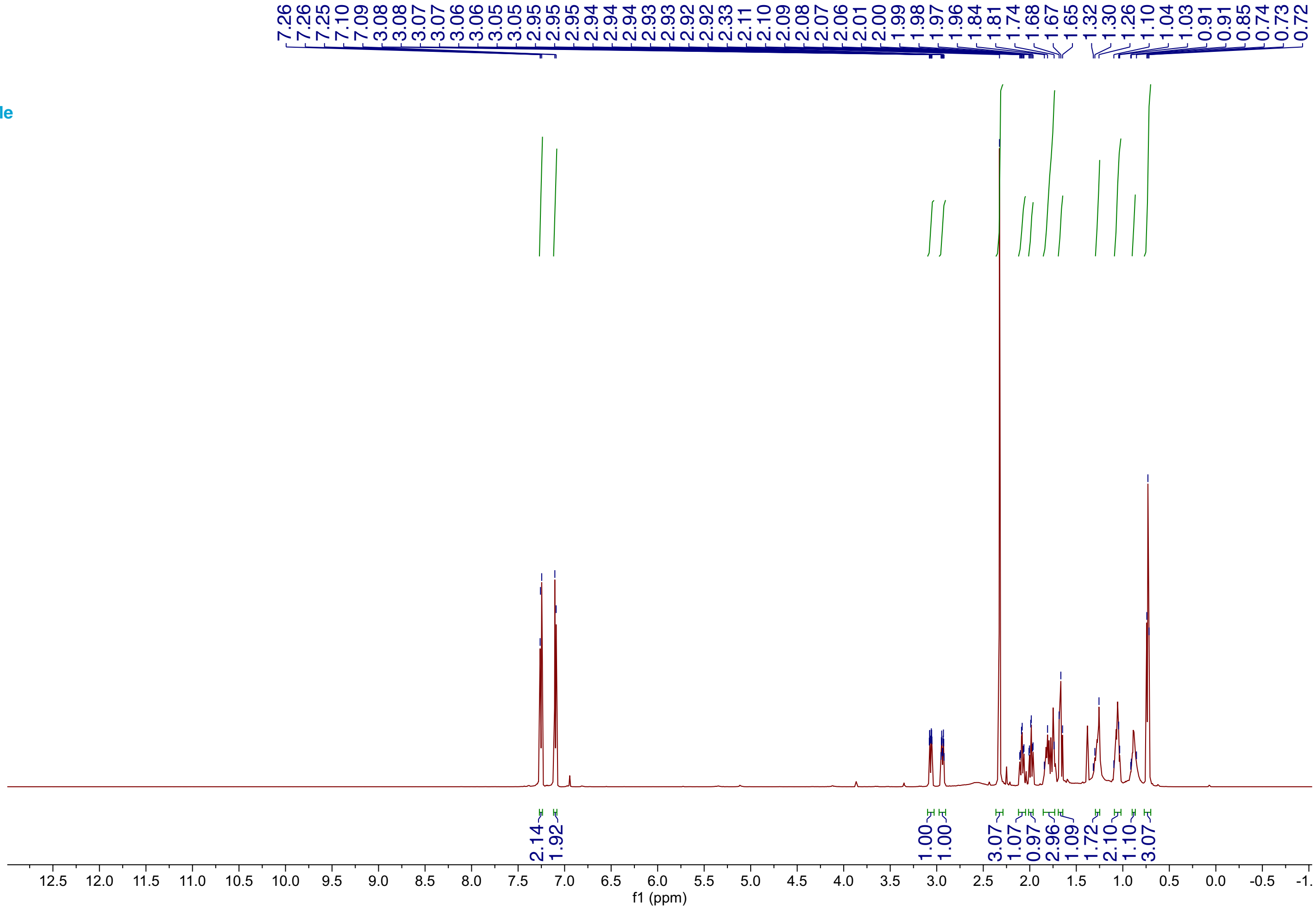

Compound 69 ^{13}C NMR

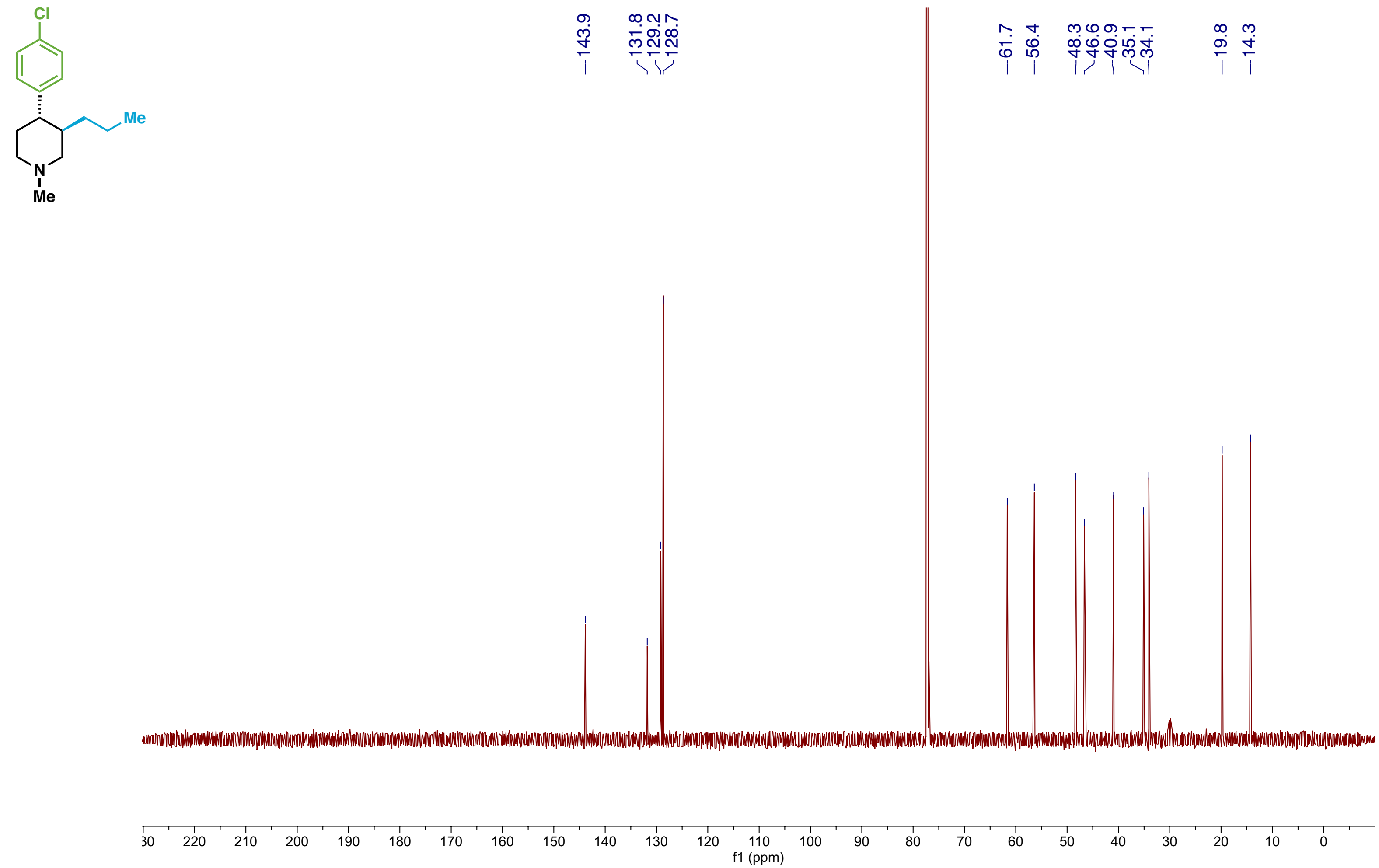

Compound SI-43 ¹H NMR

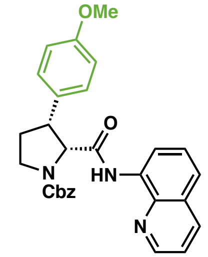

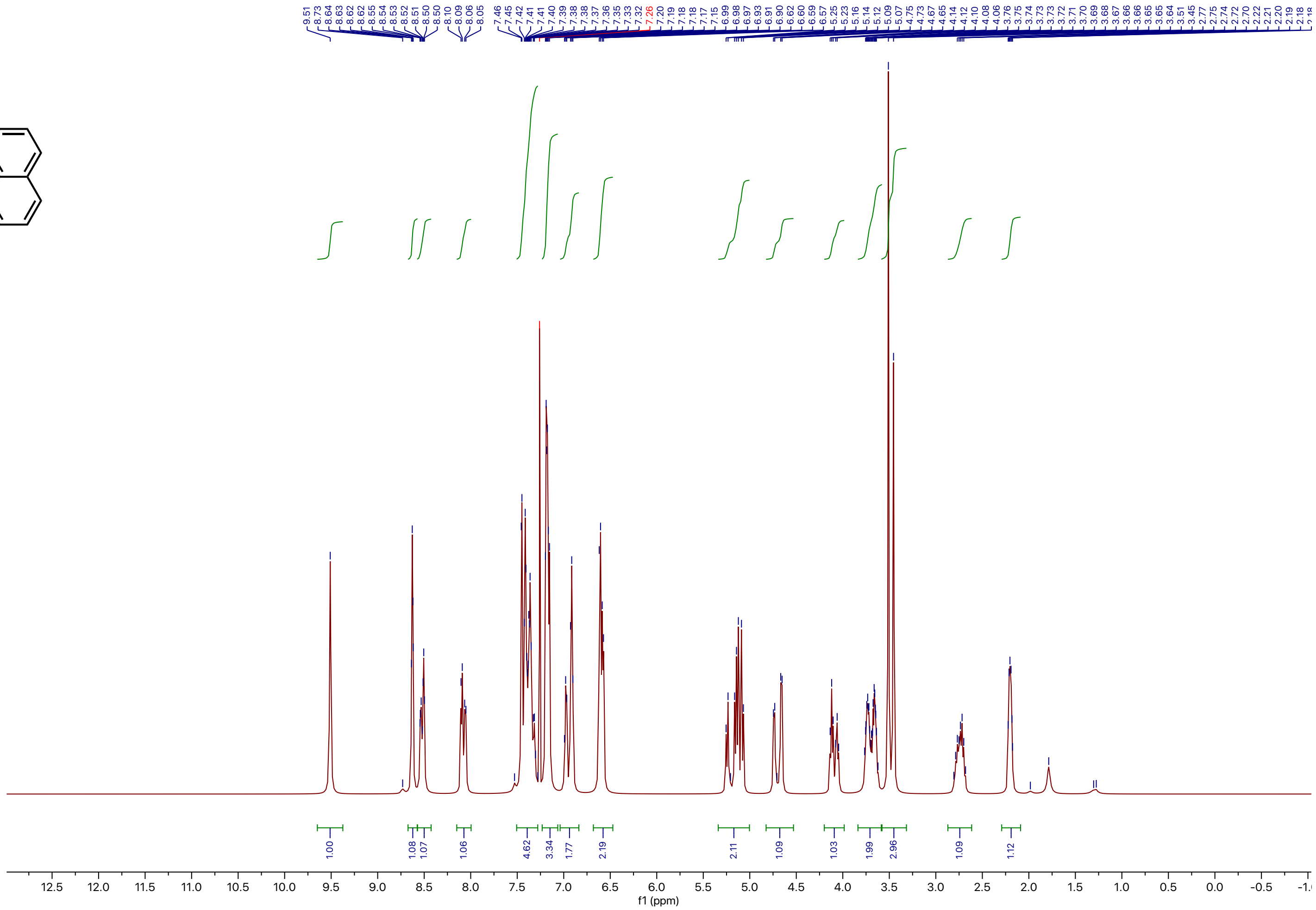

Compound SI-43 ^{13}C NMR

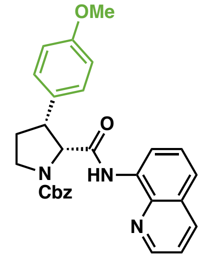

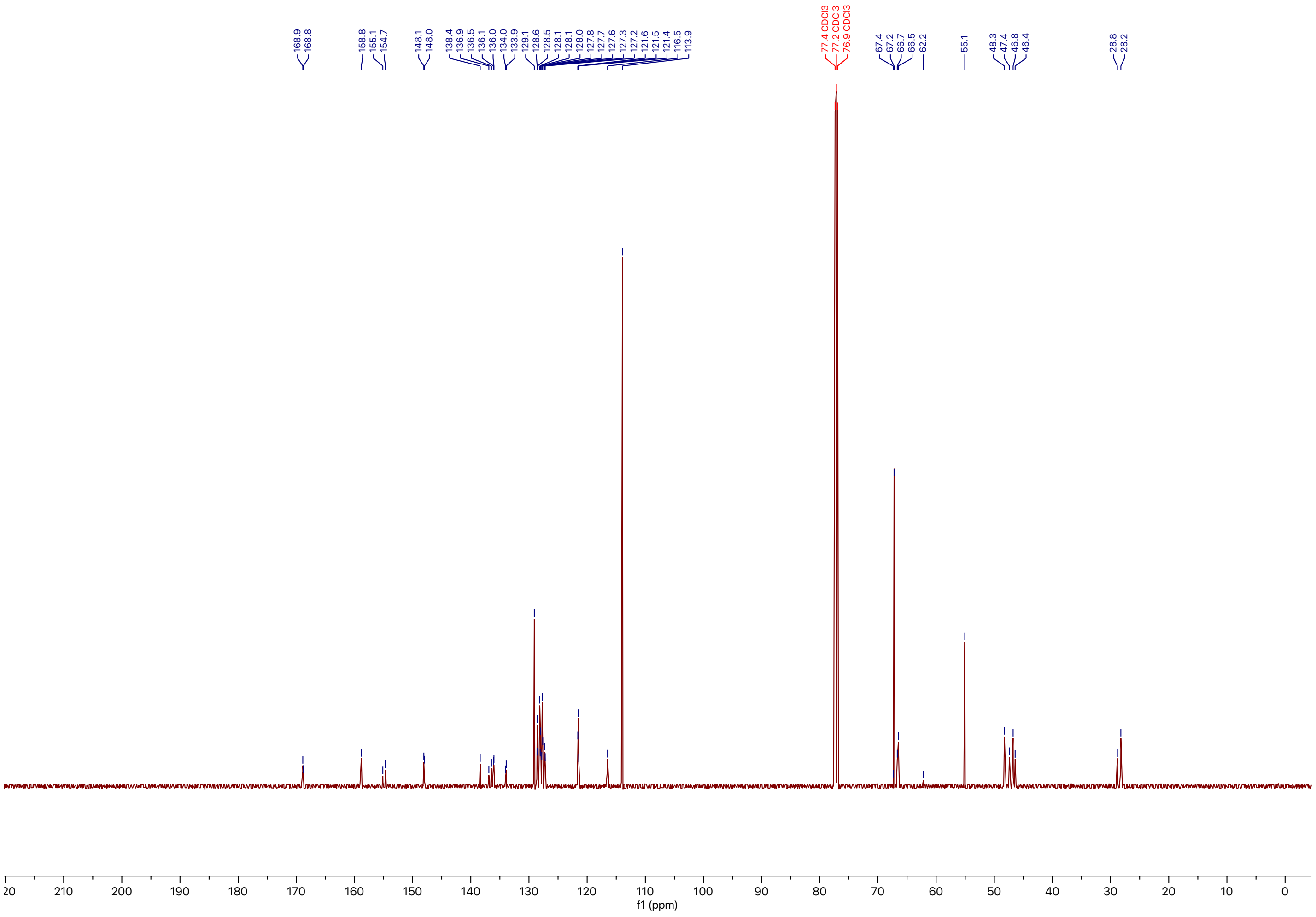

Compound A13 ¹H NMR

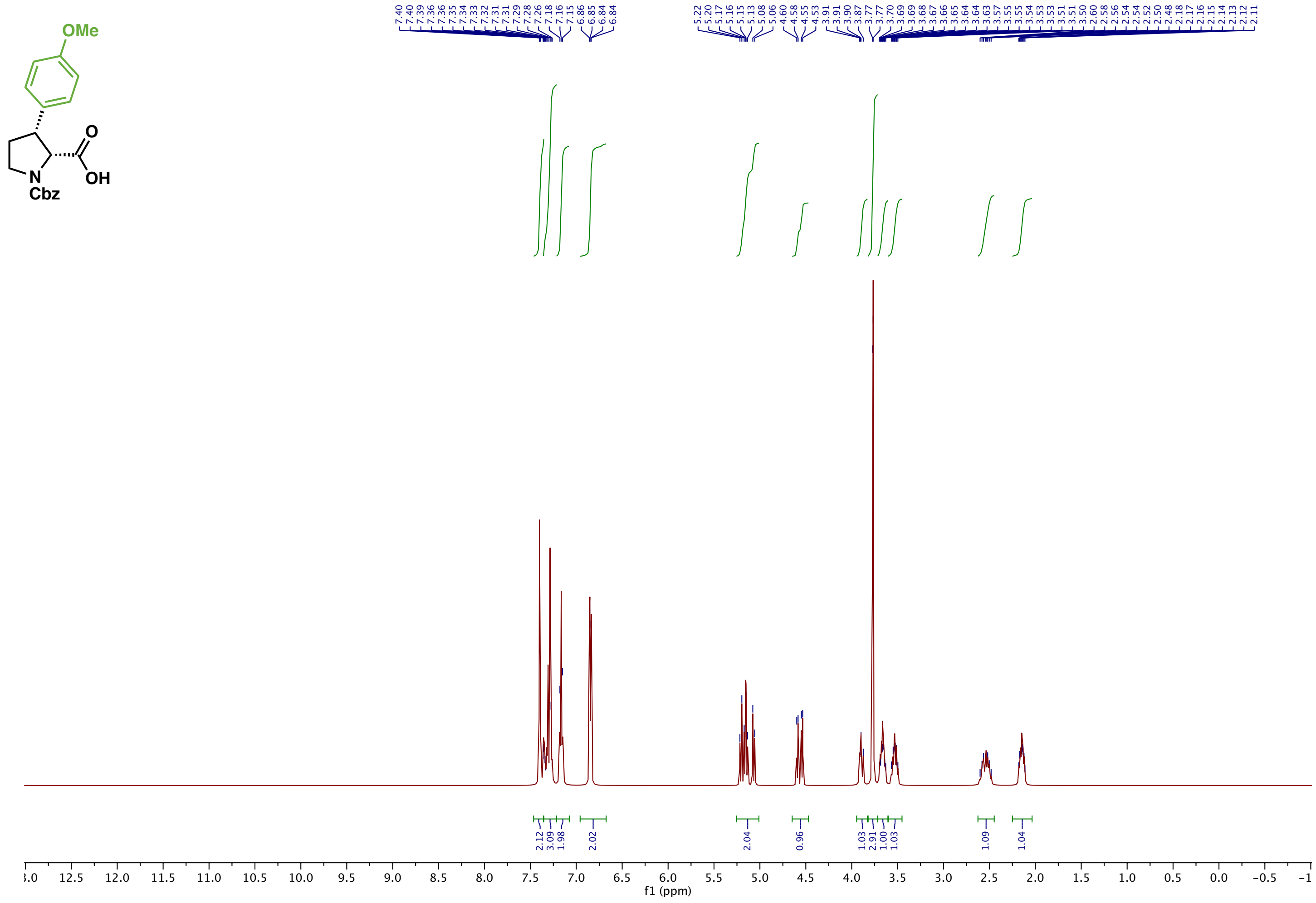

Compound A13 ^{13}C NMR

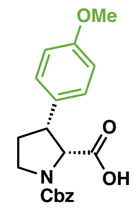

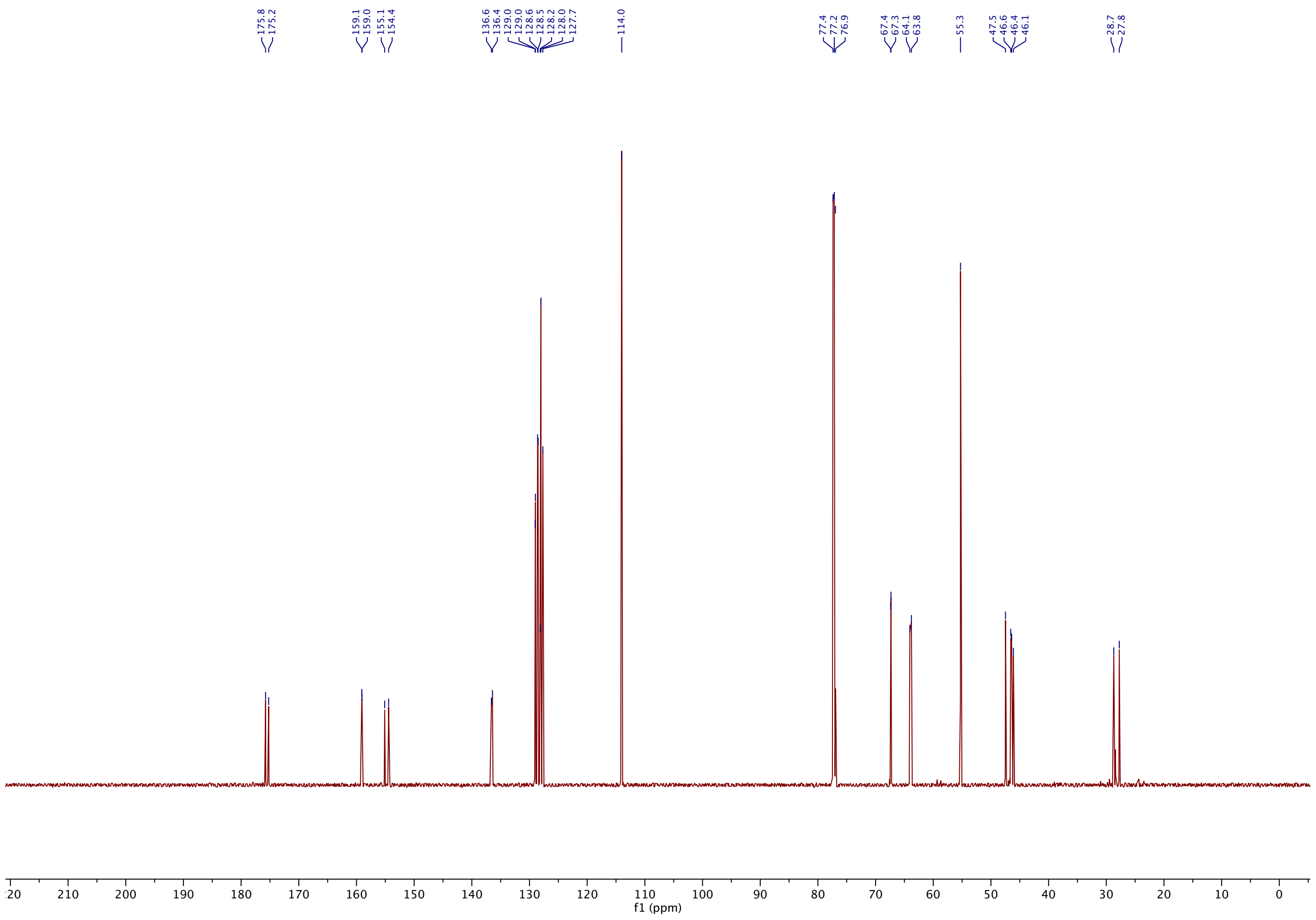

Compound 5 ^{1}H NMR

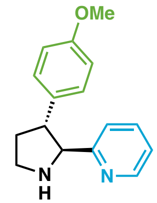

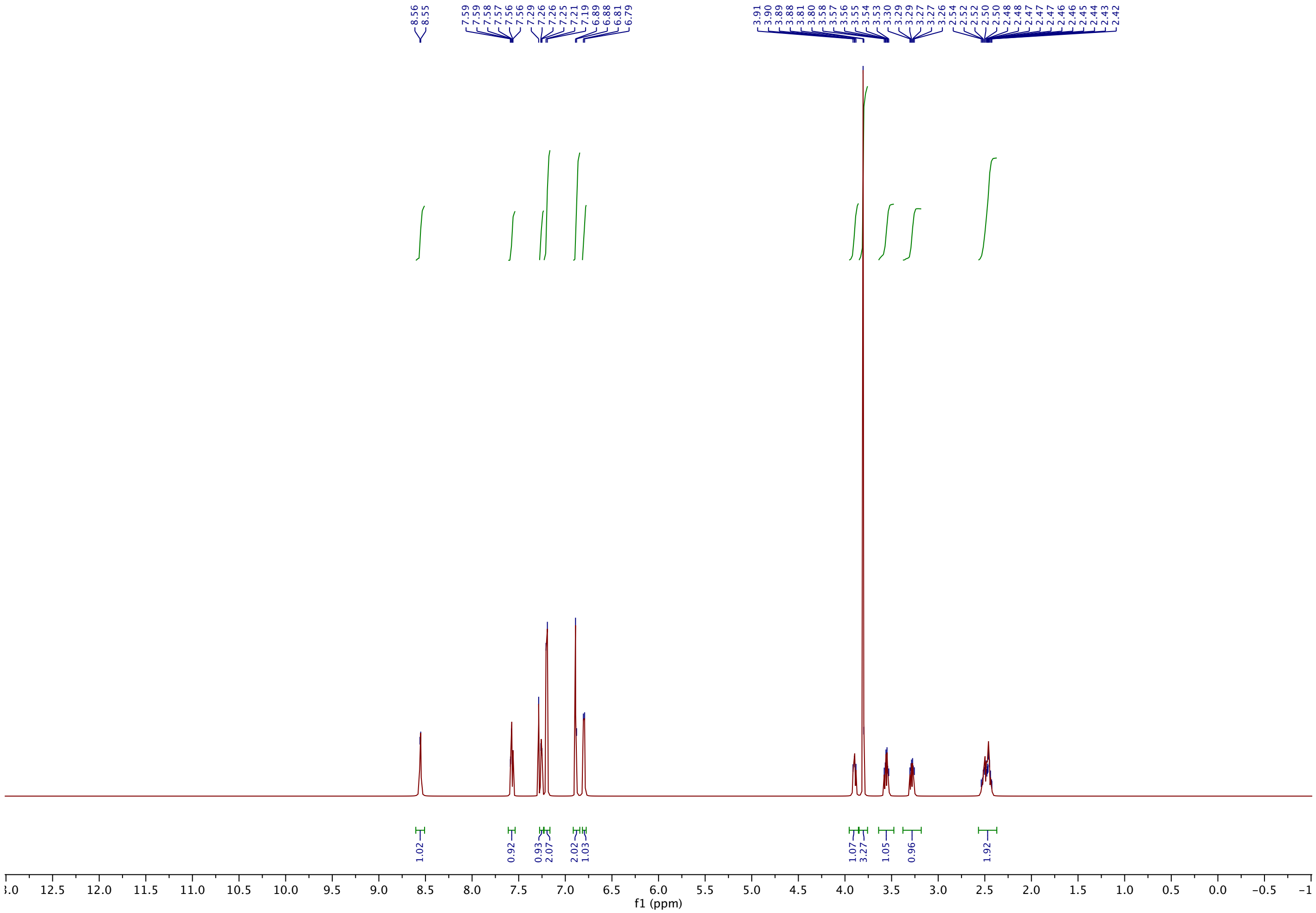

Compound SI-44 – ^{1}HNMR

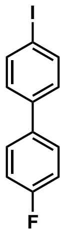

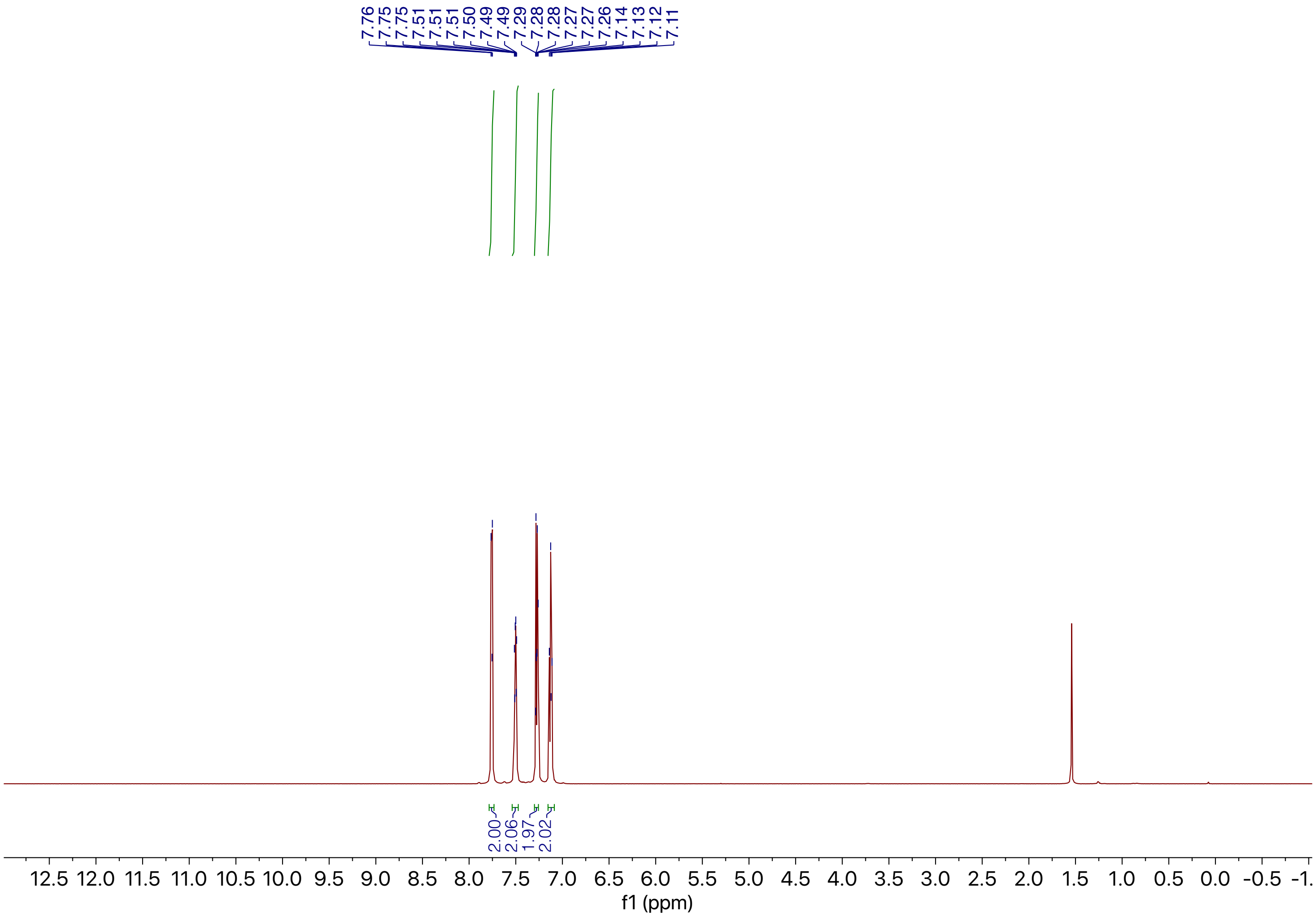

Compound SI-44 ^{19}F NMR

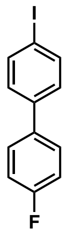

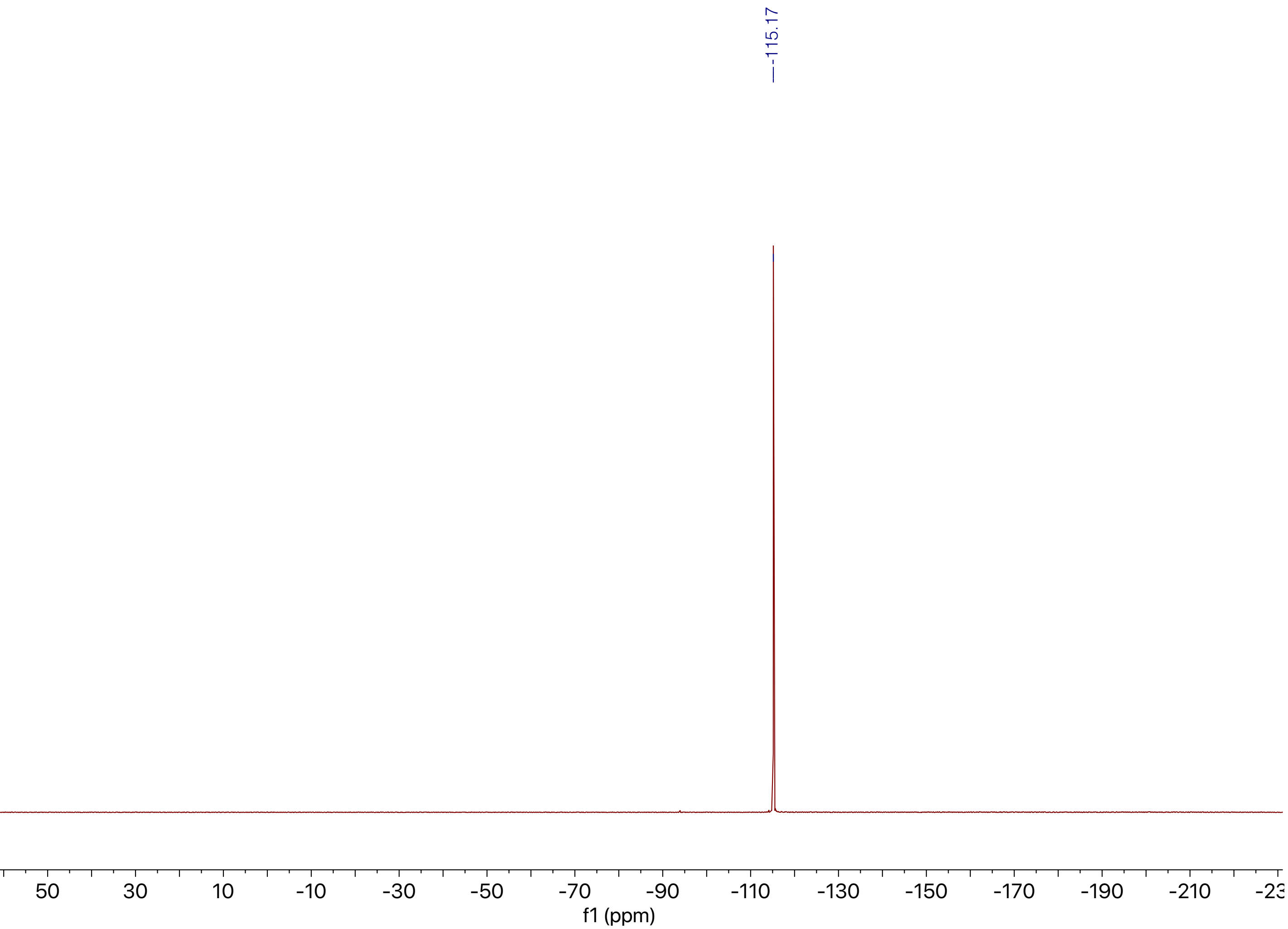

Compound SI-44 – ^{13}C NMR

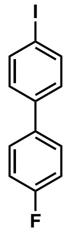

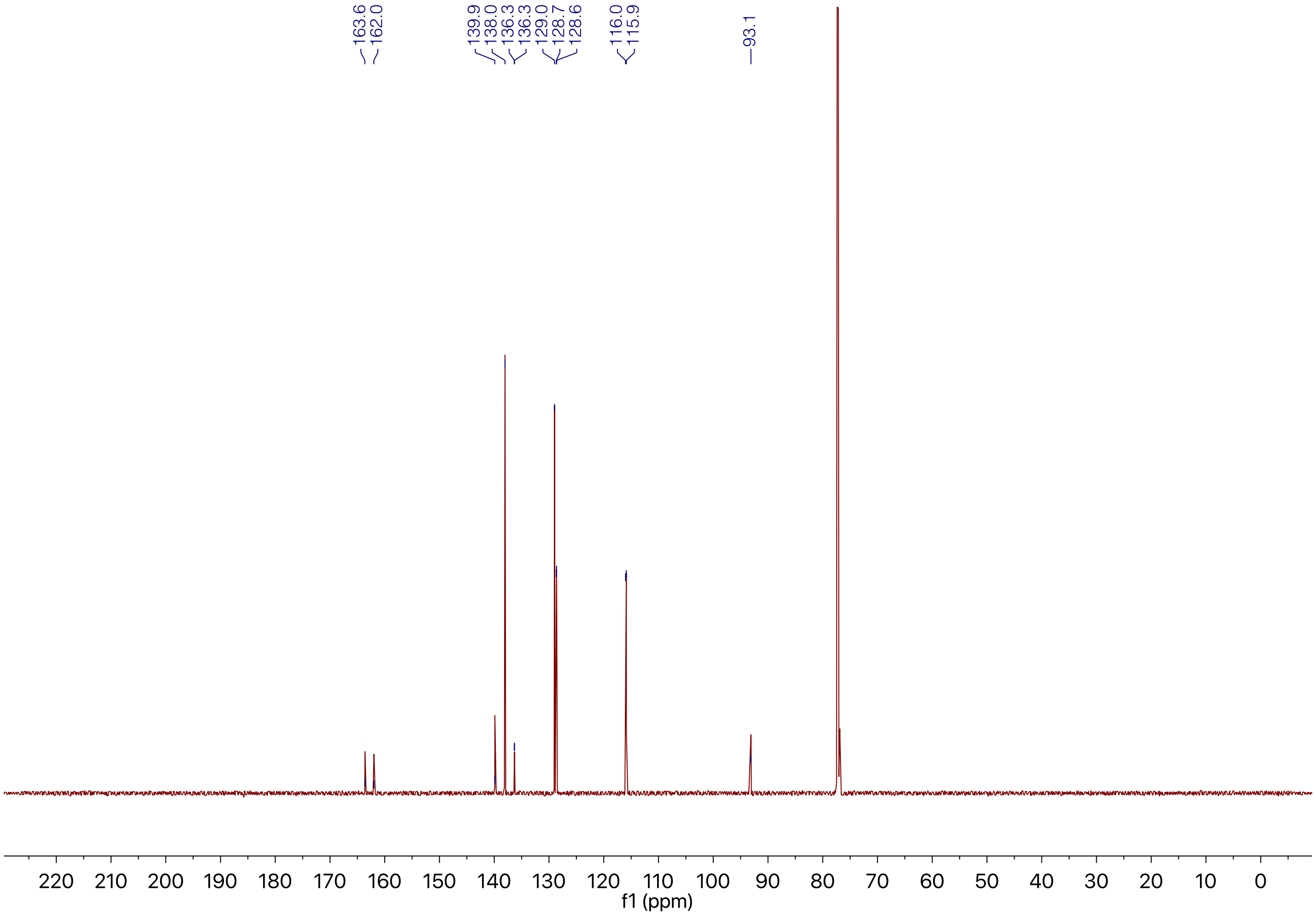

Compound 74 – ^{1}H NMR

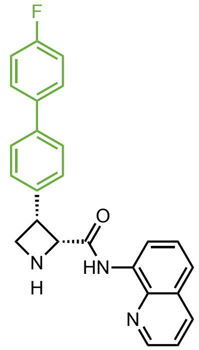

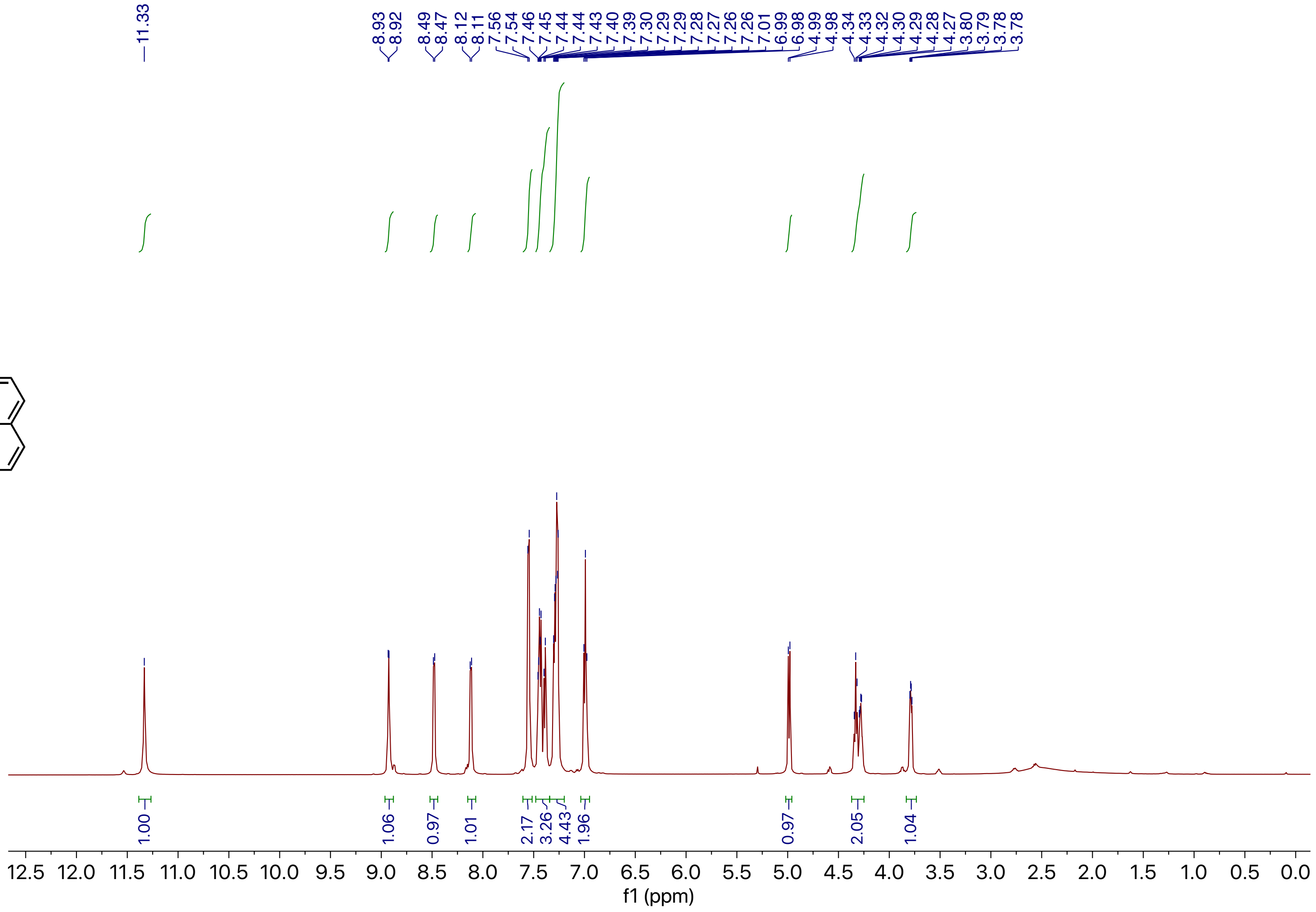

Compound 74 – ^{19}F NMR

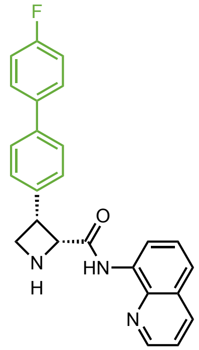

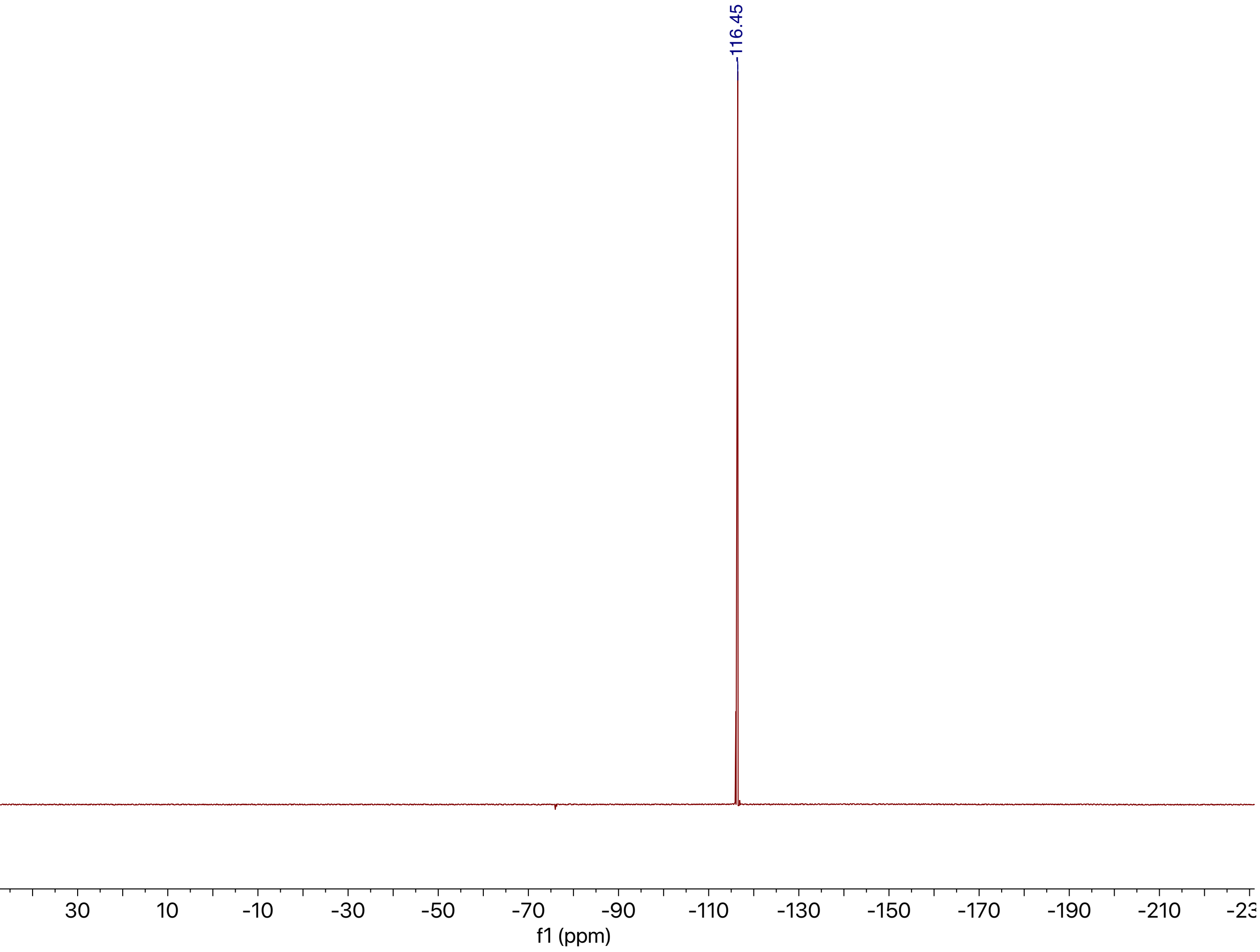

Compound 74 ^{13}C NMR

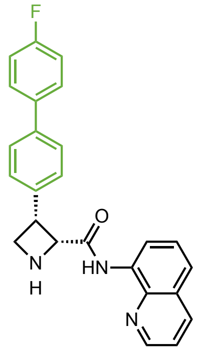

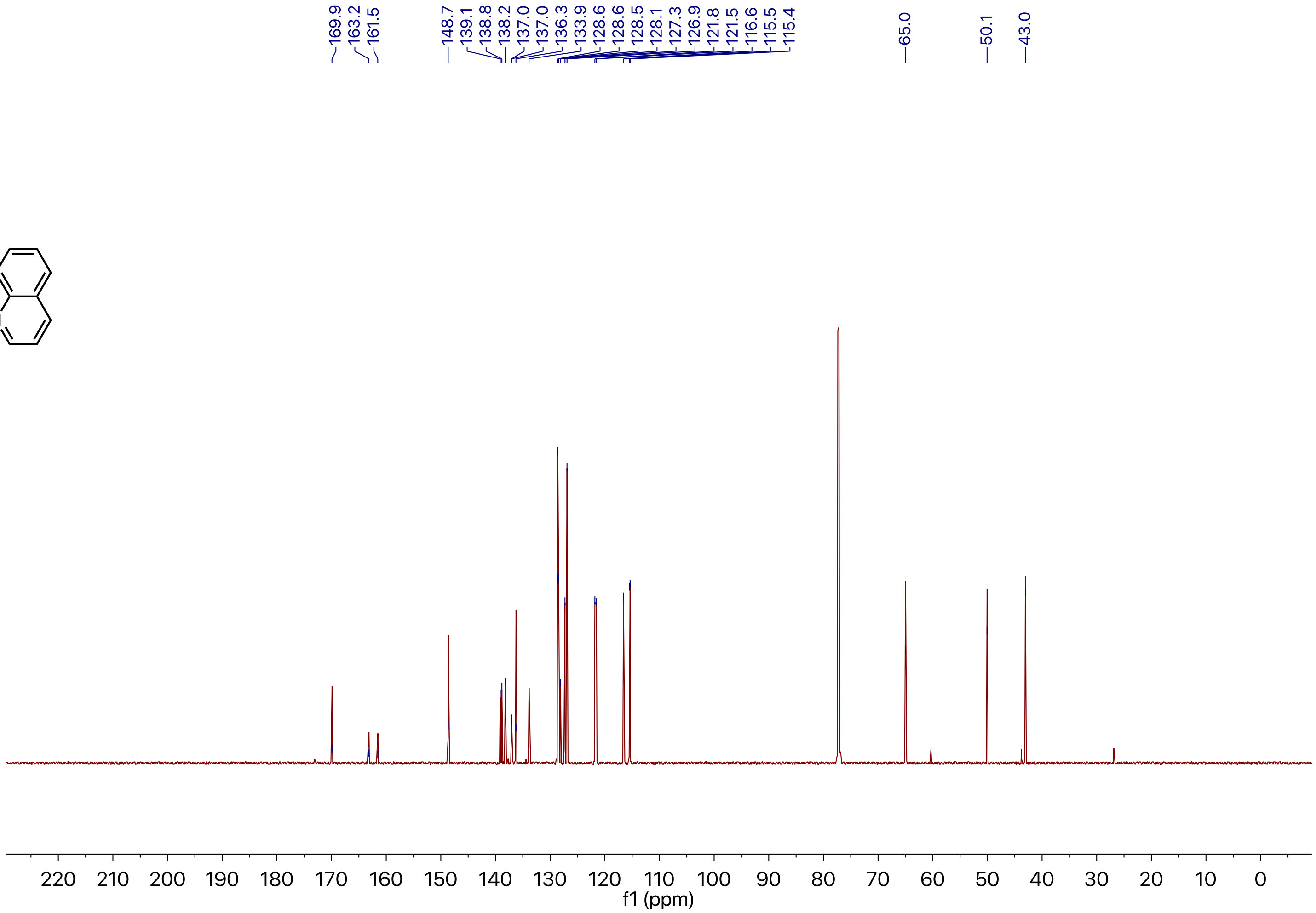

Compound SI-45– ^{1}H NMR

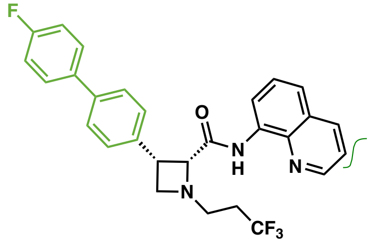

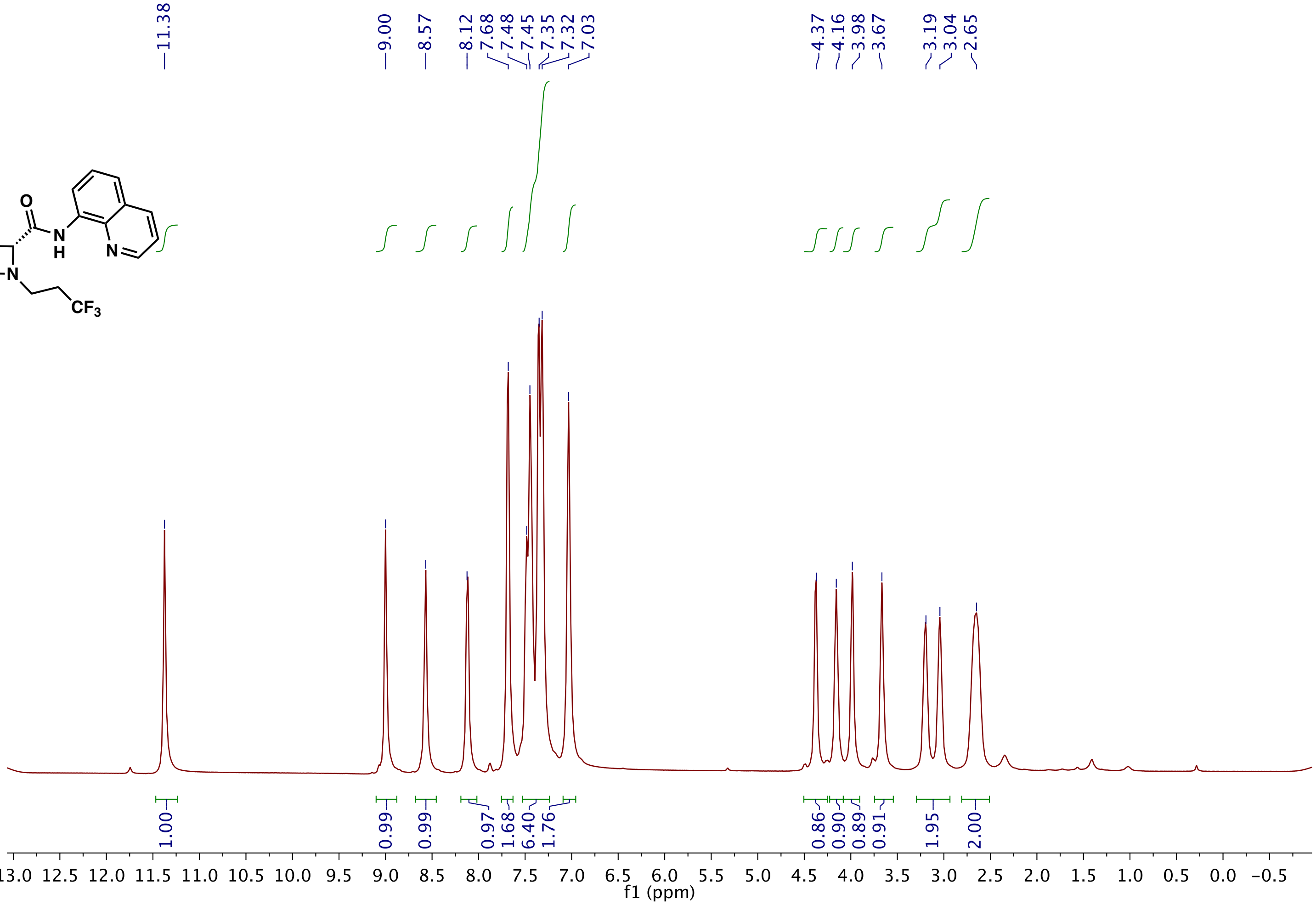

Compound SI-45 – ^{19}F NMR

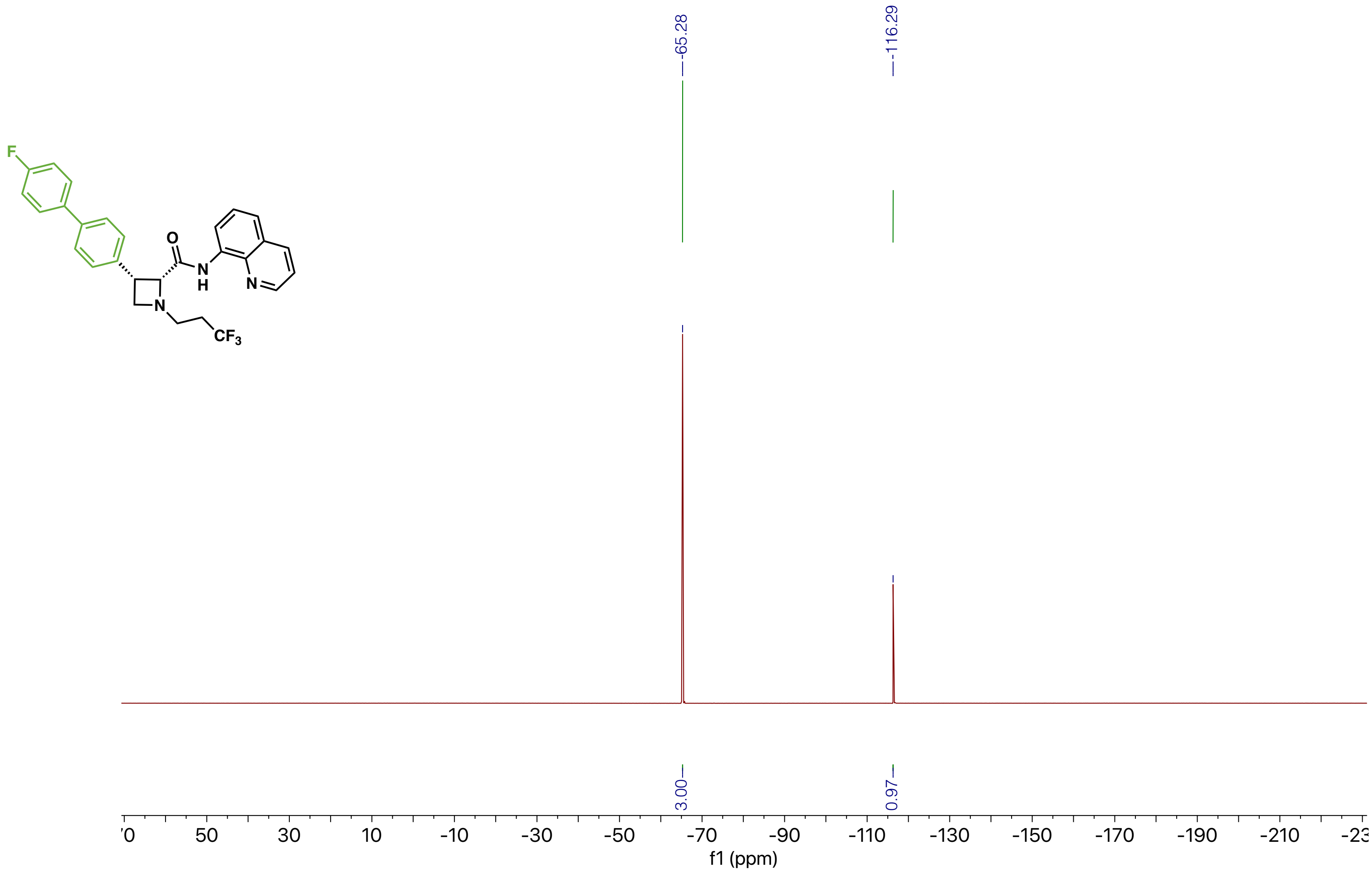

Compound SI-45 - ^{13}C NMR

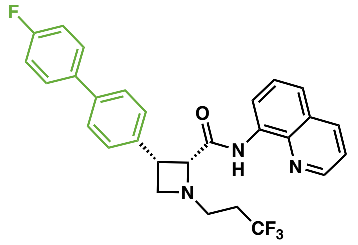

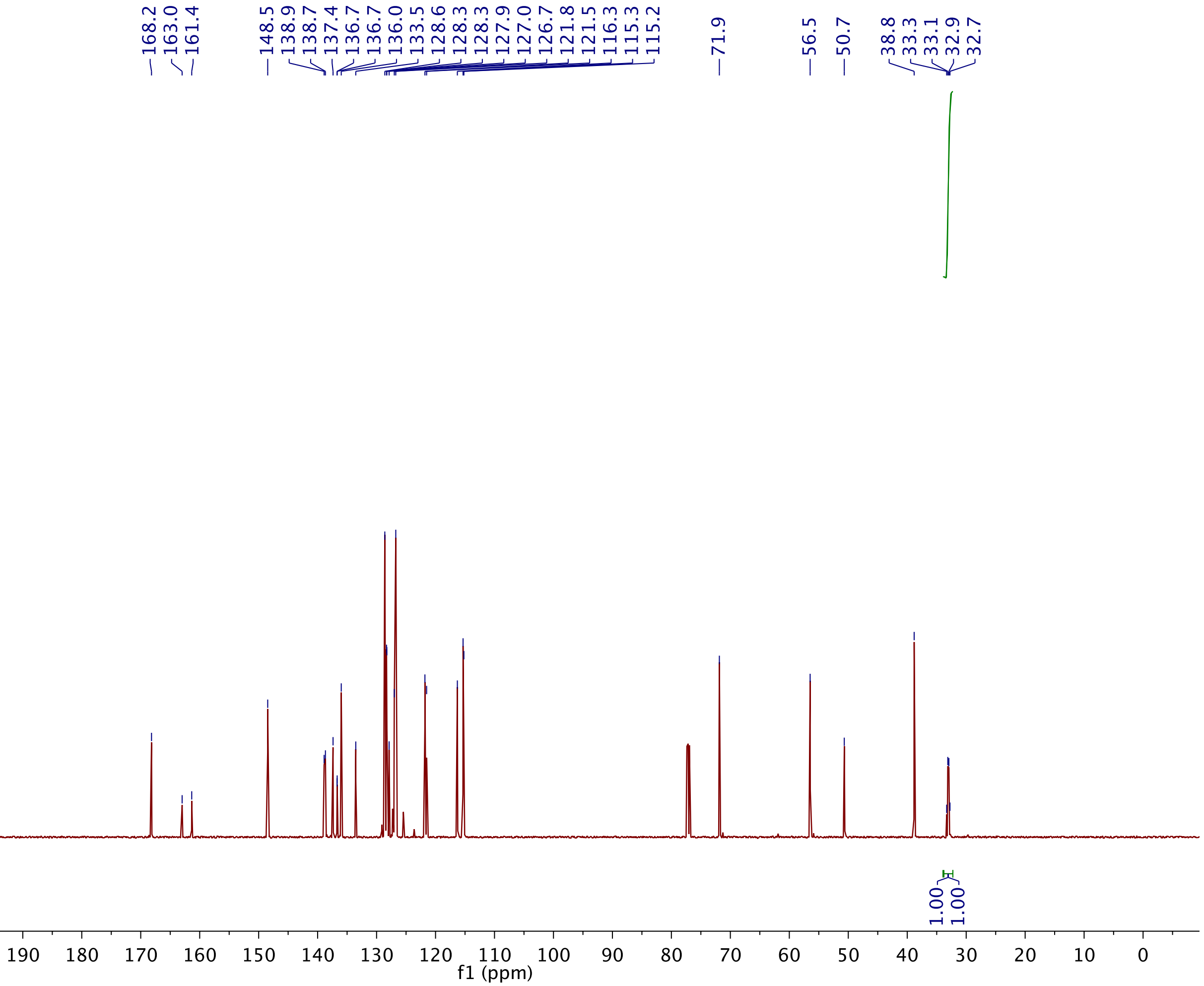

Compound A15 – ^{1}H NMR

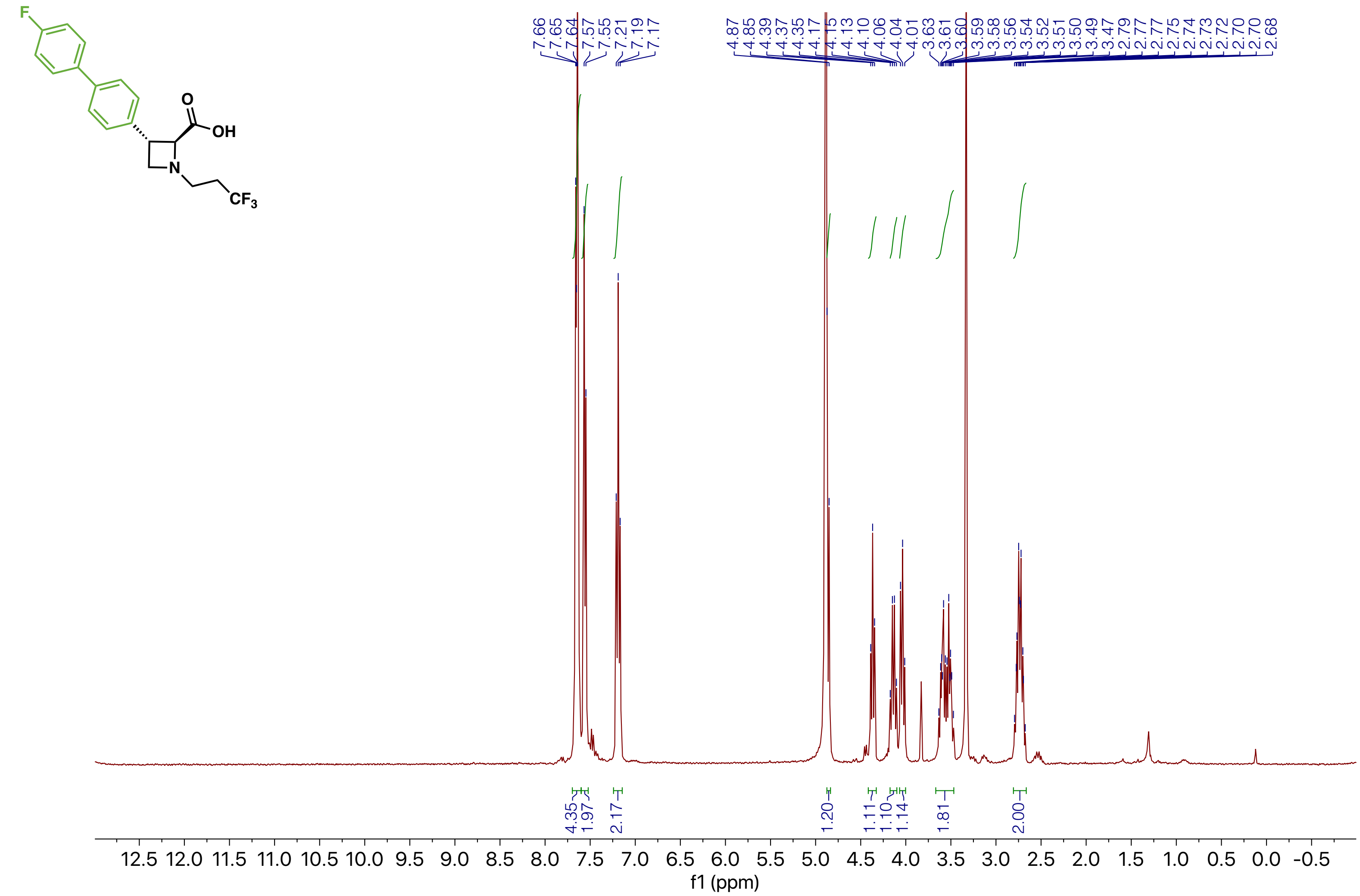

Compound A15 – ^{19}F NMR

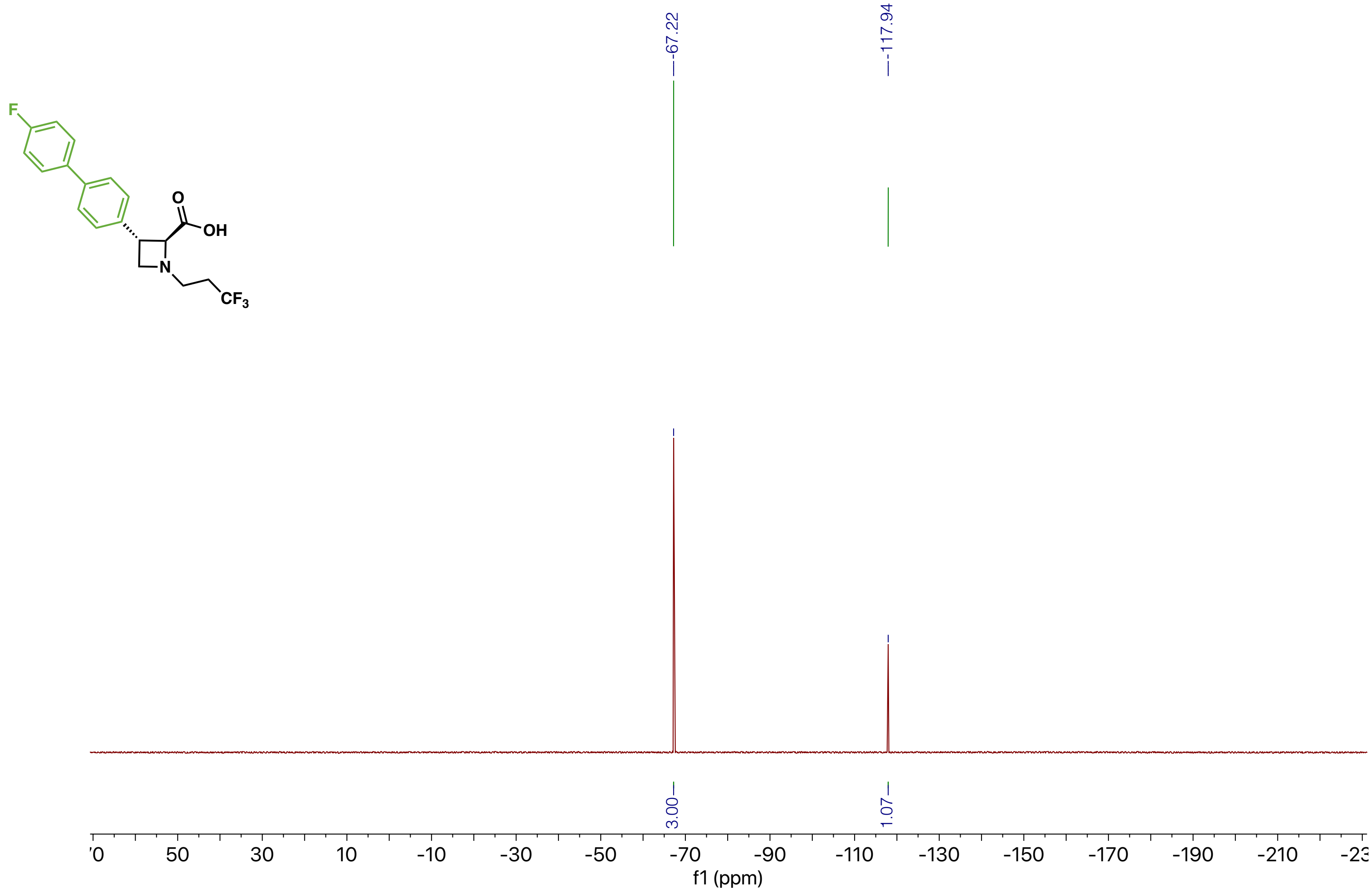

Compound A15- ^{13}C NMR

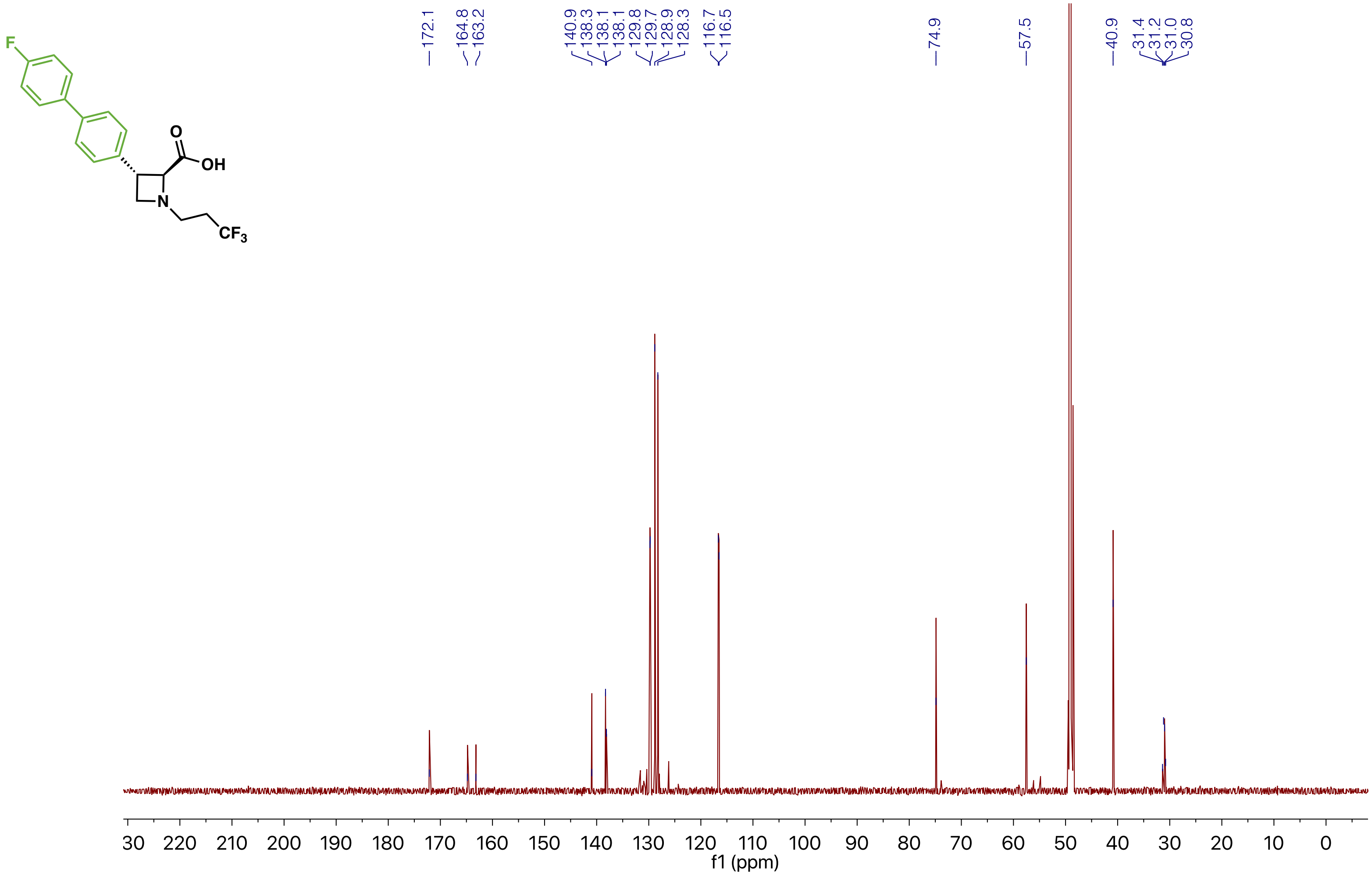

Compound A16 – ^{1}H NMR

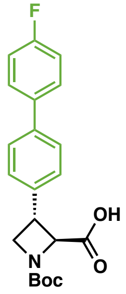

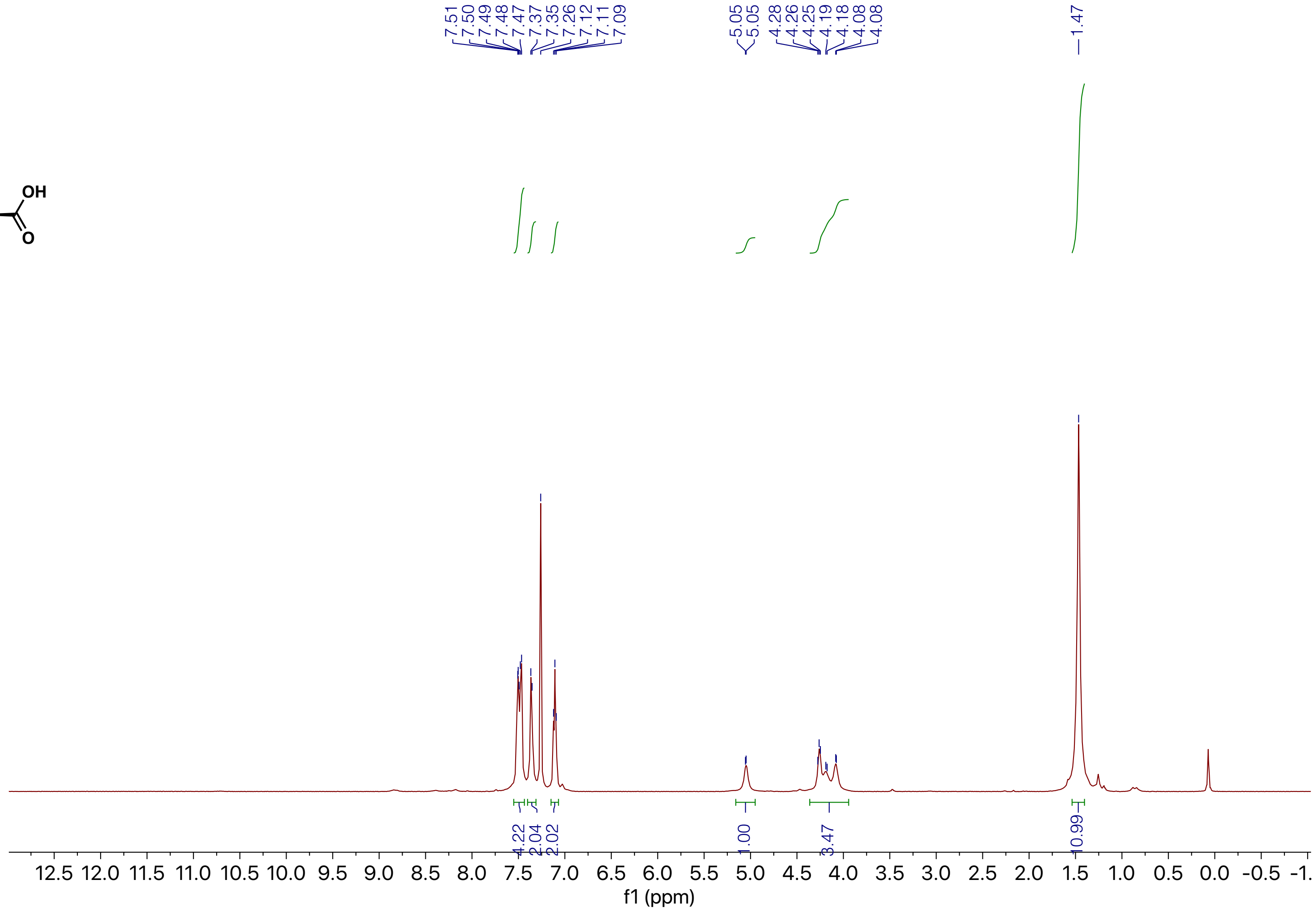

Compound A16 – ^{19}F NMR

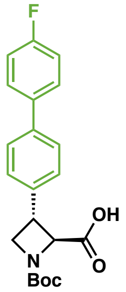

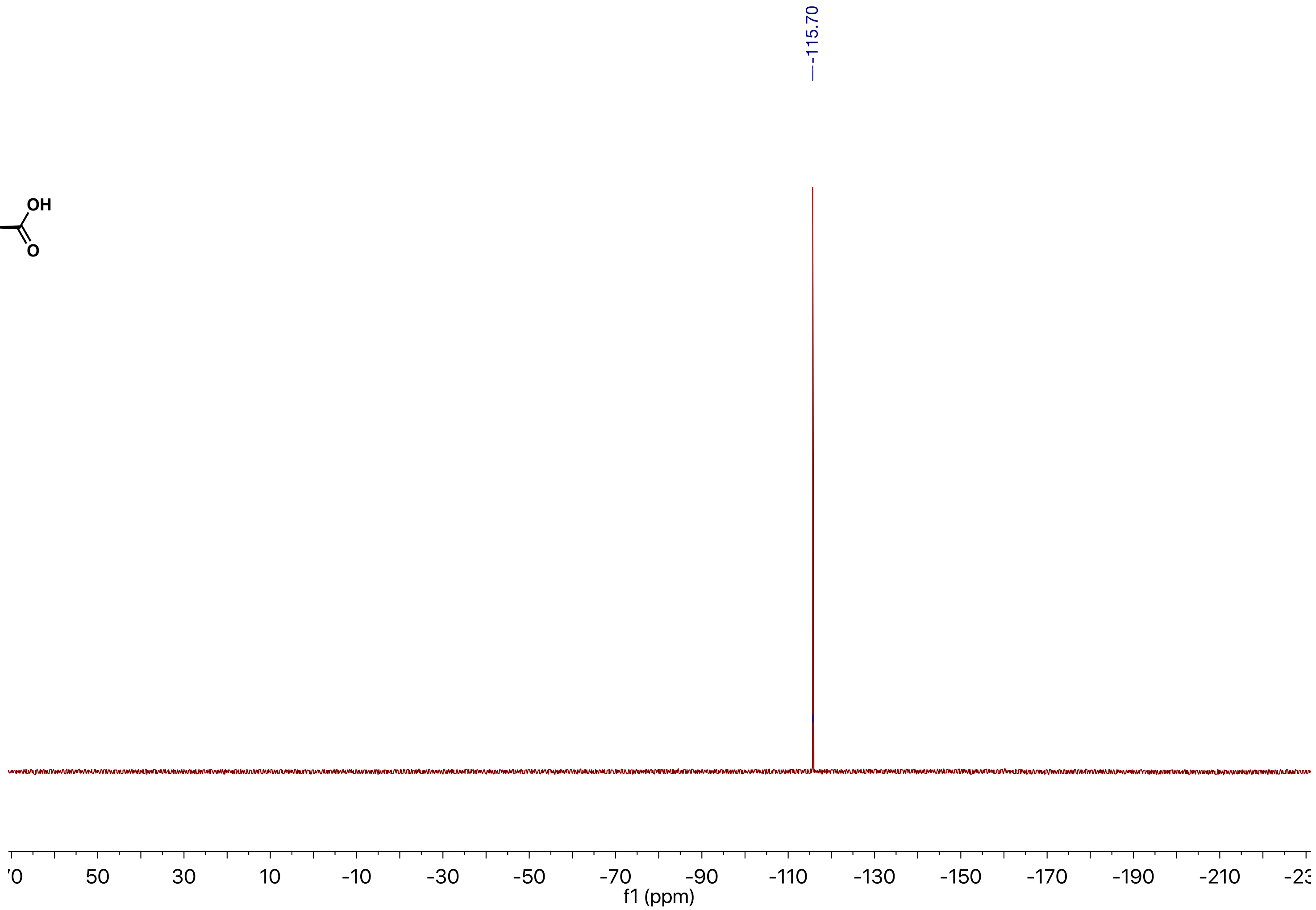

Compound A16 –^{13}C NMR

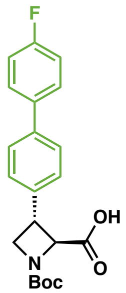

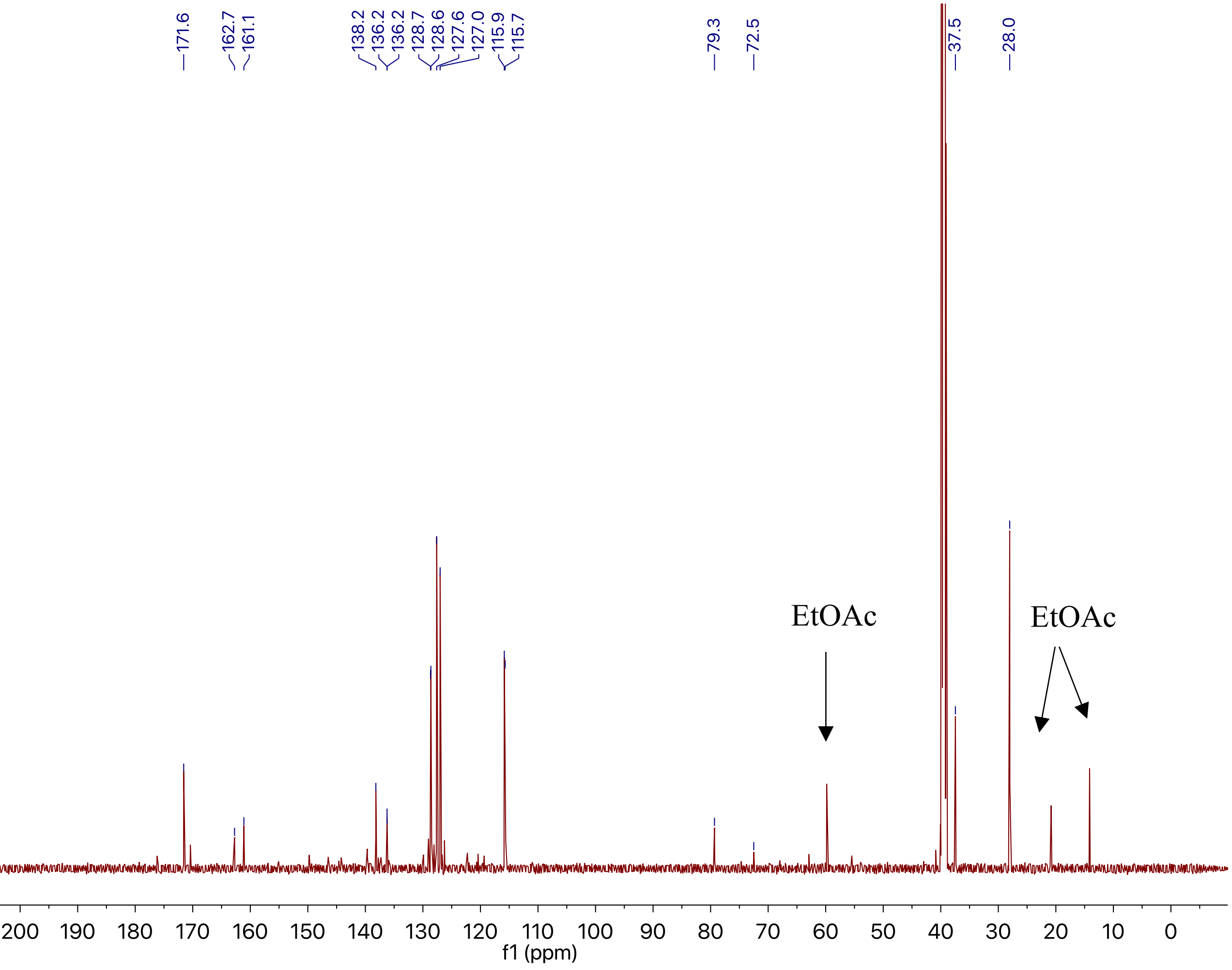

Compound B9 – ^{1}H NMR

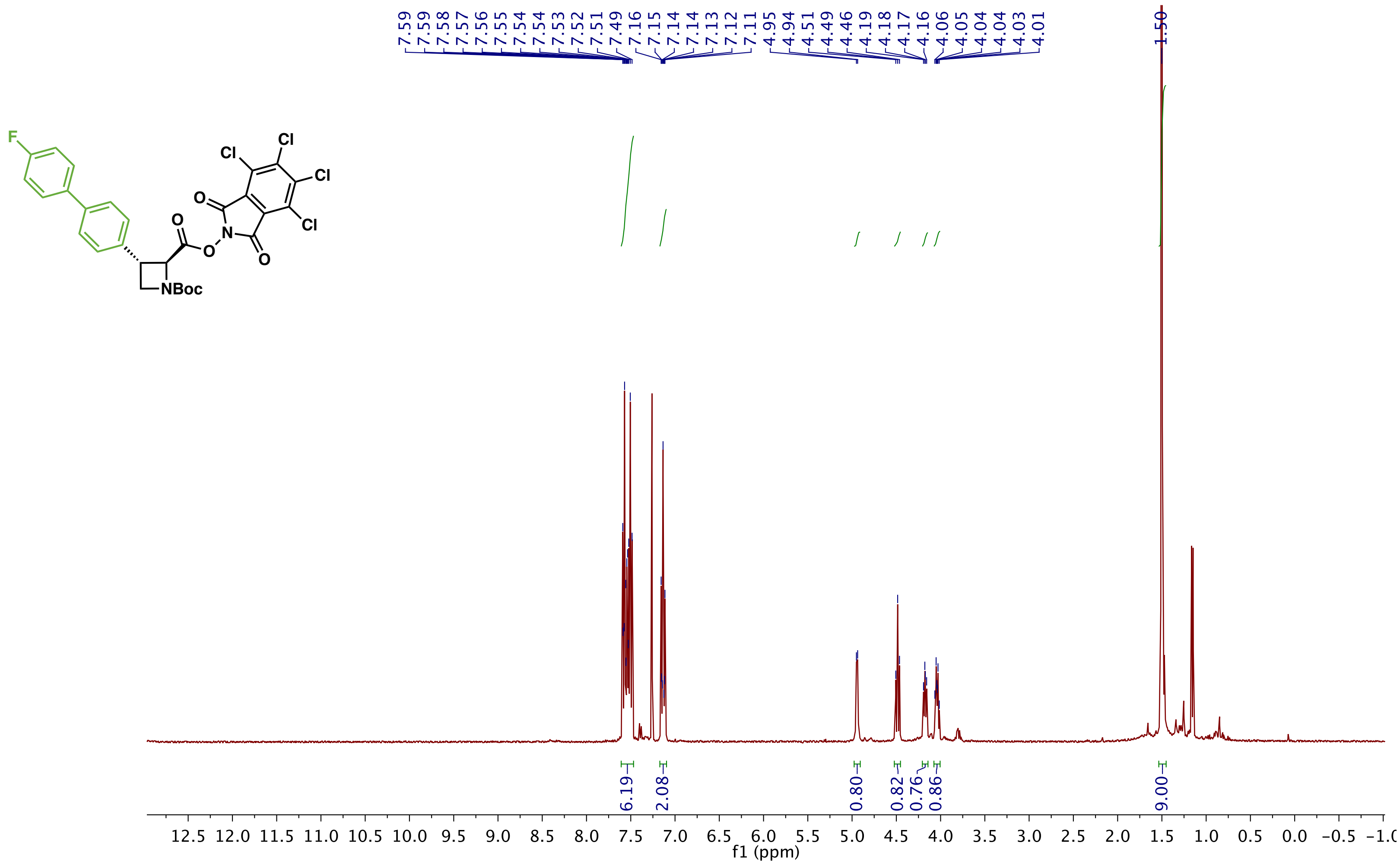

Compound B9 – ^{19}F NMR

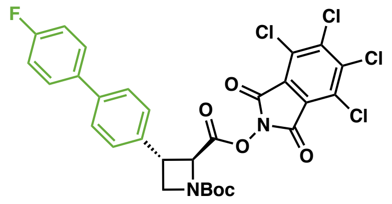

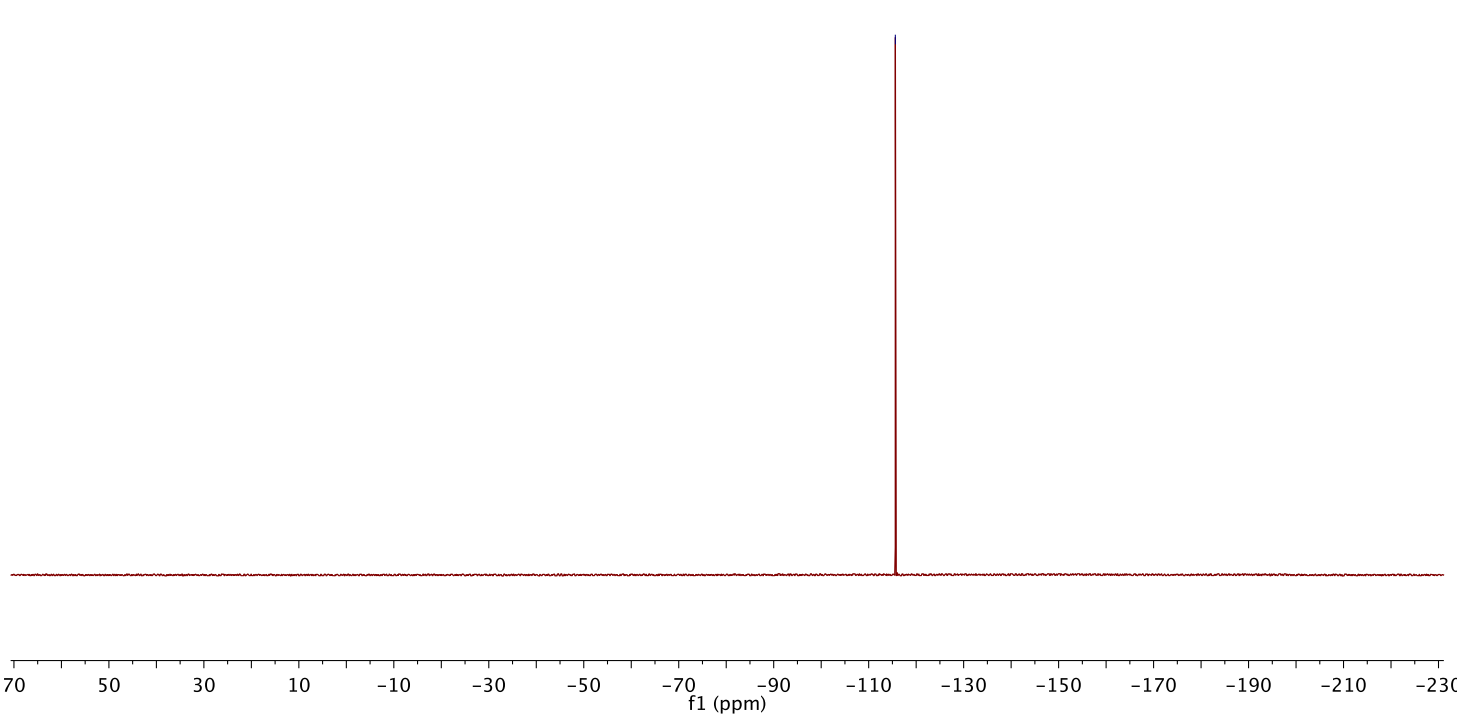

Compound B9 - ^{13}C NMR

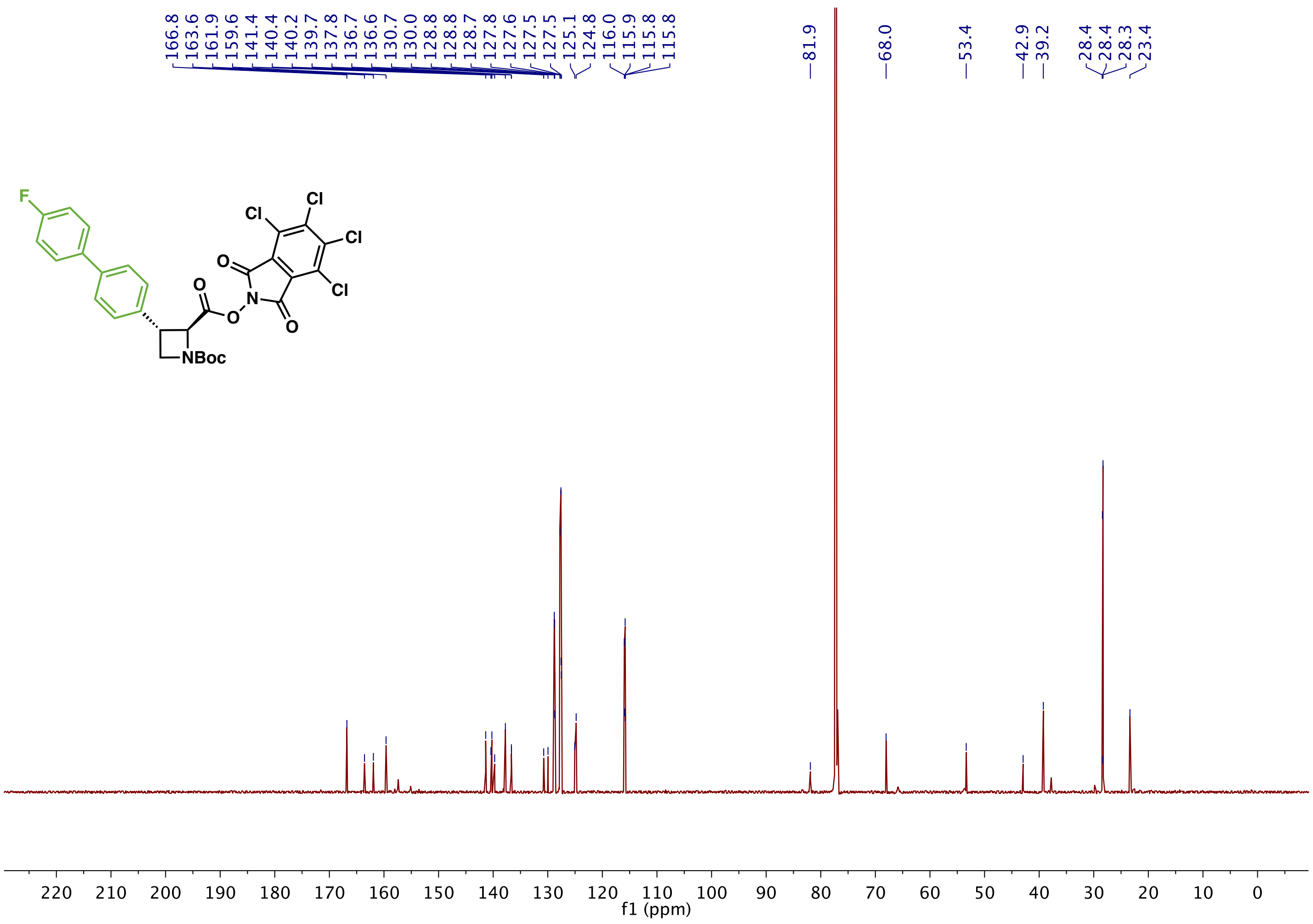

Compound SI-46 – ^{1}H NMR

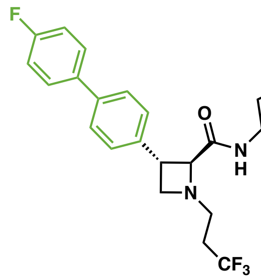

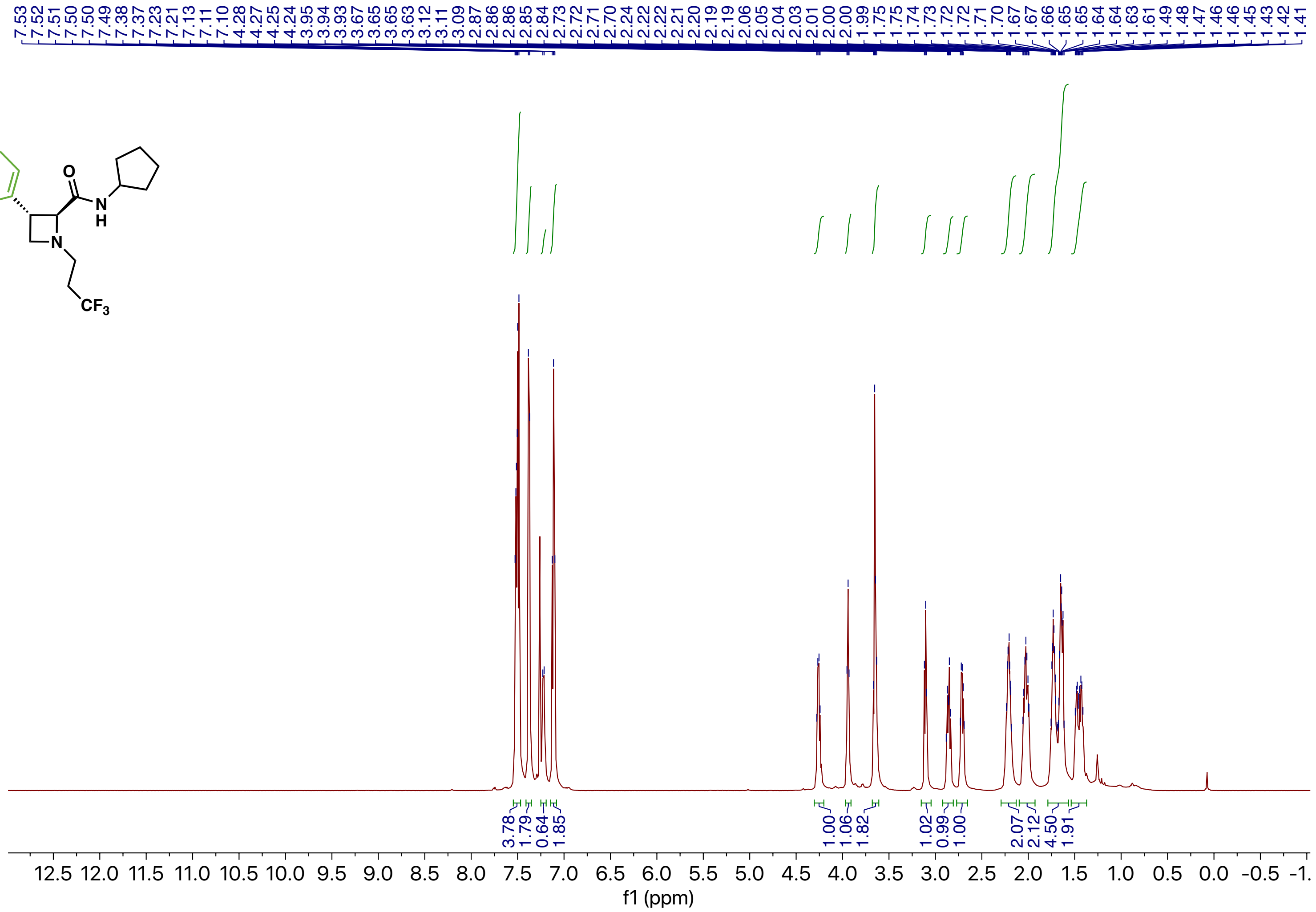

Compound SI-46 – ^{19}F NMR

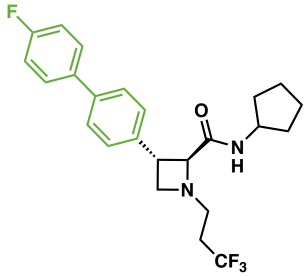

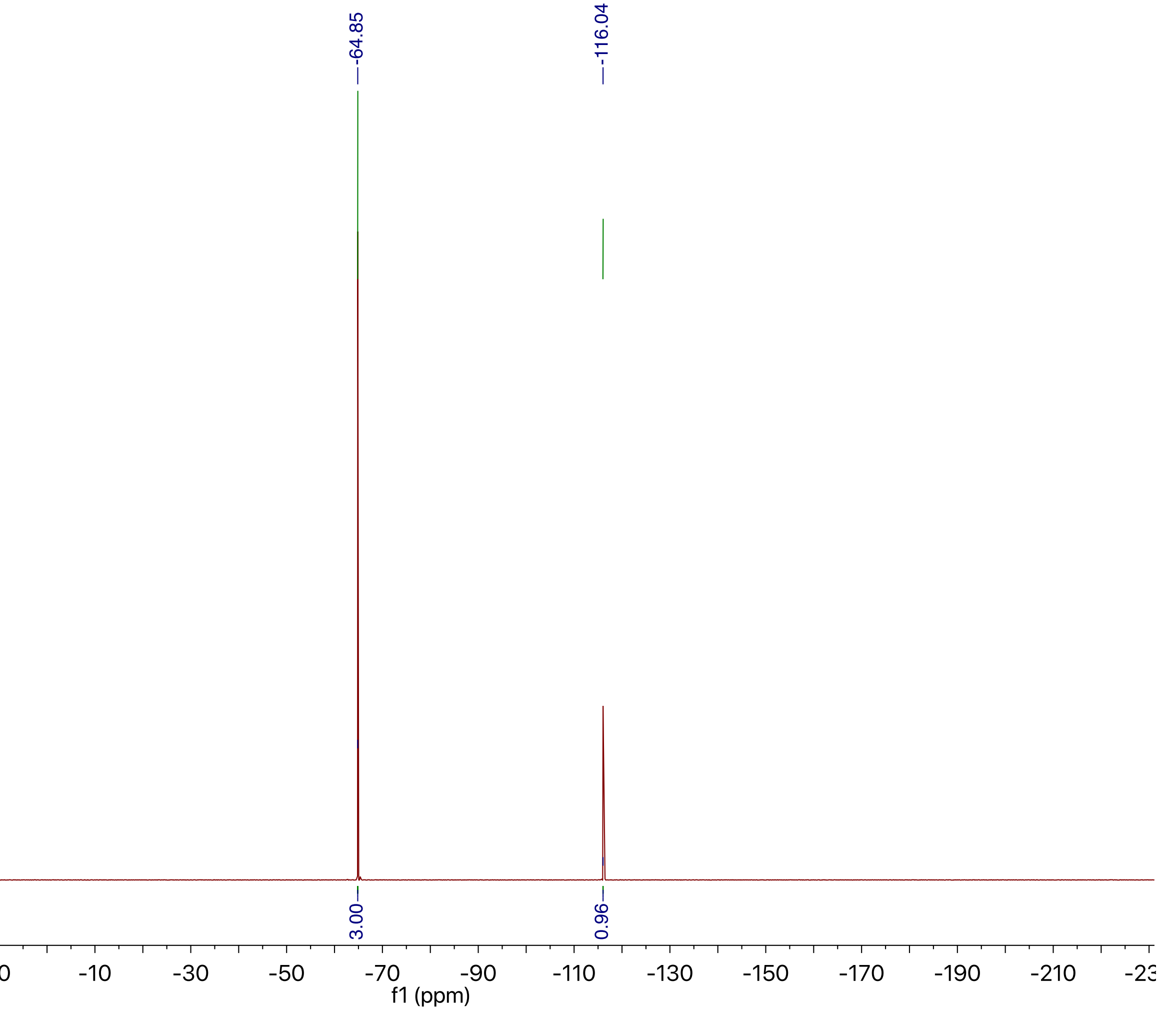

Compound SI-46 – ^{13}C NMR

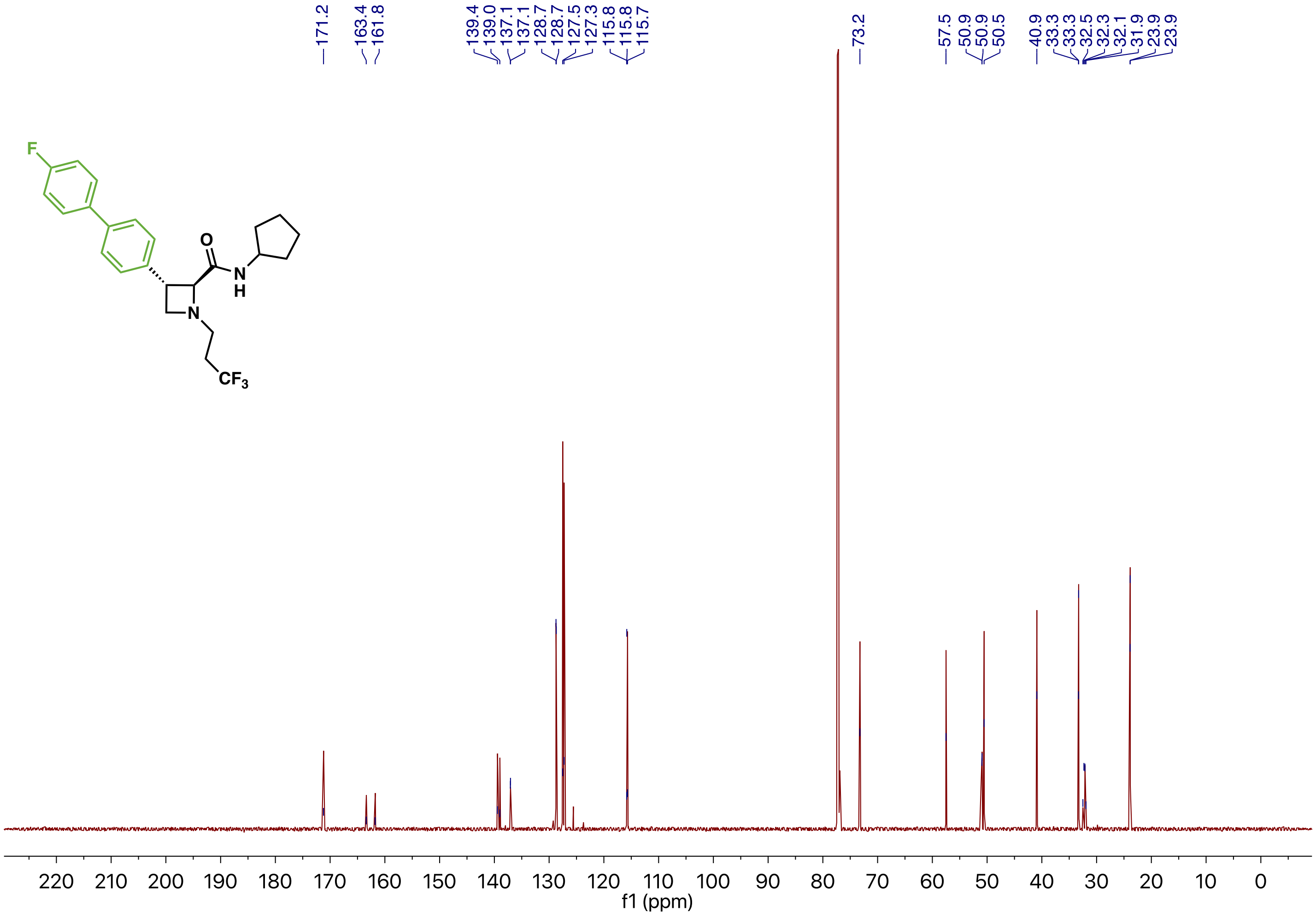

Compound 73 – ^{1}H NMR

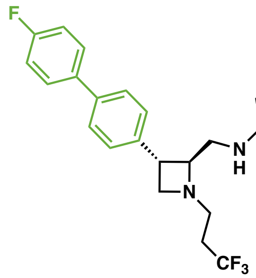

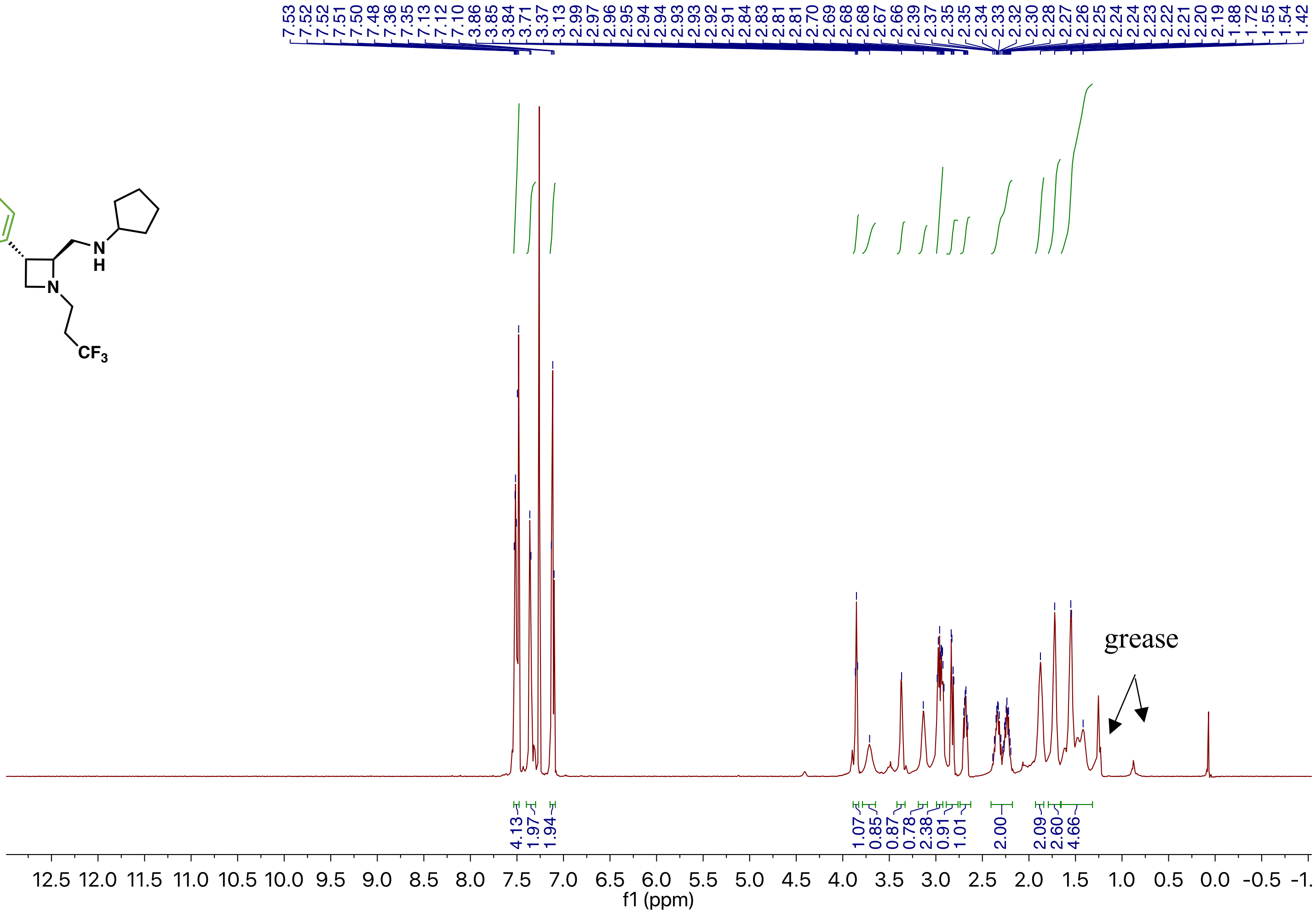

Compound 73 – ^{19}F NMR

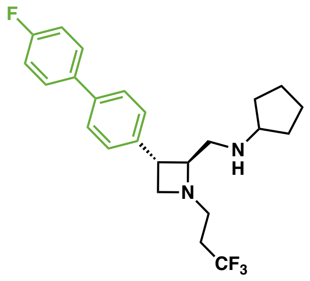

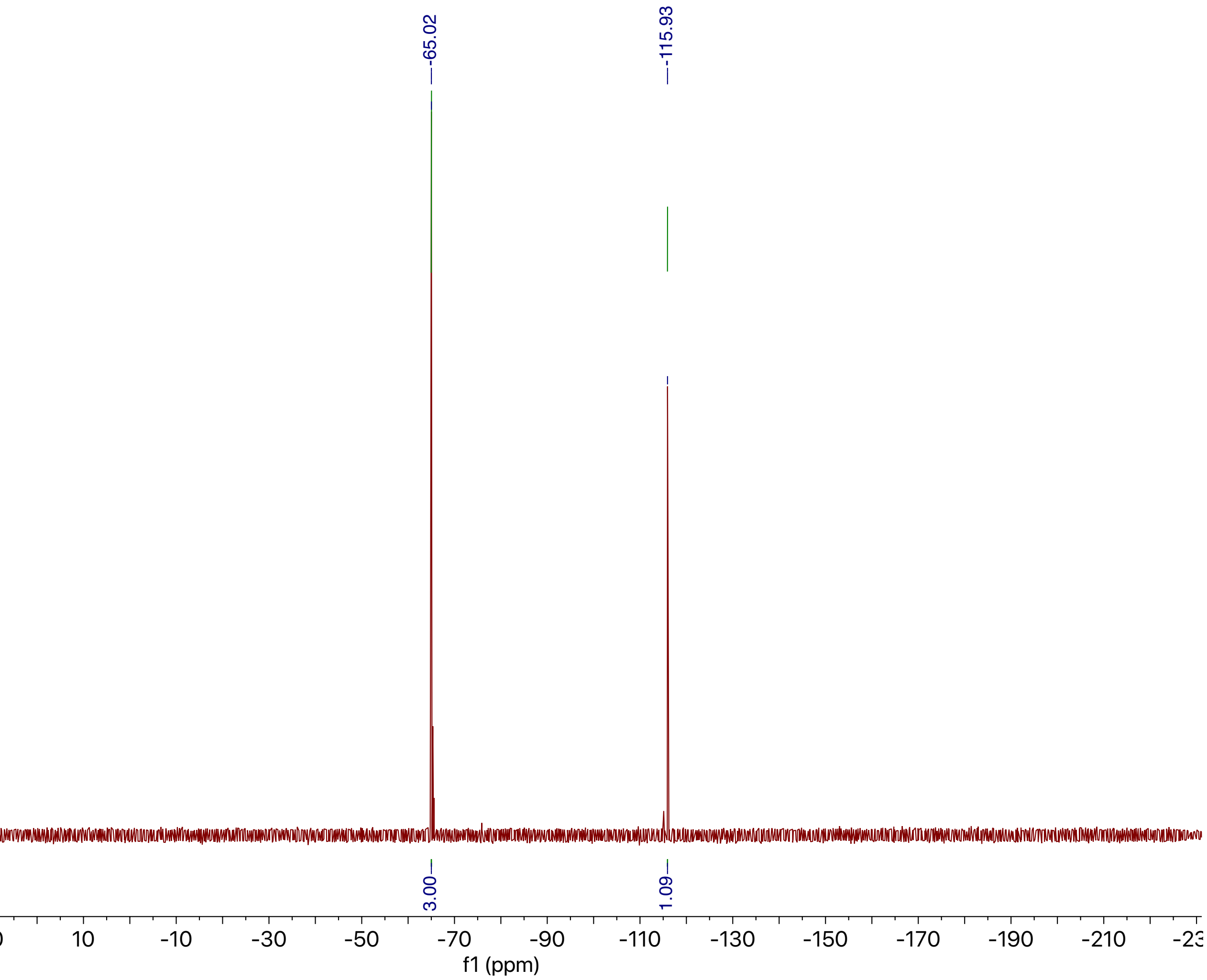

Compound 73 – ^{13}C NMR

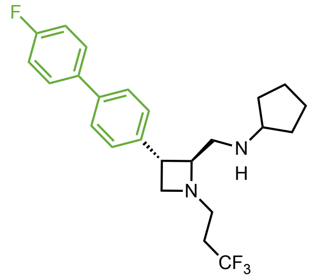

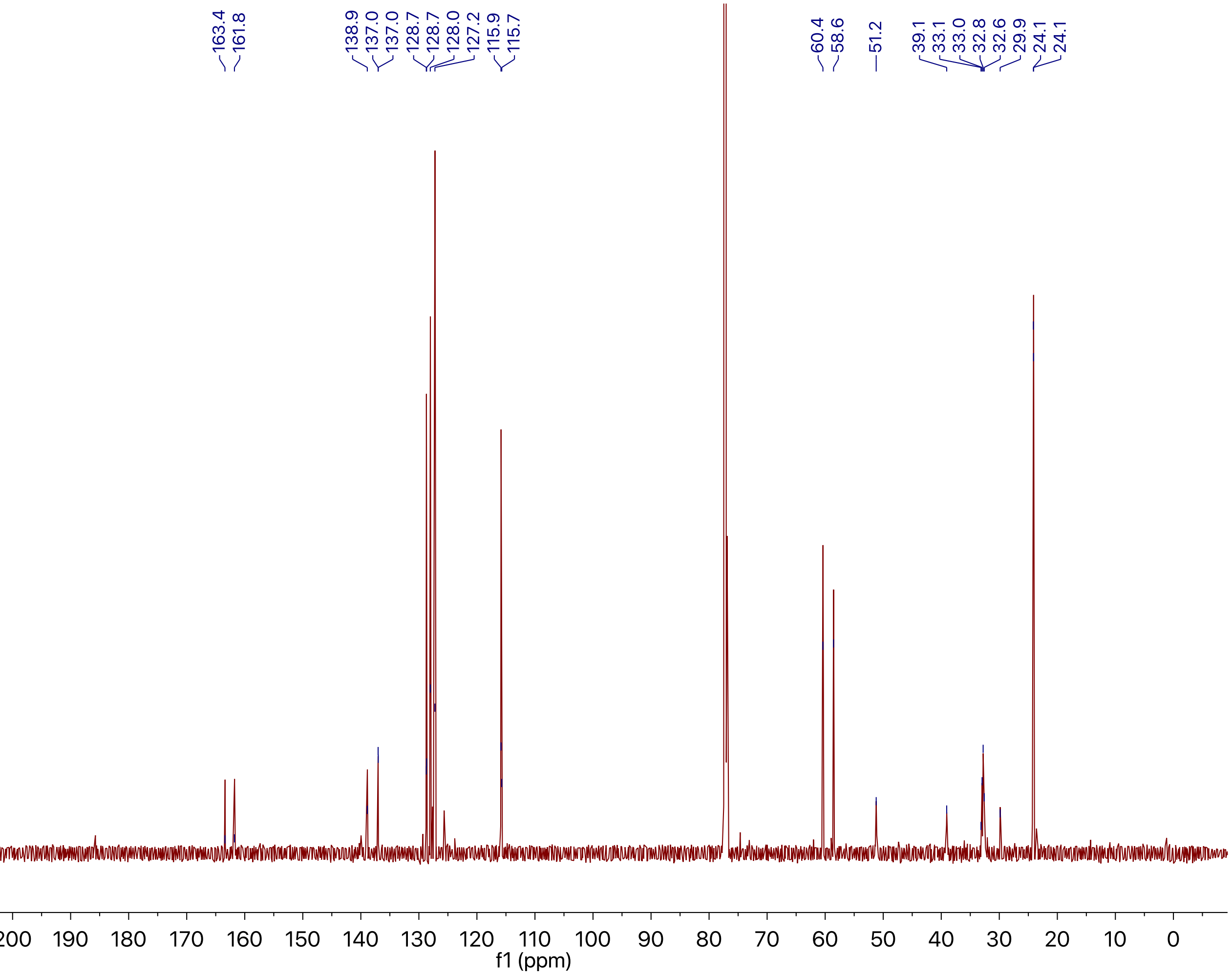

Compound 75 – ^{1}H NMR

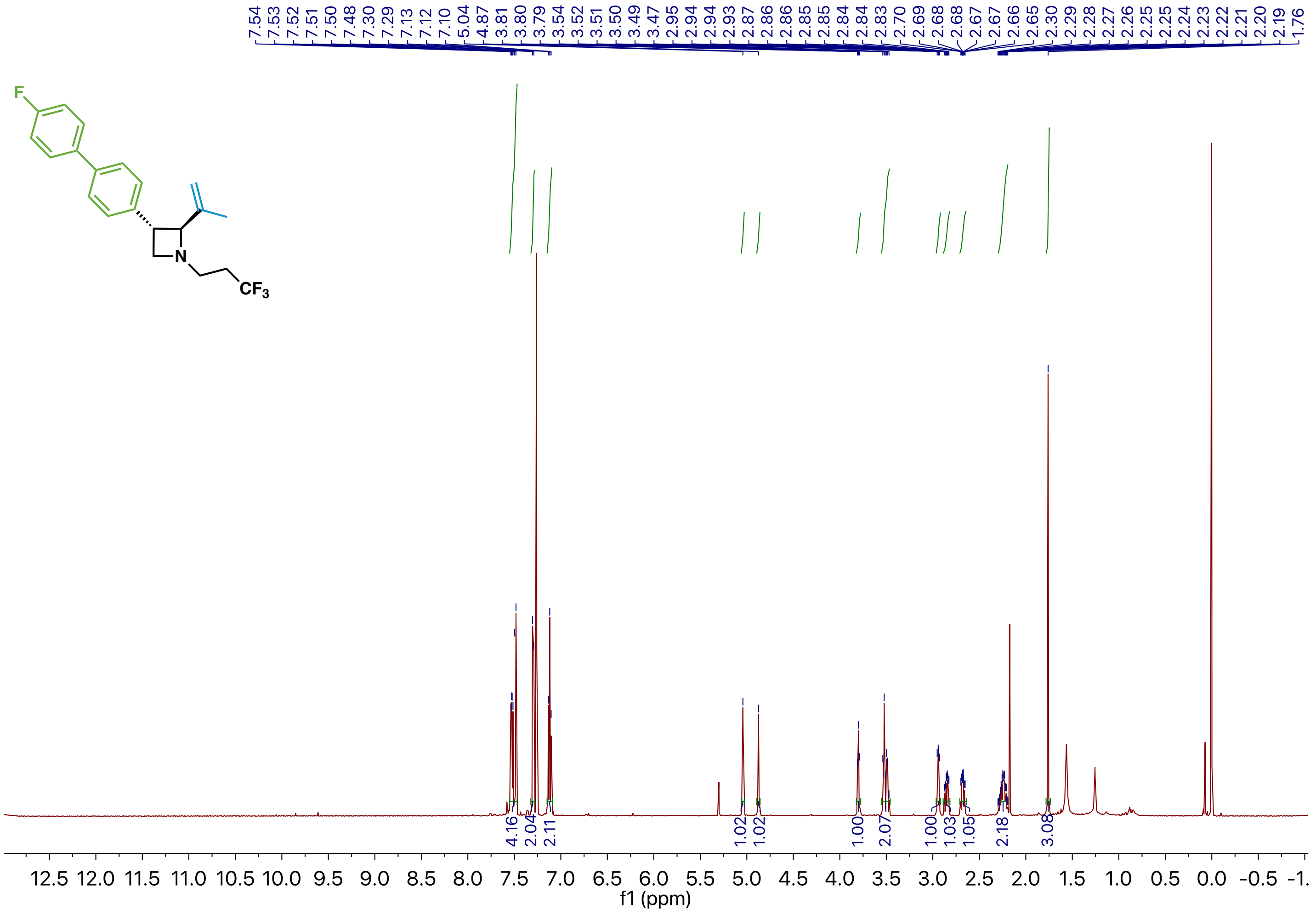

Compound 75 – ^{19}F NMR

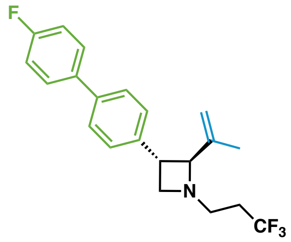

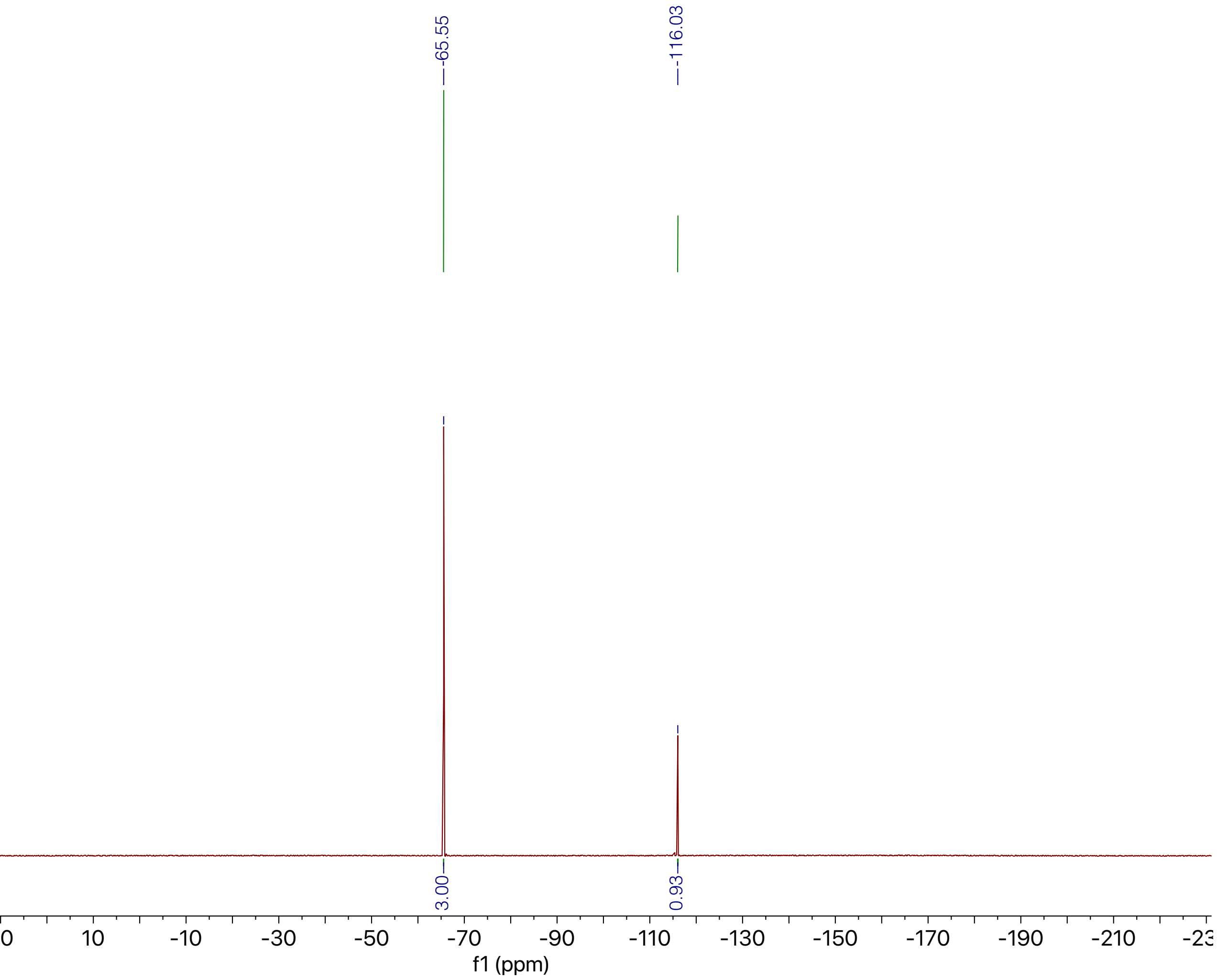

Compound 75 – ^{13}C NMR

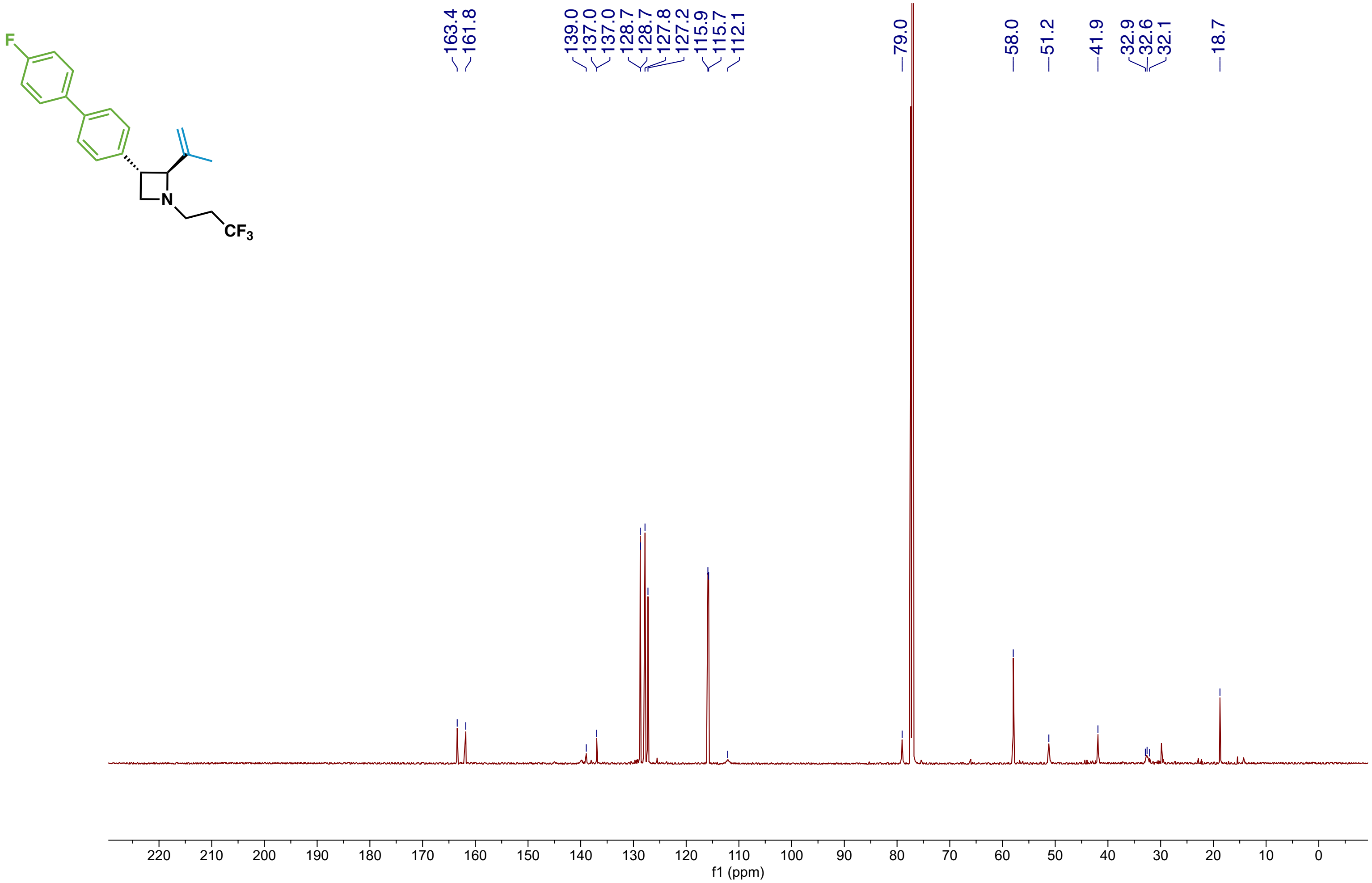

Compound SI-47 – ^{1}H NMR

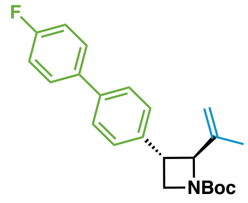

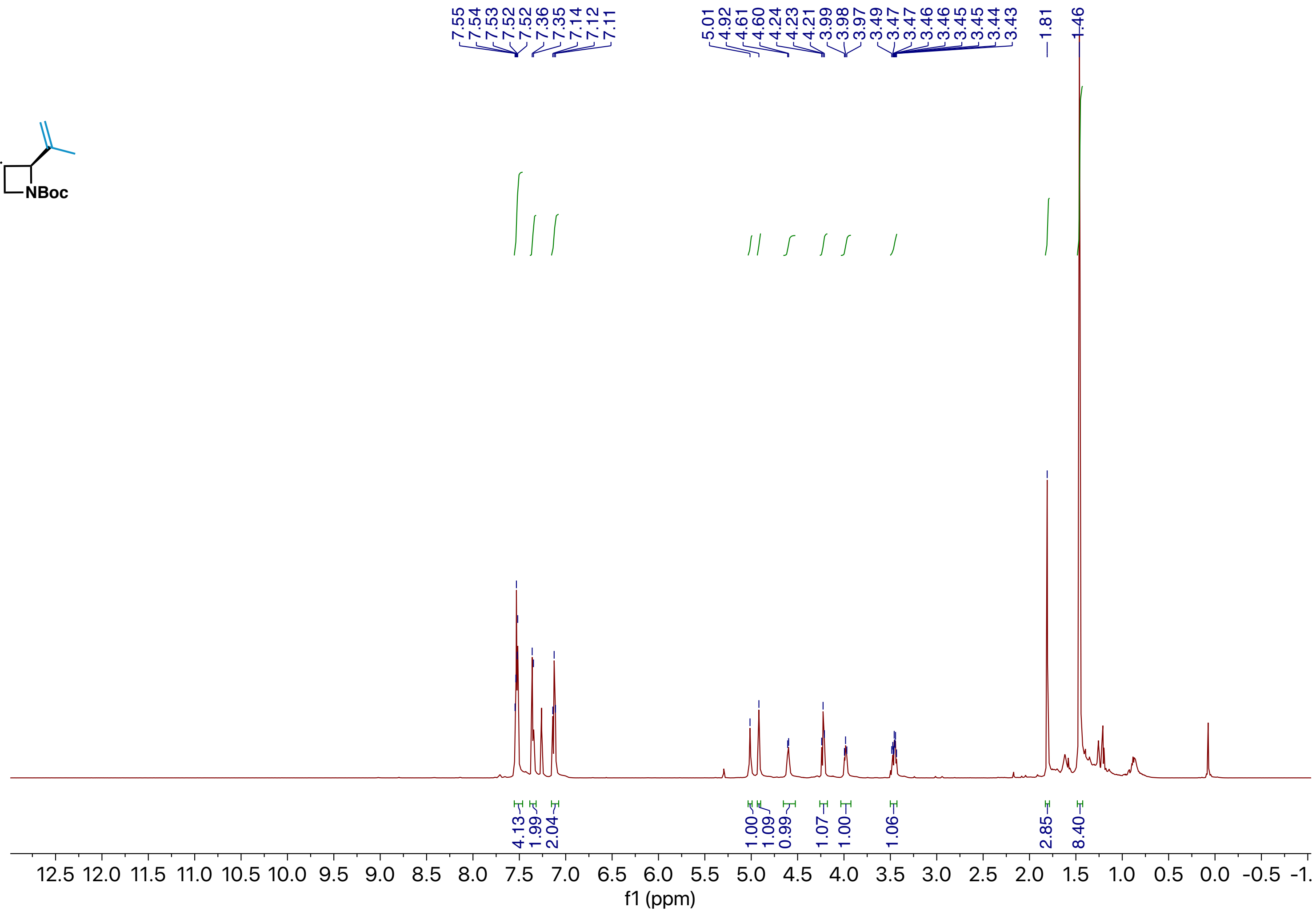

Compound SI-47 – ^{19}F NMR

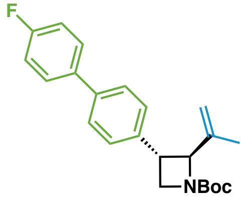

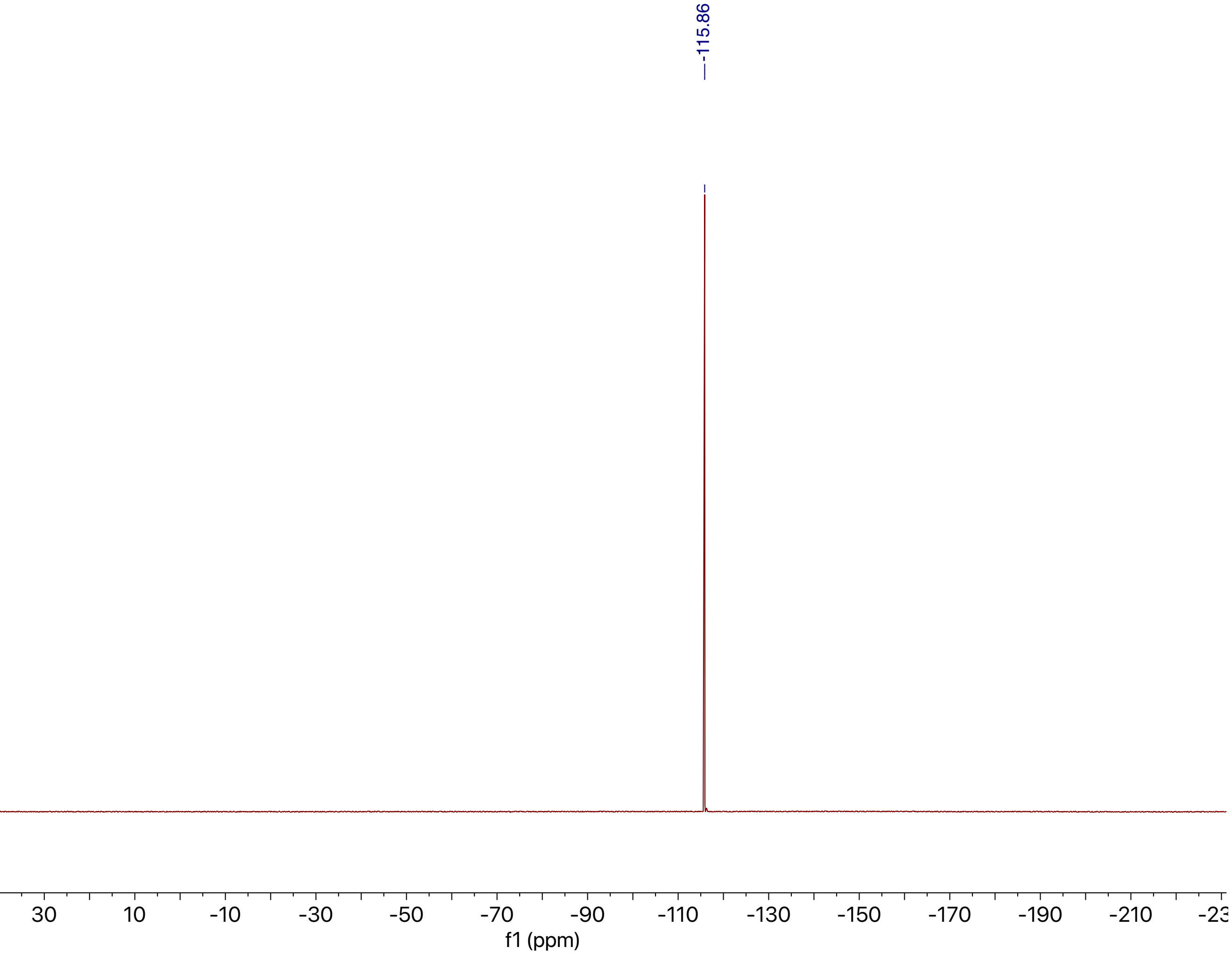

Compound SI-47 – ^{13}C NMR

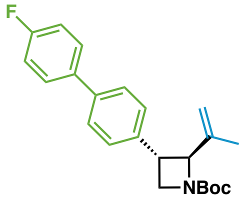

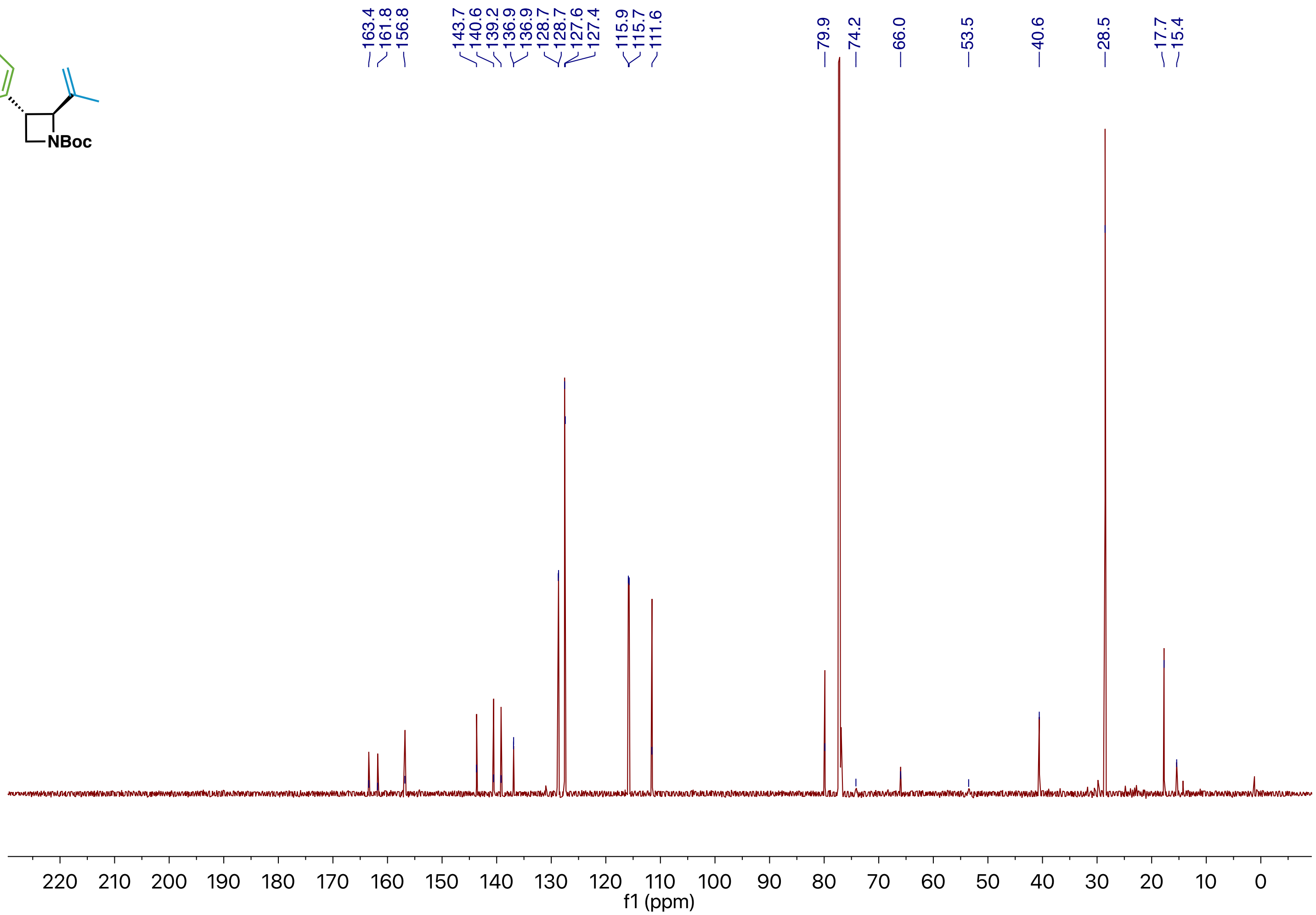

Compound 76 – ^{1}H NMR

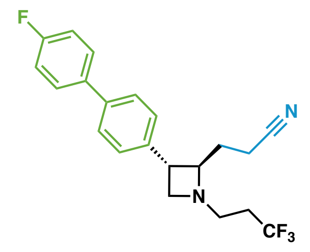

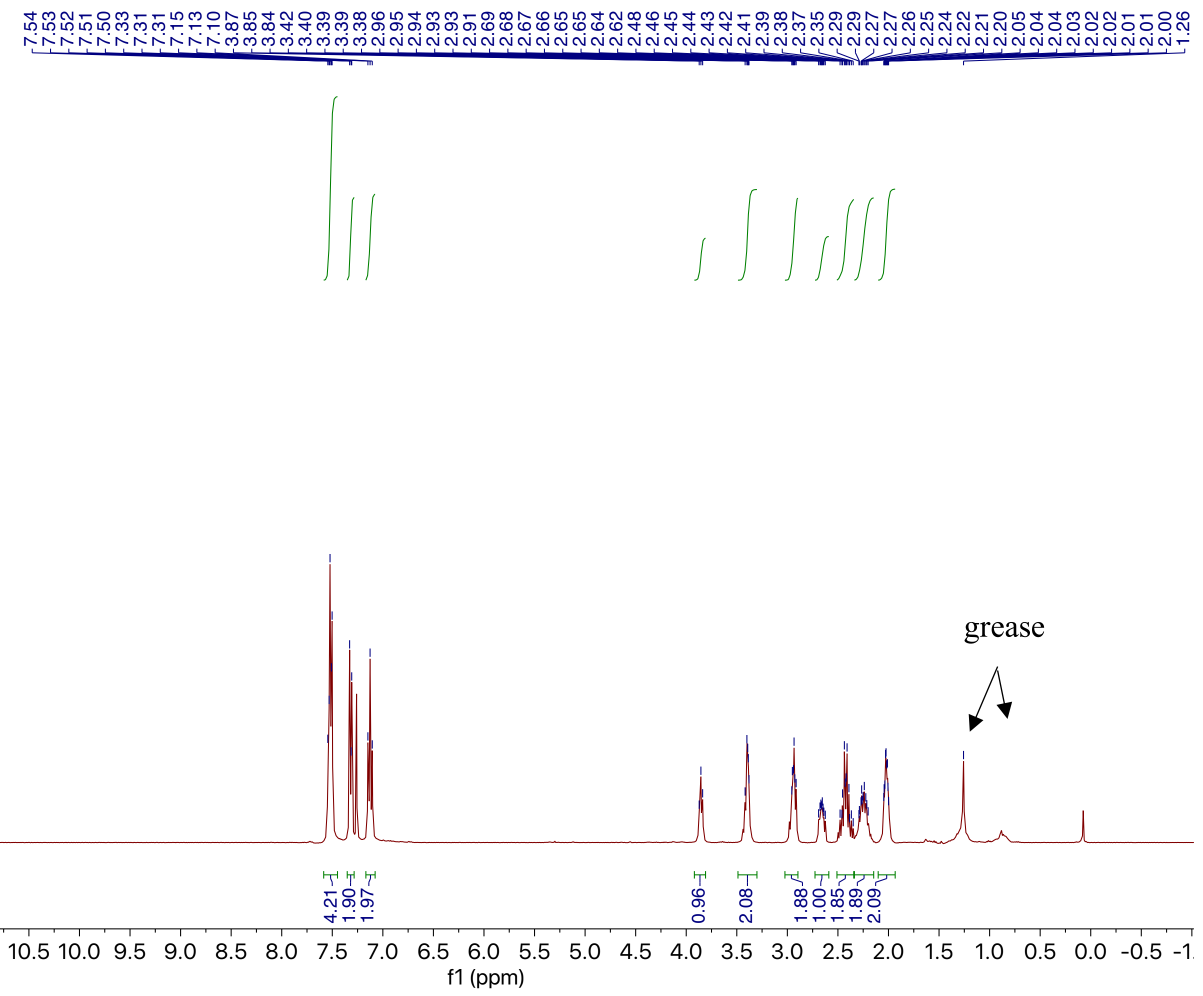

Compound 76 – ^{19}F NMR

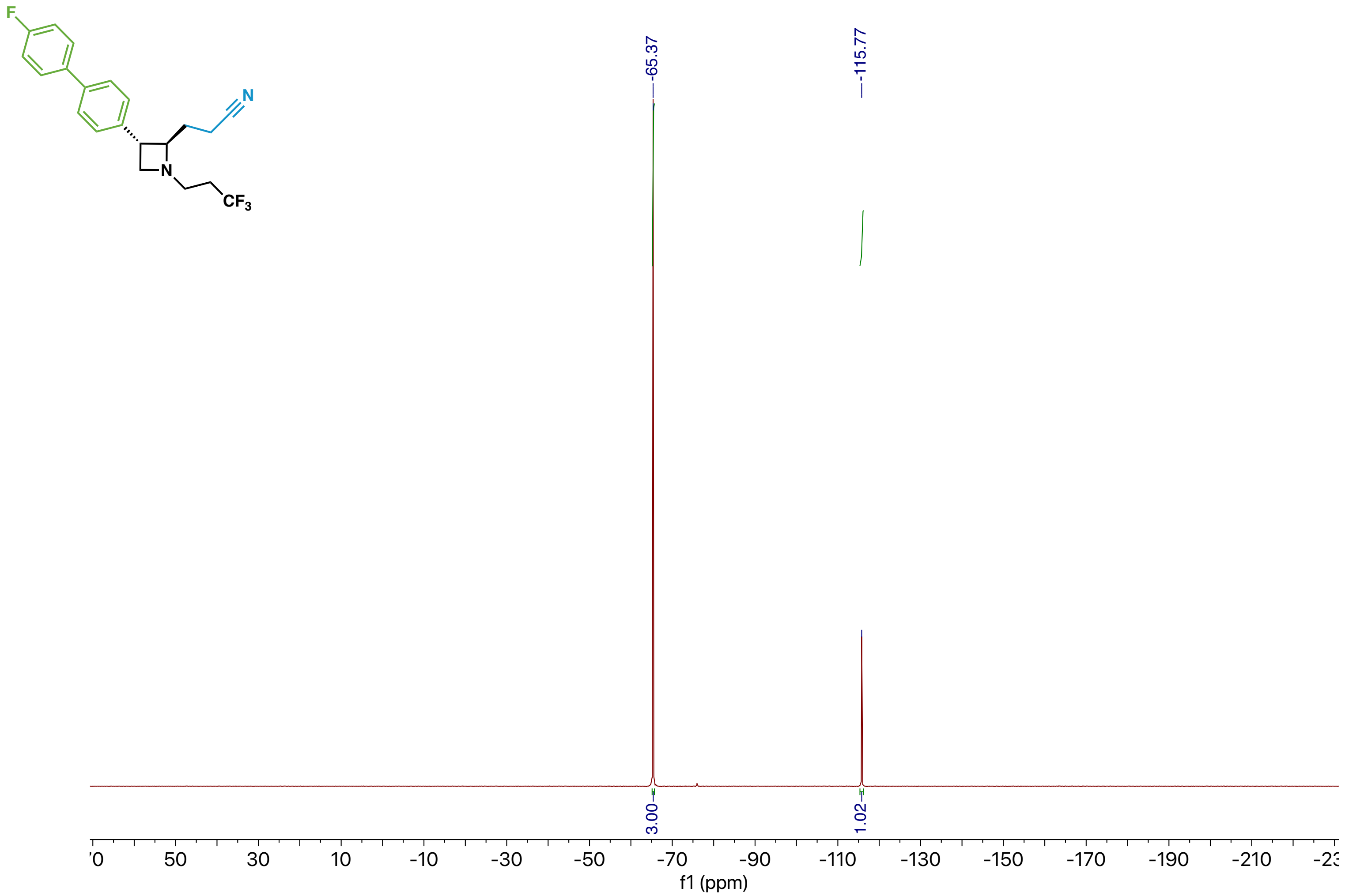

Compound 76 – ^{13}C NMR

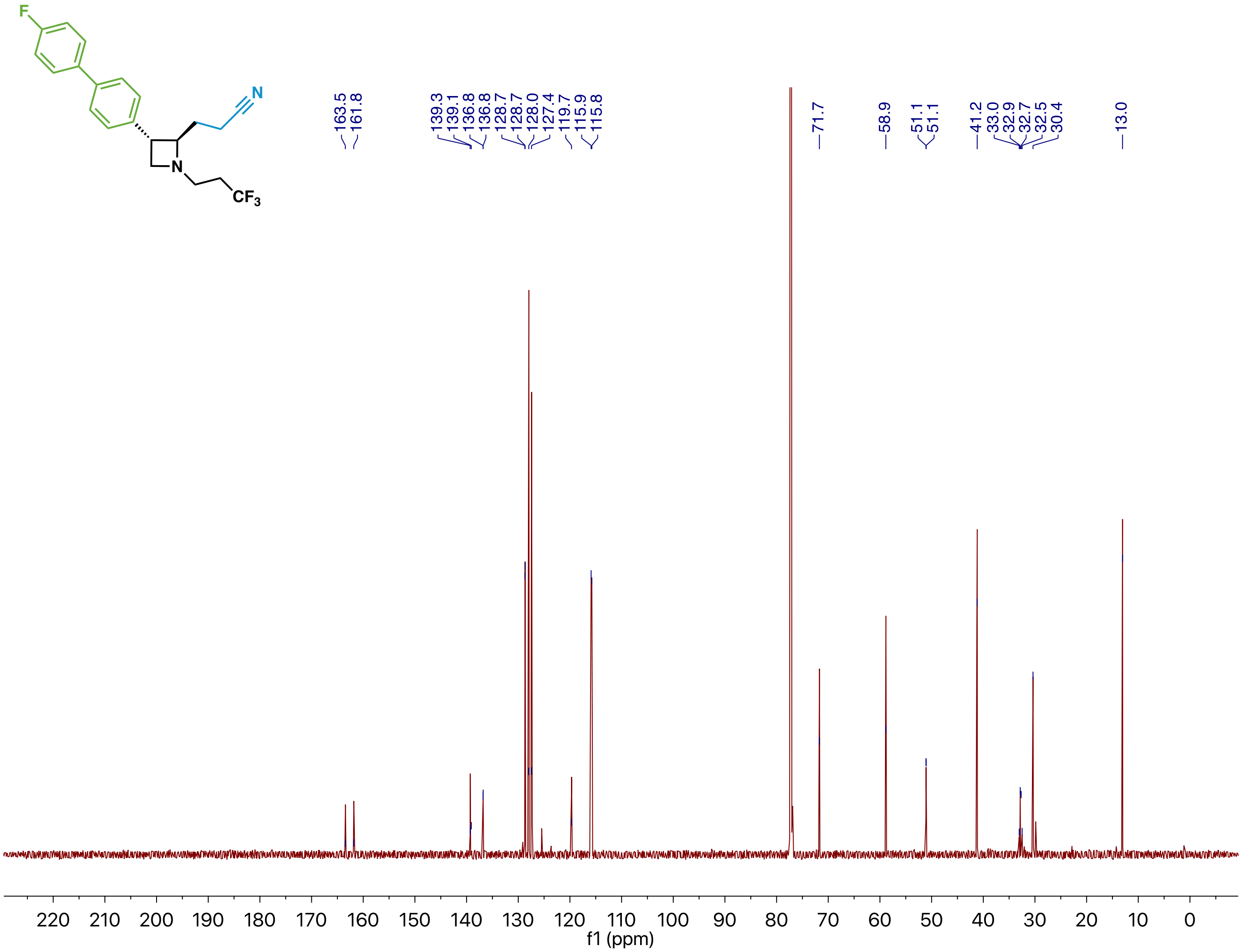

Compound SI-48 – ^{1}H NMR

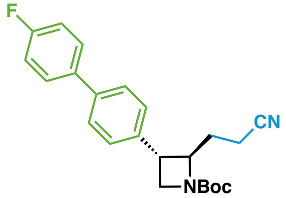

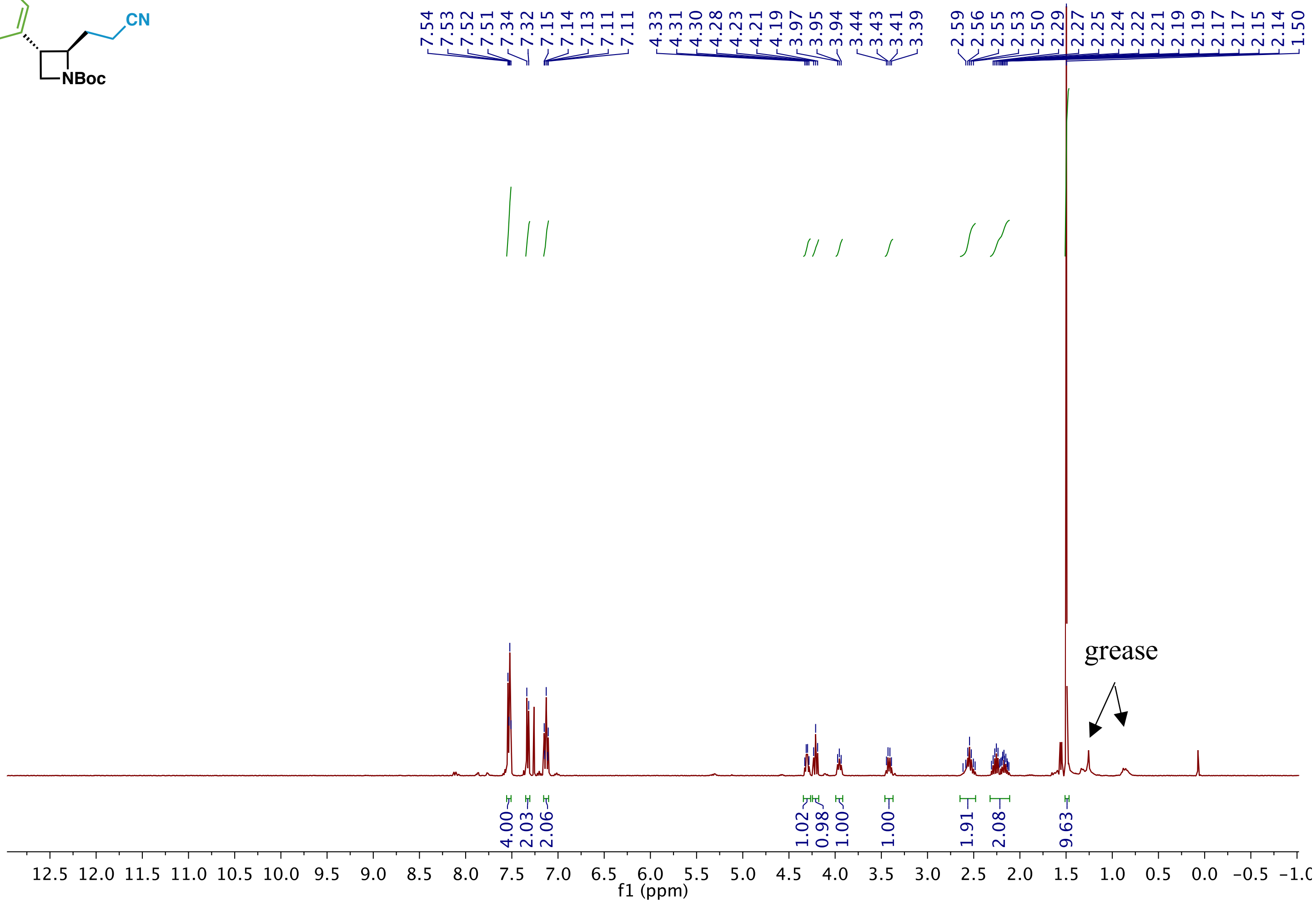

Compound SI-48 – ^{19}F NMR

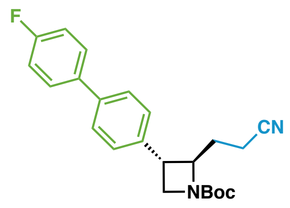

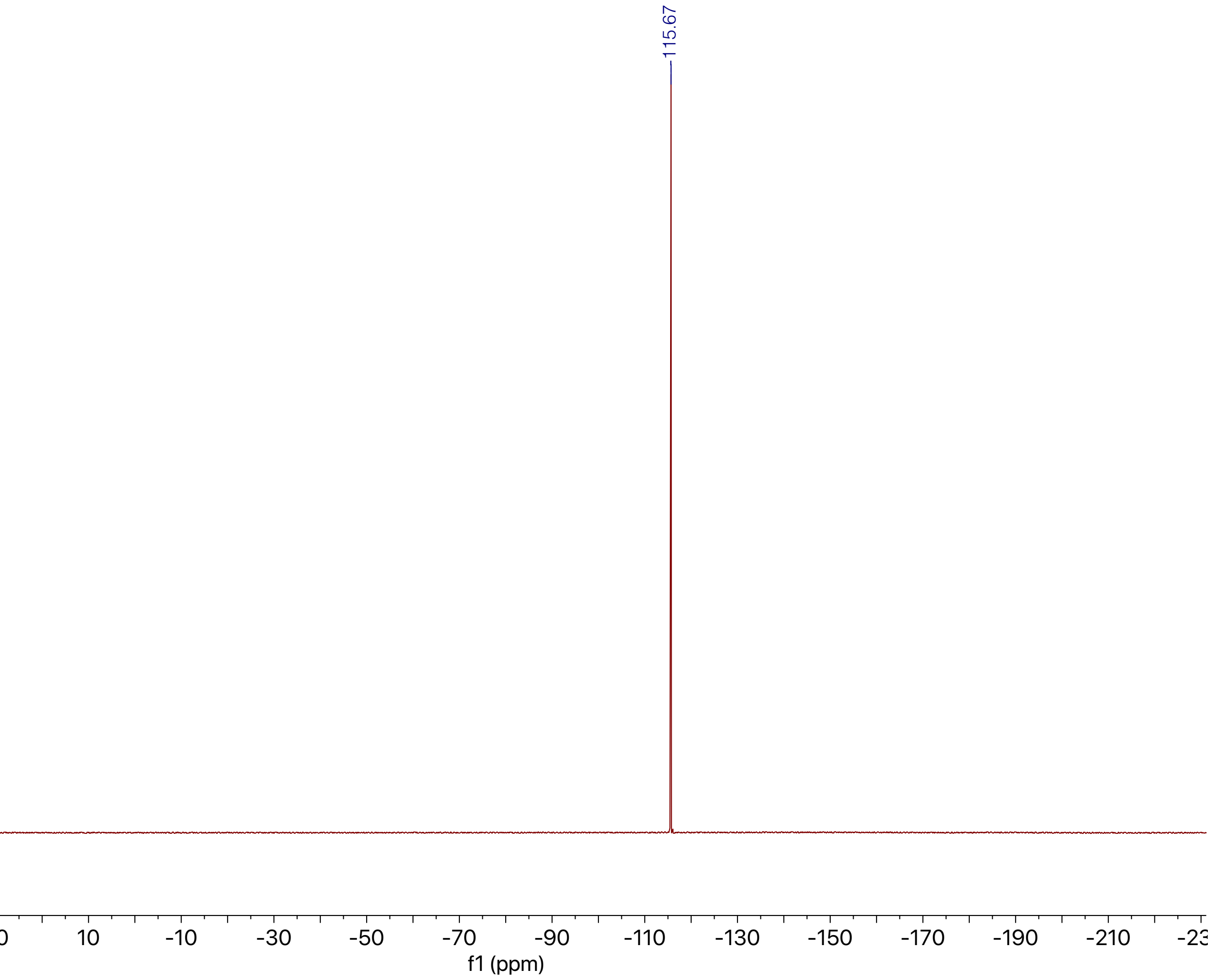

Compound SI-48 – ^{13}C NMR

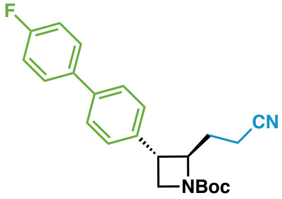

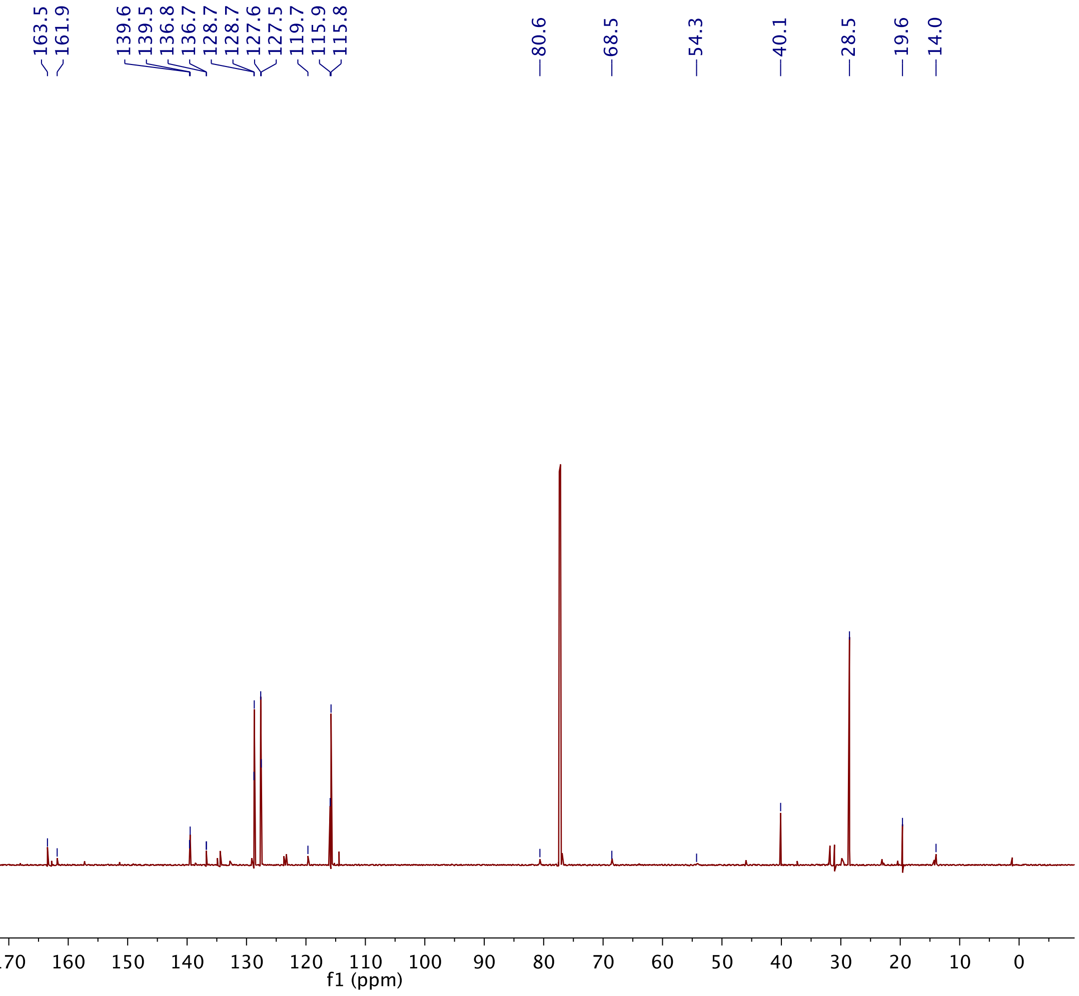

Compound 77 – ^{1}H NMR

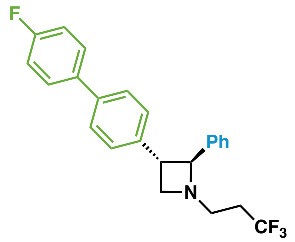

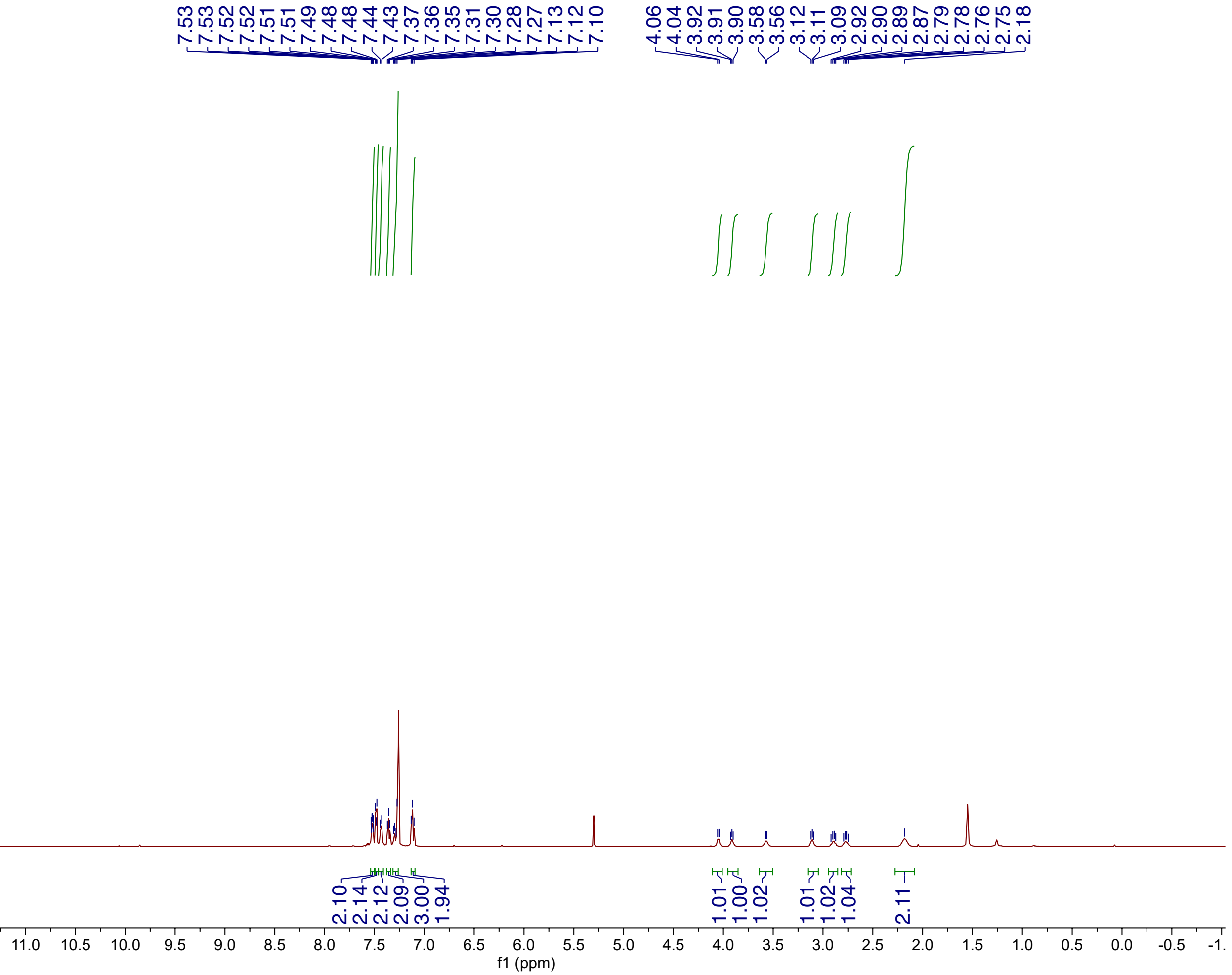

Compound 77 – ^{19}F NMR

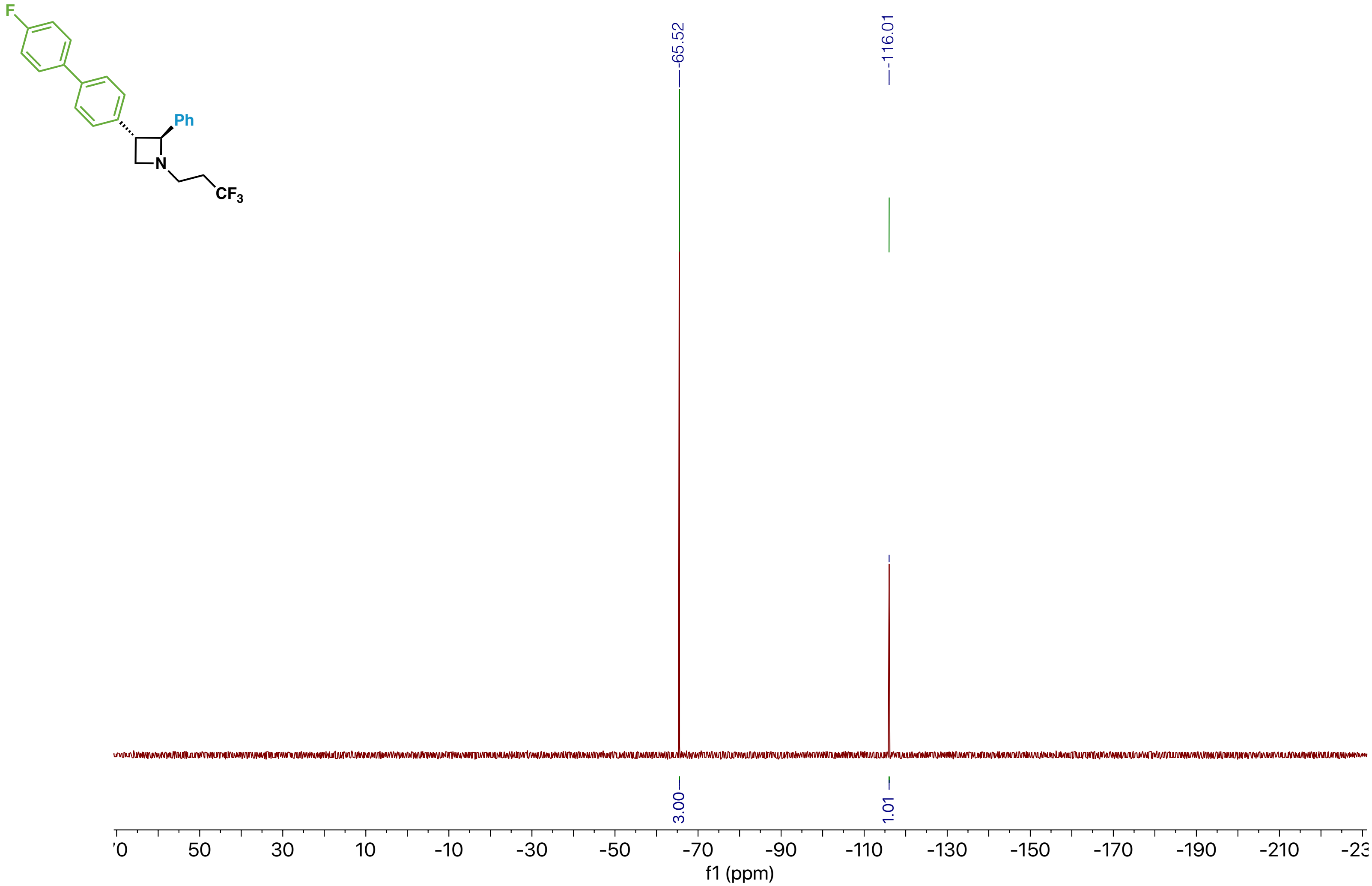

Compound 77 – ^{13}C NMR

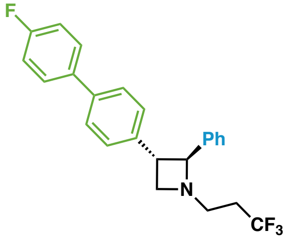

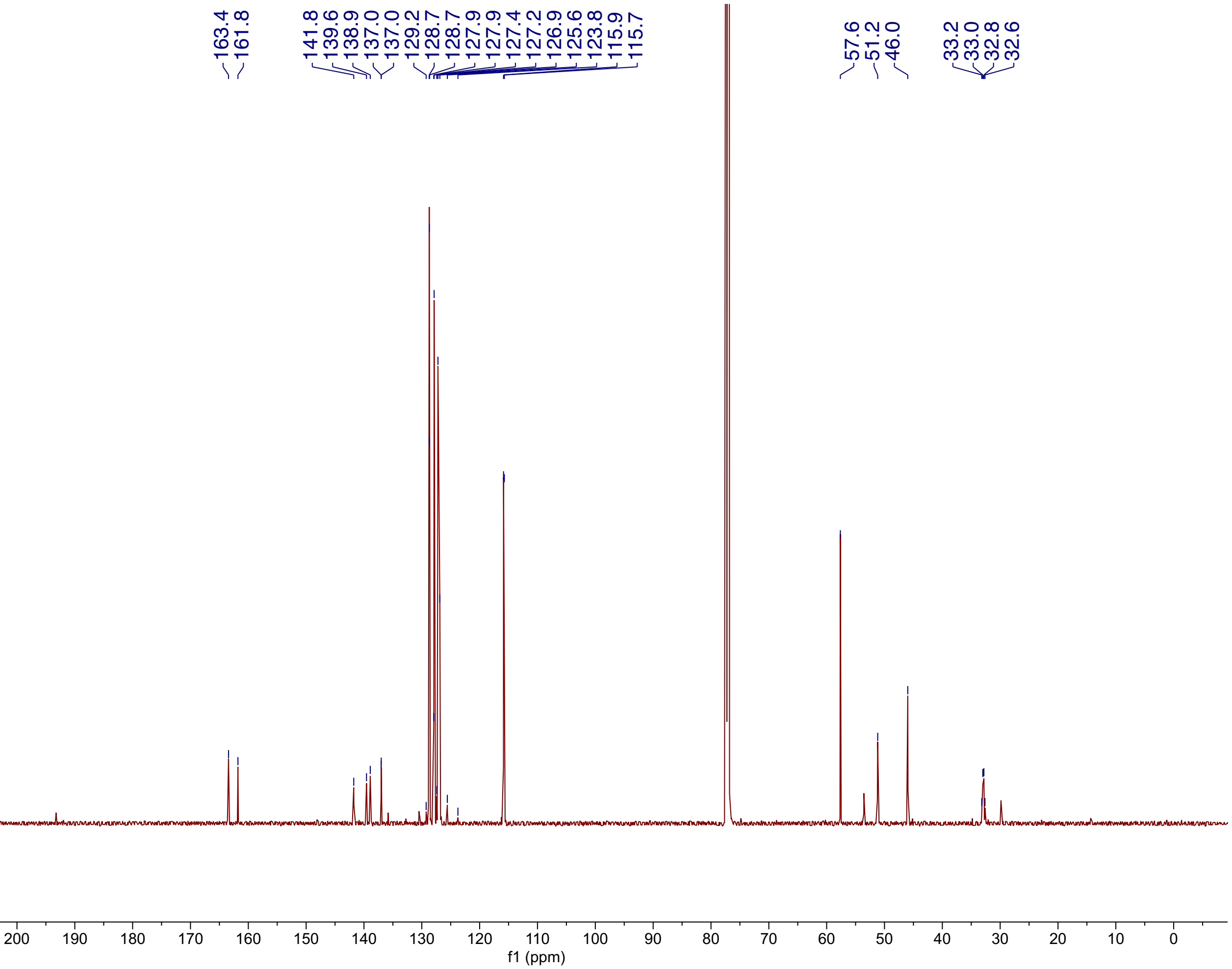

Compound SI-49 – ^{1}H NMR

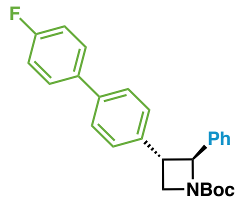

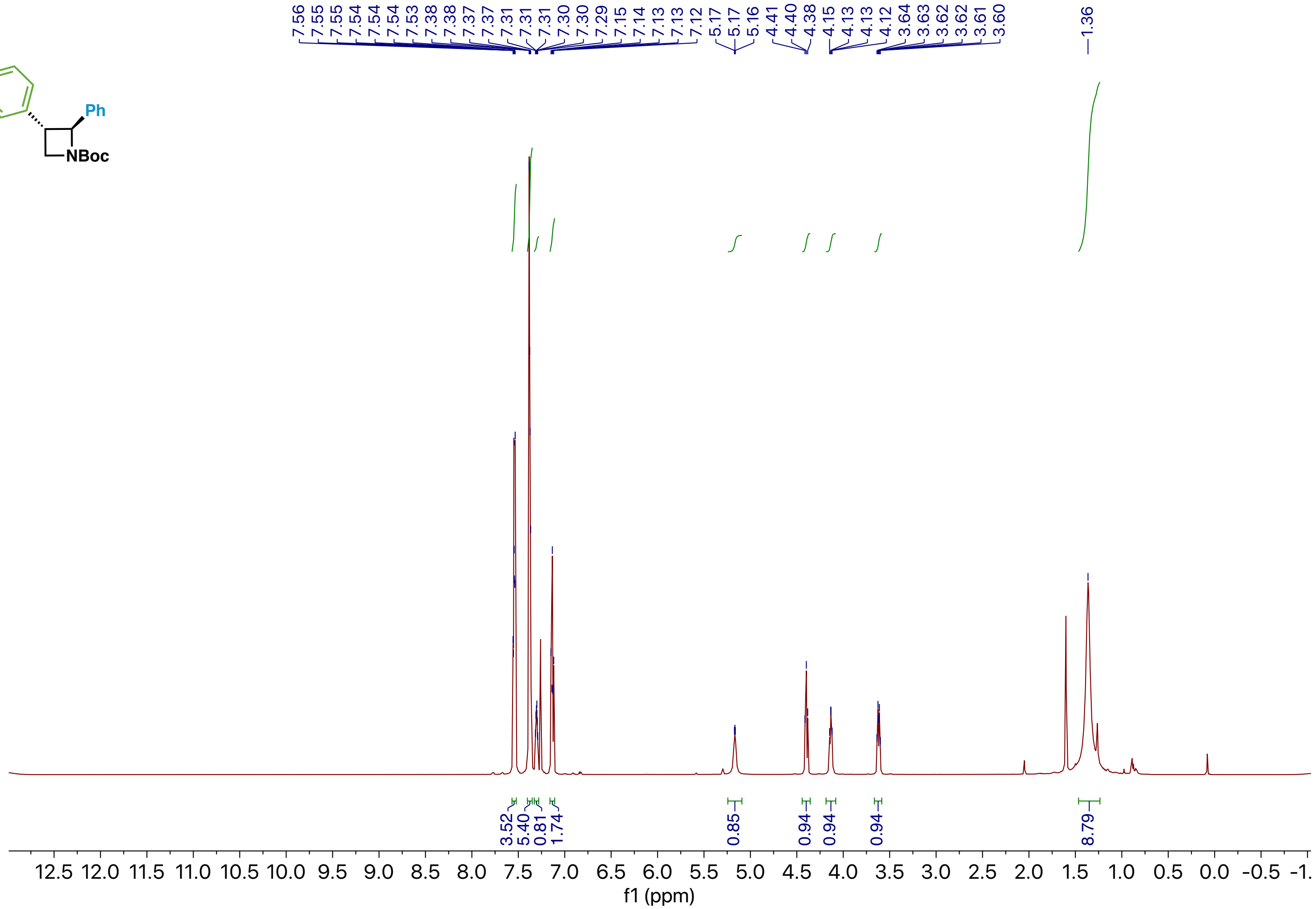

Compound SI-49 – ^{19}F NMR

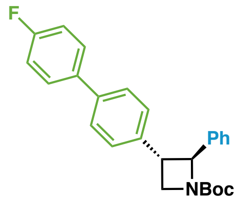

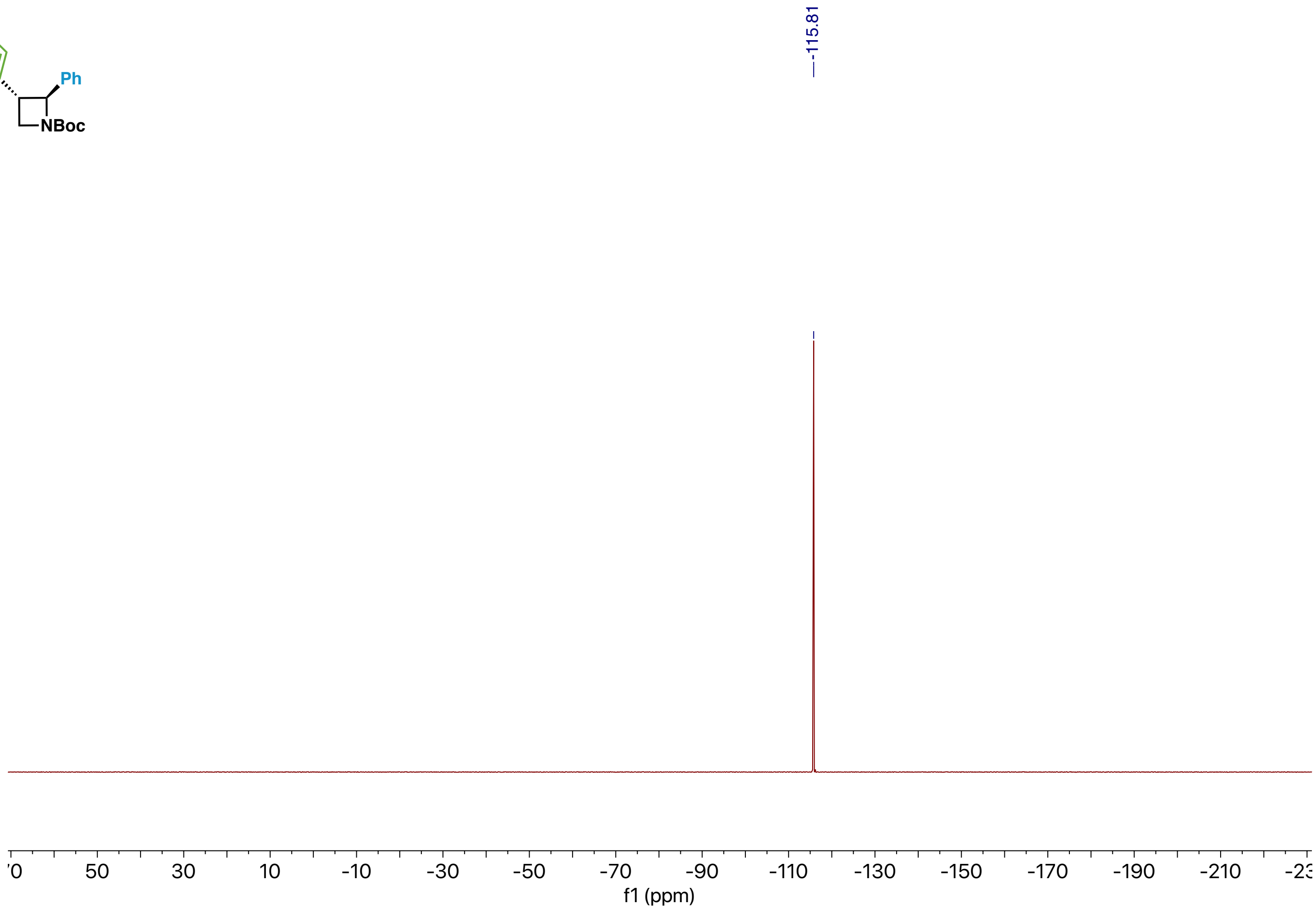

Compound SI-49 – ^{13}C NMR

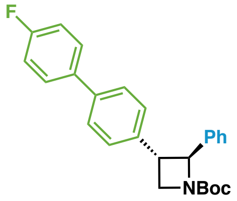

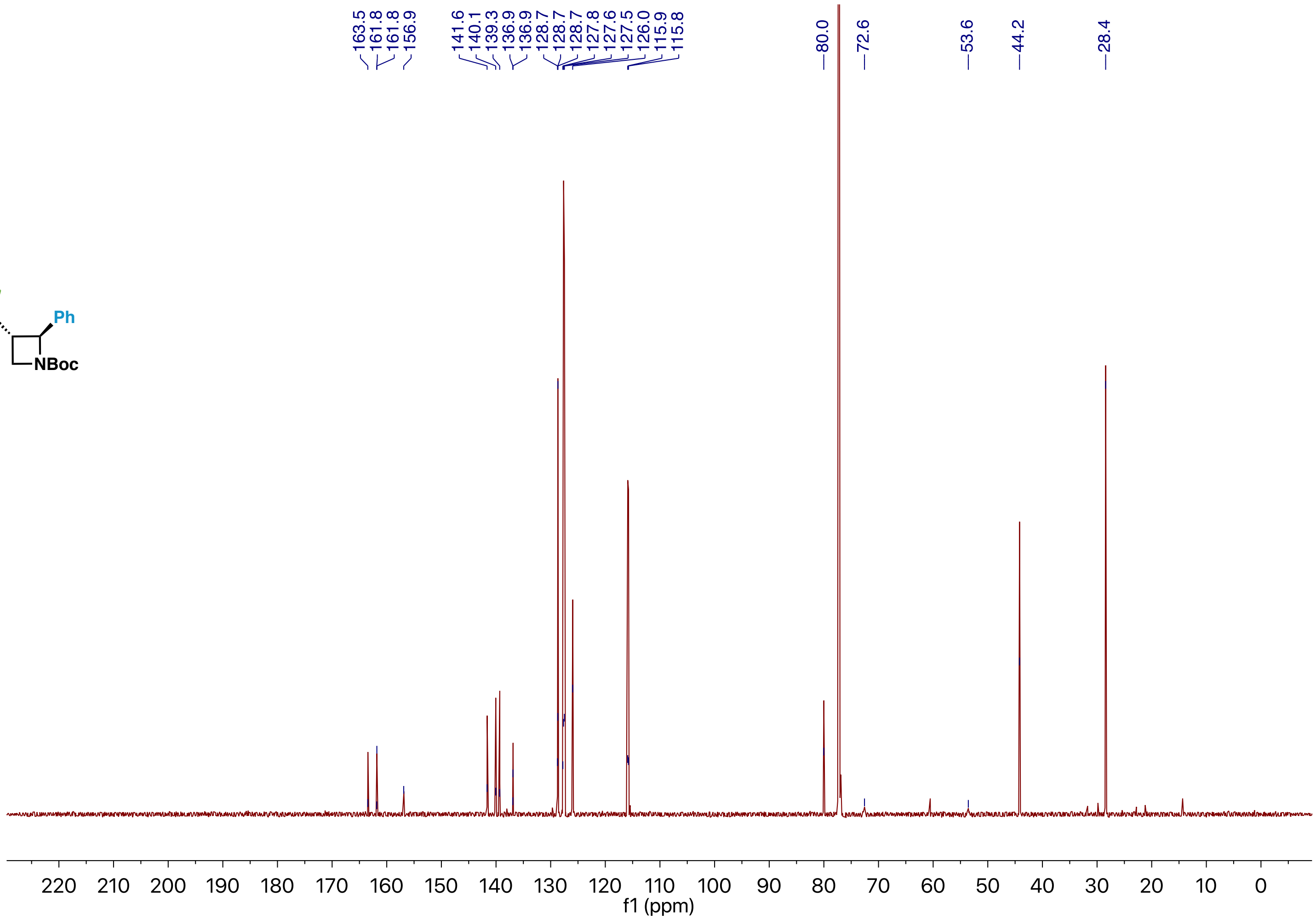

Compound 78 – ^{1}H NMR

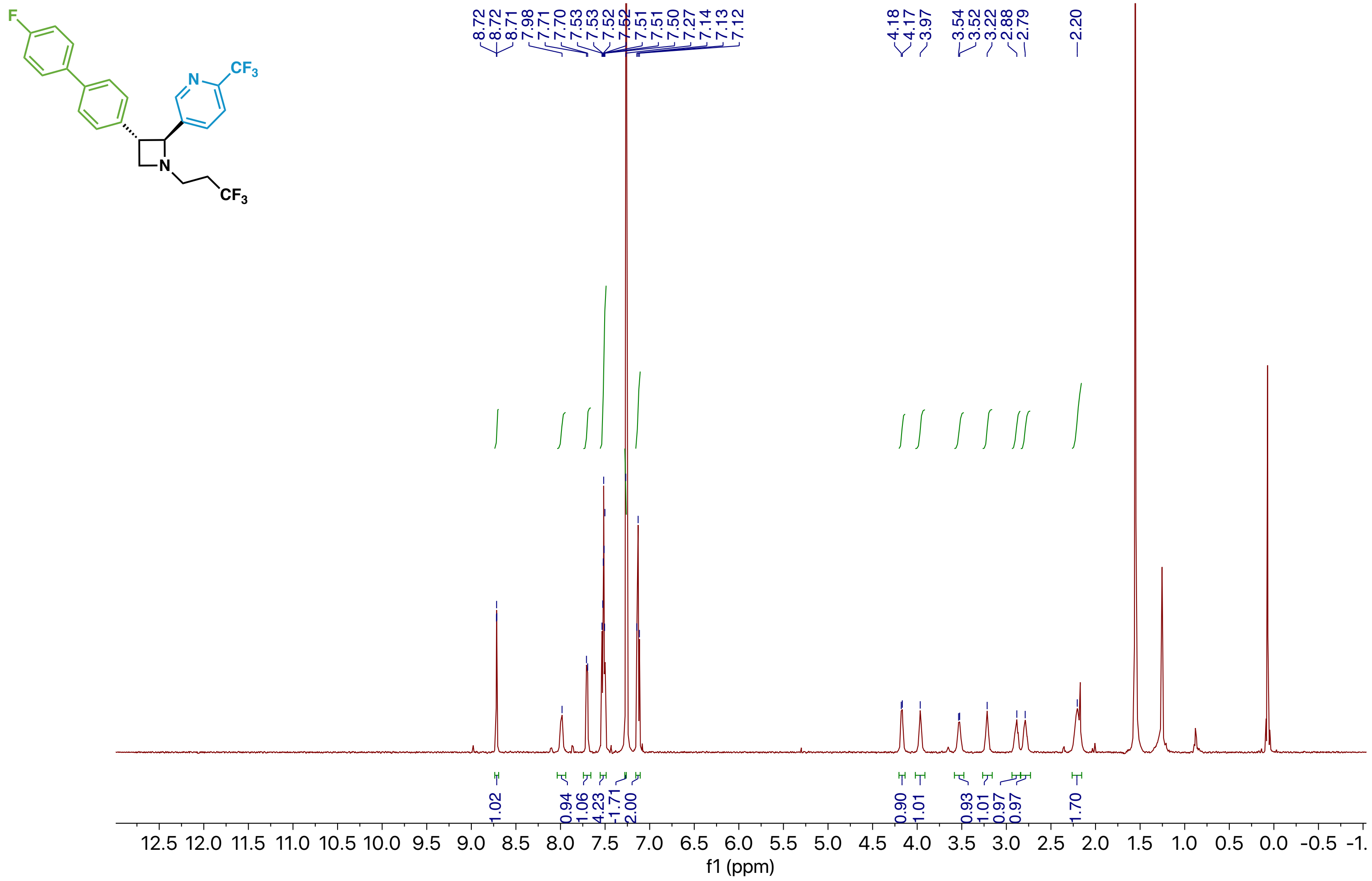

(Expanded region of compound 78 – ^{1}H NMR)

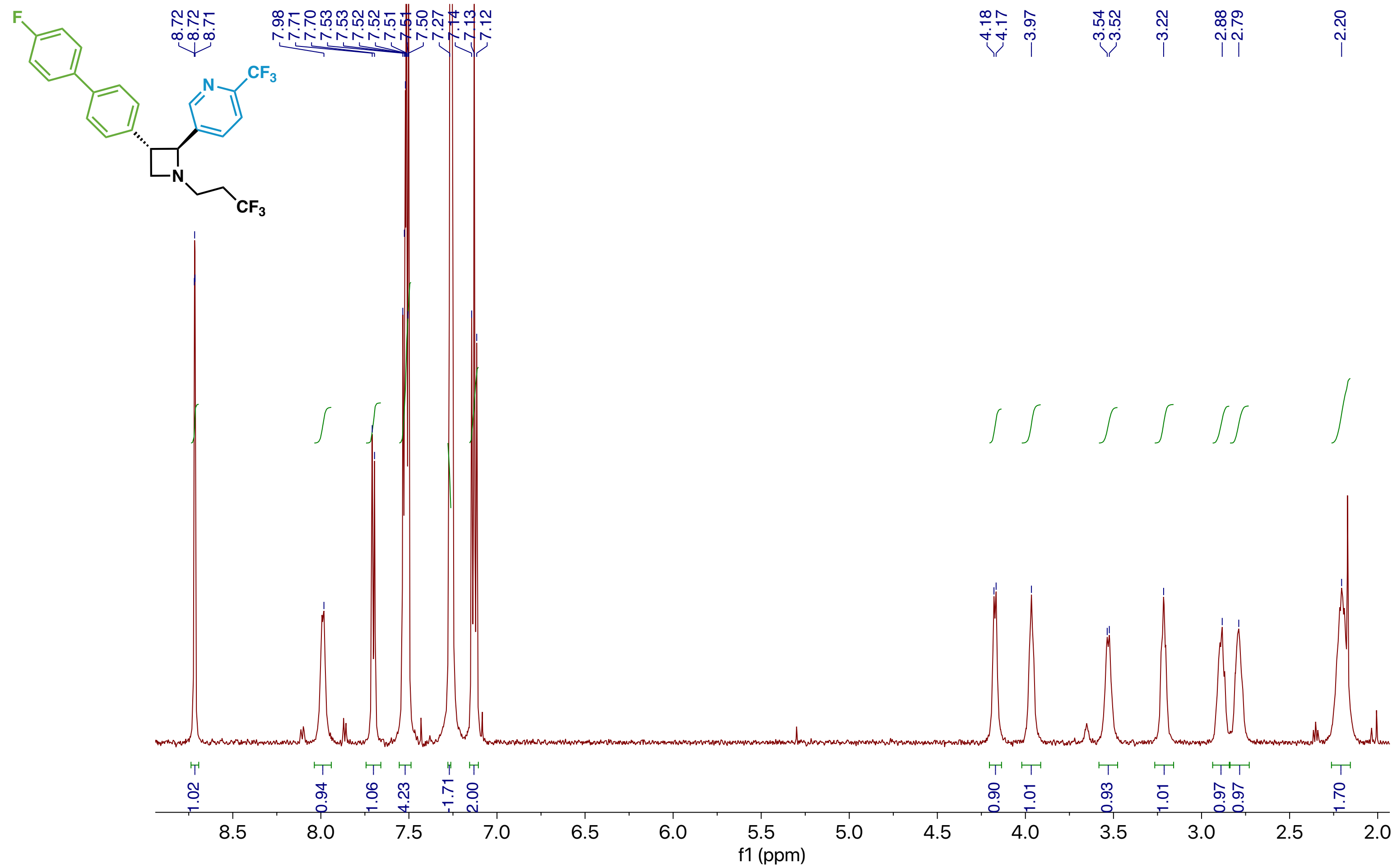

Compound 78 – ^{19}F NMR

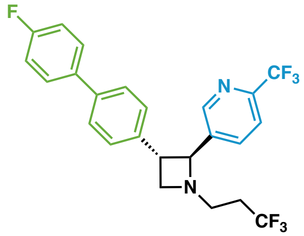

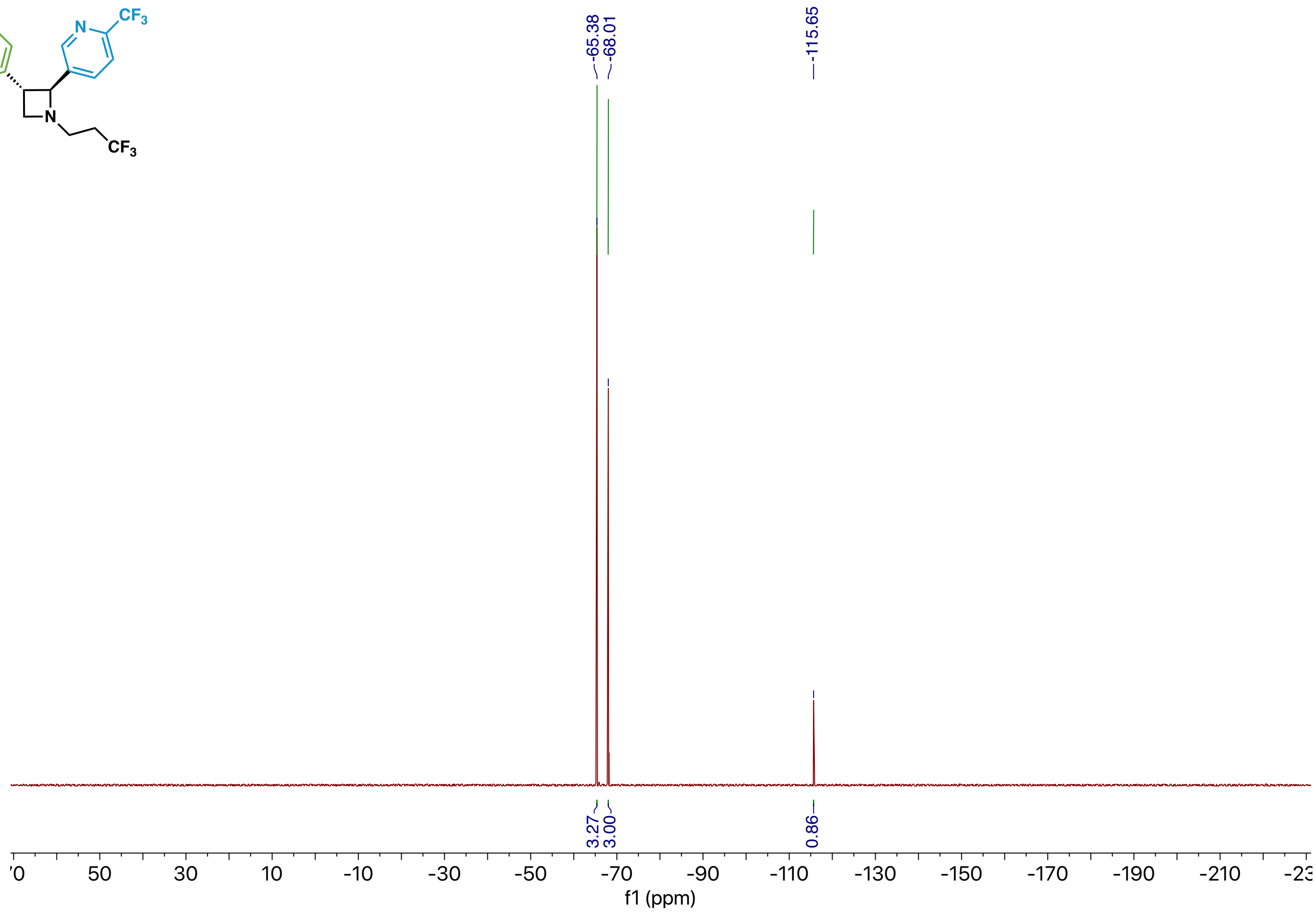

Compound 78 – ^{13}C NMR

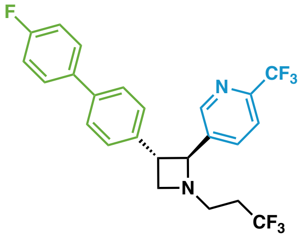

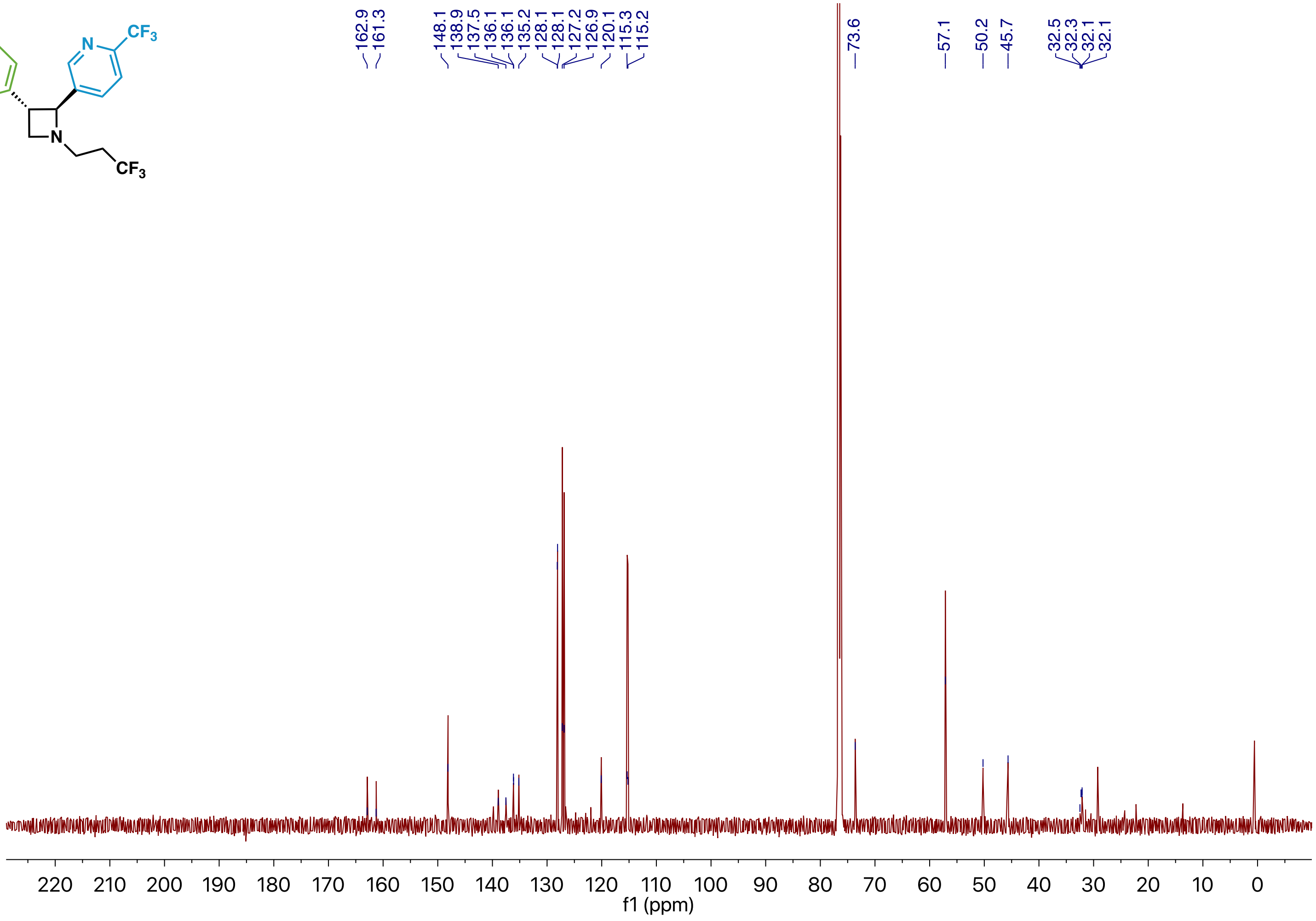

Compound 78 – HSQC

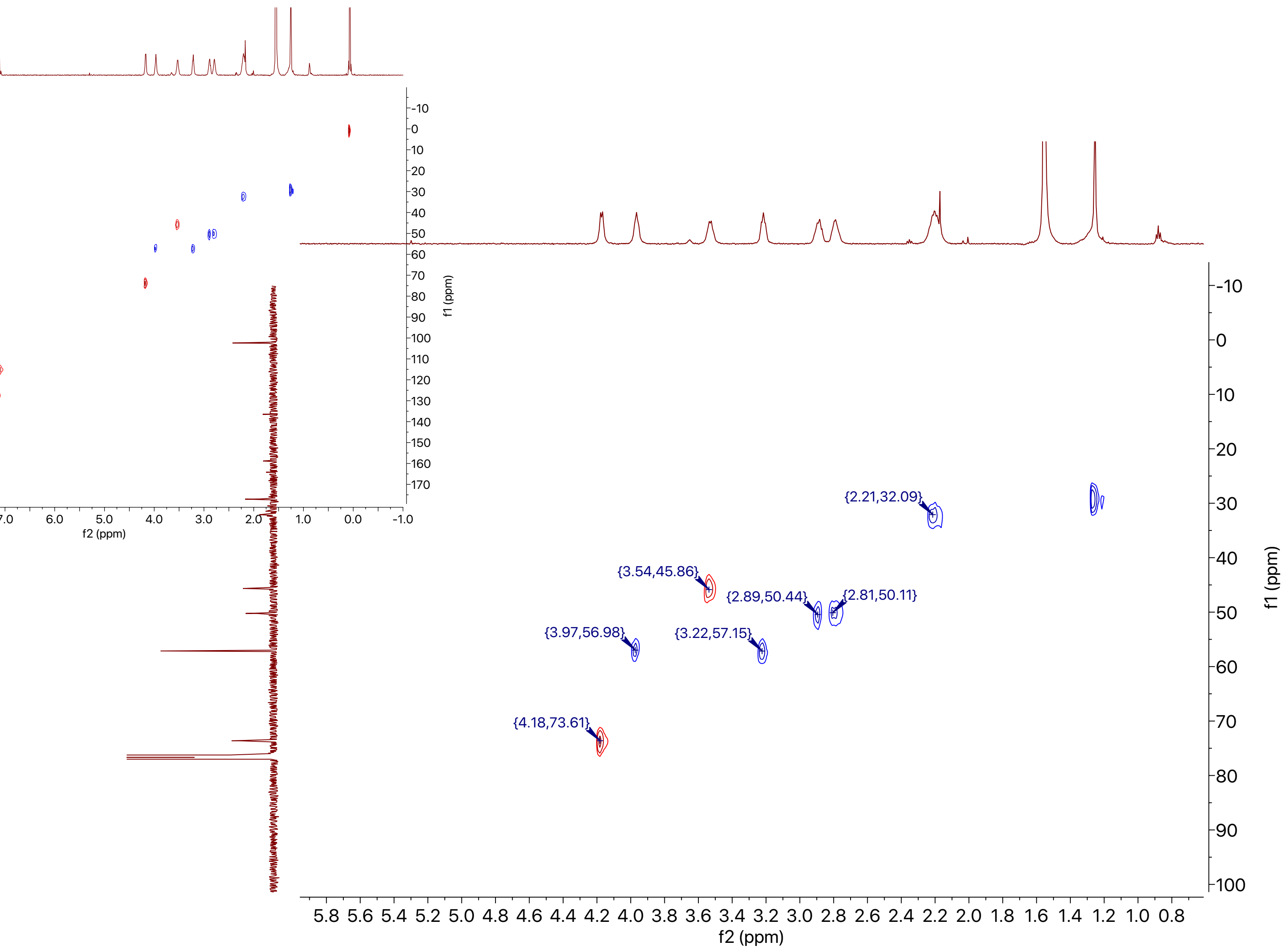

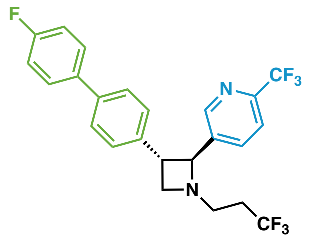

Compound SI-50 – ^{1}H NMR

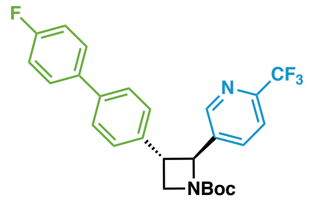

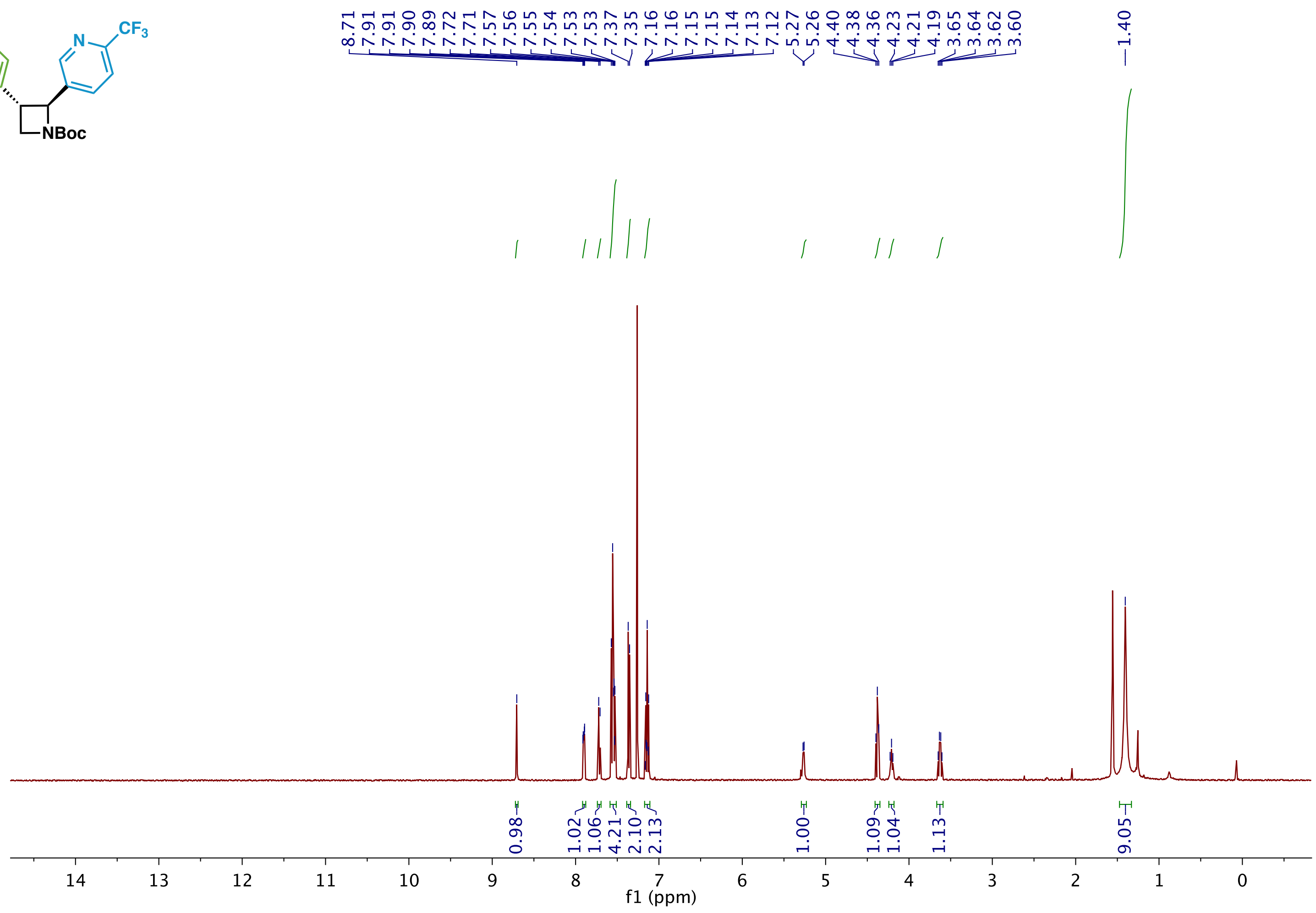

Compound SI-50 – ^{19}F NMR

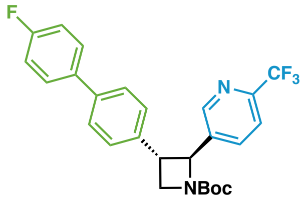

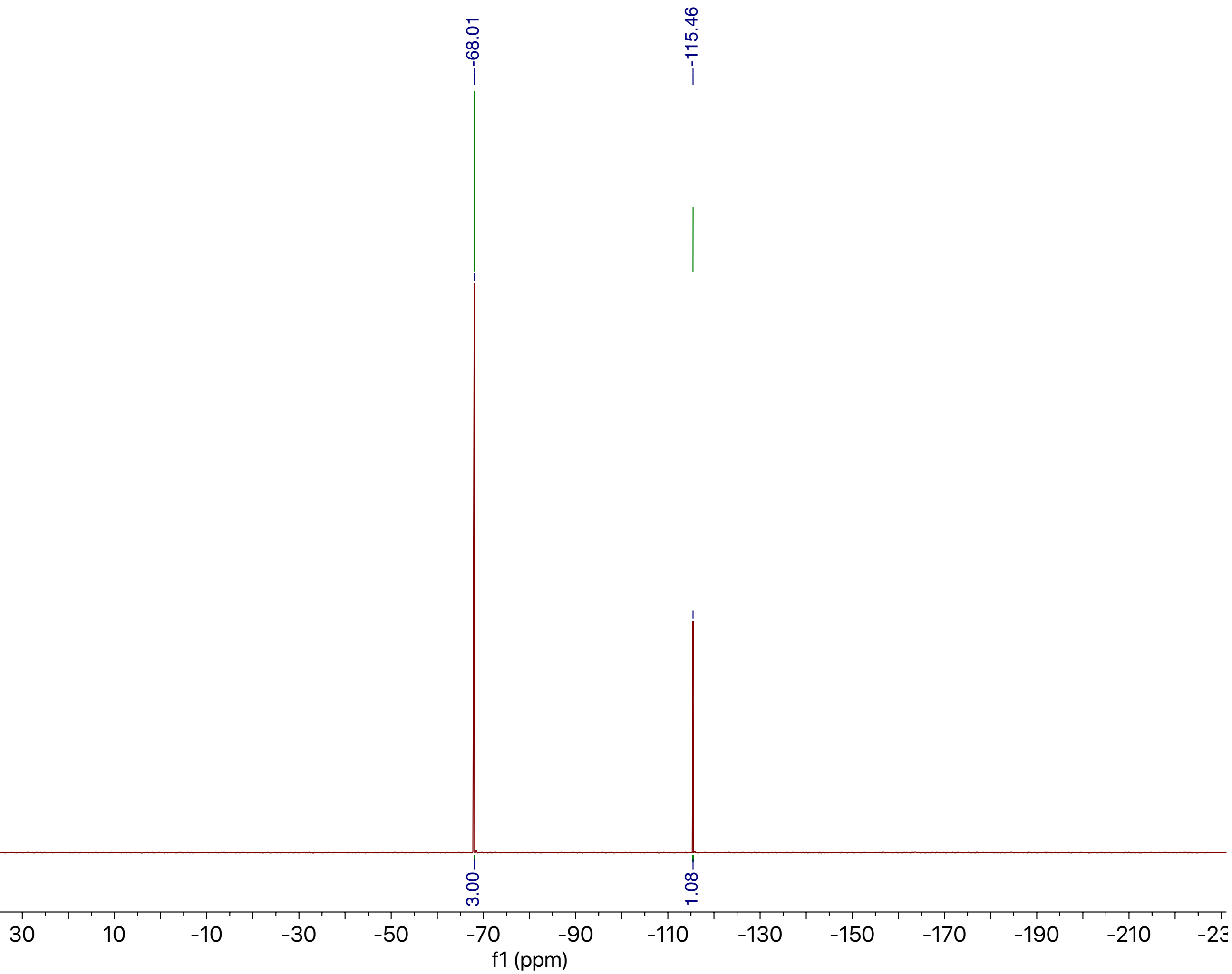

Compound SI-50 – ^{13}C NMR

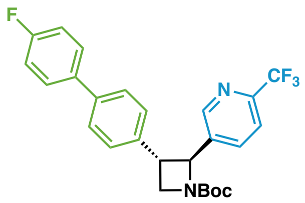

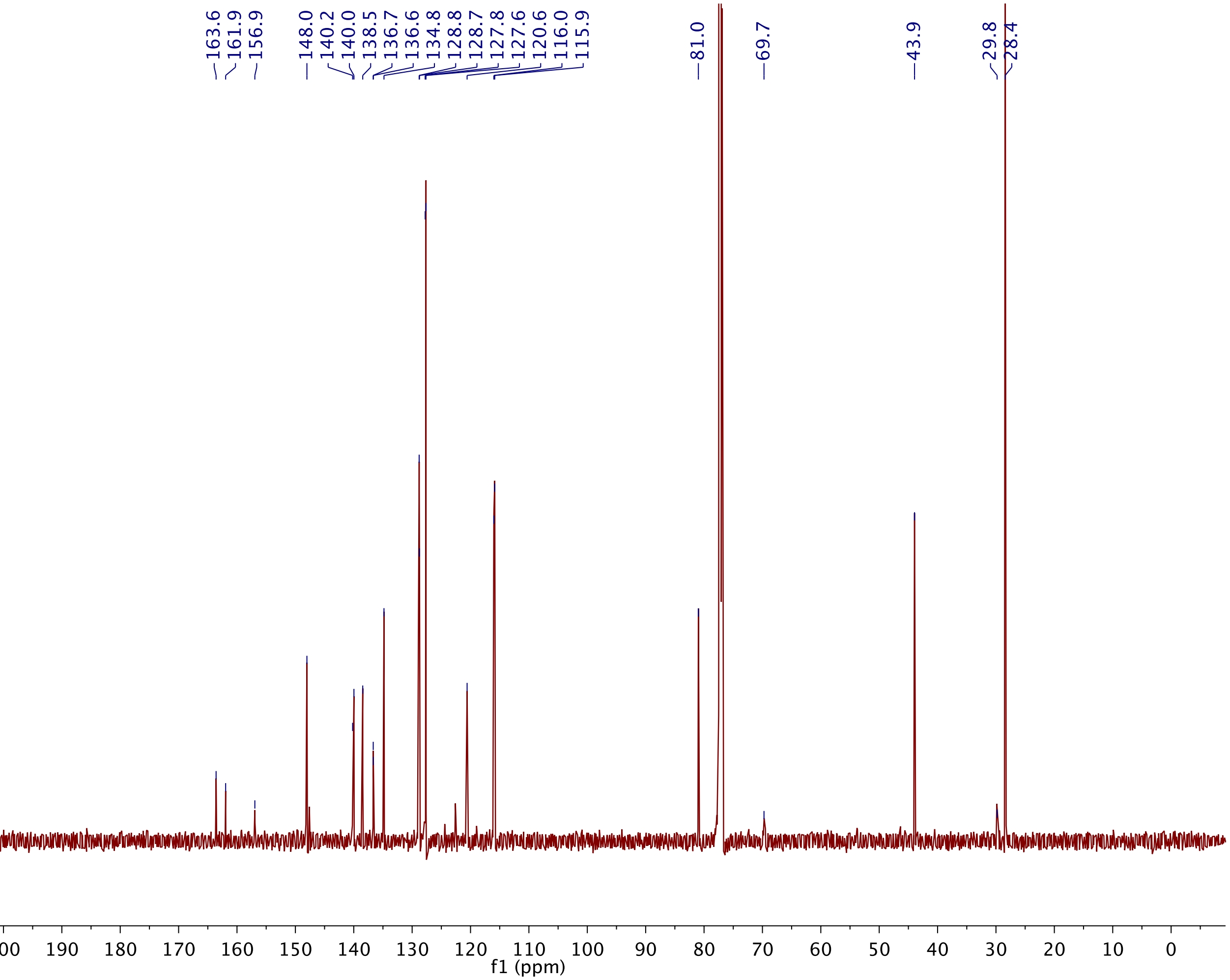

Compound 80 – ^{1}H NMR

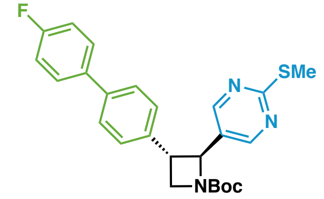

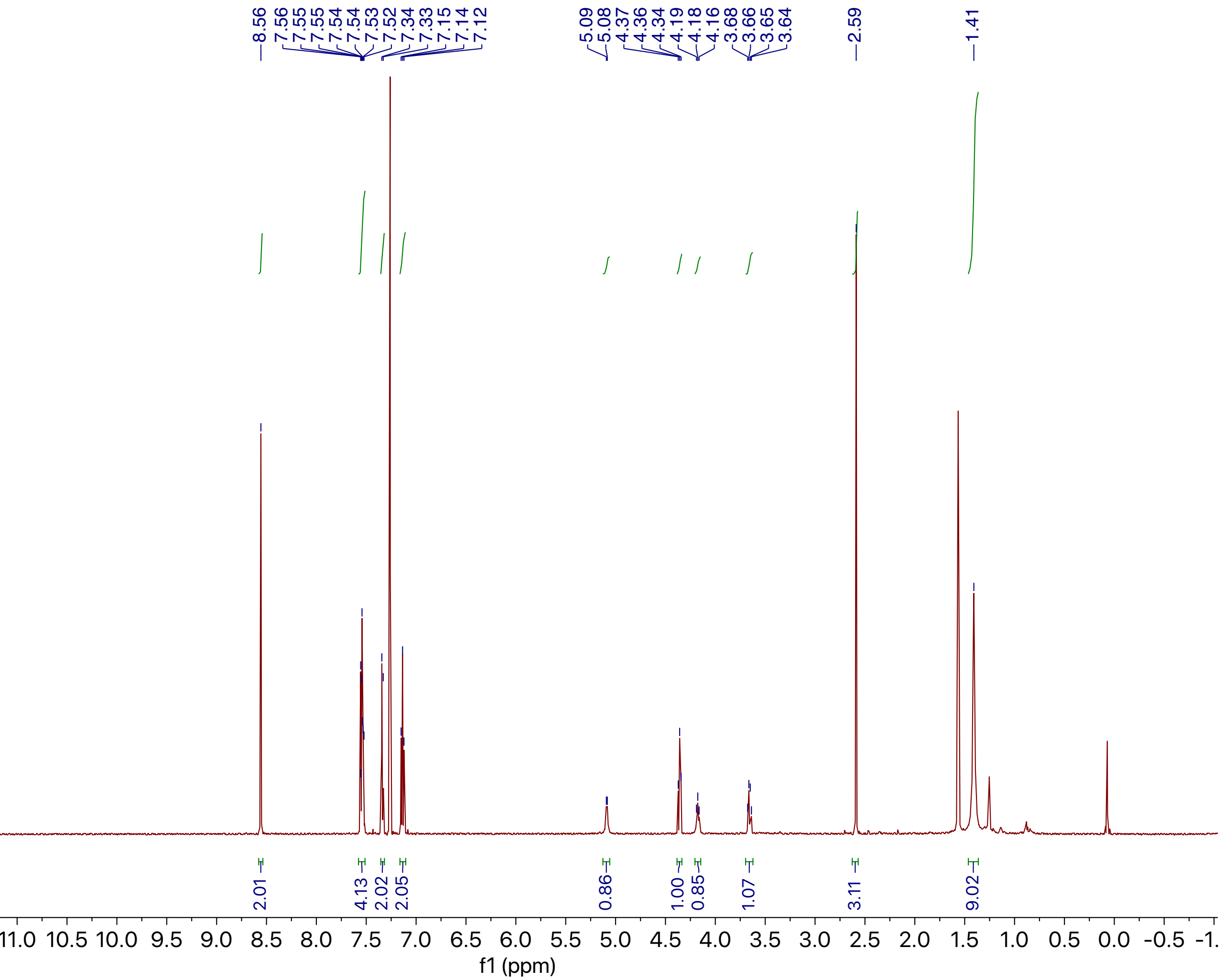

Compound 80 – ^{19}F NMR

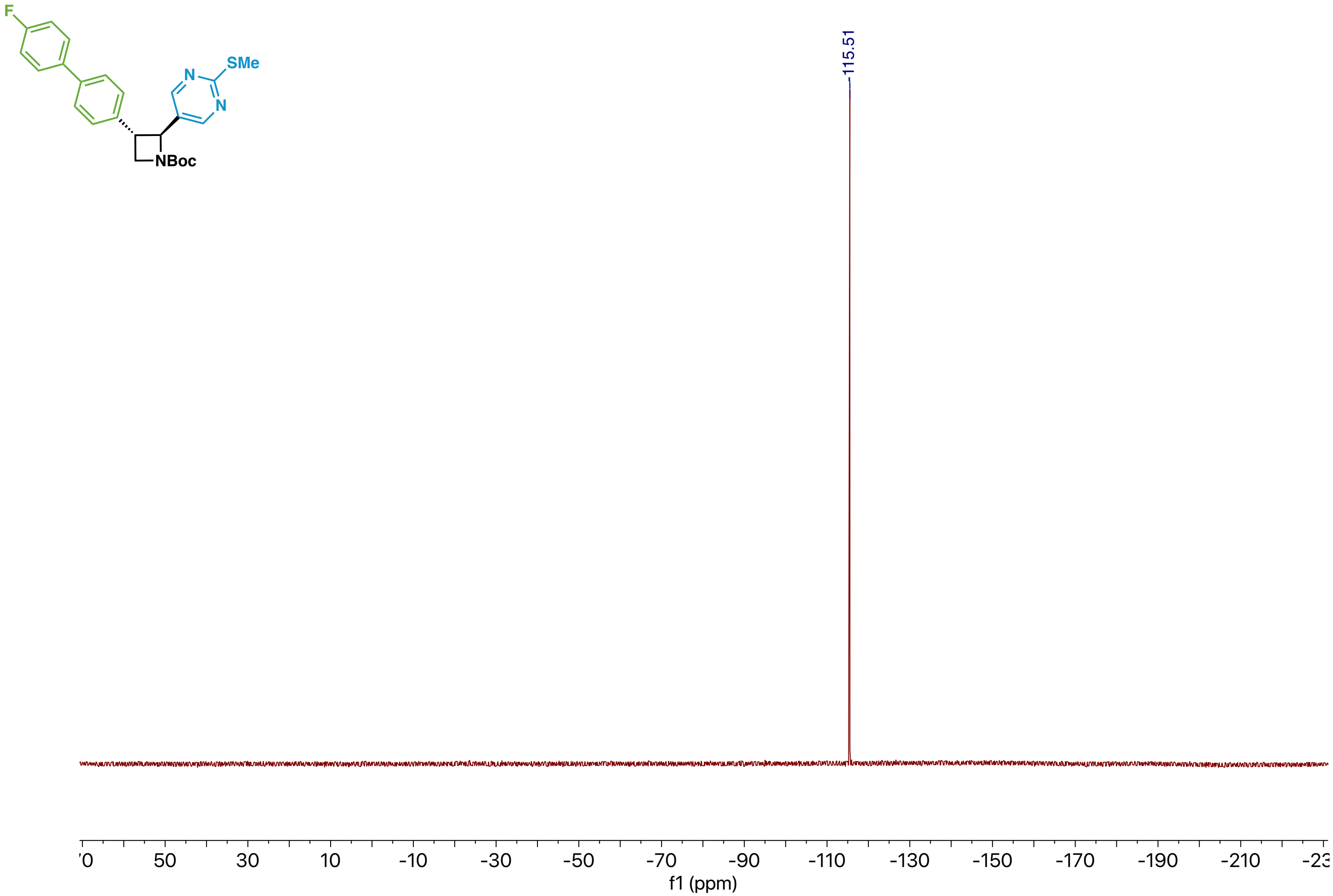

Compound 80 – ^{13}C NMR

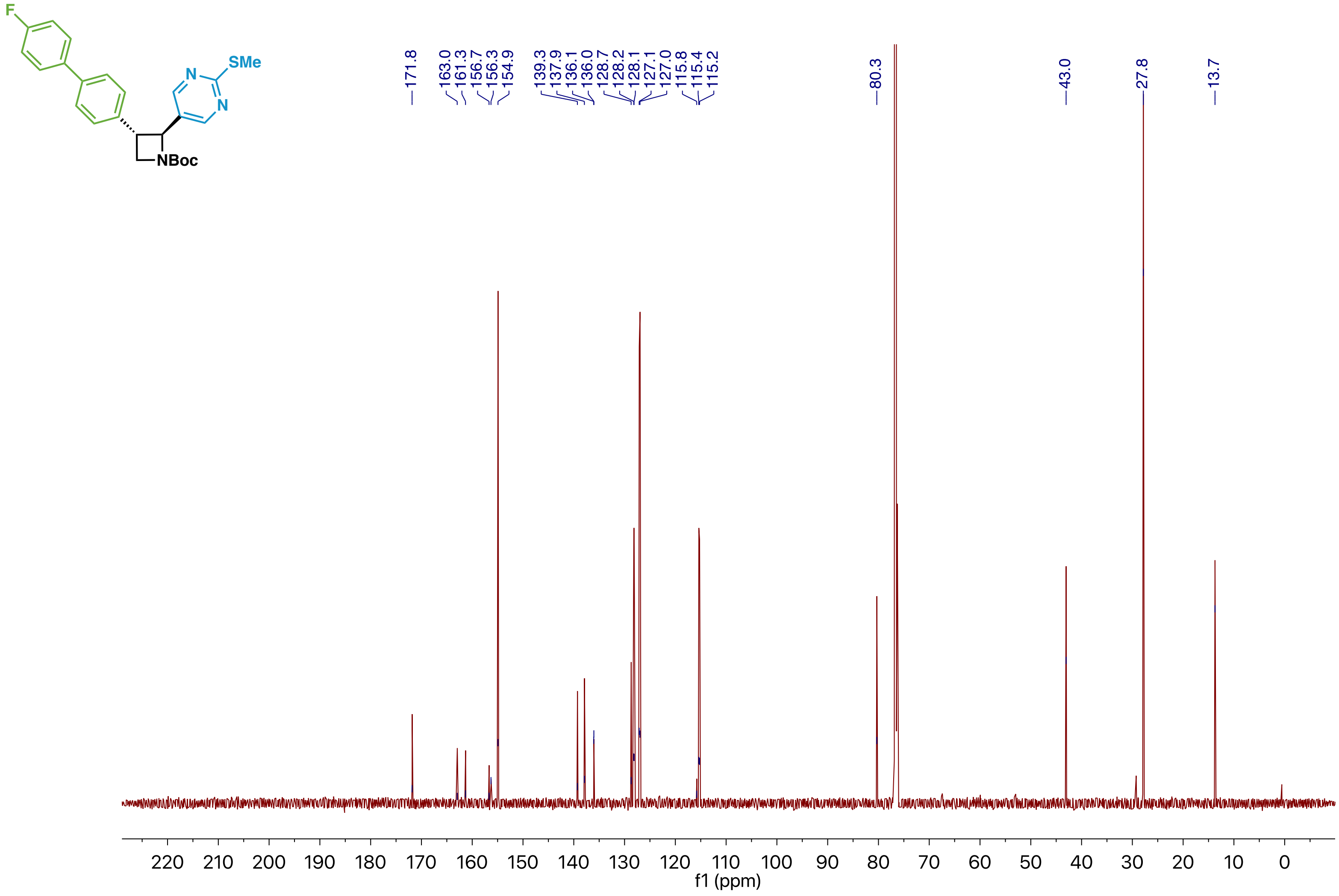

Compound 79– ^{1}H NMR

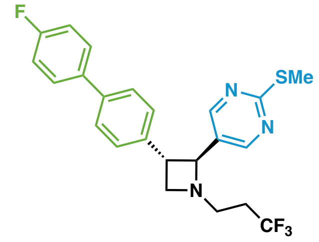

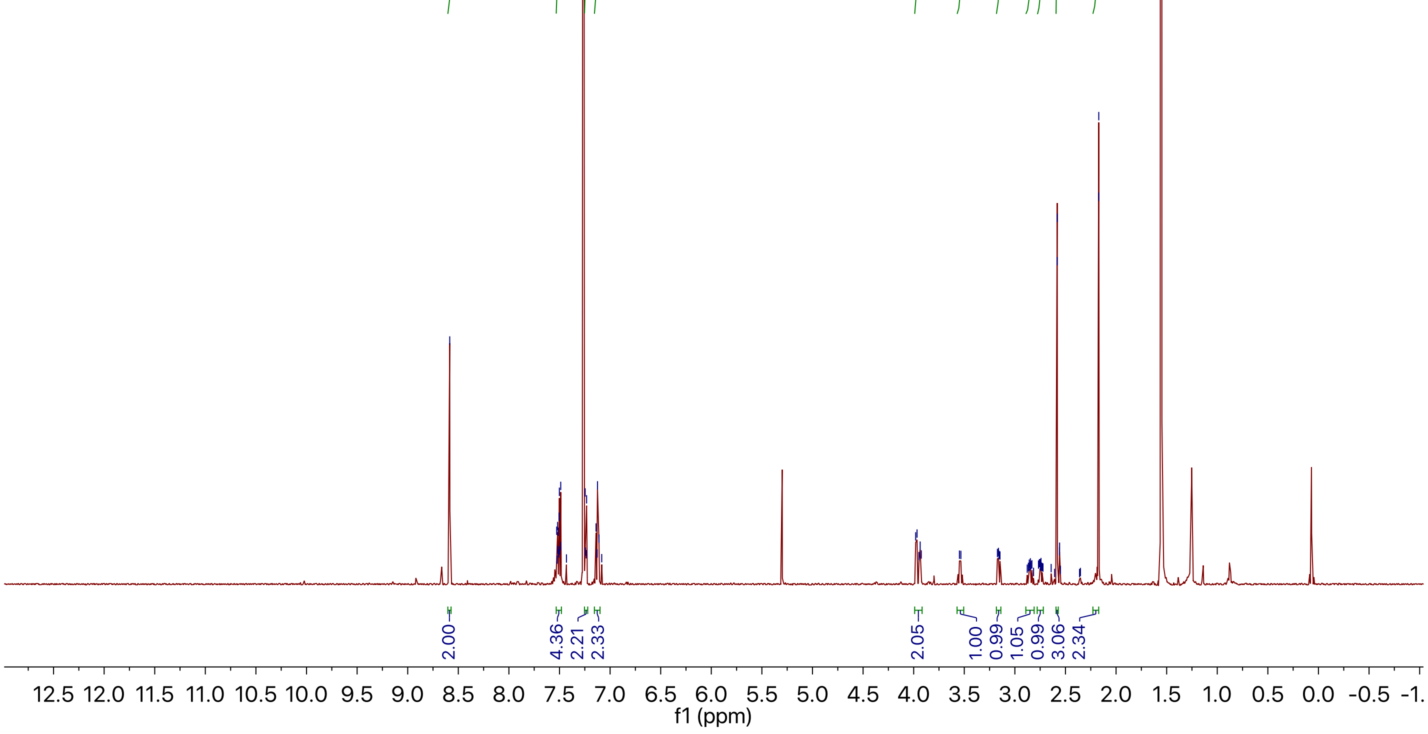

Compound 79 – ^{19}F NMR

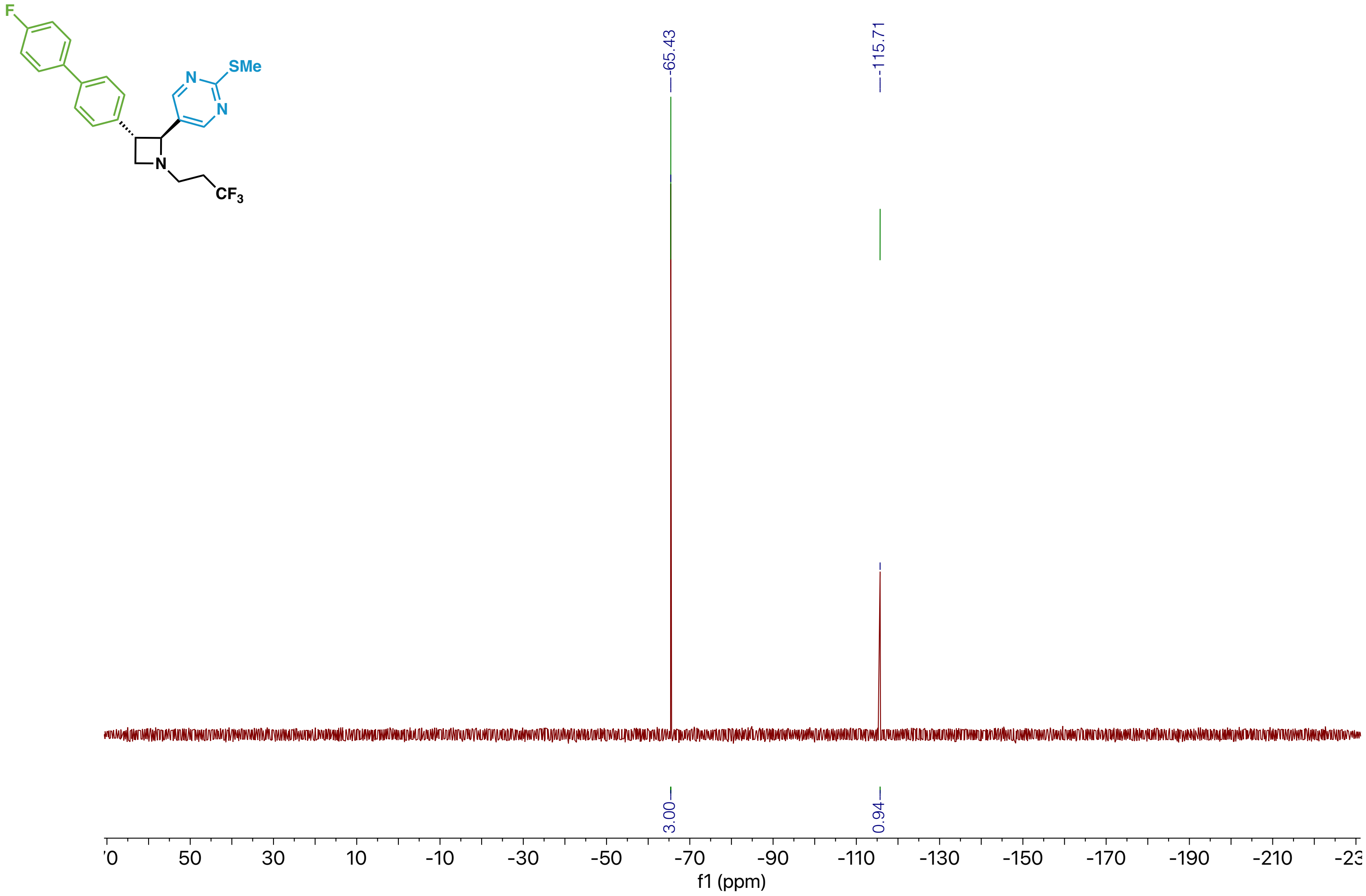

Compound 81 – ^{1}H NMR

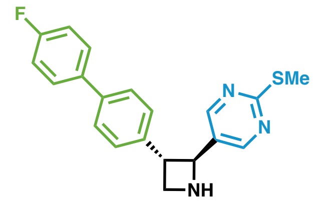

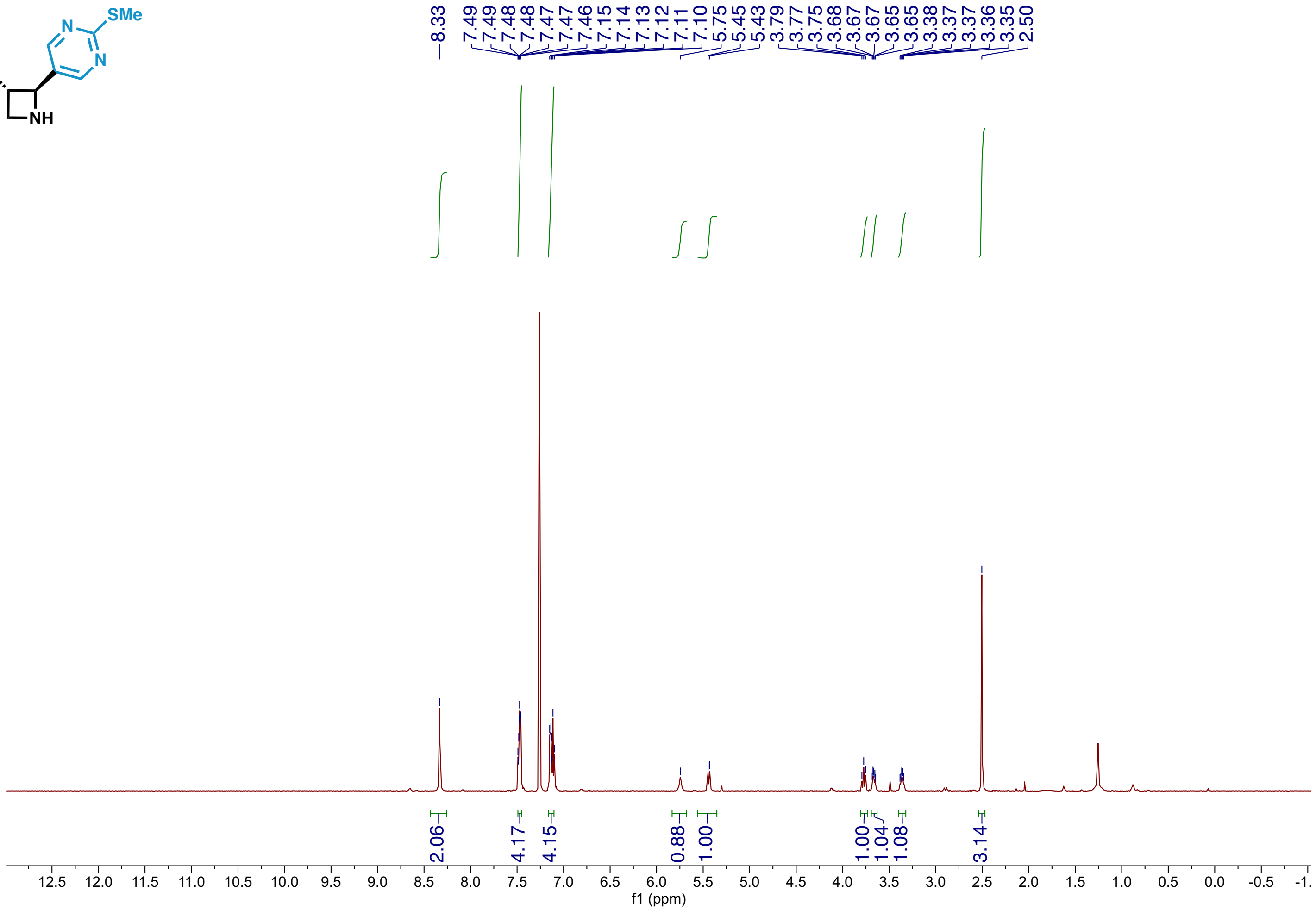

Compound 81 – ^{19}F NMR

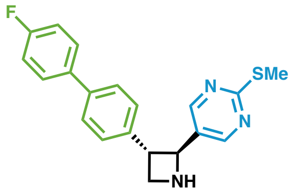

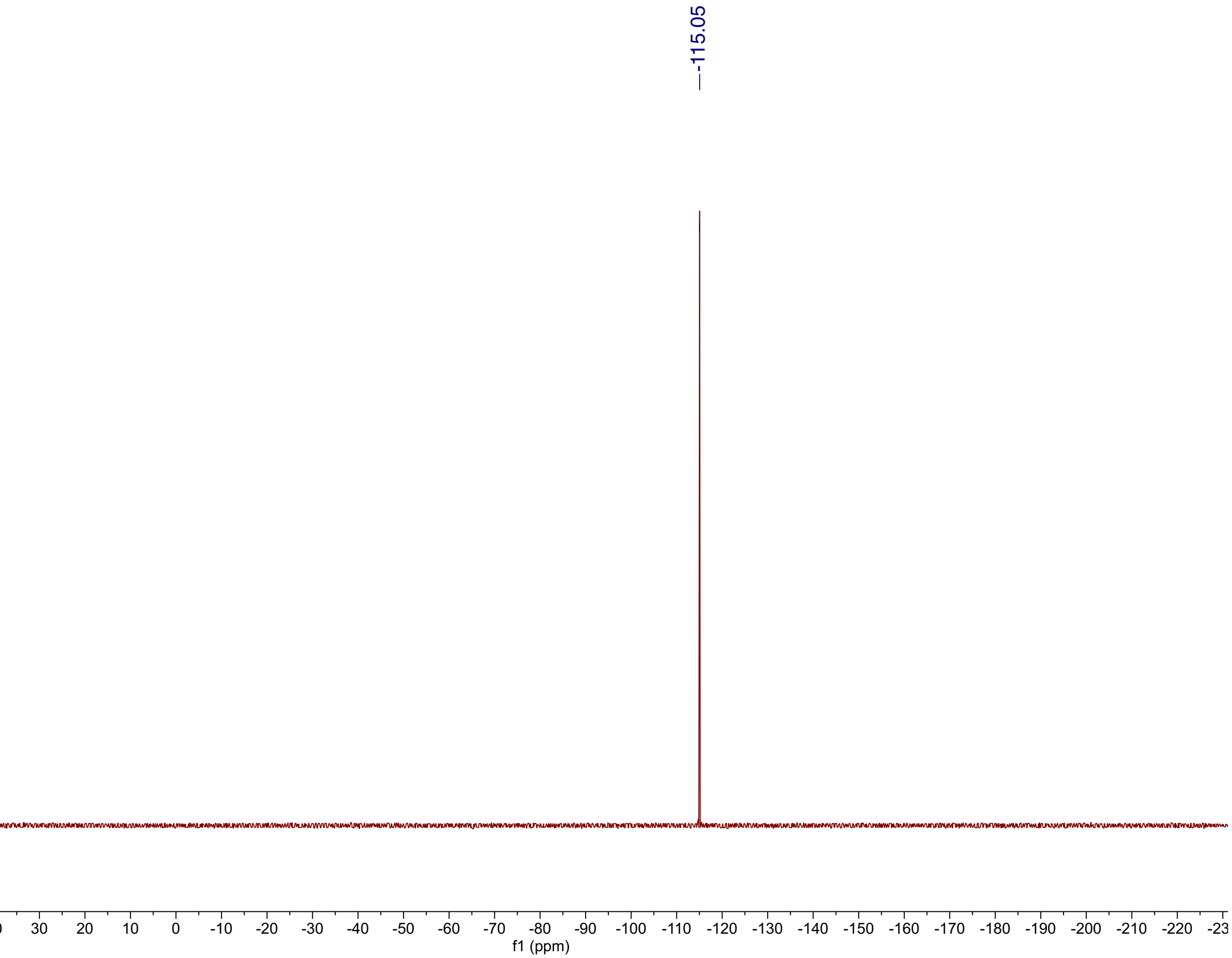

Compound 81 - ^{13}C NMR

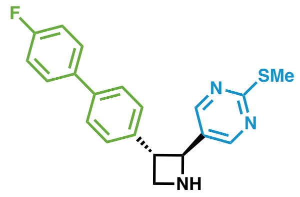

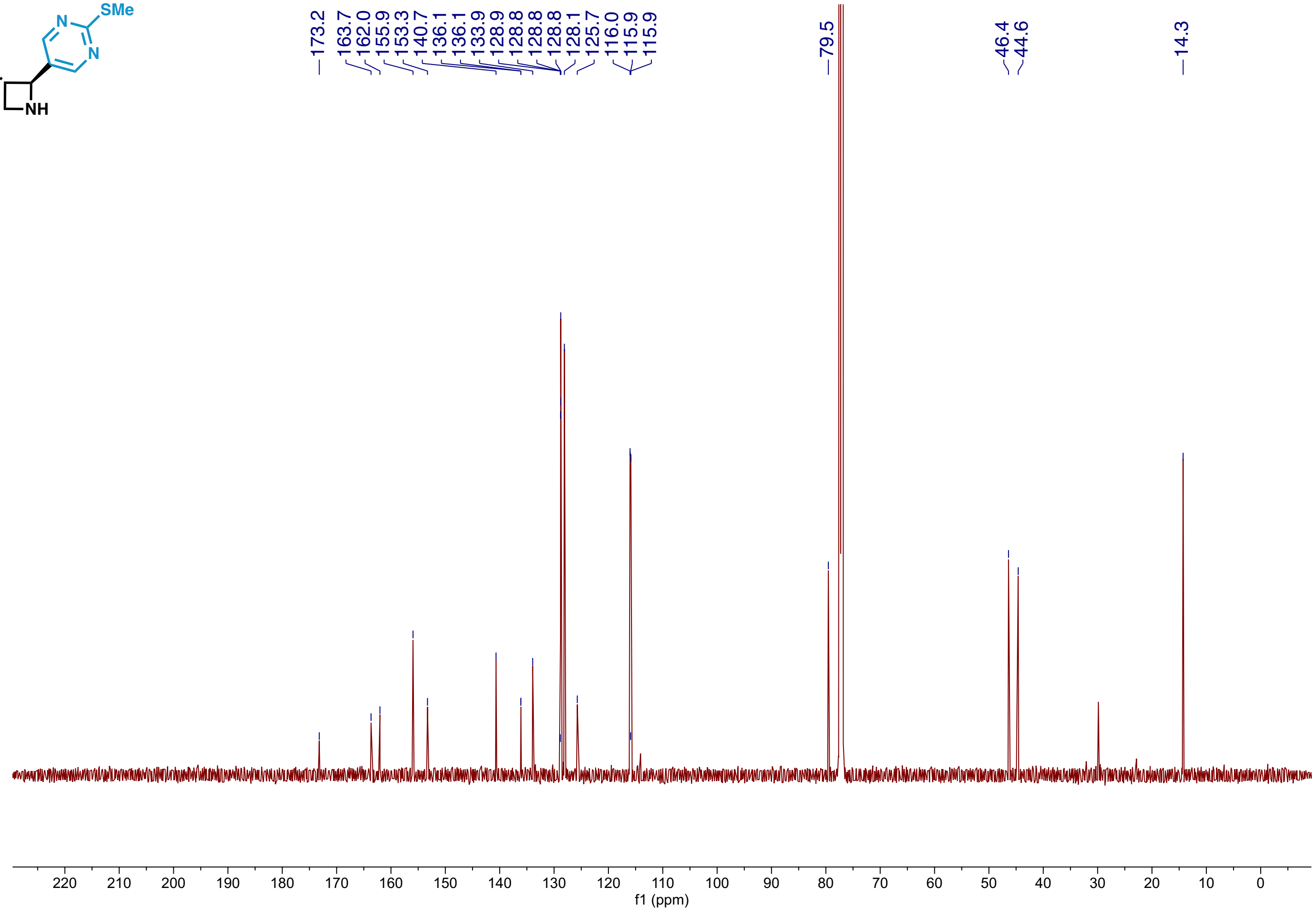

Compound 82 – ^{1}H NMR

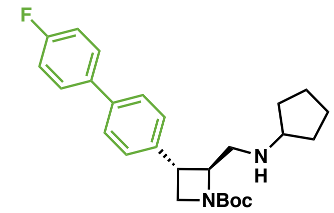

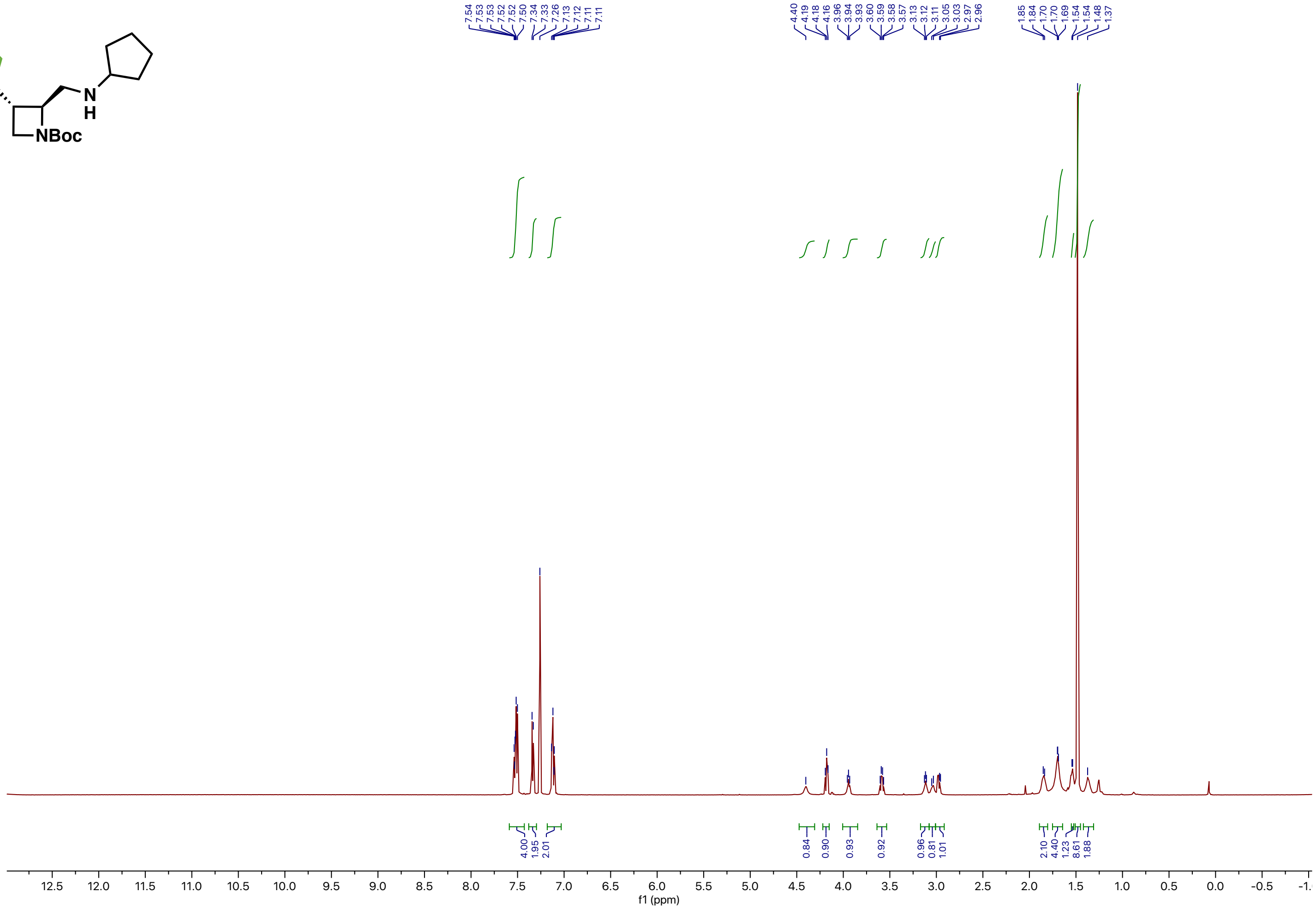

Compound 82 – ^{19}F NMR

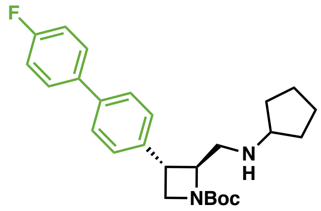

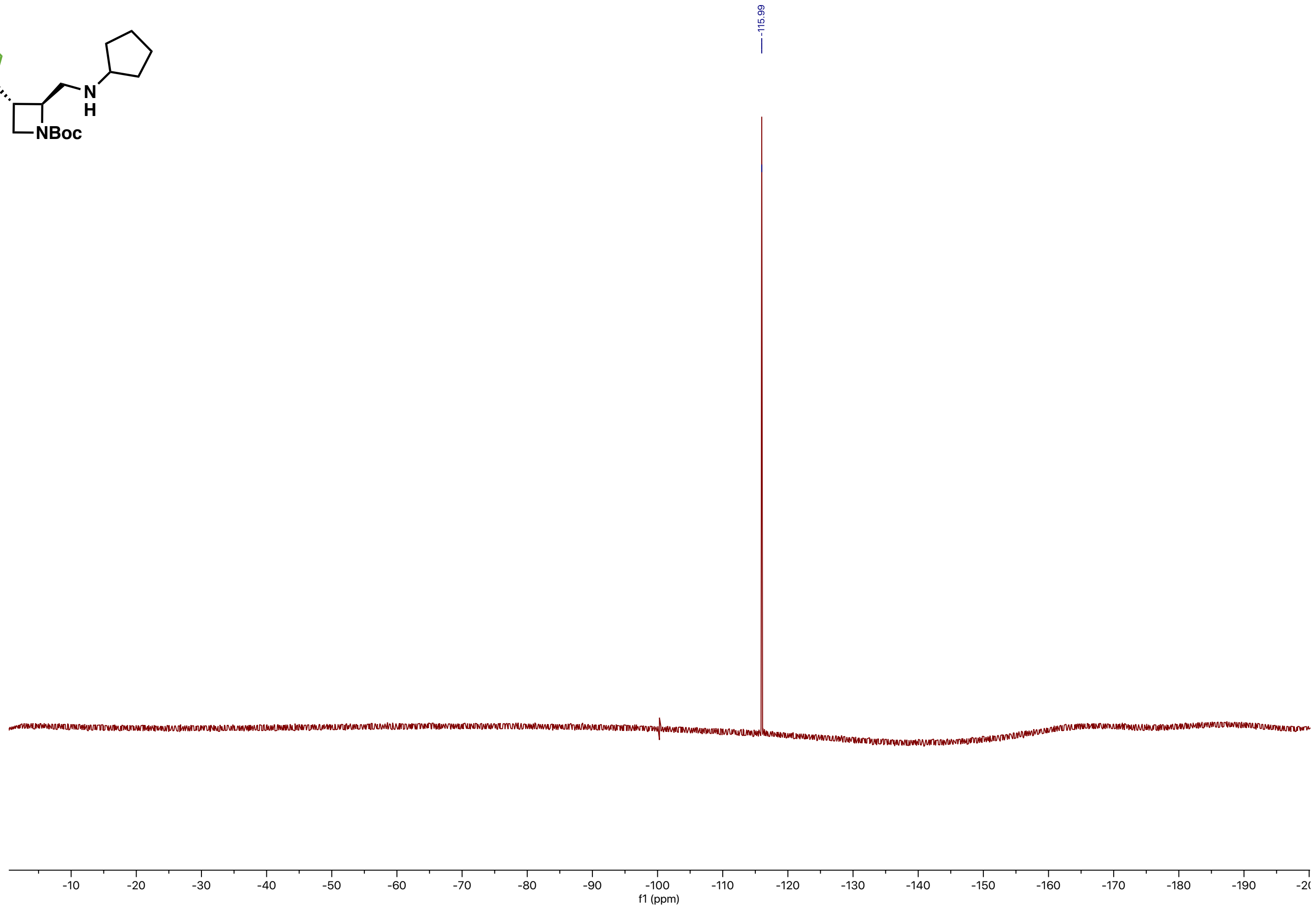

Compound 82 – ^{13}C NMR

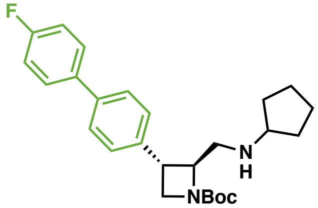

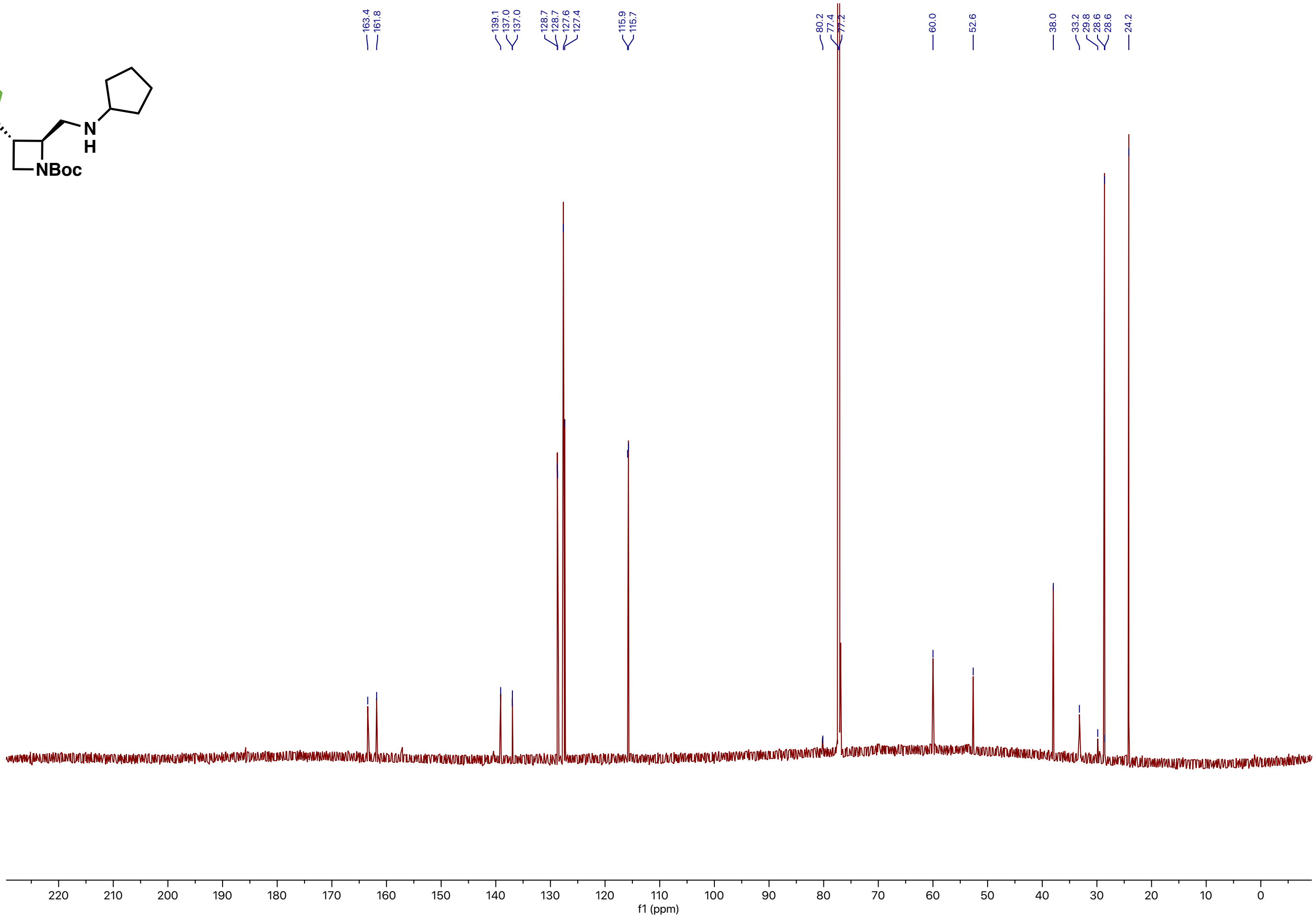

Compound 83 – ^{1}H NMR

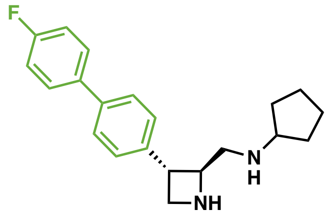

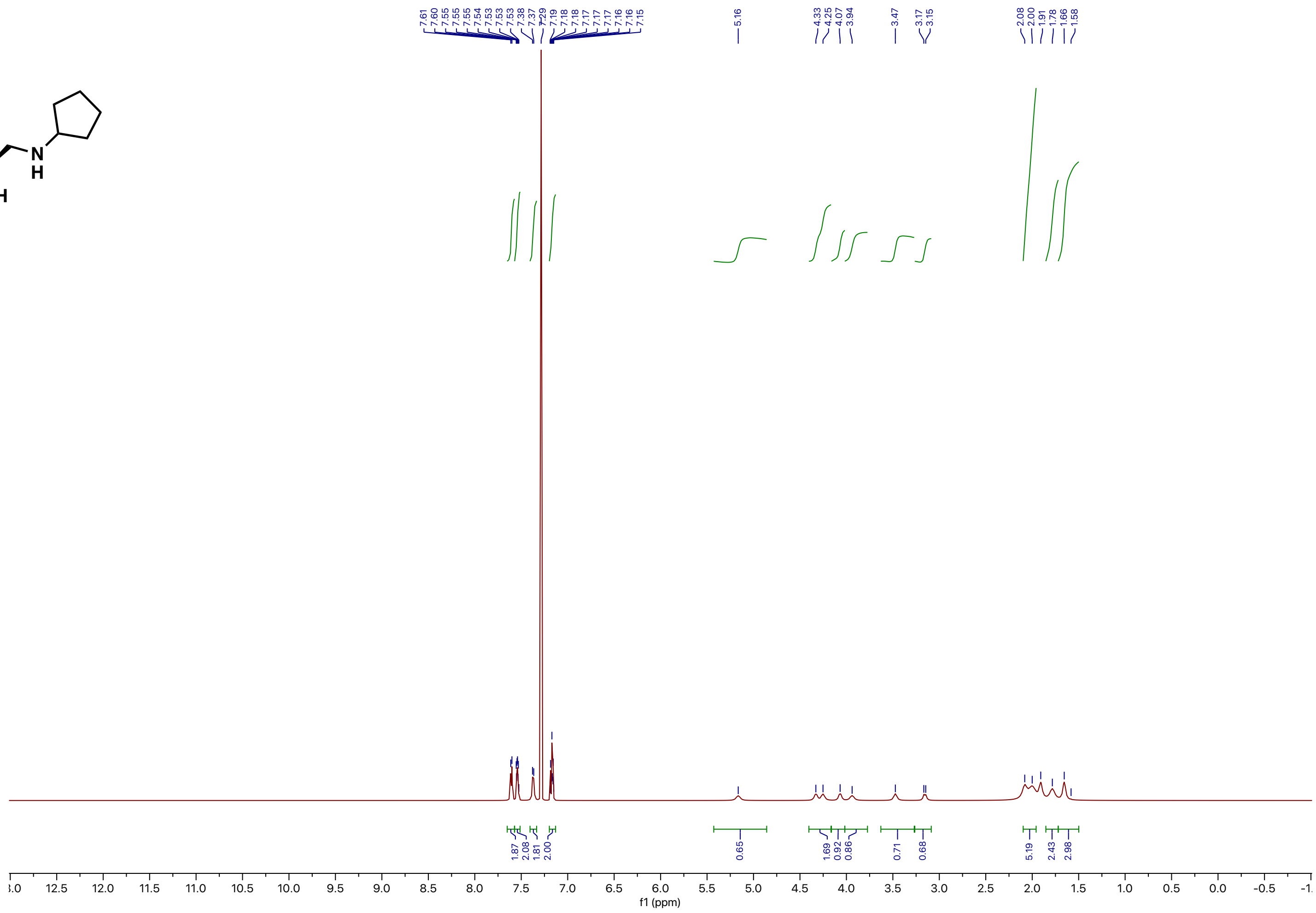

Compound 83 – ^{19}F NMR

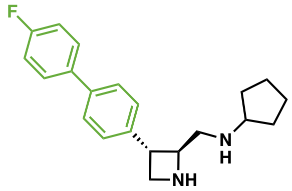

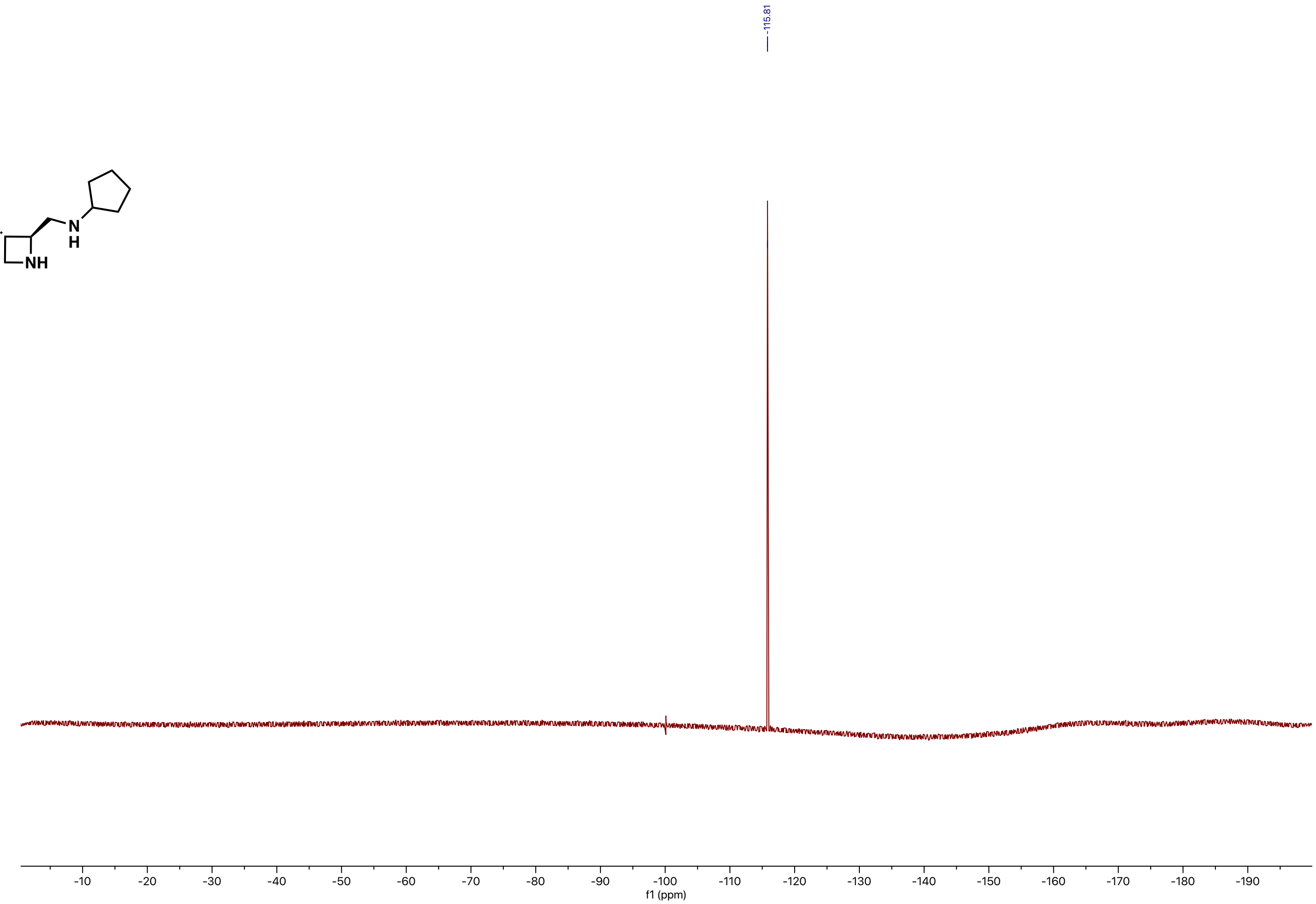

Compound 83 – ^{13}C NMR

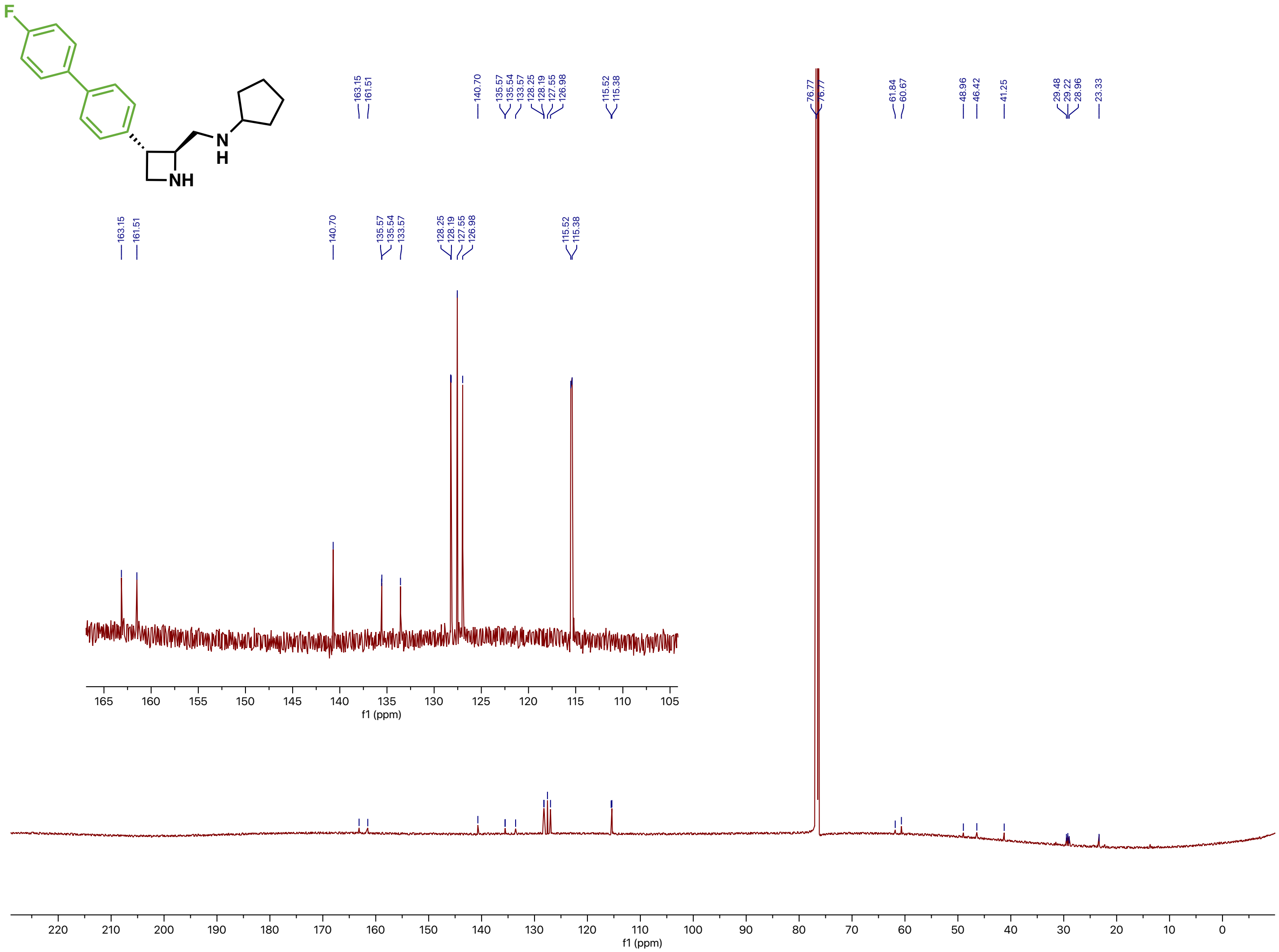

References

[1] (a) Yoo, E. J.; Ma, S.; Mei, T.-S.; Chan, K. S. L.; Yu, J.-Q. *J. Am. Chem. Soc*. **2011**, 133, 7652−7655. (b) Chan, K. S. L.; Wasa M.; Wang X.; Yu J.-Q. *Angew. Chem., Int. Ed*. **2011**, 50, 9081−9084.

[2] a) Chen, G.; Gong, W.; Zhe, Z.; Andra, M. S.; Chen, Y.-Q.; Hong, X.; Yang, Y.-F.; Liu, T.; Houk, K.N.; Yu, J.-Q. *Science*. **2016**, *353*, 1023−1027. b) Wu, Q.-F.; Wang, X.-B.; Shen, P.-X.; Yu, J.-Q. *ACS Catal*. **2018**, *8*, 2577−2581.

[3] Shen, P.-X.; Hu, L.; Shao, Q.; Hong, K.; Yu, J.-Q. *J. Am. Chem. Soc.* **2018**, *140*, 6545–6549.

[4] Maetani, M.; Zoller, J.; Melillo, B.; Verho, O.; Kato, N.; Pu, J.; Comer, E.; Schreiber, S. L. *J. Am. Chem. Soc.* **2017**, 139, 32, 11300.

[5] Affron, D. P; Bull, J. A. *Eur. J. Org. Chem.* **2016**, 139–149.

[6] Toriyama, F.; Cornella, J.; Wimmer, L.; Chen, T.-G.; Dixon, D. D.; Creech, G.; Baran, P. S. *J. Am. Chem. Soc*. **2016**, 138, 11132−11135.

[7] Wang, J.; Qin, T.; Chen, T-G; Wimmer, L.; Edwards, J. T.; Cornella, J.; Vokits, B.; Shaw, S. A.; Baran, P. S. *Angew. Chem. Int. Ed*. **2016**, 55, 9676–9679.

[8] Qin, T.; Cornella, J.; Li, C.; Malins, L. R.; Edwards, J. T.; Kawamura, S.; Maxwell, B. D.; Eastgate, M. D.; Baran, P. S. *Science* **2016**, 352, 801−805.

[9] Edwards, J. T.; Merchant, R. R.; McClymont, K. S.; Knouse, K. W.; Qin, T.; Malins, L. R.; Vokits, B.; Shaw, S. A.; Bao, D.-H.; Wei, F.-L.; Zhou, T.; Easgate, M. D.; Baran, P. S. *Nature* **2017**, 545, 213–218.

[10] Sämann, C.; Schade, M. A.; Yamada, S.; Knochel, P. Functionalized Alkenylzinc. *Angew. Chem. Int. Ed*. **2013**, 52, 9495−9499.

[11] Cornella, J.; Edwards, J. T.; Qin, T.; Kawamura, S.; Wang, J.; Pan, C. M.; Gianatassio, R.; Schmidt, M.; Eastgate, M. D.; Baran, P. S. *J. Am. Chem. Soc*. **2016,** 138, 2174−2177.

[12] Boymond, L.; Rottländer, M.; Cahiez, G.; Knochel, P. *Angew. Chem. Int. Ed*. **1998**, 37, 1701−1703.

[13] Smith, J. M.; Qin, T.; Merchant, R. R.; Edwards, J. T.; Malins, L. R.; Liu, Z.; Che, G.; Shen, Z.; Shaw, S. A.; Eastgate, M. D.; Baran, P. S. *Angew. Chem. Int. Ed*. **2017**, 56, 11906−11910.

[14] Li, C.; Wang, J.; Barton, L. M.; Yu, S.; Tian, M.; Peters, D. S.; Kumar, M.; Yu, A. W.; Johnson, K. A.; Chatterjee, A. K.; Yan, M.; Baran, P. S. *Science* **2017**, 356, eaam7355.

[15] Qin, T.; Malins, L. R.; Edwards, J. T.; Merchant, R. R.; Novak, A. J. E.; Zhong, J. Z.; Mills, R. B.; Yan, M.; Yuan, C.; Eastgate, M. D.; Baran, Phil S. *Angew. Chem. Int. Ed*. **2017**, 56, 260–265.

[16] a) Wirth, H. O.; Königstein, O.; Kern, W. *Justus Liebigs Ann. Chem*. **1960**, 634, 84 − 104. b) Dewar, M. J. S.; Marchand, A. P. *J. Am. Chem. Soc.* **1966**, 88, 3318−3327. c) Wrobel, J.; Dietrich, A.; Gorham, B. J.; Sestanj, K. *J. Org. Chem*. **1990**, 55, 2694−2702.

17 a) Yee*,* N. K. A. Practical One-Pot Synthesis of *trans*-4,5-Disubstituted 2-Pyrrolidinones and The Related Pyrrolidines. *Tetrahedron Letters*, **1997**, *38*, 5091−5093. b) Yee, N. K.; Nummy, L. J.; Byrne, D. P.; Smith, L. L.; Roth, G. P. Practical Synthesis of an Enantiomerically Pure *trans*-4,5-Disubstituted 2-Pyrrolidinone via Enzymatic Resolution. Preparation of the LTB4 Inhibitor BIRZ-227. *J. Org. Chem.* **1998**, *63*, 326−330.
